# Comparative genomic analysis of eutherian connexin genes

**DOI:** 10.1038/s41598-019-53458-x

**Published:** 2019-11-15

**Authors:** Marko Premzl

**Affiliations:** The Australian National University Alumni, Zagreb, Croatia

**Keywords:** Comparative genomics, Genome evolution

## Abstract

The eutherian connexins were characterized as protein constituents of gap junctions implicated in cell-cell communications between adjoining cells in multiple cell types, regulation of major physiological processes and disease pathogeneses. However, conventional connexin gene and protein classifications could be regarded as unsuitable in descriptions of comprehensive eutherian connexin gene data sets, due to ambiguities and inconsistencies in connexin gene and protein nomenclatures. Using eutherian comparative genomic analysis protocol and 35 public eutherian reference genomic sequence data sets, the present analysis attempted to update and revise comprehensive eutherian connexin gene data sets, and address and resolve major discrepancies in their descriptions. Among 631 potential coding sequences, the tests of reliability of eutherian public genomic sequences annotated, in aggregate, 349 connexin complete coding sequences. The most comprehensive curated eutherian connexin gene data set described 21 major gene clusters, 4 of which included evidence of differential gene expansions. For example, the present gene annotations initially described human *CXNK1* gene and annotated 22 human connexin genes. Phylogenetic tree calculations and calculations of pairwise nucleotide sequence identity patterns proposed revised and updated phylogenetic classification of eutherian connexin genes. Therefore, the present study integrating gene annotations, phylogenetic analysis and protein molecular evolution analysis proposed new nomenclature of eutherian connexin genes and proteins.

## Introduction

The eutherian connexins were characterized as protein constituents of gap junctions that were implicated in cell-cell communications between adjoining cells in multiple cell types, tissues and organs by means of passage of ions and small molecules^1–4^. Such intercellular interactions were also implicated in regulation of major physiological processes including apoptosis, development, differentiation and maintenance of tissue homeostasis, as well as in human disease pathogeneses including familial zonular pulverulent cataracts, nonsyndromic and syndromic deafness, oculodentodigital dysplasia, peripheral neuropathy Charcot-Marie-Tooth disease and skin disorders erythorokeratoderma variabilis and Vohwinkel sydrome^1–4^. In terms of protein amino acid sequence features, the eutherian connexins were classified as 4TM α-helical transmembrane proteins including 4 transmembrane helices^5–9^. Morphologically, the gap junctions were described as “plaques” or “maculae” at intercellular interfaces including numerous intercellular channels that incorporated connexins^10,11^. Structurally, the eutherian connexins included 4 transmembrane α-helices traversing plasma membrane, cytoplasmic connexin regions including N-terminus, cytoplasmic loop that was positioned between second and third transmembrane helices and C-terminal domain, and, finally, extracellular connexin regions including two loops that were positioned between first and second transmembrane helices (region E1) and third and fourth transmembrane helices (region E2)^10–18^ (see Protein molecular evolution analysis below). The connexin hexamers (connexons or hemichannels) that were located in adjacent cells were implicated in formation of gap junction channel connexon pores and intercellular docking^10–18^. The homomeric connexons included single connexins, and heteromeric connexons included multiple connexins that were encoded by about 20 connexin genes among eutherians. For example, the analyses of connexin genes in human genome included either 20 connexin genes^5,6,9,16,19–24^ or 21 connexin genes^2,4,8,25–27^. The intercellular channels included either two identical connexons (homotypic junctions) or two different connexons (heterotypic junctions), and such combinatorial code contributed to functions of multiple cell types, tissues and organs expressing connexins^19^. The conventional human connexin gene nomenclatures included phylogenetic classifications of connexin genes into several classes and subclasses, including α-connexins or group II connexins, β-connexins or group I connexins, γ-connexins or group IIIb connexins and δ-connexins or group IIIa connexins and their naming using prefix *GJ* (gap junction), but conventional human connexin protein nomenclatures included connexin protein classifications according to predicted protein molecular mass calculated in kilodaltons and their naming using prefix CX^2,4–6,8,9,16,19–27^. For example, the human connexin CX31.1 was encoded by *GJB5* gene. These conventional connexin gene and protein classifications could be regarded as unsuitable in descriptions of comprehensive human connexin gene data sets, due to numerous ambiguities and inconsistencies in connexin gene and protein nomenclatures^6,22,23,25^.

Importantly, one new era in biomedical research was ushered in by the public eutherian reference genomic sequence data sets^28–37^. For example, one major aim of initial sequencing and analysis of human genome was to revise and update human gene data sets and uncover potential new drugs and drug targets, as well as molecular markers in medical diagnostics^38^. Nevertheless, future updates and revisions of human gene data sets were expected, due to the incompleteness of human reference genomic sequence assemblies^38,39^ and potential genomic sequence errors^40^. Specifically, the potential genomic sequence errors included Sanger DNA sequencing method errors (artefactual nucleotide deletions, insertions and substitutions), as well as analytical errors (erroneous gene annotations, genomic sequence misassemblies)^38–40^. For example, whereas the human initial integrated gene index included ≈32000 known and predicted protein coding genes^38^, recent analyses included ≈20000–21000 protein coding genes in human genome^39,41,42^. Furthermore, the eutherian reference genomic sequence assemblies including lower genomic sequence redundancies were more likely to include potential genomic sequence errors^38–46^ that could influence and bias phylogenetic analyses^47,48^. The eutherian comparative genomic analysis protocol RRID:SCR_014401 was established as one framework of eutherian gene descriptions^49–51^. The protocol included new test of reliability of public eutherian genomic sequences using genomic sequence redundancies, as well as new protein molecular evolution test using relative synonymous codon usage statistics that were applicable in revisions and updates of 11 eutherian gene data sets implicated in major physiological and pathological processes, including 1504 published complete coding sequences. For example, the protocol was applicable in initial descriptions of human genes^50,52^. There was positive correlation between genomic sequence redundancies of 35 public eutherian reference genomic sequence data sets respectively and published complete coding sequence numbers^50^. Therefore, the present analysis made attempts to revise and update comprehensive eutherian connexin gene data sets (*CXN* genes according to present study) and address and resolve major discrepancies in their descriptions, using eutherian comparative genomic analysis protocol and 35 public eutherian reference genomic sequence data sets (Supplementary Data File 1).

## Results and Discussion

### Gene annotations

**Figure 1:**
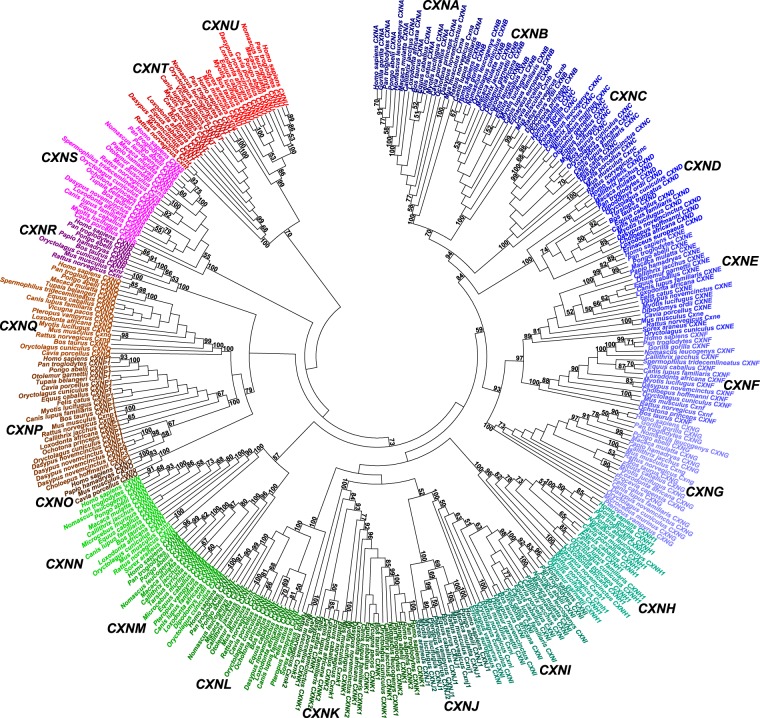
Phylogenetic analysis of eutherian connexin genes. The minimum evolution phylogenetic tree was calculated using maximum composite likelihood method. The bootstrap estimates higher than 50% were shown after 1000 replicates. The 21 major gene clusters *CXNA*-*CXNU* were indicated.

Among 631 *CXN* potential coding sequences, the tests of reliability of eutherian public genomic sequences annotated, in aggregate, 349 *CXN* complete coding sequences that were deposited in European Nucleotide Archive under accession numbers LT990249-LT990597 (https://www.ebi.ac.uk/ena/data/view/LT990249-LT990597) (Fig. 1) (Supplementary Data File 1). The most comprehensive curated eutherian *CXN* gene data set described 21 *CXN* major gene clusters *CXNA*-*CXNU*, 4 of which included evidence of differential gene expansions (*CXNH*, *CXNJ*, *CXNK* and *CXNP*) (Fig. 1) (Supplementary Data File 2). Specifically, the major gene cluster *CXNA* included 18 *GJB5* genes (Supplementary Data File 2A), major gene cluster *CXNB* included 18 *GJB4* genes (Supplementary Data File 2B), major gene cluster *CXNC* included 18 *GJB3* genes (Supplementary Data File 2C) and major gene cluster *CXND* included 15 *GJB7* genes (Supplementary Data File 2D). For example, the *CXND* gene was annotated in rodent Ord’s kangaroo rat genome although it was not annotated in mouse and brown rat genomic sequence assemblies^8,9^. Whereas the major gene cluster *CXNE* included 19 *GJB2* genes (Supplementary Data File 2E), major gene cluster *CXNF* included 17 *GJB6* genes (Supplementary Data File 2F) and major gene cluster *CXNG* included 21 *GJB1* genes (Supplementary Data File 2G). There were 18 *GJA4* genes annotated in major gene cluster *CXNH*, including *Otolemur garnettii CXNH1* paralogue (Supplementary Data File 2H). Whereas the major gene cluster *CXNI* included 20 *GJA5* genes (Supplementary Data File 2I), there were 12 *GJA3* genes annotated in major gene cluster *CXNJ*, including paralogues in little brown myotis and large flying fox genomes (Supplementary Data File 2J). Furthermore, there were 25 *GJA1* genes annotated in major gene cluster *CXNK* including evidence of differential gene expansions (Supplementary Data File 2K). For example, the present analysis initially described human *CXNK1* gene as complete coding sequence that disagreed with Fishman *et al*.^53^. Indeed, using eutherian *CXNK* orthologues and paralogues, the human *CXNK1* and *CXNK2* paralogues were annotated using indirect evidence of human gene annotations^38–41,46^. First, the pairwise nucleotide sequence identity between human paralogues *CXNK1* and *CXNK2* was *a* = 0,967 and pairwise nucleotide sequence identity between common chimpanzee paralogues *CXNK1* and *CXNK2* was *a* = 0,966. On the other hand, the pairwise nucleotide sequence identity between human *CXNK1* and common chimpanzee *CXNK1* was *a* = 0,988, and pairwise nucleotide sequence identity between human *CXNK2* and common chimpanzee *CXNK2* was *a* = 0,993. Furthermore, in agreement with Cruciani and Mikalsen^21,22^, the pairwise nucleotide sequence identity between mouse paralogues *Cxnk1* and *Cxnk2* was *a* = 0,52 and pairwise nucleotide sequence identity between brown rat paralogues *Cxnk1* and *Cxnk2* was *a* = 0,521 but pairwise nucleotide sequence identity between mouse *Cxnk1* and brown rat *Cxnk1* was *a* = 0,953 and pairwise nucleotide sequence identity between mouse *Cxnk2* and brown rat *Cxnk2* was *a* = 0,77. Third, the *CXNK1* and *CXNK2* paralogues were also annotated in horse, domestic dog, nine-banded armadillo and african bush elephant genomic sequences respectively. For example, the pairwise nucleotide sequence identity between horse paralogues *CXNK1* and *CXNK2* was *a* = 0,632 and pairwise nucleotide sequence identity between domestic dog paralogues *CXNK1* and *CXNK2* was *a* = 0,645 but pairwise nucleotide sequence identity between horse *CXNK1* and domestic dog *CXNK1* was *a* = 0,919 and pairwise nucleotide sequence identity between horse *CXNK2* and domestic dog *CXNK2* was *a* = 0,766. In addition, the pairwise nucleotide sequence identity between nine-banded armadillo paralogues *CXNK1* and *CXNK2* was *a* = 0,558 and pairwise nucleotide sequence identity between african bush elephant paralogues *CXNK1* and *CXNK2* was *a* = 0,696 but pairwise nucleotide sequence identity between nine-banded armadillo *CXNK2* and african bush elephant *CXNK1* was *a* = 0,911 and pairwise nucleotide sequence identity between nine-banded armadillo *CXNK1* and african bush elephant *CXNK2* was *a* = 0,679. Fourth, there were 4 eutherian *CXN* major gene clusters including evidence of differential gene expansions (*CXNH*, *CXNJ*, *CXNK* and *CXNP*), that was in agreement with analyses of differential gene expansions of vertebrate *CXN* genes of Hua *et al*.^5^ and Eastman *et al*.^23^. Fifth, Cruciani and Mikalsen^22^ indicated that positions of mutations in human *CXNK1* and *CXNK2* complete coding sequences were not randomly distributed, suggesting that human *CXNK1* and *CXNK2* complete coding sequences were *bona fide* paralogues.

Furthermore, the major gene cluster *CXNL* included 20 *GJA8* genes (Supplementary Data File 2L). The major gene cluster *CXNM* included 14 *GJA9* genes (Supplementary Data File 2M) and major gene cluster *CXNN* included 15 *GJA10* genes (Supplementary Data File 2N). For example, although it was not annotated in mouse and brown rat genomes^8,9^, the *CXNM* gene was annotated in rodent Ord’s kangaroo rat genomic sequence. There were 4 *GJC2* genes included in major gene cluster *CXNO* (Supplementary Data File 2O), but major gene cluster *CXNP* included 23 *GJC3* genes (Supplementary Data File 2P) and major gene cluster *CXNQ* included 17 *GJC1* genes (Supplementary Data File 2Q). For example, the evidence of differential gene expansions in major gene cluster *CXNP* included 4 *CXNP1*-*CXNP4* paralogues that were annotated in nine-banded armadillo genome. There were 8 *GJD3* genes annotated in major gene cluster *CXNR* (Supplementary Data File 2R). The major gene cluster *CXNS* included 20 *GJD2* genes (Supplementary Data File 2S). Finally, the major gene cluster *CXNT* included 14 *GJD5* genes (Supplementary Data File 2T) and major gene cluster *CXNU* included 13 *GJD4* genes (Supplementary Data File 2U). For example, the present eutherian *CXNT* gene annotations agreed with analyses of Goodenough and Paul^2^, Bosco *et al*.^4^, Beyer and Berthoud^8^, Söhl and Willecke^25,26^ and Iovine *et al*.^27^. However, they disagreed with analyses of Hua *et al*.^5^, Abascal and Zardoya^6^, Beyer and Berthoud^9^, Beyer *et al*.^16^, Willecke *et al*.^19^, Bruzzone^20^, Cruciani and Mikalsen^21,22^, Eastman *et al*.^23^ and Sonntag *et al*.^24^ that did not include major gene cluster *CXNT* (*GJD5* genes). Therefore, among 21 eutherian *CXN* major gene clusters *CXNA*-*CXNU*, the present *CXN* gene annotations initially described human *CXNK1* gene and annotated 22 human *CXN* genes. Yet, whereas the human *CXN* gene number estimates were likely complete, *CXN* gene number estimates in other 34 eutherian species were subject to future updates, due to incompleteness of eutherian reference genomic sequence assemblies and potential genomic sequence errors^38–48^ (Supplementary Data File 1).

### Phylogenetic analysis

The present phylogenetic analysis classified 21 eutherian *CXN* major gene clusters *CXNA*-*CXNU* using minimum evolution phylogenetic tree calculations (Fig. 1) and calculations of pairwise nucleotide sequence identity patterns (Supplementary Data File 3). The minimum evolution phylogenetic tree calculations were comparable with published phylogenetic analyses of human, eutherian and vertebrate *CXN* genes^4–6,20–23^. First, the clustering of β-connexins or group I connexins including major gene clusters *CXNA* (*GJB5*, CX31.1), *CXNB* (*GJB4*, CX30.3), *CXNC* (*GJB3*, CX31), *CXND* (*GJB7*, CX25), *CXNE* (*GJB2*, CX26), *CXNF* (*GJB6*, CX30) and *CXNG* (*GJB1*, CX32) agreed with phylogenetic analyses of Bosco *et al*.^4^, Hua *et al*.^5^, Abascal and Zardoya^6^, Bruzzone^20^, Cruciani and Mikalsen^21,22^ and Eastman *et al*.^23^. For example, whereas Hua *et al*.^5^ described connexin clusters I (*CXNE*-*CXNG*) and II (*CXNA*-*CXND*), Cruciani and Mikalsen^22^ described group I connexin clades IA (*CXNE*-*CXNG*) and IB (*CXNA*-*CXND*). Second, the distribution of α-connexins or group II connexins including major gene clusters *CXNH* (*GJA4*, CX37), *CXNI* (*GJA5*, CX40), *CXNJ* (*GJA3*, CX46), *CXNK* (*GJA1*, CX43) and *CXNL* (*GJA8*, CX50) was not supported by higher bootstrap estimates, except that clustering of major gene clusters *CXNI* and *CXNJ* agreed with Eastman *et al*.^23^. Of note, the clustering of major gene clusters *CXNM* (*GJA9*, CX59) and *CXNN* (*GJA10*, CX62) disagreed with phylogenetic analyses of Bosco *et al*.^4^, Hua *et al*.^5^, Abascal and Zardoya^6^, Bruzzone^20^, Cruciani and Mikalsen^21,22^ and Eastman *et al*.^23^. Third, although the grouping of γ-connexins or group IIIb connexins including major gene clusters *CXNO* (*GJC2*, CX47), *CXNP* (*GJC3*, CX30.2, CX31.3) and *CXNQ* (*GJC1*, CX45) agreed with Bosco *et al*.^4^, Hua *et al*.^5^, Abascal and Zardoya^6^, Bruzzone^20^, Cruciani and Mikalsen^21,22^ and Eastman *et al*.^23^, clustering of major gene clusters *CXNP* and *CXNQ* disagreed with these analyses. In addition, the grouping of major gene clusters *CXNO*, *CXNP* and *CXNQ* disagreed with human *CXN* nomenclature that was proposed by Söhl and Willecke^25^. Fourth, the distribution of δ-connexins or group IIIa connexins including major gene clusters *CXNR* (*GJD3*, CX31.9), *CXNS* (*GJD2*, CX36), *CXNT* (*GJD5*, *GJE1*, CX23) and *CXNU* (*GJD4*, CX40.1) was not monophyletic or supported by higher bootstrap estimates, except that clustering of major gene clusters *CXNT* and *CXNU* disagreed with phylogenetic analyses of Bosco *et al*.^4^, Hua *et al*.^5^, Abascal and Zardoya^6^, Bruzzone^20^, Cruciani and Mikalsen^21,22^ and Eastman *et al*.^23^.

Furthermore, the calculations of pairwise nucleotide sequence identity patterns among 21 eutherian *CXN* major gene clusters confirmed their phylogenetic classification (Supplementary Data File 3). First, the eutherian *CXN* gene data set including 349 complete coding sequences included average pairwise nucleotide sequence identity *ā* = 0,325 (largest pairwise nucleotide sequence identity *a*_max_ = 0,999, smallest pairwise nucleotide sequence identity *a*_min_ = 0,037, average absolute deviation *ā*_ad_ = 0,101). Second, among eutherian *CXN* major gene clusters including orthologues respectively, there were nucleotide sequence identity calculations typical in comparisons between eutherian orthologues (≈0,65–0,9)^49,50,52^. The exceptions were major gene clusters *CXNG* (*GJB1*, CX32) and *CXNQ* (*GJC1*, CX45) respectively including close orthologues (≈0,9–0,95), as well as major gene cluster *CXNU* (*GJD4*, CX40.1) including distant orthologues (≈0,45–0,65) agreeing with analyses of Abascal and Zardoya^6^ and Cruciani and Mikalsen^22^. Third, the present analysis discriminated between eutherian *CXN* major gene clusters including evidence of differential gene expansions (*CXNH*, *CXNJ*, *CXNK* and *CXNP*) and major gene clusters not including evidence of differential gene expansions. Specifically, the major gene clusters *CXNH* (*GJA4*, CX37) and *CXNK* (*GJA1*, CX43) respectively included close eutherian orthologues and paralogues (≈0,7–0,85)^49,50,52^, but major gene clusters *CXNJ* (GJA3, CX46) and *CXNP* (*GJC3*, CX30.2, CX31.3) respectively included typical eutherian orthologues and paralogues (≈0,45–0,7). Fourth, in comparisons between eutherian *CXN* major gene clusters, there were nucleotide sequence identity patterns of very close (>0,5), close (≈0,35–0,5), typical (≈0,25–0,35), distant (≈0,15–0,25) and very distant (<0,15) eutherian homologues^49,50,52^. For example, there were nucleotide sequence identity patterns of very close and close eutherian homologues in comparisons between major gene clusters *CXNA* (*GJB5*, CX31.1), *CXNB* (*GJB4*, CX30.3), *CXNC* (*GJB3*, CX31) and *CXND* (*GJB7*, CX25) respectively, and in comparisons between major gene clusters *CXNE* (*GJB2*, CX26), *CXNF* (*GJB6*, CX30) and *CXNG* (*GJB1*, CX32) respectively there were nucleotide sequence identity patterns of very close eutherian homologues^5,22^. There were nucleotide sequence identity patterns of close eutherian homologues in comparisons between major gene clusters *CXNI* (*GJA5*, CX40) and *CXNJ* (*GJA3*, CX46)^23^. In comparisons between major gene clusters *CXNM* (*GJA9*, CX59) and *CXNN* (*GJA10*, CX62) as well as in comparisons between major gene clusters *CXNO* (*GJC2*, CX47) and *CXNQ* (*GJC1*, CX45) there were nucleotide sequence identity patterns of close eutherian homologues agreeing with Bosco *et al*.^4^, Hua *et al*.^5^, Abascal and Zardoya^6^, Bruzzone^20^, Cruciani and Mikalsen^21,22^ and Eastman *et al*.^23^. Finally, in comparisons between major gene clusters *CXNR* (*GJD3*, CX31.9), *CXNS* (*GJD2*, CX36), *CXNT* (*GJD5*, *GJE1*, CX23) and *CXNU* (*GJD4*, CX40.1) respectively and other major gene clusters respectively, there were nucleotide sequence identity patterns of typical, distant and very distant eutherian homologues. Therefore, the present minimum evolution phylogenetic tree calculations (Fig. 1) and calculations of pairwise nucleotide sequence identity patterns (Supplementary Data File 3) proposed revised and updated phylogenetic classification of eutherian *CXN* genes.

### Protein molecular evolution analysis

**Figure 2:**
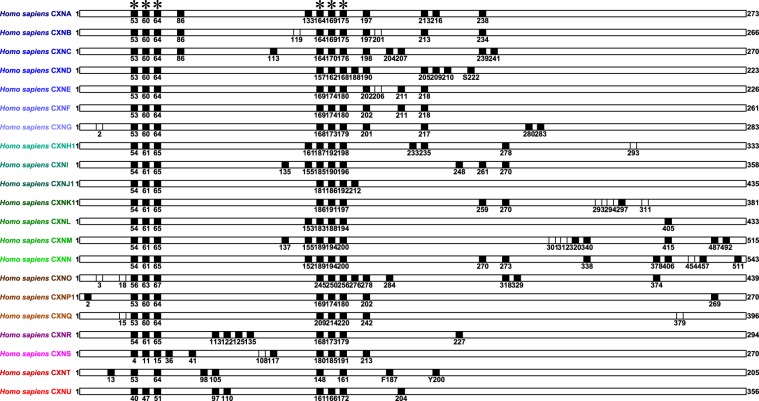
Major landmarks in eutherian connexin protein sequence alignments. The black squares labelled common cysteine amino acid residues and white squares labelled common N-glycosylation sites. The connexin amino acid signature common cysteine amino acid residues that were implicated in disulfide bonding were labelled by stars. The numbers indicated numbers of amino acid residues. The human substitutions of common cysteine amino acid residues were also indicated.

The eutherian CXN major protein cluster amino acid sequence alignments (Supplementary Data File 4) used CXN protein primary structure features as major alignment landmarks, including cysteine amino acid residues and predicted N-glycosylation sites common to 21 CXN major protein clusters respectively (Fig. 2). First, the eutherian CXN major protein clusters respectively included between 7–14 common cysteine amino acid residues. For example, whereas the CXNJ major protein cluster included 7 common cysteine amino acid residues, CXNN major protein cluster included 14 common cysteine amino acid residues. The CXN amino acid signature common cysteine amino acid residues that were implicated in disulfide bonding were described in protein amino acid sequence motifs C-x(6)-C-x(3)-C or C-x(10)-C and C-x(4,5)-C-x(5)-C or C-x(12,13)-C that agreed with phylogenetic analyses of Hua *et al*.^5^, Abascal and Zardoya^6^, Cruciani and Mikalsen^21,22^ and Eastman *et al*.^23^. Second, although they were described as not glycosylated proteins^4,10^, there were between 0–3 common predicted N-glycosylation sites annotated among eutherian CXN major protein clusters. For example, there were 3 common predicted N-glycosylation sites that were annotated in CXNK major protein cluster.

**Figure 3:**
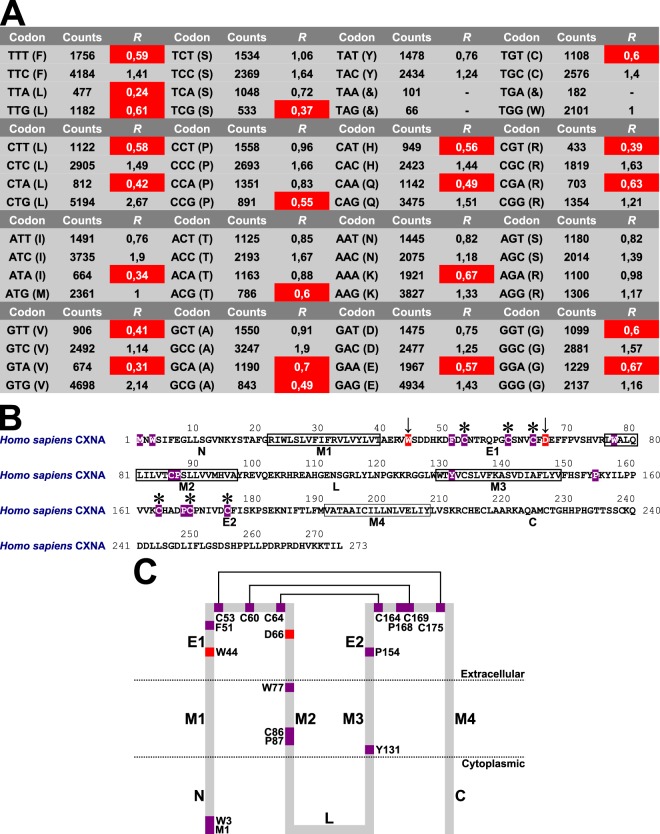
Tests of protein molecular evolution of eutherian connexins. (**A**) Relative synonymous codon usage statistics of eutherian *CXN* gene data set. The not preferable amino acid codons were indicated by white letters on red backgrounds. Counts, observed amino acid codon counts; *R*, relative synonymous codon usage statistics; &, stop codons. (**B**) Reference human CXNA protein amino acid sequence. Using white letters on violet backgrounds, the 15 invariant amino acid sites were shown. The 2 forward amino acid sites were indicated by arrows and shown using white letters on red backgrounds. The connexin amino acid signature common cysteine amino acid residues that were implicated in disulfide bonding were labelled by stars. The N-terminal and C-terminal boundaries of transmembrane α-helices 1–4 were described according to Nicholson^10^ and Sosinsky and Nicholson^11^. (**C**) Distribution of invariant and forward amino acid sites in human CXNA protein regions. The 15 invariant amino acid sites were shown using violet squares, and 2 forward amino acid sites were shown using red squares. The common cysteine amino acid residues that were implicated in disulfide bonding were connected by lines. C, C-terminal domain; E1 and E2, extracellular connexin regions 1 and 2; L, cytoplasmic loop; M1–M4, transmembrane α-helices 1–4; N, N-terminus.

Furthermore, using 349 *CXN* complete coding sequences (Supplementary Data File 4), the tests of protein molecular evolution first calculated relative synonymous codon usage statistics (*R*) of eutherian *CXN* gene data set, and described 22 amino acid codons with *R* ≤ 0.7 as not preferable amino acid codons (Fig. 3A). The tests of protein molecular evolution used human CXNA primary structure as reference protein amino acid sequence, using N-terminal and C-terminal boundaries of CXN transmembrane α-helices M1-M4, cytoplasmic CXN regions (N-terminus, cytoplasmic loop and C-terminal domain) and extracellular CXN regions E1 and E2 as reference points in analysis^10–18^ (Fig. 3B,C). For example, whereas the extracellular CXN regions E1 and E2 included average pairwise nucleotide sequence identity *ā* = 0,607 (*a*_max_ = 1, *a*_min_ = 0, *ā*_ad_ = 0,081) and CXN transmembrane α-helices M1-M4 included average pairwise nucleotide sequence identity *ā* = 0,504 (*a*_max_ = 1, *a*_min_ = 0,048, *ā*_ad_ = 0,104), cytoplasmic CXN regions included average pairwise nucleotide sequence identity *ā* = 0,177 (*a*_max_ = 1, *a*_min_ = 0,011, *ā*_ad_ = 0,1) agreeing with analyses of Hua *et al*.^5^, Abascal and Zardoya^6^, Cruciani and Mikalsen^21,22^ and Eastman *et al*.^23^. Thus, among 273 human CXNA protein amino acid residues, the tests of protein molecular evolution using relative synonymous codon usage statistics described 15 invariant amino acid sites (M1, W3, F51, C53, C60, C64, W77, C86, P87, Y131, P154, C164, P168, C169 and C175) and 2 variant alignment positions that did not include not preferable amino acid codons named forward amino acid sites (W44, D66) (Fig. 3B,C) (Supplementary Data File 4). For example, the human CXNA amino acid site W3 that was invariant in eutherian major protein clusters CXNA-CXNO, CXNQ and CXNR was described as critical in CXN protein secondary, tertiary and quaternary structural features and interactions with cytoplasmic proteins^16^. Furthermore, the human CXNA invariant amino acid sites C53, C60 and C64 in region E1 corresponded to common cysteine amino acid residues that were implicated in disulfide bonding and described in protein amino acid sequence motif C-x(6)-C-x(3)-C, and human CXNA invariant amino acid sites C164, C169 and C175 in region E2 corresponded to common cysteine amino acid residues that were implicated in disulfide bonding and described in protein amino acid sequence motif C-x(4,5)-C-x(5)-C^5,6,21–23^ (Fig. 2). Finally, the human CXNA forward amino acid sites W44 and D66 were described in extracellular region E1 that was implicated in gap junction channel connexon pore lining and ion selectivity modulation^11,13–15^. For example, the human CXNA forward amino acid site W44 was calculated among 329 *CXN* complete coding sequences, and human CXNA forward amino acid site D66 was calculated among 347 *CXN* complete coding sequences (Supplementary Data File 4). Therefore, in reference human CXNA primary structure, the present protein molecular evolution analysis described amino acid residues implicated as critical in eutherian CXN protein secondary, tertiary and quaternary structural features.

## Conclusions

The conventional connexin gene and protein classifications could be regarded as unsuitable in descriptions of comprehensive eutherian *CXN* gene data sets, due to ambiguities and inconsistencies in *CXN* gene and protein nomenclatures^6,22,23,25^. Using eutherian comparative genomic analysis protocol and 35 public eutherian reference genomic sequence assemblies^49,50,52^, the present analysis attempted to update and revise comprehensive eutherian *CXN* gene data sets, and address and resolve major discrepancies in their descriptions. The advantages of eutherian reference genomic sequence data sets included well established phylogenetic framework^28,31,33^, as well as calibrated taxon sampling including genomic sequence redundancies that were applicable in tests of reliability of eutherian public genomic sequences^29,30,32,38–41,43,44,46^. Indeed, the tests of reliability of eutherian public genomic sequences annotated most comprehensive curated eutherian *CXN* gene data set including, in aggregate, 349 *CXN* complete coding sequences. There were 21 *CXN* major gene clusters *CXNA*-*CXNU* described, 4 of which included evidence of differential gene expansions (*CXNH*, *CXNJ*, *CXNK* and *CXNP*). In addition, the present *CXN* gene annotations initially described human *CXNK1* gene and annotated 22 human *CXN* genes. The phylogenetic tree calculations and calculations of pairwise nucleotide sequence identity patterns proposed revised and updated phylogenetic classification of eutherian *CXN* genes. Finally, in reference human CXNA primary structure, the tests of protein molecular evolution using relative synonymous codon usage statistics described 15 invariant amino acid sites and 2 forward amino acid sites, including amino acid residues that were described as critical in CXN protein secondary, tertiary and quaternary structural features. In conclusion, the present comparative genomic analysis integrating gene annotations, phylogenetic analysis and protein molecular evolution analysis proposed new nomenclature of eutherian *CXN* genes and proteins.

## Methods

### Eutherian comparative genomic analysis protocol

The eutherian comparative genomic analysis protocol RRID:SCR_014401 integrated gene annotations, phylogenetic analysis and protein molecular evolution analysis with new genomics and protein molecular evolution tests into one framework of eutherian gene descriptions^49,50,52^.

### Gene annotations

In eutherian *CXN* gene annotations, the protocol included gene identifications in 35 public genomic sequences (Supplementary Data File 1), tests of reliability of eutherian public genomic sequences and multiple pairwise genomic sequence alignments. First, the protocol used sequence alignment editor BioEdit 7.0.5.3 in analyses and manipulations of nucleotide and protein sequences (http://www.mbio.ncsu.edu/BioEdit/bioedit.html). In identifications of potential *CXN* coding sequences in 35 eutherian reference genomic sequence data sets, the protocol used National Center for Biotechnology Information’s (NCBI) BLAST Genomes^35,36,54,55^ (https://blast.ncbi.nlm.nih.gov/Blast.cgi) or Ensembl genome browser’s BLAST or BLAT^37^ (https://www.ensembl.org). Second, the potential *CXN* coding sequences were then used in tests of reliability of eutherian public genomic sequences. The first test steps analysed nucleotide sequence coverages of each potential *CXN* coding sequence, using BLASTN^54,55^ and processed public Sanger DNA sequencing reads or traces deposited in NCBI’s Trace Archive^35^ (https://www.ncbi.nlm.nih.gov/Traces/trace.cgi). The protocol described potential *CXN* coding sequences as complete *CXN* coding sequences only if consensus trace sequence coverages were available for every nucleotide. On the other hand, if consensus trace sequence coverages were not available for every nucleotide, the potential *CXN* coding sequences were described as putative *CXN* coding sequences that were not used in analyses. The protocol then deposited complete *CXN* coding sequences in European Nucleotide Archive as one curated eutherian gene data set^56–58^ (https://www.ebi.ac.uk/ena/about/tpa-policy). In updated human and eutherian *CXN* gene classification and nomenclature, the protocol used guidelines of human gene nomenclature^59^ (http://www.genenames.org/about/guidelines) and guidelines of mouse gene nomenclature (http://www.informatics.jax.org/mgihome/nomen/gene.shtml). Specifically, the present eutherian *CXN* gene name assignments used both phylogenetic analysis (Fig. 1) and genomic sequence information (Supplementary Data File 1). Third, the protocol used mVISTA’s program AVID in multiple pairwise genomic sequence alignments using default settings^51,60^ (http://genome.lbl.gov/vista/index.shtml). In pairwise alignments with base sequences (*Homo sapiens*), the cut-offs of detection of common genomic sequence regions were calculated *a posteriori* using analyses of 11 eutherian major gene data sets^49,50,52^ including 95% along 100 bp (*Homo sapiens*, *Pan troglodytes*, *Gorilla gorilla*), 90% along 100 bp (*Pongo abelii*, *Nomascus leucogenys*), 85% along 100 bp (*Macaca mulatta*, *Papio hamadryas*), 80% along 100 bp (*Callithrix jacchus*), 75% along 100 bp (*Tarsius syrichta*, *Microcebus murinus*, *Otolemur garnettii*), 65% along 100 bp (Rodentia) or 70% along 100 bp in other pairwise alignments. However, the exceptions were pairwise alignments between base sequences and *Otolemur garnettii CXNH1*, *Myotis lucifugus CXNJ1* and *CXNJ2*, *Pteropus vampyrus CXNJ1* and *CXNJ2*, *Sorex araneus CXNJ1*, *Mus musculus Cxnk2*, *Rattus norvegicus Cxnk2*, *Equus caballus CXNK2*, *Canis lupus familiaris CXNK2*, *Felis catus CXNK1*, *Dasypus novemcinctus CXNK1*, *Loxodonta africana CXNK2*, *Oryctolagus cuniculus CXNP1*, *Dasypus novemcinctus CXNP1*-*CXNP4* and *Choloepus hoffmanni CXNP1* respectively including 60% along 100 bp as empirically calculated cut-off of detection of common genomic sequence regions. In preparatory steps of multiple pairwise genomic sequence alignments, the protocol used RepeatMasker version open-4.0.6 in detection and masking of transposable elements in base sequences using default settings, except that simple repeats and low complexity elements were not masked (sensitive mode, cross_match version 1.080812, RepBase Update 20160829, RM database version 20160829) (http://www.repeatmasker.org/).

### Phylogenetic analysis

In eutherian *CXN* gene data set phylogenetic analysis, the protocol included protein and nucleotide sequence alignments, calculations of phylogenetic trees, calculations of pairwise nucleotide sequence identities and analysis of differential gene expansions. First, the protocol translated complete *CXN* coding sequences using BioEdit 7.0.5.3, and aligned them at amino acid level using ClustalW that was implemented in BioEdit 7.0.5.3. The CXN protein primary sequence alignments were then manually corrected, and *CXN* nucleotide sequence alignments were prepared accordingly using BioEdit 7.0.5.3. Second, the protocol used MEGA 6.06 program^61,62^ in phylogenetic tree calculations, using minimum evolution method that was suitable in phylogenetic analysis of very close, close, typical, distant and very distant eutherian homologues (default settings, except gaps/missing data treatment = pairwise deletion and maximum composite likelihood method) (http://www.megasoftware.net/). Third, the pairwise nucleotide sequence identities of complete *CXN* coding sequences were calculated using BioEdit 7.0.5.3, and then used in statistical analyses (Microsoft Office Excel). More specifically, using *CXN* nucleotide sequence alignments, the protocol calculated average pairwise nucleotide sequence identities (*ā*) and their average absolute deviations (*ā*_ad_), as well as largest (*a*_max_) and smallest (*a*_min_) pairwise nucleotide sequence identities.

### Protein molecular evolution analysis

In protein molecular evolution analysis, the protocol included analysis of CXN protein amino acid sequence features and tests of protein molecular evolution that integrated patterns of *CXN* nucleotide sequence similarities with CXN protein primary structures. First, among 21 eutherian CXN major protein clusters respectively, the common cysteine amino acid residues were annotated manually. Second, using protein amino acid sequence motif N-x-[ST], the common predicted N-glycosylation sites were also annotated manually among 21 eutherian CXN major protein clusters respectively. Third, in eutherian CXN protein primary structures, the N-terminal and C-terminal boundaries of transmembrane α-helices 1–4 were described according to Nicholson^10^ and Sosinsky and Nicholson^11^. In tests of protein molecular evolution, the protocol used entire *CXN* nucleotide sequence alignments including 349 *CXN* nucleotide sequences and 114138 codons. For example, the average number of codons among *CXN* nucleotide sequence was 327 codons. The MEGA 6.06 program^61,62^ calculated relative synonymous codon usage statistics as ratios between observed and expected amino acid codon counts (*R* = Counts / Expected counts). The protocol described 22 amino acid codons having *R* ≤ 0.7 as not preferable amino acid codons, viz: TTT, TTA, TTG, CTT, CTA, ATA, GTT, GTA, TCG, CCG, ACG, GCA, GCG, CAT, CAA, AAA, GAA, TGT, CGT, CGA, GGT and GGA (Fig. 3A). Accordingly, the protocol described reference human CXNA protein sequence amino acid sites as invariant amino acid sites (invariant alignment positions), forward amino acid sites (variant alignment positions that did not include amino acid codons with *R* ≤ 0.7) or compensatory amino acid sites (variant alignment positions that included amino acid codons with *R* ≤ 0.7).

## Supplementary information

Supplementary Information

## Data Availability

The original curated eutherian connexin gene data set included 349 complete coding sequences that were deposited in European Nucleotide Archive (accession numbers: LT990249-LT990597).
