# Author Correction: Comparative genomic analysis of eutherian connexin genes

**DOI:** 10.1038/s41598-020-64608-x

**Published:** 2020-06-26

**Authors:** Marko Premzl

**Affiliations:** The Australian National University Alumni, Zagreb, Croatia

Correction to: *Scientific Reports* 10.1038/s41598-019-53458-x, published online 15 November 2019

This Article contains errors.

In the Introduction,

“For example, the protocol was applicable in initial descriptions of human genes^50,52^. There was positive correlation between genomic sequence redundancies of 35 public eutherian reference genomic sequence data sets respectively and published complete coding sequence numbers^50^.”

should read:

“For example, the protocol was applicable in initial descriptions of human genes^50,51^. There was positive correlation between genomic sequence redundancies of 35 public eutherian reference genomic sequence data sets respectively and published complete coding sequence numbers^51^.”

In the Results,

“For example, the present analysis initially described human *CXNK1* gene as complete coding sequence that disagreed with Fishman *et al*.^53^.”

should read:

“For example, the present analysis initially described human *CXNK1* gene as complete coding sequence that disagreed with Fishman *et al*.^52^.”

“Second, among eutherian *CXN* major gene clusters including orthologues respectively, there were nucleotide sequence identity calculations typical in comparisons between eutherian orthologues (≈0,65–0,9)^49,50,52^.”

should read:

“Second, among eutherian *CXN* major gene clusters including orthologues respectively, there were nucleotide sequence identity calculations typical in comparisons between eutherian orthologues (≈0,65–0,9)^49,50,51^.”

“Specifically, the major gene clusters *CXNH* (*GJA4*, CX37) and *CXNK* (*GJA1*, CX43) respectively included close eutherian orthologues and paralogues (≈0,7–0,85)^49,50,52^, but major gene clusters *CXNJ* (*GJA3*, CX46) and *CXNP* (*GJC3*, CX30.2, CX31.3) respectively included typical eutherian orthologues and paralogues (≈0,45–0,7). Fourth, in comparisons between eutherian *CXN* major gene clusters, there were nucleotide sequence identity patterns of very close (>0,5), close (≈0,35–0,5), typical (≈0,25–0,35), distant (≈0,15–0,25) and very distant (<0,15) eutherian homologues^49,50,52^.”

should read:

“Specifically, the major gene clusters *CXNH* (*GJA4*, CX37) and *CXNK* (*GJA1*, CX43) respectively included close eutherian orthologues and paralogues (≈0,7–0,85)^49,50,51^, but major gene clusters *CXNJ* (*GJA3*, CX46) and *CXNP* (*GJC3*, CX30.2, CX31.3) respectively included typical eutherian orthologues and paralogues (≈0,45–0,7). Fourth, in comparisons between eutherian *CXN* major gene clusters, there were nucleotide sequence identity patterns of very close (>0,5), close (≈0,35–0,5), typical (≈0,25–0,35), distant (≈0,15–0,25) and very distant (<0,15) eutherian homologues^49,50,51^.”

In the Conclusions,

“Using eutherian comparative genomic analysis protocol and 35 public eutherian reference genomic sequence assemblies^49,50,52^, the present analysis attempted to update and revise comprehensive eutherian *CXN* gene data sets, and address and resolve major discrepancies in their descriptions.”

should read:

“Using eutherian comparative genomic analysis protocol and 35 public eutherian reference genomic sequence assemblies^49,50,51^, the present analysis attempted to update and revise comprehensive eutherian *CXN* gene data sets, and address and resolve major discrepancies in their descriptions.”

In the Methods,

“The eutherian comparative genomic analysis protocol RRID:SCR_014401 integrated gene annotations, phylogenetic analysis and protein molecular evolution analysis with new genomics and protein molecular evolution tests into one framework of eutherian gene descriptions^49,50,52^.”

should read:

“The eutherian comparative genomic analysis protocol RRID:SCR_014401 integrated gene annotations, phylogenetic analysis and protein molecular evolution analysis with new genomics and protein molecular evolution tests into one framework of eutherian gene descriptions^49,50,51^.”

“In identifications of potential *CXN* coding sequences in 35 eutherian reference genomic sequence data sets, the protocol used National Center for Biotechnology Information’s (NCBI) BLAST Genomes^35,36,54,55^ (https://blast.ncbi.nlm.nih.gov/Blast.cgi) or Ensembl genome browser’s BLAST or BLAT^37^ (https://www.ensembl.org). Second, the potential *CXN* coding sequences were then used in tests of reliability of eutherian public genomic sequences. The first test steps analysed nucleotide sequence coverages of each potential *CXN* coding sequence, using BLASTN^54,55^ and processed public Sanger DNA sequencing reads or traces deposited in NCBI’s Trace Archive^35^ (https://www.ncbi.nlm.nih.gov/Traces/trace.cgi).”

should read:

“In identifications of potential *CXN* coding sequences in 35 eutherian reference genomic sequence data sets, the protocol used National Center for Biotechnology Information’s (NCBI) BLAST Genomes^35,36,53,54^ (https://blast.ncbi.nlm.nih.gov/Blast.cgi) or Ensembl genome browser’s BLAST or BLAT^37^ (https://www.ensembl.org). Second, the potential *CXN* coding sequences were then used in tests of reliability of eutherian public genomic sequences. The first test steps analysed nucleotide sequence coverages of each potential *CXN* coding sequence, using BLASTN^53,54^ and processed public Sanger DNA sequencing reads or traces deposited in NCBI’s Trace Archive^35^ (https://www.ncbi.nlm.nih.gov/Traces/trace.cgi).”

“The protocol then deposited complete *CXN* coding sequences in European Nucleotide Archive as one curated eutherian gene data set^56,57,58^ (https://www.ebi.ac.uk/ena/about/tpa-policy). In updated human and eutherian *CXN* gene classification and nomenclature, the protocol used guidelines of human gene nomenclature^59^ (http://www.genenames.org/about/guidelines) and guidelines of mouse gene nomenclature (http://www.informatics.jax.org/mgihome/nomen/gene.shtml). Specifically, the present eutherian *CXN* gene name assignments used both phylogenetic analysis (Fig. 1) and genomic sequence information (Supplementary Data File 1). Third, the protocol used mVISTA’s program AVID in multiple pairwise genomic sequence alignments using default settings^51,60^ (http://genome.lbl.gov/vista/index.shtml). In pairwise alignments with base sequences (*Homo sapiens*), the cut-offs of detection of common genomic sequence regions were calculated *a posteriori* using analyses of 11 eutherian major gene data sets^49,50,52^ including 95% along 100 bp (*Homo sapiens*, *Pan troglodytes*, *Gorilla gorilla*), 90% along 100 bp (*Pongo abelii*, *Nomascus leucogenys*), 85% along 100 bp (*Macaca mulatta*, *Papio hamadryas*), 80% along 100 bp (*Callithrix jacchus*), 75% along 100 bp (*Tarsius syrichta*, *Microcebus murinus*, *Otolemur garnettii*), 65% along 100 bp (Rodentia) or 70% along 100 bp in other pairwise alignments.”

should read:

“The protocol then deposited complete *CXN* coding sequences in European Nucleotide Archive as one curated eutherian gene data set^55,56,57^ (https://www.ebi.ac.uk/ena/about/tpa-policy). In updated human and eutherian *CXN* gene classification and nomenclature, the protocol used guidelines of human gene nomenclature^58^ (http://www.genenames.org/about/guidelines) and guidelines of mouse gene nomenclature (http://www.informatics.jax.org/mgihome/nomen/gene.shtml). Specifically, the present eutherian *CXN* gene name assignments used both phylogenetic analysis (Fig. 1) and genomic sequence information (Supplementary Data File 1). Third, the protocol used mVISTA’s program AVID in multiple pairwise genomic sequence alignments using default settings^59,60^ (http://genome.lbl.gov/vista/index.shtml). In pairwise alignments with base sequences (*Homo sapiens*), the cut-offs of detection of common genomic sequence regions were calculated *a posteriori*using analyses of 11 eutherian major gene data sets^49,50,51^ including 95% along 100 bp (*Homo sapiens, Pan troglodytes, Gorilla gorilla*), 90% along 100 bp (*Pongo abelii, Nomascus leucogenys*), 85% along 100 bp (*Macaca mulatta, Papio hamadryas*), 80% along 100 bp (*Callithrix jacchus*), 75% along 100 bp (*Tarsius syrichta, Microcebus murinus, Otolemur garnettii*), 65% along 100 bp (Rodentia) or 70% along 100 bp in other pairwise alignments.”

