# Supplementary material for: Comparative genomic analysis of eutherian connexin genes
Source: Sci Rep. 2019 Nov 15;9:16938. doi: 10.1038/s41598-019-53458-x (PMC6858305; doi:10.1038/s41598-019-53458-x)
Supplement: Supplementary file 1 — Supplementary Information [file 41598_2019_53458_MOESM1_ESM.pdf]

Title: Comparative genomic analysis of eutherian connexin genes

Author: Marko Premzl[1,*]

[1] Marko Premzl PhD, The Australian National University Alumni, 4 Kninski trg Sq., Zagreb, Croatia

[*] **E-mail contact address:** Marko.Premzl@alumni.anu.edu.au;

NCBI SciENcv: https://www.ncbi.nlm.nih.gov/myncbi/mpremzl/cv/130205/;

ORCID: https://orcid.org/0000-0002-3362-689X

Supplementary Information

Supplementary data file 1: Curated gene data set of eutherian connexin genes.

Supplementary data file 2: Multiple pairwise genomic sequence alignments of eutherian connexin genes.

Supplementary data file 3: Pairwise nucleotide sequence identity patterns of eutherian connexin genes.

Supplementary data file 4: Protein amino acid sequence alignments of eutherian connexins.

Supplementary data file 1: Curated gene data set of eutherian connexin genes.

Superordinal clade	Order	Species [a]	Genome assembly	Gene number	Gene [b]	Genomic coordinates [c]	GenBank ID
Euarchontoglires	Primates	Human (*Homo sapiens*)	GCF_000001405.33	22	*CXNA* (*GJB5*, CX31.1)	exon 1: ch.1: 34757331-34758152 bp	LT990249
					CXNB (*GJB4*, CX30.3)	exon 1: ch.1: 34761255-34762055 bp	LT990250
					CXNC (*GJB3*, CX31)	exon 1: ch.1: 34784763-34785575 bp	LT990251
					CXND (*GJB7*, CX25)	exon 1: ch.6: 87284241-87284912 bp	LT990252
					CXNE (*GJB2*, CX26)	exon 1: ch.13: 20188901-20189581 bp	LT990253
					CXNF (*GJB6*, CX30)	exon 1: ch.13: 20222695-20223480 bp	LT990254
					CXNG (*GJB1*, CX32)	exon 1: ch.X: 71223708-71224559 bp	LT990255
					CXNH1 (*GJA4*, CX37)	exon 1: ch.1: 34794214-34795215 bp	LT990256
					CXNI (*GJA5*, CX40)	exon 1: ch.1: 147758162-147759238 bp	LT990257
					CXNJ1 (*GJA3*, CX46)	exon 1: ch.13: 20141981-20143288 bp	LT990258
					CXNK1 (*GJA1P*, CX43p1)	exon 1: ch.5: 109051312-109052457 bp	LT990259
					CXNK2 (*GJA1*, CX43)	exon 1: ch.6: 121446848-121447996 bp	LT990260
					CXNL (*GJA8*, CX50)	exon 1: ch.1: 147907956-147909257 bp	LT990261
					CXNM (*GJA9*, CX59)	exon 1: ch.1: 38874551-38876098 bp	LT990262
					CXNN (*GJA10*,	exon 1: ch.6: 89894469-89896100 bp	LT990263

					CX62)		
					CXNO (*GJC2*, CX47)	exon 1: ch.1: 228157759-228159078 bp	LT990264
					CXNP1 (*GJC3*, CX30.2, CX31.3)	exon 1: ch.7: 99928808-99929620 bp	LT990265
					CXNQ (*GJC1*, CX45)	exon 1: ch.17: 44804627-44805817 bp	LT990266
					CXNR (*GJD3*, CX31.9)	exon 1: ch.17: 40362931-40363815 bp	LT990267
					CXNS (*GJD2*, CX36)	exon 1: ch.15: 34752478-34753290 bp	LT990268
					CXNT (*GJD5*, *GJE1*, CX23)	exon 1: ch.6: 142133159-142133197 bp<br>exon 2: ch.6: 142133850-142134044 bp<br>exon 3: ch.6: 142134539-142134922 bp	LT990269
					CXNU (*GJD4*, CX40.1)	exon 1: ch.10: 35607556-35608626 bp	LT990270
		Common chimpanzee (*Pan troglodytes*)	GCF_000001515.7	18	*CXNA*	exon 1: ch.1: 34074526-34075347 bp	LT990271
					CXND	exon 1: ch.6: 90385805-90386476 bp	LT990272
					CXNE	exon 1: ch.13: 4746907-4747587 bp	LT990273
					CXNF	exon 1: ch.13: 4780562-4781347 bp	LT990274
					CXNG	exon 1: ch.X: 70652809-70653660 bp	LT990275
					CXNH1	exon 1: ch.1: 34113610-34114611 bp	LT990276
					CXNI	exon 1: ch.1: 110766033-110767109 bp	LT990277
					CXNK1	exon 1: ch.5: 108517014-108518159 bp	LT990278
					CXNK2	exon 1: ch.6: 125168466-125169614 bp	LT990279
					CXNL	exon 1: ch.1: 110614087-110615388 bp	LT990280
					CXNM	exon 1: ch.1: 38272192-38273739 bp	LT990281
					CXNN	exon 1: ch.6: 93100239-93101870 bp	LT990282
					CXNP1	exon 1: ch.7: 102984319-102985131 bp	LT990283

					CXNQ	exon 1: ch.17: 12756145-12757335 bp	LT990284
					CXNR	exon 1: ch.17: 17243984-17244865 bp	LT990285
					CXNS	exon 1: ch.15: 15024393-15025205 bp	LT990286
					CXNT	exon 1: ch.6: 146120850-146120888 bp<br>exon 2: ch.6: 146121540-146121734 bp<br>exon 3: ch.6: 146122231-146122614 bp	LT990287
					CXNU	exon 1: ch.10: 36679716-36680786 bp	LT990288
					putative	exon 1: ch.1: 34078454-34079254 bp	-
					putative	exon 1: ch.1: 34104160-34104972 bp	-
					putative	exon 1: ch.1: 207198918-207200237 bp	-
					putative	exon 1: ch.13: 4699951-4701258 bp	-
		Western gorilla (*Gorilla gorilla*)	GCF_000151905.2	6	*CXNA*	exon 1: ch.1: 35051948-35052769 bp	LT990289
					CXNB	exon 1: ch.1: 35055875-35056675 bp	LT990290
					CXNC	exon 1: ch.1: 35079173-35079985 bp	LT990291
					CXNF	exon 1: ch.13: 1771182-1771967 bp	LT990292
					CXNG	exon 1: ch.X: 70994028-70994879 bp	LT990293
					CXNH1	exon 1: ch.1: 35088608-35089609 bp	LT990294
					putative	exon 1: ch.1: 39222965-39224512 bp	-
					putative	exon 1: ch.1: 127005347-127006423 bp	-
					putative	exon 1: ch.1: 127157503-127158804 bp	-
					putative	exon 1: ch.5: 38629480-38630670 bp	-
					putative	exon 1: ch.5: 43114978-43115859 bp	-
					putative	exon 1: ch.5: 92111738-92112883 bp	-
					putative	exon 1: ch.6: 92190300-92191934 bp	-
					putative	exon 1: ch.6: 123730369-123731517 bp	-
					putative	exon 1: ch.6: 144779042-144779080 bp<br>exon 2: ch.6: 144779732-144779926 bp<br>exon 3: ch.6: 144780421-144780804 bp	-
					putative	exon 1: ch.7: 78152393-78153205 bp	-

					putative	exon 1: ch.10: 85103935-85105005 bp	-
					putative	exon 1: ch.13: 1690101-1691408 bp	-
					putative	exon 1: ch.13: 1737331-1738011 bp	-
					putative	exon 1: ch.15: 12581320-12582132 bp	-
		Sumatran orangutan (*Pongo abelii*)	GCF_000001545.4	16	*CXNA*	exon 1: ch.1: 195482130-195482951 bp	LT990295
					CXNC	exon 1: ch.1: 195454390-195455202 bp	LT990296
					CXNE	exon 1: ch.13: 19280971-19281651 bp	LT990297
					CXNG	exon 1: ch.X: 68720051-68720902 bp	LT990298
					CXNH1	exon 1: ch.1: 195444720-195445721 bp	LT990299
					CXNI	exon 1: ch.1: 107057302-107058378 bp	LT990300
					CXNJ1	exon 1: ch.13: 19231933-19233240 bp	LT990301
					CXNK1	exon 1: ch.6: 123920040-123921188 bp	LT990302
					CXNL	exon 1: ch.1: 107210614-107211915 bp	LT990303
					CXNM	exon 1: ch.1: 191264020-191265564 bp	LT990304
					CXNN	exon 1: ch.6: 90874177-90875808 bp	LT990305
					CXNP1	exon 1: ch.7: 8899087-8899899 bp	LT990306
					CXNQ	exon 1: ch.17: 44635209-44636399 bp	LT990307
					CXNR	exon 1: ch.17: 49058366-49059250 bp	LT990308
					CXNS	exon 1: ch.15: 30925716-30926528 bp	LT990309
					CXNU	exon 1: ch.10: 36687541-36688566 bp	LT990310
					putative	exon 1: ch.1: 21440574-21441899 bp	-
					putative	exon 1: ch.13: 19314591-19315376 bp	-
		Northern white-cheeked gibbon (*Nomascus leucogenys*)	GCF_000146795.2	14	*CXNA*	exon 1: ch.12: 4079298-4080119 bp	LT990311
					CXNB	exon 1: ch.12: 4083193-4083993 bp	LT990312
					CXNC	exon 1: ch.12: 4106123-4106935 bp	LT990313
					CXNF	exon 1: ch.5: 64804711-64805496 bp	LT990314
					CXNG	exon 1: ch.X: 64363095-64363946 bp	LT990315

					CXNH1	exon 1: ch.12: 4115434-4116435 bp	LT990316
					CXNI	exon 1: ch.12: 53328691-53329767 bp	LT990317
					CXNK1	exon 1: ch.3: 108762348-108763496 bp	LT990318
					CXNL	exon 1: ch.12: 53182101-53183402 bp	LT990319
					CXNM	exon 1: ch.12: 8217117-8218664 bp	LT990320
					CXNN	exon 1: ch.3: 76659666-76661297 bp	LT990321
					CXNS	exon 1: ch.6: 66110954-66111766 bp	LT990322
					CXNT	exon 1: ch.3: 129508500-129508538 bp<br>exon 2: ch.3: 129509188-129509382 bp<br>exon 3: ch.3: 129509838-129510230 bp	LT990323
					CXNU	exon 1: ch.18: 47535457-47536518 bp	LT990324
					putative	exon 1: ch.17: 35050329-35051141 bp	-
					putative	exon 1: ch.19: 31972534-31973724 bp	-
					putative	exon 1: ch.19: 34198192-34199088 bp	-
		Rhesus monkey (*Macaca mulatta*)	GCF_000772875.2	14	*CXNA*	exon 1: ch.1: 33904028-33904849 bp	LT990325
					CXNB	exon 1: ch.1: 33907965-33908765 bp	LT990326
					CXND	exon 1: ch.4: 85432849-85433598 bp	LT990327
					CXNE	exon 1: ch.17: 786245-786925 bp	LT990328
					CXNG	exon 1: ch.X: 65956073-65956924 bp	LT990329
					CXNH1	exon 1: ch.1: 33941008-33942009 bp	LT990330
					CXNI	exon 1: ch.1: 122617480-122618556 bp	LT990331
					CXNJ1	exon 1: ch.17: 737979-739277 bp	LT990332
					CXNL	exon 1: ch.1: 122766159-122767466 bp	LT990333
					CXNM	exon 1: ch.1: 38063864-38065411 bp	LT990334
					CXNN	exon 1: ch.4: 88027722-88029353 bp	LT990335
					CXNQ	exon 1: ch.16: 50694168-50695358 bp	LT990336
					CXNS	exon 1: ch.7: 10745081-10745893 bp	LT990337
					CXNU	exon 1: ch.9: 35840327-35841397 bp	LT990338

					putative	exon 1: ch.1: 33931392-33932204 bp	-
					putative	exon 1: ch.1: 139158854-139160179 bp	-
					putative	exon 1: ch.3: 125437240-125438052 bp	-
					putative	exon 1: ch.4: 146334753-146335901 bp	-
					putative	exon 1: ch.16: 46190673-46191554 bp	-
					putative	exon 1: ch.17: 809894-810679 bp	-
		Hamadryas baboon (*Papio hamadryas*)	Pham_1.0 (Ensembl)	6	*CXNE*	exon 1: Contig467167_Contig450264: 9567-10247 bp	LT990339
					CXNG	exon 1: Contig670420_Contig596042: 42739-43590 bp	LT990340
					CXNI	exon 1: Contig690376_Contig243594: 38645-39721 bp	LT990341
					CXNO	exon 1: Contig187743_Contig442067: 23740-25065 bp	LT990342
					CXNR	exon 1: Contig717904_Contig713282: 46776-47663 bp	LT990343
					CXNS	exon 1: Contig770831_Contig74359: 88038-88850 bp	LT990344
					putative	exon 1: Contig341951_Contig437940: 12541-13842 bp	-
					putative	exon 1: Contig387865_Contig467746: 2944-3765 bp	-
					putative	exon 1: Contig462634_Contig355371: 9033-10103 bp	-
					putative	exon 1: Contig467167_Contig450264: 28505-29290 bp	-
					putative	exon 1: Contig503552_Contig289169: 28895-29896 bp	-
					putative	exon 1: Contig503552_Contig289169: 38510-39322 bp	-
					putative	exon 1: Contig704338_Contig333118: 30754-32061 bp	-
					putative	exon 1: Contig712940_Contig508627: 14389-15579 bp	-
					putative	exon 1: Contig724829_Contig397886: 103159-104790 bp	-
					putative	exon 1: Contig735228_Contig705091: 105280-106071 bp	-
					putative	exon 1: Contig735228_Contig705091: 170159-170971 bp	-
		Common marmoset (*Callithrix jacchus*)	GCF_000004665.1	13	*CXNA*	exon 1: ch.7: 69055001-69055822 bp	LT990345
					CXNB	exon 1: ch.7: 69059400-69060200 bp	LT990346
					CXNC	exon 1: ch.7: 69083813-69084625 bp	LT990347
					CXND	exon 1: ch.4: 86333864-86334616 bp	LT990348

					CXNE	exon 1: ch.5: 133523437-133524117 bp	LT990349
					CXNF	exon 1: ch.5: 133556169-133556954 bp	LT990350
					CXNG	exon 1: ch.X: 63110153-63111004 bp	LT990351
					CXNI	exon 1: ch.18: 1732609-1733685 bp	LT990352
					CXNK1	exon 1: ch.4: 121476944-121478092 bp	LT990353
					CXNL	exon 1: ch.18: 1594191-1595498 bp	LT990354
					CXNM	exon 1: ch.7: 73286394-73287938 bp	LT990355
					CXNN	exon 1: ch.4: 88994945-88996642 bp	LT990356
					CXNP1	exon 1: ch.2: 11994639-11995445 bp	LT990357
					putative	exon 1: ch.5: 133474902-133476206 bp	-
					putative	exon 1: ch.7: 69093093-69094094 bp	-
					putative	exon 1: ch.10: 41631234-41632046 bp	-
					putative	exon 1: ch.19: 33527576-33528895 bp	-
		Philippine tarsier (*Tarsius syrichta*)	GCF_000164805.1	1	*CXNM*	exon 1: NW_007251427.1: 795743-797272 bp	LT990358
					putative	exon 1: NW_007052101.1: 33240-34430 bp	-
					putative	exon 1: NW_007071257.1: 48623-49474 bp	-
					putative	exon 1: NW_007231388.1: 25154-26209 bp	-
					putative	exon 1: NW_007231456.1: 3676-4425 bp	-
					putative	exon 1: NW_007231875.1: 107180-108058 bp	-
					putative	exon 1: NW_007231875.1: 111152-111952 bp	-
					putative	exon 1: NW_007231875.1: 148063-149163 bp	-
					putative	exon 1: NW_007249296.1: 229282-229320 bp<br>exon 2: NW_007249296.1: 229966-230160 bp<br>exon 3: NW_007249296.1: 230712-231107 bp	-
					putative	exon 1: NW_007251932.1: 4022-4771 bp	-
					putative	exon 1: NW_007252914.1: 482127-482939 bp	-
					putative	exon 1: NW_007252931.1: 362499-363575 bp	-
					putative	exon 1: NW_007252931.1: 508650-509969 bp	-

					putative	exon 1: NW_007253900.1: 443418-444158 bp	-
					putative	exon 1: NW_007253946.1: 169825-170505 bp	-
					putative	exon 1: NW_007253946.1: 193060-193845 bp	-
					putative	exon 1: NW_007255969.1: 415308-417074 bp	-
					putative	exon 1: NW_007261241.1: 17133-17882 bp	-
		Gray mouse lemur (*Microcebus murinus*)	GCF_000165445.2	4	*CXNC*	exon 1: NC_033661.1: 32816537-32817439 bp	LT990359
					CXNG	exon 1: NC_033692.1: 3651558-3652409 bp	LT990360
					CXNM	exon 1: NC_033661.1: 36537667-36539211 bp	LT990361
					CXNN	exon 1: NC_033665.1: 22840046-22841641 bp	LT990362
					putative	exon 1: NC_033661.1: 32793945-32794724 bp	-
					putative	exon 1: NC_033661.1: 32797791-32798591 bp	-
					putative	exon 1: NC_033661.1: 32825238-32826242 bp	-
					putative	exon 1: NC_033661.1: 110052267-110053586 bp	-
					putative	exon 1: NC_033661.1: 110203754-110204827 bp	-
					putative	exon 1: NC_033662.1: 87183827-87184639 bp	-
					putative	exon 1: NC_033665.1: 20464626-20465432 bp	-
					putative	exon 1: NC_033665.1: 81086749-81087897 bp	-
					putative	exon 1: NC_033672.1: 83122435-83123220 bp	-
					putative	exon 1: NC_033672.1: 83141168-83141848 bp	-
					putative	exon 1: NC_033672.1: 83183211-83184536 bp	-
					putative	exon 1: NC_033672.1: 84491285-84492595 bp	-
					putative	exon 1: NC_033675.1: 29163065-29163931 bp	-
					putative	exon 1: NC_033675.1: 32958727-32959917 bp	-
					putative	exon 1: NC_033676.1: 29987942-29988757 bp	-
					putative	exon 1: NC_033684.1: 17496251-17497435 bp	-
		Northern greater galago (*Otolemur garnettii*)	GCF_000181295.1	8	*CXNB*	exon 1: NW_003852599.1: 2475511-2476314 bp	LT990363
					CXNC	exon 1: NW_003852599.1: 2498201-2499013 bp	LT990364

					CXNE	exon 1: NW_003852501.1: 1613971-1614651 bp	LT990365
					CXNH1	exon 1: NW_003852599.1: 2542586-2543521 bp	LT990366
					CXNI	exon 1: NW_003852468.1: 625910-626983 bp	LT990367
					CXNL	exon 1: NW_003852468.1: 484406-485860 bp	LT990368
					CXNP1	exon 1: NW_003852537.1: 1021814-1022623 bp	LT990369
					CXNS	exon 1: NW_003852445.1: 1970336-1971148 bp	LT990370
					putative	exon 1: NW_003852397.1: 16813935-16815083 bp	-
					putative	exon 1: NW_003852413.1: 19710832-19712022 bp	-
					putative	exon 1: NW_003852413.1: 23643805-23644677 bp	-
					putative	exon 1: NW_003852420.1: 21923842-21924843 bp	-
					putative	exon 1: NW_003852501.1: 1575385-1576170 bp	-
					putative	exon 1: NW_003852501.1: 1663268-1664662 bp	-
					putative	exon 1: NW_003852513.1: 2616742-2618217 bp	-
					putative	exon 1: NW_003852539.1: 4220585-4222129 bp	-
					putative	exon 1: NW_003852599.1: 2471959-2472780 bp	-
					putative	exon 1: NW_003852614.1: 622648-623499 bp	-
					putative	exon 1: NW_003852680.1: 598364-599665 bp	-
	Scadentia	Northern treeshrew (*Tupaia belangeri*)	tupBel1 (Ensembl)	3	*CXNP1*	exon 1: GeneScaffold_5273: 82918-83817 bp	LT990371
					CXNQ	exon 1: scaffold_72215: 90-1280 bp	LT990372
					CXNS	exon 1: scaffold_135811: 84295-85107 bp	LT990373
					putative	exon 1: scaffold_3495: 7907-9445 bp	-
					putative	exon 1: scaffold_119182: 29940-31565 bp	-
	Rodentia	Mouse (*Mus musculus*)	GCF_000001635.24	20	*Cxna*	exon 1: ch.4: 127355534-127356349 bp	LT990374
					Cxnb	exon 1: ch.4: 127351346-127352146 bp	LT990375
					Cxnc	exon 1: ch.4: 127325925-127326737 bp	LT990376
					Cxne	exon 1: ch.14: 57100069-57100788 bp	LT990377
					Cxnf	exon 1: ch.14: 57124017-57124802 bp	LT990378

					Cxng	exon 1: ch.X: 101384168-101385019 bp	LT990379
					Cxnh1	exon 1: ch.4: 127311967-127312968 bp	LT990380
					Cxni	exon 1: ch.3: 97050629-97051705 bp	LT990381
					Cxnj1	exon 1: ch.14: 57035660-57036913 bp	LT990382
					Cxnk1	exon 1: ch.10: 56387547-56388695 bp	LT990383
					Cxnk2	exon 1: ch.X: 160903083-160903934 bp	LT990384
					Cxnl	exon 1: ch.3: 96919022-96920344 bp	LT990385
					Cxnn	exon 1: ch.4: 32600865-32602382 bp	LT990386
					Cxno	exon 1: ch.11: 59176332-59177687 bp	LT990387
					Cxnp1	exon 1: ch.5: 137957212-137957988 bp	LT990388
					Cxnq	exon 1: ch.11: 102799985-102801175 bp	LT990389
					Cxnr	exon 1: ch.11: 98982180-98983016 bp	LT990390
					Cxns	exon 1: ch.2: 114011029-114011961 bp	LT990391
					Cxnt	exon 1: ch.10: 14718110-14718148 bp<br>exon 2: ch.10: 14717237-14717431 bp<br>exon 3: ch.10: 14716419-14716814 bp	LT990392
					Cxnu	exon 1: ch.18: 9279982-9281010 bp	LT990393
		Brown rat (*Rattus norvegicus*)	GCF_000001895.5	18	*Cxna*	exon 1: ch.5: 145422357-145423172 bp	LT990394
					Cxnb	exon 1: ch.5: 145417418-145418215 bp	LT990395
					Cxnc	exon 1: ch.5: 145391311-145392123 bp	LT990396
					Cxne	exon 1: ch.15: 37378776-37379513 bp	LT990397
					Cxnf	exon 1: ch.15: 37401556-37402341 bp	LT990398
					Cxng	exon 1: ch.X: 71278506-71279357 bp	LT990399
					Cxnh1	exon 1: ch.5: 145375199-145376200 bp	LT990400
					Cxni	exon 1: ch.2: 199181847-199183019 bp	LT990401
					Cxnj1	exon 1: ch.15: 37299846-37301096 bp	LT990402
					Cxnk1	exon 1: ch.20: 37886159-37887307 bp	LT990403
					Cxnk2	exon 1: ch.X: 35621805-35622665 bp	LT990404

					Cxnl	exon 1: ch.2: 199051071-199052393 bp	LT990405
					Cxnn	exon 1: ch.5: 47835066-47836586 bp	LT990406
					Cxnp1	exon 1: ch.12: 19166239-19167012 bp	LT990407
					Cxnq	exon 1: ch.10: 90781674-90782864 bp	LT990408
					Cxnr	exon 1: ch.10: 86889421-86890257 bp	LT990409
					Cxns	exon 1: ch.3: 105468291-105469103 bp	LT990410
					Cxnt	exon 1: ch.1: 8885929-8885967 bp<br>exon 2: ch.1: 8885046-8885240 bp<br>exon 3: ch.1: 8883961-8884356 bp	LT990411
					putative	exon 1: ch.10: 45527422-45528744 bp	-
					putative	exon 1: ch.17: 62297086-62298105 bp	-
		Ord's kangaroo rat (*Dipodomys ordii*)	GCF_000151885.1	4	*CXNB*	exon 1: NW_012267267.1: 72018-72818 bp	LT990412
					CXND	exon 1: NW_012267374.1: 2124603-2125409 bp	LT990413
					CXNE	exon 1: NW_012267217.1: 27667071-27667808 bp	LT990414
					CXNM	exon 1: NW_012267249.1: 1261279-1262517 bp	LT990415
					putative	exon 1: NW_012267217.1: 27638299-27639084 bp	-
					putative	exon 1: NW_012267217.1: 27721042-27722358 bp	-
					putative	exon 1: NW_012267227.1: 20621310-20622212 bp	-
					putative	exon 1: NW_012267248.1: 16264671-16266128 bp	-
					putative	exon 1: NW_012267267.1: 28465-29466 bp	-
					putative	exon 1: NW_012267267.1: 41156-41959 bp	-
					putative	exon 1: NW_012267267.1: 76561-77385 bp	-
					putative	exon 1: NW_012267284.1: 7065804-7066664 bp	-
					putative	exon 1: NW_012267358.1: 3772907-3773758 bp	-
					putative	exon 1: NW_012267363.1: 797014-798204 bp	-
					putative	exon 1: NW_012267420.1: 2416658-2417806 bp	-
		Domesticated guinea pig (*Cavia porcellus*)	GCF_000151735.1	14	*CXNA*	exon 1: NT_176394.1: 2698849-2699820 bp	LT990416
					CXNB	exon 1: NT_176394.1: 2695177-2695977 bp	LT990417

					CXNC	exon 1: NT_176394.1: 2663953-2664750 bp	LT990418
					CXNE	exon 1: NT_176413.1: 23592354-23593175 bp	LT990419
					CXNH1	exon 1: NT_176394.1: 2655880-2656881 bp	LT990420
					CXNI	exon 1: NT_176417.1: 45792815-45793891 bp	LT990421
					CXNK1	exon 1: NT_176418.1: 78654486-78655634 bp	LT990422
					CXNL	exon 1: NT_176417.1: 45964108-45965430 bp	LT990423
					CXNO	exon 1: NT_176413.1: 372181-373440 bp	LT990424
					CXNP1	exon 1: NT_176389.1: 4253589-4254359 bp	LT990425
					CXNQ	exon 1: NT_176352.1: 9111405-9112583 bp	LT990426
					CXNS	exon 1: NT_176409.1: 20642322-20643134 bp	LT990427
					CXNT	exon 1: NT_176351.1: 4978706-4978744 bp<br>exon 2: NT_176351.1: 4977864-4978046 bp<br>exon 3: NT_176351.1: 4976916-4977308 bp	LT990428
					CXNU	exon 1: NT_176380.1: 8560262-8561269 bp	LT990429
					putative	exon 1: NT_176413.1: 23620571-23621356 bp	-
		Thirteen-lined ground squirrel (*Spermophilus tridecemlineatus*)	GCF_000236235.1	3	*CXNF*	exon 1: NW_004936720.1: 1493733-1494518 bp	LT990430
					CXNQ	exon 1: NW_004936541.1: 1178913-1180145 bp	LT990431
					CXNS	exon 1: NW_004936673.1: 1131018-1131830 bp	LT990432
					putative	exon 1: NW_004936474.1: 17404952-17405809 bp	-
					putative	exon 1: NW_004936474.1: 17408623-17409423 bp	-
					putative	exon 1: NW_004936474.1: 17429926-17430732 bp	-
					putative	exon 1: NW_004936474.1: 17439327-17440331 bp	-
					putative	exon 1: NW_004936474.1: 20867307-20868848 bp	-
					putative	exon 1: NW_004936490.1: 15427263-15428141 bp	-
					putative	exon 1: NW_004936510.1: 2391570-2393138 bp	-
					putative	exon 1: NW_004936543.1: 73253-74110 bp	-
					putative	exon 1: NW_004936574.1: 3880025-3881305 bp	-
					putative	exon 1: NW_004936625.1: 3839999-3840037 bp	-

						exon 2: NW_004936625.1: 3839188-3839382 bp<br>exon 3: NW_004936625.1: 3838326-3838721 bp	
					putative	exon 1: NW_004936658.1: 3850481-3851737 bp	-
					putative	exon 1: NW_004936658.1: 3930067-3931215 bp	-
					putative	exon 1: NW_004936720.1: 1528201-1528929 bp	-
					putative	exon 1: NW_004936720.1: 1583588-1584904 bp	-
					putative	exon 1: NW_004936762.1: 390313-391164 bp	-
					putative	exon 1: NW_004936864.1: 69445-70752 bp	-
					putative	exon 1: NW_004936867.1: 374638-375933 bp	-
	Lagomorpha	European rabbit (*Oryctolagus cuniculus*)	GCF_000003625.3	18	*CXNB*	exon 1: NW_003159330.1: 3557075-3557590 bp	LT990433
					CXNC	exon 1: NW_003159330.1: 3530269-3531411 bp	LT990434
					CXND	exon 1: ch.12: 74119333-74120139 bp	LT990435
					CXNE	exon 1: ch.8: 44439132-44439869 bp	LT990436
					CXNF	exon 1: ch.8: 44395756-44396541 bp	LT990437
					CXNG	exon 1: ch.X: 49697184-49698035 bp	LT990438
					CXNH1	exon 1: NW_003159330.1: 3520372-3521373 bp	LT990439
					CXNI	exon 1: ch.13: 43316152-43317228 bp	LT990440
					CXNK1	exon 1: ch.12: 110525058-110526206 bp	LT990441
					CXNL	exon 1: ch.13: 43166541-43167860 bp	LT990442
					CXNM	exon 1: ch.13: 127605727-127607271 bp	LT990443
					CXNN	exon 1: ch.12: 76706801-76708456 bp	LT990444
					CXNP1	exon 1: NW_003159386.1: 223445-224194 bp	LT990445
					CXNP2	exon 1: NW_003159386.1: 267677-268501 bp	LT990446
					CXNQ	exon 1: ch.19: 45521101-45522291 bp	LT990447
					CXNR	exon 1: ch.19: 41523148-41524044 bp	LT990448
					CXNS	exon 1: ch.17: 37575030-37575842 bp	LT990449
					CXNT	exon 1: ch.12: 132653669-132653707 bp<br>exon 2: ch.12: 132654304-132654498 bp	LT990450

						exon 3: ch.12: 132655170-132655565 bp	
		American pika (*Ochotona princeps*)	GCF_000292845.1	7	*CXNA*	exon 1: NW_004535484.1: 9282196-9283008 bp	LT990451
					CXNB	exon 1: NW_004535484.1: 9276906-9277697 bp	LT990452
					CXNF	exon 1: NW_004535437.1: 72805790-72806575 bp	LT990453
					CXNH1	exon 1: NW_004535484.1: 9238072-9239115 bp	LT990454
					CXNL	exon 1: NW_004535447.1: 615350-616663 bp	LT990455
					CXNP1	exon 1: NW_004535461.1: 17730761-17731585 bp	LT990456
					CXNS	exon 1: NW_004535438.1: 61436731-61437543 bp	LT990457
					putative	exon 1: NW_004535437.1: 72846680-72847360 bp	-
					putative	exon 1: NW_004535437.1: 72903214-72904455 bp	-
					putative	exon 1: NW_004535447.1: 769690-770748 bp	-
					putative	exon 1: NW_004535460.1: 206229-207515 bp	-
					putative	exon 1: NW_004535462.1: 195921-197180 bp	-
					putative	exon 1: NW_004535462.1: 20968792-20968830 bp<br>exon 2: NW_004535462.1: 20969451-20969645 bp<br>exon 3: NW_004535462.1: 20970058-20970453 bp	-
					putative	exon 1: NW_004535484.1: 9248615-9249427 bp	-
					putative	exon 1: NW_004535510.1: 7818782-7819633 bp	-
					putative	exon 1: NW_004535524.1: 2307603-2308793 bp	-
Laurasiatheria	Cetacea	Bottlenose dolphin (*Tursiops truncatus*)	GCF_000151865.1	1	*CXND*	exon 1: NW_004201682.1: 85901-86707 bp	LT990458
					putative	exon 1: NW_004197566.1: 196485-197165 bp	-
					putative	exon 1: NW_004197566.1: 226310-227095 bp	-
					putative	exon 1: NW_004198801.1: 76829-78373 bp	-
					putative	exon 1: NW_004203194.1: 34270-35460 bp	-
					putative	exon 1: NW_004204816.1: 94925-96277 bp	-
					putative	exon 1: NW_004205116.1: 36101-37246 bp	-
					putative	exon 1: NW_004210970.1: 47418-48419 bp	-
					putative	exon 1: NW_004210970.1: 58096-58908 bp	-

					putative	exon 1: NW_004212466.1: 25919-26731 bp	-
					putative	exon 1: NW_004214893.1: 30954-32030 bp	-
					putative	exon 1: NW_004215273.1: 9994-11277 bp	-
					putative	exon 1: NW_004222048.1: 35179-35217 bp<br>exon 2: NW_004222048.1: 35897-36091 bp<br>exon 3: NW_004222048.1: 36645-37040 bp	-
	Artiodactyla	Domestic cattle (*Bos taurus*)	GCF_000003205.7	17	*CXNA*	exon 1: ch.3: 111933743-111934564 bp	LT990459
					CXNB	exon 1: ch.3: 111929662-111930462 bp	LT990460
					CXNC	exon 1: ch.3: 111903323-111904135 bp	LT990461
					CXND	exon 1: ch.9: 63791767-63792327 bp	LT990462
					CXNF	exon 1: ch.12: 36409418-36410203 bp	LT990463
					CXNG	exon 1: ch.X: 85700180-85701034 bp	LT990464
					CXNI	exon 1: ch.3: 22194539-22195618 bp	LT990465
					CXNJ1	exon 1: ch.12: 36466036-36467259 bp	LT990466
					CXNK1	exon 1: ch.9: 30251022-30252173 bp	LT990467
					CXNL	exon 1: ch.3: 22055958-22057280 bp	LT990468
					CXNN	exon 1: ch.9: 61533785-61535257 bp	LT990469
					CXNP1	exon 1: ch.25: 37181630-37182457 bp	LT990470
					CXNQ	exon 1: ch.19: 45335838-45337028 bp	LT990471
					CXNR	exon 1: ch.19: 41434662-41435516 bp	LT990472
					CXNS	exon 1: ch.10: 30447351-30448163 bp	LT990473
					CXNT	exon 1: ch.9: 81008296-81008334 bp<br>exon 2: ch.9: 81008995-81009189 bp<br>exon 3: ch.9: 81009723-81010118 bp	LT990474
					CXNU	exon 1: ch.13: 27125946-27126866 bp	LT990475
					putative	exon 1: ch.3: 108088832-108090373 bp	-
					putative	exon 1: ch.7: 2907746-2909035 bp	-
					putative	exon 1: ch.12: 36431158-36431952 bp	-
		Wild boar (*Sus scrofa*)	GCF_000003025.6	0	putative	exon 1: ch.1: 22936129-22936167 bp	-

						exon 2: ch.1: 22934927-22935121 bp<br>exon 3: ch.1: 22933885-22934280 bp	
					putative	exon 1: ch.1: 40990568-40991716 bp	-
					putative	exon 1: ch.1: 55730903-55731691 bp	-
					putative	exon 1: ch.1: 57879740-57881326 bp	-
					putative	exon 1: ch.1: 136325534-136326346 bp	-
					putative	exon 1: ch.2: 51265700-51266998 bp	-
					putative	exon 1: ch.3: 7843402-7844226 bp	-
					putative	exon 1: ch.4: 99723341-99724672 bp	-
					putative	exon 1: ch.4: 99868156-99869232 bp	-
					putative	exon 1: ch.6: 91002163-91002984 bp	-
					putative	exon 1: ch.6: 91007050-91008000 bp	-
					putative	exon 1: ch.6: 91035964-91036776 bp	-
					putative	exon 1: ch.6: 91046637-91047638 bp	-
					putative	exon 1: ch.6: 94815600-94817147 bp	-
					putative	exon 1: ch.11: 775166-775846 bp	-
					putative	exon 1: ch.11: 793914-794699 bp	-
					putative	exon 1: ch.12: 18554537-18555727 bp	-
					putative	exon 1: ch.12: 22041139-22042020 bp	-
					putative	exon 1: ch.X: 57248002-57248856 bp	-
		Vicugna (*Vicugna pacos*)	GCF_000164845.1	3	*CXNK1*	exon 1: NW_005882721.1: 13357039-13358187 bp	LT990476
					CXNQ	exon 1: NW_005882998.1: 1022284-1023474 bp	LT990477
					CXNT	exon 1: NW_005882708.1: 17155833-17155871 bp<br>exon 2: NW_005882708.1: 17154972-17155166 bp<br>exon 3: NW_005882708.1: 17154018-17154413 bp	LT990478
					putative	exon 1: NW_005882723.1: 9711786-9713294 bp	-
					putative	exon 1: NW_005882731.1: 1785565-1786377 bp	-
					putative	exon 1: NW_005882859.1: 492476-494023 bp	-

					putative	exon 1: NW_005882859.1: 3731676-3732677 bp	-
					putative	exon 1: NW_005882859.1: 3741616-3742428 bp	-
					putative	exon 1: NW_005882859.1: 3764708-3765508 bp	-
					putative	exon 1: NW_005882859.1: 3768587-3769408 bp	-
					putative	exon 1: NW_005882946.1: 2052287-2053069 bp	-
					putative	exon 1: NW_005882946.1: 2070831-2071511 bp	-
					putative	exon 1: NW_005882989.1: 1531017-1531994 bp	-
					putative	exon 1: NW_005883007.1: 356806-357660 bp	-
					putative	exon 1: NW_005883171.1: 162883-163890 bp	-
	Perissodactyla	Horse (*Equus caballus*)	GCF_000002305.2	20	*CXNA*	exon 1: ch.2: 22421662-22422483 bp	LT990479
					CXNB	exon 1: ch.2: 22417872-22418672 bp	LT990480
					CXNC	exon 1: ch.2: 22394644-22395459 bp	LT990481
					CXND	exon 1: ch.10: 40100971-40101834 bp	LT990482
					CXNE	exon 1: ch.17: 1081322-1082002 bp	LT990483
					CXNF	exon 1: ch.17: 1115647-1116432 bp	LT990484
					CXNG	exon 1: ch.X: 53095290-53096141 bp	LT990485
					CXNH1	exon 1: ch.2: 22384927-22385928 bp	LT990486
					CXNI	exon 1: ch.5: 48347997-48349073 bp	LT990487
					CXNJ1	exon 1: ch.17: 1038093-1039439 bp	LT990488
					CXNK1	exon 1: ch.10: 69022237-69023385 bp	LT990489
					CXNK2	exon 1: ch.X: 13584416-13585261 bp	LT990490
					CXNL	exon 1: ch.5: 48228678-48230003 bp	LT990491
					CXNM	exon 1: ch.2: 18986710-18988260 bp	LT990492
					CXNN	exon 1: ch.10: 42274709-42276172 bp	LT990493
					CXNP1	exon 1: ch.13: 7844187-7845011 bp	LT990494
					CXNQ	exon 1: ch.11: 18856123-18857313 bp	LT990495
					CXNS	exon 1: ch.1: 153026893-153027705 bp	LT990496

					CXNT	exon 1: ch.31: 23547266-23547304 bp<br>exon 2: ch.31: 23546390-23546584 bp<br>exon 3: ch.31: 23545443-23545835 bp	LT990497
					CXNU	exon 1: ch.29: 2733509-2734570 bp	LT990498
	Carnivora	Domestic dog (*Canis lupus familiaris*)	GCF_000002285.3	18	*CXNA*	exon 1: ch.15: 7196963-7197784 bp	LT990499
					CXNB	exon 1: ch.15: 7193413-7194363 bp	LT990500
					CXNC	exon 1: ch.15: 7175729-7176541 bp	LT990501
					CXND	exon 1: ch.12: 46801797-46802588 bp	LT990502
					CXNE	exon 1: ch.25: 17956229-17956909 bp	LT990503
					CXNF	exon 1: ch.25: 17920820-17921605 bp	LT990504
					CXNG	exon 1: ch.X: 55573808-55574659 bp	LT990505
					CXNH1	exon 1: ch.15: 7168197-7169198 bp	LT990506
					CXNI	exon 1: ch.17: 58289793-58290869 bp	LT990507
					CXNK1	exon 1: ch.1: 60941788-60942936 bp	LT990508
					CXNK2	exon 1: ch.X: 14622023-14622946 bp	LT990509
					CXNL	exon 1: ch.17: 58424072-58425391 bp	LT990510
					CXNM	exon 1: ch.15: 3862418-3863962 bp	LT990511
					CXNN	exon 1: ch.12: 49092747-49094210 bp	LT990512
					CXNP1	exon 1: ch.6: 9655078-9655983 bp	LT990513
					CXNQ	exon 1: ch.9: 18686527-18687717 bp	LT990514
					CXNS	exon 1: ch.30: 2478734-2479546 bp	LT990515
					CXNT	exon 1: ch.1: 33820248-33820286 bp<br>exon 2: ch.1: 33820967-33821161 bp<br>exon 3: ch.1: 33821682-33822077 bp	LT990516
					putative	exon 1: ch.25: 18002817-18004160 bp	-
		Domestic cat (*Felis catus*)	GCF_000181335.2	9	*CXNA*	exon 1: ch.C1: 26641598-26642419 bp	LT990517
					CXNC	exon 1: ch.C1: 26668524-26669336 bp	LT990518
					CXNE	exon 1: ch.A1: 1182406-1183086 bp	LT990519
					CXNG	exon 1: ch.X: 57716355-57717206 bp	LT990520

					CXNH1	exon 1: ch.C1: 26677847-26678848 bp	LT990521
					CXNI	exon 1: ch.C1: 104031646-104032722 bp	LT990522
					CXNK1	exon 1: ch.X: 14634136-14635035 bp	LT990523
					CXNL	exon 1: ch.C1: 104156555-104157877 bp	LT990524
					CXNP1	exon 1: ch.E3: 7103056-7103943 bp	LT990525
					putative	exon 1: ch.A1: 1215417-1216202 bp	-
					putative	exon 1: ch.B2: 78687439-78688302 bp	-
					putative	exon 1: ch.B2: 81025505-81026956 bp	-
					putative	exon 1: ch.B2: 109929370-109930518 bp	-
					putative	exon 1: ch.B2: 128641146-128641184 bp<br>exon 2: ch.B2: 128641871-128642065 bp<br>exon 3: ch.B2: 128642585-128642980 bp	-
					putative	exon 1: ch.B3: 68162774-68163586 bp	-
					putative	exon 1: ch.C1: 26645561-26646361 bp	-
					putative	exon 1: ch.C1: 30096586-30098124 bp	-
					putative	exon 1: ch.E1: 42553193-42554383 bp	-
	Chiroptera	Little brown myotis (*Myotis lucifugus*)	GCF_000147115.1	16	*CXNA*	exon 1: NW_005871179.1: 1314808-1315701 bp	LT990526
					CXNB	exon 1: NW_005871179.1: 1310849-1311652 bp	LT990527
					CXNC	exon 1: NW_005871179.1: 1282693-1283502 bp	LT990528
					CXND	exon 1: NW_005871200.1: 1926846-1927646 bp	LT990529
					CXNE	exon 1: NW_005871067.1: 4281786-4282466 bp	LT990530
					CXNF	exon 1: NW_005871067.1: 4312762-4313547 bp	LT990531
					CXNG	exon 1: NW_005871052.1: 12953349-12954200 bp	LT990532
					CXNH1	exon 1: NW_005871179.1: 1266220-1267305 bp	LT990533
					CXNJ1	exon 1: NW_005871067.1: 2958345-2959118 bp	LT990534
					CXNJ2	exon 1: NW_005871067.1: 2988781-2989590 bp	LT990535
					CXNK1	exon 1: NW_005871105.1: 4032039-4033187 bp	LT990536
					CXNP1	exon 1: NW_005871048.1: 890447-891385 bp	LT990537

					CXNQ	exon 1: NW_005871814.1: 193962-195152 bp	LT990538
					CXNS	exon 1: NW_005871086.1: 4177462-4178274 bp	LT990539
					CXNT	exon 1: NW_005871059.1: 8483259-8483297 bp<br>exon 2: NW_005871059.1: 8482409-8482603 bp<br>exon 3: NW_005871059.1: 8481510-8481905 bp	LT990540
					CXNU	exon 1: NW_005871077.1: 8469532-8470593 bp	LT990541
		Large flying fox (*Pteropus vampyrus*)	GCF_000151845.1	7	*CXNG*	exon 1: NW_011888972.1: 273378-274229 bp	LT990542
					CXNI	exon 1: NW_011888787.1: 440448-441524 bp	LT990543
					CXNJ1	exon 1: NW_011888833.1: 3554648-3556447 bp	LT990544
					CXNJ2	exon 1: NW_011888833.1: 3619646-3620695 bp	LT990545
					CXNL	exon 1: NW_011888787.1: 339573-340895 bp	LT990546
					CXNM	exon 1: NW_011889216.1: 429276-430811 bp	LT990547
					CXNQ	exon 1: NW_011889087.1: 1654821-1656011 bp	LT990548
					putative	exon 1: NW_011888800.1: 1748653-1749465 bp	-
					putative	exon 1: NW_011888805.1: 6113597-6113635 bp<br>exon 2: NW_011888805.1: 6112711-6112905 bp<br>exon 3: NW_011888805.1: 6111804-6112199 bp	-
					putative	exon 1: NW_011888817.1: 11121399-11122196 bp	-
					putative	exon 1: NW_011888817.1: 11125264-11126067 bp	-
					putative	exon 1: NW_011888817.1: 11150432-11151244 bp	-
					putative	exon 1: NW_011888817.1: 11160295-11161293 bp	-
					putative	exon 1: NW_011888824.1: 8608513-8609661 bp	-
					putative	exon 1: NW_011888831.1: 6605571-6606452 bp	-
					putative	exon 1: NW_011888833.1: 3590592-3591545 bp	-
					putative	exon 1: NW_011888833.1: 3635855-3636535 bp	-
					putative	exon 1: NW_011888833.1: 3657003-3657788 bp	-
					putative	exon 1: NW_011888914.1: 3135569-3137170 bp	-
					putative	exon 1: NW_011888914.1: 5067811-5068617 bp	-
					putative	exon 1: NW_011889262.1: 445860-446864 bp	-

					putative	exon 1: NW_011889881.1: 58409-59236 bp	-
					putative	exon 1: NW_011913342.1: 1513-2511 bp	-
	Eulipotyphla	West European hedgehog (*Erinaceus europaeus*)	GCF_000296755.1	3	*CXNB*	exon 1: NW_006804122.1: 280824-281624 bp	LT990549
					CXND	exon 1: NW_006804319.1: 974965-975774 bp	LT990550
					CXNI	exon 1: NW_006804317.1: 446907-447989 bp	LT990551
					putative	exon 1: NW_006803952.1: 3186797-3187945 bp	-
					putative	exon 1: NW_006804069.1: 2706281-2707429 bp	-
					putative	exon 1: NW_006804078.1: 2749981-2750793 bp	-
					putative	exon 1: NW_006804122.1: 222920-223921 bp	-
					putative	exon 1: NW_006804122.1: 239210-240022 bp	-
					putative	exon 1: NW_006804122.1: 285041-285859 bp	-
					putative	exon 1: NW_006804147.1: 1190118-1191395 bp	-
					putative	exon 1: NW_006804250.1: 1582307-1583839 bp	-
					putative	exon 1: NW_006804317.1: 302559-303881 bp	-
					putative	exon 1: NW_006804352.1: 191266-192348 bp	-
					putative	exon 1: NW_006804352.1: 259352-260032 bp	-
					putative	exon 1: NW_006804352.1: 286225-287007 bp	-
					putative	exon 1: NW_006804407.1: 99732-101228 bp	-
					putative	exon 1: NW_006804682.1: 941945-943198 bp	-
					putative	exon 1: NW_006804768.1: 770740-771930 bp	-
					putative	exon 1: NW_006804792.1: 308654-309505 bp	-
		Common shrew (*Sorex araneus*)	GCF_000181275.1	5	*CXNE*	exon 1: NW_004545868.1: 7790932-7791612 bp	LT990552
					CXNJ1	exon 1: NW_004545868.1: 7859140-7860003 bp	LT990553
					CXNL	exon 1: NW_004545990.1: 451429-452565 bp	LT990554
					CXNN	exon 1: NW_004545872.1: 5079644-5081119 bp	LT990555
					CXNU	exon 1: NW_004545870.1: 21763782-21764744 bp	LT990556
					putative	exon 1: NW_004545868.1: 7748347-7749132 bp	-

					putative	exon 1: NW_004545872.1: 2666683-2667489 bp	-
					putative	exon 1: NW_004545893.1: 7754788-7755801 bp	-
					putative	exon 1: NW_004545924.1: 6131290-6132438 bp	-
					putative	exon 1: NW_004545928.1: 4795125-4796645 bp	-
					putative	exon 1: NW_004545928.1: 8428172-8429236 bp	-
					putative	exon 1: NW_004545928.1: 8436291-8437091 bp	-
					putative	exon 1: NW_004545928.1: 8461072-8461872 bp	-
					putative	exon 1: NW_004545928.1: 8464946-8465731 bp	-
					putative	exon 1: NW_004545960.1: 816258-817112 bp	-
					putative	exon 1: NW_004545990.1: 606227-607279 bp	-
					putative	exon 1: NW_004546032.1: 1209894-1211084 bp	-
					putative	exon 1: NW_004546052.1: 535685-536536 bp	-
Xenarthra	Xenarthra	Nine-banded armadillo (*Dasypus novemcinctus*)	GCF_000208655.1	18	*CXNA*	exon 1: NW_004481703.1: 476079-476900 bp	LT990557
					CXNC	exon 1: NW_004481703.1: 510337-511155 bp	LT990558
					CXND	exon 1: NW_004472605.1: 435861-436667 bp	LT990559
					CXNE	exon 1: NW_004482607.1: 8721-9401 bp	LT990560
					CXNF	exon 1: NW_004482607.1: 42289-43074 bp	LT990561
					CXNG	exon 1: NW_004496608.1: 99131-99982 bp	LT990562
					CXNH1	exon 1: NW_004481703.1: 518876-519877 bp	LT990563
					CXNI	exon 1: NW_004482974.1: 1155814-1156890 bp	LT990564
					CXNK1	exon 1: NW_004465862.1: 556547-557287 bp	LT990565
					CXNK2	exon 1: NW_004483017.1: 132328-133479 bp	LT990566
					CXNL	exon 1: NW_004480641.1: 70133-71461 bp	LT990567
					CXNP1	exon 1: NW_004479389.1: 302121-302876 bp	LT990568
					CXNP2	exon 1: NW_004479389.1: 341460-342215 bp	LT990569
					CXNP3	exon 1: NW_004479389.1: 381829-382818 bp	LT990570
					CXNP4	exon 1: NW_004479389.1: 473522-474277 bp	LT990571

					CXNS	exon 1: NW_004473855.1: 2006020-2006832 bp	LT990572
					CXNT	exon 1: NW_004468059.1: 549749-549787 bp<br>exon 2: NW_004468059.1: 550453-550647 bp<br>exon 3: NW_004468059.1: 551179-551574 bp	LT990573
					CXNU	exon 1: NW_004490346.1: 4058719-4059834 bp	LT990574
					putative	exon 1: NW_004480269.1: 96724-97914 bp	-
		Hoffmann's two-toed sloth (*Choloepus hoffmanni*)	GCA_000164785.2	3	*CXND*	exon 1: KN188820.1: 27035-27841 bp	LT990575
					CXNF	exon 1: KN194419.1: 145387-146172 bp	LT990576
					CXNP1	exon 1: KN189092.1: 672301-673056 bp	LT990577
					putative	exon 1: KN175891.1: 15985-16881 bp	-
					putative	exon 1: KN175891.1: 23263-24264 bp	-
					putative	exon 1: KN177995.1: 87345-88661 bp	-
					putative	exon 1: KN178164.1: 187045-187896 bp	-
					putative	exon 1: KN179942.1: 25327-26517 bp	-
					putative	exon 1: KN180552.1: 85810-86985 bp	-
					putative	exon 1: KN184972.1: 171302-172474 bp	-
					putative	exon 1: KN189092.1: 631745-632728 bp	-
					putative	exon 1: KN191310.1: 841351-842499 bp	-
					putative	exon 1: KN191369.1: 184916-185728 bp	-
					putative	exon 1: KN193759.1: 316796-316834 bp<br>exon 2: KN193759.1: 315937-316131 bp<br>exon 3: KN193759.1: 315020-315415 bp	-
					putative	exon 1: KN194419.1: 54290-55633 bp	-
					putative	exon 1: KN194419.1: 116515-117195 bp	-
					putative	exon 1: KN195032.1: 804492-805415 bp	-
Afrotheria	Tenrecidae	Lesser hedgehog tenrec (*Echinops telfairi*)	GCF_000313985.1	0	putative	exon 1: NW_004558701.1: 74706271-74707131 bp	-
					putative	exon 1: NW_004558711.1: 27392444-27392482 bp<br>exon 2: NW_004558711.1: 27391563-27391757 bp<br>exon 3: NW_004558711.1: 27390475-27390870 bp	-

					putative	exon 1: NW_004558711.1: 53067214-53068362 bp	-
					putative	exon 1: NW_004558712.1: 13417161-13418210 bp	-
					putative	exon 1: NW_004558721.1: 9276064-9277065 bp	-
					putative	exon 1: NW_004558721.1: 9295623-9296444 bp	-
					putative	exon 1: NW_004558721.1: 9358837-9359649 bp	-
					putative	exon 1: NW_004558722.1: 9921488-9922324 bp	-
					putative	exon 1: NW_004558727.1: 31829265-31830455 bp	-
					putative	exon 1: NW_004558743.1: 8811716-8812750 bp	-
					putative	exon 1: NW_004558768.1: 2558184-2558996 bp	-
					putative	exon 1: NW_004558794.1: 5503866-5504651 bp	-
					putative	exon 1: NW_004558794.1: 5542315-5542995 bp	-
					putative	exon 1: NW_004558794.1: 5645507-5646790 bp	-
					putative	exon 1: NW_004558830.1: 461206-462057 bp	-
					putative	exon 1: NW_004558844.1: 1219841-1220917 bp	-
					putative	exon 1: NW_004558844.1: 1411818-1413092 bp	-
	Proboscidea	African bush elephant (*Loxodonta africana*)	GCF_000001905.1	19	*CXNA*	exon 1: NW_003573454.1: 13688738-13689559 bp	LT990578
					CXNB	exon 1: NW_003573454.1: 13692747-13693547 bp	LT990579
					CXNC	exon 1: NW_003573454.1: 13760661-13761461 bp	LT990580
					CXND	exon 1: NW_003573420.1: 78429550-78430347 bp	LT990581
					CXNE	exon 1: NW_003573449.1: 3472195-3472875 bp	LT990582
					CXNF	exon 1: NW_003573449.1: 3517663-3518448 bp	LT990583
					CXNG	exon 1: NW_003573444.1: 31314767-31315618 bp	LT990584
					CXNH1	exon 1: NW_003573454.1: 13772159-13773160 bp	LT990585
					CXNI	exon 1: NW_003573547.1: 1001041-1002117 bp	LT990586
					CXNK1	exon 1: NW_003573420.1: 40617298-40618446 bp	LT990587
					CXNK2	exon 1: NW_003573459.1: 13038682-13039626 bp	LT990588
					CXNL	exon 1: NW_003573547.1: 1165799-1167112 bp	LT990589

					CXNM	exon 1: NW_003573454.1: 18272874-18274403 bp	LT990590
					CXNN	exon 1: NW_003573420.1: 75732313-75733782 bp	LT990591
					CXNP1	exon 1: NW_003573572.1: 910230-910664 bp	LT990592
					CXNQ	exon 1: NW_003573451.1: 25921239-25922417 bp	LT990593
					CXNS	exon 1: NW_003573484.1: 3831583-3832779 bp	LT990594
					CXNT	exon 1: NW_003573420.1: 16996081-16996119 bp<br>exon 2: NW_003573420.1: 16995197-16995379 bp<br>exon 3: NW_003573420.1: 16994414-16994803 bp	LT990595
					CXNU	exon 1: NW_003573517.1: 630455-631663 bp	LT990596
					putative	exon 1: NW_003573449.1: 3340723-3342072 bp	-
	Hyracoidea	Rock hyrax (*Procavia capensis*)	GCA_000152225.2	1	*CXNG*	exon 1: KN678155.1: 554687-555538 bp	LT990597
					putative	exon 1: KN676606.1: 464118-465110 bp	-
					putative	exon 1: KN676318.1: 1553402-1554787 bp	-
					putative	exon 1: KN676381.1: 1551632-1552450 bp	-
					putative	exon 1: KN676381.1: 1555568-1556368 bp	-
					putative	exon 1: KN676381.1: 1625515-1626327 bp	-
					putative	exon 1: KN676381.1: 1637472-1638473 bp	-
					putative	exon 1: KN676734.1: 1354602-1355645 bp	-
					putative	exon 1: KN677021.1: 30788-31717 bp	-
					putative	exon 1: KN677126.1: 629868-631400 bp	-
					putative	exon 1: KN677341.1: 806256-807518 bp	-
					putative	exon 1: KN677944.1: 468488-469564 bp	-
					putative	exon 1: KN677944.1: 656019-657332 bp	-
					putative	exon 1: KN678008.1: 551860-553008 bp	-
					putative	exon 1: KN678092.1: 72592-73800 bp	-
					putative	exon 1: KN678396.1: 288227-289636 bp	-
					putative	exon 1: KN678396.1: 417935-418615 bp	-
					putative	exon 1: KN678396.1: 453609-454394 bp	-

					putative	exon 1: KN679470.1: 138184-139482 bp	-

[a], the eutherian species common names were cited from Wilson and Reeder[31]; [b], the human connexin gene and protein nomenclatures provided in parentheses were cited from Beyer and Berthoud[8,9], Söhl and Willecke[25] and Fishman et al.[52]; [c], translated exons; ch., chromosome.

Supplementary data file 2: Multiple pairwise genomic sequence alignments of eutherian connexin genes. In base sequences (*Homo sapiens*), the translated genomic sequence regions were displayed as indigo rectangles (top). Accordingly, the genomic sequence regions including sequence identity levels above empirical cut-offs of detection of common genomic sequence regions were shown in multiple pairwise alignments.

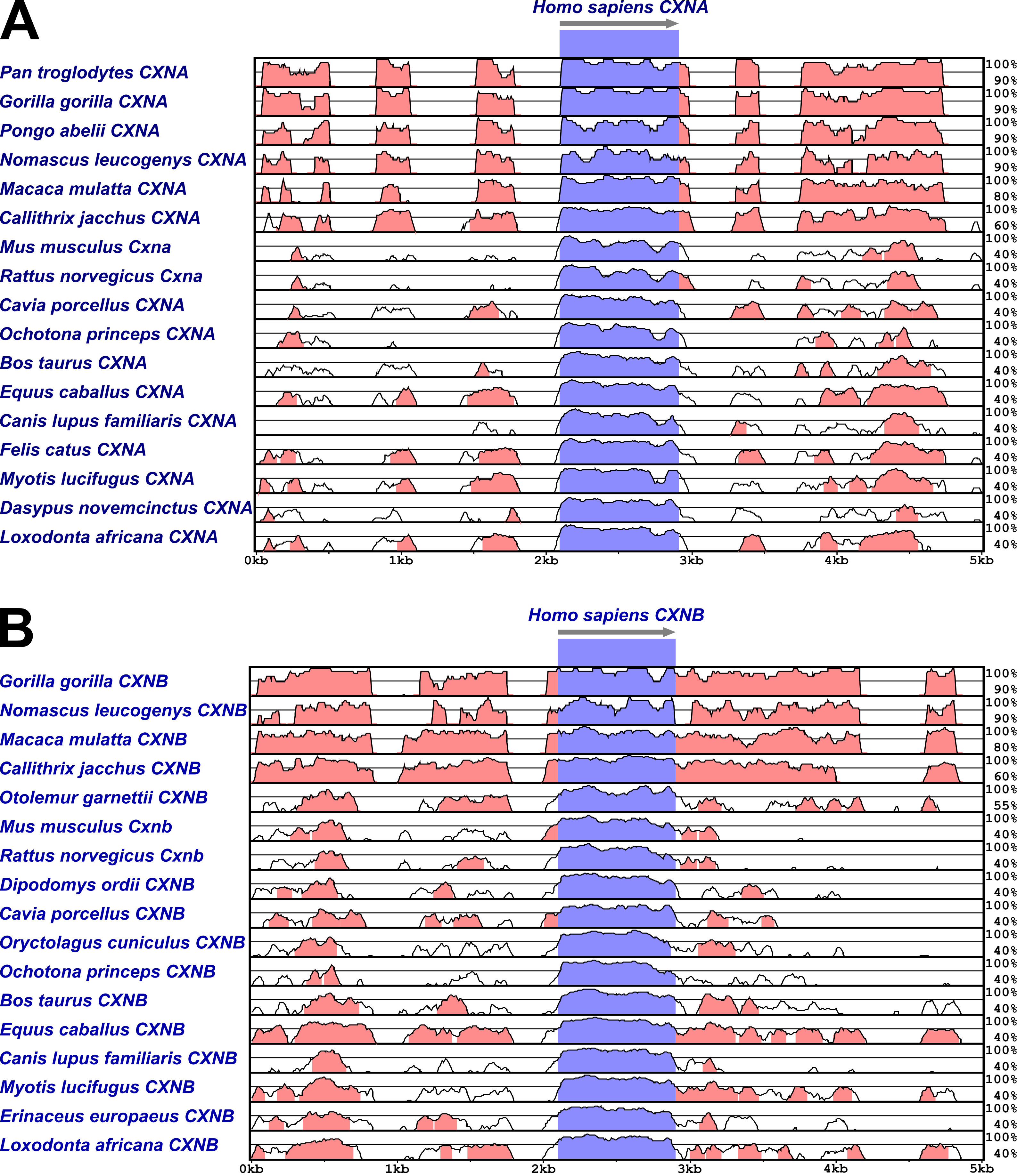

A
Homo sapiens CXNA
Pan troglodytes CXNA
Gorilla gorilla CXNA
Pongo abelii CXNA
Nomascus leucogenys CXNA
Macaca mulatta CXNA
Callithrix jacchus CXNA
Mus musculus Cxna
Rattus norvegicus Cxna
Cavia porcellus CXNA
Ochotona princeps CXNA
Bos taurus CXNA
Equus caballus CXNA
Canis lupus familiaris CXNA
Felis catus CXNA
Myotis lucifugus CXNA
Dasypus novemcinctus CXNA
Loxodonta africana CXNA
100%
90%
80%
60%
40%
0kb
1kb
2kb
3kb
4kb
5kb
B
Homo sapiens CXNB
Gorilla gorilla CXNB
Nomascus leucogenys CXNB
Macaca mulatta CXNB
Callithrix jacchus CXNB
Otolemur garnettii CXNB
Mus musculus Cxnb
Rattus norvegicus Cxnb
Dipodomys ordii CXNB
Cavia porcellus CXNB
Oryctolagus cuniculus CXNB
Ochotona princeps CXNB
Bos taurus CXNB
Equus caballus CXNB
Canis lupus familiaris CXNB
Myotis lucifugus CXNB
Erinaceus europaeus CXNB
Loxodonta africana CXNB
100%
90%
80%
60%
55%
40%
0kb
1kb
2kb
3kb
4kb
5kb

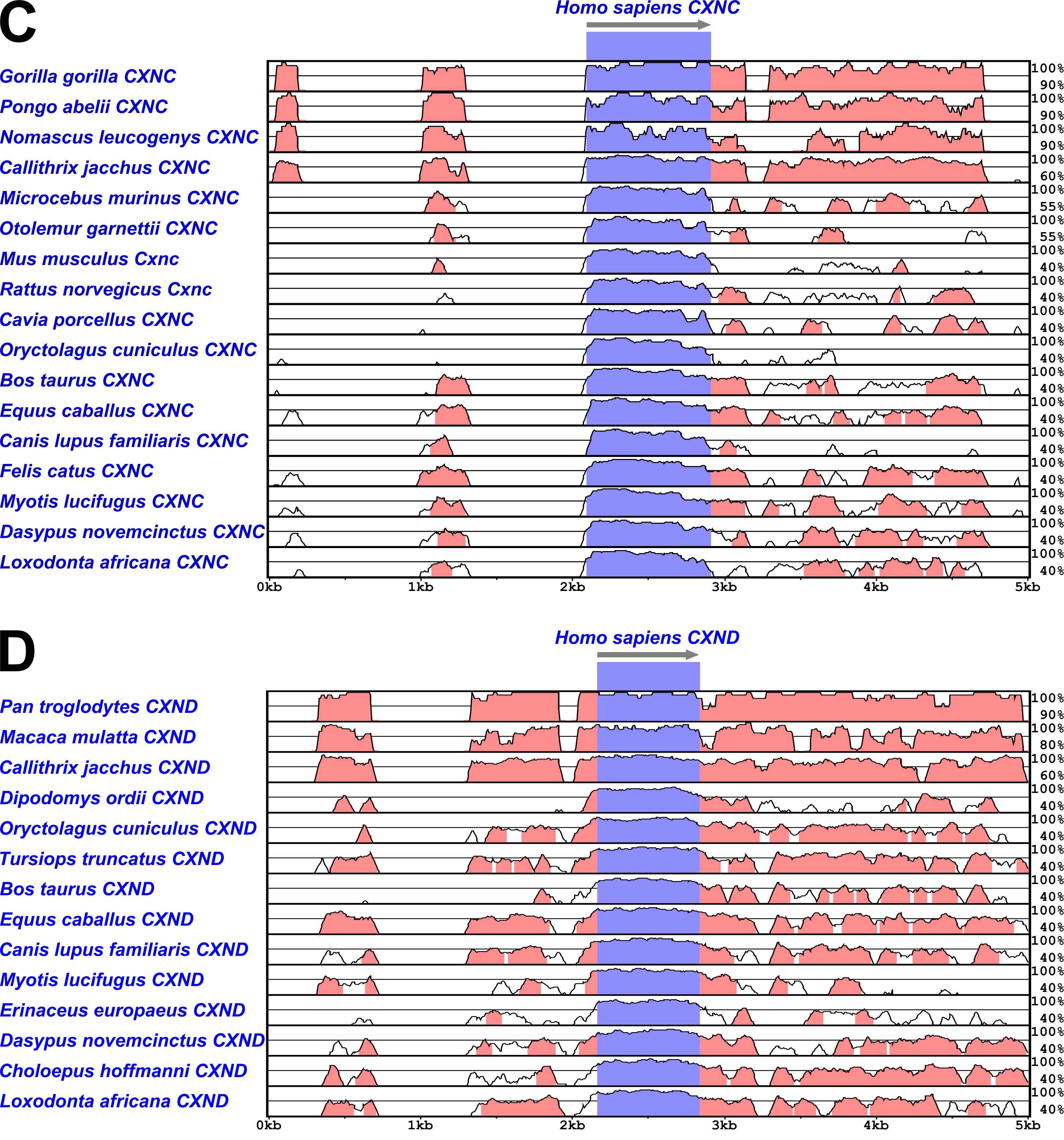
C
Homo sapiens CXNC
Gorilla gorilla CXNC
Pongo abelii CXNC
Nomascus leucogenys CXNC
Callithrix jacchus CXNC
Microcebus murinus CXNC
Otolemur garnettii CXNC
Mus musculus Cxnc
Rattus norvegicus Cxnc
Cavia porcellus CXNC
Oryctolagus cuniculus CXNC
Bos taurus CXNC
Equus caballus CXNC
Canis lupus familiaris CXNC
Felis catus CXNC
Myotis lucifugus CXNC
Dasypus novemcinctus CXNC
Loxodonta africana CXNC
100%
90%
60%
55%
40%
0kb
1kb
2kb
3kb
4kb
5kb
D
Homo sapiens CXND
Pan troglodytes CXND
Macaca mulatta CXND
Callithrix jacchus CXND
Dipodomys ordii CXND
Oryctolagus cuniculus CXND
Tursiops truncatus CXND
Bos taurus CXND
Equus caballus CXND
Canis lupus familiaris CXND
Myotis lucifugus CXND
Erinaceus europaeus CXND
Dasypus novemcinctus CXND
Choloepus hoffmanni CXND
Loxodonta africana CXND
100%
90%
80%
60%
40%
0kb
1kb
2kb
3kb
4kb
5kb

E

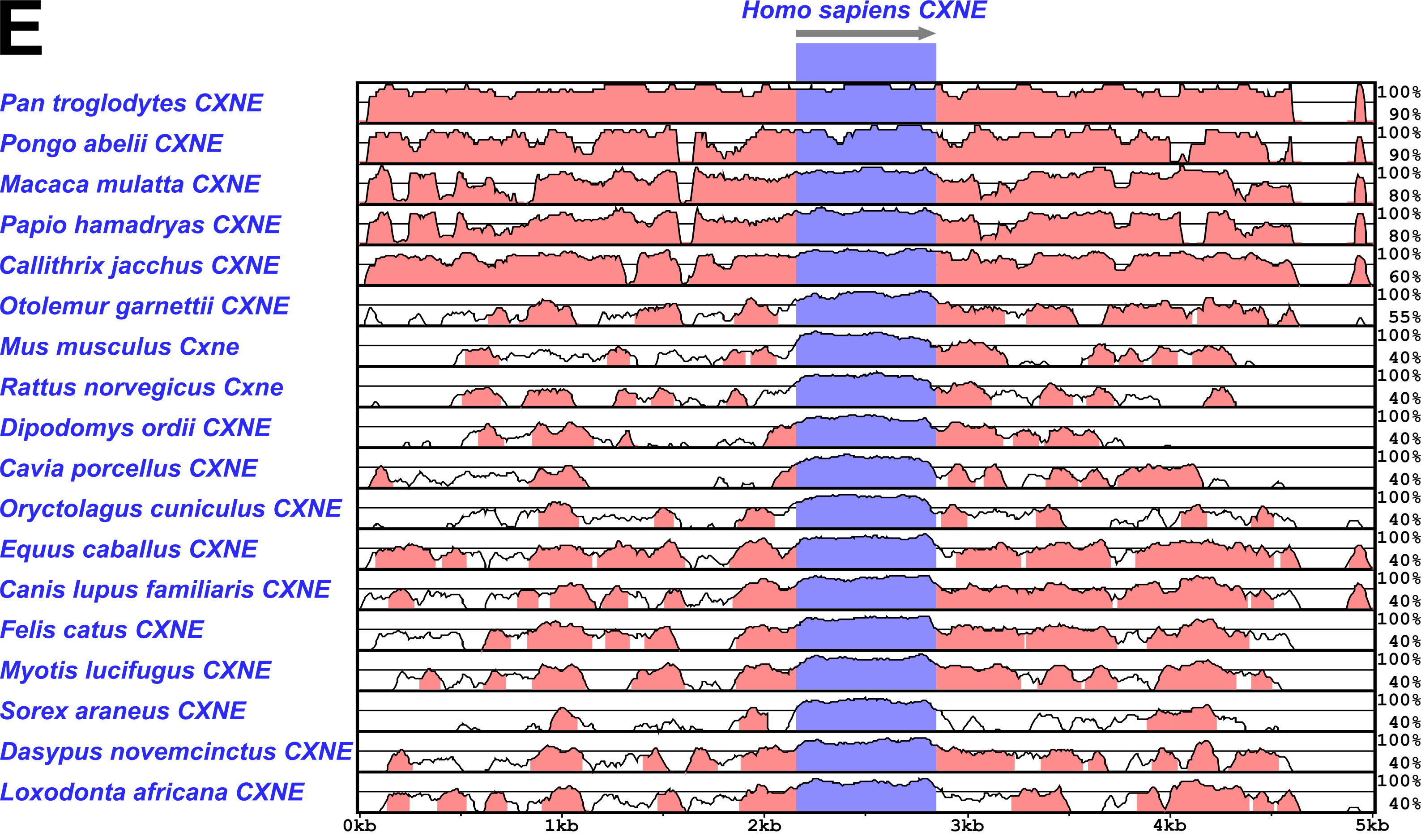

Homo sapiens CXNE
Pan troglodytes CXNE
Pongo abelii CXNE
Macaca mulatta CXNE
Papio hamadryas CXNE
Callithrix jacchus CXNE
Otolemur garnettii CXNE
Mus musculus Cxne
Rattus norvegicus Cxne
Dipodomys ordii CXNE
Cavia porcellus CXNE
Oryctolagus cuniculus CXNE
Equus caballus CXNE
Canis lupus familiaris CXNE
Felis catus CXNE
Myotis lucifugus CXNE
Sorex araneus CXNE
Dasypus novemcinctus CXNE
Loxodonta africana CXNE
100%
90%
80%
60%
55%
40%
0kb
1kb
2kb
3kb
4kb
5kb

F

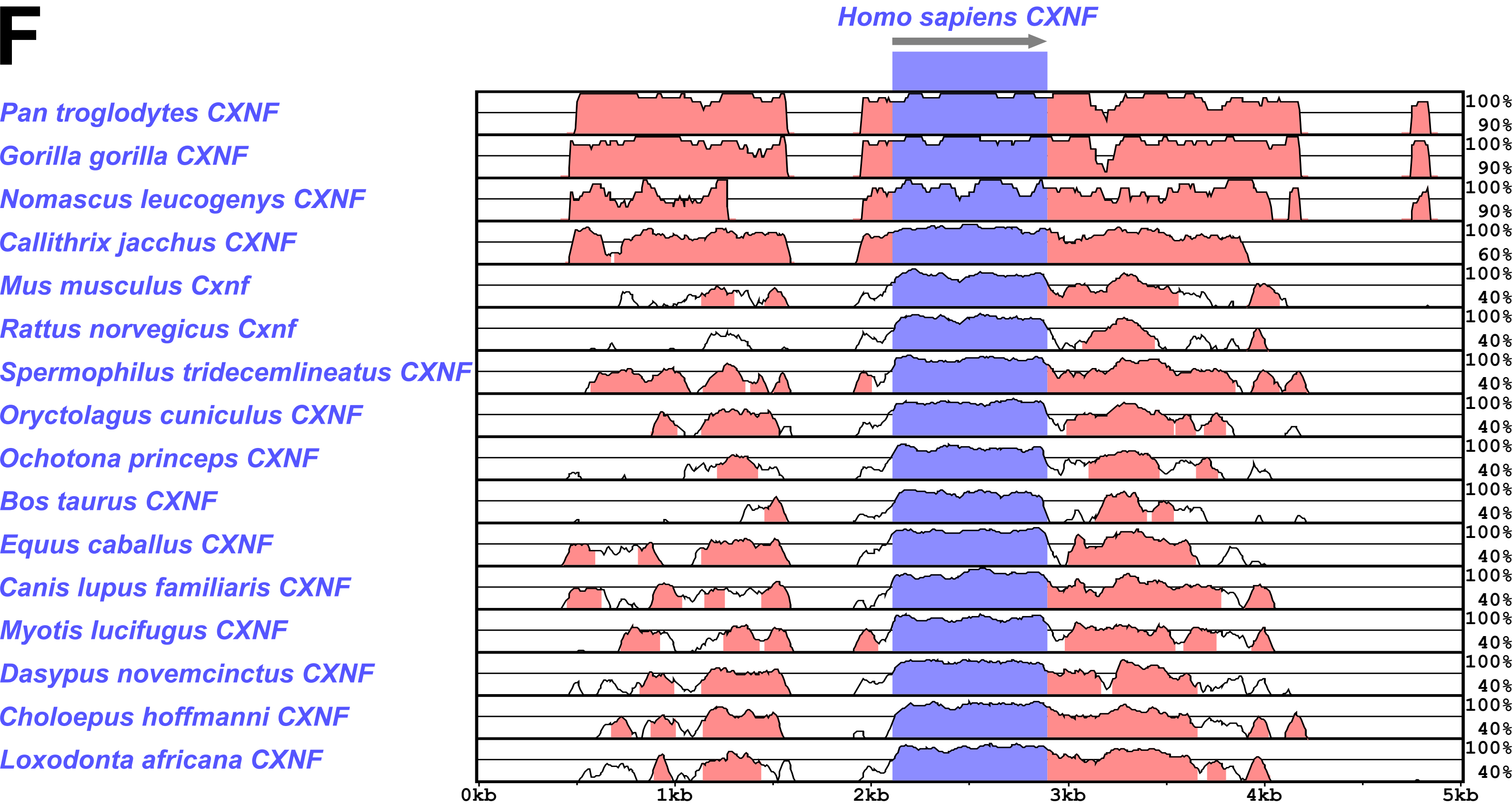

Homo sapiens CXNF
Pan troglodytes CXNF
Gorilla gorilla CXNF
Nomascus leucogenys CXNF
Callithrix jacchus CXNF
Mus musculus Cxnf
Rattus norvegicus Cxnf
Spermophilus tridecemlineatus CXNF
Oryctolagus cuniculus CXNF
Ochotona princeps CXNF
Bos taurus CXNF
Equus caballus CXNF
Canis lupus familiaris CXNF
Myotis lucifugus CXNF
Dasypus novemcinctus CXNF
Choloepus hoffmanni CXNF
Loxodonta africana CXNF
100%
90%
60%
40%
0kb
1kb
2kb
3kb
4kb
5kb

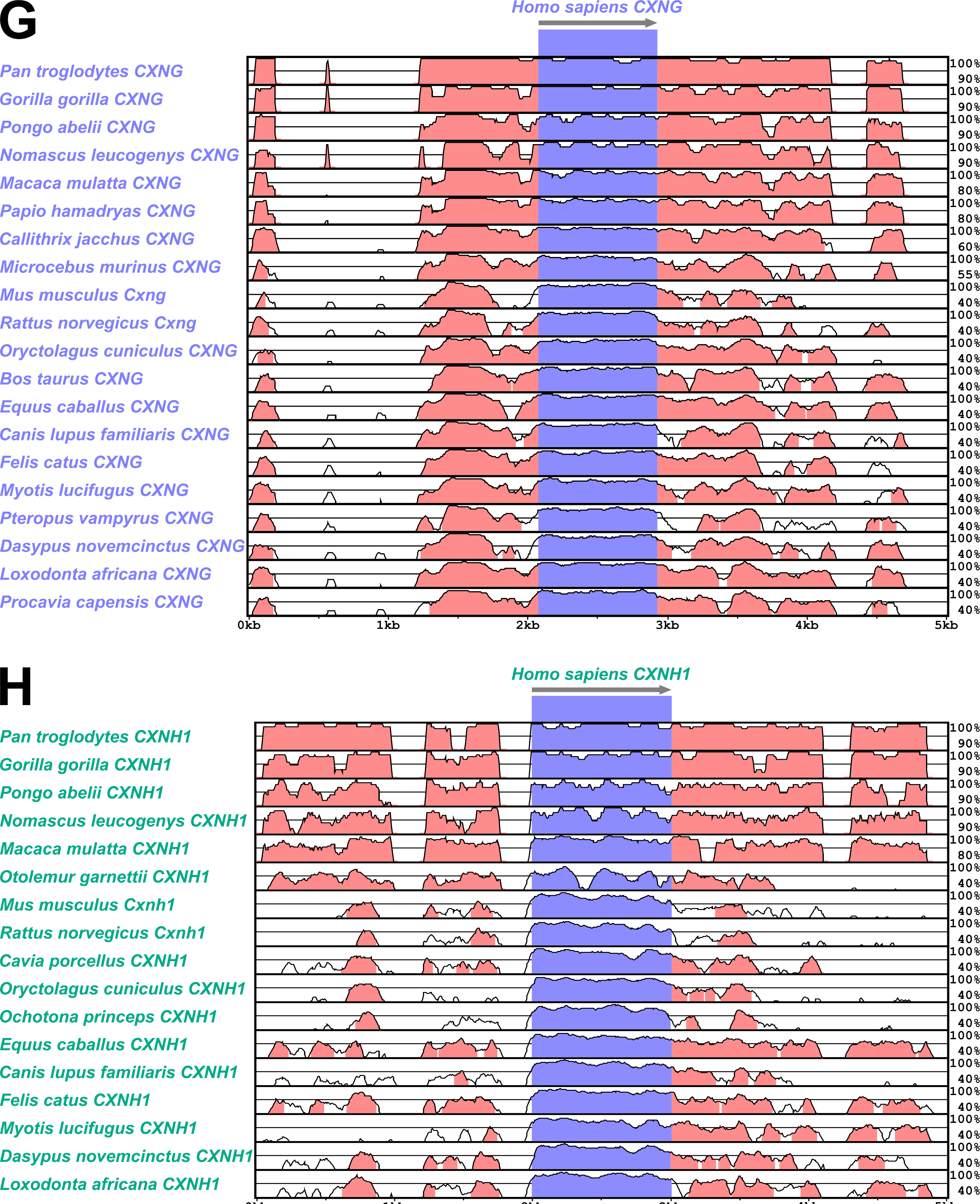
G
Homo sapiens CXNG
Pan troglodytes CXNG
Gorilla gorilla CXNG
Pongo abelii CXNG
Nomascus leucogenys CXNG
Macaca mulatta CXNG
Papio hamadryas CXNG
Callithrix jacchus CXNG
Microcebus murinus CXNG
Mus musculus Cxng
Rattus norvegicus Cxng
Oryctolagus cuniculus CXNG
Bos taurus CXNG
Equus caballus CXNG
Canis lupus familiaris CXNG
Felis catus CXNG
Myotis lucifugus CXNG
Pteropus vampyrus CXNG
Dasypus novemcinctus CXNG
Loxodonta africana CXNG
Procavia capensis CXNG
100%
90%
80%
60%
55%
40%
0kb
1kb
2kb
3kb
4kb
5kb
H
Homo sapiens CXNH1
Pan troglodytes CXNH1
Gorilla gorilla CXNH1
Pongo abelii CXNH1
Nomascus leucogenys CXNH1
Macaca mulatta CXNH1
Otolemur garnettii CXNH1
Mus musculus Cxnh1
Rattus norvegicus Cxnh1
Cavia porcellus CXNH1
Oryctolagus cuniculus CXNH1
Ochotona princeps CXNH1
Equus caballus CXNH1
Canis lupus familiaris CXNH1
Felis catus CXNH1
Myotis lucifugus CXNH1
Dasypus novemcinctus CXNH1
Loxodonta africana CXNH1
100%
90%
80%
40%
0kb
1kb
2kb
3kb
4kb
5kb

I

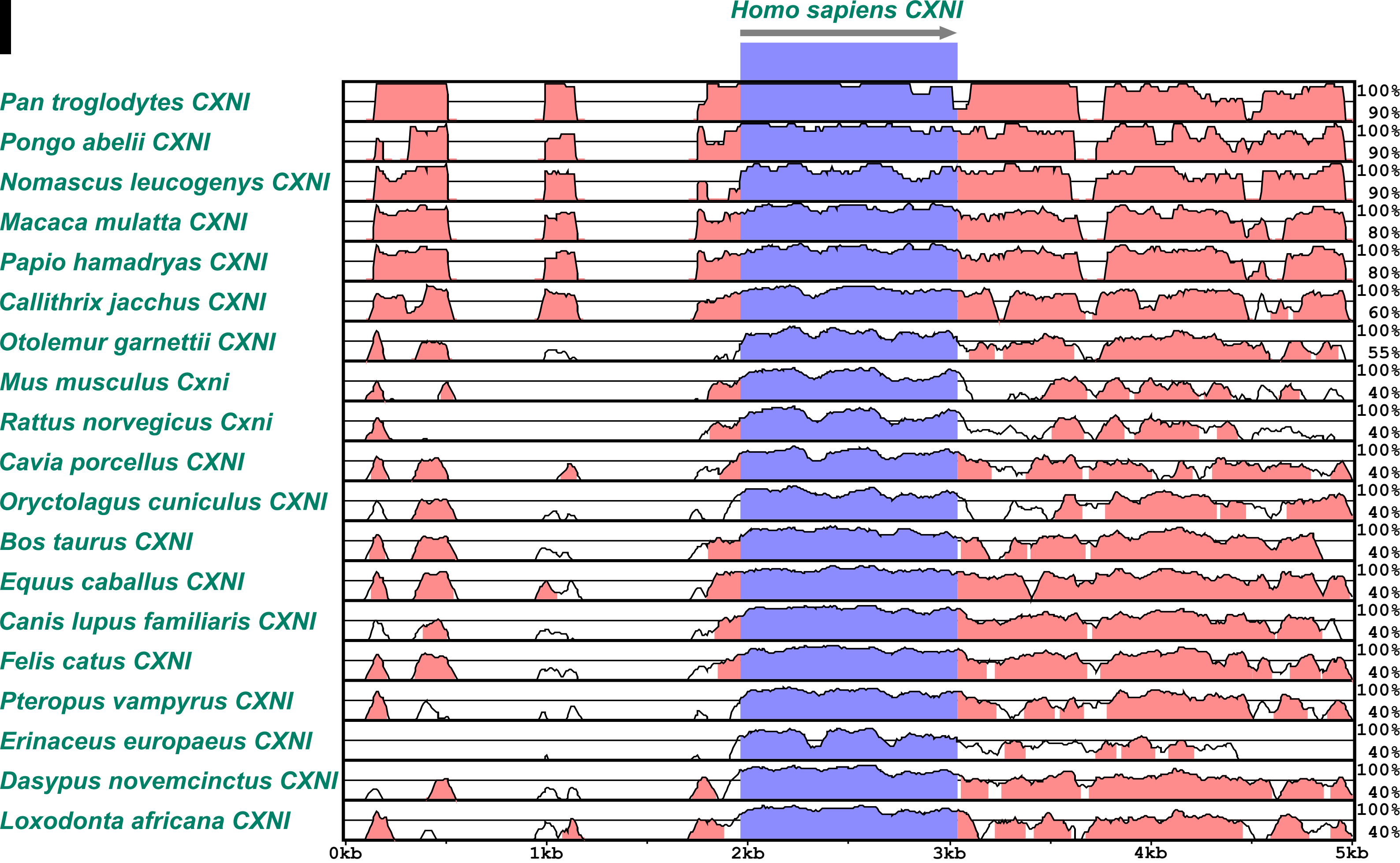
Homo sapiens CXNI
Pan troglodytes CXNI
Pongo abelii CXNI
Nomascus leucogenys CXNI
Macaca mulatta CXNI
Papio hamadryas CXNI
Callithrix jacchus CXNI
Otolemur garnettii CXNI
Mus musculus Cxni
Rattus norvegicus Cxni
Cavia porcellus CXNI
Oryctolagus cuniculus CXNI
Bos taurus CXNI
Equus caballus CXNI
Canis lupus familiaris CXNI
Felis catus CXNI
Pteropus vampyrus CXNI
Erinaceus europaeus CXNI
Dasypus novemcinctus CXNI
Loxodonta africana CXNI
100%
90%
80%
60%
55%
40%
0kb
1kb
2kb
3kb
4kb
5kb

J

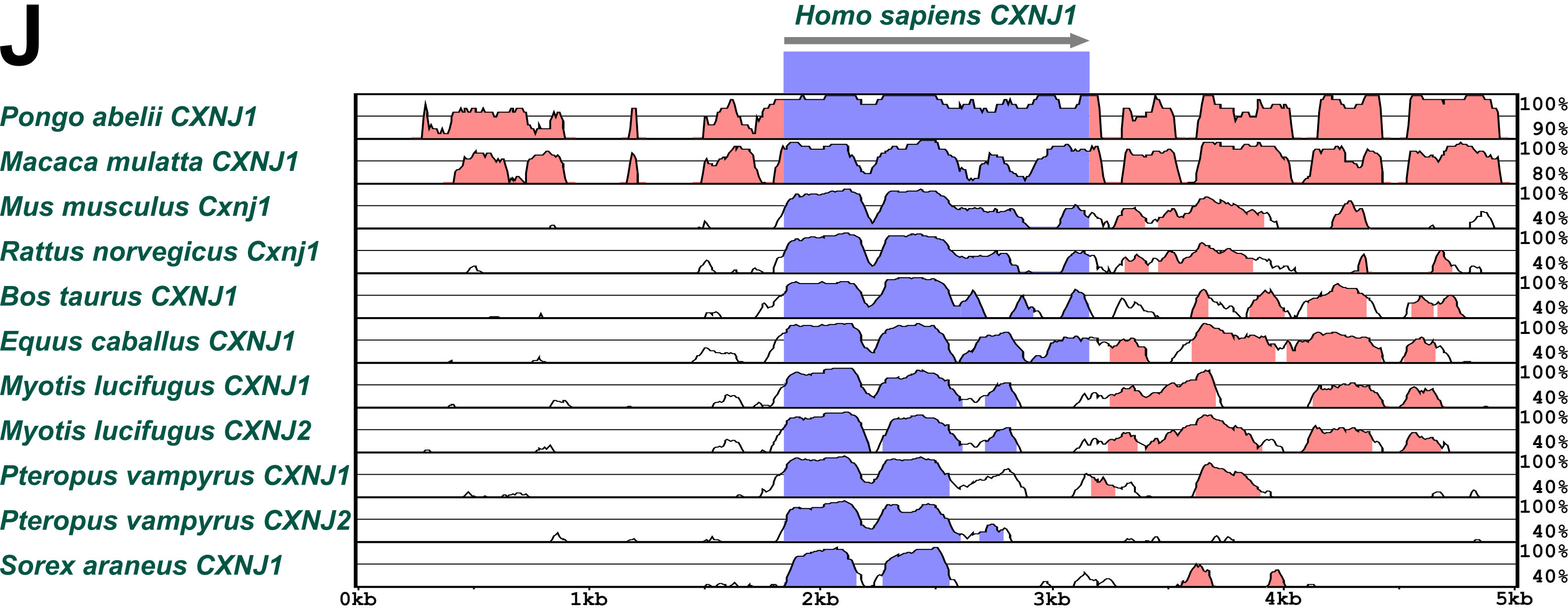
Homo sapiens CXNJ1
Pongo abelii CXNJ1
Macaca mulatta CXNJ1
Mus musculus Cxnj1
Rattus norvegicus Cxnj1
Bos taurus CXNJ1
Equus caballus CXNJ1
Myotis lucifugus CXNJ1
Myotis lucifugus CXNJ2
Pteropus vampyrus CXNJ1
Pteropus vampyrus CXNJ2
Sorex araneus CXNJ1
100%
90%
80%
40%
0kb
1kb
2kb
3kb
4kb
5kb

K

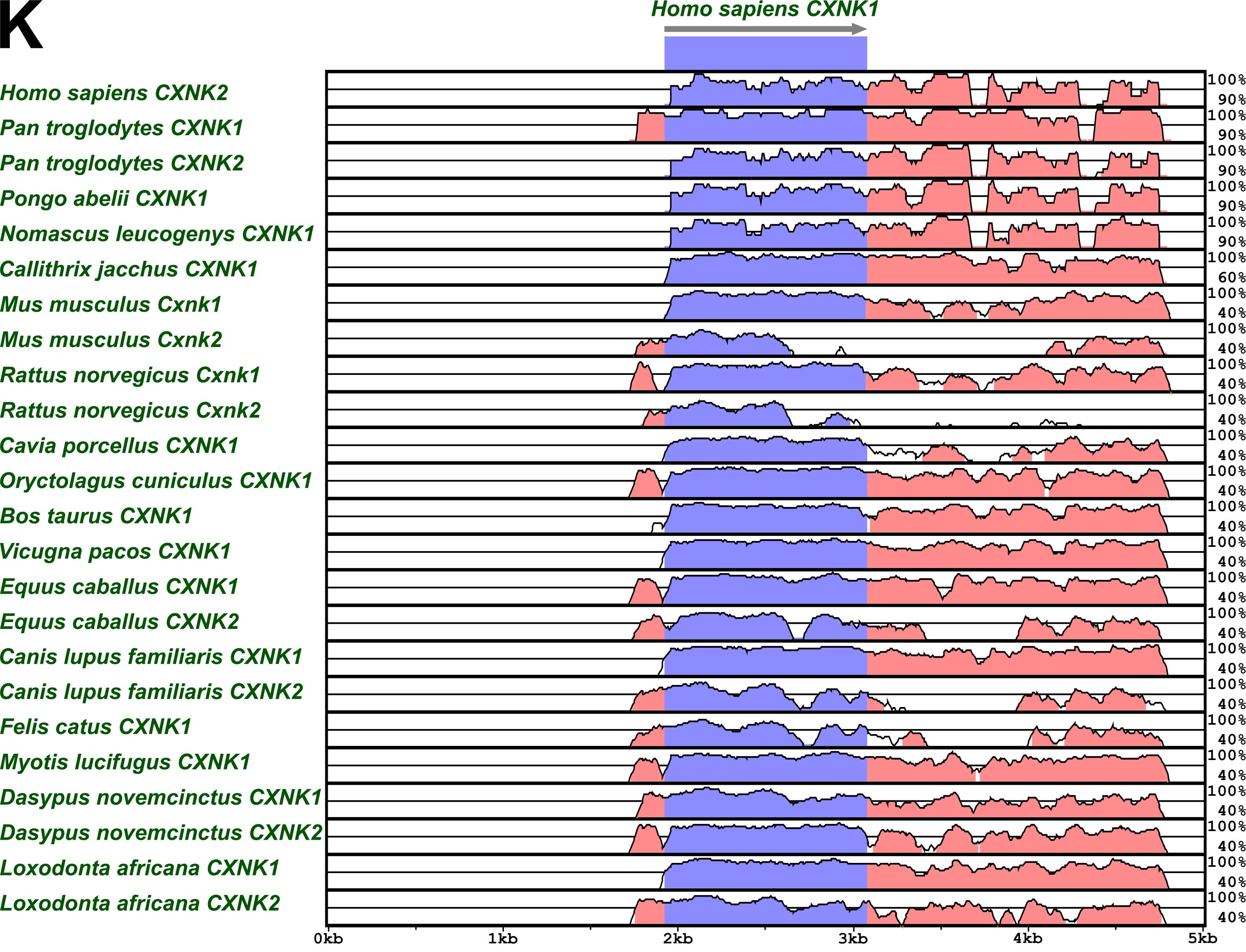
Homo sapiens CXNK1
Homo sapiens CXNK2
Pan troglodytes CXNK1
Pan troglodytes CXNK2
Pongo abelii CXNK1
Nomascus leucogenys CXNK1
Callithrix jacchus CXNK1
Mus musculus Cxnk1
Mus musculus Cxnk2
Rattus norvegicus Cxnk1
Rattus norvegicus Cxnk2
Cavia porcellus CXNK1
Oryctolagus cuniculus CXNK1
Bos taurus CXNK1
Vicugna pacos CXNK1
Equus caballus CXNK1
Equus caballus CXNK2
Canis lupus familiaris CXNK1
Canis lupus familiaris CXNK2
Felis catus CXNK1
Myotis lucifugus CXNK1
Dasypus novemcinctus CXNK1
Dasypus novemcinctus CXNK2
Loxodonta africana CXNK1
Loxodonta africana CXNK2
100%
90%
60%
40%
0kb
1kb
2kb
3kb
4kb
5kb

L

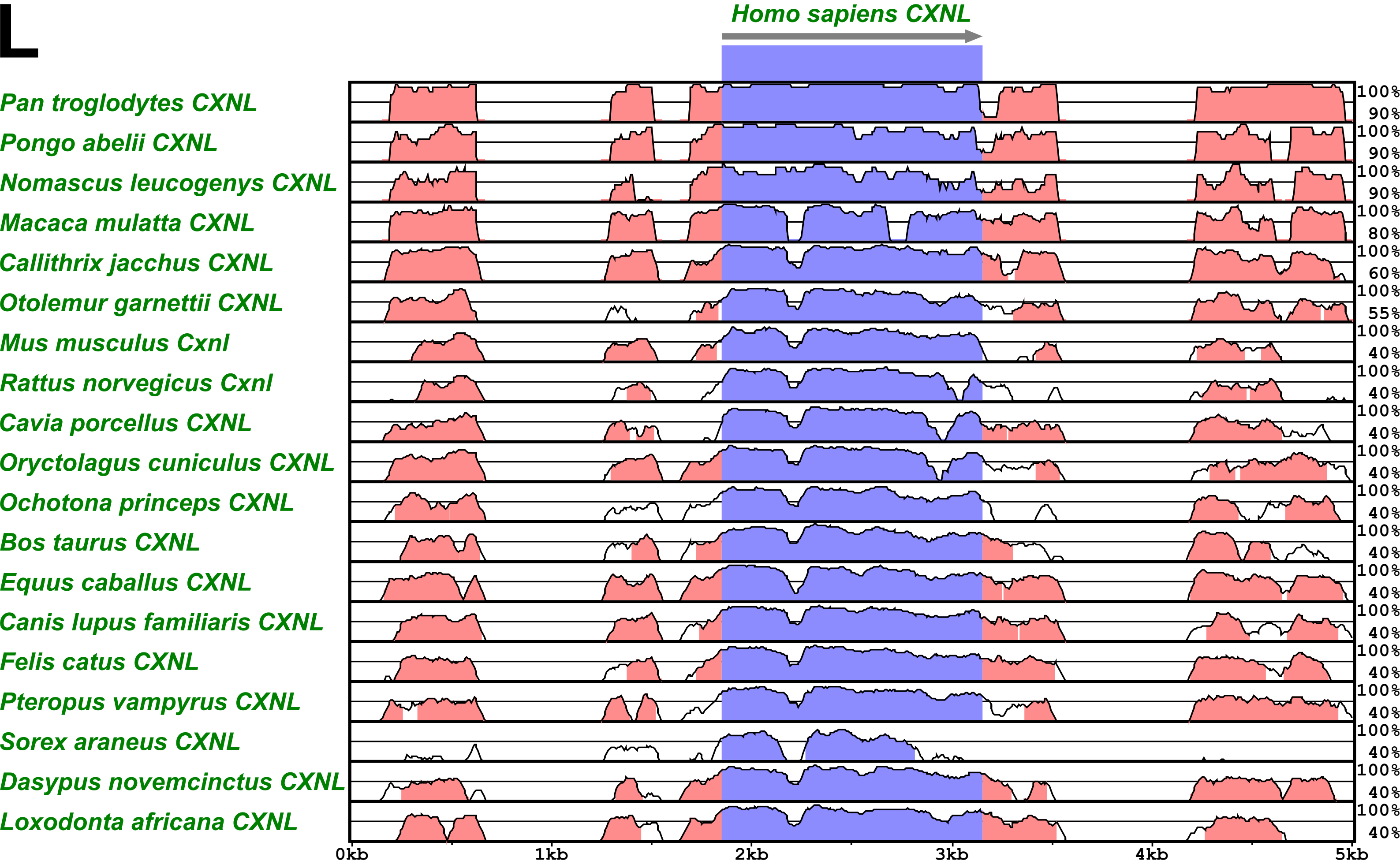
Homo sapiens CXNL
Pan troglodytes CXNL
Pongo abelii CXNL
Nomascus leucogenys CXNL
Macaca mulatta CXNL
Callithrix jacchus CXNL
Otolemur garnettii CXNL
Mus musculus Cxnl
Rattus norvegicus Cxnl
Cavia porcellus CXNL
Oryctolagus cuniculus CXNL
Ochotona princeps CXNL
Bos taurus CXNL
Equus caballus CXNL
Canis lupus familiaris CXNL
Felis catus CXNL
Pteropus vampyrus CXNL
Sorex araneus CXNL
Dasypus novemcinctus CXNL
Loxodonta africana CXNL
100%
90%
80%
60%
55%
40%
0kb
1kb
2kb
3kb
4kb
5kb

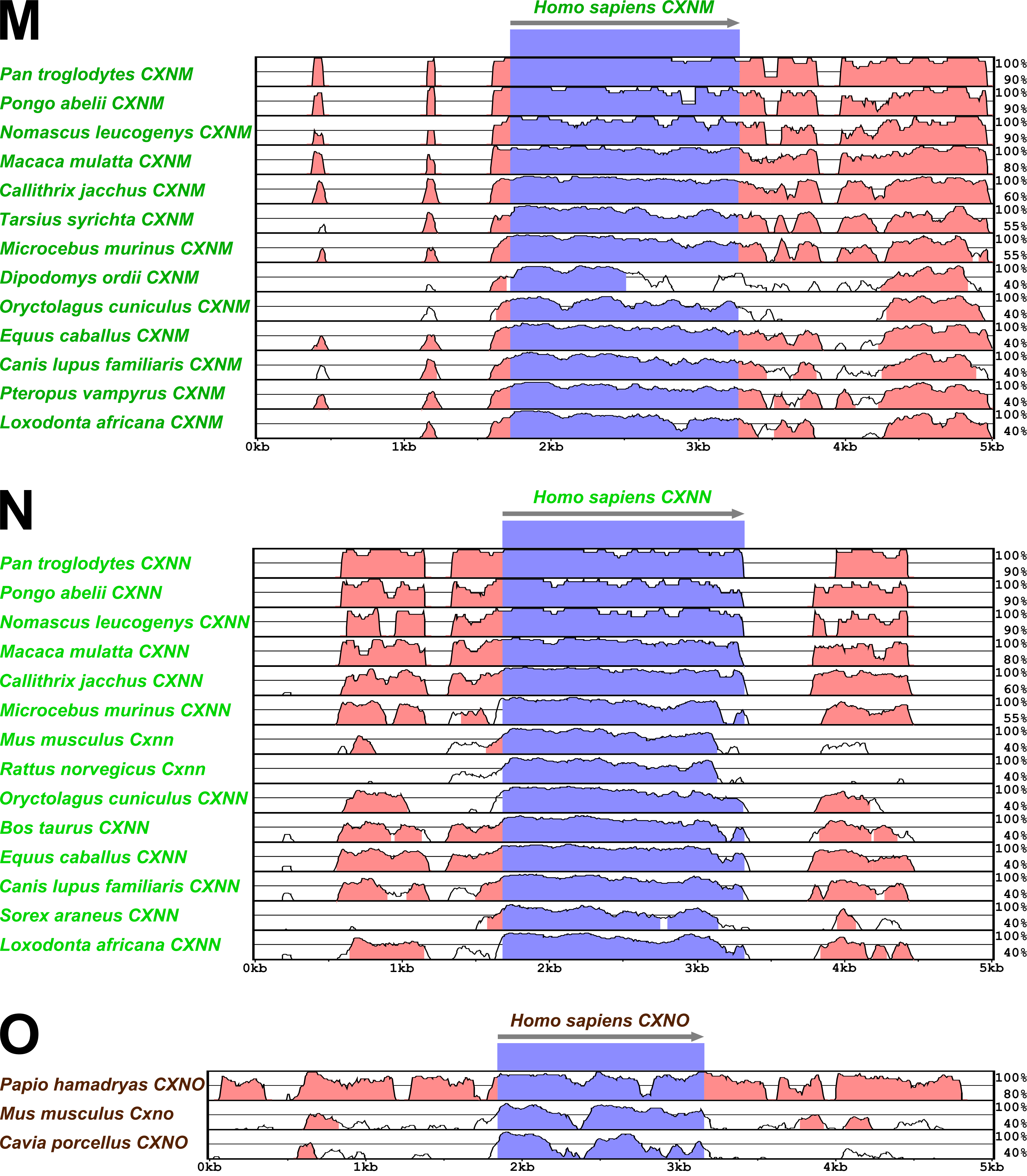

M
Homo sapiens CXNM
Pan troglodytes CXNM
Pongo abelii CXNM
Nomascus leucogenys CXNM
Macaca mulatta CXNM
Callithrix jacchus CXNM
Tarsius syrichta CXNM
Microcebus murinus CXNM
Dipodomys ordii CXNM
Oryctolagus cuniculus CXNM
Equus caballus CXNM
Canis lupus familiaris CXNM
Pteropus vampyrus CXNM
Loxodonta africana CXNM
100%
90%
80%
60%
55%
40%
0kb
1kb
2kb
3kb
4kb
5kb
N
Homo sapiens CXNN
Pan troglodytes CXNN
Pongo abelii CXNN
Nomascus leucogenys CXNN
Macaca mulatta CXNN
Callithrix jacchus CXNN
Microcebus murinus CXNN
Mus musculus Cxnn
Rattus norvegicus Cxnn
Oryctolagus cuniculus CXNN
Bos taurus CXNN
Equus caballus CXNN
Canis lupus familiaris CXNN
Sorex araneus CXNN
Loxodonta africana CXNN
0kb
1kb
2kb
3kb
4kb
5kb
O
Homo sapiens CXNO
Papio hamadryas CXNO
Mus musculus Cxno
Cavia porcellus CXNO
0kb
1kb
2kb
3kb
4kb
5kb

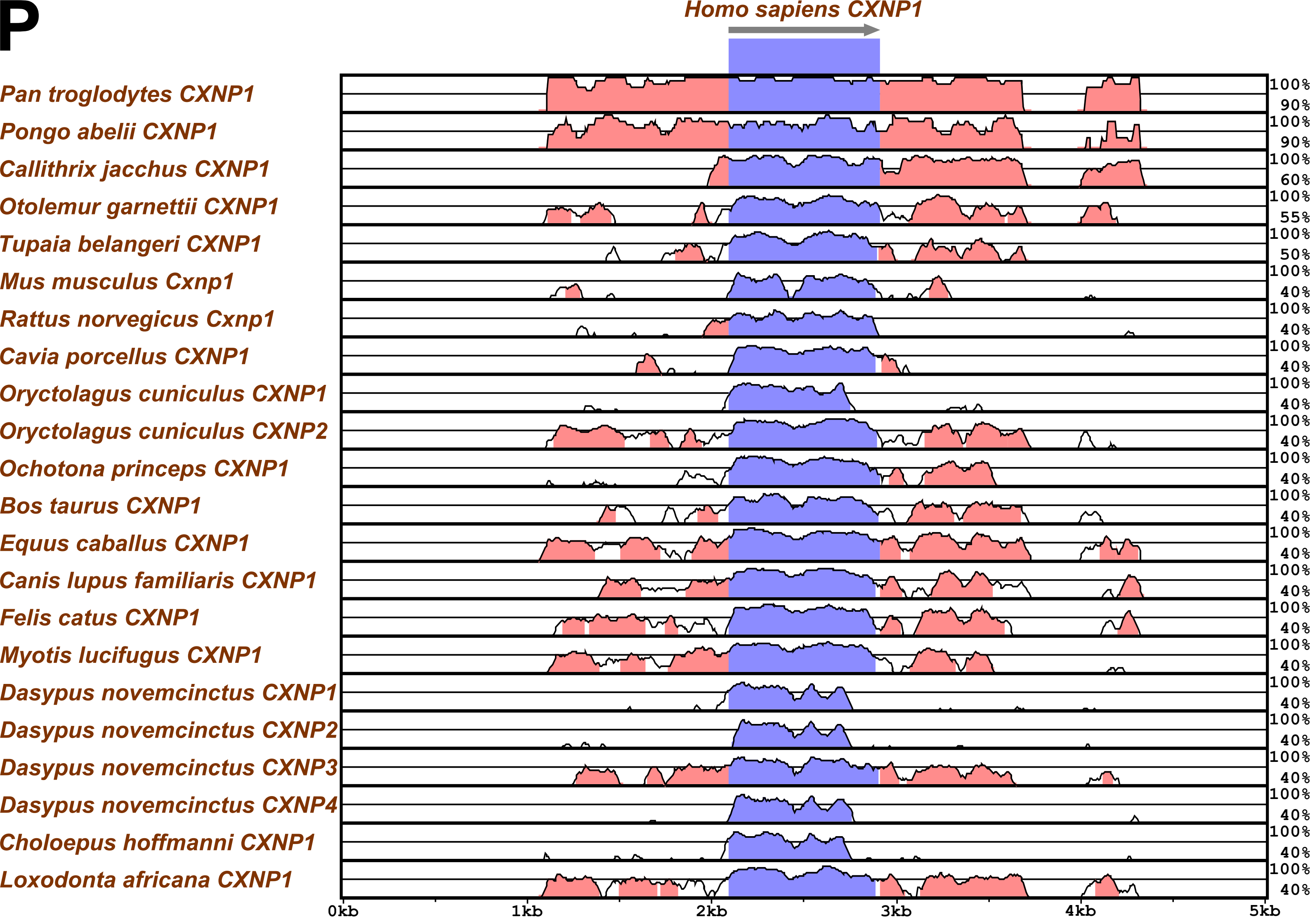
P
Homo sapiens CXNP1
Pan troglodytes CXNP1
Pongo abelii CXNP1
Callithrix jacchus CXNP1
Otolemur garnettii CXNP1
Tupaia belangeri CXNP1
Mus musculus Cxnp1
Rattus norvegicus Cxnp1
Cavia porcellus CXNP1
Oryctolagus cuniculus CXNP1
Oryctolagus cuniculus CXNP2
Ochotona princeps CXNP1
Bos taurus CXNP1
Equus caballus CXNP1
Canis lupus familiaris CXNP1
Felis catus CXNP1
Myotis lucifugus CXNP1
Dasypus novemcinctus CXNP1
Dasypus novemcinctus CXNP2
Dasypus novemcinctus CXNP3
Dasypus novemcinctus CXNP4
Choloepus hoffmanni CXNP1
Loxodonta africana CXNP1
0kb
1kb
2kb
3kb
4kb
5kb

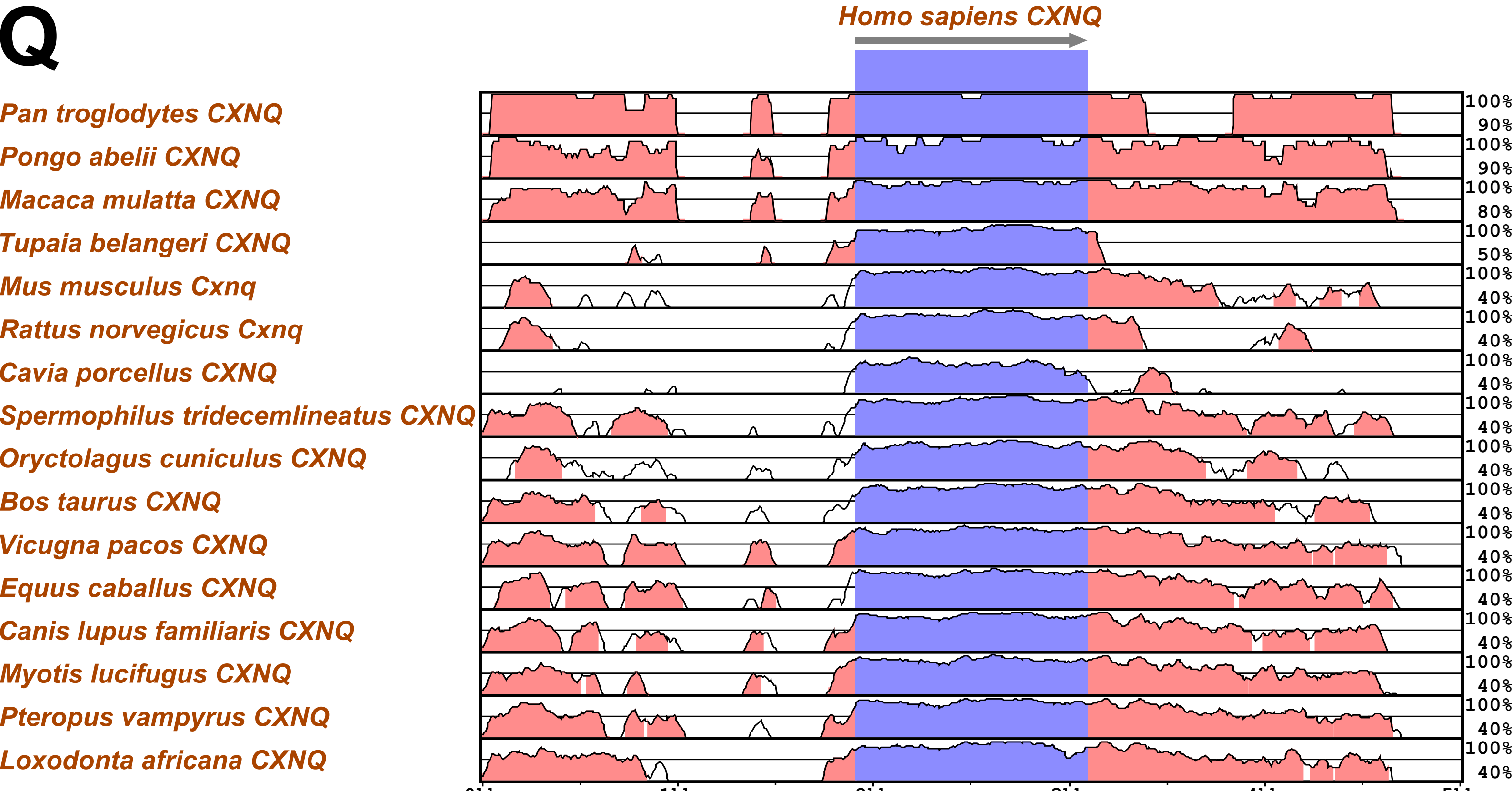
Q
Homo sapiens CXNQ
Pan troglodytes CXNQ
Pongo abelii CXNQ
Macaca mulatta CXNQ
Tupaia belangeri CXNQ
Mus musculus Cxnq
Rattus norvegicus Cxnq
Cavia porcellus CXNQ
Spermophilus tridecemlineatus CXNQ
Oryctolagus cuniculus CXNQ
Bos taurus CXNQ
Vicugna pacos CXNQ
Equus caballus CXNQ
Canis lupus familiaris CXNQ
Myotis lucifugus CXNQ
Pteropus vampyrus CXNQ
Loxodonta africana CXNQ
0kb
1kb
2kb
3kb
4kb
5kb

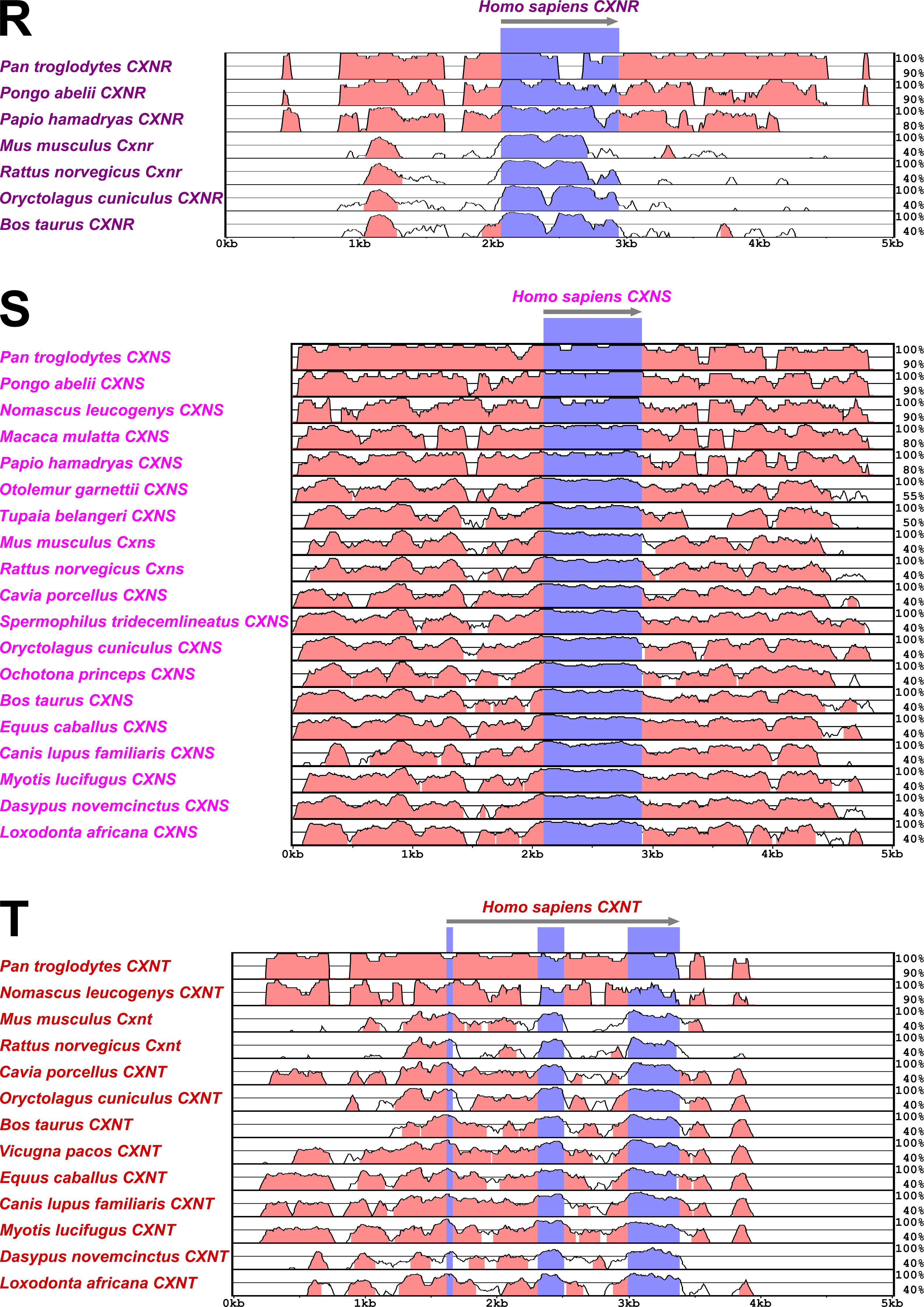
R
Homo sapiens CXNR
Pan troglodytes CXNR
Pongo abelii CXNR
Papio hamadryas CXNR
Mus musculus Cxnr
Rattus norvegicus Cxnr
Oryctolagus cuniculus CXNR
Bos taurus CXNR
100%
90%
80%
40%
0kb
1kb
2kb
3kb
4kb
5kb
S
Homo sapiens CXNS
Pan troglodytes CXNS
Pongo abelii CXNS
Nomascus leucogenys CXNS
Macaca mulatta CXNS
Papio hamadryas CXNS
Otolemur garnettii CXNS
Tupaia belangeri CXNS
Mus musculus Cxns
Rattus norvegicus Cxns
Cavia porcellus CXNS
Spermophilus tridecemlineatus CXNS
Oryctolagus cuniculus CXNS
Ochotona princeps CXNS
Bos taurus CXNS
Equus caballus CXNS
Canis lupus familiaris CXNS
Myotis lucifugus CXNS
Dasypus novemcinctus CXNS
Loxodonta africana CXNS
100%
90%
80%
55%
50%
40%
0kb
1kb
2kb
3kb
4kb
5kb
T
Homo sapiens CXNT
Pan troglodytes CXNT
Nomascus leucogenys CXNT
Mus musculus Cxnt
Rattus norvegicus Cxnt
Cavia porcellus CXNT
Oryctolagus cuniculus CXNT
Bos taurus CXNT
Vicugna pacos CXNT
Equus caballus CXNT
Canis lupus familiaris CXNT
Myotis lucifugus CXNT
Dasypus novemcinctus CXNT
Loxodonta africana CXNT
100%
90%
40%
0kb
1kb
2kb
3kb
4kb
5kb

U

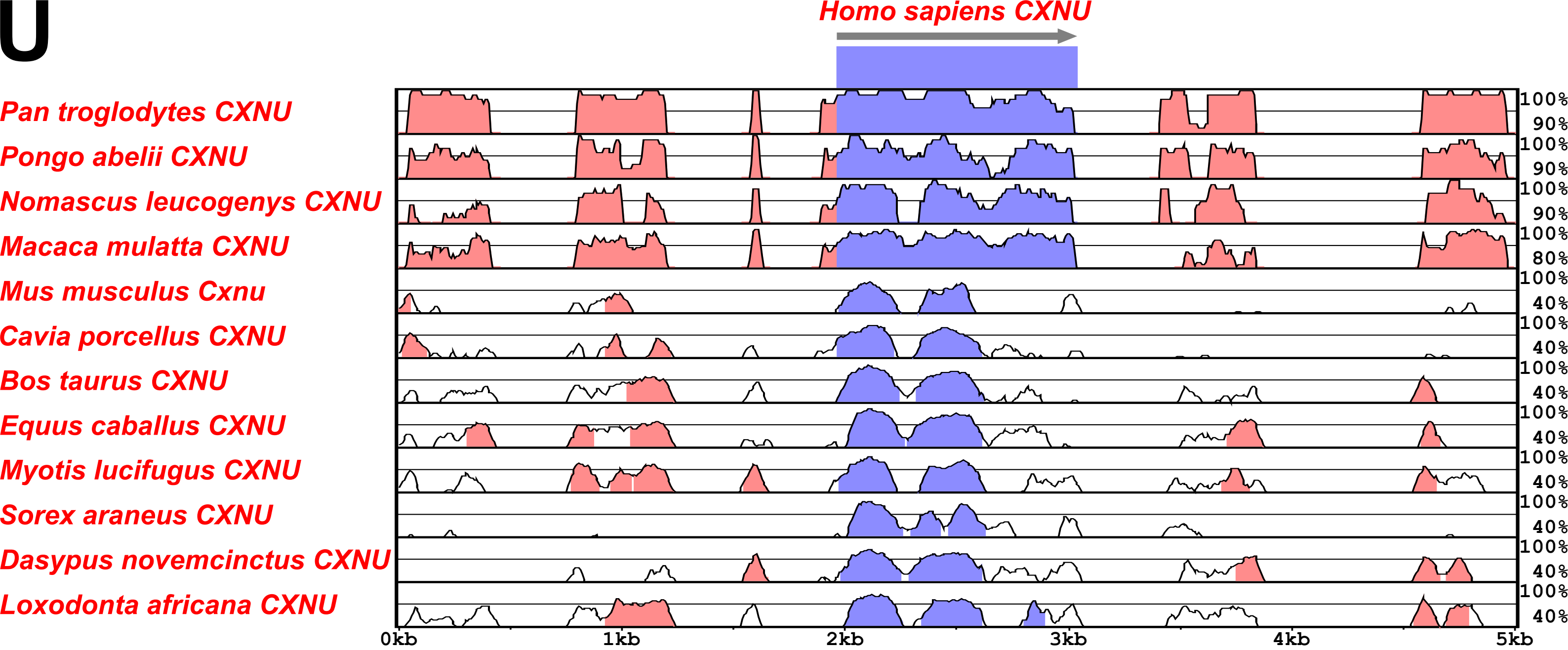

Homo sapiens CXNU
Pan troglodytes CXNU
Pongo abelii CXNU
Nomascus leucogenys CXNU
Macaca mulatta CXNU
Mus musculus Cxnu
Cavia porcellus CXNU
Bos taurus CXNU
Equus caballus CXNU
Myotis lucifugus CXNU
Sorex araneus CXNU
Dasypus novemcinctus CXNU
Loxodonta africana CXNU
100%
90%
80%
40%
0kb
1kb
2kb
3kb
4kb
5kb

Supplementary data file 3: Pairwise nucleotide sequence identity patterns of eutherian connexin genes.

	$\bar{a}$	a_{max}	a_{min}	$\bar{a}_{ad}$	$\bar{a}$	a_{max}	a_{min}	$\bar{a}_{ad}$	$\bar{a}$	a_{max}	a_{min}	$\bar{a}_{ad}$	$\bar{a}$	a_{max}	a_{min}	$\bar{a}_{ad}$	$\bar{a}$	a_{max}	a_{min}	$\bar{a}_{ad}$
Cluster	*CXNA*				*CXNB*				*CXNC*				*CXND*				*CXNE*			
CXNA	**0,828**	**0,992**	**0,656**	**0,058**																
CXNB	0,611	0,671	0,321	0,046	**0,806**	**0,987**	**0,455**	**0,081**												
CXNC	0,571	0,622	0,416	0,03	0,599	0,657	0,27	0,048	**0,834**	**0,991**	**0,575**	**0,059**								
CXND	0,492	0,542	0,277	0,035	0,475	0,526	0,256	0,047	0,471	0,526	0,219	0,037	**0,77**	**0,995**	**0,449**	**0,08**				
CXNE	0,439	0,47	0,364	0,017	0,454	0,504	0,223	0,033	0,459	0,511	0,319	0,024	0,432	0,527	0,228	0,034	**0,846**	**0,992**	**0,688**	**0,058**
CXNF	0,475	0,536	0,4	0,015	0,475	0,527	0,255	0,03	0,489	0,545	0,342	0,021	0,441	0,478	0,276	0,024	0,63	0,673	0,535	0,021
CXNG	0,503	0,549	0,418	0,016	0,485	0,522	0,268	0,028	0,493	0,529	0,361	0,018	0,421	0,457	0,265	0,024	0,51	0,542	0,43	0,016
CXNH	0,334	0,36	0,292	0,011	0,331	0,369	0,17	0,02	0,332	0,361	0,258	0,012	0,293	0,315	0,166	0,017	0,33	0,353	0,295	0,011
CXNI	0,317	0,349	0,279	0,009	0,311	0,353	0,152	0,02	0,318	0,357	0,244	0,013	0,279	0,304	0,153	0,017	0,327	0,353	0,283	0,011
CXNJ	0,301	0,403	0,195	0,041	0,302	0,423	0,105	0,048	0,317	0,43	0,202	0,045	0,28	0,425	0,118	0,049	0,326	0,485	0,202	0,06
CXNK	0,317	0,378	0,27	0,018	0,304	0,381	0,163	0,025	0,318	0,38	0,239	0,019	0,308	0,435	0,18	0,027	0,33	0,478	0,276	0,032
CXNL	0,289	0,331	0,253	0,01	0,288	0,334	0,146	0,018	0,292	0,331	0,232	0,011	0,255	0,308	0,145	0,017	0,288	0,323	0,25	0,009
CXNM	0,212	0,266	0,189	0,009	0,198	0,251	0,091	0,013	0,199	0,246	0,164	0,007	0,206	0,264	0,116	0,013	0,229	0,288	0,2	0,009
CXNN	0,211	0,23	0,182	0,01	0,2	0,226	0,094	0,016	0,205	0,229	0,165	0,012	0,205	0,233	0,115	0,014	0,223	0,247	0,192	0,01
CXNO	0,254	0,277	0,231	0,008	0,249	0,267	0,143	0,013	0,254	0,276	0,213	0,01	0,228	0,246	0,146	0,011	0,249	0,272	0,226	0,008
CXNP	0,268	0,318	0,175	0,018	0,272	0,32	0,037	0,027	0,272	0,321	0,158	0,02	0,258	0,341	0,05	0,028	0,315	0,367	0,208	0,023
CXNQ	0,265	0,303	0,235	0,008	0,253	0,302	0,128	0,016	0,258	0,296	0,204	0,009	0,256	0,283	0,151	0,014	0,26	0,279	0,229	0,007
CXNR	0,294	0,326	0,246	0,009	0,307	0,345	0,151	0,019	0,307	0,333	0,234	0,013	0,28	0,303	0,159	0,015	0,291	0,319	0,25	0,012
CXNS	0,217	0,263	0,185	0,007	0,223	0,275	0,127	0,011	0,233	0,283	0,177	0,009	0,221	0,288	0,136	0,01	0,239	0,292	0,204	0,01
CXNT	0,238	0,266	0,2	0,01	0,235	0,276	0,111	0,018	0,25	0,286	0,167	0,014	0,261	0,314	0,134	0,021	0,293	0,328	0,235	0,013
CXNU	0,221	0,246	0,178	0,011	0,222	0,251	0,12	0,015	0,231	0,259	0,163	0,014	0,202	0,232	0,123	0,014	0,211	0,231	0,172	0,011

Cluster	CXNF				CXNG				CXNH				CXNI				CXNJ			
CXNF	**0,875**	**0,993**	**0,783**	**0,029**																
CXNG	0,554	0,59	0,52	0,011	**0,933**	**0,996**	**0,888**	**0,016**												
CXNH	0,336	0,373	0,302	0,009	0,343	0,361	0,311	0,008	**0,85**	**0,994**	**0,613**	**0,066**								
CXNI	0,33	0,376	0,302	0,008	0,323	0,343	0,292	0,006	0,399	0,443	0,317	0,016	**0,854**	**0,995**	**0,703**	**0,043**				
CXNJ	0,311	0,446	0,198	0,05	0,328	0,43	0,215	0,041	0,362	0,464	0,222	0,035	0,421	0,503	0,302	0,032	**0,573**	**0,987**	**0,357**	**0,131**
CXNK	0,33	0,414	0,296	0,023	0,338	0,397	0,308	0,02	0,391	0,456	0,314	0,014	0,435	0,511	0,314	0,037	0,381	0,52	0,223	0,044
CXNL	0,288	0,33	0,255	0,009	0,311	0,346	0,275	0,007	0,373	0,407	0,305	0,013	0,435	0,485	0,372	0,012	0,417	0,496	0,298	0,048
CXNM	0,219	0,279	0,202	0,008	0,22	0,271	0,202	0,007	0,261	0,286	0,225	0,007	0,296	0,307	0,276	0,004	0,278	0,32	0,205	0,024
CXNN	0,214	0,236	0,194	0,01	0,216	0,237	0,192	0,012	0,272	0,3	0,205	0,014	0,286	0,318	0,252	0,014	0,273	0,323	0,204	0,023
CXNO	0,244	0,275	0,229	0,007	0,27	0,285	0,253	0,007	0,317	0,339	0,266	0,01	0,332	0,358	0,309	0,008	0,308	0,347	0,244	0,022
CXNP	0,28	0,331	0,205	0,017	0,276	0,322	0,206	0,016	0,278	0,316	0,182	0,017	0,266	0,302	0,173	0,015	0,254	0,372	0,12	0,04
CXNQ	0,267	0,296	0,246	0,006	0,263	0,293	0,247	0,005	0,318	0,354	0,266	0,01	0,328	0,363	0,3	0,006	0,29	0,343	0,214	0,019
CXNR	0,289	0,32	0,267	0,009	0,323	0,344	0,307	0,006	0,277	0,295	0,246	0,009	0,294	0,315	0,263	0,009	0,277	0,355	0,191	0,026
CXNS	0,226	0,277	0,212	0,006	0,234	0,278	0,217	0,005	0,234	0,285	0,205	0,008	0,227	0,271	0,205	0,007	0,208	0,323	0,141	0,031
CXNT	0,267	0,287	0,241	0,007	0,243	0,268	0,224	0,007	0,207	0,233	0,182	0,008	0,202	0,222	0,179	0,007	0,193	0,29	0,119	0,036
CXNU	0,22	0,247	0,19	0,01	0,223	0,241	0,2	0,007	0,24	0,275	0,194	0,014	0,257	0,289	0,221	0,01	0,226	0,287	0,163	0,019

Cluster	*CXNK*				*CXNL*				*CXNM*				*CXNN*				*CXNO*			
CXNK	**0,758**	**0,993**	**0,493**	**0,152**																
CXNL	0,376	0,439	0,281	0,03	**0,835**	**0,996**	**0,589**	**0,049**												
CXNM	0,285	0,328	0,228	0,019	0,298	0,316	0,254	0,009	**0,838**	**0,998**	**0,577**	**0,072**								
CXNN	0,276	0,311	0,217	0,017	0,301	0,331	0,249	0,014	0,408	0,436	0,348	0,014	**0,798**	**0,993**	**0,664**	**0,062**				
CXNO	0,314	0,368	0,257	0,022	0,329	0,36	0,296	0,012	0,238	0,255	0,218	0,008	0,243	0,262	0,222	0,009	**0,766**	**0,958**	**0,683**	**0,077**
CXNP	0,256	0,378	0,173	0,02	0,234	0,275	0,151	0,014	0,202	0,229	0,118	0,011	0,194	0,224	0,11	0,014	0,261	0,298	0,17	0,016
CXNQ	0,316	0,357	0,266	0,016	0,315	0,345	0,28	0,007	0,258	0,267	0,239	0,004	0,264	0,286	0,242	0,011	0,469	0,529	0,429	0,018
CXNR	0,266	0,297	0,24	0,01	0,255	0,308	0,231	0,009	0,194	0,214	0,183	0,005	0,19	0,208	0,171	0,009	0,305	0,325	0,275	0,011
CXNS	0,21	0,295	0,193	0,01	0,201	0,246	0,18	0,006	0,152	0,207	0,14	0,006	0,151	0,197	0,134	0,009	0,242	0,282	0,232	0,006
CXNT	0,213	0,309	0,177	0,023	0,171	0,199	0,146	0,006	0,147	0,181	0,134	0,005	0,146	0,162	0,13	0,006	0,164	0,183	0,147	0,006
CXNU	0,234	0,272	0,194	0,013	0,24	0,271	0,201	0,012	0,187	0,213	0,157	0,01	0,188	0,216	0,16	0,011	0,267	0,306	0,231	0,015

Cluster	*CXNP*				*CXNQ*				*CXNR*				*CXNS*				*CXNT*			
CXNP	**0,669**	**0,996**	**0,339**	**0,087**																
CXNQ	0,286	0,324	0,183	0,016	**0,908**	**0,999**	**0,785**	**0,025**												
CXNR	0,261	0,303	0,191	0,016	0,304	0,338	0,274	0,01	**0,852**	**0,993**	**0,748**	**0,061**								
CXNS	0,226	0,295	0,118	0,016	0,261	0,317	0,243	0,007	0,245	0,303	0,224	0,009	**0,888**	**0,996**	**0,598**	**0,071**				
CXNT	0,245	0,291	0,175	0,017	0,198	0,21	0,182	0,004	0,223	0,251	0,202	0,01	0,175	0,224	0,161	0,007	**0,855**	**0,991**	**0,779**	**0,032**
CXNU	0,194	0,23	0,111	0,013	0,271	0,309	0,23	0,015	0,286	0,327	0,236	0,018	0,221	0,273	0,179	0,014	0,19	0,212	0,165	0,009

Cluster	*CXNU*			
CXNU	**0,618**	**0,985**	**0,44**	**0,09**

$\bar{a}$, average pairwise identity; $\bar{a}_{ad}$, average absolute deviation for $\bar{a}$; a_{max}, largest pairwise identity; a_{min}, smallest pairwise identity; bold, calculation within major cluster.

Supplementary data file 4: Protein amino acid sequence alignments of eutherian connexins. Using white letters on black background (≥90% sequence identity level), white letters on dark grey background (≥75% sequence identity level) or black letters on grey background (≥50% sequence identity level), the amino acid positions were labelled. In reference human CXNA protein amino acid sequence, the 15 invariant amino acid sites were shown using white letters on violet backgrounds and 2 forward amino acid sites were shown using white letters on red backgrounds (top). The stop codons were indicated by &s.

```
Homo sapiens CXNA                - -----------------------------------------------------------------  -
                                           10        20        30        40        50        60
Homo sapiens CXNA                - -----------------------------------------------------------------  -
Pan troglodytes CXNA             - -----------------------------------------------------------------  -
Gorilla gorilla CXNA             - -----------------------------------------------------------------  -
Pongo abelii CXNA                - -----------------------------------------------------------------  -
Nomascus leucogenys CXNA         - -----------------------------------------------------------------  -
Macaca mulatta CXNA              - -----------------------------------------------------------------  -
Callithrix jacchus CXNA          - -----------------------------------------------------------------  -
Mus musculus Cxna                - -----------------------------------------------------------------  -
Rattus norvegicus Cxna           - -----------------------------------------------------------------  -
Cavia porcellus CXNA             - -----------------------------------------------------------------  -
Ochotona princeps CXNA           - -----------------------------------------------------------------  -
Bos taurus CXNA                  - -----------------------------------------------------------------  -
Equus caballus CXNA              - -----------------------------------------------------------------  -
Canis lupus familiaris CXNA      - -----------------------------------------------------------------  -
Felis catus CXNA                 - -----------------------------------------------------------------  -
Myotis lucifugus CXNA            - -----------------------------------------------------------------  -
Dasypus novemcinctus CXNA        - -----------------------------------------------------------------  -
Loxodonta africana CXNA          - -----------------------------------------------------------------  -
Homo sapiens CXNB                - -----------------------------------------------------------------  -
Gorilla gorilla CXNB             - -----------------------------------------------------------------  -
Nomascus leucogenys CXNB         - -----------------------------------------------------------------  -
Macaca mulatta CXNB              - -----------------------------------------------------------------  -
Callithrix jacchus CXNB          - -----------------------------------------------------------------  -
Otolemur garnettii CXNB          - -----------------------------------------------------------------  -
Mus musculus Cxnb                - -----------------------------------------------------------------  -
Rattus norvegicus Cxnb           - -----------------------------------------------------------------  -
Dipodomys ordii CXNB             - -----------------------------------------------------------------  -
Cavia porcellus CXNB             - -----------------------------------------------------------------  -
Oryctolagus cuniculus CXNB       - -----------------------------------------------------------------  -
Ochotona princeps CXNB           - -----------------------------------------------------------------  -
Bos taurus CXNB                  - -----------------------------------------------------------------  -
Equus caballus CXNB              - -----------------------------------------------------------------  -
Canis lupus familiaris CXNB      - -----------------------------------------------------------------  -
Myotis lucifugus CXNB            - -----------------------------------------------------------------  -
Erinaceus europaeus CXNB         - -----------------------------------------------------------------  -
Loxodonta africana CXNB          - -----------------------------------------------------------------  -
Homo sapiens CXNC                - -----------------------------------------------------------------  -
Gorilla gorilla CXNC             - -----------------------------------------------------------------  -
Pongo abelii CXNC                - -----------------------------------------------------------------  -
Nomascus leucogenys CXNC         - -----------------------------------------------------------------  -
Callithrix jacchus CXNC          - -----------------------------------------------------------------  -
Microcebus murinus CXNC          - -----------------------------------------------------------------  -
Otolemur garnettii CXNC          - -----------------------------------------------------------------  -
Mus musculus Cxnc                - -----------------------------------------------------------------  -
Rattus norvegicus Cxnc           - -----------------------------------------------------------------  -
Cavia porcellus CXNC             - -----------------------------------------------------------------  -
Oryctolagus cuniculus CXNC       1 -------------------------------------------------------------MAAT  4
Bos taurus CXNC                  - -----------------------------------------------------------------  -
Equus caballus CXNC              - -----------------------------------------------------------------  -
Canis lupus familiaris CXNC      - -----------------------------------------------------------------  -
Felis catus CXNC                 - -----------------------------------------------------------------  -
Myotis lucifugus CXNC            - -----------------------------------------------------------------  -
Dasypus novemcinctus CXNC        - -----------------------------------------------------------------  -
Loxodonta africana CXNC          - -----------------------------------------------------------------  -
Homo sapiens CXND                - -----------------------------------------------------------------  -
Pan troglodytes CXND             - -----------------------------------------------------------------  -
Macaca mulatta CXND              - -----------------------------------------------------------------  -
Callithrix jacchus CXND          - -----------------------------------------------------------------  -
Dipodomys ordii CXND             - -----------------------------------------------------------------  -
Oryctolagus cuniculus CXND       - -----------------------------------------------------------------  -
Tursiops truncatus CXND          - -----------------------------------------------------------------  -
Bos taurus CXND                  - -----------------------------------------------------------------  -
Equus caballus CXND              - -----------------------------------------------------------------  -
Canis lupus familiaris CXND      - -----------------------------------------------------------------  -
Myotis lucifugus CXND            - -----------------------------------------------------------------  -
Erinaceus europaeus CXND         - -----------------------------------------------------------------  -
Dasypus novemcinctus CXND        - -----------------------------------------------------------------  -
Choloepus hoffmanni CXND         - -----------------------------------------------------------------  -
Loxodonta africana CXND          - -----------------------------------------------------------------  -
Homo sapiens CXNE                - -----------------------------------------------------------------  -
Pan troglodytes CXNE             - -----------------------------------------------------------------  -
Pongo abelii CXNE                - -----------------------------------------------------------------  -
Macaca mulatta CXNE              - -----------------------------------------------------------------  -
Papio hamadryas CXNE             - -----------------------------------------------------------------  -
Callithrix jacchus CXNE          - -----------------------------------------------------------------  -
Otolemur garnettii CXNE          - -----------------------------------------------------------------  -
Mus musculus Cxne                - -----------------------------------------------------------------  -
```

```
Rattus norvegicus Cxne          - ----------------------------------------------------------------- -
Dipodomys ordii CXNE            - ----------------------------------------------------------------- -
Cavia porcellus CXNE            - ----------------------------------------------------------------- -
Oryctolagus cuniculus CXNE      - ----------------------------------------------------------------- -
Equus caballus CXNE             - ----------------------------------------------------------------- -
Canis lupus familiaris CXNE     - ----------------------------------------------------------------- -
Felis catus CXNE                - ----------------------------------------------------------------- -
Myotis lucifugus CXNE           - ----------------------------------------------------------------- -
Sorex araneus CXNE              - ----------------------------------------------------------------- -
Dasypus novemcinctus CXNE       - ----------------------------------------------------------------- -
Loxodonta africana CXNE         - ----------------------------------------------------------------- -
Homo sapiens CXNF               - ----------------------------------------------------------------- -
Pan troglodytes CXNF            - ----------------------------------------------------------------- -
Gorilla gorilla CXNF            - ----------------------------------------------------------------- -
Nomascus leucogenys CXNF        - ----------------------------------------------------------------- -
Callithrix jacchus CXNF         - ----------------------------------------------------------------- -
Mus musculus Cxnf               - ----------------------------------------------------------------- -
Rattus norvegicus Cxnf          - ----------------------------------------------------------------- -
S.tridecemlineatus CXNF         - ----------------------------------------------------------------- -
Oryctolagus cuniculus CXNF      - ----------------------------------------------------------------- -
Ochotona princeps CXNF          - ----------------------------------------------------------------- -
Bos taurus CXNF                 - ----------------------------------------------------------------- -
Equus caballus CXNF             - ----------------------------------------------------------------- -
Canis lupus familiaris CXNF     - ----------------------------------------------------------------- -
Myotis lucifugus CXNF           - ----------------------------------------------------------------- -
Dasypus novemcinctus CXNF       - ----------------------------------------------------------------- -
Choloepus hoffmanni CXNF        - ----------------------------------------------------------------- -
Loxodonta africana CXNF         - ----------------------------------------------------------------- -
Homo sapiens CXNG               - ----------------------------------------------------------------- -
Pan troglodytes CXNG            - ----------------------------------------------------------------- -
Gorilla gorilla CXNG            - ----------------------------------------------------------------- -
Pongo abelii CXNG               - ----------------------------------------------------------------- -
Nomascus leucogenys CXNG        - ----------------------------------------------------------------- -
Macaca mulatta CXNG             - ----------------------------------------------------------------- -
Papio hamadryas CXNG            - ----------------------------------------------------------------- -
Callithrix jacchus CXNG         - ----------------------------------------------------------------- -
Microcebus murinus CXNG         - ----------------------------------------------------------------- -
Mus musculus Cxng               - ----------------------------------------------------------------- -
Rattus norvegicus Cxng          - ----------------------------------------------------------------- -
Oryctolagus cuniculus CXNG      - ----------------------------------------------------------------- -
Bos taurus CXNG                 - ----------------------------------------------------------------- -
Equus caballus CXNG             - ----------------------------------------------------------------- -
Canis lupus familiaris CXNG     - ----------------------------------------------------------------- -
Felis catus CXNG                - ----------------------------------------------------------------- -
Myotis lucifugus CXNG           - ----------------------------------------------------------------- -
Pteropus vampyrus CXNG          - ----------------------------------------------------------------- -
Dasypus novemcinctus CXNG       - ----------------------------------------------------------------- -
Loxodonta africana CXNG         - ----------------------------------------------------------------- -
Procavia capensis CXNG          - ----------------------------------------------------------------- -
Homo sapiens CXNH1              - ----------------------------------------------------------------- -
Pan troglodytes CXNH1           - ----------------------------------------------------------------- -
Gorilla gorilla CXNH1           - ----------------------------------------------------------------- -
Pongo abelii CXNH1              - ----------------------------------------------------------------- -
Nomascus leucogenys CXNH1       - ----------------------------------------------------------------- -
Macaca mulatta CXNH1            - ----------------------------------------------------------------- -
Otolemur garnettii CXNH1        - ----------------------------------------------------------------- -
Mus musculus Cxnh1              - ----------------------------------------------------------------- -
Rattus norvegicus Cxnh1         - ----------------------------------------------------------------- -
Cavia porcellus CXNH1           - ----------------------------------------------------------------- -
Oryctolagus cuniculus CXNH1     - ----------------------------------------------------------------- -
Ochotona princeps CXNH1         - ----------------------------------------------------------------- -
Equus caballus CXNH1            - ----------------------------------------------------------------- -
Canis lupus familiaris CXNH1    - ----------------------------------------------------------------- -
Felis catus CXNH1               - ----------------------------------------------------------------- -
Myotis lucifugus CXNH1          - ----------------------------------------------------------------- -
Dasypus novemcinctus CXNH1      - ----------------------------------------------------------------- -
Loxodonta africana CXNH1        - ----------------------------------------------------------------- -
Homo sapiens CXNI               - ----------------------------------------------------------------- -
Pan troglodytes CXNI            - ----------------------------------------------------------------- -
Pongo abelii CXNI               - ----------------------------------------------------------------- -
Nomascus leucogenys CXNI        - ----------------------------------------------------------------- -
Macaca mulatta CXNI             - ----------------------------------------------------------------- -
Papio hamadryas CXNI            - ----------------------------------------------------------------- -
Callithrix jacchus CXNI         - ----------------------------------------------------------------- -
Otolemur garnettii CXNI         - ----------------------------------------------------------------- -
Mus musculus Cxni               - ----------------------------------------------------------------- -
Rattus norvegicus Cxni          - ----------------------------------------------------------------- -
Cavia porcellus CXNI            - ----------------------------------------------------------------- -
Oryctolagus cuniculus CXNI      - ----------------------------------------------------------------- -
```

```
Bos taurus CXNI                  - -----------------------------------------------------------------  -
Equus caballus CXNI              - -----------------------------------------------------------------  -
Canis lupus familiaris CXNI      - -----------------------------------------------------------------  -
Felis catus CXNI                 - -----------------------------------------------------------------  -
Pteropus vampyrus CXNI           - -----------------------------------------------------------------  -
Erinaceus europaeus CXNI         - -----------------------------------------------------------------  -
Dasypus novemcinctus CXNI        - -----------------------------------------------------------------  -
Loxodonta africana CXNI          - -----------------------------------------------------------------  -
Homo sapiens CXNJ1               - -----------------------------------------------------------------  -
Pongo abelii CXNJ1               - -----------------------------------------------------------------  -
Macaca mulatta CXNJ1             - -----------------------------------------------------------------  -
Mus musculus Cxnj1               - -----------------------------------------------------------------  -
Rattus norvegicus Cxnj1          - -----------------------------------------------------------------  -
Bos taurus CXNJ1                 - -----------------------------------------------------------------  -
Equus caballus CXNJ1             - -----------------------------------------------------------------  -
Myotis lucifugus CXNJ1           - -----------------------------------------------------------------  -
Myotis lucifugus CXNJ2           - -----------------------------------------------------------------  -
Pteropus vampyrus CXNJ1          1 MYICTCIHMRVCMPICTMHMCACKAHVCTHACVYMYTQMRARSLLVCMSTHVIHTCVCTVNMHPY  65
Pteropus vampyrus CXNJ2          - -----------------------------------------------------------------  -
Sorex araneus CXNJ1              - -----------------------------------------------------------------  -
Homo sapiens CXNK1               - -----------------------------------------------------------------  -
Homo sapiens CXNK2               - -----------------------------------------------------------------  -
Pan troglodytes CXNK1            - -----------------------------------------------------------------  -
Pan troglodytes CXNK2            - -----------------------------------------------------------------  -
Pongo abelii CXNK1               - -----------------------------------------------------------------  -
Nomascus leucogenys CXNK1        - -----------------------------------------------------------------  -
Callithrix jacchus CXNK1         - -----------------------------------------------------------------  -
Mus musculus Cxnk1               - -----------------------------------------------------------------  -
Mus musculus Cxnk2               - -----------------------------------------------------------------  -
Rattus norvegicus Cxnk1          - -----------------------------------------------------------------  -
Rattus norvegicus Cxnk2          - -----------------------------------------------------------------  -
Cavia porcellus CXNK1            - -----------------------------------------------------------------  -
Oryctolagus cuniculus CXNK1      - -----------------------------------------------------------------  -
Bos taurus CXNK1                 - -----------------------------------------------------------------  -
Vicugna pacos CXNK1              - -----------------------------------------------------------------  -
Equus caballus CXNK1             - -----------------------------------------------------------------  -
Equus caballus CXNK2             - -----------------------------------------------------------------  -
Canis lupus familiaris CXNK1     - -----------------------------------------------------------------  -
Canis lupus familiaris CXNK2     - -----------------------------------------------------------------  -
Felis catus CXNK1                - -----------------------------------------------------------------  -
Myotis lucifugus CXNK1           - -----------------------------------------------------------------  -
Dasypus novemcinctus CXNK1       - -----------------------------------------------------------------  -
Dasypus novemcinctus CXNK2       - -----------------------------------------------------------------  -
Loxodonta africana CXNK1         - -----------------------------------------------------------------  -
Loxodonta africana CXNK2         - -----------------------------------------------------------------  -
Homo sapiens CXNL                - -----------------------------------------------------------------  -
Pan troglodytes CXNL             - -----------------------------------------------------------------  -
Pongo abelii CXNL                - -----------------------------------------------------------------  -
Nomascus leucogenys CXNL         - -----------------------------------------------------------------  -
Macaca mulatta CXNL              - -----------------------------------------------------------------  -
Callithrix jacchus CXNL          - -----------------------------------------------------------------  -
Otolemur garnettii CXNL          - -----------------------------------------------------------------  -
Mus musculus Cxnl                - -----------------------------------------------------------------  -
Rattus norvegicus Cxnl           - -----------------------------------------------------------------  -
Cavia porcellus CXNL             - -----------------------------------------------------------------  -
Oryctolagus cuniculus CXNL       - -----------------------------------------------------------------  -
Ochotona princeps CXNL           - -----------------------------------------------------------------  -
Bos taurus CXNL                  - -----------------------------------------------------------------  -
Equus caballus CXNL              - -----------------------------------------------------------------  -
Canis lupus familiaris CXNL      - -----------------------------------------------------------------  -
Felis catus CXNL                 - -----------------------------------------------------------------  -
Pteropus vampyrus CXNL           - -----------------------------------------------------------------  -
Sorex araneus CXNL               - -----------------------------------------------------------------  -
Dasypus novemcinctus CXNL        - -----------------------------------------------------------------  -
Loxodonta africana CXNL          - -----------------------------------------------------------------  -
Homo sapiens CXNM                - -----------------------------------------------------------------  -
Pan troglodytes CXNM             - -----------------------------------------------------------------  -
Pongo abelii CXNM                - -----------------------------------------------------------------  -
Nomascus leucogenys CXNM         - -----------------------------------------------------------------  -
Macaca mulatta CXNM              - -----------------------------------------------------------------  -
Callithrix jacchus CXNM          - -----------------------------------------------------------------  -
Tarsius syrichta CXNM            - -----------------------------------------------------------------  -
Microcebus murinus CXNM          - -----------------------------------------------------------------  -
Dipodomys ordii CXNM             - -----------------------------------------------------------------  -
Oryctolagus cuniculus CXNM       - -----------------------------------------------------------------  -
Equus caballus CXNM              - -----------------------------------------------------------------  -
Canis lupus familiaris CXNM      - -----------------------------------------------------------------  -
Pteropus vampyrus CXNM           - -----------------------------------------------------------------  -
Loxodonta africana CXNM          - -----------------------------------------------------------------  -
```

Homo sapiens CXNN - --- -
Pan troglodytes CXNN - --- -
Pongo abelii CXNN - --- -
Nomascus leucogenys CXNN - --- -
Macaca mulatta CXNN - --- -
Callithrix jacchus CXNN - --- -
Microcebus murinus CXNN - --- -
Mus musculus Cxnn - --- -
Rattus norvegicus Cxnn - --- -
Oryctolagus cuniculus CXNN - --- -
Bos taurus CXNN - --- -
Equus caballus CXNN - --- -
Canis lupus familiaris CXNN - --- -
Sorex araneus CXNN - --- -
Loxodonta africana CXNN - --- -
Homo sapiens CXNO - --- -
Papio hamadryas CXNO - --- -
Mus musculus Cxno - --- -
Cavia porcellus CXNO - --- -
Homo sapiens CXNP1 - --- -
Pan troglodytes CXNP1 - --- -
Pongo abelii CXNP1 - --- -
Callithrix jacchus CXNP1 - --- -
Otolemur garnettii CXNP1 - --- -
Tupaia belangeri CXNP1 - --- -
Mus musculus Cxnp1 - --- -
Rattus norvegicus Cxnp1 - --- -
Cavia porcellus CXNP1 - --- -
Oryctolagus cuniculus CXNP1 - --- -
Oryctolagus cuniculus CXNP2 - --- -
Ochotona princeps CXNP1 - --- -
Bos taurus CXNP1 - --- -
Equus caballus CXNP1 - --- -
Canis lupus familiaris CXNP1 - --- -
Felis catus CXNP1 - --- -
Myotis lucifugus CXNP1 - --- -
Dasypus novemcinctus CXNP1 - --- -
Dasypus novemcinctus CXNP2 - --- -
Dasypus novemcinctus CXNP3 - --- -
Dasypus novemcinctus CXNP4 - --- -
Choloepus hoffmanni CXNP1 - --- -
Loxodonta africana CXNP1 - --- -
Homo sapiens CXNQ - --- -
Pan troglodytes CXNQ - --- -
Pongo abelii CXNQ - --- -
Macaca mulatta CXNQ - --- -
Tupaia belangeri CXNQ - --- -
Mus musculus Cxnq - --- -
Rattus norvegicus Cxnq - --- -
Cavia porcellus CXNQ - --- -
S.tridecemlineatus CXNQ - --- -
Oryctolagus cuniculus CXNQ - --- -
Bos taurus CXNQ - --- -
Vicugna pacos CXNQ - --- -
Equus caballus CXNQ - --- -
Canis lupus familiaris CXNQ - --- -
Myotis lucifugus CXNQ - --- -
Pteropus vampyrus CXNQ - --- -
Loxodonta africana CXNQ - --- -
Homo sapiens CXNR - --- -
Pan troglodytes CXNR - --- -
Pongo abelii CXNR - --- -
Papio hamadryas CXNR - --- -
Mus musculus Cxnr - --- -
Rattus norvegicus Cxnr - --- -
Oryctolagus cuniculus CXNR - --- -
Bos taurus CXNR - --- -
Homo sapiens CXNS - --- -
Pan troglodytes CXNS - --- -
Pongo abelii CXNS - --- -
Nomascus leucogenys CXNS - --- -
Macaca mulatta CXNS - --- -
Papio hamadryas CXNS - --- -
Otolemur garnettii CXNS - --- -
Tupaia belangeri CXNS - --- -
Mus musculus Cxns - --- -
Rattus norvegicus Cxns - --- -
Cavia porcellus CXNS - --- -
S.tridecemlineatus CXNS - --- -

```
Oryctolagus cuniculus CXNS     - -----------------------------------------------------------------   -
Ochotona princeps CXNS         - -----------------------------------------------------------------   -
Bos taurus CXNS                - -----------------------------------------------------------------   -
Equus caballus CXNS            - -----------------------------------------------------------------   -
Canis lupus familiaris CXNS    - -----------------------------------------------------------------   -
Myotis lucifugus CXNS          - -----------------------------------------------------------------   -
Dasypus novemcinctus CXNS      - -----------------------------------------------------------------   -
Loxodonta africana CXNS        - -----------------------------------------------------------------   -
Homo sapiens CXNT              - -----------------------------------------------------------------   -
Pan troglodytes CXNT           - -----------------------------------------------------------------   -
Nomascus leucogenys CXNT       - -----------------------------------------------------------------   -
Mus musculus Cxnt              - -----------------------------------------------------------------   -
Rattus norvegicus Cxnt         - -----------------------------------------------------------------   -
Cavia porcellus CXNT           - -----------------------------------------------------------------   -
Oryctolagus cuniculus CXNT     - -----------------------------------------------------------------   -
Bos taurus CXNT                - -----------------------------------------------------------------   -
Vicugna pacos CXNT             - -----------------------------------------------------------------   -
Equus caballus CXNT            - -----------------------------------------------------------------   -
Canis lupus familiaris CXNT    - -----------------------------------------------------------------   -
Myotis lucifugus CXNT          - -----------------------------------------------------------------   -
Dasypus novemcinctus CXNT      - -----------------------------------------------------------------   -
Loxodonta africana CXNT        - -----------------------------------------------------------------   -
Homo sapiens CXNU              - -----------------------------------------------------------------   -
Pan troglodytes CXNU           - -----------------------------------------------------------------   -
Pongo abelii CXNU              - -----------------------------------------------------------------   -
Nomascus leucogenys CXNU       - -----------------------------------------------------------------   -
Macaca mulatta CXNU            - -----------------------------------------------------------------   -
Mus musculus Cxnu              - -----------------------------------------------------------------   -
Cavia porcellus CXNU           - -----------------------------------------------------------------   -
Bos taurus CXNU                - -----------------------------------------------------------------   -
Equus caballus CXNU            - -----------------------------------------------------------------   -
Myotis lucifugus CXNU          - -----------------------------------------------------------------   -
Sorex araneus CXNU             - -----------------------------------------------------------------   -
Dasypus novemcinctus CXNU      - -----------------------------------------------------------------   -
Loxodonta africana CXNU        - -----------------------------------------------------------------   -
```

```
Homo sapiens CXNA              - -----------------------------------------------------------------  -
                                     70        80        90       100       110       120       130
Homo sapiens CXNA              - -----------------------------------------------------------------  -
Pan troglodytes CXNA           - -----------------------------------------------------------------  -
Gorilla gorilla CXNA           - -----------------------------------------------------------------  -
Pongo abelii CXNA              - -----------------------------------------------------------------  -
Nomascus leucogenys CXNA       - -----------------------------------------------------------------  -
Macaca mulatta CXNA            - -----------------------------------------------------------------  -
Callithrix jacchus CXNA        - -----------------------------------------------------------------  -
Mus musculus Cxna              - -----------------------------------------------------------------  -
Rattus norvegicus Cxna         - -----------------------------------------------------------------  -
Cavia porcellus CXNA           1 --------------------------------------------------------MGTIVSPIP  9
Ochotona princeps CXNA         - -----------------------------------------------------------------  -
Bos taurus CXNA                - -----------------------------------------------------------------  -
Equus caballus CXNA            - -----------------------------------------------------------------  -
Canis lupus familiaris CXNA    - -----------------------------------------------------------------  -
Felis catus CXNA               - -----------------------------------------------------------------  -
Myotis lucifugus CXNA          - -----------------------------------------------------------------  -
Dasypus novemcinctus CXNA      - -----------------------------------------------------------------  -
Loxodonta africana CXNA        - -----------------------------------------------------------------  -
Homo sapiens CXNB              - -----------------------------------------------------------------  -
Gorilla gorilla CXNB           - -----------------------------------------------------------------  -
Nomascus leucogenys CXNB       - -----------------------------------------------------------------  -
Macaca mulatta CXNB            - -----------------------------------------------------------------  -
Callithrix jacchus CXNB        - -----------------------------------------------------------------  -
Otolemur garnettii CXNB        - -----------------------------------------------------------------  -
Mus musculus Cxnb              - -----------------------------------------------------------------  -
Rattus norvegicus Cxnb         - -----------------------------------------------------------------  -
Dipodomys ordii CXNB           - -----------------------------------------------------------------  -
Cavia porcellus CXNB           - -----------------------------------------------------------------  -
Oryctolagus cuniculus CXNB     - -----------------------------------------------------------------  -
Ochotona princeps CXNB         - -----------------------------------------------------------------  -
Bos taurus CXNB                - -----------------------------------------------------------------  -
Equus caballus CXNB            - -----------------------------------------------------------------  -
Canis lupus familiaris CXNB    1 --------------------------------------------------------MAIPTPRHR  9
Myotis lucifugus CXNB          - -----------------------------------------------------------------  -
Erinaceus europaeus CXNB       - -----------------------------------------------------------------  -
Loxodonta africana CXNB        - -----------------------------------------------------------------  -
Homo sapiens CXNC              - -----------------------------------------------------------------  -
Gorilla gorilla CXNC           - -----------------------------------------------------------------  -
Pongo abelii CXNC              - -----------------------------------------------------------------  -
Nomascus leucogenys CXNC       - -----------------------------------------------------------------  -
Callithrix jacchus CXNC        - -----------------------------------------------------------------  -
Microcebus murinus CXNC        - -----------------------------------------------------------------  -
Otolemur garnettii CXNC        - -----------------------------------------------------------------  -
Mus musculus Cxnc              - -----------------------------------------------------------------  -
Rattus norvegicus Cxnc         - -----------------------------------------------------------------  -
Cavia porcellus CXNC           - -----------------------------------------------------------------  -
Oryctolagus cuniculus CXNC     5 GPSESQEISWVSHVGAEAQALGPSSAAFPGHSRELDWKWSSQDLNWCPYGMPGTASGGLTFSTTA  69
Bos taurus CXNC                - -----------------------------------------------------------------  -
Equus caballus CXNC            - -----------------------------------------------------------------  -
Canis lupus familiaris CXNC    - -----------------------------------------------------------------  -
Felis catus CXNC               - -----------------------------------------------------------------  -
Myotis lucifugus CXNC          - -----------------------------------------------------------------  -
Dasypus novemcinctus CXNC      - -----------------------------------------------------------------  -
Loxodonta africana CXNC        - -----------------------------------------------------------------  -
Homo sapiens CXND              - -----------------------------------------------------------------  -
Pan troglodytes CXND           - -----------------------------------------------------------------  -
Macaca mulatta CXND            - -----------------------------------------------------------------  -
Callithrix jacchus CXND        - -----------------------------------------------------------------  -
Dipodomys ordii CXND           - -----------------------------------------------------------------  -
Oryctolagus cuniculus CXND     - -----------------------------------------------------------------  -
Tursiops truncatus CXND        - -----------------------------------------------------------------  -
Bos taurus CXND                - -----------------------------------------------------------------  -
Equus caballus CXND            - -----------------------------------------------------------------  -
Canis lupus familiaris CXND    - -----------------------------------------------------------------  -
Myotis lucifugus CXND          - -----------------------------------------------------------------  -
Erinaceus europaeus CXND       - -----------------------------------------------------------------  -
Dasypus novemcinctus CXND      - -----------------------------------------------------------------  -
Choloepus hoffmanni CXND       - -----------------------------------------------------------------  -
Loxodonta africana CXND        - -----------------------------------------------------------------  -
Homo sapiens CXNE              - -----------------------------------------------------------------  -
Pan troglodytes CXNE           - -----------------------------------------------------------------  -
Pongo abelii CXNE              - -----------------------------------------------------------------  -
Macaca mulatta CXNE            - -----------------------------------------------------------------  -
Papio hamadryas CXNE           - -----------------------------------------------------------------  -
Callithrix jacchus CXNE        - -----------------------------------------------------------------  -
Otolemur garnettii CXNE        - -----------------------------------------------------------------  -
Mus musculus Cxne              - -----------------------------------------------------------------  -
```

Rattus norvegicus Cxne	-	---	-
Dipodomys ordii CXNE	-	---	-
Cavia porcellus CXNE	1	---MGDRVR	6
Oryctolagus cuniculus CXNE	-	---	-
Equus caballus CXNE	-	---	-
Canis lupus familiaris CXNE	-	---	-
Felis catus CXNE	-	---	-
Myotis lucifugus CXNE	-	---	-
Sorex araneus CXNE	-	---	-
Dasypus novemcinctus CXNE	-	---	-
Loxodonta africana CXNE	-	---	-
Homo sapiens CXNF	-	---	-
Pan troglodytes CXNF	-	---	-
Gorilla gorilla CXNF	-	---	-
Nomascus leucogenys CXNF	-	---	-
Callithrix jacchus CXNF	-	---	-
Mus musculus Cxnf	-	---	-
Rattus norvegicus Cxnf	-	---	-
S.tridecemlineatus CXNF	-	---	-
Oryctolagus cuniculus CXNF	-	---	-
Ochotona princeps CXNF	-	---	-
Bos taurus CXNF	-	---	-
Equus caballus CXNF	-	---	-
Canis lupus familiaris CXNF	-	---	-
Myotis lucifugus CXNF	-	---	-
Dasypus novemcinctus CXNF	-	---	-
Choloepus hoffmanni CXNF	-	---	-
Loxodonta africana CXNF	-	---	-
Homo sapiens CXNG	-	---	-
Pan troglodytes CXNG	-	---	-
Gorilla gorilla CXNG	-	---	-
Pongo abelii CXNG	-	---	-
Nomascus leucogenys CXNG	-	---	-
Macaca mulatta CXNG	-	---	-
Papio hamadryas CXNG	-	---	-
Callithrix jacchus CXNG	-	---	-
Microcebus murinus CXNG	-	---	-
Mus musculus Cxng	-	---	-
Rattus norvegicus Cxng	-	---	-
Oryctolagus cuniculus CXNG	-	---	-
Bos taurus CXNG	-	---	-
Equus caballus CXNG	-	---	-
Canis lupus familiaris CXNG	-	---	-
Felis catus CXNG	-	---	-
Myotis lucifugus CXNG	-	---	-
Pteropus vampyrus CXNG	-	---	-
Dasypus novemcinctus CXNG	-	---	-
Loxodonta africana CXNG	-	---	-
Procavia capensis CXNG	-	---	-
Homo sapiens CXNH1	-	---	-
Pan troglodytes CXNH1	-	---	-
Gorilla gorilla CXNH1	-	---	-
Pongo abelii CXNH1	-	---	-
Nomascus leucogenys CXNH1	-	---	-
Macaca mulatta CXNH1	-	---	-
Otolemur garnettii CXNH1	-	---	-
Mus musculus Cxnh1	-	---	-
Rattus norvegicus Cxnh1	-	---	-
Cavia porcellus CXNH1	-	---	-
Oryctolagus cuniculus CXNH1	-	---	-
Ochotona princeps CXNH1	-	---	-
Equus caballus CXNH1	-	---	-
Canis lupus familiaris CXNH1	-	---	-
Felis catus CXNH1	-	---	-
Myotis lucifugus CXNH1	-	---	-
Dasypus novemcinctus CXNH1	-	---	-
Loxodonta africana CXNH1	-	---	-
Homo sapiens CXNI	-	---	-
Pan troglodytes CXNI	-	---	-
Pongo abelii CXNI	-	---	-
Nomascus leucogenys CXNI	-	---	-
Macaca mulatta CXNI	-	---	-
Papio hamadryas CXNI	-	---	-
Callithrix jacchus CXNI	-	---	-
Otolemur garnettii CXNI	-	---	-
Mus musculus Cxni	-	---	-
Rattus norvegicus Cxni	-	---	-
Cavia porcellus CXNI	-	---	-
Oryctolagus cuniculus CXNI	-	---	-

Bos taurus CXNI	-	---	-
Equus caballus CXNI	-	---	-
Canis lupus familiaris CXNI	-	---	-
Felis catus CXNI	-	---	-
Pteropus vampyrus CXNI	-	---	-
Erinaceus europaeus CXNI	-	---	-
Dasypus novemcinctus CXNI	-	---	-
Loxodonta africana CXNI	-	---	-
Homo sapiens CXNJ1	-	---	-
Pongo abelii CXNJ1	-	---	-
Macaca mulatta CXNJ1	-	---	-
Mus musculus Cxnj1	-	---	-
Rattus norvegicus Cxnj1	-	---	-
Bos taurus CXNJ1	-	---	-
Equus caballus CXNJ1	-	---	-
Myotis lucifugus CXNJ1	-	---	-
Myotis lucifugus CXNJ2	-	---	-
Pteropus vampyrus CXNJ1	66	TCMNCTRKWLYTCTHVCKCMWWICMYMPHAYTCAHMHARVYTHAHTPAQSSVAQPTRDMAGQPPS	130
Pteropus vampyrus CXNJ2	1	---------------MHAHRPHTCTQHMHTHIYSHIYPHVHARTHTPAESSVGEPTRELAGQPPN	50
Sorex araneus CXNJ1	-	---	-
Homo sapiens CXNK1	-	---	-
Homo sapiens CXNK2	-	---	-
Pan troglodytes CXNK1	-	---	-
Pan troglodytes CXNK2	-	---	-
Pongo abelii CXNK1	-	---	-
Nomascus leucogenys CXNK1	-	---	-
Callithrix jacchus CXNK1	-	---	-
Mus musculus Cxnk1	-	---	-
Mus musculus Cxnk2	-	---	-
Rattus norvegicus Cxnk1	-	---	-
Rattus norvegicus Cxnk2	-	---	-
Cavia porcellus CXNK1	-	---	-
Oryctolagus cuniculus CXNK1	-	---	-
Bos taurus CXNK1	-	---	-
Vicugna pacos CXNK1	-	---	-
Equus caballus CXNK1	-	---	-
Equus caballus CXNK2	-	---	-
Canis lupus familiaris CXNK1	-	---	-
Canis lupus familiaris CXNK2	-	---	-
Felis catus CXNK1	-	---	-
Myotis lucifugus CXNK1	-	---	-
Dasypus novemcinctus CXNK1	-	---	-
Dasypus novemcinctus CXNK2	-	---	-
Loxodonta africana CXNK1	-	---	-
Loxodonta africana CXNK2	-	---	-
Homo sapiens CXNL	-	---	-
Pan troglodytes CXNL	-	---	-
Pongo abelii CXNL	-	---	-
Nomascus leucogenys CXNL	-	---	-
Macaca mulatta CXNL	-	---	-
Callithrix jacchus CXNL	-	---	-
Otolemur garnettii CXNL	1	---MVLL	4
Mus musculus Cxnl	-	---	-
Rattus norvegicus Cxnl	-	---	-
Cavia porcellus CXNL	-	---	-
Oryctolagus cuniculus CXNL	-	---	-
Ochotona princeps CXNL	-	---	-
Bos taurus CXNL	-	---	-
Equus caballus CXNL	-	---	-
Canis lupus familiaris CXNL	-	---	-
Felis catus CXNL	-	---	-
Pteropus vampyrus CXNL	-	---	-
Sorex araneus CXNL	-	---	-
Dasypus novemcinctus CXNL	-	---	-
Loxodonta africana CXNL	-	---	-
Homo sapiens CXNM	-	---	-
Pan troglodytes CXNM	-	---	-
Pongo abelii CXNM	-	---	-
Nomascus leucogenys CXNM	-	---	-
Macaca mulatta CXNM	-	---	-
Callithrix jacchus CXNM	-	---	-
Tarsius syrichta CXNM	-	---	-
Microcebus murinus CXNM	-	---	-
Dipodomys ordii CXNM	-	---	-
Oryctolagus cuniculus CXNM	-	---	-
Equus caballus CXNM	-	---	-
Canis lupus familiaris CXNM	-	---	-
Pteropus vampyrus CXNM	-	---	-
Loxodonta africana CXNM	-	---	-

Homo sapiens CXNN - --- -
Pan troglodytes CXNN - --- -
Pongo abelii CXNN - --- -
Nomascus leucogenys CXNN - --- -
Macaca mulatta CXNN - --- -
Callithrix jacchus CXNN - --- -
Microcebus murinus CXNN - --- -
Mus musculus Cxnn - --- -
Rattus norvegicus Cxnn - --- -
Oryctolagus cuniculus CXNN - --- -
Bos taurus CXNN - --- -
Equus caballus CXNN - --- -
Canis lupus familiaris CXNN - --- -
Sorex araneus CXNN - --- -
Loxodonta africana CXNN - --- -
Homo sapiens CXNO - --- -
Papio hamadryas CXNO - --- -
Mus musculus Cxno - --- -
Cavia porcellus CXNO - --- -
Homo sapiens CXNP1 - --- -
Pan troglodytes CXNP1 - --- -
Pongo abelii CXNP1 - --- -
Callithrix jacchus CXNP1 - --- -
Otolemur garnettii CXNP1 - --- -
Tupaia belangeri CXNP1 - --- -
Mus musculus Cxnp1 - --- -
Rattus norvegicus Cxnp1 - --- -
Cavia porcellus CXNP1 - --- -
Oryctolagus cuniculus CXNP1 - --- -
Oryctolagus cuniculus CXNP2 - --- -
Ochotona princeps CXNP1 - --- -
Bos taurus CXNP1 - --- -
Equus caballus CXNP1 - --- -
Canis lupus familiaris CXNP1 - --- -
Felis catus CXNP1 - --- -
Myotis lucifugus CXNP1 - --- -
Dasypus novemcinctus CXNP1 - --- -
Dasypus novemcinctus CXNP2 - --- -
Dasypus novemcinctus CXNP3 - --- -
Dasypus novemcinctus CXNP4 - --- -
Choloepus hoffmanni CXNP1 - --- -
Loxodonta africana CXNP1 - --- -
Homo sapiens CXNQ - --- -
Pan troglodytes CXNQ - --- -
Pongo abelii CXNQ - --- -
Macaca mulatta CXNQ - --- -
Tupaia belangeri CXNQ - --- -
Mus musculus Cxnq - --- -
Rattus norvegicus Cxnq - --- -
Cavia porcellus CXNQ - --- -
S.tridecemlineatus CXNQ - --- -
Oryctolagus cuniculus CXNQ - --- -
Bos taurus CXNQ - --- -
Vicugna pacos CXNQ - --- -
Equus caballus CXNQ - --- -
Canis lupus familiaris CXNQ - --- -
Myotis lucifugus CXNQ - --- -
Pteropus vampyrus CXNQ - --- -
Loxodonta africana CXNQ - --- -
Homo sapiens CXNR - --- -
Pan troglodytes CXNR - --- -
Pongo abelii CXNR - --- -
Papio hamadryas CXNR - --- -
Mus musculus Cxnr - --- -
Rattus norvegicus Cxnr - --- -
Oryctolagus cuniculus CXNR - --- -
Bos taurus CXNR - --- -
Homo sapiens CXNS - --- -
Pan troglodytes CXNS - --- -
Pongo abelii CXNS - --- -
Nomascus leucogenys CXNS - --- -
Macaca mulatta CXNS - --- -
Papio hamadryas CXNS - --- -
Otolemur garnettii CXNS - --- -
Tupaia belangeri CXNS - --- -
Mus musculus Cxns - --- -
Rattus norvegicus Cxns - --- -
Cavia porcellus CXNS - --- -
S.tridecemlineatus CXNS - --- -

Oryctolagus cuniculus CXNS	-	---	-
Ochotona princeps CXNS	-	---	-
Bos taurus CXNS	-	---	-
Equus caballus CXNS	-	---	-
Canis lupus familiaris CXNS	-	---	-
Myotis lucifugus CXNS	-	---	-
Dasypus novemcinctus CXNS	-	---	-
Loxodonta africana CXNS	1	----------------------------MKVYLCLCTFHIDEISLLCTHHVPCMETCTWDLCNME	37
Homo sapiens CXNT	-	---	-
Pan troglodytes CXNT	-	---	-
Nomascus leucogenys CXNT	-	---	-
Mus musculus Cxnt	-	---	-
Rattus norvegicus Cxnt	-	---	-
Cavia porcellus CXNT	-	---	-
Oryctolagus cuniculus CXNT	-	---	-
Bos taurus CXNT	-	---	-
Vicugna pacos CXNT	-	---	-
Equus caballus CXNT	-	---	-
Canis lupus familiaris CXNT	-	---	-
Myotis lucifugus CXNT	-	---	-
Dasypus novemcinctus CXNT	-	---	-
Loxodonta africana CXNT	-	---	-
Homo sapiens CXNU	-	---	-
Pan troglodytes CXNU	-	---	-
Pongo abelii CXNU	-	---	-
Nomascus leucogenys CXNU	-	---	-
Macaca mulatta CXNU	-	---	-
Mus musculus Cxnu	-	---	-
Cavia porcellus CXNU	-	---	-
Bos taurus CXNU	-	---	-
Equus caballus CXNU	-	---	-
Myotis lucifugus CXNU	-	---	-
Sorex araneus CXNU	-	---	-
Dasypus novemcinctus CXNU	-	---	-
Loxodonta africana CXNU	-	---	-

```
Homo sapiens CXNA               1 -----------------------------------------M--N--WSIFEGLLSG-VNKYSTA  19
                                          140       150       160       170       180       190
Homo sapiens CXNA               1 -----------------------------------------M--N--WSIFEGLLSG-VNKYSTA  19
Pan troglodytes CXNA            1 -----------------------------------------M--N--WSIFEGLLSG-VNKYSTA  19
Gorilla gorilla CXNA            1 -----------------------------------------M--N--WSIFEGLLSG-VNKYSTA  19
Pongo abelii CXNA               1 -----------------------------------------M--N--WSIFEGLLSG-VNKYSTA  19
Nomascus leucogenys CXNA        1 -----------------------------------------M--N--WSIFEGLLSG-VNKYSTA  19
Macaca mulatta CXNA             1 -----------------------------------------M--N--WSIFEGLLSG-VNKYSTA  19
Callithrix jacchus CXNA         1 -----------------------------------------M--N--WGIFEGLLSG-VNKYSTA  19
Mus musculus Cxna               1 -----------------------------------------M--N--WSVFEGLLSG-VNKYSTA  19
Rattus norvegicus Cxna          1 -----------------------------------------M--N--WSVFEGLLSG-VNKYSTA  19
Cavia porcellus CXNA           10 LVVVRIKYDSVFYEGLRTSFLSTWEIILFLPLQWQPTLGSTM--N--WGIFEALLSG-VNKYSTA  69
Ochotona princeps CXNA          1 -----------------------------------------M--N--WGVFEGLLSG-VNKYSTA  19
Bos taurus CXNA                 1 -----------------------------------------M--N--WGIFEGLLSG-ANKYSTA  19
Equus caballus CXNA             1 -----------------------------------------M--N--WGSFEGLLSG-VNKYSTA  19
Canis lupus familiaris CXNA     1 -----------------------------------------M--N--WGVFEGLLSG-VNKYSTA  19
Felis catus CXNA                1 -----------------------------------------M--N--WGIFEGLLSG-VNKYSTA  19
Myotis lucifugus CXNA           1 ---------MFLSSVHSDYLINVGDSYYRSLQQQPPTPGSTM--N--WGIFEGLLSG-VNKYSTA  51
Dasypus novemcinctus CXNA       1 -----------------------------------------M--N--WGVFEGLLSG-VNKYSTA  19
Loxodonta africana CXNA         1 -----------------------------------------M--N--WGIFEGLLSG-VNKYSTA  19
Homo sapiens CXNB               1 -----------------------------------------M--N--WAFLQGLLSG-VNKYSTV  19
Gorilla gorilla CXNB            1 -----------------------------------------M--N--WAFLQGLLSG-VNKYSTA  19
Nomascus leucogenys CXNB        1 -----------------------------------------M--N--WAFLQGLLSG-VNKYSTA  19
Macaca mulatta CXNB             1 -----------------------------------------M--N--WAFLQGLLSG-VNKYSTA  19
Callithrix jacchus CXNB         1 -----------------------------------------M--N--WAFLQGLLSG-VNKYSTA  19
Otolemur garnettii CXNB         1 -----------------------------------------M--N--WAFLQGLLSG-VNKYSTA  19
Mus musculus Cxnb               1 -----------------------------------------M--N--WGFLQGILSG-VNKYSTA  19
Rattus norvegicus Cxnb          1 -----------------------------------------M--N--WGFLQGILSG-VNKYSTA  19
Dipodomys ordii CXNB            1 -----------------------------------------M--N--WAFLQGILSG-VNKYSTA  19
Cavia porcellus CXNB            1 -----------------------------------------M--D--WEFLYKRLSG-ANKYSTA  19
Oryctolagus cuniculus CXNB      - -----------------------------------------------------------------   -
Ochotona princeps CXNB          1 -----------------------------------------M--N--WAFLQGLLSG-VNKYSTA  19
Bos taurus CXNB                 1 -----------------------------------------M--N--WAFLRDVLSG-VNKYSTA  19
Equus caballus CXNB             1 -----------------------------------------M--N--WAFLQGLLSG-VNKYSTA  19
Canis lupus familiaris CXNB    10 RHEDVVTGPFFDPGPPSAGSTPRVGAPDGRRSPRGPTDSPGM--N--WASLQGLLSG-VNKYSTA  69
Myotis lucifugus CXNB           1 -----------------------------------------M--N--WASLQGLLSG-VNKYSTA  19
Erinaceus europaeus CXNB        1 -----------------------------------------M--N--WASLQATLSG-VNKYSTR  19
Loxodonta africana CXNB         1 -----------------------------------------M--N--WAFLQGLLSG-VNKYSTA  19
Homo sapiens CXNC               1 -----------------------------------------M--D--WKTLQALLSG-VNKYSTA  19
Gorilla gorilla CXNC            1 -----------------------------------------M--D--WKTLQALLSG-VNKYSTA  19
Pongo abelii CXNC               1 -----------------------------------------M--D--WKTLQALLSG-VNKYSTA  19
Nomascus leucogenys CXNC        1 -----------------------------------------M--D--WKTLQALLSG-VNKYSTA  19
Callithrix jacchus CXNC         1 -----------------------------------------M--D--WKTLQALLSG-VNKYSTA  19
Microcebus murinus CXNC         1 -----------MLSEVTSSSISRFFLFFPRWARPPEPLPGAM--D--WKTFQALLSG-VNKYSTA  49
Otolemur garnettii CXNC         1 -----------------------------------------M--D--WKTLQALLSG-VNKYSTA  19
Mus musculus Cxnc               1 -----------------------------------------M--D--WKKLQDLLSG-VNQYSTA  19
Rattus norvegicus Cxnc          1 -----------------------------------------M--D--WKKLQDLLSG-VNQYSTA  19
Cavia porcellus CXNC            1 -----------------------------------------M--D--WKTFQALLSG-VNKYSTA  19
Oryctolagus cuniculus CXNC     70 LAPKYIYSSLKRSAWHLARGKCSPVHCLHRWARPPEPPPGTM--D--WKTLQALLSG-VNKYSTA 129
Bos taurus CXNC                 1 -----------------------------------------M--D--WKTLQALLSG-VNKYSTA  19
Equus caballus CXNC             1 -----------------------------------------M--D--WKTFQALLSG-VNKYSTA  19
Canis lupus familiaris CXNC     1 -----------------------------------------M--D--WKTLQALLSG-VNKYSTA  19
Felis catus CXNC                1 -----------------------------------------M--D--WKTLQALLSG-VNKYSTA  19
Myotis lucifugus CXNC           1 -----------------------------------------M--D--WKTLQSLLSG-VNKYSTA  19
Dasypus novemcinctus CXNC       1 -----------------------------------------M--D--WKTLQGLLSG-VNKYSTA  19
Loxodonta africana CXNC         1 -----------------------------------------M--D--WKTFQALLSG-VNKYSTA  19
Homo sapiens CXND               1 -----------------------------------------M--S--WMFLRDLLSG-VNKYSTG  19
Pan troglodytes CXND            1 -----------------------------------------M--S--WMFLRDLLSG-VNKYSTG  19
Macaca mulatta CXND             1 -----------------------------------------M--S--WMFLRDLLSG-VNKYSTG  19
Callithrix jacchus CXND         1 -----------------------------------------M--S--WMFLRDLLSG-VNKYSTG  19
Dipodomys ordii CXND            1 -----------------------------------------M--S--WMFLRDLLSG-VNKYSTG  19
Oryctolagus cuniculus CXND      1 -----------------------------------------M--S--WMFLRDLLSG-VNKYSTG  19
Tursiops truncatus CXND         1 -----------------------------------------M--S--WTFLRDLLSG-VNKYSTG  19
Bos taurus CXND                 - -----------------------------------------------------------------   -
Equus caballus CXND             1 ----------------------MNHDEIVFFCRILPSFEPVM--S--WMFLRDLLSG-VNKYSTG  38
Canis lupus familiaris CXND     1 -----------------------------------------M--S--WMFFRDLLTG-VNKYSTG  19
Myotis lucifugus CXND           1 -----------------------------------------M--S--WIFLRDLLSG-VNKYSTG  19
Erinaceus europaeus CXND        1 -----------------------------------------M--S--WMFLRDLLSG-VNKYSTG  19
Dasypus novemcinctus CXND       1 -----------------------------------------M--S--WMFLRDLLGG-VNKYSTE  19
Choloepus hoffmanni CXND        1 -----------------------------------------M--S--WMFLRDLLSG-VNKYSTG  19
Loxodonta africana CXND         1 -----------------------------------------M--S--WKFLRDLLSG-VNKYSTG  19
Homo sapiens CXNE               1 -----------------------------------------M--D--WGTLQTILGG-VNKHSTS  19
Pan troglodytes CXNE            1 -----------------------------------------M--D--WGTLQTILGG-VNKHSTS  19
Pongo abelii CXNE               1 -----------------------------------------M--D--WGALQTILGG-VNKHSTS  19
Macaca mulatta CXNE             1 -----------------------------------------M--D--WGALQTILGG-VNKYSTS  19
Papio hamadryas CXNE            1 -----------------------------------------M--D--WGALQTILGG-VNKYSTS  19
Callithrix jacchus CXNE         1 -----------------------------------------M--D--WSTLQTILGG-VNKHSTS  19
Otolemur garnettii CXNE         1 -----------------------------------------M--D--WSTLHTILGG-VNKHSTS  19
Mus musculus Cxne               1 ----------------------------MFVFSSANHPEDKM--D--WGTLQSILGG-VNKHSTS  32
```

```
Rattus norvegicus Cxne          1 ----------------------MIPVLHFFVFSSANHPEDKM--D--WGTLQSILGG-VNKHSTS  38
Dipodomys ordii CXNE            1 ----------------------MIPMLCSFVFFSVHRSEGRM--D--WGALQTILGG-VNKHSTS  38
Cavia porcellus CXNE            7 VRQRLRSTFLLKQETSWVLSKPMTAVLCVFVFSSTNLSVDRM--D--WGALHAILGG-VNKYSTS  66
Oryctolagus cuniculus CXNE      1 ----------------------MAPVLCPLVFSSAHRAEGRM--D--WSTLQTILGG-VNKHSTS  38
Equus caballus CXNE             1 -----------------------------------------M--D--WSTLQTILGG-VNKHSTS  19
Canis lupus familiaris CXNE     1 -----------------------------------------M--D--WSTLQTILGG-VNKHSTS  19
Felis catus CXNE                1 -----------------------------------------M--D--WSTLQTILGG-VNKHSTS  19
Myotis lucifugus CXNE           1 -----------------------------------------M--D--WSTLHTILGG-VNKHSTS  19
Sorex araneus CXNE              1 -----------------------------------------M--D--WSTLHTILGG-VNKYSTS  19
Dasypus novemcinctus CXNE       1 -----------------------------------------M--D--WGSLQTILGG-VNKYSTS  19
Loxodonta africana CXNE         1 -----------------------------------------M--D--WSTLQTILGG-VNKHSTS  19
Homo sapiens CXNF               1 -----------------------------------------M--D--WGTLHTFIGG-VNKHSTS  19
Pan troglodytes CXNF            1 -----------------------------------------M--D--WGTLHTFIGG-VNKHSTS  19
Gorilla gorilla CXNF            1 -----------------------------------------M--D--WGTLHTFIGG-VNKHSTS  19
Nomascus leucogenys CXNF        1 -----------------------------------------M--D--WGTLHTFIGG-VNKHSTS  19
Callithrix jacchus CXNF         1 -----------------------------------------M--D--WGTLHAVIGG-VNKHSTS  19
Mus musculus Cxnf               1 -----------------------------------------M--D--WGTLHTVIGG-VNKHSTS  19
Rattus norvegicus Cxnf          1 -----------------------------------------M--D--WGTLHTVIGG-VNKHSTS  19
S.tridecemlineatus CXNF         1 -----------------------------------------M--D--WGTLHTFIGG-VNKHSTS  19
Oryctolagus cuniculus CXNF      1 -----------------------------------------M--D--WGTLHAFIGG-VNKHSTN  19
Ochotona princeps CXNF          1 -----------------------------------------M--D--WGTLHTFIGG-VNKHSTS  19
Bos taurus CXNF                 1 -----------------------------------------M--D--WGTLHTFVGG-VNKHSTS  19
Equus caballus CXNF             1 -----------------------------------------M--D--WGTLHTFIGG-VNKHSTS  19
Canis lupus familiaris CXNF     1 -----------------------------------------M--D--WGTLHTFIGG-VNKHSTS  19
Myotis lucifugus CXNF           1 -----------------------------------------M--D--WGTLHTFIGG-VNKHSTS  19
Dasypus novemcinctus CXNF       1 -----------------------------------------M--D--WGSLYTFIGG-VNKHSTS  19
Choloepus hoffmanni CXNF        1 -----------------------------------------M--D--WDTLHTLLGG-SNRHSTS  19
Loxodonta africana CXNF         1 -----------------------------------------M--D--WGTLHTFIGG-VNKHSTS  19
Homo sapiens CXNG               1 -----------------------------------------M--N--WTGLYTLLSG-VNRHSTA  19
Pan troglodytes CXNG            1 -----------------------------------------M--N--WTGLYTLLSG-VNRHSTA  19
Gorilla gorilla CXNG            1 -----------------------------------------M--N--WTGLYTLLSG-VNRHSTA  19
Pongo abelii CXNG               1 -----------------------------------------M--N--WTGLYTLLSG-VNRHSTA  19
Nomascus leucogenys CXNG        1 -----------------------------------------M--N--WTGLYTLLSG-VNRHSTA  19
Macaca mulatta CXNG             1 -----------------------------------------M--N--WTGLYTLLSG-VNRHSTA  19
Papio hamadryas CXNG            1 -----------------------------------------M--N--WTGLYTLLSG-VNRHSTA  19
Callithrix jacchus CXNG         1 -----------------------------------------M--N--WTGLYTLLSG-VNRHSTA  19
Microcebus murinus CXNG         1 -----------------------------------------M--N--WTGLYTLLSG-VNRHSTA  19
Mus musculus Cxng               1 -----------------------------------------M--N--WTGLYTLLSG-VNRHSTA  19
Rattus norvegicus Cxng          1 -----------------------------------------M--N--WTGLYTLLSG-VNRHSTA  19
Oryctolagus cuniculus CXNG      1 -----------------------------------------M--N--WTGLYTLLSG-VNRHSTA  19
Bos taurus CXNG                 1 -----------------------------------------M--N--WTGLYTLLSG-VNRHSTA  19
Equus caballus CXNG             1 -----------------------------------------M--N--WTGLYTLLSG-VNRHSTA  19
Canis lupus familiaris CXNG     1 -----------------------------------------M--N--WTGLYTLLSG-VNRHSTA  19
Felis catus CXNG                1 -----------------------------------------M--N--WTGLYTLLSG-VNRHSTA  19
Myotis lucifugus CXNG           1 -----------------------------------------M--N--WTGLYTVLSG-VNRHSTA  19
Pteropus vampyrus CXNG          1 -----------------------------------------M--N--WTGLYTLLSG-VNRHSTA  19
Dasypus novemcinctus CXNG       1 -----------------------------------------M--N--WTGLYTLLSG-VNRHSTA  19
Loxodonta africana CXNG         1 -----------------------------------------M--N--WTGLYTLLSG-VNRHSTA  19
Procavia capensis CXNG          1 -----------------------------------------M--N--WTGLYTLLSG-VNRHSTA  19
Homo sapiens CXNH1              1 -----------------------------------------M-GD--WGFLEKLLDQ-VQEHSTV  20
Pan troglodytes CXNH1           1 -----------------------------------------M-GD--WGFLEKLLDQ-VQEHSTV  20
Gorilla gorilla CXNH1           1 -----------------------------------------M-GD--WGFLEKLLDQ-VQEHSTV  20
Pongo abelii CXNH1              1 -----------------------------------------M-GD--WGFLEKLLDQ-VQEHSTV  20
Nomascus leucogenys CXNH1       1 -----------------------------------------M-GD--WGFLEKLLDQ-VQEHSTV  20
Macaca mulatta CXNH1            1 -----------------------------------------M-GD--WGFLEKLLDQ-VQEHSTV  20
Otolemur garnettii CXNH1        1 -----------------------------MLCLPADRDPGAM-VN--WDFFKKLLKL-VKEHSTV  32
Mus musculus Cxnh1              1 -----------------------------------------M-GD--WGFLEKLLDQ-VQEHSTV  20
Rattus norvegicus Cxnh1         1 -----------------------------------------M-GD--WGFLEKLLDQ-VQEHSTV  20
Cavia porcellus CXNH1           1 -----------------------------------------M-GD--WGFLEKLLDQ-VQEHSTV  20
Oryctolagus cuniculus CXNH1     1 -----------------------------------------M-GD--WGFLEKLLDQ-VQEHSTV  20
Ochotona princeps CXNH1         1 ---------------------------MSSLPSPPDGGPRTM-GD--WGFLEKLLDQ-VQEHSTV  34
Equus caballus CXNH1            1 -----------------------------------------M-GD--WGFLEKLLHQ-VQEHSTV  20
Canis lupus familiaris CXNH1    1 -----------------------------------------M-GD--WGFLEKLLDQ-VQEHSTV  20
Felis catus CXNH1               1 -----------------------------------------M-GD--WGFLEKLLDQ-VQEHSTV  20
Myotis lucifugus CXNH1          1 ---------MARPAAGLWLGRLRRCHADRVFCLPADRSPGAM-GD--WGFLEKLLDQ-VQQHSTV  52
Dasypus novemcinctus CXNH1      1 -----------------------------------------M-GD--WGFLEKLLDQ-VQEHSTV  20
Loxodonta africana CXNH1        1 -----------------------------------------M-GD--WSFLEKLLDQ-VQEHSTV  20
Homo sapiens CXNI               1 -----------------------------------------M-GD--WSFLGNFLEE-VHKHSTV  20
Pan troglodytes CXNI            1 -----------------------------------------M-GD--WSFLGNFLEE-VHKHSTV  20
Pongo abelii CXNI               1 -----------------------------------------M-GD--WSFLGNFLEE-VHKHSTV  20
Nomascus leucogenys CXNI        1 -----------------------------------------M-GD--WSFLGNFLEE-VHKHSTV  20
Macaca mulatta CXNI             1 -----------------------------------------M-GD--WSFLGEFLEE-VHKHSTV  20
Papio hamadryas CXNI            1 -----------------------------------------M-GD--WSFLGEFLEE-VHKHSTV  20
Callithrix jacchus CXNI         1 -----------------------------------------M-GD--WSFLGEFLEE-VHKHSTV  20
Otolemur garnettii CXNI         1 -----------------------------------------M-GD--WSFLGEFLEE-VHKHSTV  20
Mus musculus Cxni               1 -----------------------------------------M-GD--WSFLGEFLEE-VHKHSTV  20
Rattus norvegicus Cxni          1 -------MMGPSKPLETSHADIFLFLLYLQKAQHQSPTLGKM-GD--WSFLGEFLEE-VHKHSTV  54
Cavia porcellus CXNI            1 -----------------------------------------M-SD--WSFLGAFLEE-VHKHSTV  20
Oryctolagus cuniculus CXNI      1 -----------------------------------------M-GD--WSFLGEFLEE-VHKHSTV  20
```

```
Bos taurus CXNI                   1 -----------------------------------------M-GD--WSFLGEFLEE-VHKHSTV  20
Equus caballus CXNI               1 -----------------------------------------M-GD--WSYLAEFLEE-VHKHSTE  20
Canis lupus familiaris CXNI       1 -----------------------------------------M-GD--WSFLGEFLEE-VHKHSTV  20
Felis catus CXNI                  1 -----------------------------------------M-GD--WSFLGEFLEE-VHKHSTV  20
Pteropus vampyrus CXNI            1 -----------------------------------------M-GD--WSFLGEFLEE-VHKHSTV  20
Erinaceus europaeus CXNI          1 -----------------------------------------M-GD--WSFLGEFLEE-VHKHSTV  20
Dasypus novemcinctus CXNI         1 -----------------------------------------M-GD--WSFLGEFLEE-VHKHSTV  20
Loxodonta africana CXNI           1 -----------------------------------------M-GD--WSFLGEFLEE-VHKHSTV  20
Homo sapiens CXNJ1                1 -----------------------------------------M-GD--WSFLGRLLEN-AQEHSTV  20
Pongo abelii CXNJ1                1 -----------------------------------------M-GD--WSFLGRLLEN-AQEHSTV  20
Macaca mulatta CXNJ1              1 -----------------------------------------M-GD--WSFLGRLLEN-AQEHSTV  20
Mus musculus Cxnj1                1 -----------------------------------------M-GD--WSFLGRLLEN-AQEHSTV  20
Rattus norvegicus Cxnj1           1 -----------------------------------------M-GD--WSFLGRLLEN-AQEHSTV  20
Bos taurus CXNJ1                  1 -----------------------------------------M-GD--WSFLGRLLEN-AQEHSTV  20
Equus caballus CXNJ1              1 -----------------------------------------M-GD--WSFLGKLLEN-AQEHSTV  20
Myotis lucifugus CXNJ1            1 -----------------------------------------M-GD--WSLLGRLLDT-AQGHSTV  20
Myotis lucifugus CXNJ2            1 -----------------------------------------M-GD--WSFLGRLLEN-AQEHSTV  20
Pteropus vampyrus CXNJ1         131 DTIFQMPCTVT--PLGGGPAAHLCYLRINRQCSLTGWQSEAM-GD--WSFLGRLLEN-AQEHSTV 189
Pteropus vampyrus CXNJ2          51 DTASQPPCTATPAPVGGGPAAHLCYLRINRQRSLTGWQSEAM-GD--WSLLGRLLDA-AQEHSTV 111
Sorex araneus CXNJ1               1 -----------------------------------------M-GD--WSLLGRLLEN-AQEHSTV  20
Homo sapiens CXNK1                1 -----------------------------------------M-RD--WSTLGKLLDK-VQAYLTA  20
Homo sapiens CXNK2                1 -----------------------------------------M-GD--WSALGKLLDK-VQAYSTA  20
Pan troglodytes CXNK1             1 -----------------------------------------M-RD--WSTLGKLLDK-FQAYSTA  20
Pan troglodytes CXNK2             1 -----------------------------------------M-GD--WSALGKLLDK-VQAYSTA  20
Pongo abelii CXNK1                1 -----------------------------------------M-GD--WSALGKLLDK-VQAYSTA  20
Nomascus leucogenys CXNK1         1 -----------------------------------------M-GD--WSALGKLLDK-VQAYSTA  20
Callithrix jacchus CXNK1          1 -----------------------------------------M-GD--WSALGKLLDK-VQAYSTA  20
Mus musculus Cxnk1                1 -----------------------------------------M-GD--WSALGKLLDK-VQAYSTA  20
Mus musculus Cxnk2                1 -----------------------------------------M-SD--WSALHQLLEK-VQPYSTA  20
Rattus norvegicus Cxnk1           1 -----------------------------------------M-GD--WSALGKLLDK-VQAYSTA  20
Rattus norvegicus Cxnk2           1 -----------------------------------------M-SD--WSALHQLLEK-VQPYSTA  20
Cavia porcellus CXNK1             1 -----------------------------------------M-GD--WSALGKLLDK-VQAYSTA  20
Oryctolagus cuniculus CXNK1       1 -----------------------------------------M-GD--WSALGKLLDK-VQAYSTA  20
Bos taurus CXNK1                  1 -----------------------------------------M-GD--WSALGKLLDK-VQAYSTA  20
Vicugna pacos CXNK1               1 -----------------------------------------M-GD--WSALGKLLDK-VQAYSTA  20
Equus caballus CXNK1              1 -----------------------------------------M-GD--WSALGKLLDK-VQAYSTA  20
Equus caballus CXNK2              1 -----------------------------------------M-----------------------   1
Canis lupus familiaris CXNK1      1 -----------------------------------------M-GD--WSALGKLLDK-VQAYSTA  20
Canis lupus familiaris CXNK2      1 -----------------------------------------M-SD--WSALGQLLDQ-VQAYSTA  20
Felis catus CXNK1                 1 -----------------------------------------M-SD--WGSLGQLLGK-VQTYSNP  20
Myotis lucifugus CXNK1            1 -----------------------------------------M-GD--WSALGKLLDK-VQAYSTA  20
Dasypus novemcinctus CXNK1        1 -----------------------------------------M-SD--WSSLSQLFDK-VQAYSTP  20
Dasypus novemcinctus CXNK2        1 -----------------------------------------M-GD--WSALGKLLDK-VQAYSTA  20
Loxodonta africana CXNK1          1 -----------------------------------------M-GD--WSALGKLLDK-VQAYSTA  20
Loxodonta africana CXNK2          1 -----------------------------------------M-SD--WSALGQLMDK-VQANSTA  20
Homo sapiens CXNL                 1 -----------------------------------------M-GD--WSFLGNILEE-VNEHSTV  20
Pan troglodytes CXNL              1 -----------------------------------------M-GD--WSFLGNILEE-VNEHSTV  20
Pongo abelii CXNL                 1 -----------------------------------------M-GD--WSFLGNILEE-VNEHSTI  20
Nomascus leucogenys CXNL          1 -----------------------------------------M-GD--WSFLGNILEE-VNEHSTV  20
Macaca mulatta CXNL               1 -----------------------------------------M-GD--WSFLGNILEE-VNEHSTV  20
Callithrix jacchus CXNL           1 -----------------------------------------M-GD--WSFLGNILEE-VNEHSTV  20
Otolemur garnettii CXNL           5 HVDCSSNSERRGTPRVSQKRKTQDCTVAAQVFLLPSLGGREM-GD--WSFLGNILEE-VNEHSTV  65
Mus musculus Cxnl                 1 -----------------------------------------M-GD--WSFLGNILEE-VNEHSTV  20
Rattus norvegicus Cxnl            1 -----------------------------------------M-GD--WSFLGNILEE-VNEHSTV  20
Cavia porcellus CXNL              1 -----------------------------------------M-GD--WSFLGNILEE-VNEHSTV  20
Oryctolagus cuniculus CXNL        1 -----------------------------------------M-GD--WSFLGNILEE-VNEHSTV  20
Ochotona princeps CXNL            1 -----------------------------------------M-GD--WSFLGNILEE-VNEHSTV  20
Bos taurus CXNL                   1 -----------------------------------------M-GD--WSFLGNILEE-VNEHSTV  20
Equus caballus CXNL               1 -----------------------------------------M-GD--WSFLGNILEE-VNEHSTV  20
Canis lupus familiaris CXNL       1 -----------------------------------------M-GD--WSFLGNILEE-VNEHSTV  20
Felis catus CXNL                  1 -----------------------------------------M-GD--WSFLGNILEE-VNEHSTV  20
Pteropus vampyrus CXNL            1 -----------------------------------------M-GD--WSFLGNILEE-VNEHSTV  20
Sorex araneus CXNL                1 -----------------------------------------M-GD--WSFLGNLLEE-VNEHSTV  20
Dasypus novemcinctus CXNL         1 -----------------------------------------M-GD--WSFLGNILEE-VNEHSTV  20
Loxodonta africana CXNL           1 -----------------------------------------M-GD--WSFLGNILEE-VNEHSTV  20
Homo sapiens CXNM                 1 -----------------------------------------M-GD--WNLLGDTLEE-VHIHSTM  20
Pan troglodytes CXNM              1 -----------------------------------------M-GD--WNLLGDTLEE-VHIHSTM  20
Pongo abelii CXNM                 1 -----------------------------------------M-GD--WNLLGDTLEE-VHIHSTM  20
Nomascus leucogenys CXNM          1 -----------------------------------------M-GD--WNLLGDTLEE-VHIHSTM  20
Macaca mulatta CXNM               1 -----------------------------------------M-GD--WNLLGDTLEE-VHIHSTT  20
Callithrix jacchus CXNM           1 -----------------------------------------M-GD--WNLLGDTLEE-VHIHSTM  20
Tarsius syrichta CXNM             1 -----------------------------------------M-GD--WNLLGDTLEE-VHIHSTM  20
Microcebus murinus CXNM           1 -----------------------------------------M-GD--WNLLGDTLEE-VHIHSTM  20
Dipodomys ordii CXNM              1 -----------------------------------------M-GD--WNLLEGVLEE-VHIHSTM  20
Oryctolagus cuniculus CXNM        1 -----------------------------------------M-GA--WSLLGRILEE-VHIHSTM  20
Equus caballus CXNM               1 -----------------------------------------M-GD--WYFLGGILEE-VHIHSTM  20
Canis lupus familiaris CXNM       1 -----------------------------------------M-GN--WNFLGGILEE-VHIHSTI  20
Pteropus vampyrus CXNM            1 -----------------------------------------M-GD--WNFLGGILEE-VHTHSTM  20
Loxodonta africana CXNM           1 -----------------------------------------M-GD--WNFLGGILEE-VHIHSTM  20
```

```
Homo sapiens CXNN                1 -----------------------------------------M-GD--WNLLGGILEE-VHSHSTI  20
Pan troglodytes CXNN             1 -----------------------------------------M-GD--WNLLGGILEE-VHSHSTI  20
Pongo abelii CXNN                1 -----------------------------------------M-GD--WNLLGGILEE-VHSHSTI  20
Nomascus leucogenys CXNN         1 -----------------------------------------M-GD--WNLLGGILEE-VHSHSTI  20
Macaca mulatta CXNN              1 -----------------------------------------M-GD--WNLLGGILEE-VHSHSTI  20
Callithrix jacchus CXNN          1 -----------------------------------------M-GD--WNLLGGILEE-VHSHSTI  20
Microcebus murinus CXNN          1 -----------------------------------------M-GD--WNLLGGILEE-VHSHSTI  20
Mus musculus Cxnn                1 -----------------------------------------M-GD--WNLLGGILEE-VHSHSTI  20
Rattus norvegicus Cxnn           1 -----------------------------------------M-GD--WNLLGGILEE-VHSHSTI  20
Oryctolagus cuniculus CXNN       1 -----------MNLCPSAFYTISVSVINLSVFCIFQTLSLIM-GD--WNLLGGILEE-VHYHSTI  50
Bos taurus CXNN                  1 -----------------------------------------M-GD--WNLLGGILED-VHSHSTM  20
Equus caballus CXNN              1 -----------------------------------------M-GD--WNLLGGILEE-VHSHSTI  20
Canis lupus familiaris CXNN      1 -----------------------------------------M-GD--WNLLGGILEE-VHSHSTI  20
Sorex araneus CXNN               1 -----------------------------------------M-GD--WNLLGGILEE-VHSHSTI  20
Loxodonta africana CXNN          1 -----------------------------------------M-GD--WNLLGGILEE-VHSHSTI  20
Homo sapiens CXNO                1 -----------------------------------------M-TNMSWSFLTRLLEE-IHNHSTF  22
Papio hamadryas CXNO             1 -----------------------------------------M-TNMSWSFLTRLLEE-IHNHSTF  22
Mus musculus Cxno                1 ------------------------------MHACRSHLLAPM-TNMSWSFLTRLLEE-IHNHSTF  33
Cavia porcellus CXNO             1 ---------------------------MLTRSVCRSPTPAPM-TNMSWSFLTRLLEE-IHNHSTF  36
Homo sapiens CXNP1               1 -----------------------------------------MCGR----FLRRLLAE-ESRRSTP  19
Pan troglodytes CXNP1            1 -----------------------------------------MCGR----FLRRLLAE-ESRRSTP  19
Pongo abelii CXNP1               1 -----------------------------------------MCGR----FLRRLLAE-ESWHSTP  19
Callithrix jacchus CXNP1         1 -----------------------------------------MCGR----FVRQLLAE-ESRHSTP  19
Otolemur garnettii CXNP1         1 -----------------------------------------MCGR----LLWRLLAE-EARYSTP  19
Tupaia belangeri CXNP1           1 -----------------------MNSEQHNVTNVYPLLHNRMCGS----FLRKLLTE-ESRHSTP  37
Mus musculus Cxnp1               1 -----------------------------------------MCGR----FLRQLLAQ-ESQHSTP  19
Rattus norvegicus Cxnp1          1 -----------------------------------------MCCS----FLRQLLAR-ESQHSTP  19
Cavia porcellus CXNP1            1 -----------------------------------------MCAR----FLRQLLAQ-ESQCSTP  19
Oryctolagus cuniculus CXNP1      1 -----------------------------------------MRGR----FLRQLLAE-ESRHSTA  19
Oryctolagus cuniculus CXNP2      1 -----------------------------------------MCGP----FLRRLLRE-ESQHSTP  19
Ochotona princeps CXNP1          1 -----------------------------------------MSGQ----FLRQLLAE-ESQHSTA  19
Bos taurus CXNP1                1 -----------------------------------------MCGS----FLRRVAAE-ESRHPTP  19
Equus caballus CXNP1             1 -----------------------------------------MCGR----FLRWLVAE-ESRHSTH  19
Canis lupus familiaris CXNP1     1 -----------------------------------------MCGS----FLRQLLAE-DSRHSTA  19
Felis catus CXNP1                1 -------------------MHLLKIREQYTHTHYCPLLPNRMCGR----FLRQLVAE-ESRHSTS  41
Myotis lucifugus CXNP1           1 -----------------------------------------MCGR----FLRRLMAE-ESWHSTP  19
Dasypus novemcinctus CXNP1       1 -----------------------------------------MSGR----FLRRLLAE-ESRHSSP  19
Dasypus novemcinctus CXNP2       1 -----------------------------------------MSGR----LLRRLLAE-ESRHSSP  19
Dasypus novemcinctus CXNP3       1 -----------------------------------------MCGR----FLRRLLAE-ESRHSSP  19
Dasypus novemcinctus CXNP4       1 -----------------------------------------MSGR----FLRRLLAE-ESRHSSP  19
Choloepus hoffmanni CXNP1        1 -----------------------------------------MCGG----FLRRLLAE-ESRHSTP  19
Loxodonta africana CXNP1         1 -----------------------------------------MCGR----FLRQLLAE-ESRHSTP  19
Homo sapiens CXNQ                1 -----------------------------------------M--S--WSFLTRLLEE-IHNHSTF  19
Pan troglodytes CXNQ             1 -----------------------------------------M--S--WSFLTRLLEE-IHNHSTF  19
Pongo abelii CXNQ                1 -----------------------------------------M--S--WSFLTRLLEE-IHNHSTF  19
Macaca mulatta CXNQ              1 -----------------------------------------M--S--WSFLTRLLEE-IHNHSTF  19
Tupaia belangeri CXNQ            1 -----------------------------------------M--S--WSFLTRLLEE-IHNHSTF  19
Mus musculus Cxnq                1 -----------------------------------------M--S--WSFLTRLLEE-IHNHSTF  19
Rattus norvegicus Cxnq           1 -----------------------------------------M--S--WSFLTRLLEE-IHNHSTF  19
Cavia porcellus CXNQ             1 -----------------------------------------M--S--WSFLTRLLEE-IHNHSTF  19
S.tridecemlineatus CXNQ          1 ---------------------------MIFSTLSGQTKFITM--S--WSFLTRLLEE-IHNHSTF  33
Oryctolagus cuniculus CXNQ       1 -----------------------------------------M--S--WSFLTRLLEE-IHNHSTF  19
Bos taurus CXNQ                  1 -----------------------------------------M--S--WSFLTRLLEE-IHNHSTF  19
Vicugna pacos CXNQ               1 -----------------------------------------M--S--WSFLTRLLEE-IHNHSTF  19
Equus caballus CXNQ              1 -----------------------------------------M--S--WSFLTRLLEE-IHNHSTF  19
Canis lupus familiaris CXNQ      1 -----------------------------------------M--S--WSFLTRLLEE-IHNHSTF  19
Myotis lucifugus CXNQ            1 -----------------------------------------M--S--WSFLTRLLEE-IHNHSTF  19
Pteropus vampyrus CXNQ           1 -----------------------------------------M--S--WSFLTRLLEE-IHNHSTF  19
Loxodonta africana CXNQ          1 -----------------------------------------M--S--WSFLTRLLEE-IHNHSTF  19
Homo sapiens CXNR                1 -----------------------------------------M-GE--WAFLGSLLDA-VQLQSPL  20
Pan troglodytes CXNR             1 -----------------------------------------M-GE--WAFLGSLLDA-VQLQSPL  20
Pongo abelii CXNR                1 -----------------------------------------M-GE--WAFLGSLLDA-VQLQSPL  20
Papio hamadryas CXNR             1 -----------------------------------------M-GE--WAFLGSLLDA-VQLQSPL  20
Mus musculus Cxnr                1 -----------------------------------------M-GE--WAFLGSLLDA-VQLQSPL  20
Rattus norvegicus Cxnr           1 -----------------------------------------M-GE--WAFLGSLLDA-VQLQSPL  20
Oryctolagus cuniculus CXNR       1 -----------------------------------------M-GE--WAFLGSLLDA-VQLQSPL  20
Bos taurus CXNR                  1 -----------------------------------------M-GE--WAFLGSLLDA-VQLQSPL  20
Homo sapiens CXNS                - -----------------------------------------------------------------   -
Pan troglodytes CXNS             - -----------------------------------------------------------------   -
Pongo abelii CXNS                - -----------------------------------------------------------------   -
Nomascus leucogenys CXNS         - -----------------------------------------------------------------   -
Macaca mulatta CXNS              - -----------------------------------------------------------------   -
Papio hamadryas CXNS             - -----------------------------------------------------------------   -
Otolemur garnettii CXNS          - -----------------------------------------------------------------   -
Tupaia belangeri CXNS            - -----------------------------------------------------------------   -
Mus musculus Cxns                1 ------------------------------------------------------MRT-FLPPPLP  10
Rattus norvegicus Cxns           - -----------------------------------------------------------------   -
Cavia porcellus CXNS             - -----------------------------------------------------------------   -
S.tridecemlineatus CXNS          - -----------------------------------------------------------------   -
```

```
Oryctolagus cuniculus CXNS      -  -----------------------------------------------------------------   -
Ochotona princeps CXNS          -  -----------------------------------------------------------------   -
Bos taurus CXNS                 -  -----------------------------------------------------------------   -
Equus caballus CXNS             -  -----------------------------------------------------------------   -
Canis lupus familiaris CXNS     -  -----------------------------------------------------------------   -
Myotis lucifugus CXNS           -  -----------------------------------------------------------------   -
Dasypus novemcinctus CXNS       -  -----------------------------------------------------------------   -
Loxodonta africana CXNS        38  CSQSTSIQEGKRQKGFPDPVPHRGGQLWSQRTLYMLRQGGE-EPGSP-KPVTQPIRT-FLPPSSL  99
Homo sapiens CXNT               1  -----------------------------------------M--SL--NYIKNFYEGCV-KPPTV  19
Pan troglodytes CXNT            1  -----------------------------------------M--SL--NYIKNLYEGCV-KPPTV  19
Nomascus leucogenys CXNT        1  -----------------------------------------M--FL--NYIKNFYEGCV-KPPTV  19
Mus musculus Cxnt               1  -----------------------------------------M--SL--NYIKNFYEGCV-KPPTV  19
Rattus norvegicus Cxnt          1  -----------------------------------------M--SL--NYIKNFYEGCV-KPPTV  19
Cavia porcellus CXNT            1  -----------------------------------------M--SL--NYIKNFYEGCL-KPPTV  19
Oryctolagus cuniculus CXNT      1  -----------------------------------------M--SL--NYIKNFYEGCV-KPPTV  19
Bos taurus CXNT                1  -----------------------------------------M--SL--NYIKNFYEGCM-KPPTV  19
Vicugna pacos CXNT              1  -----------------------------------------M--SL--NYIKNFYEGCM-KPPTV  19
Equus caballus CXNT             1  -----------------------------------------M--SL--NYIKNFYEGCM-KPPTV  19
Canis lupus familiaris CXNT     1  -----------------------------------------M--SL--NYIRNFYEGCV-KPPTV  19
Myotis lucifugus CXNT           1  -----------------------------------------M--SL--NYIKNFYEGCV-KPPTV  19
Dasypus novemcinctus CXNT       1  -----------------------------------------M--SS--NYIRNFYEGCV-KPPTV  19
Loxodonta africana CXNT         1  -----------------------------------------M--SL--NYFKNFYEGCV-KPPTV  19
Homo sapiens CXNU               1  -----------------------------------------------------------MPASSL   6
Pan troglodytes CXNU            1  -----------------------------------------------------------MPASSL   6
Pongo abelii CXNU               -  -----------------------------------------------------------------   -
Nomascus leucogenys CXNU        1  -----------------------------------------------------------MPASSL   6
Macaca mulatta CXNU             1  -----------------------------------------------------------MPASSL   6
Mus musculus Cxnu               -  -----------------------------------------------------------------   -
Cavia porcellus CXNU            -  -----------------------------------------------------------------   -
Bos taurus CXNU                 -  -----------------------------------------------------------------   -
Equus caballus CXNU             -  -----------------------------------------------------------------   -
Myotis lucifugus CXNU           1  --------------------------------------------------------M-VVP-FSF   7
Sorex araneus CXNU              -  -----------------------------------------------------------------   -
Dasypus novemcinctus CXNU       1  --------------------------------------------MKC-IY-RAVMMS-FIPAASF  18
Loxodonta africana CXNU         1  --------------------------------------------MKH-LP-MAVMLS-LIPAPSC  18
```

```
Homo sapiens CXNA                20 FGRIWLSLVFIFRVLVYLVTAERVWS--DDHKDFDCNTRQPGCSNV-CFDEFFPVS-H--VR-LW  77
                                       200       210       220       230       240       250       260
Homo sapiens CXNA                20 FGRIWLSLVFIFRVLVYLVTAERVWS--DDHKDFDCNTRQPGCSNV-CFDEFFPVS-H--VR-LW  77
Pan troglodytes CXNA             20 FGRIWLSLVFIFRVLVYLVTAERVWS--DDHKDFDCNTRQPGCSNV-CFDEFFPVS-H--VR-LW  77
Gorilla gorilla CXNA             20 FGRIWLSLVFIFRVLVYLVTAERVWS--DDHKDFDCNTRQPGCSNV-CFDEFFPVS-H--VR-LW  77
Pongo abelii CXNA                20 FGRIWLSLVFIFRVLVYLVTAERVWS--DDHKDFDCNTRQPGCSNV-CFDEFFPVS-H--VR-LW  77
Nomascus leucogenys CXNA         20 FGRVWLSLVFIFRVLVYLVTAERVWS--DDHKDFDCNTHQPGCSNV-CFDEFFPVS-H--VR-LW  77
Macaca mulatta CXNA              20 FGRIWLSLVFIFRVLVYLVTAERVWS--DDHKDFDCNTRQPGCSNV-CFDEFFPVS-H--VR-LW  77
Callithrix jacchus CXNA          20 FGRIWLSLVFIFRVLVYLVTAERVWS--DDHKDFDCNTRQPGCSNV-CFDEFFPVS-H--VR-LW  77
Mus musculus Cxna                20 FGRIWLSLVFVFRVLVYLVTAERVWG--DDQKDFDCNTRQPGCTNV-CYDEFFPVS-H--VR-LW  77
Rattus norvegicus Cxna           20 FGRIWLSLVFVFRVLVYLVTAERVWG--DDQKDFDCNTRQPGCTNV-CYDEFFPVS-H--VR-LW  77
Cavia porcellus CXNA             70 FGRIWLSLVFIFRVLVYVVTAERVWS--DDHKDFDCNTHQPGCSNV-CFDQFFPVS-H--VR-LW 127
Ochotona princeps CXNA           20 FGRIWLSLVFIFRVLVYLVTAERVWS--DDHKDFDCNTRQPGCSNV-CFDEFFPVS-H--VR-LW  77
Bos taurus CXNA                  20 FGRVWLSLVFIFRVLVYLVTAERVWS--DDHKDFDCDTRQPGCSNV-CFDEFFPVS-H--VR-LW  77
Equus caballus CXNA              20 FGRIWLSLVFIFRVLVYLVTAEHVWS--DDHKDFDCNTRQPGCSNV-CFDEFFPVS-H--VR-LW  77
Canis lupus familiaris CXNA      20 FGRIWLSLVFIFRVLVYLVTAERVWS--DDHKDFDCNTRQPGCTNV-CFDQFFPVS-H--VR-LW  77
Felis catus CXNA                 20 FGRIWLSLVFVFRVLVYLVTAERVWS--DDHKDFDCNTKQPGCSNV-CFDEFFPVS-H--VR-LW  77
Myotis lucifugus CXNA            52 FGRIWLSLVFIFRVMVYLVTAERVWS--DDHKDFDCNTRQPGCSNV-CYDEFFPVS-H--VR-LW 109
Dasypus novemcinctus CXNA        20 FGRIWLSLVFIFRVLVYLVTVEHVWS--DDHKDFDCNTRQPGCTNV-CFDEFFPVS-H--VR-LW  77
Loxodonta africana CXNA          20 FGRIWLSLVFIFRIMVYLVTAERVWS--DDHKDFDCNTRQPGCSNV-CFDKFFPVS-H--VR-LW  77
Homo sapiens CXNB                20 LSRIWLSVVFIFRVLVYVVAAEEVWD--DEQKDFVCNTKQPGCPNV-CYDEFFPVS-H--VR-LW  77
Gorilla gorilla CXNB             20 LSRIWLSVVFIFRVLVYVVAAEEVWD--DEQKDFVCNTKQPGCPNV-CYDEFFPVS-H--VR-LW  77
Nomascus leucogenys CXNB         20 LSRIWLSVVFIFRVLVYVVAAEEVWD--DEQKDFVCNTKQPGCPNV-CYDEFFPVS-H--VR-LW  77
Macaca mulatta CXNB              20 LSRIWLSVVFIFRVLVYVVAAEEVWD--DEQKDFVCNTKQPGCPNV-CYDEFFPVS-H--VR-LW  77
Callithrix jacchus CXNB          20 LSRIWLSVVFIFRVLVYVVAAEEVWD--DEQKDFVCNTKQPGCPNV-CYDEFFPVS-H--VR-LW  77
Otolemur garnettii CXNB          20 VSRIWLSVVFIFRVLVFVVAAEDVWD--DEQKDFVCNTKQPGCPNV-CYDEFFPVS-H--VR-LW  77
Mus musculus Cxnb                20 LGRIWLSVVFIFRVLVYVVAAEEVWD--DDQKDFICNTKQPGCPNV-CYDEFFPVS-H--VR-LW  77
Rattus norvegicus Cxnb           20 LGRIWLSVVFIFRVLVYVVAAEEVWD--DEQKDFICNTKQPGCPNV-CYDEFFPVS-H--VR-LW  77
Dipodomys ordii CXNB             20 LGRIWLSVVFIFRVLVYVVAAEDVWD--DEQKDFICNTKQPGCPNV-CYDEFFPVS-H--VR-LW  77
Cavia porcellus CXNB             20 LSRIWLSVVFVFRLLVYLVAAKDVWN--EEQKDFVCNTLQPGCPNV-CYDHFFPVS-H--VR-LW  77
Oryctolagus cuniculus CXNB        - -----------------------------------------------------------------   -
Ochotona princeps CXNB           20 LGRIWLSVVFIFRVLVYVVAAEDVWD--DEQKDFDCNTRQPGCTNV-CYDEFFPVS-H--VR-LW  77
Bos taurus CXNB                  20 LGRIWLSVVFIFRVLVYVVAAEEVWD--DEQKDFVCNTKQPGCPNV-CYDQFFPVS-H--VR-LW  77
Equus caballus CXNB              20 LGRIWLSVVFIFRVLVYVVAAEEVWD--DEQKDFICNTKQPGCPNV-CYDEFFPVS-H--VR-LW  77
Canis lupus familiaris CXNB      70 LGRIWLSVVFLFRVLVYVVAAEEVWD--DEQKDFVCNTRQPGCPNV-CYDEFFPVS-H--VR-LW 127
Myotis lucifugus CXNB            20 LGRIWLSVVFIFRVLVFVVAAEDVWD--DEQKDFVCNTKQPGCPNV-CYDEFFPVS-H--VR-LW  77
Erinaceus europaeus CXNB         20 LSRIWLSVVFIFRVLVYVVAAEDVWD--DEQKDFICNTKQPGCTNV-CYDHFFPVS-H--VR-LW  77
Loxodonta africana CXNB          20 LGRIWLSVVFIFRLLVYVVAAEDVWH--DEQDDFVCNTKQPGCPNV-CYDEFFPVS-H--VR-LW  77
Homo sapiens CXNC                20 FGRIWLSVVFVFRVLVYVVAAERVWG--DEQKDFDCNTKQPGCTNV-CYDNYFPIS-N--IR-LW  77
Gorilla gorilla CXNC             20 FGRIWLSVVFVFRVLVYVVAAERVWG--DEQKDFDCNTRQPGCTNV-CYDNYFPIS-N--IR-LW  77
Pongo abelii CXNC                20 FGRIWLSVVFVFRVLVYVVAAERVWG--DEQKDFDCNTKQPGCTNV-CYDNYFPIS-N--IR-LW  77
Nomascus leucogenys CXNC         20 FGRIWLSVVFVFRVLVYVVAAERVWG--DEQKDFDCNTKQPGCTNV-CYDNYFPIS-N--IR-LW  77
Callithrix jacchus CXNC          20 FGRIWLSVVFVFRVLVYVVAAERVWG--DEQKDFDCNTKQPGCTNV-CYDNFFPIS-N--IR-LW  77
Microcebus murinus CXNC          50 FGRIWLSVVFVFRVLVYVVAAERVWG--DEQKDFDCNTRQPGCTNV-CYDEFFPIS-N--IR-LW 107
Otolemur garnettii CXNC          20 FGRIWLSVVFVFRVLVYVVAAERVWG--DEQKDFDCNTKQPGCTNV-CYDNFFPIS-N--IR-LW  77
Mus musculus Cxnc                20 FGRIWLSVVFVFRVLVYVVAAERVWG--DEQKDFDCNTRQPGCTNV-CYDNFFPIS-N--IR-LW  77
Rattus norvegicus Cxnc           20 FGRIWLSVVFVFRVLVYVVAAERVWG--DEQKDFDCNTRQPGCTNV-CYDNFFPIS-N--IR-LW  77
Cavia porcellus CXNC             20 FGRIWLSVVFVFRVLVYVVAAERVWG--DEQKDFDCNTRQPGCTNV-CYDDFFPIS-N--IR-LW  77
Oryctolagus cuniculus CXNC      130 FGRIWLSVVFVFRVLVYVVAAERVWG--DEQKDFDCNTKQPGCTNV-CYDNFFPIS-N--IR-LW 187
Bos taurus CXNC                  20 FGRIWLSVVFVFRVLVYVVAAERVWG--DEQKDFDCNTKQPGCTNV-CYDEFFPIS-N--IR-LW  77
Equus caballus CXNC              20 FGRIWLSVVFVFRVLVYVVAAERVWR--DEQKDFDCNTKQPGCTNV-CYDHFFPIS-N--IR-LW  77
Canis lupus familiaris CXNC      20 FGRIWLSVVFVFRVLVYVVAAERVWG--DEQKDFDCNTKQPGCTNV-CYDDFFPIS-N--IR-LW  77
Felis catus CXNC                 20 FGRIWLSVVFVFRVLVYVVAAERVWG--DEQKDFDCNTRQPGCTNV-CYDNFFPIS-N--IR-LW  77
Myotis lucifugus CXNC            20 FGRIWLSVVFVFRVLVYVVAAERVWG--DEQKDFDCNTKQPGCTNV-CYDNYFPIS-N--IR-LW  77
Dasypus novemcinctus CXNC        20 FGRIWLSVVFVFRVLVYVVAAERVWG--DEQKDFDCNTKQPGCTNV-CYDHFFPIS-N--IR-LW  77
Loxodonta africana CXNC          20 FGRIWLSVVFVFRLLVYVVAAERVWG--DEQKDFDCNTKQPGCTNV-CYDNFFPIS-N--IR-LW  77
Homo sapiens CXND                20 IGWIWLAVVFVFRLLVYMVAAEHVWK--DEQKEFECNSRQPGCKNV-CFDDFFPIS-Q--VR-LW  77
Pan troglodytes CXND             20 IGWIWLAVVFVFRLLVYMVAAEHVWK--DEQKEFECNSRQPGCKNV-CFDDFFPIS-Q--VR-LW  77
Macaca mulatta CXND              20 IGLIWLAVVFVFRLLVYMVATEHVWK--DEQKEFECNSRQPGCENV-CFDDFFPIS-Q--VR-LW  77
Callithrix jacchus CXND          20 NGQIWLAVVFVFCLLVYMVAAEHVWK--DEQKEFECNSRQPGCENV-CFDNFFPIS-Q--VR-LW  77
Dipodomys ordii CXND             20 IGRIWLAVVFIFRLLVYIVAAEHVWK--DEDKEFECNTRQPGCENV-CFDYFFPIS-Q--VR-LW  77
Oryctolagus cuniculus CXND       20 TGRLWLAVVFIFRLLVYIVAAEHVWK--DERREFECNVRQPGCETV-CFDHFFPIS-Q--VR-LW  77
Tursiops truncatus CXND          20 IGRIWLAVTFIFRLLVYMVAAEHVWK--DEQKEFECNVRQPGCENV-CFDYFFPIS-Q--VR-LW  77
Bos taurus CXND                   - -----------------------------------------------------------------   -
Equus caballus CXND              39 IGRIWLAVVFIFRLLVYMVAAEHVWK--DEQKEFECNIRQPGCENV-CFDYYFPVS-Q--VR-LW  96
Canis lupus familiaris CXND      20 IGRIWLAVVFIFRLLVYIVAAEHVWK--DEQKEFECNVKQPGCENV-CFDHFFPIS-Q--VR-LW  77
Myotis lucifugus CXND            20 IGRIWLAVVFIFRLLVYMVAAEHVWK--DEQKEFECNIRQPGCENM-CFDYFFPIS-Q--VR-LW  77
Erinaceus europaeus CXND         20 IGRIWLAVVFVFRLLVYIVAAEHVWK--DEQKEFECNIKQPGCENV-CFDYFFPIS-Q--VR-LW  77
Dasypus novemcinctus CXND        20 IGRIWLAVIFIFRLLVYMVAAEHVWK--DEQKEFECNIRQPGCENV-CFDYYFPIS-Q--VR-LW  77
Choloepus hoffmanni CXND         20 IGRIWLAVVFIFRLLVYMVAAKHVWK--DEQKEFECNIRQPGCENV-CFDYFFPIS-Q--VR-LW  77
Loxodonta africana CXND          20 IGRIWLAVVFIFRLLVYMVAAEYVWK--DEQKEFECNIRQPGCENV-CFDYFFPIS-Q--VR-LW  77
Homo sapiens CXNE                20 IGKIWLTVLFIFRIMILVVAAKEVWG--DEQADFVCNTLQPGCKNV-CYDHYFPIS-H--IR-LW  77
Pan troglodytes CXNE             20 IGKIWLTVLFIFRIMILVVAAKEVWG--DEQADFVCNTLQPGCKNV-CYDHYFPIS-H--IR-LW  77
Pongo abelii CXNE                20 IGKIWLTVLFIFRIMILVVAAKEVWG--DEQADFVCNTLQPGCKNV-CYDHYFPIS-H--IR-LW  77
Macaca mulatta CXNE              20 IGKIWLTVLFIFRIMILVVAAKEVWG--DEQADFVCNTLQPGCKNV-CYDHYFPIS-H--IR-LW  77
Papio hamadryas CXNE             20 IGKIWLTVLFIFRIMILVVAAKEVWG--DEQADFVCNTLQPGCKNV-CYDHYFPIS-H--IR-LW  77
Callithrix jacchus CXNE          20 IGKIWLTVLFIFRIMILVVAAKEVWG--DEQADFTCNTLQPGCKNV-CYDHYFPIS-H--IR-LW  77
Otolemur garnettii CXNE          20 IGKIWLTVLFIFRIMILVVAAKEVWG--DEQADFTCNTLQPGCKNV-CYDHYFPIS-H--IR-LW  77
Mus musculus Cxne                33 IGKIWLTVLFIFRIMILVVAAKEVWG--DEQADFVCNTLQPGCKNV-CYDHHFPIS-H--IR-LW  90
```

```
Rattus norvegicus Cxne           39 IGKIWLTVLFIFRIMILVVAAKEVWG--DEQADFVCNTLQPGCKNV-CYDHYFPIS-H--IR-LW  96
Dipodomys ordii CXNE             39 IGKIWLTVLFIFRIMILVVAAKEVWG--DEQADFICNTLQPGCKNV-CYDHHFPIS-H--IR-LW  96
Cavia porcellus CXNE             67 IGKIWLTVLFIFRVMILVVATKEVWG--DEQADFTCNTLQPGCKNV-CYDHYFPIS-H--IR-LW 124
Oryctolagus cuniculus CXNE       39 IGKIWLTVLFIFRIMILVVAAKEVWG--DEQADFTCNTLQPGCKNV-CYDHYFPIS-H--IR-LW  96
Equus caballus CXNE              20 IGKIWLTVLFIFRIMILVVAAKEVWG--DEQADFVCNTLQPGCKNV-CYDHYFPIS-H--IR-LW  77
Canis lupus familiaris CXNE      20 IGKIWLTVLFIFRIMILVVAAKEVWG--DEQADFVCNTLQPGCKNV-CYDHYFPIS-H--IR-LW  77
Felis catus CXNE                 20 IGKIWLTVLFIFRIMILVVAAKEVWG--DEQADFVCNTLQPGCKNV-CYDHYFPIS-H--IR-LW  77
Myotis lucifugus CXNE            20 IGKIWLTVLFIFRIMILVVAAKEVWG--DEQADFICNTLQPGCKNV-CYDHYFPIS-H--IR-LW  77
Sorex araneus CXNE               20 IGKIWLTVLFIFRIMILVVAAKEVWG--DEQADFTCNTLQPGCKNV-CYDHYFPIS-H--IR-LW  77
Dasypus novemcinctus CXNE        20 IGKIWLTVLFIFRIMILVVAAKEVWG--DEQADFICNTLQPGCKNV-CYDHYFPIS-H--IR-LW  77
Loxodonta africana CXNE          20 IGKIWLTVLFIFRIMILVVAAKEVWG--DEQADFICNTLQPGCKNV-CYDHYFPIS-H--IR-LW  77
Homo sapiens CXNF                20 IGKVWITVIFIFRVMILVVAAQEVWG--DEQEDFVCNTLQPGCKNV-CYDHFFPVS-H--IR-LW  77
Pan troglodytes CXNF             20 IGKVWITVIFIFRVMILVVAAQEVWG--DEQEDFVCNTLQPGCKNV-CYDHFFPVS-H--IR-LW  77
Gorilla gorilla CXNF             20 IGKVWITVIFIFRVMILVVAAQEVWG--DEQEDFVCNTLQPGCKNV-CYDHFFPVS-H--IR-LW  77
Nomascus leucogenys CXNF         20 IGKVWITVIFIFRVMILVVAAQEVWG--DEQEDFVCNTLQPGCKNV-CYDHFFPVS-H--IR-LW  77
Callithrix jacchus CXNF          20 IGKVWITVIFIFRVMILVVAAHEVWG--DEQEDFVCNTLQPGCKNV-CYDHFFPVS-H--IR-LW  77
Mus musculus Cxnf                20 IGKVWITVIFIFRVMILVVAAQEVWG--DEQEDFVCNTLQPGCKNV-CYDHFFPVS-H--IR-LW  77
Rattus norvegicus Cxnf           20 IGKVWITVIFIFRVMILVVAAQEVWG--DEQEDFVCNTLQPGCKNV-CYDHFFPVS-H--IR-LW  77
S.tridecemlineatus CXNF          20 IGKVWITVIFIFRVMILVVAAQEVWG--DEQEDFVCNTLQPGCKNV-CYDHFFPIS-H--IR-LW  77
Oryctolagus cuniculus CXNF       20 IGKVWITVIFIFRVMILVVAAQEVWG--DEQEDFVCNTLQPGCKNV-CYDHFFPVS-H--IR-LW  77
Ochotona princeps CXNF           20 IGKVWITVLFIFRVMILVVAAQEVWG--DEQEDFVCNTLQPGCKNV-CYDHYFPIS-H--IR-LW  77
Bos taurus CXNF                  20 IGKVWVTVLFVFRVMILVVAAQEVWG--DEQEDFVCNTLQPGCRNV-CYDHFFPVS-H--IR-LW  77
Equus caballus CXNF              20 IGKVWITVIFIFRVMILVVAAQEVWG--DEQEDFVCNTLQPGCKNV-CYDHFFPVS-H--IR-LW  77
Canis lupus familiaris CXNF      20 IGKVWITVIFIFRVMILVVAAQEVWG--DEQEDFVCNTLQPGCKNV-CYDHFFPVS-H--IR-LW  77
Myotis lucifugus CXNF            20 IGKVWITVIFIFRVMILVVAAQEVWG--DEQEDFVCNTLQPGCRNV-CYDHFFPVS-H--IR-LW  77
Dasypus novemcinctus CXNF        20 IGKVWVTVIFIFRVMILVVAAQEVWG--DEQEDFVCNTLQPGCKNV-CYDHFFPVS-H--IR-LW  77
Choloepus hoffmanni CXNF         20 IGKVWITVIFIFRVMILVVAAQEVWG--DEQADFVCNTLQPGCKNV-CYDHFFPVS-H--IR-LW  77
Loxodonta africana CXNF          20 IGKVWITVIFIFRVMILVVAAHEVWG--DEQEDFVCNTLQPGCKNV-CYDHFFPVS-H--IR-LW  77
Homo sapiens CXNG                20 IGRVWLSVIFIFRIMVLVVAAESVWG--DEKSSFICNTLQPGCNSV-CYDQFFPIS-H--VR-LW  77
Pan troglodytes CXNG             20 IGRVWLSVIFIFRIMVLVVAAESVWG--DEKSSFICNTLQPGCNSV-CYDQFFPIS-H--VR-LW  77
Gorilla gorilla CXNG             20 IGRVWLSVIFIFRIMVLVVAAESVWG--DEKSSFICNTLQPGCNSV-CYDQFFPIS-H--VR-LW  77
Pongo abelii CXNG                20 IGRVWLSVIFIFRIMVLVVAAESVWG--DEKSSFICNTLQPGCNSV-CYDQFFPIS-H--VR-LW  77
Nomascus leucogenys CXNG         20 IGRVWLSVIFIFRIMVLVVAAESVWG--DEKSSFICNTLQPGCNSV-CYDQFFPIS-H--VR-LW  77
Macaca mulatta CXNG              20 IGRVWLSVIFIFRIMVLVVAAESVWG--DEKSSFICNTLQPGCNSV-CYDQFFPIS-H--VR-LW  77
Papio hamadryas CXNG             20 IGRVWLSVIFIFRIMVLVVAAESVWG--DEKSSFICNTLQPGCNSV-CYDQFFPIS-H--VR-LW  77
Callithrix jacchus CXNG          20 IGRVWLSVIFIFRIMVLVVAAESVWG--DEKSSFICNTLQPGCNSV-CYDQFFPIS-H--VR-LW  77
Microcebus murinus CXNG          20 IGRVWLSVIFIFRIMVLVVAAESVWG--DEKSSFICNTLQPGCNSV-CYDHFFPIS-H--VR-LW  77
Mus musculus Cxng                20 IGRVWLSVIFIFRIMVLVVAAESVWG--DEKSSFICNTLQPGCNSV-CYDHFFPIS-H--VR-LW  77
Rattus norvegicus Cxng           20 IGRVWLSVIFIFRIMVLVVAAESVWG--DEKSSFICNTLQPGCNSV-CYDHFFPIS-H--VR-LW  77
Oryctolagus cuniculus CXNG       20 IGRVWLSVIFIFRIMVLVVAAESVWG--DEKSSFICNTLQPGCNSV-CYDHFFPIS-H--VR-LW  77
Bos taurus CXNG                  20 IGRVWLSVIFIFRIMVLVVAAESVWG--DEKSSFICNTLQPGCNSV-CYDHFFPIS-H--VR-LW  77
Equus caballus CXNG              20 IGRVWLSVIFIFRIMVLVVAAESVWG--DEKSSFICNTLQPGCNSV-CYDHFFPIS-H--VR-LW  77
Canis lupus familiaris CXNG      20 IGRVWLSVIFIFRIMVLVVAAESVWG--DEKSSFICNTLQPGCNSV-CYDHFFPIS-H--VR-LW  77
Felis catus CXNG                 20 IGRVWLSVIFIFRIMVLVVAAESVWG--DEKSSFICNTLQPGCNSV-CYDHFFPIS-H--VR-LW  77
Myotis lucifugus CXNG            20 IGRVWLSVIFIFRIMVLVVAAESVWG--DEKSSFICNTLQPGCNSV-CYDHFFPIS-H--VR-LW  77
Pteropus vampyrus CXNG           20 IGRVWLSVIFIFRIMVLVVAAESVWG--DEKSSFICNTLQPGCNSV-CYDEFFPIS-H--VR-LW  77
Dasypus novemcinctus CXNG        20 IGRVWLSVIFIFRIMVLVVAAESVWG--DEKSSFICNTLQPGCNSV-CYDHFFPIS-H--VR-LW  77
Loxodonta africana CXNG          20 IGRVWLSVIFIFRIMVLVVAAESVWG--DEKSSFICNTLQPGCNSV-CYDHFFPIS-H--VR-LW  77
Procavia capensis CXNG           20 IGRVWLSVIFIFRIMVLVVAAESVWG--DEKSSFICNTLQPGCNSV-CYDHFFPIS-H--VR-LW  77
Homo sapiens CXNH1               21 VGKIWLTVLFIFRILILGLAGESVWG--DEQSDFECNTAQPGCTNV-CYDQAFPIS-H--IR-YW  78
Pan troglodytes CXNH1            21 VGKIWLTVLFIFRILILGLAGESVWG--DEQSDFECNTAQPGCTNV-CYDQAFPIS-H--IR-YW  78
Gorilla gorilla CXNH1            21 VGKIWLTVLFIFRILILGLAGESVWG--DEQSDFECNTAQPGCTNV-CYDQAFPIS-H--IR-YW  78
Pongo abelii CXNH1               21 VGKIWLTVLFIFRILILGLAGESVWG--DEQSDFECNTAQPGCTNV-CYDQAFPIS-H--IR-YW  78
Nomascus leucogenys CXNH1        21 VGKIWLTVLFIFRILILGLAGESVWG--DEQSDFECNTAQPGCTNV-CYDQAFPIS-H--IR-YW  78
Macaca mulatta CXNH1             21 VGKIWLTVLFIFRILILGLAGESVWG--DEQSDFECNTAQPGCTNV-CYDQAFPIS-H--IR-YW  78
Otolemur garnettii CXNH1         33 VGRIHLTVL-IIRILIFALATNWVWG--DEQLDFKCSMTEPGCTNV-CYNQAFPIS-P--IR-YW  89
Mus musculus Cxnh1               21 VGKIWLTVLFIFRILILGLAGESVWG--DEQSDFECNTAQPGCTNV-CYDQAFPIS-H--IR-YW  78
Rattus norvegicus Cxnh1          21 VGKIWLTVLFIFRILILGLAGESVWG--DEQSDFECNTAQPGCTNV-CYDQAFPIS-H--IR-YW  78
Cavia porcellus CXNH1            21 VGKIWLTVLFIFRILILGLAGESVWG--DEQSDFECNTAQPGCANV-CYDQAFPIS-H--IR-YW  78
Oryctolagus cuniculus CXNH1      21 VGKIWLTVLFIFRILILGLAGESVWG--DEQSDFECNTAQPGCTNV-CYDLAFPIS-H--IR-YW  78
Ochotona princeps CXNH1          35 VGKIWLTVLFIFRILILGLAGESVWG--DEQSDFECNTAQPGCTNV-CYDQAFPIS-H--IR-YW  92
Equus caballus CXNH1             21 VGKIWLTVLFIFRFLVLSTAGELVWD--DEQSDFECNTAQPGCTNV-CYDQAFPIS-H--IR-YW  78
Canis lupus familiaris CXNH1     21 VGKIWLTVLFIFRILILGLAGESVWG--DEQSDFECNTAQPGCTNV-CYDQAFPIS-H--IR-YW  78
Felis catus CXNH1                21 VGKIWLTVLFIFRILILGLAGESVWG--DEQSDFECNTAQPGCTNV-CYDQAFPIS-H--IR-YW  78
Myotis lucifugus CXNH1           53 VGKIWLTVLFIFRILILGLAGESVWG--DEQSDFQCNTAQPGCPNV-CYDQAFPIS-H--IR-YW 110
Dasypus novemcinctus CXNH1       21 VGKIWLTVLFIFRILILGLAGESVWG--DEQSDFVCNTAQPGCTNV-CYDQAFPIS-H--IR-YW  78
Loxodonta africana CXNH1         21 VGKIWLTVLFIFRILILGLAGESVWG--DEQSDFECNTAQPGCTNV-CYDQAFPIS-H--IR-YW  78
Homo sapiens CXNI                21 VGKVWLTVLFIFRMLVLGTAAESSWG--DEQADFRCDTIQPGCQNV-CYDQAFPIS-H--IR-YW  78
Pan troglodytes CXNI             21 VGKVWLTVLFIFRMLVLGTAAESSWG--DEQADFRCDTIQPGCQNV-CYDQAFPIS-H--IR-YW  78
Pongo abelii CXNI                21 VGKVWLTVLFIFRMLVLGTAAESSWG--DEQADFRCDTIQPGCQNV-CYDQAFPIS-H--IR-YW  78
Nomascus leucogenys CXNI         21 VGKVWLTVLFIFRMLVLGTAAESSWG--DEQADFRCDTIQPGCQNV-CYDQAFPIS-H--IR-YW  78
Macaca mulatta CXNI              21 VGKVWLTVLFIFRMLVLGTAAESSWG--DEQADFRCDTIQPGCQNV-CYDQAFPIS-H--IR-YW  78
Papio hamadryas CXNI             21 VGKVWLTVLFIFRMLVLGTAAESSWG--DEQADFRCDTIQPGCQNV-CYDQAFPIS-H--IR-YW  78
Callithrix jacchus CXNI          21 VGKVWLTVLFIFRMLVLGTAAESSWG--DEQADFRCDTIQPGCQNV-CYDQAFPIS-H--IR-FW  78
Otolemur garnettii CXNI          21 IGKVWLTVLFIFRMLVLGTAAESSWG--DEQADFQCDTIQPGCENV-CYDQAFPIS-H--IR-YW  78
Mus musculus Cxni                21 IGKVWLTVLFIFRMLVLGTAAESSWG--DEQADFRCDTIQPGCQNV-CYDQAFPIS-H--IR-YW  78
Rattus norvegicus Cxni           55 IGKVWLTVLFIFRMLVLGTAAESSWG--DEQADFRCDTIQPGCQNV-CYDQAFPIS-H--IR-YW 112
Cavia porcellus CXNI             21 IGKVWLTILFIFRMLVLGTAAESSWG--DEQADFQCDTMQPGCENV-CYDQAFPIS-H--IR-YW  78
Oryctolagus cuniculus CXNI       21 IGKVWLTVLFIFRMLVLGTAAESSWG--DEQSDFQCDTIQPGCENV-CYDQAFPIS-H--IR-YW  78
```

Bos taurus CXNI	21	IGKVWLTVLFIFRMLVLGTAAESSWG--DEQADFLCDTMQPGCENV-CYDQAFPIS-H--IR-YW	78
Equus caballus CXNI	21	IGKVWLTVLFIFRILVLVTAAESSWG--DEQADFLCDTLQPGCENV-CYDQAFPIS-H--IR-YW	78
Canis lupus familiaris CXNI	21	IGKVWLTVLFIFRMLVLGTAAESSWG--DEQADFQCDTMQPGCGNV-CYDQAFPIS-H--IR-YW	78
Felis catus CXNI	21	IGKVWLTVLFIFRMLVLGTAAESSWG--DEQADFQCDTQQPGCENV-CYDQAFPIS-H--IR-YW	78
Pteropus vampyrus CXNI	21	IGKVWLTVLFIFRMLVLGTAAESSWG--DEQADFQCDTIQPGCENV-CYDQAFPIS-H--IR-YW	78
Erinaceus europaeus CXNI	21	IGKVWLTVLFIFRMLVLGTAAESSWG--DEQADFKCDTIQPGCENV-CYDQAFPIS-H--IR-FW	78
Dasypus novemcinctus CXNI	21	IGKVWLTVLFIFRMLVLGTAAESSWG--DEQADFQCDTTQPGCENV-CYDQAFPIS-H--IR-YW	78
Loxodonta africana CXNI	21	IGKVWLTVLFIFRMLVLGTAAESSWG--DEQADFQCDTLQPGCENV-CYDQAFPIS-H--IR-YW	78
Homo sapiens CXNJ1	21	IGKVWLTVLFIFRILVLGAAAEDVWG--DEQSDFTCNTQQPGCENV-CYDRAFPIS-H--IR-FW	78
Pongo abelii CXNJ1	21	IGKVWLTVLFIFRILVLGAAAEDVWG--DEQSDFTCNTQQPGCENV-CYDRAFPIS-H--IR-FW	78
Macaca mulatta CXNJ1	21	IGKVWLTVLFIFRILVLGAAAEDVWG--DEQSDFTCNTQQPGCENV-CYDRAFPIS-H--IR-FW	78
Mus musculus Cxnj1	21	IGKVWLTVLFIFRILVLGAAAEEVWG--DEQSDFTCNTQQPGCENV-CYDRAFPIS-H--IR-FW	78
Rattus norvegicus Cxnj1	21	IGKVWLTVLFIFRILVLGAAAEEVWG--DEQSDFTCNTQQPGCENV-CYDRAFPIS-H--IR-FW	78
Bos taurus CXNJ1	21	IGKVWLTVLFIFRILVLGAAAEEVWG--DEQSDFTCNTQQPGCENV-CYDRAFPIS-H--VR-FW	78
Equus caballus CXNJ1	21	IGKVWLTVLFIFRILVLGAAAEEVWG--DEQSDFTCNTQQPGCENV-CYDRAFPIS-H--VR-FW	78
Myotis lucifugus CXNJ1	21	IGKVWLTVLFIFRILLLGAGVESVWG--DEQSGFTCNTQQPGCENV-CYDKAFPIS-H--TR-FW	78
Myotis lucifugus CXNJ2	21	IGKVWLTVLFIFRILVLGAAVEEVWG--DEQSDFTCNTQQPGCENV-CYDKAFPIS-H--TR-FW	78
Pteropus vampyrus CXNJ1	190	IGKVWLTVLFIFRILVLGAAAEEVWG--DEQSDFTCNTQQPGCENV-CYDKAFPIS-H--IR-FW	247
Pteropus vampyrus CXNJ2	112	TGKVWLTVLFIFRILVLGAGVESVWG--DEQSDFTCNTQQPGCENV-CYDKAFPIS-H--TR-FW	169
Sorex araneus CXNJ1	21	VGKVWLTVLFVFRILVLGAAVEAVWG--DEQSDFTCNTQQPGCENV-CYDQAFPIS-H--VR-FW	78
Homo sapiens CXNK1	21	GGKVWLSVLFIFQILLLKTVVESAWG--DEQSAFRCNTQQPGCENV-CYDKSFPIS-H--VR-FW	78
Homo sapiens CXNK2	21	GGKVWLSVLFIFRILLLGTAVESAWG--DEQSAFRCNTQQPGCENV-CYDKSFPIS-H--VR-FW	78
Pan troglodytes CXNK1	21	GGKVWLSVLFIFQILLLKTVVESAWG--DEQSAFRCNTQQPGCENV-CYDKSFPIS-H--VR-FW	78
Pan troglodytes CXNK2	21	GGKVWLSVLFIFRILLLGTAVESAWG--DEQSAFRCNTQQPGCENV-CYDKSFPIS-H--VR-FW	78
Pongo abelii CXNK1	21	GGKVWLSVLFIFRILLLGTAVESAWG--DEQSAFRCNTQQPGCENV-CYDKSFPIS-H--VR-FW	78
Nomascus leucogenys CXNK1	21	GGKVWLSVLFIFRILLLGTAVESAWG--DEQSAFRCNTQQPGCENV-CYDKSFPIS-H--VR-FW	78
Callithrix jacchus CXNK1	21	GGKVWLSVLFIFRILLLGTAVESAWG--DEQSAFRCNTQQPGCENV-CYDKSFPIS-H--VR-FW	78
Mus musculus Cxnk1	21	GGKVWLSVLFIFRILLLGTAVESAWG--DEQSAFRCNTQQPGCENV-CYDKSFPIS-H--VR-FW	78
Mus musculus Cxnk2	21	GGKVWIKVLFIFRILLLGTAIESAWS--DEQFEFHCNTQQPGCENV-CYDHAFPIS-H--VR-LW	78
Rattus norvegicus Cxnk1	21	GGKVWLSVLFIFRILLLGTAVESAWG--DEQSAFRCNTQQPGCENV-CYDKSFPIS-H--VR-FW	78
Rattus norvegicus Cxnk2	21	GGKVWIKVLFIFRILLLGTAIESAWS--DEQFEFHCNTQQPGCENV-CYDQAFPIS-H--VR-LW	78
Cavia porcellus CXNK1	21	GGKVWLSVLFIFRILLLGTAVESAWG--DEQSAFRCNTQQPGCENV-CYDKSFPIS-H--VR-FW	78
Oryctolagus cuniculus CXNK1	21	GGKVWLSVLFIFRILLLGTAVESAWG--DEQSAFRCNTQQPGCENV-CYDKSFPIS-H--VR-FW	78
Bos taurus CXNK1	21	GGKVWLSVLFIFRILLLGTAVESAWG--DEQSAFRCNTQQPGCENV-CYDKSFPIS-H--VR-FW	78
Vicugna pacos CXNK1	21	GGKVWLSVLFIFRILLLGTAVESAWG--DEQSAFRCNTQQPGCENV-CYDKSFPIS-H--VR-FW	78
Equus caballus CXNK1	21	GGKVWLSVLFIFRILLLGTAVESAWG--DEQSAFRCNTQQPGCENV-CYDKSFPIS-H--VR-FW	78
Equus caballus CXNK2	2	----WLSVIFIFRILLLGTAVESAWD--DEQFGFRCNTRQPGCENV-CYDKSFPIS-H--VC-FW	55
Canis lupus familiaris CXNK1	21	GGKVWLSVLFIFRILLLGTAVESAWG--DEQSAFRCNTQQPGCENV-CYDKSFPIS-H--VR-FW	78
Canis lupus familiaris CXNK2	21	GGKVWLSVLFIFRILLLETAVESAWD--DEQFAFHCNTRQPGCENV-CYDKSFPIS-H--VH-IW	78
Felis catus CXNK1	21	GGKVWLSILFVFRILLLGTAVESAWD--DEQFAFRCNTRQPGCENV-CYDKSFPIS-H--VR-FW	78
Myotis lucifugus CXNK1	21	GGKVWLSVLFIFRILLLGTAVESAWG--DEQSAFRCNTQQPGCENV-CYDKSFPIS-H--VR-FW	78
Dasypus novemcinctus CXNK1	21	GVKVWLSVLFIFRILLLGIAVESAWD--DEQFAFRCNTRQPGCENV-CYDKSFPIS-H--VR-FW	78
Dasypus novemcinctus CXNK2	21	GGKVWLSVLFIFRILLLGTAVESAWG--DEQSAFRCNTQQPGCENV-CYDKSFPIS-H--VR-FW	78
Loxodonta africana CXNK1	21	GGKVWLSVLFIFRILLLGTAVESAWG--DEQSAFRCNTQQPGCENV-CYDKSFPIS-H--VR-FW	78
Loxodonta africana CXNK2	21	GGKVWLSVLFIFRILVLGTAVESAWD--DEQFAFRCNTRQPGCENV-CYDKSFPIS-H--VR-FW	78
Homo sapiens CXNL	21	IGRVWLTVLFIFRILILGTAAEFVWG--DEQSDFVCNTQQPGCENV-CYDEAFPIS-H--IR-LW	78
Pan troglodytes CXNL	21	IGRVWLTVLFIFRILILGTAAEFVWG--DEQSDFVCNTQQPGCENV-CYDEAFPIS-H--IR-LW	78
Pongo abelii CXNL	21	IGRVWLTVLFIFRILILGTAAEFVWG--DEQSDFVCNTQQPGCENV-CYDEAFPIS-H--IR-LW	78
Nomascus leucogenys CXNL	21	IGRVWLTVLFIFRILILGTAAELVWG--DEQSDFVCNTQQPGCENV-CYDEAFPIS-H--IR-LW	78
Macaca mulatta CXNL	21	IGRVWLTVLFIFRILILGTAAEFVWG--DEQSDFVCNTQQPGCENV-CYDEAFPIS-H--IR-LW	78
Callithrix jacchus CXNL	21	IGRVWLTVLFIFRILILGTAAEFVWG--DEQSDFVCNTQQPGCENV-CYDEAFPIS-H--IR-LW	78
Otolemur garnettii CXNL	66	IGRVWLTVLFIFRILILGTAAEFVWG--DEQSDFVCNTQQPGCENV-CYDEAFPIS-H--IR-LW	123
Mus musculus Cxnl	21	IGRVWLTVLFIFRILILGTAAEFVWG--DEQSDFVCNTQQPGCENV-CYDEAFPIS-H--IR-LW	78
Rattus norvegicus Cxnl	21	IGRVWLTVLFIFRILILGTAAEFVWG--DEQSDFVCNTQQPGCENV-CYDEAFPIS-H--IR-LW	78
Cavia porcellus CXNL	21	IGRVWLTVLFIFRILILGTAAEFVWG--DEQSDFVCNTQQPGCENV-CYDEAFPIS-H--IR-LW	78
Oryctolagus cuniculus CXNL	21	IGRVWLTVLFIFRILILGTAAEFVWG--DEQSDFVCNTQQPGCENV-CYDEAFPIS-H--IR-LW	78
Ochotona princeps CXNL	21	IGRVWLTVLFIFRILILGTAAEFVWG--DEQSDFVCNTQQPGCENV-CYDEAFPIS-H--IR-LW	78
Bos taurus CXNL	21	IGRVWLTVLFIFRILILGTAAEFVWG--DEQSDFVCNTQQPGCENV-CYDEAFPIS-H--IR-LW	78
Equus caballus CXNL	21	IGRVWLTVLFIFRILILGTAAEFVWG--DEQSDFVCNTQQPGCENV-CYDEAFPIS-H--IR-LW	78
Canis lupus familiaris CXNL	21	IGRVWLTVLFIFRILILGTAAEFVWG--DEQSDFVCNTQQPGCENV-CYDEAFPIS-H--IR-LW	78
Felis catus CXNL	21	IGRVWLTVLFIFRILILGTAAEFVWG--DEQSDFVCNTQQPGCENV-CYDEAFPIS-H--IR-LW	78
Pteropus vampyrus CXNL	21	IGRVWLTVLFIFRILILGTAAEFVWG--DEQSDFVCNTQQPGCENV-CYDEAFPIS-H--IR-LW	78
Sorex araneus CXNL	21	VGRVWLSVLFVFRILILGTAAELVWG--DEQADFVCNTQQPGCENV-CYDAAFPIS-H--VR-LW	78
Dasypus novemcinctus CXNL	21	IGRVWLTVLFIFRILILGTAAEFVWG--DEQSDFVCNTQQPGCENV-CYDEAFPIS-H--IR-LW	78
Loxodonta africana CXNL	21	IGRVWLTVLFIFRILILGTAAEYVWG--DEQSDFVCNTQQPGCENV-CYDEAFPIS-L--IR-LW	78
Homo sapiens CXNM	21	IGKIWLTILFIFRMLVLGVAAEDVWN--DEQSGFICNTEQPGCRNV-CYDQAFPIS-L--IR-YW	78
Pan troglodytes CXNM	21	IGKIWLTILFIFRMLVLGVAAEDVWN--DEQSGFICNTEQPGCRNV-CYDQAFPIS-L--IR-YW	78
Pongo abelii CXNM	21	IGKIWLTILFIFRMLVLGVAAEDVWN--DEQSGFICNTEQPGCRNV-CYDQAFPIS-L--IR-YW	78
Nomascus leucogenys CXNM	21	IGKIWLTILFIFRMLVLGVAAEDVWN--DEQSGFICNTEQPGCRNV-CYDQAFPIS-L--IR-YW	78
Macaca mulatta CXNM	21	IGKIWLTILFIFRMLVLGVAAEDVWN--DEQSGFVCNTEQPGCRNV-CYDQAFPIS-L--IR-YW	78
Callithrix jacchus CXNM	21	IGKIWLTILFIFRMLVLGVAAEDVWN--DEQSGFICNTEQPGCRNV-CYDQAFPIS-L--IR-YW	78
Tarsius syrichta CXNM	21	IGKIWLTILFIFRMLVLGVAAEDVWN--DEQSDFICNTEQPGCRNV-CYDQAFPIS-L--IR-YW	78
Microcebus murinus CXNM	21	IGKIWLTILFIFRMLVLGVAAEDVWN--DEQSGFICNTEQPGCRNV-CYDQAFPIS-L--IR-YW	78
Dipodomys ordii CXNM	21	IGKIWLTILFIFRMLVLGIAAEDVWN--DEQSDFICNTEQPGCRNV-CYDQAFPIS-L--IR-YW	78
Oryctolagus cuniculus CXNM	21	VSKLWLTILFIFRMLVLGIAAGEIWE--DEQSGFICNTEQPGCRNA-CYDQAFPIS-L--IR-YW	78
Equus caballus CXNM	21	IGKIWLTILFVFRMLVLGVAAEDVWN--DEQSGFICNTEQPGCRNV-CYDQAFPIS-L--IR-YW	78
Canis lupus familiaris CXNM	21	IGKIWLTVLFIFRMLVLGVAAEDVWN--DEQSGFICNTEQPGCRNV-CYDQAFPIS-L--VR-YW	78
Pteropus vampyrus CXNM	21	IGKIWLTILFIFRMLVLGVAAENVWN--DEQSGFICNTEQPGCRNV-CYDQAFPIS-L--IR-YW	78
Loxodonta africana CXNM	21	IGKIWLTILFIFRMLVLGVAAEDVWN--DEQSGFICNTEQPGCRNV-CYDQAFPIS-L--IR-YW	78

```
Homo sapiens CXNN                 21 VGKIWLTILFIFRMLVLRVAAEDVWD--DEQSAFACNTRQPGCNNI-CYDDAFPIS-L--IR-FW  78
Pan troglodytes CXNN              21 VGKIWLTILFIFRMLVLRVAAEDVWD--DEQSAFACNTRQPGCNNI-CYDDAFPIS-L--IR-FW  78
Pongo abelii CXNN                 21 VGKIWLTILFIFRMLVLRVAAEDVWD--DEQSAFACNTRQPGCNNI-CYDDAFPIS-L--IR-FW  78
Nomascus leucogenys CXNN          21 VGKIWLTILFIFRMLVLRVAAEDVWD--DEQSAFACNTRQPGCNNI-CYDDAFPIS-L--IR-FW  78
Macaca mulatta CXNN               21 VGKIWLTILFIFRMLVLRVAAEDVWD--DEQSAFDCNTRQPGCNNI-CYDDAFPIS-L--IR-FW  78
Callithrix jacchus CXNN           21 VGKIWLTILFIFRMLVLRVAAEDVWD--DEQSAFACNTRQPGCNNI-CYDDAFPIS-L--IR-FW  78
Microcebus murinus CXNN           21 VGKIWLTILFIFRMLVLRVAAEGVWD--DEQSAFACNTQQPGCRNI-CYDDAFPIS-L--IR-FW  78
Mus musculus Cxnn                 21 VGKIWLTILFIFRMLVLGVAAEDVWD--DEQSAFACNTQQPGCNNI-CYDDAFPIS-L--IR-FW  78
Rattus norvegicus Cxnn            21 VGKIWLTILFIFRMLVLGVAAEDVWD--DEQSSFACNTQQPGCSNI-CYDDAFPIS-L--IR-FW  78
Oryctolagus cuniculus CXNN        51 VGKIWLTILFIFRMLVLGVAAEDVWD--DEQSAFTCNTQQPGCNNI-CYDDAFPIS-L--IR-FW 108
Bos taurus CXNN                   21 VGKIWLTILFIFRMLILGVAAEDVWD--DEQSAFACNTQQPGCNTV-CYDTAFPIS-L--IR-FW  78
Equus caballus CXNN               21 VGKIWLTILFIFRMLVLGVAAEDVWD--DEQSAFACNTQQPGCNNI-CYDDAFPIS-L--IR-FW  78
Canis lupus familiaris CXNN       21 VGKIWLTILFIFRMLVLGVAAEDVWD--DEQSAFACNTQQPGCNNI-CYDDAFPIS-L--IR-FW  78
Sorex araneus CXNN                21 VGKIWLTILFIFRMLVLGVAAEDVWD--DEQSAFVCNTQQPGCSNV-CYDDAFPIS-L--IR-FW  78
Loxodonta africana CXNN           21 VGKIWLTILFIFRMLVLGVAAEGVWD--DEQSAFACNTQQPGCNNI-CYDDAFPIS-L--IR-FW  78
Homo sapiens CXNO                 23 VGKVWLTVLVVFRIVLTAVGGEAIYS--DEQAKFTCNTRQPGCDNV-CYDAFAPLS-H--VR-FW  80
Papio hamadryas CXNO              23 VGKVWLTVLVVFRIVLTAVGGEAIYS--DEQAKFTCNTRQPGCDNV-CYDAFAPLS-H--VR-FW  80
Mus musculus Cxno                 34 VGKVWLTVLVVFRIVLTAVGGESIYS--DEQSKFTCNTRQPGCDNV-CYDAFAPLS-H--VR-FW  91
Cavia porcellus CXNO              37 VGKVWLTVLVVFRIVLTAVGGESIYS--DEQAKFTCNTRQPGCDNV-CYDAFAPLS-H--VR-FW  94
Homo sapiens CXNP1                20 VGRLLLPVLLGFRLVLLAASGPGVYG--DEQSEFVCHTQQPGCKAA-CFDAFHPLS-P--LR-FW  77
Pan troglodytes CXNP1             20 VGRLLLPVLLGFRLVLLAASGPGVYG--DEQSEFVCHTQQPGCKAA-CFDAFHPLS-P--LR-FW  77
Pongo abelii CXNP1                20 VGRLLLPVLLGFRLVLLAAGGPGVYG--DEQSEFVCHTQQPGCKAA-CFDAFQPLS-P--LR-FW  77
Callithrix jacchus CXNP1          20 VGRLLLPVLLGFRLVLLAASGPGVYG--DEQSEFVCHTQQPGCKAA-CFDSFQPLS-P--LR-FW  77
Otolemur garnettii CXNP1          20 VGRLLLPVLLGFRLVLLAASGPGIYG--DEQSEFVCHTQQAGCKAA-CFDALHPLS-P--LR-FW  77
Tupaia belangeri CXNP1            38 VGHLLLPVFLGFRLVLLAACGPGVYG--DEQSEFVCNTQQPGCKAA-CYDAFHPLS-P--LR-FW  95
Mus musculus Cxnp1                20 VGRFLLPMLMGFRLLILVSSGPGVFG--NDENEFICHLGQPGCKTI-CYDVFRPLS-P--LR-FW  77
Rattus norvegicus Cxnp1           20 VGRFLLPVLVGFRLLILVSSGPGVFG--NDENEFMCHLGQPGCKTI-CYDVFRPLS-P--LR-FW  77
Cavia porcellus CXNP1             20 VGRLLLPVLLGFRLALLAASGPGIYS--DDQSEFVCHTQQSGCKAA-CYDASHPLS-P--LR-FW  77
Oryctolagus cuniculus CXNP1       20 VGRLLLPVLLGFRLALLAASGPGVFS--DEQSEFECNTRMPGCKAA-CFDALHPIS-P--LR-FW  77
Oryctolagus cuniculus CXNP2       20 VGRLLLPVLLGFRLVLLAAGGPGVYG--DEQSEFVCHTQQPGCKSA-CYDAFHPLS-P--LR-FW  77
Ochotona princeps CXNP1           20 VGRLLLPVLLGFRLVLLAACGPGVYG--DEQSEFVCHTQQPGCKAA-CYDAFHPLS-P--LR-FW  77
Bos taurus CXNP1                  20 VGRLLLPALLGLRLVLLAAGGTGVFGGGEEQSEFVCHTQQAGCKAV-CYDAFHPLS-P--LR-FW  79
Equus caballus CXNP1              20 VGRLLLPVLLGFRLVLLAASGTGVYG--DEQSEFVCHTQQPGCKAA-CYDAFHPLS-P--LR-FW  77
Canis lupus familiaris CXNP1      20 VGHLLLPVLLGFRLVLLAASGTGIYG--DEQSEFVCHTQQAGCKAA-CYDAFHPFS-P--LR-FW  77
Felis catus CXNP1                 42 VGRLLLPVLLGFRLVLLAASGAGVYS--DEQSEFVCHTQQPGCKAA-CYDAFHPFS-P--LR-FW  99
Myotis lucifugus CXNP1            20 VGRLILPVLLGFRLILLATSGTGVYG--DEQSEFVCHTQQPGCKVA-CYDAFLPLS-P--LR-FW  77
Dasypus novemcinctus CXNP1        20 VGRLLLPGLLGFRVALLAACGPGVFA--DEQSEFQCNTVQPLCKAA-CFDALQPLS-P--LR-FW  77
Dasypus novemcinctus CXNP2        20 VGRLLLPGLLGFRVALLAACGPGVFA--DEQSEFQCSTQQPLCKAA-CFDALQPLS-P--LR-FW  77
Dasypus novemcinctus CXNP3        20 VGRLLLPGLLGFRVALLAACGPGVFT--DEQSEFECNTQQPGCKAA-CLDALRLLS-P--LR-FW  77
Dasypus novemcinctus CXNP4        20 VGRLLLPGLLGFRVALLAACGPGVFA--DEQSEFQCSTQQPLCKAI-CFDALQPLS-P--LR-FW  77
Choloepus hoffmanni CXNP1         20 VGRLLLPVLLAFRVALLAAGGPGVYS--DEQSEFQCNTQQPGCKAA-CFDAMRPLS-P--LR-FW  77
Loxodonta africana CXNP1          20 VGRLLLPVLLGFRLLLLAATGTGVYG--DEQSEFVCNTQQPGCKAA-CFDAFHPLS-P--LR-FW  77
Homo sapiens CXNQ                 20 VGKIWLTVLIVFRIVLTAVGGESIYY--DEQSKFVCNTEQPGCENV-CYDAFAPLS-H--VR-FW  77
Pan troglodytes CXNQ              20 VGKIWLTVLIVFRIVLTAVGGESIYY--DEQSKFVCNTEQPGCENV-CYDAFAPLS-H--VR-FW  77
Pongo abelii CXNQ                 20 VGKIWLTVLIVFRIVLTAVGGESIYY--DEQSKFVCNTEQPGCENV-CYDAFAPLS-H--VR-FW  77
Macaca mulatta CXNQ               20 VGKIWLTVLIVFRIVLTAVGGESIYY--DEQSKFVCNTEQPGCENV-CYDAFAPLS-H--VR-FW  77
Tupaia belangeri CXNQ             20 VGKIWLTVLIVFRIVLTAVGGESIYY--DEQSKFVCNTEQPGCENV-CYDAFAPLS-H--VR-FW  77
Mus musculus Cxnq                 20 VGKIWLTVLIVFRIVLTAVGGESIYY--DEQSKFVCNTEQPGCENV-CYDAFAPLS-H--VR-FW  77
Rattus norvegicus Cxnq            20 VGKIWLTVLIVFRIVLTAVGGESIYY--DEQSKFVCNTEQPGCENV-CYDAFAPLS-H--VR-FW  77
Cavia porcellus CXNQ              20 VGKIWLTVLIVFRIVLTAVGGESIYY--DEQSKFVCNTEQPGCENV-CYDAFAPLS-H--VR-FW  77
S.tridecemlineatus CXNQ           34 VGKIWLTVLIVFRIVLTAVGGESIYY--DEQSKFVCNTEQPGCENV-CYDAFAPLS-H--VR-FW  91
Oryctolagus cuniculus CXNQ        20 VGKIWLTVLIVFRIVLTAVGGESIYY--DEQSKFVCNTEQPGCENV-CYDAFAPLS-H--VR-FW  77
Bos taurus CXNQ                   20 VGKIWLTVLIVFRIVLTAVGGESIYY--DEQSKFVCNTEQPGCENV-CYDAFAPLS-H--VR-FW  77
Vicugna pacos CXNQ                20 VGKIWLTVLIVFRIVLTAVGGESIYY--DEQSKFVCNTEQPGCENV-CYDAFAPLS-H--VR-FW  77
Equus caballus CXNQ               20 VGKIWFTVLIVFRIVLTAVGGESIYY--DEQSKFVCNTEQPGCENV-CYDAFAPLS-H--VR-FW  77
Canis lupus familiaris CXNQ       20 VGKIWLTVLIVFRIVLTAVGGESIYY--DEQSKFVCNTEQPGCENV-CYDAFAPLS-H--VR-FW  77
Myotis lucifugus CXNQ             20 VGKIWLTVLIVFRIVLTAVGGESIYY--DEQSKFVCNTEQPGCENV-CYDAFAPLS-H--VR-FW  77
Pteropus vampyrus CXNQ            20 VGKIWLTVLIVFRIVLTAVGGESIYY--DEQSKFVCNTEQPGCENV-CYDAFAPLS-H--VR-FW  77
Loxodonta africana CXNQ           20 VGKIWLTILIVFRIVLTAVGGESIYY--DEQSKFVCNTEQPGCENV-CYDAFAPLS-H--VR-FW  77
Homo sapiens CXNR                 21 VGRLWLVVMLIFRILVLATVGGAVFE--DEQEEFVCNTLQPGCRQT-CYDRAFPVS-H--YR-FW  78
Pan troglodytes CXNR              21 VGRLWLVVMLIFRILVLATVGGAVFE--DEQEEFVCNTLQPGCRQT-CYDRAFPVS-H--YR-FW  78
Pongo abelii CXNR                 21 VGRLWLVVMLIFRILVLATVGGAVFE--DEQEEFVCNTLQPGCRQT-CYDRAFPVS-H--YR-FW  78
Papio hamadryas CXNR              21 VGRLWLVVMLIFRILVLATVGGAVFE--DEQEEFVCNTLQPGCRQT-CYDRAFPVS-H--YR-FW  78
Mus musculus Cxnr                 21 VGRLWLVIMLIFRILVLATVGGAVFE--DEQEEFVCNTLQPGCRQT-CYDRAFPVS-H--YR-FW  78
Rattus norvegicus Cxnr            21 VGRLWLVIMLIFRILVLATVGGAVFE--DEQEEFVCNTLQPGCRQT-CYDRAFPVS-H--YR-FW  78
Oryctolagus cuniculus CXNR        21 VGRLWLVVMLIFRILVLATVGGAVFE--DEQEQFVCNTLQPGCRQT-CYDRAFPVS-H--YR-FW  78
Bos taurus CXNR                   21 LGRLWLVVMLIFRILVLATVGGAVFE--DEQEEFVCNTLQPGCRQT-CYDRAFPVS-H--YR-FW  78
Homo sapiens CXNS                  1 --------------------------------MFVCNTLQPGCNQA-CYDRAFPIS-H--IR-YW  28
Pan troglodytes CXNS               1 --------------------------------MFVCNTLQPGCNQA-CYDRAFPIS-H--IR-YW  28
Pongo abelii CXNS                  1 --------------------------------MFVCNTLQPGCNQA-CYDRAFPIS-H--IR-YW  28
Nomascus leucogenys CXNS           1 --------------------------------MFVCNTLQPGCNQA-CYDRAFPIS-H--IR-YW  28
Macaca mulatta CXNS                1 --------------------------------MFVCNTLQPGCNQA-CYDRAFPIS-H--IR-YW  28
Papio hamadryas CXNS               1 --------------------------------MFVCNTLQPGCNQA-CYDRAFPIS-H--IR-YW  28
Otolemur garnettii CXNS            1 --------------------------------MFVCNTLQPGCNQA-CYDRAFPIS-H--IR-YW  28
Tupaia belangeri CXNS              1 --------------------------------MFVCNTLQPGCNQA-CYDRAFPIS-H--IR-YW  28
Mus musculus Cxns                 11 LFRILLTVVVIFRILIVAIVGETVYD--DEQTMFVCNTLQPGCNQA-CYDRAFPIS-H--IR-YW  68
Rattus norvegicus Cxns             1 --------------------------------MFVCNTLQPGCNQA-CYDRAFPIS-H--IR-YW  28
Cavia porcellus CXNS               1 --------------------------------MFVCNTLQPGCNQA-CYDRAFPIS-H--IR-YW  28
S.tridecemlineatus CXNS            1 --------------------------------MFVCNTLQPGCNQA-CYDRAFPIS-H--IR-YW  28
```

```
Oryctolagus cuniculus CXNS      1 --------------------------------MFVCNTLQPGCNQA-CYDRAFPIS-H--IR-YW  28
Ochotona princeps CXNS          1 --------------------------------MFVCNTLQPGCNQA-CYDRAFPIS-H--IR-YW  28
Bos taurus CXNS                 1 --------------------------------MFVCNTLQPGCNQA-CYDRAFPIS-H--IR-YW  28
Equus caballus CXNS             1 --------------------------------MFVCNTLQPGCNQA-CYDRAFPIS-H--IR-YW  28
Canis lupus familiaris CXNS     1 --------------------------------MFVCNTLQPGCNQA-CYDRAFPIS-H--IR-YW  28
Myotis lucifugus CXNS           1 --------------------------------MFVCNTLQPGCNQA-CYDRAFPIS-H--IR-YW  28
Dasypus novemcinctus CXNS       1 --------------------------------MFVCNTLQPGCNQA-CYDRAFPIS-H--IR-YW  28
Loxodonta africana CXNS       100 L-RILLTVVVIFRILIVAIVGETVYD--DEQTMFVCNTLQPGCNQA-CYDRAFPIS-H--IR-YW 156
Homo sapiens CXNT              20 IGQFHTLFFGSIRIFFLGVLGFAVYG--NEALHFICDPDKRE-VNLFCYNQFRPITPQVSFS---  78
Pan troglodytes CXNT           20 IGQFHTLFFGSIRIFFLGVLGFAVYG--NEVLHFICDPDKRE-VNLFCYNQFRSITPQVSFS---  78
Nomascus leucogenys CXNT       20 IGQFHTLFFGLIRMFFLGVLGFAVYG--NEALHFICDPDKKE-VNLFCYNWFSPITPQVSFSVFW  81
Mus musculus Cxnt              20 IGQFHTLFFGSVRMFFLGVLGFAVYG--NEALHFSCDPDKRE-INLFCYNQFRPITPQVSFLVFW  81
Rattus norvegicus Cxnt         20 IGQFHTLFFGSVRMFFLGVLGFAVYG--NEALHFSCDPDKRE-INLFCYNQFRPITPQVSFLVFW  81
Cavia porcellus CXNT           20 IGQFHTLFFGSVRMFFLGVLGFAVYG--NEALHFSCDPDKRE-INLFCYNQFRPITPQV----FW  77
Oryctolagus cuniculus CXNT     20 IGQFHTLFFGSVRMFFLGVLGFAVYG--NEALHFSCDPDKRE-VNLFCYNQFRPITPQVSFPVFW  81
Bos taurus CXNT                20 IGQFHTLFFGSVRMFFLGVLGFAVYG--NEALHFSCDPDKRE-INLFCYNQFRPITPQVSFPVFW  81
Vicugna pacos CXNT             20 IGQFHTLFFGSVRMFFLGVLGFAVYG--NEALHFSCDPDRRE-INLFCYNQFRPITPQVSFSVFW  81
Equus caballus CXNT            20 IGQFHTLFFGSVRMFFLGVLGFAVYG--NEAQHFSCDPDKRE-INLFCYSQFRPITPQVSLSVFW  81
Canis lupus familiaris CXNT    20 IGQFHTLFFGSVRMFFLGVLGFAVYG--NEALHFSCDPDKRE-INLFCYNQFRPITPQVSFPVFW  81
Myotis lucifugus CXNT          20 IGQFHTLFFGSIRMFFLGVLGFAVYG--NEALHFSCDPDRRE-INLFCYNQFRPITPQVSFSVFW  81
Dasypus novemcinctus CXNT      20 MGQFHTLFFGSVRTFFLGVLGFAVYG--NEALHFSCDPDKRE-VNLFCYNQFRPVTPQASSSVFW  81
Loxodonta africana CXNT        20 IGQFHTLFFGSVRMFFLGVLGFAVYG--NEALHFSCDPDKRE-INLFCYNQFRPITLQ----VFW  77
Homo sapiens CXNU               7 PGKLWFVLTMLLRMLVIVLAGRPVYQ--DEQERFVCNTLQPGCANV-CYDVFSPVS-H--LR-FW  64
Pan troglodytes CXNU            7 PGKLWFVLTMLLRMLVIVLAGRPVYQ--DEQERFVCNTLQPGCANV-CYDVFSPVS-H--LR-FW  64
Pongo abelii CXNU               1 ---------MLLRMLVIVLAGRPVYQ--DEQERFVCNTLQPGCANV-CYDVFSPVS-H--LR-FW  49
Nomascus leucogenys CXNU        7 PGKLWFVLTMLLRMLVIVLAGRPVYQ--DEQERFVCNTLQPGCANV-CYDVFSPVS-H--LR-FW  64
Macaca mulatta CXNU             7 PGKLWFVLTMLLRMLVIVLAGRPVYQ--DEQERFVCNTLQPGCANV-CYDVFFPVS-H--LR-FW  64
Mus musculus Cxnu               1 --MIWLIVEVLLRMLVVVLAGSPIYE--DEQERFICNTLQPGCANV-CYDLFSPVS-P--LR-FW  56
Cavia porcellus CXNU            1 -------------MGVVILAGYPIYQ--DEQERFVCNTLQPGCANV-CYDIFSPVS-Q--LR-FW  45
Bos taurus CXNU                 1 --------MVLLRMVVLILAGSPVYQ--DEQERFVCNTLQPGCANV-CYDIFAPVS-H--LR-FW  50
Equus caballus CXNU             1 ---------MLLRMAVVVLAGAPVYQ--DEQERFVCNTLQPGCANV-CYDLFSPVS-H--LR-FW  49
Myotis lucifugus CXNU           8 PGKIWLIFMILLRMVVIIVAGSPVYQ--DEQERFVCNTLQPGCANV-CYDVFSPVS-H--LR-FW  65
Sorex araneus CXNU              1 --------MMLLRMAVIVLAGSPVYH--DEQDRFVCNTLQPGCTNV-CYDVFSPVS-H--LR-FW  50
Dasypus novemcinctus CXNU      19 LGKIWLIFTILLRMLMIILAGYPIYQ--DEQERFVCNTLQPGCSNV-CYDIFSPVS-H--FR-FW  76
Loxodonta africana CXNU        19 LGKIWLIFMTLLRMMVIILAGYPIYQ--DEQERFVCNTLQPGCSNV-CYDIFSPVS-H--FR-FW  76
```

```
Homo sapiens CXNA               78 ALQLILV--TCPSLL-VVMHVA-YR--EVQE-K-R-HR--------------------------- 106
                                            270       280       290       300       310       320
Homo sapiens CXNA               78 ALQLILV--TCPSLL-VVMHVA-YR--EVQE-K-R-HR--------------------------- 106
Pan troglodytes CXNA            78 ALQLILV--TCPSLL-VVMHVA-YR--EVQE-K-R-HR--------------------------- 106
Gorilla gorilla CXNA            78 ALQLILV--TCPSLL-VVMHVA-YR--EVQE-K-R-HR--------------------------- 106
Pongo abelii CXNA               78 ALQLILV--TCPSLL-VVMHVA-YR--EVQE-K-R-HR--------------------------- 106
Nomascus leucogenys CXNA        78 ALQLILV--TCPSLL-VVMHVA-YR--EVQE-K-R-HR--------------------------- 106
Macaca mulatta CXNA             78 ALQLILV--TCPSLL-VVMHVA-YR--EVQE-K-R-HR--------------------------- 106
Callithrix jacchus CXNA         78 ALQLILV--TCPSLL-VVMHVA-YR--ELQE-K-K-HQ--------------------------- 106
Mus musculus Cxna               78 ALQLILV--TCPSLL-VVMHVA-YR--KARE-K-K-YQ--------------------------- 106
Rattus norvegicus Cxna          78 ALQLILV--TCPSLL-VVMHVA-YR--KARE-K-K-YQ--------------------------- 106
Cavia porcellus CXNA           128 ALQLILV--TCPSLL-VVMHVA-YR--KAQE-K-K-HR--------------------------- 156
Ochotona princeps CXNA          78 ALQLILV--TCPSLL-VVMHVA-YR--EARE-K-K-HQ--------------------------- 106
Bos taurus CXNA                 78 ALQLILV--TCPSLL-VVMHVA-YR--QARE-K-K-HQ--------------------------- 106
Equus caballus CXNA             78 ALQLILV--TCPSLL-VVMHVA-YR--EARE-K-K-HR--------------------------- 106
Canis lupus familiaris CXNA     78 ALQLILV--TCPSLL-VLMHVA-YR--EAQE-K-K-HR--------------------------- 106
Felis catus CXNA                78 ALQLILV--TCPSLL-VVMHVA-YR--EARE-K-K-HR--------------------------- 106
Myotis lucifugus CXNA          110 ALQLILV--TCPSLL-VVMHVA-YR--EARE-K-K-HR--------------------------- 138
Dasypus novemcinctus CXNA       78 ALQLILV--TCPSLL-VVMHVA-YR--KARE-K-K-HQ--------------------------- 106
Loxodonta africana CXNA         78 ALQLILV--TCPSLL-VVMHVA-YR--EARE-K-K-HR--------------------------- 106
Homo sapiens CXNB               78 ALQLILV--TCPSLL-VVMHVA-YR--EERE-R-K-HH--------------------------- 106
Gorilla gorilla CXNB            78 ALQLILV--TCPSLL-VVMHVA-YR--EERE-R-K-HH--------------------------- 106
Nomascus leucogenys CXNB        78 ALQLILV--TCPSLL-VVMHVA-YR--EERE-R-K-HH--------------------------- 106
Macaca mulatta CXNB             78 ALQLILV--TCPSLL-VVMHVA-YR--EERE-R-K-HR--------------------------- 106
Callithrix jacchus CXNB         78 ALQLILV--TCPSLL-VVMHVA-YR--EERE-R-R-YR--------------------------- 106
Otolemur garnettii CXNB         78 ALQLILV--TCPSLL-VVMHVA-YR--EDRE-R-K-HR--------------------------- 106
Mus musculus Cxnb               78 ALQLILV--TCPSLL-VVMHVA-YR--EERE-R-K-HR--------------------------- 106
Rattus norvegicus Cxnb          78 ALQLILV--TCPSLL-VVMHVA-YR--EERE-R-K-HR--------------------------- 106
Dipodomys ordii CXNB            78 ALQLILV--TCPSLL-VVMHVA-YR--EERE-R-K-HR--------------------------- 106
Cavia porcellus CXNB            78 ALQLILV--TCPSLL-VGMHVI-YR--EERE-Q-K-HR--------------------------- 106
Oryctolagus cuniculus CXNB       1 ------------------MHVA-YR--EERE-R-K-HR---------------------------  14
Ochotona princeps CXNB          78 ALQLILV--TCPSLL-VVMHVA-YR--EDRE-R-K-HR--------------------------- 106
Bos taurus CXNB                 78 ALQLILV--TCPSLL-VVMHVA-YR--QERE-R-K-HR--------------------------- 106
Equus caballus CXNB             78 ALQLILV--TCPSLL-VVMHVA-YR--EERE-R-K-HR--------------------------- 106
Canis lupus familiaris CXNB    128 ALQLILV--TCPSLL-VVMHVA-YR--QERE-R-K-HR--------------------------- 156
Myotis lucifugus CXNB           78 ALQLILV--TCPSLL-VVMHVA-YR--EERE-R-R-HR--------------------------- 106
Erinaceus europaeus CXNB        78 ALQLILV--TCPSLL-VVMHVA-YR--QERE-R-K-HR--------------------------- 106
Loxodonta africana CXNB         78 ALQLILV--TCPSLL-VVMHVA-YR--EERE-R-R-HR--------------------------- 106
Homo sapiens CXNC               78 ALQLIFV--TCPSLL-VILHVA-YR--EERE-R-R-HR--------------------------- 106
Gorilla gorilla CXNC            78 ALQLIFV--TCPSLL-VILHVA-YR--EERE-R-R-HR--------------------------- 106
Pongo abelii CXNC               78 ALQLIFV--TCPSLL-VILHVA-YR--EERE-R-R-HR--------------------------- 106
Nomascus leucogenys CXNC        78 ALQLIFV--TCPSLL-VILHVA-YR--EERE-R-R-HR--------------------------- 106
Callithrix jacchus CXNC         78 ALQLIFV--TCPSLL-VILHVA-YR--EERE-R-R-HR--------------------------- 106
Microcebus murinus CXNC        108 ALQLIFV--TCPSLL-VILHVA-YR--EDRE-R-R-HR--------------------------- 136
Otolemur garnettii CXNC         78 ALQLIFV--TCPSLL-VILHVA-YR--EERE-R-R-HR--------------------------- 106
Mus musculus Cxnc               78 ALQLIFV--TCPSML-VILHVA-YR--EERE-R-K-HR--------------------------- 106
Rattus norvegicus Cxnc          78 ALQLIFV--TCPSML-VILHVA-YR--EERE-R-K-HR--------------------------- 106
Cavia porcellus CXNC            78 ALQLIFV--TCPSLL-VILHVA-YR--EERE-R-K-HR--------------------------- 106
Oryctolagus cuniculus CXNC     188 ALQLIFV--TCPSLL-VILHVA-YR--EERE-R-R-HR--------------------------- 216
Bos taurus CXNC                 78 ALQLIFV--TCPSLL-VILHVA-YR--EERE-R-R-HR--------------------------- 106
Equus caballus CXNC             78 ALQLIFV--TCPSLL-VILHVA-YR--EERE-R-K-HR--------------------------- 106
Canis lupus familiaris CXNC     78 ALQLIFV--TCPSLL-VMLHVA-YR--EERE-R-R-HR--------------------------- 106
Felis catus CXNC                78 ALQLIFV--TCPSLL-VILHVA-YR--EERE-R-R-HR--------------------------- 106
Myotis lucifugus CXNC           78 ALQLIFV--TCPSLL-VILHVA-YR--EERE-R-R-HR--------------------------- 106
Dasypus novemcinctus CXNC       78 ALQLIFV--TCPSLL-VILHVA-YR--EERE-R-K-HR--------------------------- 106
Loxodonta africana CXNC         78 ALQLIFV--TCPSLL-VILHVA-YR--EERE-R-R-HR--------------------------- 106
Homo sapiens CXND               78 ALQLIMV-ST-PSLL-VVLHVA-YH--EGRE-K-R-HR--------------------------- 106
Pan troglodytes CXND            78 ALQLIMV-ST-PSLL-VVLHVA-YH--EGRE-K-M-HR--------------------------- 106
Macaca mulatta CXND             78 ALQLIMV-ST-PSLL-VVLHVA-YR--ESRE-K-R-HR--------------------------- 106
Callithrix jacchus CXND         78 ALQLIMV-ST-PSLL-VVLHVA-YR--EGRE-K-R-HR--------------------------- 106
Dipodomys ordii CXND            78 ALQLIMV-ST-PSLL-VVLHVA-YR--ESRE-K-R-LR--------------------------- 106
Oryctolagus cuniculus CXND      78 ALQLIMV-ST-PSLL-VVLHVA-YR--EGRE-K-R-HR--------------------------- 106
Tursiops truncatus CXND         78 ALQLIVV-ST-PSLL-VVLHVA-YR--EGRE-K-R-HR--------------------------- 106
Bos taurus CXND                  1 -----MV-ST-PSLL-VVLHVA-YR--EGRE-K-R-HR---------------------------  24
Equus caballus CXND             97 ALQLIMV-ST-PSLL-VVLHVA-YC--EGRE-K-R-HR--------------------------- 125
Canis lupus familiaris CXND     78 ALQLIMV-ST-PSLL-VVLHVA-YR--EGRE-K-R-HR--------------------------- 106
Myotis lucifugus CXND           78 ALQLIMV-ST-PSLL-VVLHVA-YR--EGRE-K-R-HR--------------------------- 106
Erinaceus europaeus CXND        78 AIQLIMV-ST-PSLL-VVLHVA-YR--EGRE-K-R-HR--------------------------- 106
Dasypus novemcinctus CXND       78 ALQLIMV-SA-PSLL-VVLHVA-YR--ESRE-K-R-QR--------------------------- 106
Choloepus hoffmanni CXND        78 ALQLIMV-ST-PSLL-VVLHVA-YR--EGRE-K-R-HR--------------------------- 106
Loxodonta africana CXND         78 ALQLIMV-SI-PSLL-VALHVA-YH--EGRE-K-K-HR--------------------------- 106
Homo sapiens CXNE               78 ALQLIFV-ST-PALL-VAMHVA-YR--RH-E-K-K-RK--------------------------- 105
Pan troglodytes CXNE            78 ALQLIFV-ST-PALL-VAMHVA-YR--RH-E-K-K-RK--------------------------- 105
Pongo abelii CXNE               78 ALQLIFV-ST-PALL-VAMHVA-YR--RH-E-K-K-RK--------------------------- 105
Macaca mulatta CXNE             78 ALQLIFV-ST-PALL-VAMHVA-YR--RH-E-K-K-RK--------------------------- 105
Papio hamadryas CXNE            78 ALQLIFV-ST-PALL-VAMHVA-YR--RH-E-K-K-RK--------------------------- 105
Callithrix jacchus CXNE         78 ALQLIFV-ST-PALL-VAMHVA-YR--RH-E-K-K-RK--------------------------- 105
Otolemur garnettii CXNE         78 ALQLIFV-ST-PALL-VAMHVA-YR--RH-E-K-K-RK--------------------------- 105
Mus musculus Cxne               91 ALQLIMV-ST-PALL-VAMHVA-YR--RH-E-K-K-RK--------------------------- 118
```

```
Rattus norvegicus Cxne            97 ALQLIMV-ST-PALL-VAMHVA-YR--RH-E-K-K-RK--------------------------- 124
Dipodomys ordii CXNE              97 ALQLIFV-ST-PALL-VAMHVA-YR--RH-E-K-K-RK--------------------------- 124
Cavia porcellus CXNE             125 ALQLIFV-ST-PALL-VAMHVA-YR--KH-E-K-K-RR--------------------------- 152
Oryctolagus cuniculus CXNE        97 ALQLIFV-ST-PALL-VAMHVA-YR--RH-E-K-K-RK--------------------------- 124
Equus caballus CXNE               78 ALQLIFV-ST-PALL-VAMHVA-YR--RH-E-K-K-RK--------------------------- 105
Canis lupus familiaris CXNE       78 ALQLIFV-ST-PALL-VAMHVA-YR--RH-E-K-K-RK--------------------------- 105
Felis catus CXNE                  78 ALQLIFV-ST-PALL-VAMHVA-YR--RH-E-K-K-RK--------------------------- 105
Myotis lucifugus CXNE             78 ALQLIFV-ST-PALL-VAMHVA-YH--RH-E-K-K-RK--------------------------- 105
Sorex araneus CXNE                78 ALQLIFV-ST-PALL-VAMHVA-YR--RH-E-K-K-RK--------------------------- 105
Dasypus novemcinctus CXNE         78 ALQLIFV-ST-PALL-VAMHVA-YQ--RH-E-K-K-RK--------------------------- 105
Loxodonta africana CXNE           78 ALQLIFV-SA-PALL-VAMHVA-YR--RH-E-K-K-RK--------------------------- 105
Homo sapiens CXNF                 78 ALQLIFV-ST-PALL-VAMHVA-YY--RH-E-T-T-RK--------------------------- 105
Pan troglodytes CXNF              78 ALQLIFV-ST-PALL-VAMHVA-YY--RH-E-T-T-RK--------------------------- 105
Gorilla gorilla CXNF              78 ALQLIFV-ST-PALL-VAMHVA-YY--RH-E-T-T-RK--------------------------- 105
Nomascus leucogenys CXNF          78 ALQLIFV-ST-PALL-VAMHVA-YY--RH-E-T-T-RK--------------------------- 105
Callithrix jacchus CXNF           78 ALQLIFV-ST-PALL-VAMHVA-YY--RQ-E-T-A-RK--------------------------- 105
Mus musculus Cxnf                 78 ALQLIFV-ST-PALL-VAMHVA-YY--RH-E-T-A-RK--------------------------- 105
Rattus norvegicus Cxnf            78 ALQLIFV-ST-PALL-VAMHVA-YY--RH-E-T-A-RK--------------------------- 105
S.tridecemlineatus CXNF           78 ALQLIFV-ST-PALL-VAMHVA-YY--RH-E-T-A-RR--------------------------- 105
Oryctolagus cuniculus CXNF        78 ALQLIFV-ST-PALL-VAMHVA-YY--RH-E-T-A-RK--------------------------- 105
Ochotona princeps CXNF            78 ALQLIFV-ST-PALL-VAMHVA-YY--RH-E-T-A-RK--------------------------- 105
Bos taurus CXNF                   78 ALQLIFV-ST-PALL-VAMHVA-YY--RH-E-A-A-RR--------------------------- 105
Equus caballus CXNF               78 ALQLIFV-ST-PALL-VAMHVA-YY--RR-E-T-A-RK--------------------------- 105
Canis lupus familiaris CXNF       78 ALQLIFV-ST-PALL-VAMHVA-YY--RH-E-T-A-RK--------------------------- 105
Myotis lucifugus CXNF             78 ALQLIFV-ST-PALL-VAMHVA-YY--RQ-E-A-A-RK--------------------------- 105
Dasypus novemcinctus CXNF         78 ALQLIFV-ST-PAML-VAMHVA-YY--RH-A-T-A-RK--------------------------- 105
Choloepus hoffmanni CXNF          78 ALQLIFV-ST-PAML-VGMHVT-YY--RH-K-T-E-RK--------------------------- 105
Loxodonta africana CXNF           78 ALQLIFV-ST-PALL-VAMHVA-YY--RH-E-T-A-RK--------------------------- 105
Homo sapiens CXNG                 78 SLQLILV-ST-PALL-VAMHVA-HQ--QHIE-K-K------------------------------ 104
Pan troglodytes CXNG              78 SLQLILV-ST-PALL-VAMHVA-HQ--QHIE-K-K------------------------------ 104
Gorilla gorilla CXNG              78 SLQLILV-ST-PALL-VAMHVA-HQ--QHIE-K-K------------------------------ 104
Pongo abelii CXNG                 78 SLQLILV-ST-PALL-VAMHVA-HQ--QHIE-K-K------------------------------ 104
Nomascus leucogenys CXNG          78 SLQLILV-ST-PALL-VAMHVA-HQ--QHIE-K-K------------------------------ 104
Macaca mulatta CXNG               78 SLQLILV-ST-PALL-VAMHVA-HQ--QHIE-K-K------------------------------ 104
Papio hamadryas CXNG              78 SLQLILV-ST-PALL-VAMHVA-HQ--QHIE-K-K------------------------------ 104
Callithrix jacchus CXNG           78 SLQLILV-ST-PALL-VAMHVA-HQ--QHIE-K-K------------------------------ 104
Microcebus murinus CXNG           78 SLQLILV-ST-PALL-VAMHVA-HQ--QHIE-K-K------------------------------ 104
Mus musculus Cxng                 78 SLQLILV-ST-PALL-VAMHVA-HQ--QHIE-K-K------------------------------ 104
Rattus norvegicus Cxng            78 SLQLILV-ST-PALL-VAMHVA-HQ--QHIE-K-K------------------------------ 104
Oryctolagus cuniculus CXNG        78 SLQLILV-ST-PALL-VAMHVA-HQ--QHIE-K-K------------------------------ 104
Bos taurus CXNG                   78 SLQLILV-ST-PALL-VAMHVA-HQ--QHIE-K-K------------------------------ 104
Equus caballus CXNG               78 SLQLILV-ST-PALL-VAMHVA-HQ--QHIE-K-K------------------------------ 104
Canis lupus familiaris CXNG       78 SLQLILV-ST-PALL-VAMHVA-HQ--QHIE-K-K------------------------------ 104
Felis catus CXNG                  78 SLQLILV-ST-PALL-VAMHVA-HQ--QHIE-K-K------------------------------ 104
Myotis lucifugus CXNG             78 SLQLILV-ST-PALL-VAMHVA-HQ--QHIE-K-K------------------------------ 104
Pteropus vampyrus CXNG            78 SLQLIMV-ST-PTLL-VAMHVA-HQ--QHVE-K-K------------------------------ 104
Dasypus novemcinctus CXNG         78 SLQLILV-ST-PALL-VAMHVA-HQ--QHLE-K-K------------------------------ 104
Loxodonta africana CXNG           78 SLQLILV-ST-PALL-VAMHVA-HQ--QHIE-K-K------------------------------ 104
Procavia capensis CXNG            78 SLQLILV-ST-PALL-VAMHVA-HQ--QHIE-K-K------------------------------ 104
Homo sapiens CXNH1                79 VLQFLFV-ST-PTLV-YLGHVI-YLS-RREE-R-L-RQ-K------------------------- 109
Pan troglodytes CXNH1             79 VLQFLFV-ST-PTLV-YLGHVI-YLS-RQEE-R-L-RQ-K------------------------- 109
Gorilla gorilla CXNH1             79 VLQFLFV-ST-PTLV-YLGHVI-YLS-RREE-R-L-RQ-K------------------------- 109
Pongo abelii CXNH1                79 VLQFLFV-ST-PTLV-YLGHVI-YLS-RREE-R-L-RQ-K------------------------- 109
Nomascus leucogenys CXNH1         79 VLQFLFV-ST-PTLV-YLGHVI-YLS-RREE-R-L-RQ-K------------------------- 109
Macaca mulatta CXNH1              79 VLQFLFV-ST-PTLV-YLGHVI-YLS-RREE-R-L-RQ-K------------------------- 109
Otolemur garnettii CXNH1          90 VLQFFFI-ST-PTLV-YLGHVM-YYS-WRKK-Q---RQ-K------------------------- 119
Mus musculus Cxnh1                79 VLQFLFV-ST-PTLI-YLGHVI-YLS-RREE-R-L-RQ-K------------------------- 109
Rattus norvegicus Cxnh1           79 VLQFLFV-ST-PTLI-YLGHVI-YLS-RREE-R-L-RQ-K------------------------- 109
Cavia porcellus CXNH1             79 VLQFLFV-ST-PTLV-YLGHVI-YLS-RREE-R-L-RQ-K------------------------- 109
Oryctolagus cuniculus CXNH1       79 VLQFLFV-ST-PTLV-YLGHVI-YLS-RREE-R-L-RQ-K------------------------- 109
Ochotona princeps CXNH1           93 VLQFLFV-ST-PTLV-YLGHVI-YLS-RREE-R-L-RQ-K------------------------- 123
Equus caballus CXNH1              79 VLQFLFV-ST-PTLV-YLGHVI-YLS-HREE-R-L-RQ-K------------------------- 109
Canis lupus familiaris CXNH1      79 VLQFLFV-ST-PTLV-YLGHVI-YLS-RREE-R-L-RQ-K------------------------- 109
Felis catus CXNH1                 79 VLQFLFV-ST-PTLV-YLGHVI-YLS-RREE-R-L-RQ-K------------------------- 109
Myotis lucifugus CXNH1           111 VLQFLFV-ST-PTLI-YLAHVI-HLS-RREE-R-L-RQ-K------------------------- 141
Dasypus novemcinctus CXNH1        79 VLQFLFV-ST-PTLV-YLGHVI-YLS-RREE-R-L-RQ-K------------------------- 109
Loxodonta africana CXNH1          79 VLQFLFV-ST-PTLV-YLGHVI-YLS-RREE-R-L-RQ-K------------------------- 109
Homo sapiens CXNI                 79 VLQIIFV-ST-PSLV-YMGHAM-HTV-RMQE-K-R-KL-R------------------------- 109
Pan troglodytes CXNI              79 VLQIIFV-ST-PSLV-YMGHAM-HTV-RMQE-K-R-KL-R------------------------- 109
Pongo abelii CXNI                 79 VLQIIFV-ST-PSLV-YMGHAM-HTV-RMQE-K-R-KL-R------------------------- 109
Nomascus leucogenys CXNI          79 VLQIIFV-ST-PSLV-YMGHAM-HTV-RMQE-K-R-KL-R------------------------- 109
Macaca mulatta CXNI               79 VLQIIFV-ST-PSLV-YMGHAM-HTV-RMQE-K-R-KL-R------------------------- 109
Papio hamadryas CXNI              79 VLQIIFV-ST-PSLV-YMGHAM-HTV-RMQE-K-R-KL-R------------------------- 109
Callithrix jacchus CXNI           79 VLQIIFV-ST-PSLV-YMGHAM-HTV-RMQE-K-R-RL-R------------------------- 109
Otolemur garnettii CXNI           79 VLQIIFV-ST-PSLV-YMGHAM-HTV-RMQE-K-R-KL-R------------------------- 109
Mus musculus Cxni                 79 VLQIIFV-ST-PSLV-YMGHAM-HTV-RMQE-K-Q-KL-R------------------------- 109
Rattus norvegicus Cxni           113 VLQIIFV-ST-PSLV-YMGHAM-HTV-RMQE-K-Q-KL-R------------------------- 143
Cavia porcellus CXNI              79 VLQIIFV-ST-PSLV-YMGHAM-HTV-RMQE-K-R-KL-R------------------------- 109
Oryctolagus cuniculus CXNI        79 VLQIIFV-ST-PSLV-YMGHAM-HTV-RMQE-K-R-KM-R------------------------- 109
```

```
Bos taurus CXNI                     79 VLQVIFV-ST-PSLV-YLGHAV-HMV-RVQE-K-R-KLLR------------------------- 110
Equus caballus CXNI                 79 VLQVIFV-ST-PSLV-YLFHTM-HRV-RMQE-K-Q-RQ-R------------------------- 109
Canis lupus familiaris CXNI         79 VLQIIFV-ST-PSLV-YMGHAM-HTV-RMQE-K-R-KL-R------------------------- 109
Felis catus CXNI                    79 VLQTIFV-ST-PSLV-YMGHAM-HTV-RMQE-K-R-KL-R------------------------- 109
Pteropus vampyrus CXNI              79 VLQIIFV-ST-PSLV-YMGHAV-HHV-RMEE-K-R-KL-R------------------------- 109
Erinaceus europaeus CXNI            79 VLQIIFV-ST-PSLL-YMGHAM-HTV-RMQE-K-R-RL-R------------------------- 109
Dasypus novemcinctus CXNI           79 VLQIIFV-ST-PSLL-YMGHAM-HTV-RMQE-K-R-KL-R------------------------- 109
Loxodonta africana CXNI             79 VLQIIFV-ST-PSLV-YMGHAM-HMV-RMQE-K-R-KL-R------------------------- 109
Homo sapiens CXNJ1                  79 ALQIIFV-ST-PTLI-YLGHVL-HIV-RMEE-K-K-KE-R------------------------- 109
Pongo abelii CXNJ1                  79 ALQIIFV-ST-PTLI-YLGHVL-HIV-RMEE-K-K-KE-R------------------------- 109
Macaca mulatta CXNJ1                79 ALQIIFV-ST-PTLI-YLGHVL-HMV-RMEE-K-R-KA-R------------------------- 109
Mus musculus Cxnj1                  79 ALQIIFV-ST-PTLI-YLGHVL-HIV-RMEE-K-K-KE-R------------------------- 109
Rattus norvegicus Cxnj1             79 ALQIIFV-ST-PTLI-YLGHVL-HIV-RMEE-K-K-KE-R------------------------- 109
Bos taurus CXNJ1                    79 VLQIIFV-ST-PTLI-YLGHVL-HLV-RMEE-K-R-KE-R------------------------- 109
Equus caballus CXNJ1                79 VLQIIFV-ST-PTLI-YLGHVL-HLV-RMEE-K-K-KE-R------------------------- 109
Myotis lucifugus CXNJ1              79 VLQIIFV-ST-PTLI-YLGHVL-HMV-RMEE-K-K-KG-R------------------------- 109
Myotis lucifugus CXNJ2              79 VLQIIFV-ST-PTLI-YLGHVL-HIV-RMEE-K-K-KG-R------------------------- 109
Pteropus vampyrus CXNJ1            248 VLQIIFV-ST-PTLI-YLGHVL-HIV-RTEQ-K-R-KE-R------------------------- 278
Pteropus vampyrus CXNJ2            170 VLQIIFV-ST-PTLI-YLGHVL-HIV-RTEQ-K-R-KE-R------------------------- 200
Sorex araneus CXNJ1                 79 VLQIIFV-ST-PTLV-YLGHVL-HLA-RVGV-R-R-GQ-R------------------------- 109
Homo sapiens CXNK1                  79 VLQIIFV-SV-PILL-YLAHVF-YVM-QKEE-K-L-NK-K------------------------- 109
Homo sapiens CXNK2                  79 VLQIIFV-SV-PTLL-YLAHVF-YVM-RKEE-K-L-NK-K------------------------- 109
Pan troglodytes CXNK1               79 VLQIIFV-SV-PTLL-YLAHVF-YVM-QKEE-K-L-NK-K------------------------- 109
Pan troglodytes CXNK2               79 VLQIIFV-SV-PTLL-YLAHVF-YVM-RKEE-K-L-NK-K------------------------- 109
Pongo abelii CXNK1                  79 VLQIIFV-SV-PTLL-YLAHVF-YVM-RKEE-K-L-NK-K------------------------- 109
Nomascus leucogenys CXNK1           79 VLQIIFV-SV-PTLL-YLAHVF-YVM-RKEE-K-L-NK-K------------------------- 109
Callithrix jacchus CXNK1            79 VLQIIFV-SV-PTLL-YLAHVF-YVM-RKEE-K-L-NK-K------------------------- 109
Mus musculus Cxnk1                  79 VLQIIFV-SV-PTLL-YLAHVF-YVM-RKEE-K-L-NK-K------------------------- 109
Mus musculus Cxnk2                  79 VLQVIFV-SV-PILL-YLAHVY-YVV-RQNK-K-L-NK-Q------------------------- 109
Rattus norvegicus Cxnk1             79 VLQIIFV-SV-PTLL-YLAHVF-YVM-RKEE-K-L-NK-K------------------------- 109
Rattus norvegicus Cxnk2             79 VLQVIFV-SV-PTLL-HLAHVY-YVI-RQNE-K-L-KK-Q------------------------- 109
Cavia porcellus CXNK1               79 VLQIIFV-SV-PTLL-YLAHVF-YVM-RKEE-K-L-NK-K------------------------- 109
Oryctolagus cuniculus CXNK1         79 VLQIIFV-SV-PTLL-YLAHVF-YVM-RKEE-K-L-NK-K------------------------- 109
Bos taurus CXNK1                    79 VLQIIFV-SV-PTLL-YLAHVF-YVM-RKEE-K-L-NK-K------------------------- 109
Vicugna pacos CXNK1                 79 VLQIIFV-SV-PTLL-YLAHVF-YVM-RKEE-K-L-NK-K------------------------- 109
Equus caballus CXNK1                79 VLQIIFV-SV-PTLL-YLAHVF-YVT-RKEE-K-L-NK-K------------------------- 109
Equus caballus CXNK2                56 VLQIIFV-SV-PTLF-YLAHVF-YVM-RKEE-K-L-NK-K-------------------------  86
Canis lupus familiaris CXNK1         79 VLQIIFV-SV-PTLL-YLAHVF-YVM-RKEE-K-L-NK-K------------------------- 109
Canis lupus familiaris CXNK2         79 VLQIIFV-SI-PSLL-YLVHIF-YIM-RKEE-K-L-KQ-K------------------------- 109
Felis catus CXNK1                   79 VLQIIFV-SV-PSLL-YLVHVF-YLL-RKEH-N-L-KK-K------------------------- 109
Myotis lucifugus CXNK1              79 VLQIIFV-SV-PTLL-YLAHVF-YVM-RKEE-K-L-NK-K------------------------- 109
Dasypus novemcinctus CXNK1          79 VLQIIFV-SV-PTLL-YLSHVF-YVM-RKEE-K-L-NK-K------------------------- 109
Dasypus novemcinctus CXNK2          79 VLQIIFV-SV-PTLL-YLAHVF-YVM-RKEE-K-L-NK-K------------------------- 109
Loxodonta africana CXNK1            79 VLQIIFV-SV-PTLL-YLAHVF-YVM-RKEE-K-L-NK-K------------------------- 109
Loxodonta africana CXNK2            79 GLQIIFV-SV-PTLV-YLAHVF-YVM-RKEE-K-L-NK-K------------------------- 109
Homo sapiens CXNL                   79 VLQIIFV-ST-PSLM-YVGHAV-HYV-RMEE-K-R-KS-R------------------------- 109
Pan troglodytes CXNL                79 VLQIIFV-ST-PSLM-YVGHAV-HYV-RMEE-K-R-KS-R------------------------- 109
Pongo abelii CXNL                   79 VLQIIFV-ST-PSLM-YVGHAV-HYV-RMEE-K-R-KS-R------------------------- 109
Nomascus leucogenys CXNL            79 VLQIIFV-ST-PSLM-YVGHAV-HYV-RMEE-K-R-KS-R------------------------- 109
Macaca mulatta CXNL                 79 VLQIIFV-ST-PSLM-YVGHAV-HYV-RMKE-K-R-KS-R------------------------- 109
Callithrix jacchus CXNL             79 VLQIIFV-ST-PSLM-YVGHAV-HFV-RMEE-K-R-KS-R------------------------- 109
Otolemur garnettii CXNL            124 VLQIIFV-ST-PSLM-YVGHAV-HHV-RMEE-K-R-KD-R------------------------- 154
Mus musculus Cxnl                   79 VLQIIFV-ST-PSLM-YVGHAV-HHV-RMEE-K-R-KD-R------------------------- 109
Rattus norvegicus Cxnl              79 VLQIIFV-ST-PSLM-YVGHAV-HHV-RMEE-K-R-KD-R------------------------- 109
Cavia porcellus CXNL                79 VLQIIFV-ST-PSLM-YVGHAV-HYV-RMEE-K-R-KD-R------------------------- 109
Oryctolagus cuniculus CXNL          79 VLQIIFV-ST-PSLM-YVGHAV-HHV-RMEE-K-R-KD-R------------------------- 109
Ochotona princeps CXNL              79 VLQIIFV-ST-PSLM-YVGHAV-HHV-RMEE-K-R-KD-R------------------------- 109
Bos taurus CXNL                     79 VLQIIFV-ST-PSLV-YVGHAV-HHV-RMEE-K-R-KE-R------------------------- 109
Equus caballus CXNL                 79 VLQIIFV-ST-PSLV-YVGHAV-HHV-RMEE-K-R-KE-R------------------------- 109
Canis lupus familiaris CXNL          79 VLQIIFV-ST-PSLV-YVGHAV-HHV-RMEE-K-R-KE-R------------------------- 109
Felis catus CXNL                    79 VLQIIFV-ST-PSLV-YVGHAV-HHV-RMQE-K-R-KE-R------------------------- 109
Pteropus vampyrus CXNL              79 VLQIIFV-ST-PSLV-YVGHAV-HHV-RMEE-K-R-KE-R------------------------- 109
Sorex araneus CXNL                  79 VLQIIFV-SA-PSLV-LVGHAL-HLL-RAQQ-K-R-Q---------------------------- 107
Dasypus novemcinctus CXNL           79 VLQIIFI-ST-PSLV-YVGHAM-HHV-RMEE-K-R-KE-R------------------------- 109
Loxodonta africana CXNL             79 VLQIIFV-ST-PSLV-YVGHAV-HYL-RMEE-K-R-KE-R------------------------- 109
Homo sapiens CXNM                   79 VLQVIFV-SS-PSLV-YMGHAL-YRL-RVLE-E-E-RQ-R------------------------- 109
Pan troglodytes CXNM                79 VLQVIFV-SS-PSLV-YMGHAL-YRL-RVLE-E-E-RQ-R------------------------- 109
Pongo abelii CXNM                   79 VLQVIFV-SS-PSLV-YMGHAL-YRL-RVLE-E-E-RE-R------------------------- 109
Nomascus leucogenys CXNM            79 VLQVIFV-SS-PSLV-YMGHAL-YRL-RVLE-E-E-RQ-R------------------------- 109
Macaca mulatta CXNM                 79 VLQVIFV-SS-PSLV-YMGHAL-YRL-RVLE-E-E-RQ-R------------------------- 109
Callithrix jacchus CXNM             79 VLQVILV-SS-PSLV-YMGHAL-YRL-RVLE-K-E-RQ-R------------------------- 109
Tarsius syrichta CXNM               79 VLQVIFV-SS-PSLV-YMGHAL-YRL-RILE-E-E-RQ-R------------------------- 109
Microcebus murinus CXNM             79 VLQVIFV-SS-PSLV-YMGHAL-YRL-RVLE-E-E-RQ-R------------------------- 109
Dipodomys ordii CXNM                79 VLQVIFV-SS-PSLV-YMGHAL-YRL-RALQ-K-E-RQ-R------------------------- 109
Oryctolagus cuniculus CXNM          79 ALQMVFV-SS-PSLC-YMGHAL-YRL-RVLG-K-E-RR-R------------------------- 109
Equus caballus CXNM                 79 VLQVIFV-SS-PSLV-YVGHAL-YRL-RILE-K-E-RQ-R------------------------- 109
Canis lupus familiaris CXNM          79 VLQVIFV-SS-PSLV-YMGHAL-YRL-RVLE-K-E-RQ-R------------------------- 109
Pteropus vampyrus CXNM              79 VLQVIFV-SS-PSLV-YMGHAL-YQL-RVLE-K-E-RQ-R------------------------- 109
Loxodonta africana CXNM             79 VLQVIFV-SS-PSLV-YMGHAL-YRL-RVLE-K-E-RQ-E------------------------- 109
```

```
Homo sapiens CXNN              79 VLQIIFV-SS-PSLV-YMGHAL-YRL-RAFE-K-D-RQ-R------------------------- 109
Pan troglodytes CXNN           79 VLQIIFV-SS-PSLV-YMGHAL-YRL-RAFE-K-D-RQ-R------------------------- 109
Pongo abelii CXNN              79 VLQIIFV-SS-PSLV-YMGHAL-YRL-RAFE-K-E-RQ-R------------------------- 109
Nomascus leucogenys CXNN       79 VLQIIFV-SS-PSLV-YMGHAL-YRL-RAFE-K-E-RQ-R------------------------- 109
Macaca mulatta CXNN            79 VLQIIFV-SS-PSLV-YMGHAL-YRL-RAFE-K-E-RQ-R------------------------- 109
Callithrix jacchus CXNN        79 VLQIIFV-SS-PSLV-YMGHAL-YRL-RAFE-K-E-RQ-R------------------------- 109
Microcebus murinus CXNN        79 VLQIIFV-SS-PSLV-YMGHAL-YRL-RAFE-K-E-RQ-R------------------------- 109
Mus musculus Cxnn              79 VLQIIFV-SS-PSLV-YMGHAL-YRL-RDFE-K-Q-RQ-K------------------------- 109
Rattus norvegicus Cxnn         79 VLQIIFV-SS-PSLV-YMGHAL-YRL-RDFE-K-Q-RQ-K------------------------- 109
Oryctolagus cuniculus CXNN    109 VLQIIFV-SS-PSLV-YMGHAL-YRL-RDFE-K-E-RQ-R------------------------- 139
Bos taurus CXNN                79 VLQIIFV-SS-PSLV-YMGHAL-YRL-RAFE-K-E-RQ-R------------------------- 109
Equus caballus CXNN            79 VLQIIFV-SS-PSLV-YMGHAL-YRL-RAFE-K-E-RQ-R------------------------- 109
Canis lupus familiaris CXNN    79 VLQIIFV-SS-PSLV-YMGHAL-YRL-RAFE-K-E-RQ-R------------------------- 109
Sorex araneus CXNN             79 VLQIIFV-SS-PSLV-YMGHAL-YRL-RTFE-K-E-RQ-K------------------------- 109
Loxodonta africana CXNN        79 VLQIIFV-SS-PSLV-YMGHAL-YRL-RALE-K-E-RW-R------------------------- 109
Homo sapiens CXNO              81 VFQIVVI-ST-PSVM-YLGYAV-HRLARASE-Q-E-RR-R------------------------- 112
Papio hamadryas CXNO           81 VFQIVVI-ST-PSVM-YLGYAV-HRLARASE-Q-E-RR-R------------------------- 112
Mus musculus Cxno              92 VFQIVVI-ST-PSVM-YLGYAV-HRLARASE-Q-E-RR-R------------------------- 123
Cavia porcellus CXNO           95 VFQIVVI-ST-PSVL-YLGYAV-HRLARASE-Q-E-RK-R------------------------- 126
Homo sapiens CXNP1             78 VFQVILV-AV-PSAL-YMGFTLYHVIWHWEL-SGK-GK--------------------------- 110
Pan troglodytes CXNP1          78 VFQVILV-AV-PSAL-YMGFTLYHVIWHWEL-SGK-GK--------------------------- 110
Pongo abelii CXNP1             78 VFQVILV-AV-PSTL-YMGFTLYHVIWHWEL-SGK-GK--------------------------- 110
Callithrix jacchus CXNP1       78 VFQVILV-AV-PSAL-YMGFTLYHVIWHWEE-SGK-GK-G------------------------- 111
Otolemur garnettii CXNP1       78 TLQVMLV-AV-PSAL-YMGFTLYHVIWHWEE-SEK-VK-K------------------------- 111
Tupaia belangeri CXNP1         96 AFQIFLV-AV-PSAL-YMGFTLYHVIWHCEE-SGK-GK-K------------------------- 129
Mus musculus Cxnp1             78 AFQVILM-AV-PSAI-YVAFTLYHVIGYWEV-PGK-EN-K------------------------- 111
Rattus norvegicus Cxnp1        78 AFQVILM-AV-PSAI-YVAFTLYHVIGYWEV-PGR--N-K------------------------- 110
Cavia porcellus CXNP1          78 AFQVILV-AV-PSAL-YVGFTLCHVIEHWEE-PRK-VK-K------------------------- 111
Oryctolagus cuniculus CXNP1    78 AFQVILV-AV-PSAL-YMVFVLYQVIWHYEE-PGK-VK-K------------------------- 111
Oryctolagus cuniculus CXNP2    78 AFHVTLV-TV-PSAL-YMGFILYHVIWHWEE-SGK-LK-K------------------------- 111
Ochotona princeps CXNP1        78 AFQVTLV-TV-PSAL-YVGFVLYHVIWRWEI-SGK-LK-K------------------------- 111
Bos taurus CXNP1               80 AFQVTLV-AV-PSAL-YMGFILYHVIWHWEA-SEK-VK-T------------------------- 113
Equus caballus CXNP1           78 AFQVILV-AV-PSIL-YVGFTLYHVIWHWEE-SEK-VK-K------------------------- 111
Canis lupus familiaris CXNP1   78 AFQVILV-AV-PSTL-YMGLILYHVIWRWEE-SGK-VK--------------------------- 110
Felis catus CXNP1             100 VFQVILV-AV-PSAL-YMGLTLYHVIWLWEE-SGK-GK--------------------------- 132
Myotis lucifugus CXNP1         78 AFQVMLV-AV-PSVL-YMGFTMYHGIWHWED-SRK-VK-K------------------------- 111
Dasypus novemcinctus CXNP1     78 AFQVALA-AV-PGAL-YLAFTLYHVLWHWEE-PGK-AK--------------------------- 110
Dasypus novemcinctus CXNP2     78 AFQVALA-AV-PGAL-YLAFTLYHVLWHWEE-LEK-AK--------------------------- 110
Dasypus novemcinctus CXNP3     78 AFQVMLA-AV-PGAL-YLAFTLYHVLWHWEE-PGK-EK--------------------------- 110
Dasypus novemcinctus CXNP4     78 AFQVALA-AL-PGAL-YLAFTLYHVLWHWEE-PGK-EK--------------------------- 110
Choloepus hoffmanni CXNP1      78 AFQVILV-AV-PGAL-YLAFTLYHVVWHWED-LGK-AK-E------------------------- 111
Loxodonta africana CXNP1       78 AFQVMLV-AV-PSTL-YVGFTLYHVVWHWEE-SEK-VK-K------------------------- 111
Homo sapiens CXNQ              78 VFQIILV-AT-PSVM-YLGYAI-HKIAKM-E-HGEADK-K------------------------- 110
Pan troglodytes CXNQ           78 VFQIILV-AT-PSVM-YLGYAI-HKIAKM-E-HGEADK-K------------------------- 110
Pongo abelii CXNQ              78 VFQIILV-AT-PSVM-YLGYAI-HKIAKM-E-HGEADK-K------------------------- 110
Macaca mulatta CXNQ            78 VFQIILV-AT-PSVM-YLGYAI-HKIAKM-E-HGEADK-K------------------------- 110
Tupaia belangeri CXNQ          78 VFQIILV-AT-PSVM-YLGYAI-HKIAKM-E-HGEADK-K------------------------- 110
Mus musculus Cxnq              78 VFQIILV-AT-PSVM-YLGYAI-HKIAKM-E-HGEADK-K------------------------- 110
Rattus norvegicus Cxnq         78 VFQIILV-AT-PSVM-YLGYAI-HKIAKM-E-HGEADK-K------------------------- 110
Cavia porcellus CXNQ           78 VFQIILV-AT-PSVM-YLGYAI-HKIAKM-E-HGEADK-K------------------------- 110
S.tridecemlineatus CXNQ        92 VFQIILV-AT-PSVM-YLGYAI-HKIAKM-E-HGEADK-K------------------------- 124
Oryctolagus cuniculus CXNQ     78 VFQIILV-AT-PSVM-YLGYAI-HKIAKM-E-HGEADK-K------------------------- 110
Bos taurus CXNQ                78 VFQIILV-AT-PSVM-YLGYAI-HKIAKM-E-HGDADK-K------------------------- 110
Vicugna pacos CXNQ             78 VFQIILV-AT-PSVM-YLGYAI-HKIAKM-E-HGEADK-K------------------------- 110
Equus caballus CXNQ            78 VFQIILV-AT-PSVM-YLGYAI-HKIAKM-E-HGEADK-K------------------------- 110
Canis lupus familiaris CXNQ    78 VFQIILV-AT-PSVM-YLGYAI-HKIAKM-E-HGEADK-K------------------------- 110
Myotis lucifugus CXNQ          78 VFQIILV-AT-PSVM-YLGYAI-HKIAKM-E-HGEADK-K------------------------- 110
Pteropus vampyrus CXNQ         78 VFQIILV-AT-PSVM-YLGYAI-HKIAKM-E-HGEADK-K------------------------- 110
Loxodonta africana CXNQ        78 VFQIILV-AT-PSVM-YLGYAI-HKIAKM-E-HGAADK-K------------------------- 110
Homo sapiens CXNR              79 LFHILLL-SA-PPVL-FVVYSM-HRAGK--E-AGGAEA-AAQC--------A--------PGLPE 119
Pan troglodytes CXNR           79 LFHILLL-SA-PPVL-FVVYSM-HRAGK--E-AGGAEA-AAQC--------A--------PGLPE 119
Pongo abelii CXNR              79 LFHILLL-SA-PPVL-FVVYSM-HRAGK--E-AGGAEA-AAQC--------A--------PGLPE 119
Papio hamadryas CXNR           79 LFHILLL-SA-PPVL-FVVYSM-HRAGK--E-AGGAQA-AAQC--------A--------PGLPE 119
Mus musculus Cxnr              79 LFHILLL-SA-PPVL-FVIYSM-HQASK--E-AGGAQL-APPC--------A--------RGRAE 119
Rattus norvegicus Cxnr         79 LFHILLL-SA-PPVL-FVIYSM-HQASK--E-AGGAQP-APPC--------A--------RGRAE 119
Oryctolagus cuniculus CXNR     79 LFHILLL-SA-PPVL-FVIYSM-HQAGK--E-AGGAEA-AAAA--------AEAEAETETAVAAE 127
Bos taurus CXNR                79 LFHILLL-SA-PPVL-FVIYSV-HRASK--E-PGGADG-GAG----------------------- 112
Homo sapiens CXNS              29 VFQIIMVC-T-PS-LCFITYSV-HQSAKQRE-R-RYST-VFL---------ALDRDPPESIGGPG  77
Pan troglodytes CXNS           29 VFQIIMVC-T-PS-LCFITYSV-HQSAKQRE-R-RYST-VFL---------ALDRDPPESIGGPG  77
Pongo abelii CXNS              29 VFQIIMVC-T-PS-LCFITYSV-HQSAKQRE-R-RYST-VFL---------ALDRDPPESIGGPG  77
Nomascus leucogenys CXNS       29 VFQIIMVC-T-PS-LCFITYSV-HQSAKQRE-R-RYST-VFL---------ALDREPPESIGGPG  77
Macaca mulatta CXNS            29 VFQIIMVC-T-PS-LCFITYSV-HQSAKQRE-R-RYST-VFL---------ALDRDPPESIGGPG  77
Papio hamadryas CXNS           29 VFQIIMVC-T-PS-LCFITYSV-HQSAKQRE-R-RYST-VFL---------ALDRDPPESIGGPG  77
Otolemur garnettii CXNS        29 VFQIIMVC-T-PS-LCFITYSV-HQSAKQRE-R-RYST-VFL---------ALDRDPPESIGGPG  77
Tupaia belangeri CXNS          29 VFQIIMVC-T-PS-LCFITYSV-HQSAKQRE-R-RYST-VFL---------ALDRDPPESIGVPG  77
Mus musculus Cxns              69 VFQIIMVC-T-PS-LCFITYSV-HQSAKQRE-R-RYST-VFL---------ALDRDPAESIGGPG 117
Rattus norvegicus Cxns         29 VFQIIMVC-T-PS-LCFITYSV-HQSAKQRE-R-RYST-VFL---------ALDRDPAESIGGPG  77
Cavia porcellus CXNS           29 VFQIIMVC-T-PS-LCFITYSV-HQSAKQRE-R-RYST-VFL---------ALDRDAPESMGGPG  77
S.tridecemlineatus CXNS        29 VFQIIMVC-T-PS-LCFITYSV-HQSAKQRE-R-RYST-VFL---------ALDRDPPESIGGPG  77
```

```
Oryctolagus cuniculus CXNS     29 VFQIIMVC-T-PS-LCFITYSV-HQSAKQRE-R-RYST-VFL---------SLDRDPPDSMGGPG  77
Ochotona princeps CXNS         29 VFQIIMVC-T-PS-LCFITYSV-HQSAKQRE-R-RYST-VFL---------ALDRDPPESIGGPG  77
Bos taurus CXNS                29 VFQIIMVC-T-PS-LCFITYSV-HQSAKQRE-R-RYST-VFL---------ALDRDPPESMGGPG  77
Equus caballus CXNS            29 VFQIIMVC-T-PS-LCFITYSV-HQSAKQRE-R-RYST-VFL---------ALDRDAPESMGGPG  77
Canis lupus familiaris CXNS    29 VFQIIMVC-T-PS-LCFITYSV-HQSAKQRE-R-RYST-VFL---------ALDREPPESMGGPG  77
Myotis lucifugus CXNS          29 VFQIIMVC-T-PS-LCFITYSV-HQSAKQRE-R-RYST-VFL---------ALDRDPPESMGGPG  77
Dasypus novemcinctus CXNS      29 VFQIIMVC-T-PS-LCFITYSV-HQSAKQRE-R-RYST-VFL---------ALDRDPPESMGGPG  77
Loxodonta africana CXNS       157 VFQIIMVC-T-PS-LCFITYSV-HQSAKQRE-R-RYST-VFL---------ALDRDPPESMGGPG 205
Homo sapiens CXNT              79 ALQLVIV-LV-PGAL-FHLY---------AACK-SINQ---EC---------------------- 105
Pan troglodytes CXNT           79 ALQLVIV-LV-PGAL-FHLY---------AACK-SINQ---EC---------------------- 105
Nomascus leucogenys CXNT       82 ALQLVIV-LV-PGAI-FHLY---------AACK-SINQ---EC---------------------- 108
Mus musculus Cxnt              82 ALQLVIV-LL-PGAI-FHLY---------AACK-SINQ---DC---------------------- 108
Rattus norvegicus Cxnt         82 ALQLVIV-LL-PGAI-FHLY---------AACK-SINQ---EC---------------------- 108
Cavia porcellus CXNT           78 ALQLVIV-LV-PGAI-FHLY---------AACK-SINQ---EC---------------------- 104
Oryctolagus cuniculus CXNT     82 ALQLVIV-LV-PGAI-FHLY---------AACK-SINQ---EC---------------------- 108
Bos taurus CXNT                82 ALQLVIV-LV-PGAI-FHLY---------AACK-SINQ---EC---------------------- 108
Vicugna pacos CXNT             82 ALQLVIV-LV-PGAI-FHLY---------AACK-SINQ---EC---------------------- 108
Equus caballus CXNT            82 ALQLVIV-LV-PGAI-FHLY---------AACK-SINQ---EC---------------------- 108
Canis lupus familiaris CXNT    82 ALQLVIV-LV-PGAI-FHLY---------AACK-SINQ---EC---------------------- 108
Myotis lucifugus CXNT          82 ALQLVIV-LV-PGAT-FHVY---------AAFK-SISQ---EC---------------------- 108
Dasypus novemcinctus CXNT      82 ALQLVIV-LA-PGAI-FHLY---------AACK-DISH---EC---------------------- 108
Loxodonta africana CXNT        78 ALQLVIV-LV-PGAI-FHLY---------AAGK-SISQ---EC---------------------- 104
Homo sapiens CXNU              65 LIQGVCV-LL-PSAV-FSVYVL-HRGATLAA-LGP-RR----C--------PDPREPA-S-G--Q 107
Pan troglodytes CXNU           65 LIQGVCV-LL-PSAV-FSVYVL-HRGATLAA-LGP-RR----C--------PDPREPA-S-G--Q 107
Pongo abelii CXNU              50 LIQGVCV-LL-PSAV-FSVYVL-HRGATLAA-LGP-SR----C--------PDPRDPA-S-G--Q  92
Nomascus leucogenys CXNU       65 LIQGVCV-LL-PSAV-FSVYVL-HRGAALAA-LGP-RR----F--------PDPRDPA------- 104
Macaca mulatta CXNU            65 LIQGVCV-LL-PSAV-FSVYVL-HRGATLAA-LGP-RR----C--------PEPRDTA-S-G--Q 107
Mus musculus Cxnu              57 LVQSLAL-LL-PSVV-FGTYTL-HRGAKLAA-VGG-A-----C----------------------  88
Cavia porcellus CXNU           46 LVQGVSV-LL-PYAM-FSVYVL-HKSTQLAT-WEG-------C----------------------  76
Bos taurus CXNU                51 LIQSVSV-LL-PSAI-FGVYVL-HKGAELAA-RRS-R------GLEDASEDHDAPGLT-P-G--A  99
Equus caballus CXNU            50 LIQSVSV-LL-PSAV-FSVYVL-HRGVVLAA-RGP-------CRPECRPEGHDPSDLT-P-G--D  98
Myotis lucifugus CXNU          66 LVQSVSV-LL-PSVV-FSVYVL-HKGAELAA-RGP-------CGPDGGSGSQDLADLS-P-R--D 114
Sorex araneus CXNU             51 LLQSVSA-LL-PSAV-FLVYVL-HQGAALAA-GLG-GH-----GPSHLP----------------  88
Dasypus novemcinctus CXNU      77 LAQSVAV-LL-PHAL-FSVYVL-HKGTARAA-GGP-------CGEEGCAAGHGLADQA-T-G--- 124
Loxodonta africana CXNU        77 LIQSVSV-LF-PYAV-FSVYVL-HKGAMHAA-AGG-------CPPDGGQGGPTPWDLT-T-A--E 125
```

```
Homo sapiens CXNA                 107 ---------------------------------------EA-HGEN------S-GR-------- 115
                                        330       340       350       360       370       380       390
Homo sapiens CXNA                 107 ---------------------------------------EA-HGEN------S-GR-------- 115
Pan troglodytes CXNA              107 ---------------------------------------EA-HGEN------S-GR-------- 115
Gorilla gorilla CXNA              107 ---------------------------------------EA-HGEN------S-GR-------- 115
Pongo abelii CXNA                 107 ---------------------------------------EA-HGEN------S-GR-------- 115
Nomascus leucogenys CXNA          107 ---------------------------------------EA-HGEN------S-GR-------- 115
Macaca mulatta CXNA               107 ---------------------------------------EA-HGEN------S-GR-------- 115
Callithrix jacchus CXNA           107 ---------------------------------------EA-YGED------G-GR-------- 115
Mus musculus Cxna                 107 ---------------------------------------EK-IGE---------GY-------- 113
Rattus norvegicus Cxna            107 ---------------------------------------QE-VGK---------GY-------- 113
Cavia porcellus CXNA              157 ---------------------------------------EA-VGED------G-GR-------- 165
Ochotona princeps CXNA            107 ---------------------------------------EI-VGKD------G-GR-------- 115
Bos taurus CXNA                   107 ---------------------------------------EA-IGKD------G-GR-------- 115
Equus caballus CXNA               107 ---------------------------------------EA-VGQD------G-GR-------- 115
Canis lupus familiaris CXNA       107 ---------------------------------------QA-AGEG------G-GR-------- 115
Felis catus CXNA                  107 ---------------------------------------EA-VGED------G-GR-------- 115
Myotis lucifugus CXNA             139 ---------------------------------------EA-VGED------G-GR-------- 147
Dasypus novemcinctus CXNA         107 ---------------------------------------EA-AGEG------T-RH-------- 115
Loxodonta africana CXNA           107 ---------------------------------------EA-AGEG------S-GR-------- 115
Homo sapiens CXNB                 107 ---------------------------------------LK-HGPN------A-PS-------- 115
Gorilla gorilla CXNB              107 ---------------------------------------LK-HGPN------A-PS-------- 115
Nomascus leucogenys CXNB          107 ---------------------------------------LK-HGPN------A-PS-------- 115
Macaca mulatta CXNB               107 ---------------------------------------LK-HGPN------A-PS-------- 115
Callithrix jacchus CXNB           107 ---------------------------------------LK-HGPN------A-PS-------- 115
Otolemur garnettii CXNB           107 ---------------------------------------LK-HGPN------A-RS-------- 115
Mus musculus Cxnb                 107 ---------------------------------------LK-HGPN------A-PA-------- 115
Rattus norvegicus Cxnb            107 ---------------------------------------LK-HGPD------A-PA-------- 115
Dipodomys ordii CXNB              107 ---------------------------------------EK-HGPN------A-PS-------- 115
Cavia porcellus CXNB              107 ---------------------------------------MK-RGPN------A-PP-------- 115
Oryctolagus cuniculus CXNB         15 ---------------------------------------LK-HGPN------A-PA--------  23
Ochotona princeps CXNB            107 ---------------------------------------LK-HGPN------A-PP-------- 115
Bos taurus CXNB                   107 ---------------------------------------RK-HGPH------A-PS-------- 115
Equus caballus CXNB               107 ---------------------------------------LK-HGPN------A-PS-------- 115
Canis lupus familiaris CXNB       157 ---------------------------------------LK-HGPG------A-PS-------- 165
Myotis lucifugus CXNB             107 ---------------------------------------LK-HGPN------A-PS-------- 115
Erinaceus europaeus CXNB          107 ---------------------------------------LK-HGPN------A-RS-------- 115
Loxodonta africana CXNB           107 ---------------------------------------LK-HGPN------A-RS-------- 115
Homo sapiens CXNC                 107 ---------------------------------------QK-HGDQC-----A--K-------- 115
Gorilla gorilla CXNC              107 ---------------------------------------QK-HGDQC-----A--K-------- 115
Pongo abelii CXNC                 107 ---------------------------------------QK-HGDQC-----A--K-------- 115
Nomascus leucogenys CXNC          107 ---------------------------------------QK-HGDQC-----A--K-------- 115
Callithrix jacchus CXNC           107 ---------------------------------------QK-HGDQC-----S--K-------- 115
Microcebus murinus CXNC           137 ---------------------------------------LK-HGDQC-----A--K-------- 145
Otolemur garnettii CXNC           107 ---------------------------------------LK-HGDQC-----A--K-------- 115
Mus musculus Cxnc                 107 ---------------------------------------QK-HGEQC-----A--K-------- 115
Rattus norvegicus Cxnc            107 ---------------------------------------QK-HGEHC-----A--K-------- 115
Cavia porcellus CXNC              107 ---------------------------------------QK-YGEQC-----T--K-------- 115
Oryctolagus cuniculus CXNC        217 ---------------------------------------QK-HGDQC-----A--K-------- 225
Bos taurus CXNC                   107 ---------------------------------------QK-HGDQC-----T--K-------- 115
Equus caballus CXNC               107 ---------------------------------------LK-HGDQC-----A--K-------- 115
Canis lupus familiaris CXNC       107 ---------------------------------------LK-HGDQC-----A--R-------- 115
Felis catus CXNC                  107 ---------------------------------------QK-HGDQC-----A--K-------- 115
Myotis lucifugus CXNC             107 ---------------------------------------LK-HGDQC-----T--K-------- 115
Dasypus novemcinctus CXNC         107 ---------------------------------------QK-HGEQC-----A--K-------- 115
Loxodonta africana CXNC           107 ---------------------------------------QK-HGEQC-----A--K-------- 115
Homo sapiens CXND                 107 ---------------------------------------KK----------------------- 108
Pan troglodytes CXND              107 ---------------------------------------KK----------------------- 108
Macaca mulatta CXND               107 ---------------------------------------KK----------------------- 108
Callithrix jacchus CXND           107 ---------------------------------------KK----------------------- 108
Dipodomys ordii CXND              107 ---------------------------------------RK----------------------- 108
Oryctolagus cuniculus CXND        107 ---------------------------------------KK----------------------- 108
Tursiops truncatus CXND           107 ---------------------------------------KK----------------------- 108
Bos taurus CXND                    25 ---------------------------------------KK-----------------------  26
Equus caballus CXND               126 ---------------------------------------KK----------------------- 127
Canis lupus familiaris CXND       107 ---------------------------------------KK----------------------- 108
Myotis lucifugus CXND             107 ---------------------------------------KK----------------------- 108
Erinaceus europaeus CXND          107 ---------------------------------------KK----------------------- 108
Dasypus novemcinctus CXND         107 ---------------------------------------KK----------------------- 108
Choloepus hoffmanni CXND          107 ---------------------------------------KK----------------------- 108
Loxodonta africana CXND           107 ---------------------------------------KK----------------------- 108
Homo sapiens CXNE                 106 ---------------------------------------FI-KGEI-----KSEFKDIEE---- 120
Pan troglodytes CXNE              106 ---------------------------------------FI-KGEI-----KSEFKDIEE---- 120
Pongo abelii CXNE                 106 ---------------------------------------FI-KGEI-----KSEFKDIEE---- 120
Macaca mulatta CXNE               106 ---------------------------------------FI-KGEI-----KNEFKDIEE---- 120
Papio hamadryas CXNE              106 ---------------------------------------FI-KGEI-----KNEFKDIEE---- 120
Callithrix jacchus CXNE           106 ---------------------------------------FI-KGEI-----KSEFKDIEE---- 120
Otolemur garnettii CXNE           106 ---------------------------------------FI-KGEI-----KTEFKDIEE---- 120
Mus musculus Cxne                 119 ---------------------------------------FM-KGEI-----KNEFKDIEE---- 133
```

```
Rattus norvegicus Cxne        125 ----------------------------------------FM-KGEI-----KNEFKDIEE---- 139
Dipodomys ordii CXNE          125 ----------------------------------------FI-KGEI-----KNEYKDIEE---- 139
Cavia porcellus CXNE          153 ----------------------------------------FI-KGEM-----KSEFKDIEE---- 167
Oryctolagus cuniculus CXNE    125 ----------------------------------------FM-KGET-----KNEFKDIEE---- 139
Equus caballus CXNE           106 ----------------------------------------FI-KGEI-----KSEFKDIEE---- 120
Canis lupus familiaris CXNE   106 ----------------------------------------FI-KGEI-----KSEFKDIEE---- 120
Felis catus CXNE              106 ----------------------------------------FI-KGEI-----KNEFKDIEE---- 120
Myotis lucifugus CXNE         106 ----------------------------------------FI-KGEI-----KSEYKDIEE---- 120
Sorex araneus CXNE            106 ----------------------------------------FI-KGEI-----KSEFKDLEE---- 120
Dasypus novemcinctus CXNE     106 ----------------------------------------FI-KGET-----KTEFKDLEE---- 120
Loxodonta africana CXNE       106 ----------------------------------------FI-KGEI-----KSEFKDIEE---- 120
Homo sapiens CXNF             106 ----------------------------------------FR-RGEK-----RNDFKDIED---- 120
Pan troglodytes CXNF          106 ----------------------------------------FR-RGEK-----RNDFKDIED---- 120
Gorilla gorilla CXNF          106 ----------------------------------------FR-RGEK-----RNDFKDIED---- 120
Nomascus leucogenys CXNF      106 ----------------------------------------FR-RGEK-----RNEFKDIED---- 120
Callithrix jacchus CXNF       106 ----------------------------------------FR-RGEK-----RNEFKDLED---- 120
Mus musculus Cxnf             106 ----------------------------------------FI-RGEK-----RNEFKDLED---- 120
Rattus norvegicus Cxnf        106 ----------------------------------------FI-RGEK-----RNEFKDLED---- 120
S.tridecemlineatus CXNF       106 ----------------------------------------FR-KGEK-----RNEFKDLED---- 120
Oryctolagus cuniculus CXNF    106 ----------------------------------------FR-RGEK-----RNEFKDLED---- 120
Ochotona princeps CXNF        106 ----------------------------------------FQ-RGEK-----RNEFKDLED---- 120
Bos taurus CXNF               106 ----------------------------------------FR-RGET-----RSEFKDLED---- 120
Equus caballus CXNF           106 ----------------------------------------FR-RGEK-----RNEFKDLED---- 120
Canis lupus familiaris CXNF   106 ----------------------------------------FR-RGEK-----RNEFKDLED---- 120
Myotis lucifugus CXNF         106 ----------------------------------------FR-RGEK-----RNEFKDLED---- 120
Dasypus novemcinctus CXNF     106 ----------------------------------------FR-RGEK-----RKEFKDIEE---- 120
Choloepus hoffmanni CXNF      106 ----------------------------------------FR-RGER-----KSEFKDIED---- 120
Loxodonta africana CXNF       106 ----------------------------------------FR-RGEK-----RNEFKDIED---- 120
Homo sapiens CXNG             105 ----------------------------------------ML-RLEG-----HGDPLHLEE---- 119
Pan troglodytes CXNG          105 ----------------------------------------ML-RLEG-----HGDPLHLEE---- 119
Gorilla gorilla CXNG          105 ----------------------------------------ML-RLEG-----HGDPLHLEE---- 119
Pongo abelii CXNG             105 ----------------------------------------ML-RLEG-----HGDPLHLEE---- 119
Nomascus leucogenys CXNG      105 ----------------------------------------ML-RLEG-----HGDPLHLEE---- 119
Macaca mulatta CXNG           105 ----------------------------------------ML-RLEG-----HGDPLHLEE---- 119
Papio hamadryas CXNG          105 ----------------------------------------ML-RLEG-----HGDPLHLEE---- 119
Callithrix jacchus CXNG       105 ----------------------------------------ML-RLEG-----HGDPLHLEE---- 119
Microcebus murinus CXNG       105 ----------------------------------------ML-RLEG-----HGDPLHLEE---- 119
Mus musculus Cxng             105 ----------------------------------------ML-RLEG-----HGDPLHLEE---- 119
Rattus norvegicus Cxng        105 ----------------------------------------ML-RLEG-----HGDPLHLEE---- 119
Oryctolagus cuniculus CXNG    105 ----------------------------------------ML-RLEG-----HVDPLQLEE---- 119
Bos taurus CXNG               105 ----------------------------------------ML-RLEG-----HGDPLHLEE---- 119
Equus caballus CXNG           105 ----------------------------------------ML-RLEG-----HGDPIHLEE---- 119
Canis lupus familiaris CXNG   105 ----------------------------------------ML-RLEG-----HGDPLHLEE---- 119
Felis catus CXNG              105 ----------------------------------------ML-RLEG-----HGDPLHLEE---- 119
Myotis lucifugus CXNG         105 ----------------------------------------ML-RLEG-----HGDPLHLEE---- 119
Pteropus vampyrus CXNG        105 ----------------------------------------ML-RLEG-----HGDPLHMEE---- 119
Dasypus novemcinctus CXNG     105 ----------------------------------------ML-RLEG-----HGDPLHLEE---- 119
Loxodonta africana CXNG       105 ----------------------------------------ML-RLEG-----HGDPLHLEE---- 119
Procavia capensis CXNG        105 ----------------------------------------ML-RLEG-----HGDPLHLEE---- 119
Homo sapiens CXNH1            110 ----------------------------------------EGE-LRA---LPAKD-PQVE--R-A 126
Pan troglodytes CXNH1         110 ----------------------------------------EGE-LRA---LPAKD-PQVE--R-A 126
Gorilla gorilla CXNH1         110 ----------------------------------------EGE-LRA---LPAKD-PQVE--Q-A 126
Pongo abelii CXNH1            110 ----------------------------------------EGE-LRA---LPAKN-PQVE--R-A 126
Nomascus leucogenys CXNH1     110 ----------------------------------------EGE-LRA---LPAKD-PQVE--R-A 126
Macaca mulatta CXNH1          110 ----------------------------------------EGE-LRA---LPAKD-PQVE--R-A 126
Otolemur garnettii CXNH1      120 ----------------------------------------KKE---------------VI--Q-A 126
Mus musculus Cxnh1            110 ----------------------------------------EGE-LRA---LPSKD-LHVE--R-A 126
Rattus norvegicus Cxnh1       110 ----------------------------------------EGE-LRA---LPSKD-PHVE--R-A 126
Cavia porcellus CXNH1         110 ----------------------------------------EGE-LRA---LPAKD-PQVE--Q-E 126
Oryctolagus cuniculus CXNH1   110 ----------------------------------------EGE-LRA---LPAKD-PQVE--R-A 126
Ochotona princeps CXNH1       124 ----------------------------------------EGE-LRA---LPAKD-PHVE--R-E 140
Equus caballus CXNH1          110 ----------------------------------------EGE-LRA---LSAKD-PRVQ--R-A 126
Canis lupus familiaris CXNH1  110 ----------------------------------------EGE-LRA---LPAKD-PRVE--R-A 126
Felis catus CXNH1             110 ----------------------------------------EGE-LRA---LPAKD-PRVE--R-A 126
Myotis lucifugus CXNH1        142 ----------------------------------------EEE-LRA---LPHKD-PCVE--R-T 158
Dasypus novemcinctus CXNH1    110 ----------------------------------------ERE-LRA---LPAKD-PHVE--R-A 126
Loxodonta africana CXNH1      110 ----------------------------------------EGE-LRA---LPAKD-PQVE--R-A 126
Homo sapiens CXNI             110 ----------------------------------------EAERAK--------EV-RGSG-S-Y 123
Pan troglodytes CXNI          110 ----------------------------------------EAERAK--------EV-RGSG-S-Y 123
Pongo abelii CXNI             110 ----------------------------------------EAERAK--------EV-RGSD-S-Y 123
Nomascus leucogenys CXNI      110 ----------------------------------------EAERAK--------EV-QGSG-S-Y 123
Macaca mulatta CXNI           110 ----------------------------------------EAERAK--------EV-QRSG-S-Y 123
Papio hamadryas CXNI          110 ----------------------------------------EAERAK--------EV-QRSG-S-Y 123
Callithrix jacchus CXNI       110 ----------------------------------------EAQKGR--------EG-GGSG-S-Y 123
Otolemur garnettii CXNI       110 ----------------------------------------EAERAR--------DS-RGAG-S-Y 123
Mus musculus Cxni             110 ----------------------------------------DAEKAK--------EA-HRTG-A-Y 123
Rattus norvegicus Cxni        144 ----------------------------------------EAEKAK--------EA-GGTG-T-Y 157
Cavia porcellus CXNI          110 ----------------------------------------EAERAK--------EI-HRPG-SPY 124
Oryctolagus cuniculus CXNI    110 ----------------------------------------EAETGK--------EV-QGAG-S-Y 123
```

```
Bos taurus CXNI                 111 ----------------------------------------EAERAK--------EA-RAAG-S-Y 124
Equus caballus CXNI             110 ----------------------------------------EAERAK--------HV-QGAG-S-Y 123
Canis lupus familiaris CXNI     110 ----------------------------------------EAERAK--------EA-RGAG-S-Y 123
Felis catus CXNI                110 ----------------------------------------EAERAK--------EV-RGAG-S-Y 123
Pteropus vampyrus CXNI          110 ----------------------------------------EAERAR--------EA-RGAG-P-C 123
Erinaceus europaeus CXNI        110 ----------------------------------------EAERAR--------EA-QGAG---Y 122
Dasypus novemcinctus CXNI       110 ----------------------------------------EAERTK--------EV-RGPG-A-Y 123
Loxodonta africana CXNI         110 ----------------------------------------EAERAK--------GV-QGSG-S-Y 123
Homo sapiens CXNJ1              110 --------------------------------------E-EEEQLK-------RESPSP-----K 123
Pongo abelii CXNJ1              110 --------------------------------------E-EEEQLK-------RDSPSP-----K 123
Macaca mulatta CXNJ1            110 --------------------------------------Q-EEEPPK-------TDSPSP-----P 123
Mus musculus Cxnj1              110 ----------------------------------------EEELLR-------RDNPQHG--RGR 125
Rattus norvegicus Cxnj1         110 ----------------------------------------EEELLR-------RDNPQHG--RGR 125
Bos taurus CXNJ1                110 ----------------------------------------EEEPPK-------AAGPEGH----- 122
Equus caballus CXNJ1            110 ----------------------------------------EEEPLK-------GDNLHHEP-AGP 126
Myotis lucifugus CXNJ1          110 ----------------------------------------EEEQAK-------GESDHG------ 121
Myotis lucifugus CXNJ2          110 ----------------------------------------EEEKLK-------GGSLHLRP-DGQ 126
Pteropus vampyrus CXNJ1         279 ----------------------------------------DAEQPN-------GDGDRG------ 290
Pteropus vampyrus CXNJ2         201 ----------------------------------------DAEQPN-------GDGDRG------ 212
Sorex araneus CXNJ1             110 ----------------------------------------EEAPPQ-------ARGPPG------ 121
Homo sapiens CXNK1              110 ----------------------------------------EEE-LKV----AQTDGVNVEM-HLK 128
Homo sapiens CXNK2              110 ----------------------------------------EEE-LKV----AQTDGVNVDM-HLK 128
Pan troglodytes CXNK1           110 ----------------------------------------EEE-LKV----AQTDGVNVEI-HLK 128
Pan troglodytes CXNK2           110 ----------------------------------------EEE-LKV----AQTDGVNVDM-HLK 128
Pongo abelii CXNK1              110 ----------------------------------------EEE-LKV----AQTDGANVDM-HLK 128
Nomascus leucogenys CXNK1       110 ----------------------------------------EEE-LKV----AQTDGANVDM-HLK 128
Callithrix jacchus CXNK1        110 ----------------------------------------EEE-LKV----AQTDGVNVEM-HLK 128
Mus musculus Cxnk1              110 ----------------------------------------EEE-LKV----AQTDGVNVEM-HLK 128
Mus musculus Cxnk2              110 ----------------------------------------EEE-LEA----AHFNEASVER-HLE 128
Rattus norvegicus Cxnk1         110 ----------------------------------------EEE-LKV----AQTDGVNVEM-HLK 128
Rattus norvegicus Cxnk2         110 ----------------------------------------EEEELKV----AHFNGASGER-RLQ 129
Cavia porcellus CXNK1           110 ----------------------------------------EEE-LKV----AQTDGANVEM-HLK 128
Oryctolagus cuniculus CXNK1     110 ----------------------------------------EEE-LKV----AQTDGVNVEM-HLK 128
Bos taurus CXNK1                110 ----------------------------------------EEE-LKV---VAQTDGANVDM-HLK 129
Vicugna pacos CXNK1             110 ----------------------------------------EEE-LKV----AQTDGANVDM-HLK 128
Equus caballus CXNK1            110 ----------------------------------------EEE-LKV----AQTDGVNVEM-HLK 128
Equus caballus CXNK2             87 ----------------------------------------EEE-HKV----AQTNGVNVEM-RLQ 105
Canis lupus familiaris CXNK1    110 ----------------------------------------EEE-LKV----AQTDGANVDM-HLK 128
Canis lupus familiaris CXNK2    110 ----------------------------------------GEE-LRA----AQTSGVNMEV-HLQ 128
Felis catus CXNK1               110 ----------------------------------------GEE-LTV----AQTNDVNVE--EIR 127
Myotis lucifugus CXNK1          110 ----------------------------------------EEE-LKV----AQTDGMNVDM-HLK 128
Dasypus novemcinctus CXNK1      110 ----------------------------------------EEE-LKV----AQTSVVSVEM-HLQ 128
Dasypus novemcinctus CXNK2      110 ----------------------------------------EEE-LKV----AQTDGANVDM-HLK 128
Loxodonta africana CXNK1        110 ----------------------------------------EEE-LKV----AQTDGANVDM-HLK 128
Loxodonta africana CXNK2        110 ----------------------------------------EEE-LKV----VQTEGVSVDM-RSQ 128
Homo sapiens CXNL               110 ----------------------------------------EAEELG-----QQA-GTNG-G---- 123
Pan troglodytes CXNL            110 ----------------------------------------EAEELG-----QQA-GTNG-G---- 123
Pongo abelii CXNL               110 ----------------------------------------EAEELG-----QQA-GTNG-G---- 123
Nomascus leucogenys CXNL        110 ----------------------------------------EAEELG-----QQA-GTNG-G---- 123
Macaca mulatta CXNL             110 ----------------------------------------EAEELG-----QQA-GANG-GERGP 127
Callithrix jacchus CXNL         110 ----------------------------------------EAEQLG-----QQA-GTDG--ERGP 126
Otolemur garnettii CXNL         155 ----------------------------------------EAEELS-----QQA--ADG-GERGP 171
Mus musculus Cxnl               110 ----------------------------------------EAEELC-----QQS-RSNG-GERVP 127
Rattus norvegicus Cxnl          110 ----------------------------------------EAEELC-----QQS-RSNG-GERVP 127
Cavia porcellus CXNL            110 ----------------------------------------EAEELC-----QQS-RSNG-CERGT 127
Oryctolagus cuniculus CXNL      110 ----------------------------------------EAEELC-----QQS-RGDG-SERVP 127
Ochotona princeps CXNL          110 ----------------------------------------EAEE--------QS-RGDG-SERVP 124
Bos taurus CXNL                 110 ----------------------------------------EAEELS-----QQS-PGNG-GERAP 127
Equus caballus CXNL             110 ----------------------------------------EAEELG-----QQP-PGNG--ERAP 126
Canis lupus familiaris CXNL     110 ----------------------------------------EAEELC-----QQA-AGDG-GDRVP 127
Felis catus CXNL                110 ----------------------------------------EADELC-----QQG-AAEG-GDRAP 127
Pteropus vampyrus CXNL          110 ----------------------------------------EAEELC-----QQA-AGDS-GERGP 127
Sorex araneus CXNL                - -----------------------------------------------------------------   -
Dasypus novemcinctus CXNL       110 ----------------------------------------EAEELC-----QQS-GGNG-GDRAP 127
Loxodonta africana CXNL         110 ----------------------------------------EAEELC-----QQP-GGNG-PEKGP 127
Homo sapiens CXNM               110 ----------------------------------------MKAQLRV-----ELEEVEFEM---- 125
Pan troglodytes CXNM            110 ----------------------------------------MKAQLRV-----ELEEVEFEM---- 125
Pongo abelii CXNM               110 ----------------------------------------MKAQLRV-----ELEEVEFEM---- 125
Nomascus leucogenys CXNM        110 ----------------------------------------MKAQLRV-----ELEEVEFEM---- 125
Macaca mulatta CXNM             110 ----------------------------------------MKAQLRV-----ELEGVEFEM---- 125
Callithrix jacchus CXNM         110 ----------------------------------------MKAQLRV-----ELEE-ESEM---- 124
Tarsius syrichta CXNM           110 ----------------------------------------MKAQLRG-----ELEWVEFEM---- 125
Microcebus murinus CXNM         110 ----------------------------------------MKAQLRG-----ELEGVEFEM---- 125
Dipodomys ordii CXNM            110 ----------------------------------------LKAQLKK-----ELEEVEFKM---- 125
Oryctolagus cuniculus CXNM      110 ----------------------------------------MKAHL-------EPERVGFET---- 123
Equus caballus CXNM             110 ----------------------------------------KKAQLRG-----ELEGVEFEM---- 125
Canis lupus familiaris CXNM     110 ----------------------------------------RKAQLRG-----ALGGVGFEV---- 125
Pteropus vampyrus CXNM          110 ----------------------------------------EKAQLRG-----ELKGLEFEV---- 125
Loxodonta africana CXNM         110 ----------------------------------------MKTQLRR-----ELEEVEFEV---- 125
```

Homo sapiens CXNN	110	--KKSHLRA-----QMENPDLDL----	125
Pan troglodytes CXNN	110	--KKSHLRA-----QMENPDLDL----	125
Pongo abelii CXNN	110	--KKSHLRA-----QMENPELDL----	125
Nomascus leucogenys CXNN	110	--KKSHLRA-----QMENPELDL----	125
Macaca mulatta CXNN	110	--KKSHLRA-----QMENPELDL----	125
Callithrix jacchus CXNN	110	--KKSHLRA-----QMENPELDL----	125
Microcebus murinus CXNN	110	--KKSYLRA-----QMENPELDL----	125
Mus musculus Cxnn	110	--KKLYLRA-----QMENPELDL----	125
Rattus norvegicus Cxnn	110	--KKLYLKA-----QMENPDLDL----	125
Oryctolagus cuniculus CXNN	140	--KKSYLRA-----QMENPELDL----	155
Bos taurus CXNN	110	--KKSQLRA-----QMENPELEL----	125
Equus caballus CXNN	110	--KKSHLRA-----LMENPELEF----	125
Canis lupus familiaris CXNN	110	--KKSHLRV-----QMENPELEL----	125
Sorex araneus CXNN	110	--RKAHLRA-----HLEDPELEW----	125
Loxodonta africana CXNN	110	--KKSYLRT-----QMENPEVDL----	125
Homo sapiens CXNO	113	-------ALR--RRPGPRR-APRAHLPPPHAGWPEPADLGEEE-----PMLGLGEEEEEEETGAA	162
Papio hamadryas CXNO	113	-------ALR--RRPGPRR-APRAHLPPPHAGWPEPADLGEEE-----PMLGLGEEEEEEETGAA	162
Mus musculus Cxno	124	-------ALR--RRPGTRR-LPRAQLPPPPPGWPDTTDLGEAE-----PILAL-EEDEDEEPGAP	172
Cavia porcellus CXNO	127	-------ALR--RHPAPRR-VA-----PP--GWPEP---GEAE-----PMLGLGTGEDNEE----	162
Homo sapiens CXNP1	111	--EEE-----TLIQ--GREGNTDV---	125
Pan troglodytes CXNP1	111	--EEE-----TLIQ--GREGNTDV---	125
Pongo abelii CXNP1	111	--EEE-----TLIQ--GGEGNTDV---	125
Callithrix jacchus CXNP1	112	--EEE-----ALIQ--GDESSRDA---	126
Otolemur garnettii CXNP1	112	--EEE-----TLPH--EGQSSRDA---	126
Tupaia belangeri CXNP1	130	--EEE-----NLIQ--EGD-SRDG---	143
Mus musculus Cxnp1	112	--EQE-----TQIS--KGDHSKDV---	126
Rattus norvegicus Cxnp1	111	--EQE-----AQIC--KGGRCKDV---	125
Cavia porcellus CXNP1	112	--EEE-----TLIH--KGDSCRDA---	126
Oryctolagus cuniculus CXNP1	112	--EEE-----TLIY--QRDSSRDA---	126
Oryctolagus cuniculus CXNP2	112	--EEE-----ALVP--EGESSKDA---	126
Ochotona princeps CXNP1	112	--EEE-----TLAC--EGESSGDG---	126
Bos taurus CXNP1	114	--EEE-----TL-S--QGEKGGEA---	127
Equus caballus CXNP1	112	--EEE-----TLVR--QREDSRDA---	126
Canis lupus familiaris CXNP1	111	--E-E-----TLIH--QGEKSRDA---	124
Felis catus CXNP1	133	--EEE-----TLIR--RGEKSRDA---	147
Myotis lucifugus CXNP1	112	--EEE-----TLIR--QGQGSTDV---	126
Dasypus novemcinctus CXNP1	111	--EED-----TLAV--EGDGGRAA---	125
Dasypus novemcinctus CXNP2	111	--EED-----TLAV--EGDGGRAA---	125
Dasypus novemcinctus CXNP3	111	--EED-----TLAV--EGDGGRVA---	125
Dasypus novemcinctus CXNP4	111	--EED-----TLAI--EGDGGRAA---	125
Choloepus hoffmanni CXNP1	112	--EKD-----TLIL--EGERSKDA---	126
Loxodonta africana CXNP1	112	--GEE-----TVFQ--EEGSSRDA---	126
Homo sapiens CXNQ	111	-------AAR--SKPYAMR-W-KQH----RAL-EETEEDNEED-----PMMYP-EMELESDK---	150
Pan troglodytes CXNQ	111	-------AAR--SKPYAMR-W-KQH----RAL-EETEEDNEED-----PMMYP-EMELESDK---	150
Pongo abelii CXNQ	111	-------AAR--SKPYAMR-W-KQH----RAL-EETEEDNEED-----PMMYP-EMELESDK---	150
Macaca mulatta CXNQ	111	-------AAR--SKPYAMR-W-KQH----RAL-EETEEDNEED-----PMMYP-EMELESDK---	150
Tupaia belangeri CXNQ	111	-------ASR--SKPYAMR-W-KQH----RAL-EETEEDHEED-----PMMYP-EMELESEK---	150
Mus musculus Cxnq	111	-------AAR--SKPYAMR-W-KQH----RAL-EETEEDHEED-----PMMYP-EMELESEK---	150
Rattus norvegicus Cxnq	111	-------AAR--SKPYAMR-W-KQH----RAL-EETEEDHEED-----PMMYP-EMELESEK---	150
Cavia porcellus CXNQ	111	-------AAR--SKPYAMH-W-KQH----RAL-EETEEDHEED-----PMMYP-EMELESEK---	150
S.tridecemlineatus CXNQ	125	-------AAR--SKPYAMR-W-KQH----RAL-EETEEDHEED-----PMMYP-EMELESEK---	164
Oryctolagus cuniculus CXNQ	111	-------AAR--SKPYAMR-W-KQH----RAL-EETEEDHEED-----PMMYP-EMELESEK---	150
Bos taurus CXNQ	111	-------AAR--SKPYAMR-W-KQH----RAL-EETEEDHEED-----PMMYP-EMELESEK---	150
Vicugna pacos CXNQ	111	-------AAR--SKPYAMH-W-KQH----RAL-EETEEDHEED-----PMMYP-EMELESEK---	150
Equus caballus CXNQ	111	-------AAR--SKLYAMR-W-KQH----RAL-EETEEDHEED-----PMMYP-EMELESEK---	150
Canis lupus familiaris CXNQ	111	-------AAR--SKPYAMR-W-KQH----RAL-EETEEDHEED-----PMMYP-EMELESEK---	150
Myotis lucifugus CXNQ	111	-------AAR--SKPYAMR-W-KQH----RAL-EETEEDHEED-----PMMYP-EVELESEK---	150
Pteropus vampyrus CXNQ	111	-------AAR--SKPYAMR-W-KQH----RAL-EETEEDHEED-----PMMYP-EMELESEK---	150
Loxodonta africana CXNQ	111	-------AAR--SRPYAMR-W-KQH----RAL-EETEEDHEED-----PMMYP-EMELESEK---	150
Homo sapiens CXNR	120	AQC-APCALR--AR-RARRC---	135
Pan troglodytes CXNR	120	AQC-APCALR--AR-RARSC---	135
Pongo abelii CXNR	120	AQC-APCALR--AR-RARRC---	135
Papio hamadryas CXNR	120	AQC-APCALR--AR-RARRC---	135
Mus musculus Cxnr	120	APC-SPCALR--AR-RARRC---	135
Rattus norvegicus Cxnr	120	VPC-SPCALR--AR-RARRC---	135
Oryctolagus cuniculus CXNR	128	AAR-GPRGPR--AR-AARRC---	143
Bos taurus CXNR	113	----APGRPG--DR-RARRC---	125
Homo sapiens CXNS	78	GT--GG-GGSGGGKREDKK-LQNAIVNGVLQNTENTSKETEPD----C-L---------------	118
Pan troglodytes CXNS	78	GT--GG-GGSGGGKREDKK-LQNAIVNGVLQNTENTSKETEPD----C-L---------------	118
Pongo abelii CXNS	78	GT--GG-GGSGGGKREDKK-LQNAIVNGVLQNTENTSKETEPD----C-L---------------	118
Nomascus leucogenys CXNS	78	GT--GG-GGSGGGKREDKK-LQNAIVNGVLQNTENTSKETEPD----C-L---------------	118
Macaca mulatta CXNS	78	GT--GG-GGSGGGKREDKK-LQNAIVNGVLQNTENTSKETEPD----C-L---------------	118
Papio hamadryas CXNS	78	GT--GG-GGSGGGKREDKK-LQNAIVNGVLQNTENTGKETEPD----C-L---------------	118
Otolemur garnettii CXNS	78	GT--GG-GGSGGGKREDKK-LQNAIVNGVLQNTENTNKETEPD----C-L---------------	118
Tupaia belangeri CXNS	78	GT--GG-GGSGGGKREDKK-LQNAIVNGVLQNTENTSKETEPD----C-L---------------	118
Mus musculus Cxns	118	GT--GG-GGSGGSKREDKK-LQNAIVNGVLQNTETTSKETEPD----C-L---------------	158
Rattus norvegicus Cxns	78	GT--GG-GGSGGSKREDKK-LQNAIVNGVLQNTETTSKETEPD----C-L---------------	118
Cavia porcellus CXNS	78	GT--GG-GGSGGGKREDKK-LQNAIVNGVLQNTENTSKETEPD----C-L---------------	118
S.tridecemlineatus CXNS	78	GT--GG-GVSGGGKREDKK-LQNAIVNGVLQNTENTSKETEPD----C-L---------------	118

```
Oryctolagus cuniculus CXNS     78 GT--GG-GGSGGGKREDKK-LQNAIVNGVLQNPENPSKETEPD----C-L--------------- 118
Ochotona princeps CXNS         78 GS--GG-GGSGGGKREDKK-LQNAIVNGVLQNTENPSKETEPD----C-L--------------- 118
Bos taurus CXNS                78 GT--GG-GGSGGGKREDKK-LQNAIVNGVLQNTENTSKETEPD----C-L--------------- 118
Equus caballus CXNS            78 GT--GG-GSSSGGKREDKK-LQNAIVNGVLQNTENTSKETEPD----C-L--------------- 118
Canis lupus familiaris CXNS    78 GT--GS-GASGSSKREDKK-LQNAIVNGVLQNTENTSKEVEPD----C-L--------------- 118
Myotis lucifugus CXNS          78 GT--GG-MGSGGGKREDKK-MQNAIVNGVLQNTENTSKETEPD----C-L--------------- 118
Dasypus novemcinctus CXNS      78 GT--GG-GGSGGGKREDKK-LQNAIVNGVLQNTENTSKETEPD----C-L--------------- 118
Loxodonta africana CXNS       206 GT--GA-GGSGGGKREDKK-LQNAIVNGVLQNTESTSKETEPD----C-L--------------- 246
Homo sapiens CXNT            106 ------------I---------------------------------------------------- 106
Pan troglodytes CXNT         106 ------------I---------------------------------------------------- 106
Nomascus leucogenys CXNT     109 ------------I---------------------------------------------------- 109
Mus musculus Cxnt            109 ------------I---------------------------------------------------- 109
Rattus norvegicus Cxnt       109 ------------I---------------------------------------------------- 109
Cavia porcellus CXNT         105 ------------I---------------------------------------------------- 105
Oryctolagus cuniculus CXNT   109 ------------I---------------------------------------------------- 109
Bos taurus CXNT              109 ------------I---------------------------------------------------- 109
Vicugna pacos CXNT           109 ------------I---------------------------------------------------- 109
Equus caballus CXNT          109 ------------I---------------------------------------------------- 109
Canis lupus familiaris CXNT  109 ------------I---------------------------------------------------- 109
Myotis lucifugus CXNT        109 ------------I---------------------------------------------------- 109
Dasypus novemcinctus CXNT    109 ------------I---------------------------------------------------- 109
Loxodonta africana CXNT      105 ------------I---------------------------------------------------- 105
Homo sapiens CXNU            108 RRCPRP-FGERGG---------------------------------------------------- 119
Pan troglodytes CXNU         108 RRCPRP-CGEGGG---------------------------------------------------- 119
Pongo abelii CXNU             93 RRCPRP-CRERGG---------------------------------------------------- 104
Nomascus leucogenys CXNU     105 RRCPRP-CRGRGG---------------------------------------------------- 116
Macaca mulatta CXNU          108 RRCPGS-CRERGG---------------------------------------------------- 119
Mus musculus Cxnu             89 ------------R----------------------------------------------------  89
Cavia porcellus CXNU          77 -------QSERGV----------------------------------------------------  82
Bos taurus CXNU              100 RRC-------------------------------------------------------------- 102
Equus caballus CXNU           99 RRCPPP-YREARN---------------------------------------------------- 110
Myotis lucifugus CXNU        115 RLCPLL-CREGRS---------------------------------------------------- 126
Sorex araneus CXNU            89 --CPRP-RGACGH----------------------------------------------------  98
Dasypus novemcinctus CXNU    125 ---------VGSD---------------------------------------------------- 128
Loxodonta africana CXNU      126 RHHPRP-SREGRG---------------------------------------------------- 137
```

```
Homo sapiens CXNA               116 -----------------------------------------LYLNPGKKRGGLWWTYV----C-- 133
                                            400       410       420       430       440       450
Homo sapiens CXNA               116 -----------------------------------------LYLNPGKKRGGLWWTYV----C-- 133
Pan troglodytes CXNA            116 -----------------------------------------LYLNPGKKRGGLWWTYV----C-- 133
Gorilla gorilla CXNA            116 -----------------------------------------LYLNPGKKRGGLWWTYV----C-- 133
Pongo abelii CXNA               116 -----------------------------------------LYLNPGKKRGGLWWTYV----C-- 133
Nomascus leucogenys CXNA        116 -----------------------------------------LYLNPGKKRGGLWWTYV----C-- 133
Macaca mulatta CXNA             116 -----------------------------------------LYLNPGKKRGGLWWTYV----C-- 133
Callithrix jacchus CXNA         116 -----------------------------------------LYLNPGKKRGGLWWTYV----C-- 133
Mus musculus Cxna               114 -----------------------------------------LYPNPGKKRGGLWWTYV----F-- 131
Rattus norvegicus Cxna          114 -----------------------------------------LYPNPGKKRGGLWWTYV----C-- 131
Cavia porcellus CXNA            166 -----------------------------------------LYLDPGKKRGGLWWTYV----C-- 183
Ochotona princeps CXNA          116 -----------------------------------------LYLNPGKKRGGLWWTYV----C-- 133
Bos taurus CXNA                 116 -----------------------------------------LYLDPGKKRGGLWWTYV----C-- 133
Equus caballus CXNA             116 -----------------------------------------LYLNPGKKQGGLWWTYV----C-- 133
Canis lupus familiaris CXNA     116 -----------------------------------------LYLDPGKKRGGLWWTYV----C-- 133
Felis catus CXNA                116 -----------------------------------------LYLNPGKKRGGLWWTYV----C-- 133
Myotis lucifugus CXNA           148 -----------------------------------------LYPNPGKKRGGLWWTYV----F-- 165
Dasypus novemcinctus CXNA       116 -----------------------------------------LYLNPGKKRGGLWWTYV----G-- 133
Loxodonta africana CXNA         116 -----------------------------------------LYLDPGKKRGGLWWTYV----C-- 133
Homo sapiens CXNB               116 -----------------------------------------LYDNLSKKRGGLWWTYL------- 132
Gorilla gorilla CXNB            116 -----------------------------------------LYDNLSKKRGGLWWTYL------- 132
Nomascus leucogenys CXNB        116 -----------------------------------------LYDNPSKKRGGLWWTYL------- 132
Macaca mulatta CXNB             116 -----------------------------------------LYNNPSKKRGGLWWTYL------- 132
Callithrix jacchus CXNB         116 -----------------------------------------LYDNPSKKRGGLWWTYL------- 132
Otolemur garnettii CXNB         116 -----------------------------------------LYDNPTKKRGGLWWTYL------- 132
Mus musculus Cxnb               116 -----------------------------------------LYSNLSKKRGGLWWTYL------- 132
Rattus norvegicus Cxnb          116 -----------------------------------------LYSNLSKKRGGLWWTYL------- 132
Dipodomys ordii CXNB            116 -----------------------------------------LYNNLSKKRGGLWWTYL------- 132
Cavia porcellus CXNB            116 -----------------------------------------LYSNPSKKRGGLWWTYL------- 132
Oryctolagus cuniculus CXNB       24 -----------------------------------------LYSNLSKKRGGLWWTYL-------  40
Ochotona princeps CXNB          116 -----------------------------------------LYSNLGKKRGGLWWTYL------- 132
Bos taurus CXNB                 116 -----------------------------------------LYDNPDKKRGGLWWTYL------- 132
Equus caballus CXNB             116 -----------------------------------------LYDNPSKKRGGLWWTYL------- 132
Canis lupus familiaris CXNB     166 -----------------------------------------LYDNPSKKRGGLWWTYL------- 182
Myotis lucifugus CXNB           116 -----------------------------------------LYDNPSKKRGGLWWTYL------- 132
Erinaceus europaeus CXNB        116 -----------------------------------------LYNDPEKKRGGLWWTYL------- 132
Loxodonta africana CXNB         116 -----------------------------------------LYDNAGKKRGGLWWTYL------- 132
Homo sapiens CXNC               116 -----------------------------------------LYDNAGKKHGGLWWTYL------- 132
Gorilla gorilla CXNC            116 -----------------------------------------LYDNAGKKHGGLWWTYL------- 132
Pongo abelii CXNC               116 -----------------------------------------LYDNAGKKHGGLWWTYL------- 132
Nomascus leucogenys CXNC        116 -----------------------------------------LYDNAGKKHGGLWWTYL------- 132
Callithrix jacchus CXNC         116 -----------------------------------------LYDNAGKKHGGLWWTYL------- 132
Microcebus murinus CXNC         146 -----------------------------------------LYDDAGKKHGGLWWTYL------- 162
Otolemur garnettii CXNC         116 -----------------------------------------LYDNAGKKHGGLWWTYL------- 132
Mus musculus Cxnc               116 -----------------------------------------LYSHPGKKHGGLWWTYL------- 132
Rattus norvegicus Cxnc          116 -----------------------------------------LYSHPGKKHGGLWWTYL------- 132
Cavia porcellus CXNC            116 -----------------------------------------LYSNTGKKHGGLWWTYL------- 132
Oryctolagus cuniculus CXNC      226 -----------------------------------------LYSDTGKKHGGLWWTYL------- 242
Bos taurus CXNC                 116 -----------------------------------------LYDDTGKKHGGLWWTYL------- 132
Equus caballus CXNC             116 -----------------------------------------LYDNTGKKHGGLWWTYL------- 132
Canis lupus familiaris CXNC     116 -----------------------------------------LYADAGKKHGGLWWTYL------- 132
Felis catus CXNC                116 -----------------------------------------LYANAGKKHGGLWWTYL------- 132
Myotis lucifugus CXNC           116 -----------------------------------------LYDNAGKKHGGLWWTYL------- 132
Dasypus novemcinctus CXNC       116 -----------------------------------------LYDDTGKKHGGLWWTYL------- 132
Loxodonta africana CXNC         116 -----------------------------------------LYDDATKKHGGLWWTYL------- 132
Homo sapiens CXND               109 -----------------------------------------LYVSPGTMDGGLWYAYL------- 125
Pan troglodytes CXND            109 -----------------------------------------LYVSPGTMDGGLWYAYL------- 125
Macaca mulatta CXND             109 -----------------------------------------LYVSLGTMDGGLWYTYL------- 125
Callithrix jacchus CXND         109 -----------------------------------------LYVSPGTMHRGLWYTYL------- 125
Dipodomys ordii CXND            109 -----------------------------------------LYVSPGTMDGGLWYTYL------- 125
Oryctolagus cuniculus CXND      109 -----------------------------------------LYVSPGMMDGGLWYTYI------- 125
Tursiops truncatus CXND         109 -----------------------------------------LYVSPGTMDGGLWYTYP------- 125
Bos taurus CXND                  27 -----------------------------------------LYVSPGTMGGGLWYTYL-------  43
Equus caballus CXND             128 -----------------------------------------LYVSPGTMDGGLWYTYL------- 144
Canis lupus familiaris CXND     109 -----------------------------------------LYVSPGMMDGGLWYTYL------- 125
Myotis lucifugus CXND           109 -----------------------------------------LYVSLGTMDGGLWYTYL------- 125
Erinaceus europaeus CXND        109 -----------------------------------------LYVSTGRMDGGLWYTYL------- 125
Dasypus novemcinctus CXND       109 -----------------------------------------LYVSPGTMDGGLWYTYL------- 125
Choloepus hoffmanni CXND        109 -----------------------------------------LYVSPGTMDGGLWYTYL------- 125
Loxodonta africana CXND         109 -----------------------------------------LYVSPGAMDGGLWYTYL------- 125
Homo sapiens CXNE               121 -----------------------------------------IKTQKVRIEGSLWWTYT------- 137
Pan troglodytes CXNE            121 -----------------------------------------IKTQKVRIEGSLWWTYT------- 137
Pongo abelii CXNE               121 -----------------------------------------IKTQKVRIEGSLWWTYT------- 137
Macaca mulatta CXNE             121 -----------------------------------------IKTQKVRIEGSLWWTYT------- 137
Papio hamadryas CXNE            121 -----------------------------------------IKTQKVRIEGSLWWTYT------- 137
Callithrix jacchus CXNE         121 -----------------------------------------IKSQKVRIEGSLWWTYT------- 137
Otolemur garnettii CXNE         121 -----------------------------------------IKSQKVRIEGSLWWTYT------- 137
Mus musculus Cxne               134 -----------------------------------------IKTQKVRIEGSLWWTYT------- 150
```

```
Rattus norvegicus Cxne          140 -----------------------------------------IKTQKVRIEGSLWWTYT------- 156
Dipodomys ordii CXNE            140 -----------------------------------------IKTQKVRIEGSLWWTYT------- 156
Cavia porcellus CXNE            168 -----------------------------------------IKNEKIRIEGSLWWTYT------- 184
Oryctolagus cuniculus CXNE      140 -----------------------------------------IKSQKVRIEGSLWWTYT------- 156
Equus caballus CXNE             121 -----------------------------------------IKSQKVRIEGSLWWTYT------- 137
Canis lupus familiaris CXNE     121 -----------------------------------------IKSQKVRIEGSLWWTYT------- 137
Felis catus CXNE                121 -----------------------------------------IKSQKVRIEGSLWWTYT------- 137
Myotis lucifugus CXNE           121 -----------------------------------------IKNQKVRIEGSLWWTYT------- 137
Sorex araneus CXNE              121 -----------------------------------------IKNQKVRIQGSLWWTYT------- 137
Dasypus novemcinctus CXNE       121 -----------------------------------------VKSQKVRIEGSLWWTYT------- 137
Loxodonta africana CXNE         121 -----------------------------------------IKSQKVRIEGSLWWTYT------- 137
Homo sapiens CXNF               121 -----------------------------------------IKKQKVRIEGSLWWTYT------- 137
Pan troglodytes CXNF            121 -----------------------------------------IKKQKVRIEGSLWWTYT------- 137
Gorilla gorilla CXNF            121 -----------------------------------------IKKQKVRIEGSLWWTYT------- 137
Nomascus leucogenys CXNF        121 -----------------------------------------IKAQKVRIEGSLWWTYT------- 137
Callithrix jacchus CXNF         121 -----------------------------------------IKKQKVRIEGSLWWTYT------- 137
Mus musculus Cxnf               121 -----------------------------------------IKRQKVRIEGSLWWTYT------- 137
Rattus norvegicus Cxnf          121 -----------------------------------------IKRQKVRIEGSLWWTYT------- 137
S.tridecemlineatus CXNF         121 -----------------------------------------IKKQKVRIEGSLWWTYT------- 137
Oryctolagus cuniculus CXNF      121 -----------------------------------------IKKQKVRIEGSLWWTYT------- 137
Ochotona princeps CXNF          121 -----------------------------------------IKKQKVRIEGSLWWTYT------- 137
Bos taurus CXNF                 121 -----------------------------------------IKRQKVRIEGSLWWTYT------- 137
Equus caballus CXNF             121 -----------------------------------------IKKQKVRIEGSLWWTYT------- 137
Canis lupus familiaris CXNF     121 -----------------------------------------IKKQKVRIEGSLWWTYT------- 137
Myotis lucifugus CXNF           121 -----------------------------------------IKKQKVRIEGALWWTYT------- 137
Dasypus novemcinctus CXNF       121 -----------------------------------------IKTQKVRIEGSLWWTYT------- 137
Choloepus hoffmanni CXNF        121 -----------------------------------------IKKQKVRIVGSLWWTYT------- 137
Loxodonta africana CXNF         121 -----------------------------------------IKKQKVRIEGSLWWTYT------- 137
Homo sapiens CXNG               120 -----------------------------------------VKRHKVHISGTLWWTYV------- 136
Pan troglodytes CXNG            120 -----------------------------------------VKRHKVHISGTLWWTYV------- 136
Gorilla gorilla CXNG            120 -----------------------------------------VKRHKVHISGTLWWTYV------- 136
Pongo abelii CXNG               120 -----------------------------------------VKRHKVHISGTLWWTYV------- 136
Nomascus leucogenys CXNG        120 -----------------------------------------VKRHKVHISGTLWWTYV------- 136
Macaca mulatta CXNG             120 -----------------------------------------VKRHKVHISGTLWWTYV------- 136
Papio hamadryas CXNG            120 -----------------------------------------VKRHKVHISGTLWWTYV------- 136
Callithrix jacchus CXNG         120 -----------------------------------------VKRHKVHISGTLWWTYV------- 136
Microcebus murinus CXNG         120 -----------------------------------------VKRHKVHISGTLWWTYV------- 136
Mus musculus Cxng               120 -----------------------------------------VKRHKVHISGTLWWTYV------- 136
Rattus norvegicus Cxng          120 -----------------------------------------VKRHKVHISGTLWWTYV------- 136
Oryctolagus cuniculus CXNG      120 -----------------------------------------VKRHKVHISGTLWWTYV------- 136
Bos taurus CXNG                 120 -----------------------------------------VKRHKVHISGTLWWTYV------- 136
Equus caballus CXNG             120 -----------------------------------------VKRHKVHISGTLWWTYV------- 136
Canis lupus familiaris CXNG     120 -----------------------------------------VKRHKVHISGTLWWTYV------- 136
Felis catus CXNG                120 -----------------------------------------VKRHKVHISGTLWWTYV------- 136
Myotis lucifugus CXNG           120 -----------------------------------------VKRHKVHISGTLWWTYV------- 136
Pteropus vampyrus CXNG          120 -----------------------------------------VKRHKFHISGTLWWTYV------- 136
Dasypus novemcinctus CXNG       120 -----------------------------------------VKRHKVHISGTLWWTYV------- 136
Loxodonta africana CXNG         120 -----------------------------------------VKRHKVHISGTLWWTYV------- 136
Procavia capensis CXNG          120 -----------------------------------------VKRHKVHISGTLWWTYV------- 136
Homo sapiens CXNH1              127 LAA------------------VERQMAK--IS-V---AE-DGRL-RIR--GALMGTYVASVLC-- 161
Pan troglodytes CXNH1           127 LAA------------------VERQMAK--IS-V---AE-DGRL-RIR--GALMGTYVASVLC-- 161
Gorilla gorilla CXNH1           127 LAA------------------VERQMAK--IS-V---AE-DGRL-RIR--GALMGTYVASVLC-- 161
Pongo abelii CXNH1              127 LAA------------------VERQMAK--IS-V---AE-DGRL-RIR--GALMGTYVASVLC-- 161
Nomascus leucogenys CXNH1       127 LAA------------------VERQMAK--IS-V---AE-DGRL-RIR--GALMGTYVASVLC-- 161
Macaca mulatta CXNH1            127 LAA------------------VERQMAK--IS-V---AE-DGRL-RIR--GALMGTYVASVLC-- 161
Otolemur garnettii CXNH1        127 PGT------------------IKHQMSN--KS---------GRR-QIK--GALKQTYVATVLW-- 157
Mus musculus Cxnh1              127 LAA------------------IEHQMAK--IS-V---AE-DGRL-RIR--GALMGTYVVSVLC-- 161
Rattus norvegicus Cxnh1         127 LAA------------------IEHQMAK--IS-V---AE-DGRL-RIR--GALMGTYVISVLC-- 161
Cavia porcellus CXNH1           127 LAA------------------VERQMAK--IS-V---AE-DGRL-RIR--GALMGTYVASVLC-- 161
Oryctolagus cuniculus CXNH1     127 LAA------------------VERQMAK--IS-V---AE-DGRL-RIR--GALMGTYVASVLC-- 161
Ochotona princeps CXNH1         141 LAA------------------VERQMAK--IS-V---AE-DGRL-RIR--GALMGTYVASVLC-- 175
Equus caballus CXNH1            127 LAA------------------VERQMTK--IS-V---AE-DGRV-RIR--GALMGTYVASVLC-- 161
Canis lupus familiaris CXNH1    127 LAA------------------VERQMAK--IS-V---AE-DGRL-RIR--GALMGTYVASVLC-- 161
Felis catus CXNH1               127 LAA------------------IERQMAK--IS-V---AE-DGRL-RIR--GALMGTYVASVLC-- 161
Myotis lucifugus CXNH1          159 LAA------------------IERQMAK--IS-V---AE-DGRM-RIR--GALMGTYVLSVIC-- 193
Dasypus novemcinctus CXNH1      127 LVA------------------VEHQMAK--IS-V---AE-DGRL-RIR--GALMGTYVASVLC-- 161
Loxodonta africana CXNH1        127 LAT------------------VERQMAK--IS-V---AE-DGRL-RIR--GALMGTYVTSVLC-- 161
Homo sapiens CXNI               124 EYP--V---------------AEKAELS-C-W------E-EGNG-RIALQGTLLNTYV----C-- 155
Pan troglodytes CXNI            124 EYP--V---------------AEKAELS-C-W------E-EGNG-RIALQGTLLNTYV----C-- 155
Pongo abelii CXNI               124 EYP--V---------------AEKAELS-C-W------E-EGNG-RIALQGTLLNTYV----C-- 155
Nomascus leucogenys CXNI        124 EYP--V---------------AEKAELS-C-W------E-EGNG-RIALQGTLLNTYV----C-- 155
Macaca mulatta CXNI             124 EYP--V---------------AEKAELS-C-W------E-EGNG-RIVLQGSLLNTYV----C-- 155
Papio hamadryas CXNI            124 EYP--V---------------AEKAELS-C-W------E-EGNG-RIVLQGSLLNTYV----C-- 155
Callithrix jacchus CXNI         124 EYP--V---------------AEKAEPS-C-W------E-EGNG-RIVLQGTLLHTYV----C-- 155
Otolemur garnettii CXNI         124 EYP--V---------------G-KAELT-C-W------E-EANG-KIVLQGTLLNTYV----C-- 154
Mus musculus Cxni               124 EYP--V---------------AEKAELS-C-W------K-EVDG-KIVLQGTLLNTYV----C-- 155
Rattus norvegicus Cxni          158 EY---L---------------AEKAELS-C-W------K-EVNG-KIVLQGTLLNTYV----C-- 188
Cavia porcellus CXNI            125 EFQ--V---------------AEKPELS-C-W------E-EMNG-KVVLQGNLLNTYV----C-- 156
Oryctolagus cuniculus CXNI      124 EYP--A---------------AEKAELS-R-W------E-EVNG-RILLRGTLLRTYV----C-- 155
```

```
Bos taurus CXNI                  125 EYP--V---------------AEKTELS-C-W------E-EVNG-RIALQGSLLNTYV----C-- 156
Equus caballus CXNI              124 EYP--V---------------AEKTALS-C-W------E-EGNE-KIVLQGTLLNTYV----C-- 155
Canis lupus familiaris CXNI      124 EYP--V---------------AEKAELS-C-W------E-EVNG-RIVLQGTLLNTYV----C-- 155
Felis catus CXNI                 124 EYP--V---------------AEKAELS-C-W------E-EANG-RIVLQGTLLNTYV----C-- 155
Pteropus vampyrus CXNI           124 AYP--V---------------GEKAELS-C-W------E-EANG-RIVLRGTLLNTYV----C-- 155
Erinaceus europaeus CXNI         123 QYP-AVTT-------------AEKAELS-C-W------E-EVNG-KIVLQGTLLNTYV----C-- 157
Dasypus novemcinctus CXNI        124 EYP--V---------------AEKTELS-C-W------E-EANG-KIILRGTLLNTYV----C-- 155
Loxodonta africana CXNI          124 EYP--V---------------AEKTELS-C-W------E-EVNG-KVVLQGALLNTYV----C-- 155
Homo sapiens CXNJ1               124 EPP------------------QDN-PSS---R------D-D-RG-RVRMAGALLRTYV------- 150
Pongo abelii CXNJ1               124 EPP------------------QDN-PSS---R------D-D-RG-RVRMAGALLRTYV------- 150
Macaca mulatta CXNJ1             124 EPP------------------QDR-PSS---R------E-D-RG-RVRMAGALLRTYV------- 150
Mus musculus Cxnj1               126 EPM-RT-G-------------SPRDPPL---R------D-D-RG-KVRIAGALLRTYV------- 156
Rattus norvegicus Cxnj1          126 EPM-RT-G-------------SPRDPPL---R------D-D-RG-KVRIAGALLRTYV------- 156
Bos taurus CXNJ1                 123 ---------------------QDP-APV---R------D-D-RG-KVRIAGALLRTYV------- 146
Equus caballus CXNJ1             127 CGL-SSDS-------------QDT-PPV---R------D-D-RG-KVRIAGALLRTYV------- 157
Myotis lucifugus CXNJ1           122 ---------------------QKNRPLV---R------D-D-QG-KVLIAGALLRTYI------- 146
Myotis lucifugus CXNJ2           127 CGP-RNHS-------------QKDKFIL---Q------D-N-RG-KVRIAGALLRTYI------- 158
Pteropus vampyrus CXNJ1          291 ---------------------QRDRPPV---R------D-D-RG-KVRIAGALLRTYV------- 315
Pteropus vampyrus CXNJ2          213 ---------------------QKDRPPV---R------D-D-RG-KVRISGALLRTYV------- 237
Sorex araneus CXNJ1              122 ------------------------------------------RR-STSLAGALLRTYV------- 136
Homo sapiens CXNK1               129 Q--------------------IEIKKFK---Y-G---IE-E-HG-KVKMQGGLLRTYI------- 156
Homo sapiens CXNK2               129 Q--------------------IEIKKFK---Y-G---IE-E-HG-KVKMRGGLLRTYI------- 156
Pan troglodytes CXNK1            129 Q--------------------IEITKFK---Y-G---IE-E-HG-KVKMQGGLLRTYI------- 156
Pan troglodytes CXNK2            129 Q--------------------IEIKKFK---Y-G---IE-E-HG-KVKMRGGLLRTYI------- 156
Pongo abelii CXNK1               129 Q--------------------IEIKKFK---Y-G---IE-E-HG-KVKMRGGLLRTYI------- 156
Nomascus leucogenys CXNK1        129 Q--------------------IEIKKFK---Y-G---IE-E-HG-KVKMRGGLLRTYI------- 156
Callithrix jacchus CXNK1         129 Q--------------------IEIKKFK---Y-G---IE-E-HG-KVKMRGGLLRTYI------- 156
Mus musculus Cxnk1               129 Q--------------------IEIKKFK---Y-G---IE-E-HG-KVKMRGGLLRTYI------- 156
Mus musculus Cxnk2               129 T--------------------IAGEQFK---C-G---SE-E-QS-KVKMRGRLLLTYM------- 156
Rattus norvegicus Cxnk1          129 Q--------------------IEIKKFK---Y-G---IE-E-HG-KVKMRGGLLRTYI------- 156
Rattus norvegicus Cxnk2          130 K--------------------HTGKHIK---C-G---SK-E-HG-NRKMRGRLLLTYM------- 157
Cavia porcellus CXNK1            129 Q--------------------IEIKKFK---Y-G---IE-E-HG-KVKMRGGLLRTYI------- 156
Oryctolagus cuniculus CXNK1      129 Q--------------------IEIKKFK---Y-G---IE-E-HG-KVKMRGGLLRTYI------- 156
Bos taurus CXNK1                 130 Q--------------------IEIKKFK---Y-G---IE-E-HG-KVKMRGGLLRTYI------- 157
Vicugna pacos CXNK1              129 Q--------------------IEIKKFK---Y-G---IE-E-HG-KVKMRGGLLRTYI------- 156
Equus caballus CXNK1             129 Q--------------------IEIKKFK---Y-G---IE-E-HG-KVKMRGGLLRTYI------- 156
Equus caballus CXNK2             106 E--------------------IEIKKMK---Y-G---IE-E-HG-KVKVRGGLLRTYT------- 133
Canis lupus familiaris CXNK1     129 Q--------------------IEIKKFK---Y-G---IE-E-HG-KVKMRGGLLRTYI------- 156
Canis lupus familiaris CXNK2     129 E---E-IE-------------IEIKNFR---F-D---IE-K-YC-KMKMRRGLLRIYI------- 159
Felis catus CXNK1                128 ---------------------IEIKKFK---F-S---AE-E-HY-KVKMRRDLLRIYT------- 154
Myotis lucifugus CXNK1           129 Q--------------------IEIKKFK---Y-G---IE-E-HG-KVKMRGGLLRTYI------- 156
Dasypus novemcinctus CXNK1       129 E--------------------VEIRKLK---Y-G---ME-E-HG-KMKMRGDLLWTYI------- 156
Dasypus novemcinctus CXNK2       129 Q--------------------IEIKKFK---Y-G---IE-E-HG-KVKMRGGLLRTYI------- 156
Loxodonta africana CXNK1         129 Q--------------------IEIKKFK---Y-G---IE-E-HG-KVKMRGGLLRTYI------- 156
Loxodonta africana CXNK2         129 E--------------------SEIRKLR---F-G---VE-E-HG-KVKTRGGLLQTYI------- 156
Homo sapiens CXNL                124 --P-DQ---------------GSVKKSS-----G----S-KGTK-KFRLEGTLLRTYI----C-- 153
Pan troglodytes CXNL             124 --P-DQ---------------GSVKNSS-----G----S-KGTK-KFRLEGTLLRTYI----C-- 153
Pongo abelii CXNL                124 --P-DQ---------------GSVKKSS-----G----S-KGSK-KFRLEGTLLRTYI----C-- 153
Nomascus leucogenys CXNL         124 --P-DQ---------------GSVKKSS-----G----S-KGTK-KFRLEGTLLRTYI----C-- 153
Macaca mulatta CXNL              128 LSP-DQ---------------GNVKKSS-----G----S-KGTK-KFRLEGTLLRTYI----C-- 159
Callithrix jacchus CXNL          127 LGA-DQ---------------GGGKKGG-----G------KGTK-KFRLEGTLLRTYI----C-- 157
Otolemur garnettii CXNL          172 LTP-DQ---------------GSIKKSS-----S---SS-KGTK-KFRLEGTLLRTYI----C-- 204
Mus musculus Cxnl                128 IAP-DQ---------------ASIRKSS-----S---SS-KGTK-KFRLEGTLLRTYV----C-- 160
Rattus norvegicus Cxnl           128 IAP-DQ---------------ASIRKSS-----S---SS-KGTK-KFRLEGTLLRTYV----C-- 160
Cavia porcellus CXNL             128 LAP-DQ---------------ASIRKSS-----S---SS-KGTK-KFRLEGTLLRTYI----C-- 160
Oryctolagus cuniculus CXNL       128 ISP-DQ---------------GSIRKSS-----S---SS-KGTK-KFRLEGTLLRTYI----C-- 160
Ochotona princeps CXNL           125 MSP-DQ---------------ASIRKSS-----S---SS-KGTK-KFRLEGTLLRTYV----C-- 157
Bos taurus CXNL                  128 LAA-DQ---------------GSVKKSS-----S---SS-KGTK-KFRLEGTLLRTYI----C-- 160
Equus caballus CXNL              127 PAA-DQ---------------CSIKKGG-----G---GS-KGTK-KFRLEGTLLRTYI----C-- 159
Canis lupus familiaris CXNL      128 LAT-DQ---------------GSIKKGG-----N---SS-KGAK-KFRLEGTLLRTYI----C-- 160
Felis catus CXNL                 128 LAA-DQ---------------GSVKKGS-----N---SS-KGTK-KFRLEGTLLRTYI----C-- 160
Pteropus vampyrus CXNL           128 LPA-DQ---------------GSIKKSS-----S---SS-KGTK-KFRLEGTLLRTYI----C-- 160
Sorex araneus CXNL               108 ---------------------------G-----Q---SS-RGAP-RLRLEGTLLRTYV----C-- 129
Dasypus novemcinctus CXNL        128 LPP-DQ---------------GSLKKSS-----S---SS-KGTK-KFRLEGTLLRTYI----C-- 160
Loxodonta africana CXNL          128 VAP-DQ---------------GSIKKSS---------SS-RSTK-KFRLEGTLLRTYI----C-- 159
Homo sapiens CXNM                126 --P-RD---------------RRRLEQELC-Q-----LE---KR-KLN-K-APLR--G-TLLC-- 155
Pan troglodytes CXNM             126 --P-RD---------------RRRLEQELC-Q-----LE---KR-KLN-K-APLR--G-TLLC-- 155
Pongo abelii CXNM                126 --P-RD---------------RRRLEQELC-Q-----LE---KR-KLN-K-APLR--G-TLLC-- 155
Nomascus leucogenys CXNM         126 --P-RD---------------RRRLEQELC-Q-----LE---KR-KLN-K-APLR--G-TLLC-- 155
Macaca mulatta CXNM              126 --P-RD---------------RRRLEQELC-Q-----LE---KR-KLN-K-APLR--G-TLLC-- 155
Callithrix jacchus CXNM          125 --P-RD---------------RRRLEQELC-Q-----LE---KR-KLN-K-APLR--G-TLLC-- 154
Tarsius syrichta CXNM            126 --P-GD---------------RRRLEQELC-Q-----LE---KR-KLN-K-APLR--G-TLLC-- 155
Microcebus murinus CXNM          126 --P-GD---------------RRRLEQELC-Q-----LE---KR-KLN-K-APLR--G-TLLC-- 155
Dipodomys ordii CXNM             126 --P-ED---------------QKRLEQELC-Q-----LE-G-KR-KPN-K-APLR--G-SLLC-- 156
Oryctolagus cuniculus CXNM       124 --S-GD---------------QRNLEPELW-Q-----LG---QR-KRS-K-APLP--E-ALLC-- 153
Equus caballus CXNM              126 --P-GD---------------RRRLEQELY-Q-----LE---QR-KLN-K-APLR--G-TLLC-- 155
Canis lupus familiaris CXNM      126 --P-GD---------------RRRLEQELC-Q-----LE---QR-KLN-K-APLR--G-TLLC-- 155
Pteropus vampyrus CXNM           126 --S-GD---------------RRKLEKELY-Q-----LE----R-TLN-K-APLR--G-TLLC-- 154
Loxodonta africana CXNM          126 --T-GH---------------RRRLEQELC-Q-----LE---QR-KLN-K-APLR--G-MLLC-- 155
```

```
Homo sapiens CXNN              126 ----EE---------------QQRIDREL--R-R---LE-E-QK-RIH-K-VPLK--G----CLL 154
Pan troglodytes CXNN           126 ----EE---------------QQRIDREL--R-R---LE-E-QK-RIH-K-VPLK--G----CLL 154
Pongo abelii CXNN              126 ----DE---------------QQRIDREL--R-R---LE-E-QK-RIH-K-VPLK--G----CLL 154
Nomascus leucogenys CXNN       126 ----EE---------------QQRIDREL--R-R---LE-E-QK-RIH-K-VPLK--G----CLL 154
Macaca mulatta CXNN            126 ----EE---------------QQRIDREL--R-R---LE-E-QK-RIH-K-VPLK--G----CLL 154
Callithrix jacchus CXNN        126 ----EE---------------QQRIDREL--R-R---LE-E-QK-RIH-K-VPLK--G----CLL 154
Microcebus murinus CXNN        126 ----EE---------------QQRIDREL--R-R---LD-E-QK-RIH-K-VPLK--G----CLL 154
Mus musculus Cxnn              126 ----EE---------------QQRVDKEL--R-R---LE-E-QK-RIH-K-VPLK--G----CLL 154
Rattus norvegicus Cxnn         126 ----EE---------------QQRVEREL--R-R---LE-E-QK-RIQ-K-VPLK--G----CLL 154
Oryctolagus cuniculus CXNN     156 ----ED---------------QQRIDREL--R-R---LE-E-QK-RIH-K-VPLK--G----CLL 184
Bos taurus CXNN                126 ----ED---------------QQRMDREL--R-K---LE-E-QK-RIQ-K-VPLK--G----CLL 154
Equus caballus CXNN            126 ----DE---------------QQRIDREL--R-R---LE-E-QK-RIH-K-VPLK--G----CLL 154
Canis lupus familiaris CXNN    126 ----EE---------------QQRIDREL--K-R---LE-E-EK-RIH-K-VPLK--G----CLL 154
Sorex araneus CXNN             126 ----EE---------------QQRIEREL--R-R---LE-E-HR-RVQ-K-VPLR--G----CLL 154
Loxodonta africana CXNN        126 ----EE---------------QQRIHREL--R-M---LE-E-QK-RIY-K-VPLR--G----CLL 154
Homo sapiens CXNO              163 EGAGEEAEEAGAEEACTKAVGADGKAAG---TPGPTG-QHDGRR-RIQR-EGLMRVYV------- 214
Papio hamadryas CXNO           163 EGAGEEAEEAGAEEACTKGAGADGKATG---TPGTAG-QHDGRR-RIQR-EGLMRVYV------- 214
Mus musculus Cxno              173 EGPGEDTEEERAEDVAAKGGGGDGKTVV---TPGPAG-QHDGRR-RIQR-EGLMRVYV------- 224
Cavia porcellus CXNO           163 EE-EEEPVAPGAED---KTGAGD--AVG---AVGPGV-QHDGRR-RIQR-EGLMRAYV------- 208
Homo sapiens CXNP1             126 ----------------------------------------PGAG-SLR----LLWAYV------- 138
Pan troglodytes CXNP1          126 ----------------------------------------PGAG-SLR----LLWAYV------- 138
Pongo abelii CXNP1             126 ----------------------------------------PGTG-SLR----LLWAYV------- 138
Callithrix jacchus CXNP1       127 ----------------------------------------SGDG-NLR----LLWAYV------- 139
Otolemur garnettii CXNP1       127 ----------------------------------------PGAQ-SPL----LLWAYV------- 139
Tupaia belangeri CXNP1         144 ----------------------------------------SRAG-SSR----LFWAYV------- 156
Mus musculus Cxnp1             127 ----------------------------------------SGAK-SLK----LLWAYV------- 139
Rattus norvegicus Cxnp1        126 ----------------------------------------SGAM-SLK----LLWAYV------- 138
Cavia porcellus CXNP1          127 ----------------------------------------SGAG-SHK----IFWAYV------- 139
Oryctolagus cuniculus CXNP1    127 ----------------------------------------LGAG-SLR----LLWAYV------- 139
Oryctolagus cuniculus CXNP2    127 ----------------------------------------SGTG-NLR----LLWAYV------- 139
Ochotona princeps CXNP1        127 ----------------------------------------SGTG-KLR----LLWAYV------- 139
Bos taurus CXNP1               128 ----------------------------------------SRAG-SSR----LLWAYV------- 140
Equus caballus CXNP1           127 ----------------------------------------SGSG-SSR----LFWAYV------- 139
Canis lupus familiaris CXNP1   125 ----------------------------------------SGAG-SPR----LLWAYV------- 137
Felis catus CXNP1              148 ----------------------------------------SGPG-SPR----LLWAYV------- 160
Myotis lucifugus CXNP1         127 ----------------------------------------SGAE-SPK----LLWAYV------- 139
Dasypus novemcinctus CXNP1     126 ----------------------------------------AGSR-GRR----LLWAYV------- 138
Dasypus novemcinctus CXNP2     126 ----------------------------------------AGSR-GRR----LLWAYV------- 138
Dasypus novemcinctus CXNP3     126 ----------------------------------------AGSR-GRR----LLWAYV------- 138
Dasypus novemcinctus CXNP4     126 ----------------------------------------AGSR-GRR----LLWAYV------- 138
Choloepus hoffmanni CXNP1      127 ----------------------------------------V--R-TCK----QLWTYV------- 137
Loxodonta africana CXNP1       127 ----------------------------------------SGAG-SPR----LLWAYV------- 139
Homo sapiens CXNQ              151 ----------------------ENKEQS---QPKP---KHDGRR-RIRE-DGLMKIYV------- 178
Pan troglodytes CXNQ           151 ----------------------ENKEQS---QPKP---KHDGRR-RIRE-DGLMKIYV------- 178
Pongo abelii CXNQ              151 ----------------------ENKEQS---QPKP---KHDGRR-RIRE-DGLMKIYV------- 178
Macaca mulatta CXNQ            151 ----------------------ENKEQS---QPKP---KHDGRR-RIRE-DGLMKIYV------- 178
Tupaia belangeri CXNQ          151 ----------------------ENKEQS---QPKP---KHDGRR-RIRE-DGLMKIYV------- 178
Mus musculus Cxnq              151 ----------------------ENKEQS---QPKP---KHDGRR-RIRE-DGLMKIYV------- 178
Rattus norvegicus Cxnq         151 ----------------------ENKEQS---QPKP---KHDGRR-RIRE-DGLMKIYV------- 178
Cavia porcellus CXNQ           151 ----------------------ENKEQS---PLKP---KHDGRR-RIRE-DGLMKIYV------- 178
S.tridecemlineatus CXNQ        165 ----------------------ENKEQS---QPKP---KHDGRR-RIRE-DGLMKIYV------- 192
Oryctolagus cuniculus CXNQ     151 ----------------------ENKEQT---QPKP---KHDGRR-RIRE-DGLMKIYV------- 178
Bos taurus CXNQ                151 ----------------------ENKDQN---QSKP---KHDGRR-RIRE-DGLMKIYV------- 178
Vicugna pacos CXNQ             151 ----------------------ENKEQN---QSKP---KHDGRR-RIRE-DGLMKIYV------- 178
Equus caballus CXNQ            151 ----------------------ENKEQN---QPKP---KHDGRR-RIRE-DGLMKIYV------- 178
Canis lupus familiaris CXNQ    151 ----------------------ENKEQN---QPKP---KHDGRR-RIRE-DGLMKIYV------- 178
Myotis lucifugus CXNQ          151 ----------------------ENKEQN---QPKP---KHDGRR-RIRE-DGLMKIYV------- 178
Pteropus vampyrus CXNQ         151 ----------------------ENKEQN---QPKA---KHDGRQ-RIRE-DGLMKIYV------- 178
Loxodonta africana CXNQ        151 ----------------------ENKEHS---QPKP---KHDGRR-QIRE-DGLMKIYV------- 178
Homo sapiens CXNR              136 --------------------------------------------------------YL------- 137
Pan troglodytes CXNR           136 --------------------------------------------------------YL------- 137
Pongo abelii CXNR              136 --------------------------------------------------------YL------- 137
Papio hamadryas CXNR           136 --------------------------------------------------------YL------- 137
Mus musculus Cxnr              136 --------------------------------------------------------YL------- 137
Rattus norvegicus Cxnr         136 --------------------------------------------------------YL------- 137
Oryctolagus cuniculus CXNR     144 --------------------------------------------------------YL------- 145
Bos taurus CXNR                126 --------------------------------------------------------YL------- 127
Homo sapiens CXNS              119 ----------------------EVKEL----TPHPSGLRTASKS-KLRRQEGISRFYI------- 149
Pan troglodytes CXNS           119 ----------------------EVKEL----TPHPSGLRTASKS-KLRRQEGISRFYI------- 149
Pongo abelii CXNS              119 ----------------------EVKEL----TPHPSGLRTASKS-KLRRQEGISRFYI------- 149
Nomascus leucogenys CXNS       119 ----------------------EVKEL----TPHPSGLRTASKS-KLRRQEGISRFYI------- 149
Macaca mulatta CXNS            119 ----------------------EVKEL----TPHPSGLRTASKS-KLRRQEGISRFYI------- 149
Papio hamadryas CXNS           119 ----------------------EVKEL----TPHPSGLRTASKS-KLRRQEGISRFYI------- 149
Otolemur garnettii CXNS        119 ----------------------EVKEL----TPHPSGLRTASKS-KLRRQEGISRFYI------- 149
Tupaia belangeri CXNS          119 ----------------------EVKEL----TPHSSGLRTAARS-KLRRQEGISRFYI------- 149
Mus musculus Cxns              159 ----------------------EVKEL----TPHPSGLRTAARS-KLRRQEGISRFYI------- 189
Rattus norvegicus Cxns         119 ----------------------EVKEL----APHPSGLRTAARS-KLRRQEGISRFYI------- 149
Cavia porcellus CXNS           119 ----------------------EVKEL----TPHPSGLRTAARS-KLRRQEGISRFYI------- 149
S.tridecemlineatus CXNS        119 ----------------------EVKEL----TPHPSGLRTAARS-KLRRQEGISRFYI------- 149
```

```
Oryctolagus cuniculus CXNS   119 ----------------------EVKEL----TPHPSGMRTAAKS-KLRRQEGISRFYI------- 149
Ochotona princeps CXNS       119 ----------------------EVKEL----TPHPSGMRTAARS-KLRRQEGISRFYI------- 149
Bos taurus CXNS              119 ----------------------EVKEL----TPHPSGLRTASRS-KLRRQEGISRFYI------- 149
Equus caballus CXNS          119 ----------------------EVKEL----TPHPSGLRTAARS-KLRRQEGISRFYI------- 149
Canis lupus familiaris CXNS  119 ----------------------EVKEL----TPHPSGLRTAARS-KLRRQEGISRFYI------- 149
Myotis lucifugus CXNS        119 ----------------------EVKEL----TPHPSGLRTAARS-KLRRQEGISRFYI------- 149
Dasypus novemcinctus CXNS    119 ----------------------EVKEL----APHPSGMRTAARS-KLRRQEGISRLYI------- 149
Loxodonta africana CXNS      247 ----------------------EVKDL----TPHPSGLRTASRS-KLRRQEGISRFYI------- 277
Homo sapiens CXNT            107 ----------------------------------------------LQKPI-YTIIYI------- 117
Pan troglodytes CXNT         107 ----------------------------------------------LQKPI-YTIIYI------- 117
Nomascus leucogenys CXNT     110 ----------------------------------------------LQKSI-YTIIYI------- 120
Mus musculus Cxnt            110 ----------------------------------------------LQKPV-YTVIYV------- 120
Rattus norvegicus Cxnt       110 ----------------------------------------------LQKPT-YTVIYI------- 120
Cavia porcellus CXNT         106 ----------------------------------------------LQKPI-YTVIYI------- 116
Oryctolagus cuniculus CXNT   110 ----------------------------------------------LQKPI-YTVIYI------- 120
Bos taurus CXNT              110 ----------------------------------------------LQKPI-YSVIYI------- 120
Vicugna pacos CXNT           110 ----------------------------------------------LQKPI-YTVIYI------- 120
Equus caballus CXNT          110 ----------------------------------------------LQKPI-YSVLYI------- 120
Canis lupus familiaris CXNT  110 ----------------------------------------------LQKPI-YTVIYI------- 120
Myotis lucifugus CXNT        110 ----------------------------------------------LQKPI-YTVVYI------- 120
Dasypus novemcinctus CXNT    110 ----------------------------------------------LQKPA-STVIYI------- 120
Loxodonta africana CXNT      106 ----------------------------------------------LQKPI-YTVIYI------- 116
Homo sapiens CXNU            120 ----------------------------------------------LQVPD-FSAGYI------- 130
Pan troglodytes CXNU         120 ----------------------------------------------LQVPD-FSAGYI------- 130
Pongo abelii CXNU            105 ----------------------------------------------LQVPD-FSAGYI------- 115
Nomascus leucogenys CXNU     117 ----------------------------------------------PQVPD-FSAGYI------- 127
Macaca mulatta CXNU          120 ----------------------------------------------LEVPD-FSAGYI------- 130
Mus musculus Cxnu             90 ----------------------------------------------PQVPD-LSTAYL------- 100
Cavia porcellus CXNU          83 ----------------------------------------------LRVPD-FSCGYL-------  93
Bos taurus CXNU              103 ----------------------------------------------LTVPD-FSSGYV------- 113
Equus caballus CXNU          111 ----------------------------------------------LDVPD-FSCGYM------- 121
Myotis lucifugus CXNU        127 ----------------------------------------------LVVPD-FSYGYI------- 137
Sorex araneus CXNU            99 ----------------------------------------------PAVPD-FTWGYI------- 109
Dasypus novemcinctus CXNU    129 ----------------------------------------------LNIPD-FSSGYI------- 139
Loxodonta africana CXNU      138 ----------------------------------------------LDIPD-FSSGYI------- 148
```

```
Homo sapiens CXNA               134 -----SLVFKASVDI-AFLYVFHSFYPK--YILPPV-VKC-HADPCPN----IVDCFISKPSEKN 184
                                      460       470       480       490       500       510       520
Homo sapiens CXNA               134 -----SLVFKASVDI-AFLYVFHSFYPK--YILPPV-VKC-HADPCPN----IVDCFISKPSEKN 184
Pan troglodytes CXNA            134 -----SLVFKASVDI-AFLYVFHSFYPK--YILPPV-VKC-HADPCPN----IVDCFIAKPSEKN 184
Gorilla gorilla CXNA            134 -----SLVFKASVDI-AFLYVFHSFYPK--YILPPV-VKC-HADPCPN----IVDCFIAKPSEKN 184
Pongo abelii CXNA               134 -----SLVFKASVDI-AFLYVFHSFYPK--YILPPV-VKC-HADPCPN----IVDCFISKPSEKN 184
Nomascus leucogenys CXNA        134 -----SLVFKASVDI-AFLYVFHSFYPK--YILPPV-VKC-HADPCPN----IVDCFISKPSEKN 184
Macaca mulatta CXNA             134 -----SLVFKASVDI-AFLYVFHSLYPK--YILPPV-VKC-HADPCPN----IVDCFISKPSEKN 184
Callithrix jacchus CXNA         134 -----SLVFKASVDI-AFLYVFHSFYPK--YILPPV-VKC-HADPCPN----TVDCFISKPSEKN 184
Mus musculus Cxna               132 -----SLSFKATIDI-IFLYLFHAFYPR--YTLPSM-VKC-HAEPCPN----TVDCFIAKPSEKN 182
Rattus norvegicus Cxna          132 -----SLLFKATIDI-IFLYLFHAFYPR--YTLPSM-VKC-HSAPCPN----TVDCFIAKPSEKN 182
Cavia porcellus CXNA            184 -----SLVFKAGIDV-TFLYVFHSFYPR--YTLPPV-VKC-HIAPCPN----TVDCFISKPSEKN 234
Ochotona princeps CXNA          134 -----SLVFKAGVDA-AFLYVFHSFYPN--YTLPPM-VKC-HAAPCPN----TVDCFIAKPSEKN 184
Bos taurus CXNA                 134 -----SLVFKAGVDA-TFLYVFHSIYPR--YTLPRV-VKC-QVAPCPN----VVDCFISKPEEKN 184
Equus caballus CXNA             134 -----SLVFKASVDA-TFLYVFHSFYPK--YTLPRV-VKC-HAAPCPN----SVDCFISKPSEKN 184
Canis lupus familiaris CXNA     134 -----SLVCKAGVDA-AFLCVFHSFYPK--YTLPRV-VKC-HIAPCPN----TVDCFISKPSEKN 184
Felis catus CXNA                134 -----SLAFKAGVDT-AFLYVFHSFYPK--YTLPHV-VKC-HAAPCPN----TVDCFISKPSEKN 184
Myotis lucifugus CXNA           166 -----SLVFKASIDI-IFLYVFHSFYPK--YTLPPV-VKC-HAAPCPN----IVDCFISKPSEKN 216
Dasypus novemcinctus CXNA       134 -----SLVCKVGVDA-AFLYMFHSFYPK--YTLPHV-VQC-HVAPCPN----SVDCFISKPSEKN 184
Loxodonta africana CXNA         134 -----SLVFKAGVDA-AFLYVFHSFYPK--YTLPPV-VKC-HVAPCPN----IVDCFISKPSEKN 184
Homo sapiens CXNB               133 ----LSLIFKAAVDA-GFLYIFHRLY-K-DYDMPRV-VAC-SVEPCPH----TVDCYISRPTEKK 184
Gorilla gorilla CXNB            133 ----LSLIFKAAVDA-GFLYIFHRLY-K-DYDMPRV-VAC-SVEPCPH----TVDCYISRPTEKK 184
Nomascus leucogenys CXNB        133 ----LSLIFKAAVDA-GFLYIFHRLY-K-DYDMPRV-VAC-SVEPCPH----TVDCYISRPTEKK 184
Macaca mulatta CXNB             133 ----LSLIFKAAVDA-GFLYIFHRLY-K-DYDMPRV-VAC-SVEPCPH----TVDCYISRPTEKK 184
Callithrix jacchus CXNB         133 ----LSLIFKAAVDT-GFLYIFHRLY-K-DYDMPRV-VAC-SVEPCPH----TVDCYISRPTEKK 184
Otolemur garnettii CXNB         133 ----LSLIFKAAVDS-TFLYIFHRLY-R-DYDMPRV-VAC-SVAPCPH----TVDCYISRPTEKK 184
Mus musculus Cxnb               133 ----LSLIFKAAVDS-GFLYIFHCIY-K-DYDMPRV-VAC-SVTPCPH----TVDCYIARPTEKK 184
Rattus norvegicus Cxnb          133 ----LSLIFKAAVDS-GFLYIFHCIY-K-DYDMPRV-VAC-SVQPCPH----TVDCYISRPTEKK 184
Dipodomys ordii CXNB            133 ----LSLIFKAAVDS-GFLYIFHHLY-R-DYDMPRV-VAC-SVDPCPH----TVDCYISRPTEKK 184
Cavia porcellus CXNB            133 ----VSLIFKVAMDS-AFLYVFHRLY-K-NYDMPRV-VHC-SIDPCPH----TVDCYISRPTEKK 184
Oryctolagus cuniculus CXNB       41 ----LSLIFKASVDS-GFLYIFHYLY-Q-DYDMPRV-VAC-SVAPCPH----TVDCYISRPTEKK  92
Ochotona princeps CXNB          133 ----LSLIFKAAVDS-AFLYIFHRLY-Q-DYDMPRV-VAC-SVAPCPH----TVNCYISRPTEKK 184
Bos taurus CXNB                 133 ----LSLLFKAAVDV-SFLYIFHRLY-K-DYDMPRV-VAC-SESPCPH----TVDCYISRPTEKK 184
Equus caballus CXNB             133 ----LSLIFKAAVDS-GFLYIFHRLY-Q-NYDMPRV-VAC-SESPCPH----TVDCYISRPTEKK 184
Canis lupus familiaris CXNB     183 ----LSLIFKAAVDS-GFLYIFHRLY-Q-DYDMPRV-VAC-SEAPCPH----TVDCYISRPTEKK 234
Myotis lucifugus CXNB           133 ----LSLIFKAAVDS-GFLYTFHRLY-QGHYDMPRV-VAC-SQAPCPH----TVDCYISRPTEKR 185
Erinaceus europaeus CXNB        133 ----LSLIFKASVDS-GFLFIFHRLY-Q-NYDMPHV-VAC-TQSPCPH----TVDCFISRPTEKK 184
Loxodonta africana CXNB         133 ----LSLIFKATVDA-SFLYIFHRLY-K-DYDMPRV-VAC-SVAPCPH----TVDCFISRPTEKK 184
Homo sapiens CXNC               133 ----FSLIFKLIIEF-LFLYLLHTLW-H-GFNMPRL-VQCANVAPCPN----IVDCYIARPTEKK 185
Gorilla gorilla CXNC            133 ----FSLIFKLIIEF-LFLYLLHTLW-H-GFNMPRL-VQCANVAPCPN----IVDCYIARPTEKK 185
Pongo abelii CXNC               133 ----FSLIFKLIIEF-LFLYLLHTLW-H-GFDMPRL-VQCANVAPCPN----IVDCYIARPTEKK 185
Nomascus leucogenys CXNC        133 ----FSLIFKLIIEL-LFLYLLHTLW-H-GFNMPRL-VQCANVAPCPN----IVDCYIARPTEKK 185
Callithrix jacchus CXNC         133 ----FSLIFKLIIEF-LFLYLLHTLW-H-GFDMPRL-VQCANVAPCPN----IVDCYIARPTEKK 185
Microcebus murinus CXNC         163 ----FSLIFKLLIEL-LFLYMLHTLW-Y-GFGMPRL-VQCANVAPCPN----TVDCYIARPTEKK 215
Otolemur garnettii CXNC         133 ----LSLVFKLLIEL-LFLYVLHTLW-Y-GFGMPRL-VQCVNVAPCPN----TVDCYIARPTEKK 185
Mus musculus Cxnc               133 ----FSLIFKLIIEL-VFLYVLHTLW-H-GFTMPRL-VQCASIVPCPN----TVDCYIARPTEKK 185
Rattus norvegicus Cxnc          133 ----FSLIFKLIIEL-VFLYVLHTLW-H-GFTMPRL-VQCASVVPCPN----TVDCYIARPTEKK 185
Cavia porcellus CXNC            133 ----FSLVFKLIIEL-LFLYVLHTLW-H-GFGMPRL-VQCAGVAPCPN----TVDCYIARPTEKK 185
Oryctolagus cuniculus CXNC      243 ----LSLVFKLIIEF-LFLYVLHTLW-H-GFGMPRL-VQCANVAPCPN----VVDCYIARPTEKK 295
Bos taurus CXNC                 133 ----LSLVFKLLIEF-LFLYLLHTLW-Y-GFGMPRL-VQCANVAPCPN----TVDCYIARPTEKK 185
Equus caballus CXNC             133 ----FSLIFKLIIEF-LFLYLLHTLW-Y-GFGMPRL-VQCTNVAPCPN----TVDCYIARPTEKK 185
Canis lupus familiaris CXNC     133 ----LSLVFKLAIEL-LFLYVLHTLW-R-GFAMPRL-VQCANVAPCPN----VVDCYIARPTEKK 185
Felis catus CXNC                133 ----FSLIFKLLIEF-LFLYVLHTLW-H-GFSMPRL-VQCDNMAPCPN----IVDCYIARPTEKK 185
Myotis lucifugus CXNC           133 ----LSLIFKLIIEF-VFLYVLHTLW-Y-GFSMPRL-VQCSNVAPCPN----TVDCYIARPTEKK 185
Dasypus novemcinctus CXNC       133 ----LSLVFKLIMEL-LFLYVLHTLW-H-GFGMPRL-VQCASVAPCPN----TVDCYIARPTEKK 185
Loxodonta africana CXNC         133 ----FSLVFKLVIEF-LFLYVLHTLW-H-GFGMPRL-VQCSNVAPCPN----TVDCYIARPTEKK 185
Homo sapiens CXND               126 ----ISLIVKTGFEI-GFLVLFYKLY-D-GFSVPYL-IKC-DLKPCPN----TVDCFISKPTEKT 177
Pan troglodytes CXND            126 ----ISLIVKTGFEI-GFLVLFYKLY-D-GFSVPYL-IKC-DLKPCPN----TVDCFISKPTEKT 177
Macaca mulatta CXND             126 ----ISLIVKTGFEI-GFLVLFYKLY-D-GFSVPYL-IKC-DLKPCPN----TVDCFISRPTEKT 177
Callithrix jacchus CXND         126 ----ISLIVKTGFEI-GFLVLFYNVY-D-GFSVPYL-IKC-DLKPCPN----TVDCFISKPTEKM 177
Dipodomys ordii CXND            126 ----ISLIVKTGFEI-GFLVLFCKLY-G-GFSVSHI-MKC-DLKPCPN----TVDCFISKPTEKT 177
Oryctolagus cuniculus CXND      126 ----ISLIVKTGFEI-GFLVLFYKLY-D-GFRVPYL-LKC-DLQPCPN----TVDCFISRPTEKT 177
Tursiops truncatus CXND         126 ----ISLIVKTGFEI-GFLVLFYKLY-G-GFSVPYF-MKC-DLKPCPN----TVDCFVSKPTEKT 177
Bos taurus CXND                  44 ----ISLMVKTGFEI-GFLVLFYKLY-D-GFRVPYL-LKC-DLKPCPN----TVDCFVSKPTEKT  95
Equus caballus CXND             145 ----ISLIVKTGFEI-GFLVLFYKLY-D-GFSVPYL-VKC-DLKPCPN----TVDCFISKPTEKT 196
Canis lupus familiaris CXND     126 ----ISLIVKTGFEI-GFLVLFYKLY-N-GFSVPYL-MKC-ALKPCPN----TVDCFISKPTEKT 177
Myotis lucifugus CXND           126 ----TSLIVKLGFEI-GFLVLFYKLY-D-GFSVPYL-MKC-DLKPCPN----TVDCFMSRPTEKT 177
Erinaceus europaeus CXND        126 ----VSLIVKTGFEI-GFLVLFYKIY-D-GFSVPYL-MKC-DLKPCPN----TVDCFISKATEKT 177
Dasypus novemcinctus CXND       126 ----ISLIVKTGFEM-GFLVLFYKMY-D-GFSVPYL-VKC-DLKPCPN----TVDCFISKPTEKT 177
Choloepus hoffmanni CXND        126 ----ISLIVKTGFEM-GFLVLFYKMY-D-GFSVPYL-VKC-DLKPCPN----TVDCFISKPTEKT 177
Loxodonta africana CXND         126 ----ISLIVKTGFEI-GFLVLFYKLY-D-GFNVPYL-VKC-DLKPCPN----TVDCFISKPTEKT 177
Homo sapiens CXNE               138 ----SSIFFRVIFEA-AFMYVFYVMY-D-GFSMQRL-VKC-NAWPCPN----TVDCFVSRPTEKT 189
Pan troglodytes CXNE            138 ----SSIFFRVIFEA-AFMYVFYVMY-D-GFSMQRL-VKC-NAWPCPN----TVDCFVSRPTEKT 189
Pongo abelii CXNE               138 ----SSIFFRVIFEA-AFMYVFYVMY-D-GFSMQRL-VKC-NAWPCPN----TVDCFVSRPTEKT 189
Macaca mulatta CXNE             138 ----SSIFFRVVFEA-AFMYVFYVMY-D-GFSMQRL-VKC-NAWPCPN----TVDCFVSRPTEKT 189
Papio hamadryas CXNE            138 ----SSIFFRVVFEA-AFMYVFYVMY-D-GFSMQRL-VKC-NAWPCPN----TVDCFVSRPTEKT 189
Callithrix jacchus CXNE         138 ----SSIFFRVVFEA-VFMYVFYIMY-D-GFAMQRL-VKC-NAWPCPN----TVDCFVSRPTEKT 189
Otolemur garnettii CXNE         138 ----SSIFFRVVFEA-VFMYVFYIMY-D-GFSMQRL-VKC-NAWPCPN----TVDCFVSRPTEKT 189
Mus musculus Cxne               151 ----TSIFFRVIFEA-VFMYVFYIMY-N-GFFMQRL-VKC-NAWPCPN----TVDCFISRPTEKT 202
```

Sequence	Start	Alignment	End
Rattus norvegicus Cxne	157	----TSIFFRVIFEA-VFMYVFYIMY-N-GFFMQRL-VKC-NAWPCPN----TVDCFISRPTEKT	208
Dipodomys ordii CXNE	157	----SSVFFRVIFEA-VFMYVFYVMY-N-GFFMQRL-VKC-NAWPCPN----TVDCFISRPTEKT	208
Cavia porcellus CXNE	185	----TSIFFRVIFEG-AFMYVFYIMY-N-GFFMQRL-VKC-NAWPCPN----TVDCFISRPTEKT	236
Oryctolagus cuniculus CXNE	157	----SSIFFRVIFEA-VFMYVFYIMY-N-GFSMQRL-VKC-NAWPCPN----TVDCFVSRPTEKT	208
Equus caballus CXNE	138	----SSIFFRVIFEA-VFMYVFYVMY-D-GFAMQRL-VKC-NAWPCPN----TVDCFVSRPTEKT	189
Canis lupus familiaris CXNE	138	----SSIFFRVIFEA-VFMYVFYVMY-D-GFSMQRL-VKC-NAWPCPN----TVDCFVSRPTEKT	189
Felis catus CXNE	138	----SSIFFRVIFEA-VFMYVFYIMY-D-GFSMQRL-VKC-NAWPCPN----TVDCFVSRPTEKT	189
Myotis lucifugus CXNE	138	----SSIFFRVIFEA-VFMYVFYIMY-D-GFSMQRL-VKC-NAWPCPN----TVDCFVSRPTEKT	189
Sorex araneus CXNE	138	----SSIFFRIIFEA-VFMYVFYIMY-D-GFYMKRL-VKC-TNWPCPN----TVDCFVSRPTEKT	189
Dasypus novemcinctus CXNE	138	----SSIFFRVIFEA-AFMYVFYIMY-D-GFAMQRL-VKC-SSWPCPN----TVDCFVSRPTEKT	189
Loxodonta africana CXNE	138	----SSIFFRVIFEA-VFMYVFYVMY-D-GFSMKRL-VKC-SAWPCPN----TVDCFVSRPTEKT	189
Homo sapiens CXNF	138	----SSIFFRIIFEA-AFMYVFYFLY-N-GYHLPWV-LKC-GIDPCPN----LVDCFISRPTEKT	189
Pan troglodytes CXNF	138	----SSIFFRIIFEA-AFMYVFYFLY-N-GYHLPWV-LKC-GIDPCPN----LVDCFISRPTEKT	189
Gorilla gorilla CXNF	138	----SSIFFRIIFEA-AFMYVFYFLY-N-GYHLPWV-LKC-GIDPCPN----LVDCFISRPTEKT	189
Nomascus leucogenys CXNF	138	----SSIFFRIIFEA-AFMYVFYFLY-N-GYHLPWV-LKC-GIDPCPN----LVDCFISRPTEKT	189
Callithrix jacchus CXNF	138	----SSIFFRIIFEA-AFMYVFYFLY-S-GYHLPWV-LKC-GIDPCPN----LVDCFISRPTEKT	189
Mus musculus Cxnf	138	----SSIFFRIIFEA-AFMYVFYFLY-N-GYHLPWV-LKC-GIDPCPN----LVDCFISRPTEKT	189
Rattus norvegicus Cxnf	138	----SSIFFRIIFEA-AFMYVFYFLY-N-GYHLPWV-LKC-GIDPCPN----LVDCFISRPTEKT	189
S.tridecemlineatus CXNF	138	----SSIFFRIIFEA-AFMYVFYFLY-N-GYHLPWV-LKC-GIEPCPN----LVDCFISRPTEKT	189
Oryctolagus cuniculus CXNF	138	----SSIFFRIIFEA-SFMYVFYFLY-N-GYHLPWV-LKC-GIEPCPN----LVDCFISRPTEKT	189
Ochotona princeps CXNF	138	----GSIFFRIIFEA-SFMYVFYFLY-N-GYHLPWV-LKC-GIEPCPN----LVDCFISRPTEKT	189
Bos taurus CXNF	138	----SSIFFRIVFEA-AFMYVFYFLY-N-GYHLPWV-LKC-GIQPCPN----LVDCFISRPTEKT	189
Equus caballus CXNF	138	----SSIFFRIIFEA-AFMYVFYFLY-N-GYHLPWV-LKC-GIDPCPN----LVDCFISRPTEKT	189
Canis lupus familiaris CXNF	138	----SSIFFRIIFEA-SFMYVFYFLY-N-GYHLPWV-LKC-GIDPCPN----LVDCFISRPTEKT	189
Myotis lucifugus CXNF	138	----SSIFFRIIFEA-SFMYVFYFLY-N-GYHLPWV-LKC-GVDPCPN----LVDCFISRPTEKT	189
Dasypus novemcinctus CXNF	138	----SSIIFRIIFEA-CFMYVFYFLY-N-GYHLPWV-LKC-GIDPCPN----IVDCFISRPTEKT	189
Choloepus hoffmanni CXNF	138	----SSIFFRIIFEA-CFMYVFYFLY-N-GYHLPWV-LQC-GIDPCPN----LVDCFISRPTEKT	189
Loxodonta africana CXNF	138	----SSIFFRIIFEA-SFMYVFYFLY-N-GYHLPWV-LKC-GIDPCPN----LVDCFISRPTEKT	189
Homo sapiens CXNG	137	----ISVVFRLLFEA-VFMYVFYLLYP--GYAMVRL-VKC-DVYPCPN----TVDCFVSRPTEKT	188
Pan troglodytes CXNG	137	----ISVVFRLLFEA-VFMYVFYLLYP--GYAMVRL-VKC-DVYPCPN----TVDCFVSRPTEKT	188
Gorilla gorilla CXNG	137	----ISVVFRLLFEA-VFMYVFYLLYP--GYAMVRL-VKC-DVYPCPN----TVDCFVSRPTEKT	188
Pongo abelii CXNG	137	----ISVVFRLLFEA-VFMYVFYLLYP--GYAMVRL-VKC-DVYPCPN----TVDCFVSRPTEKT	188
Nomascus leucogenys CXNG	137	----ISVVFRLLFEA-VFMYVFYLLYP--GYAMVRL-VKC-DVYPCPN----TVDCFVSRPTEKT	188
Macaca mulatta CXNG	137	----ISVVFRLLFEA-VFMYVFYLLYP--GYAMVRL-VKC-DVYPCPN----TVDCFVSRPTEKT	188
Papio hamadryas CXNG	137	----ISVVFRLLFEA-VFMYVFYLLYP--GYAMVRL-VKC-DVYPCPN----TVDCFVSRPTEKT	188
Callithrix jacchus CXNG	137	----ISVVFRLLFEA-VFMYVFYLLYP--GYAMVRL-VKC-DVYPCPN----TVDCFVSRPTEKT	188
Microcebus murinus CXNG	137	----ISVVFRLLFEA-VFMYVFYLLYP--GYAMVRL-VKC-EAYPCPN----TVDCFVSRPTEKT	188
Mus musculus Cxng	137	----ISVVFRLLFEA-VFMYVFYLLYP--GYAMVRL-VKC-EAFPCPN----TVDCFVSRPTEKT	188
Rattus norvegicus Cxng	137	----ISVVFRLLFEA-VFMYVFYLLYP--GYAMVRL-VKC-EAFPCPN----TVDCFVSRPTEKT	188
Oryctolagus cuniculus CXNG	137	----ISVVFRLLFEA-VFMYVFYLLYP--GYAMVRL-VKC-EAYPCPN----TVDCFVSRPTEKT	188
Bos taurus CXNG	137	----ISVVFRLLFEA-AFMYVFYLLYP--GYAMVRL-VKC-DAYPCPN----TVDCFVSRPTEKT	188
Equus caballus CXNG	137	----ISVVFRLLFEA-AFMYVFYLLYP--GYAMVRL-VKC-DAYPCPN----TVDCFVSRPTEKT	188
Canis lupus familiaris CXNG	137	----ISVVFRLLFEA-AFMYVFYLLYP--GYAMVRL-VKC-EAYPCPN----TVDCFVSRPTEKT	188
Felis catus CXNG	137	----ISVVFRLLFEA-AFMYVFYLLYP--GYAMVRL-VKC-EAYPCPN----TVDCFVSRPTEKT	188
Myotis lucifugus CXNG	137	----ISVVFRLMFEA-AFMYVFYLLYP--GYAMVRL-VKC-EAYPCPN----TVDCFVSRPTEKT	188
Pteropus vampyrus CXNG	137	----ISVVFRLLFEA-AFMYVFYLLYP--GYAMVRL-VKC-EAYPCPN----TVDCFVSRPTEKT	188
Dasypus novemcinctus CXNG	137	----ISVVFRLLFEA-VFMYVFYLLYP--GYTMVRL-VKC-EAYPCPN----TVDCFVSRPTEKT	188
Loxodonta africana CXNG	137	----ISVVFRLLFEA-VFMYVFYLLYP--GYAMVRL-VKC-EAYPCPN----TVDCFVSRPTEKT	188
Procavia capensis CXNG	137	----ISVVFRLLFEA-VFMYVFYLLYP--GYAMVRL-VKC-EAYPCPN----TVDCFVSRPTEKT	188
Homo sapiens CXNH1	162	---------KSVLEA-GFLYGQWRLY---GWTMEPV-FVC-QRAPCPY----LVDCFVSRPTEKT	207
Pan troglodytes CXNH1	162	---------KSVLEA-GFLYGQWRLY---GWTMEPV-FVC-QRAPCPY----LVDCFVSRPTEKT	207
Gorilla gorilla CXNH1	162	---------KSVLEA-GFLYGQWRLY---GWTMEPV-FVC-QRAPCPY----LVDCFVSRPTEKT	207
Pongo abelii CXNH1	162	---------KSVLEA-GFLYGQWRLY---GWTMEPV-FVC-QRAPCPY----LVDCFVSRPTEKT	207
Nomascus leucogenys CXNH1	162	---------KSVLEA-GFLYGQWRLY---GWTMEPV-FVC-QRAPCPY----LVDCFVSRPTEKT	207
Macaca mulatta CXNH1	162	---------KSVLEA-GFLYGQWRLY---GWTMEPV-FVC-QRAPCPY----LVDCFVSRPTEKT	207
Otolemur garnettii CXNH1	158	---------RSVLEA-GFLYGQWHIY---GWTMEPA-FSC-HRAPCPY----VVECFVSHPLAKT	203
Mus musculus Cxnh1	162	---------KSVLEA-GFLYGQWRLY---GWTMEPV-FVC-QRAPCPH----IVDCYVSRPTEKT	207
Rattus norvegicus Cxnh1	162	---------KSVLEA-GFLYGQWRLY---GWTMEPV-FVC-QRAPCPH----VVDCYVSRPTEKT	207
Cavia porcellus CXNH1	162	---------KSLLEA-GFLYGQWRLY---GWTMEPL-YVC-QRAPCPH----RVDCYVSRPTEKT	207
Oryctolagus cuniculus CXNH1	162	---------KSVLEA-GFLYGQWRLY---GWTMAPV-FVC-QRAPCPH----RVDCFVSRPTEKT	207
Ochotona princeps CXNH1	176	---------KSVLEA-GFLYGQWRLY---GWTMEPV-FVC-QRAPCPY----LVDCFVSRPTEKT	221
Equus caballus CXNH1	162	---------KSVLEA-GFLYGQWRLY---GWTMEPV-FVC-QRPPCPH----LVDCFVSRPTEKT	207
Canis lupus familiaris CXNH1	162	---------KSVLEA-GFLYGQWRLY---GWTMEPV-FVC-QRAPCPY----LVDCFVSRPTEKT	207
Felis catus CXNH1	162	---------KSVLEA-GFLYGQWRLY---GWTMEPV-FVC-QRAPCPY----LVDCFVSRPTEKT	207
Myotis lucifugus CXNH1	194	---------KSILEA-GFLYGQWRLY---GWTMAPV-YVC-HHAPCPH----SVDCYVSRPTEKT	239
Dasypus novemcinctus CXNH1	162	---------KSVLEA-GFLYGQWRLY---GWTMAPT-FVC-QRAPCPH----QVDCFVSRPTEKT	207
Loxodonta africana CXNH1	162	---------KSVLEA-GFLYGQWRLY---GWTMEPV-FVC-QRAPCPY----LVDCFVSRPTEKT	207
Homo sapiens CXNI	156	-----SILIRTTMEV-GFIVGQYFIY---GIFLTTL-HVC-RRSPCPH----PVNCYVSRPTEKN	205
Pan troglodytes CXNI	156	-----SILIRTTMEV-GFIVGQYFIY---GIFLTTL-HVC-RRSPCPH----PVNCYVSRPTEKN	205
Pongo abelii CXNI	156	-----SILIRTTMEV-GFIVGQYFIY---GIFLTTL-HVC-RRSPCPH----PVNCYVSRPTEKN	205
Nomascus leucogenys CXNI	156	-----SILIRTTMEV-GFIVGQYFIY---GIFLTTL-HVC-RRSPCPH----PVNCYVSRPTEKN	205
Macaca mulatta CXNI	156	-----SILIRTTMEV-GFIVGQYLIY---GIFLTTL-HVC-RRSPCPH----PVNCYVSRPTEKN	205
Papio hamadryas CXNI	156	-----SILIRTTMEV-GFIVGQYLIY---GIFLTTL-HVC-RRSPCPH----PVNCYVSRPTEKN	205
Callithrix jacchus CXNI	156	-----SILIRTTMEV-GFIVGQYLIY---GVFLTTL-HVC-RRSPCPH----PVNCYVSRPTEKN	205
Otolemur garnettii CXNI	155	-----SILIRTTMEV-AFIVGQYLLY---GIFLSTL-HVC-RRSPCPH----PVNCYVSRPTEKN	204
Mus musculus Cxni	156	-----TILIRTTMEV-AFIVGQYLLY---GIFLDTL-HVC-RRSPCPH----PVNCYVSRPTEKN	205
Rattus norvegicus Cxni	189	-----TILIRTAMEV-AFMVGQYLLY---GIFLDTL-HVC-RRSPCPH----PVNCYVSRPTEKN	238
Cavia porcellus CXNI	157	-----TILIRTAMEV-AFIVGQYFLY---GIFLNTL-HVC-RRSPCPH----PVNCYVSRPTEKN	206
Oryctolagus cuniculus CXNI	156	-----SIVIRTTVEV-AFIVGQYLLY---GIFLHTL-HVC-RRSPCPH----PVNCYVSRPTEKN	205

```
Bos taurus CXNI                 157 -----SILIRTTMEV-AFIVGQYLLY---GVFLDTL-HVC-RRSPCPH----PVNCYVSRPTEKN 206
Equus caballus CXNI             156 -----SILIRTTMEV-AFIVGQYLLY---GIFLDTL-HVC-RRSPCPH----PVNCYVSRPTEKN 205
Canis lupus familiaris CXNI     156 -----SILIRTTMEV-AFIVGQYLLY---GIFLDTL-HVC-RRSPCPH----PVNCYVSRPTEKN 205
Felis catus CXNI                156 -----SILIRTTMEV-AFIVGQYLLY---GIFLDTL-HVC-RRSPCPH----PVNCYVSRPTEKN 205
Pteropus vampyrus CXNI          156 -----SILIRTTMEV-AFIVGQYLLY---GVFLDTL-HVC-RRSPCPH----PVNCYVSRPTEKN 205
Erinaceus europaeus CXNI        158 -----SILIRTTMEV-AFIVGQYLLY---GIFLDTL-HIC-RRSPCPH----PVNCYVSRPTEKN 207
Dasypus novemcinctus CXNI       156 -----SILIRTTMEV-AFIVGQYLLY---GIFLDTL-HVC-RRSPCPH----PVNCYVSRPTEKN 205
Loxodonta africana CXNI         156 -----SILIRTTMEV-TFIVVQYLLY---GIFLDTL-HVC-RRSPCPH----PVNCYVSRPTEKN 205
Homo sapiens CXNJ1              151 ----FNIIFKTLFEV-GFIAGQYFLY---GFELKPL-YRC-DRWPCPN----TVDCFISRPTEKT 201
Pongo abelii CXNJ1              151 ----FNIIFKTLFEV-GFIAGQYFLY---GFELKPL-YRC-DRWPCPN----TVDCFISRPTEKT 201
Macaca mulatta CXNJ1            151 ----FNIIFKTLLEV-GFIAGQYFLY---GFELQPL-YRC-DRWPCPN----TVDCFISRPTEKT 201
Mus musculus Cxnj1              157 ----FNIIFKTLFEV-GFIAGQYFLY---GFQLQPL-YRC-DRWPCPN----TVDCFISRPTEKT 207
Rattus norvegicus Cxnj1         157 ----FNIIFKTLFEV-GFIAGQYFLY---GFQLQPL-YRC-DRWPCPN----TVDCFISRPTEKT 207
Bos taurus CXNJ1                147 ----FNIIFKTLFEV-GFIAGQYFLY---GFQLKPL-YRC-DRWPCPN----TVDCFISRPTEKT 197
Equus caballus CXNJ1            158 ----FNIIFKTLFEV-GFIAGQYFLY---GFQLKPL-YRC-DRWPCPN----TVDCFISRPTEKT 208
Myotis lucifugus CXNJ1          147 ----FNIIFKMLFEV-GFIVGQYYLY---GFQLKPL-YHC-NRWPCPN----MVDCFISRPTEKT 197
Myotis lucifugus CXNJ2          159 ----FNIIFKTLFEV-GFIAGQYFLY---GFELKPL-YHC-DRWPCPN----IVDCFISRPTEKT 209
Pteropus vampyrus CXNJ1         316 ----FNIIFKTLFEV-GFIAGQYFLY---GFQLNPL-YRC-DRWPCPN----TVDCFISRPTEKT 366
Pteropus vampyrus CXNJ2         238 ----FTTIFKMLFEV-GFIAGQYFLY---GFQLKPN-YVC-AQRPCPN----AVDCFISRPTEKT 288
Sorex araneus CXNJ1             137 ----LNVLFRTLLEV-GFIAGQYCLY---GFQLQPL-FHC-DRWPCPN----TVDCFISRPTEKT 187
Homo sapiens CXNK1              157 ----ISILFKSIFEV-T-LLIQWYIY---GFSLSAV-HTC-KRDPCPH----QVDCFLSCPMEKT 206
Homo sapiens CXNK2              157 ----ISILFKSIFEV-AFLLIQWYIY---GFSLSAV-YTC-KRDPCPH----QVDCFLSRPTEKT 207
Pan troglodytes CXNK1           157 ----ISILFKSIFEV-A-LLIQWYIY---GFSLSAV-HTC-KRDPCPH----QVDCFLSCPMEKT 206
Pan troglodytes CXNK2           157 ----ISILFKSIFEV-AFLLIQWYIY---GFSLSAV-YTC-KRDPCPH----QVDCFLSRPTEKT 207
Pongo abelii CXNK1              157 ----ISILFKSIFEV-AFLLIQWYIY---GFSLSAV-YTC-KRDPCPH----QVDCFLSRPTEKT 207
Nomascus leucogenys CXNK1       157 ----ISILFKSIFEV-AFLLIQWYIY---GFSLSAV-YTC-KRDPCPH----QVDCFLSRPTEKT 207
Callithrix jacchus CXNK1        157 ----ISILFKSVFEV-AFLLIQWYIY---GFSLSAV-YTC-KRDPCPH----QVDCFLSRPTEKT 207
Mus musculus Cxnk1              157 ----ISILFKSVFEV-AFLLIQWYIY---GFSLSAV-YTC-KRDPCPH----QVDCFLSRPTEKT 207
Mus musculus Cxnk2              157 ----ASIFFKSVFEM-AFLLIQWYIY---GFTLSAL-YIC-EQSPCPR----RVDCFLSRPTEKT 207
Rattus norvegicus Cxnk1         157 ----ISILFKSVFEV-AFLLIQWYIY---GFSLSAV-YTC-KRDPCPH----QVDCFLSRPTEKT 207
Rattus norvegicus Cxnk2         158 ----ASIFFKSVFEV-AFLLIQWYLY---GFTLSAV-YIC-EQSPCPH----RVDCFLSRPTEKT 208
Cavia porcellus CXNK1           157 ----ISILFKSVFEV-AFLLIQWYIY---GFSLSAV-YTC-KRDPCPH----QVDCFLSRPTEKT 207
Oryctolagus cuniculus CXNK1     157 ----ISILFKSVFEV-AFLLIQWYIY---GFSLSAV-YTC-KRDPCPH----QVDCFLSRPTEKT 207
Bos taurus CXNK1                158 ----ISILFKSVFEV-AFLLIQWYIY---GFSLSAV-YTC-KRDPCPH----QVDCFLSRPTEKT 208
Vicugna pacos CXNK1             157 ----ISILFKSVFEV-AFLLIQWYIY---GFSLSAV-YTC-KRDPCPH----QVDCFLSRPTEKT 207
Equus caballus CXNK1            157 ----ISILFKSLFEV-AFLLIQWYIY---GFSLSAV-YTC-KRDPCPH----QVDCFLSRPTEKT 207
Equus caballus CXNK2            134 ----ISIFFRSLFEV-AFLLIQWYLY---GFSLNAI-YTC-KRDPCPH----QVDCFLSRPTEKS 184
Canis lupus familiaris CXNK1    157 ----ISILFKSVFEV-AFLLIQWYIY---GFSLSAV-YTC-KREPCPH----QVDCFLSRPTEKT 207
Canis lupus familiaris CXNK2    160 ----ISVFFKAVFEV-AFLLIQWYIY---GFSLNAV-YSC-KRDPCPH----QVDCFLSRPTEKS 210
Felis catus CXNK1               155 ----VSIFFKSLLEV-AFLLIQWYIY---GFSLNAV-YTC-KRDPCPH----QVDCFLSRPTEKN 205
Myotis lucifugus CXNK1          157 ----ISILFKSVFEV-AFLLIQWYIY---GFTLSAV-YTC-KRDPCPH----QVDCFLSRPTEKT 207
Dasypus novemcinctus CXNK1      157 ----ISIFFKSVFEV-TFLLIQWYIY---GFSLSAV-YTC-KRDPCPH----QVDCFLSRPTEKS 207
Dasypus novemcinctus CXNK2      157 ----ISILFKSIFEV-AFLLIQWYIY---GFSLSAV-YTC-KRAPCPH----QVDCFLSRPTEKT 207
Loxodonta africana CXNK1        157 ----ISILFKSIFEV-AFLLIQWYIY---GFSLSAV-YTC-KRDPCPH----QVDCFLSRPTEKT 207
Loxodonta africana CXNK2        157 ----ISIVFNSLFEV-AFLLIQWYLY---GFSLSAV-YTC-KRDPCPH----QVDCFLSRPTEKS 207
Homo sapiens CXNL               154 -----HIIFKTLFEV-GFIVGHYFLY---GFRILPL-YRC-SRWPCPN----VVDCFVSRPTEKT 203
Pan troglodytes CXNL            154 -----HIIFKTLFEV-GFIVGHYFLY---GFRILPL-YRC-SRWPCPN----VVDCFVSRPTEKT 203
Pongo abelii CXNL               154 -----HIIFKTLFEV-GFIVGHYFLY---GFRILPL-YRC-SRWPCPN----VVDCFVSRPTEKT 203
Nomascus leucogenys CXNL        154 -----HIIFKTLFEV-GFIVGHYFLY---GFRILPL-YRC-SRWPCPN----VVDCFVSRPTEKT 203
Macaca mulatta CXNL             160 -----HIIFKTLFEV-GFIVGHYFLY---GFRILPL-YRC-SRWPCPN----VVDCFVSRPTEKT 209
Callithrix jacchus CXNL         158 -----HIIFKTLFEV-GFIVGHYFLY---GFRILPL-YRC-SRWPCPN----VVDCFVSRPTEKT 207
Otolemur garnettii CXNL         205 -----HIIFKTLFEV-GFIVGHYFLY---GFRILPL-YRC-SRWPCPN----VVDCFVSRPTEKT 254
Mus musculus Cxnl               161 -----HIIFKTLFEV-GFIVGHYFLY---GFRILPL-YRC-SRWPCPN----VVDCFVSRPTEKT 210
Rattus norvegicus Cxnl          161 -----HIIFKTLFEV-GFIVGHYFLY---GFRILPL-YRC-SRWPCPN----VVDCFVSRPTEKT 210
Cavia porcellus CXNL            161 -----HIIFKTLFEV-GFIVGHYFLY---GFRILPL-YRC-SRWPCPN----VVDCFVSRPTEKT 210
Oryctolagus cuniculus CXNL      161 -----HIIFKTLFEV-GFIVGHYFLY---GFRILPL-YRC-SRWPCPN----VVDCFVSRPTEKT 210
Ochotona princeps CXNL          158 -----HIIFKTLFEV-GFIVGHYFLY---GFRILPL-YRC-SRWPCPN----VVDCFVSRPTEKT 207
Bos taurus CXNL                 161 -----HIIFKTLFEV-GFIVGHYFLY---GFRILPL-YRC-SRWPCPN----VVDCFVSRPTEKT 210
Equus caballus CXNL             160 -----HIVFKTLFEV-GFIVGHYFLY---GFRILPL-YRC-SRWPCPN----VVDCFVSRPTEKT 209
Canis lupus familiaris CXNL     161 -----HIIFKTLFEV-GFIVGHYFLY---GFRILPL-YRC-SRWPCPN----VVDCFVSRPTEKT 210
Felis catus CXNL                161 -----HIIFKTLFEV-GFVVGHYFLY---GFRILPL-YRC-SRWPCPN----VVDCFVSRPTEKT 210
Pteropus vampyrus CXNL          161 -----HIVFKTLFEV-GFVVGHYFLY---GFRILPL-YRC-SRWPCPN----VVDCFVSRPTEKT 210
Sorex araneus CXNL              130 -----HVIFKTLFEV-AFVVGHYLLY---GFRVLAL-YRC-NQWPCPN----VVDCFVSRPTEKT 179
Dasypus novemcinctus CXNL       161 -----HIIFKTLFEV-GFVVGHYFLY---GFQILPL-YRC-SRWPCPN----VVDCFVSRPTEKT 210
Loxodonta africana CXNL         160 -----HIIFKTLFEV-GFIVGHYFLY---GFRILPL-YRC-SRWPCPN----VVDCFVSRPTEKT 209
Homo sapiens CXNM               156 -TYVIHIFTRSVVEV-GFMIGQYLLY---GFHLEPL-FKC-HGHPCPN----IIDCFVSRPTEKT 209
Pan troglodytes CXNM            156 -TYVIHIFTRSVVEV-GFMIGQYLLY---GFHLEPL-FKC-HGHPCPN----IIDCFVSRPTEKT 209
Pongo abelii CXNM               156 -TYVIHIFTRSVVEV-GFMIGQYLLY---GFHLEPL-FKC-HGHPCPN----IIDCFVSRPTEKT 209
Nomascus leucogenys CXNM        156 -TYVIHIFTRSVVEV-GFMIGQYLLY---GFHLEPL-FKC-HGHPCPN----IIDCFVSRPTEKT 209
Macaca mulatta CXNM             156 -TYVIHIFTRSVVEV-GFMIGQYLLY---GFHLEPL-FKC-HGHPCPN----IIDCFVSRPTEKT 209
Callithrix jacchus CXNM         155 -TYVIHIFTRSVVEV-GFMIGQYLLY---GFHLEPL-FKC-HGHPCPN----IIDCFVSRPTEKT 208
Tarsius syrichta CXNM           156 -TYVIHILTRSVLEV-GFMIGQYLLY---GFHLEPL-FKC-HGHPCPN----IIDCFVSRPTEKT 209
Microcebus murinus CXNM         156 -TYVIHIFIRSVVEV-GFMIGQYLLY---GFHLEPL-FKC-HGHPCPN----IIDCFVSRPTEKT 209
Dipodomys ordii CXNM            157 -TYVIHIFTRSVFEV-GFMTGQYFLY---GFHLDPL-FKC-HGHPCPN----IIDCFISRPTEKT 210
Oryctolagus cuniculus CXNM      154 -TYVIHVLTRSVLEV-SFMIGQYLLY---GFHLQPL-FKC-HRQPCPN----IIDCFVSRPTEKT 207
Equus caballus CXNM             156 -TYVIHIFTRSVVEV-GFMIGQYLLY---GFHLKPL-FKC-HGHPCPN----IVDCFVSRPTEKT 209
Canis lupus familiaris CXNM     156 -TYVVHIFTRSMVEV-GFMIGQYLLY---GFHLQPL-FKC-HGHPCPN----VIDCFVSRPTEKT 209
Pteropus vampyrus CXNM          155 -TYVIHIFARSVVEV-GFMTGQYLLY---GFHLEPL-FKC-HGHPCPN----IIDCFVSRPTEKT 208
Loxodonta africana CXNM         156 -TYVIHIFTRSAVEV-GFMIGQYFLY---GFYLEPL-FKC-HSHPCPN----TIDCFISRPTEKT 209
```

```
Homo sapiens CXNN              155 RTYVLHILTRSVLEV-GFMIGQYILY---GFQMHPL-YKC-TQPPCPN----AVDCFVSRPTEKT 209
Pan troglodytes CXNN           155 RTYVLHILTRSVLEV-GFMIGQYILY---GFQMHPL-YKC-TQPPCPN----AVDCFVSRPTEKT 209
Pongo abelii CXNN              155 RTYVLHILTRSVLEV-GFMIGQYILY---GFQMHPL-YKC-TQPPCPN----AVDCFVSRPTEKT 209
Nomascus leucogenys CXNN       155 RTYVLHILTRSVLEV-GFMIGQYILY---GFQMHPL-YKC-TQPPCPN----AVDCFVSRPTEKT 209
Macaca mulatta CXNN            155 RTYVLHILTRSVLEV-GFMIGQYILY---GFQMHPL-YKC-TQPPCPN----AVDCFVSRPTEKT 209
Callithrix jacchus CXNN        155 RTYVLHILTRSVLEV-GFMIGQYILY---GFQMHPL-YKC-TQPPCPN----AVDCFVSRPTEKT 209
Microcebus murinus CXNN        155 RTYVLHILTRSVLEV-GFMIGQYILY---GFQMHPL-YKC-TQPPCPN----TVDCFVSRPTEKT 209
Mus musculus Cxnn              155 RTYVLHILTRSVLEV-GFMIGQYILY---GFQMHPI-YKC-TQAPCPN----SVDCFVSRPTEKT 209
Rattus norvegicus Cxnn         155 RTYVLHILTRSVLEV-GFMIGQYILY---GFQMHPI-YKC-TQAPCPN----AVDCFVSRPTEKT 209
Oryctolagus cuniculus CXNN     185 RTYVLHILTRSVLEV-GFMIGQYVLY---GFQMYPL-YKC-TQPPCPN----AVDCFVSRPTEKT 239
Bos taurus CXNN                155 RTYVLHVLTRSVLEV-GFMVGQYILY---GFQMHPL-YKC-TQPPCPN----AVDCFVSRPTEKT 209
Equus caballus CXNN            155 RTYVLHILTRSVLEV-GFIIGQYILY---GFQMHPL-YKC-TQPPCPN----AVDCFVSRPTEKT 209
Canis lupus familiaris CXNN    155 RTYVLHILTRSVMEV-GFMIGQFILY---GFQMHPL-YKC-TQPPCPN----AVDCFVSRPTEKT 209
Sorex araneus CXNN             155 RTYVLHVLTRSVLEV-GFMTGQYILY---GFQMNPL-YKC-TQPPCPN----VVDCFVSRPTEKT 209
Loxodonta africana CXNN        155 RTYILHILTRSMLEI-GFMIGQYILY---GFQMHPL-YKC-TQSPCPN----TVDCFVSRPTEKT 209
Homo sapiens CXNO              215 ----AQLVARAAFEV-AFLVGQYLLY---GFEVRPF-FPC-SRQPCPH----VVDCFVSRPTEKT 265
Papio hamadryas CXNO           215 ----AQLVARAAFEV-AFLVGQYLLY---GFEVRPF-FPC-SRQPCPH----VVDCFVSRPTEKT 265
Mus musculus Cxno              225 ----AQLVVRAAFEV-AFLVGQYLLY---GFEVPPF-FAC-SRQPCPH----VVDCFVSRPTEKT 275
Cavia porcellus CXNO           209 ----AQLAARAALEV-AFLLGQYLLY---GFAVRPS-FAC-GRWPCPH----VVDCFVSRPTEKT 259
Homo sapiens CXNP1             139 ----AQLGARLVLEG-AALGLQYHLY---GFQMPSS-FAC-RREPCLG----SITCNLSRPSEKT 189
Pan troglodytes CXNP1          139 ----AQLGARLVLEG-AALGLQYHLY---GFQMPSS-FAC-RREPCLG----SITCNLSRPSEKT 189
Pongo abelii CXNP1             139 ----AQLGARLVLEG-AALGLQYHLY---GFQMPSS-FAC-RREPCLG----SITCNLSRPSEKT 189
Callithrix jacchus CXNP1       140 ----AQLGARLVLEG-TALGLQYHLY---GFQTPSS-FAC-RREPCLG----SITCNLSRPSEKT 190
Otolemur garnettii CXNP1       140 ----AQLGARLALEG-AALGGQYHLY---GFQVPSS-FAC-RREPCLG----SITCNLSRPSEKT 190
Tupaia belangeri CXNP1         157 ----AQLGARLVLEG-AALGLQYHLY---GFQVPSS-FAC-RREPCLG----SITCHLSRPSEKT 207
Mus musculus Cxnp1             140 ----AHLGVRLALEG-AALGVQYNLY---GFKMSST-FIC-REDPCIG----STTCFQSHPSEKT 190
Rattus norvegicus Cxnp1        139 ----AHLGVRLVLEG-AALGVQYHLY---GFKMPST-FIC-REDPCIG----STTCFQSHPSEKT 189
Cavia porcellus CXNP1          140 ----VQLGARVVLEG-AALGGQYHLY---GFKVQSS-FSC-RRDPCLG----SVNCNLSRPSEKT 190
Oryctolagus cuniculus CXNP1    140 ----AQLGVRLILEG-VTLGVQYHLY---GFQVPRV-SQC-RTDPCPN----LVECIHSRPSEKT 190
Oryctolagus cuniculus CXNP2    140 ----AQLGTRLVLEG-AALGGQYHLY---GFKMPSS-FAC-RREPCLG----SITCNLSRPSEKT 190
Ochotona princeps CXNP1        140 ----AQLGARLALEG-AALGGQFHLY---GFQMPGS-FAC-RREPCLG----SITCTLSRPSEKT 190
Bos taurus CXNP1               141 ----AQLGVRLALEG-AALGGQYHLY---GFRMPSS-FVC-RLEPCLG----STNCYLSRPSEKS 191
Equus caballus CXNP1           140 ----AQLGVRLVLEG-AALGGQYHLY---GFKMPSS-FAC-RREPCLG----SITCTVSRPSEKT 190
Canis lupus familiaris CXNP1   138 ----AQLGVRLVLEG-AALGLQYHLY---GFKIPSS-FAC-RREPCLG----SITCYLSRPFEKT 188
Felis catus CXNP1              161 ----AQLGVRLVLEG-TALGVQYHVY---GFNVPSS-FAC-RREPCLG----SITCYLSRPFEKT 211
Myotis lucifugus CXNP1         140 ----AQLGVRLVLEG-AALGGQYHLY---GFKMPSS-FSC-RREPCLG----SITCHVPRPSEKT 190
Dasypus novemcinctus CXNP1     139 ----AQLGARVVLEG-ATLAGQYHLY---GFHVPLL-FRC-SRFPCPH----MVDCVLPRPSEKS 189
Dasypus novemcinctus CXNP2     139 ----AQLGVRVVLEG-ATLAGQYHLY---GFHVPLI-FRC-SRFPCPH----MVDCVLPRPSEKS 189
Dasypus novemcinctus CXNP3     139 ----AQLGARVVLEG-ATLAGQYHLY---GVHMPSS-SSC-RKEPCFG----SITCVSSRASEQS 189
Dasypus novemcinctus CXNP4     139 ----AQLGVRVVLEG-ATLAGQYHLY---GFHVPLL-FRC-SRFPCPH----MVDCVLPRPSEKS 189
Choloepus hoffmanni CXNP1      138 ----AQLGARLVLEG-ATPGVQYHLY---GVHMPLV-FEC-HAIPCPY----SVTCSPSCPSEKS 188
Loxodonta africana CXNP1       140 ----AHMGV-------------------------------------------------------- 144
Homo sapiens CXNQ              179 ----LQLLARTVFEV-GFLIGQYFLY---GFQVHPF-YVC-SRLPCPH----KIDCFISRPTEKT 229
Pan troglodytes CXNQ           179 ----LQLLARTVFEV-GFLIGQYFLY---GFQVHPF-YVC-SRLPCPH----KIDCFISRPTEKT 229
Pongo abelii CXNQ              179 ----LQLLARTVFEV-GFLIGQYFLY---GFQVHPF-YVC-SRLPCPH----KIDCFISRPTEKT 229
Macaca mulatta CXNQ            179 ----LQLLARTVFEV-GFLIGQYFLY---GFQVHPF-YVC-SRLPCPH----KIDCFISRPTEKT 229
Tupaia belangeri CXNQ          179 ----LQLLARTVFEV-GFLIGQYFLY---GFQVHPS-YVC-SRHPCPH----KIDCFISRPTEKT 229
Mus musculus Cxnq              179 ----LQLLARTVFEV-GFLIGQYFLY---GFQVHPF-YVC-SRLPCPH----KIDCFISRPTEKT 229
Rattus norvegicus Cxnq         179 ----LQLLARTVFEV-GFLIGQYFLY---GFQVHPF-YVC-SRLPCPH----KIDCFISRPTEKT 229
Cavia porcellus CXNQ           179 ----LQLLARTAFEV-GFLIGQYFLY---GFQVHPF-YVC-SRLPCPH----KIDCFISRPTEKT 229
S.tridecemlineatus CXNQ        193 ----LQLLARTVFEV-GFLIGQYFLY---GFQVHPF-YVC-SRLPCPH----KIDCFISRPTEKT 243
Oryctolagus cuniculus CXNQ     179 ----LQLLARTVFEV-GFLVGQYFLY---GFQVHPF-YVC-SRLPCPH----KIDCFISRPTEKT 229
Bos taurus CXNQ                179 ----LQLLARTVFEV-GFLVGQYFLY---GFQVHPF-YVC-SRLPCPH----KIDCFISRPTEKT 229
Vicugna pacos CXNQ             179 ----LQLLARTVFEV-GFLIGQYFLY---GFQVHPF-YVC-SRLPCPH----KIDCFISRPTEKT 229
Equus caballus CXNQ            179 ----LQLLARTVFEV-GFLIGQYFLY---GFQVHPF-YVC-SRIPCPH----KIDCFISRPTEKT 229
Canis lupus familiaris CXNQ    179 ----LQLLARTVFEV-GFLIGQYFLY---GFQVHPF-YVC-SRLPCPH----KIDCFISRPTEKT 229
Myotis lucifugus CXNQ          179 ----LQLLARTVFEV-GFLIGQYFLY---GFQVHPS-YVC-SRIPCPH----KIDCFISRPTEKT 229
Pteropus vampyrus CXNQ         179 ----LQLLARTMFEV-GFLIGQYFLY---GFQVHPS-YVC-SRIPCPH----KIDCFISRPTEKT 229
Loxodonta africana CXNQ        179 ----LQLLARTVFEV-GFLIGQYFLY---GFQVHPF-YVC-SRLPCPH----KIDCFISRPTEKT 229
Homo sapiens CXNR              138 ----LSVALRLLAEL-TFLGGQALLY---GFRVAPH-FAC-AGPPCPH----TVDCFVSRPTEKT 188
Pan troglodytes CXNR           138 ----LSVALRLLAEL-TFLGGQALLY---GFRVAPH-FAC-AGPPCPH----TVDCFVSRPTEKT 188
Pongo abelii CXNR              138 ----LSVALRLLAEL-TFLGGQALLY---GFRVAPH-FAC-AGPPCPH----TVDCFVSRPTEKT 188
Papio hamadryas CXNR           138 ----LSVALRLLAEL-TFLGGQALLY---GFRVVPH-FAC-AGPPCPH----TVDCFVSRPTEKT 188
Mus musculus Cxnr              138 ----LSVALRLLAEL-AFLGGQALLY---GFRVDPH-YAC-AGPPCPH----TVDCFVSRPTEKT 188
Rattus norvegicus Cxnr         138 ----LSVALRLLAEL-AFLGGQALLY---GFRVAPH-YAC-AGPPCPH----TVDCFVSRPTEKT 188
Oryctolagus cuniculus CXNR     146 ----LSVALRLLAEL-AFLGGQARLY---GFRVAPH-FAC-AGPPCPH----TVDCFVSRPTEKT 196
Bos taurus CXNR                128 ----LSVALRLLAEL-TFLAGQALLY---GFRVAPR-FVC-AGPPCPH----TVDCFVSRPTEKT 178
Homo sapiens CXNS              150 ----IQVVFRNALEI-GFLVGQYFLY---GFSVPGL-YEC-NRYPCIK----EVECYVSRPTEKT 200
Pan troglodytes CXNS           150 ----IQVVFRNALEI-GFLVGQYFLY---GFSVPGL-YEC-NRYPCIK----EVECYVSRPTEKT 200
Pongo abelii CXNS              150 ----IQVVFRNALEI-GFLVGQYFLY---GFSVPGL-YEC-NRYPCIK----EVECYVSRPTEKT 200
Nomascus leucogenys CXNS       150 ----IQVVFRNALEI-GFLVGQYFLY---GFSVPGL-YEC-NRYPCIK----EVECYVSRPTEKT 200
Macaca mulatta CXNS            150 ----IQVVFRNALEI-GFLVGQYFLY---GFSVPGL-YEC-NRYPCIK----EVECYVSRPTEKT 200
Papio hamadryas CXNS           150 ----IQVVFRNALEI-GFLVGQYFLY---GFSVPGL-YEC-NRYPCIK----EVECYVSRPTEKT 200
Otolemur garnettii CXNS        150 ----IQVVFRNALEI-GFLVGQYFLY---GFSVPGL-YEC-NRYPCIK----EVECYVSRPTEKT 200
Tupaia belangeri CXNS          150 ----IQVVFRNALEI-GFLVGQYFLY---GFSVPGL-YEC-NRYPCIK----EVECYVSRPTEKT 200
Mus musculus Cxns              190 ----IQVVFRNALEI-GFLVGQYFLY---GFSVPGL-YEC-NRYPCIK----EVECYVSRPTEKT 240
Rattus norvegicus Cxns         150 ----IQVVFRNALEI-GFLVGQYFLY---GFSVPGL-YEC-NRYPCIK----EVECYVSRPTEKT 200
Cavia porcellus CXNS           150 ----IQVVFRNALEI-GFLVGQYFLY---GFSVPGL-YEC-NRYPCIK----EVECYVSRPTEKT 200
S.tridecemlineatus CXNS        150 ----IQVVFRNALEI-GFLVGQYFLY---GFSVPGL-YEC-NRYPCIK----EVECYVSRPTEKT 200
```

```
Oryctolagus cuniculus CXNS    150 ----IQVVFRNALEI-GFLVGQYFLY---GFSVPGL-YEC-NRYPCIK----EVECYVSRPTEKT 200
Ochotona princeps CXNS        150 ----IQVVFRNALEI-GFLVGQYFLY---GFSVPGL-YEC-NRYPCIK----EVECYVSRPTEKT 200
Bos taurus CXNS               150 ----IQVVFRNALEI-GFLVGQYFLY---GFSVPGL-YEC-DRYPCIK----EVECYVSRPTEKT 200
Equus caballus CXNS           150 ----IQVVFRNALEI-GFLVGQYFLY---GFSVPGL-YEC-DRYPCIK----EVECYVSRPTEKT 200
Canis lupus familiaris CXNS   150 ----IQVVFRNALEI-GFLVGQYFLY---GFSVPGL-YEC-DRYPCIK----EVECYVSRPTEKT 200
Myotis lucifugus CXNS         150 ----IQVVFRNALEI-GFLVGQYFLY---GFSVPGL-YEC-DRYPCIK----EVECYVSRPTEKT 200
Dasypus novemcinctus CXNS     150 ----IQVVFRNALEI-GFLVGQYFLY---GFSVPGL-YEC-DRYPCIK----EVECYVSRPTEKT 200
Loxodonta africana CXNS       278 ----IQVVFRNALEI-GFLVGQYFLY---GFSVPGL-YEC-DRYPCIK----EVECYVSRPTEKT 328
Homo sapiens CXNT             118 ----LSVLLRISLAAIAFW-LQIYLF---GFQV-KSLYLCDARS--LGE-NMIIRCMVPEHFEKT 170
Pan troglodytes CXNT          118 ----LSVLLRISLAAIAFW-LQIYLF---GFQV-KSLYLCDARS--LGE-NMNIRCMVPEHFEKT 170
Nomascus leucogenys CXNT      121 ----LSILLRISLEAIAFW-LQIYLF---GFQV-KSLYLCDARS--L-RKNMIIRCMVPEHFEKM 173
Mus musculus Cxnt             121 ----LSVLLRISLEVFAFW-LQIHLF---GFQV-KPIYLCDTES--LGKKPNILKCMVPEHFEKT 174
Rattus norvegicus Cxnt        121 ----LSALLRISLEVSAFW-LQIHLF---GFQV-KPVYLCDAES--LGQKINTLKCMVPEHFEKT 174
Cavia porcellus CXNT          117 ----LSVLLRISLEVIAFW-LQIHLF---GFQV-KALYLCDATS--LGKKMTIIRCMVPEHFEKT 170
Oryctolagus cuniculus CXNT    121 ----LSVLLRISLEVIAFW-LQIHLF---GFQV-KPLFLCDAGS--LGGKLTIIRCMVPEHFEKT 174
Bos taurus CXNT               121 ----LSVLLRISLEVVAFW-LQIHLF---GFQV-KPLYLCDAVS--LGKKLTIIKCMVPEHFEKT 174
Vicugna pacos CXNT            121 ----LSVLLRISLEVVAFW-LQIHLF---GFQV-KPLYLCDAVS--LGKKFTIIKCMVPEHFEKT 174
Equus caballus CXNT           121 ----LSVLLRISLEVIAFW-LQTRLF---GFQV-KPLYLCDAGS--LGGKFTIVQCMVPEHLEKT 174
Canis lupus familiaris CXNT   121 ----LSVLLRISLEVIAFW-LQIHLF---GFQV-KALYLCDAGS--LGKKFTIIKCMVPEHFEKT 174
Myotis lucifugus CXNT         121 ----VSVLVRISLEVISFW-LQIHLF---GFQV-TPLYLCDAGS--LGGKFTIIKCMVPDHFEKT 174
Dasypus novemcinctus CXNT     121 ----LSILLRISLEVIAFW-LQIHLF---GFQV-KPLFLCDARS--LGEKNNIIKCMVPEHFEKT 174
Loxodonta africana CXNT       117 ----LSVLLRISLEVIAFW-LQIRLF---GFQV-KSLYLCDARS--LKGRNNIITCMVPEHFEKT 170
Homo sapiens CXNU             131 ----IHLLLRTLLEA-AFGALHYFLF---GFLAPKK-FPC-TRPPCTG----VVDCYVSRPTEKS 181
Pan troglodytes CXNU          131 ----IHLLLRTLLEA-AFGALHYFLF---GFLAPKK-FPC-TRPPCTG----VVDCYVSRPTEKS 181
Pongo abelii CXNU             116 ----IHLLLRTLLEA-AFGALHYFLF---GFLAPKK-FPC-TRPPCTG----VVDCYVSRPTEKS 166
Nomascus leucogenys CXNU      128 ----IHLLLRTLLEA-AFGALHYFLF---GFLAPKK-FPC-TRPPCTG----VVDCYVSRPTEKS 178
Macaca mulatta CXNU           131 ----IHLLLRTLLEA-AFGALNYLLF---GFLAPNK-FPC-TRPPCTG----VVDCYVSRPTEKS 181
Mus musculus Cxnu             101 ----VHLLLRMLLEA-GLAFLHYFLF---GFSVPAR-VSC-SHVPCSG----AVDCYVSRPTEKS 151
Cavia porcellus CXNU           94 ----FQLCFRTLTEA-AFGAWHYFLF---GFLVPKS-FSC-THPPCSS----VVDCYISRPTEKS 144
Bos taurus CXNU               114 ----VHLCLRTLTEA-AFGALHYLLF---GFLVPKR-FSC-THPPCTS----VVDCYVSRPTEKS 164
Equus caballus CXNU           122 ----VHLFLRTLTEA-AFGALHYLLF---GFLVPKR-FSC-TRPPCTS----VVDCYVSRPTEKS 172
Myotis lucifugus CXNU         138 ----VHLCLRTLAET-ALGALHYLLF---GFSVPNR-FSC-AHSPCSG----SVDCYVSRPTEKS 188
Sorex araneus CXNU            110 ----AHLLLRTLLEA-AFGALHYLLF---GFVVPKR-FSC-THPPCSS----VVDCYVSRPTEKS 160
Dasypus novemcinctus CXNU     140 ----VHLFLRTLIEA-TFGALHYLLF---GFLVPKR-FSC-SHAPCTS----VVDCYISRPTEKS 190
Loxodonta africana CXNU       149 ----AHLFLRTLIEA-AFGALHYFLF---GFMVPKR-FSC-SHSPCTS----VVDCYISRPTEKS 199
```

```
Homo sapiens CXNA               185 IFTLFMVATA-AIC-ILLNL---VEL--IYLVS-KRC-H-EC-----L--AA------------R 220
                                            530       540       550       560       570       580

Homo sapiens CXNA               185 IFTLFMVATA-AIC-ILLNL---VEL--IYLVS-KRC-H-EC-----L--AA------------R 220
Pan troglodytes CXNA            185 VFTLFMVATA-AIC-ILLNL---VEL--IYLVS-KRC-H-EC-----L--AA------------R 220
Gorilla gorilla CXNA            185 IFTLFMVATA-AIC-ILLNL---VEL--IYLVS-KRC-H-EC-----L--AA------------R 220
Pongo abelii CXNA               185 IFTLFMVATA-AVC-ILLNL---VEL--IYLLN-KRC-H-EC-----L--AA------------R 220
Nomascus leucogenys CXNA        185 IFTLFMVATA-AVC-ILLNL---VEL--IYLVS-KRC-H-EY-----L--AA------------R 220
Macaca mulatta CXNA             185 IFTLFMVATA-AIC-ILLNL---VEL--IYLVS-KRC-H-EC-----L--AA------------R 220
Callithrix jacchus CXNA         185 IFTFFMVATA-AIC-ILLNL---MEL--IYLVS-KRC-H-EC-----L--EA------------R 220
Mus musculus Cxna               183 IFIVFMVVTA-VIC-ILLNL---VEL--IYLVI-KRC-S-EC-----A--QL------------R 218
Rattus norvegicus Cxna          183 IFIVFMLVTA-IVC-ILLNL---VEL--LYLVI-KRC-S-EC-----A--PA------------K 218
Cavia porcellus CXNA            235 IFTLFMVITA-VIC-ILLNL---VEL--SYLVS-KRC-R-EY-----L--AA------------R 270
Ochotona princeps CXNA          185 IFTLFMVITA-IIC-ILLNL---VEL--TYLVG-KRC-R-EC-----L--GA------------R 220
Bos taurus CXNA                 185 IFTLFMVITA-LVC-IVLNL---VEL--AYLVS-KRC-R-EC-----L--EA------------R 220
Equus caballus CXNA             185 IFTLFMIITA-AIC-ILLNL---VEL--AYLVS-KRC-R-EC-----L--AA------------R 220
Canis lupus familiaris CXNA     185 IFTLFMVTTA-VVC-ILLNL---VEL--AYLVG-KRC-R-EG-----L--AP------------R 220
Felis catus CXNA                185 IFTLFMVVTA-AVC-ILLNL---VEL--AYLVS-KRC-R-EG-----L--AA------------R 220
Myotis lucifugus CXNA           217 IFTLFMVATA-AVC-ILLNL---VEL--AYLVS-KRC-R-EC-----L--AA------------R 252
Dasypus novemcinctus CXNA       185 IFTLFMVITA-AIC-ILLNL---LEL--AYLVS-KRC-R-ES-----L--AA------------R 220
Loxodonta africana CXNA         185 IFTLFMVVTA-AIC-ILLNL---VEL--AYLVS-KRC-C-EC-----L--AA------------R 220
Homo sapiens CXNB               185 VFTYFMVTTA-AIC-ILLNL---SEV--FYLVG-KRC-M-E-----IF--GP------------R 220
Gorilla gorilla CXNB            185 VFTYFMVTTA-AIC-ILLNL---SEV--FYLVG-KRC-M-E-----IF--GS------------R 220
Nomascus leucogenys CXNB        185 VFTYFMVTTA-AIC-ILLNL---SEV--FYLVG-KRC-L-E-----IF--GP------------R 220
Macaca mulatta CXNB             185 VFTYFMVTTA-AIC-ILLNL---SEV--FYLVG-KRC-T-E-----IF--SP------------R 220
Callithrix jacchus CXNB         185 VFTYFMVTTA-AIC-ILLNL---SEV--FYLVG-KRC-M-E-----IF--GP------------R 220
Otolemur garnettii CXNB         185 VFTYFMVTTA-AIC-ILLNL---CEV--FYLVG-KRC-M-E-----LL--GP------------R 220
Mus musculus Cxnb               185 VFTYFMVVTA-AIC-ILLNL---SEV--VYLVG-KRC-M-E-----VF--RP------------R 220
Rattus norvegicus Cxnb          185 VFTYFMVVTA-AIC-ILLNL---SEV--AYLVG-KRC-M-E-----VF--RP------------R 220
Dipodomys ordii CXNB            185 VFTYFMVATA-VIC-ILLNL---SEV--TYLVG-KRC-M-E-----LL--GP------------R 220
Cavia porcellus CXNB            185 VFTCFMVGTA-VVC-ILLNL---CEV--TYLVG-KRC-R-E-----SL--GP------------R 220
Oryctolagus cuniculus CXNB       93 VFTYFMVTTA-VIC-ILLNL---SEV--TYLVG-KRC-L-E-----IF--GT------------K 128
Ochotona princeps CXNB          185 VFTYFMVATA-AVC-ILLNL---SEV--AYLVG-KRC-L-E-----IF--SP------------K 220
Bos taurus CXNB                 185 IFTYFMVATA-VLC-ILLNL---GEV--SYLVG-KRC-L-E-----IL--GP------------R 220
Equus caballus CXNB             185 VFTYFMVATA-VIC-ILLNL---SEV--TYLVG-KRC-L-E-----TL--GP------------R 220
Canis lupus familiaris CXNB     235 VFTYFMVATA-VIC-ILLNL---SEV--TYLVG-KRC-L-E-----TF--HP------------R 270
Myotis lucifugus CXNB           186 VFTYFMVATA-VIC-ILLNL---SEV--AYLVG-KRC-L-E-----IV--GP------------R 221
Erinaceus europaeus CXNB        185 VFTYFMVATS-VIC-ILLNL---CEV--SYLVG-KRC-L-R-----TL--GP------------R 220
Loxodonta africana CXNB         185 VFTYFMVATA-VIC-ILLNL---SEV--AYLVG-KRC-M-E-----IL--GP------------R 220
Homo sapiens CXNC               186 IFTYFMVGAS-AVC-IVLTIC---EL-C-YLIC-HR--V-L----RGL--HK------------D 221
Gorilla gorilla CXNC            186 IFTYFMVGAS-AVC-IVLTIC---EL-C-YLIC-HR--V-L----RGL--HK------------D 221
Pongo abelii CXNC               186 IFTYFMVGAS-AVC-IVLTIC---EL-C-YLIC-HR--V-L----RGL--HK------------D 221
Nomascus leucogenys CXNC        186 IFTYFMVGAS-AVC-IVLTIC---EL-C-YLIC-HR--V-L----RGL--HK------------D 221
Callithrix jacchus CXNC         186 IFTYFMVGAS-AVC-IVLTIC---EL-C-YLIC-HR--V-L----RAL--RK------------D 221
Microcebus murinus CXNC         216 VFTYFMVGAS-AVC-IVLTIC---EI-C-YLIF-HR--V-L----RCL--QK------------D 251
Otolemur garnettii CXNC         186 VFTYFMVAAS-AVC-IVLTIC---EI-C-YLIF-HR--L-L----RCM--HR------------D 221
Mus musculus Cxnc               186 VFTYFMVGAS-AVC-IILTIC---EI-C-YLIF-HR--I-M----RGI--SK------------G 221
Rattus norvegicus Cxnc          186 VFTYFMVGAS-AVC-IILTIC---EI-C-YLIF-HR--I-M----RGL--SK------------D 221
Cavia porcellus CXNC            186 IFTYFMVGAS-AVC-IVLTIC---EI-C-YLIF-HR--I-M----RGL--GK------------G 221
Oryctolagus cuniculus CXNC      296 VFTYFMVGAS-AVC-IVLTIC---EI-C-YLIF-HR--L-L----KVL--QR------------N 331
Bos taurus CXNC                 186 LFTYFMVGAS-AVC-IVLTFC---EI-C-YLIF-HR--V-V----RSL--HR------------K 221
Equus caballus CXNC             186 VLTYFMVGAS-AVC-IVLTIC---EI-C-YLIF-HR--I-L----RGR--SK------------N 221
Canis lupus familiaris CXNC     186 VFTYFMVGAS-AVC-IVLTIC---EI-C-YLIF-HR--L-L----RRV--TR------------N 221
Felis catus CXNC                186 VFTYFMVGAS-AVC-IVLTIC---EI-C-YLIF-HR--V-L----RGI--AR------------N 221
Myotis lucifugus CXNC           186 VFTYFMVGAS-AVC-IVLTIS---EI-C-YLIF-HR--V-I----KGI--RN------------Q 221
Dasypus novemcinctus CXNC       186 IFTYFMVGAS-AIC-IVLTIC---EI-C-YLIF-HR--L-M----RGMRRHK------------D 223
Loxodonta africana CXNC         186 VFTYFMVGAS-AVC-IVLTVC---EI-C-YLIV-HR--V-F----RAL--RK------------D 221
Homo sapiens CXND               178 IFILFLVITSC-LC-IVLNF---IEL--SFLVL-K-CLI-KCC----L--QK------------Y 214
Pan troglodytes CXND            178 IFILFLVITSC-LC-IVLNF---IEL--SFLVL-K-CFI-KCC----L--QK------------Y 214
Macaca mulatta CXND             178 IFILFLVITSC-LC-IVLNF---IEL--SFLVL-K-CFI-KCC----L--QK------------Y 214
Callithrix jacchus CXND         178 IFVLLLVITSC-LC-IALNF---TEL--SFLVF-K-CFI-KCC----L--QK------------H 214
Dipodomys ordii CXND            178 IFIFFLVIASG-LC-IVLNF---AEL--CFLVL-K-YFF-KCS----L--QK------------H 214
Oryctolagus cuniculus CXND      178 IFILFLIITSC-LC-MVLNF---IEL--SFLVL-K-CFI-KCC----L--KR------------D 214
Tursiops truncatus CXND         178 IFTLFLVITSC-LC-VVLNF---TEL--SFLVL-K-CFV-KCC----L--QK------------Y 214
Bos taurus CXND                  96 IFILFLVVTSC-LC-IVLNV---TEL--SFLVL-K-CFI-KCC----L--QK------------Y 132
Equus caballus CXND             197 IFILFLVSTSC-LC-VILNF---IEL--SFLVL-K-CLI-KCC----L--QK------------Y 233
Canis lupus familiaris CXND     178 IFILFLVITSC-LS-IVLNF---IEL--SFLVL-K-CLI-MCC----L--QK------------Y 214
Myotis lucifugus CXND           178 IFILFMVVTSC-LC-IVLNF---TEL--SFLIL-K-CLL-KCC----L--QK------------Y 214
Erinaceus europaeus CXND        178 IFILFLVITSC-LC-IVLNL---VEL--SFLVL-K-CFI-KFC----L--QK------------Y 214
Dasypus novemcinctus CXND       178 IFILFLVITSG-LC-IVLNF---TEL--SLLVL-K-CFI-KCC----L--QQ------------Y 214
Choloepus hoffmanni CXND        178 IFILFLVITSC-LC-IVLNV---IEL--NFLVL-K-CFI-KCC----L--QQ------------Y 214
Loxodonta africana CXND         178 IFILFLVITSC-LC-IVLNF---IEL--SFLVL-K-CFI-KYH----L--QQ------------Y 214
Homo sapiens CXNE               190 VFTVFMIAVS-GIC-ILLNV---TEL-C-YLLI-RYCSG-K---------SK------------K 224
Pan troglodytes CXNE            190 VFTVFMIAVS-GIC-ILLNV---TEL-C-YLLI-RYCSG-K---------SK------------K 224
Pongo abelii CXNE               190 VFTVFMIAVS-GIC-ILLNV---TEL-C-YLLI-RYCSG-R---------SK------------K 224
Macaca mulatta CXNE             190 VFTVFMIAVS-GIC-ILLNV---TEL-C-YLLI-RYCSG-K---------SK------------K 224
Papio hamadryas CXNE            190 VFTVFMIAVS-GIC-ILLNV---TEL-C-YLLI-RYCSG-K---------SK------------K 224
Callithrix jacchus CXNE         190 VFTVFMIAVS-GIC-ILLNV---TEL-C-YLLI-RYCSG-K---------SK------------K 224
Otolemur garnettii CXNE         190 VFTVFMIAVS-GIC-ILLNV---TEL-C-YLLI-RYCSG-K---------SR------------K 224
Mus musculus Cxne               203 VFTVFMISVS-GIC-ILLNI---TEL-C-YLFV-RYCSG-K---------SK------------R 237
```

```
Rattus norvegicus Cxne          209 VFTVFMISVS-GIC-ILLNI---TEL-C-YLFI-RYCSG-K---------SK------------R 243
Dipodomys ordii CXNE            209 VFTIFMISVS-GIC-ILLNI---TEL-C-YLFI-RYCSG-K---------SR------------R 243
Cavia porcellus CXNE            237 VFTVFMIVVS-GIC-ILLNI---TEL-C-YLFI-RYCSK-K---------SK------------K 271
Oryctolagus cuniculus CXNE      209 VFTVFMIAVS-GIC-ILLNV---TEL-C-YLLI-RYCSG-K---------SK------------K 243
Equus caballus CXNE             190 VFTVFMIAVS-GIC-ILLNV---TEL-C-YLLI-RYCSG-K---------SK------------K 224
Canis lupus familiaris CXNE     190 VFTVFMIAVS-GIC-ILLNV---TEL-C-YLLI-RYCSG-K---------SK------------K 224
Felis catus CXNE                190 VFTVFMIAVS-GIC-ILLNV---TEL-C-YLLI-RYCSG-K---------SK------------K 224
Myotis lucifugus CXNE           190 VFTVFMIAVS-GIC-ILLNV---TEL-C-YLLI-RYCSG-K---------SK------------K 224
Sorex araneus CXNE              190 VFTFFMIAVS-GIC-IMLNV---TEL-C-YLLI-RYCSG-K---------SK------------K 224
Dasypus novemcinctus CXNE       190 VFTVFMIAVS-GIC-ILLNV---TEL-C-YLLI-RYCSG-K---------SR------------K 224
Loxodonta africana CXNE         190 VFTVFMIAVS-GIC-ILLNV---TEL-C-YLLI-RYCSG-K---------SK------------K 224
Homo sapiens CXNF               190 VFTIFMISAS-VIC-MLLNV---AEL-C-YLLL-KVCFR-R---------SK------------R 224
Pan troglodytes CXNF            190 VFTIFMISAS-VIC-MLLNV---AEL-C-YLLL-KVCFR-R---------SK------------R 224
Gorilla gorilla CXNF            190 VFTIFMISAS-VIC-MLLNV---AEL-C-YLLL-KVCFR-R---------SK------------R 224
Nomascus leucogenys CXNF        190 VFTIFMISAS-VIC-MLLNV---AEL-C-YLLL-KVCFR-R---------SK------------R 224
Callithrix jacchus CXNF         190 VFTIFMISAS-VIC-MLLNV---AEL-C-YLLL-KVCFR-R---------SK------------R 224
Mus musculus Cxnf               190 VFTVFMISAS-VIC-MLLNV---AEL-C-YLLL-KLCFR-R---------SK------------R 224
Rattus norvegicus Cxnf          190 VFTVFMISAS-VIC-MLLNV---AEL-C-YLLL-KLCFR-R---------SK------------R 224
S.tridecemlineatus CXNF         190 VFTIFMISAS-VIC-MLLNV---AEL-C-YLLL-KVCFR-R---------SK------------R 224
Oryctolagus cuniculus CXNF      190 VFTIFMISAS-VIC-MLLNV---AEL-C-YLLL-KVCFR-R---------SK------------R 224
Ochotona princeps CXNF          190 VFTVFMISAS-VVC-MLLNV---AEL-C-YLLL-KVCVR-S---------SK------------R 224
Bos taurus CXNF                 190 VFTIFMISAS-VIC-MLLNV---AEL-C-YLLL-KVCFR-R---------SK------------R 224
Equus caballus CXNF             190 VFTVFMISAS-VIC-MLLNV---AEL-C-YLLL-KVCFR-R---------SK------------R 224
Canis lupus familiaris CXNF     190 VFTIFMISAS-VIC-MLLNV---AEL-C-YLLL-KVCFR-R---------SK------------R 224
Myotis lucifugus CXNF           190 VFTIFMISAS-VIC-MLLNV---AEL-C-YLLL-KVCFR-K---------SK------------R 224
Dasypus novemcinctus CXNF       190 VFTVFMIAAS-VIC-MLLNV---AEL-C-YLLL-KVCFR-R---------SK------------R 224
Choloepus hoffmanni CXNF        190 VFTIFMISAS-AIC-MLLNV---AEL-C-YLLL-KVCFK-R---------SK------------S 224
Loxodonta africana CXNF         190 VFTVFMISAS-VIC-MLLNV---AEL-C-YLLL-KVCFR-R---------SK------------R 224
Homo sapiens CXNG               189 VFTVFMLAAS-GIC-IILNV---AEV--VYLII-RACAR-R------A--QR------------R 224
Pan troglodytes CXNG            189 VFTVFMLAAS-GIC-IILNV---AEV--VYLII-RACAR-R------A--QR------------R 224
Gorilla gorilla CXNG            189 VFTVFMLAAS-GIC-IILNV---AEV--VYLII-RACAR-R------A--QR------------R 224
Pongo abelii CXNG               189 VFTVFMLAAS-GIC-IILNV---AEV--VYLII-RACAR-R------A--QR------------R 224
Nomascus leucogenys CXNG        189 VFTVFMLAAS-GIC-IILNV---AEV--VYLII-RACAR-R------A--QR------------R 224
Macaca mulatta CXNG             189 VFTVFMLAAS-GIC-IILNV---AEV--VYLII-RACAR-R------A--QR------------R 224
Papio hamadryas CXNG            189 VFTVFMLAAS-GIC-IILNV---AEV--VYLII-RACAR-R------A--QR------------R 224
Callithrix jacchus CXNG         189 VFTVFMLAAS-GIC-IILNV---AEV--VYLII-RACAR-R------A--QR------------R 224
Microcebus murinus CXNG         189 VFTVFMLAAS-GIC-IILNV---AEV--VYLII-RACAR-R------A--QR------------R 224
Mus musculus Cxng               189 VFTVFMLAAS-GIC-IILNV---AEV--VYLII-RACAR-R------A--QR------------R 224
Rattus norvegicus Cxng          189 VFTVFMLAAS-GIC-IILNV---AEV--VYLII-RACAR-R------A--QR------------R 224
Oryctolagus cuniculus CXNG      189 VFTVFMLAAS-GIC-IILNV---AEV--VYLII-RACAR-R------A--QR------------R 224
Bos taurus CXNG                 189 IFTVFMLAAS-GIC-IILNV---AEV--VYLIF-RACAR-R------A--QR------------R 224
Equus caballus CXNG             189 VFTVFMLAAS-GIC-IILNV---AEV--VYLIV-RACAR-R------A--QR------------R 224
Canis lupus familiaris CXNG     189 VFTVFMLAAS-GIC-IILNV---AEV--VYLII-RACAR-R------A--QR------------R 224
Felis catus CXNG                189 VFTVFMLAAS-GIC-IILNV---AEV--VYLII-RACAR-R------A--QR------------R 224
Myotis lucifugus CXNG           189 VFTVFMLVAS-GIC-IILNV---AEV--VYLII-RACAR-R------A--RR------------H 224
Pteropus vampyrus CXNG          189 VFTVFMLAAS-GIC-IILNV---AEL--VYLII-RACTR-R------A--RR------------R 224
Dasypus novemcinctus CXNG       189 VFTVFMLAAS-GIC-IILNV---AEV--VYLII-RACAR-R------A--QR------------R 224
Loxodonta africana CXNG         189 VFTVFMLAAS-GIC-IILNV---AEV--VYLII-RACAR-R------A--QR------------R 224
Procavia capensis CXNG          189 VFTVFMLAAS-GIC-IILNV---AEV--VYLII-RACAR-R------A--QR------------S 224
Homo sapiens CXNH1              208 IFIIFMLVVG-LI-SLVLNL---LEL--VHL-LCR-CLS-R--GMRARQGQ-------------D 247
Pan troglodytes CXNH1           208 IFIIFMLVVG-LI-SLVLNL---LEL--VHL-LCR-CLS-R--GMRARQGQ-------------D 247
Gorilla gorilla CXNH1           208 IFIIFMLVVG-LI-SLVLNL---LEL--VHL-LCR-CLS-R--GMRARQGQ-------------D 247
Pongo abelii CXNH1              208 IFIIFMLVVG-LI-SLVLNL---LEL--VHL-LCR-CLS-R--GMRAPQGQ-------------D 247
Nomascus leucogenys CXNH1       208 IFIIFMLVVG-LI-SLVLNL---LEL--VHL-LCR-CLS-R--GMRARQGQ-------------D 247
Macaca mulatta CXNH1            208 IFIIFMLVVG-LI-SLVLNL---LEL--VHL-LCR-CLS-Q--GMRARQGQ-------------D 247
Otolemur garnettii CXNH1        204 VFMIFMLLVG-LM-FLVLNI---VDL--VYL-WCV-SISPK--NQTLKKPQ-------------D 244
Mus musculus Cxnh1              208 IFIIFMLVVG-VI-SLVLNL---LEL--VHL-LCR-CVS-R--EIKARRDH-------------D 247
Rattus norvegicus Cxnh1         208 IFIIFMLVVG-VI-SLVLNL---LEL--VHL-LCR-CVS-R--EIKARRDH-------------D 247
Cavia porcellus CXNH1           208 IFIIFMLVVG-FI-SLVLNL---LEL--MHL-VCH-HLS-H--RTKAQQAQ-------------D 247
Oryctolagus cuniculus CXNH1     208 IFIIFMLVVG-LI-SLVLNL---LEL--VYL-LCR-CLS-R--GLRARQGQ-------------G 247
Ochotona princeps CXNH1         222 IFIIFMLVVG-LI-SLVLNL---LEL--VHL-LCR-CLS-R--ELRARQSR-------------G 261
Equus caballus CXNH1            208 VFIIFMLVVG-CI-SLVLNV---LEL--AHL-LCR-SLS-Q--RMKAREGQ-------------D 247
Canis lupus familiaris CXNH1    208 IFIIFMLVVG-LI-SLVLNL---LEL--AHL-LCR-CLS-R--GMKAQRGQ-------------D 247
Felis catus CXNH1               208 IFIIFMLVVG-LI-SLVLNL---LEL--GHL-LCR-CLS-R--GVRARQGQ-------------D 247
Myotis lucifugus CXNH1          240 IFIIFMQVVG-FI-SLLLNL---LEL--VHL-LYR-YLT-R--RLKAQQGQ-------------H 279
Dasypus novemcinctus CXNH1      208 IFIIFMLVVG-LI-SLVLNL---LEL--AYL-LCQ-CLY-R--GVKVRQSQ-------------D 247
Loxodonta africana CXNH1        208 IFIIFMLVVG-LI-SLVLNL---LEL--VHL-LAR-CLS-R--GMRARRGQ-------------E 247
Homo sapiens CXNI               206 VFIVFMLAVA-AL-SLLLSL---AEL--YHLGW-K-----K---IRQRFVKP------------R 242
Pan troglodytes CXNI            206 VFIVFMLAVA-AL-SLLLSL---AEL--YHLGW-K-----K---IRQRFVKP------------R 242
Pongo abelii CXNI               206 VFIVFMLAVA-AL-SLLLSL---AEL--YHLGW-K-----K---IRQRFVKP------------R 242
Nomascus leucogenys CXNI        206 VFIVFMLAVA-AL-SLLLSL---AEL--YHLGW-K-----K---IRQRFVKP------------R 242
Macaca mulatta CXNI             206 VFIVFMLAVA-AL-SLLLSL---AEL--YHLGW-K-----K---IRQRFVKP------------R 242
Papio hamadryas CXNI            206 VFIVFMLAVA-AL-SLLLSL---AEL--YHLGW-K-----K---IRQRFVKP------------R 242
Callithrix jacchus CXNI         206 VFIVFMLAVA-AL-SLFLSL---AEL--YHLGW-K-----K---IRQRCVKP------------R 242
Otolemur garnettii CXNI         205 VFIVFMLAVA-GL-SLFLSL---AEL--YHLGW-K-----K---IRRRFVRS------------H 241
Mus musculus Cxni               206 VFIVFMMAVA-GL-SLFLSL---AEL--YHLGW-K-----K---IRQRFGKS------------R 242
Rattus norvegicus Cxni          239 VFIVFMMAVA-GL-SLFLSL---AEL--YHLGW-K-----K---IRQRLAKS------------R 275
Cavia porcellus CXNI            207 VFIVFMLAVA-GL-SLFLSL---AEL--FHLGW-------R---IRQRFVKS------------R 242
Oryctolagus cuniculus CXNI      206 VFIVFMLAVA-GL-SLFLSL---AEL--YHLGW-K-----K---IGQRLARS------------P 242
```

Bos taurus CXNI	207	VFIVFMLAVA-GL-SLFLSL---AEL--YHLGW-K-----K---IRQRYVKS------------Q	243
Equus caballus CXNI	206	VFIVFMLAVA-GL-SLFLSL---VEL--YHLGW-K-----R---IRQRFARS------------R	242
Canis lupus familiaris CXNI	206	VFIVFMLAVA-AL-SLFLSL---AEL--YHLGW-K-----K---LRQRFVKS------------G	242
Felis catus CXNI	206	VFIVFMLAVA-AL-SLFLSL---AEL--YHLGW-K-----K---IRQRFAKS------------R	242
Pteropus vampyrus CXNI	206	VFIVFMLAVA-GL-SLVLSL---AEL--YHLGW-K-----K---FRQRFAKS------------R	242
Erinaceus europaeus CXNI	208	VFIVFMLAVA-GL-SLFLSL---AEL--YHLGW-K-----K---IRQRFSKG------------S	244
Dasypus novemcinctus CXNI	206	VFIVFMLAVA-GL-SLFLSL---AEL--YHLGW-K-----K---IRQRFAKS------------Q	242
Loxodonta africana CXNI	206	VFIVFMLAVA-AL-SLFLSL---AEL--YHLGW-K-----K---IRHRFVKS------------Q	242
Homo sapiens CXNJ1	202	IFIIFMLAVAC-A-SLLLNM---LEI--YHLGW-K-----K---LKQG-VTS------------R	237
Pongo abelii CXNJ1	202	IFIIFMLAVAC-A-SLLLNM---LEI--YHLGW-K-----K---LKQG-VTS------------R	237
Macaca mulatta CXNJ1	202	IFIIFMLAVAC-A-SLLLNM---LEI--YHLGW-K-----K---LKQG-MTS-------------	236
Mus musculus Cxnj1	208	IFVIFMLAVAC-A-SLVLNM---LEI--YHLGW-K-----K---LKQG-VTN------------H	243
Rattus norvegicus Cxnj1	208	IFVIFMLAVAC-A-SLVLNM---LEI--YHLGW-K-----K---LKQG-VTN------------H	243
Bos taurus CXNJ1	198	IFILFMLAVAC-V-SLLLNV---LEI--YHLGW-K-----K---LKQG-MTS------------P	233
Equus caballus CXNJ1	209	IFIIFMLAVAC-L-SLLLNM---LEI--YHLGW-K-----K---FKQG-MTN------------H	244
Myotis lucifugus CXNJ1	198	VFIIFMLVVAC-L-SLLLNV---LEI--YHLGW-K-----K---FKQG-MTN------------H	233
Myotis lucifugus CXNJ2	210	VFIIFMLVVAC-L-SLLLNV---LEI--YHLGW-K-----K---FKQG-MTN------------H	245
Pteropus vampyrus CXNJ1	367	IFIIFMLAVAC-L-SLFLNV---LEI--YHLGW-K-----K---LKQG-MTN------------Y	402
Pteropus vampyrus CXNJ2	289	IFIIFMLAVAC-L-SLFLNV---LEI--YHLGW-K-----K---LKHG-MTQ------------R	324
Sorex araneus CXNJ1	188	IFIIFMLAVAC-V-SLLLNV---LEI--YHLGW-K-----K---LKQG-VTN------------Q	223
Homo sapiens CXNK1	207	IFIIFMLVVS-LV-SLALNI---IEL--FYVFF-K--GV-K---DWVK-GKS------------D	244
Homo sapiens CXNK2	208	IFIIFMLVVS-LV-SLALNI---IEL--FYVFF-K--GV-K---DRVK-GKS------------D	245
Pan troglodytes CXNK1	207	IFIIFMLVVS-LV-SLALNI---IEL--FYVFF-K--GV-K---DRVK-GKS------------D	244
Pan troglodytes CXNK2	208	IFIIFMLVVS-LV-SLALNI---IEL--FYVFF-K--GV-K---DRVK-GKS------------D	245
Pongo abelii CXNK1	208	IFIIFMLVVS-LV-SLALNI---IEL--FYVFF-K--GV-K---DRVK-GKS------------D	245
Nomascus leucogenys CXNK1	208	IFIIFMLVVS-LV-SLALNI---IEL--FYVFF-K--GV-K---DRVK-GKS------------D	245
Callithrix jacchus CXNK1	208	IFIIFMLVVS-LV-SLALNI---IEL--FYVFF-K--GV-K---DRVK-GKS------------D	245
Mus musculus Cxnk1	208	IFIIFMLVVS-LV-SLALNI---IEL--FYVFF-K--GV-K---DRVK-GRS------------D	245
Mus musculus Cxnk2	208	IFILFMFVVS-VV-SFVLDI---IEL--FYVLF-K--AI-K---NRMRKAED------------E	246
Rattus norvegicus Cxnk1	208	IFIIFMLVVS-LV-SLALNI---IEL--FYVFF-K--GV-K---DRVK-GRS------------D	245
Rattus norvegicus Cxnk2	209	IFILFMLVVS-MV-SFVLNV---IEL--FYVLF-K--AI-K---NHLGNEKE------------E	247
Cavia porcellus CXNK1	208	IFIIFMLVVS-LV-SLALNI---IEL--FYVFF-K--GV-K---DRVK-GRS------------D	245
Oryctolagus cuniculus CXNK1	208	IFIIFMLVVS-LV-SLALNI---IEL--FYVFF-K--GV-K---DRVK-GKS------------D	245
Bos taurus CXNK1	209	IFIIFMLVVS-LV-SLALNI---IEL--FYVFF-K--GV-K---DRVK-GKS------------D	246
Vicugna pacos CXNK1	208	IFIIFMLVVS-LV-SLALNI---IEL--FYVFF-K--GV-K---DRVK-GKS------------D	245
Equus caballus CXNK1	208	IFIIFMLVVS-LV-SLALNV---IEL--FYVFF-K--GV-K---DRVK-GKG------------H	245
Equus caballus CXNK2	185	IFTLFMLVVS-LV-SLALNI---IEL--LCVLF-K--GI-K---DRSKDLEI------------E	223
Canis lupus familiaris CXNK1	208	IFIIFMLVVS-LV-SLALNI---IEL--FYVFF-K--GV-K---DRVK-GKS------------D	245
Canis lupus familiaris CXNK2	211	TFILFMLVVS-LV-SLALNI---IEL--FYIFF-K--TF-K---KHMKHPQS------------E	249
Felis catus CXNK1	206	IFILFMLVVS-LV-SLALNI---IEL--FSVFV-K--SI-K---HRVKDSES------------E	244
Myotis lucifugus CXNK1	208	IFIIFMLVVS-LV-SLALNI---IEL--FYVFF-K--GV-K---DRVK-GKS------------D	245
Dasypus novemcinctus CXNK1	208	IFIIFMLVVS-LV-SLTLNI---IEL--FSVFL-K--SF-K---ERVK-GQN------------D	245
Dasypus novemcinctus CXNK2	208	IFIIFMLVVS-LV-SLALNI---IEL--FYVFF-K--GV-K---DRVK-GKT------------D	245
Loxodonta africana CXNK1	208	IFIIFMLVVS-LV-SLALNI---IEL--FYVFF-K--GV-K---DRVK-GKS------------D	245
Loxodonta africana CXNK2	208	IFIIFMLVVS-VA-SLALNI---VEL--FCVLF-K--NI-K---DNLK-EQS------------D	245
Homo sapiens CXNL	204	IFILFMLSVA-SV-SLFLNV---MEL--GHLGL-K--GI-R---SALK--RPV-----------E	241
Pan troglodytes CXNL	204	IFILFMLSVA-SV-SLFLNV---MEL--GHLGL-K--GI-R---SALK--RPV-----------E	241
Pongo abelii CXNL	204	IFILFMLSVA-SV-SLFLNV---MEL--GHLGL-K--GI-R---SALR--RPV-----------E	241
Nomascus leucogenys CXNL	204	IFILFMLSVA-SV-SLFLNV---MEL--GHLGL-K--GI-R---SAFK--RPV-----------E	241
Macaca mulatta CXNL	210	IFILFMLSVA-SV-SLFLNV---MEL--GHLGL-K--GI-R---SAFK--RPV-----------E	247
Callithrix jacchus CXNL	208	IFILFMLSVA-SV-SLFLNV---MEL--GHLGL-K--GI-R---AAFK--RPV-----------E	245
Otolemur garnettii CXNL	255	IFILFMLSVA-SV-SLFLNI---MEM--SHLGL-K--GI-R---SAFK--RPT-----------E	292
Mus musculus Cxnl	211	IFILFMLSVA-FV-SLFLNI---MEM--SHLGM-K--GI-R---SAFK--RPV-----------E	248
Rattus norvegicus Cxnl	211	IFILFMLSVA-FV-SLFLNI---MEM--SHLGM-K--GI-R---SAFK--RPA-----------E	248
Cavia porcellus CXNL	211	IFILFMLSVA-SV-SLFLNI---MEM--SHLGL-K--GI-R---SAFK--RPV-----------E	248
Oryctolagus cuniculus CXNL	211	IFILFMLSVA-FV-SLFLNI---MEL--SHLGL-K--GI-R---SAFK--RPV-----------E	248
Ochotona princeps CXNL	208	IFILFMLSVA-FV-SLFLNI---MEI--SHLGL-K--GI-R---SAFK--RPV-----------E	245
Bos taurus CXNL	211	IFILFMLSVA-SV-SLFLNI---LEM--SHLGL-K--KI-R---SAFK--RPV-----------E	248
Equus caballus CXNL	210	IFILFMLSVA-SV-SLFLNI---LEI--SHLGL-K--KI-R---SALK--EPA-----------E	247
Canis lupus familiaris CXNL	211	IFILFMLSVA-SV-SLFLNI---LEM--SHLGL-K--RI-R---SAFK--RPV-----------E	248
Felis catus CXNL	211	IFILFMLSVA-SV-SLFLNI---LEM--SHLGL-K--RI-R---SAFK--RPV-----------E	248
Pteropus vampyrus CXNL	211	IFILFMLSVA-SV-SLFLNI---LEM--SHLGL-K--RI-R---SAFK--RQV-----------E	248
Sorex araneus CXNL	180	IFVLFMLSVA-SV-SLLLNV---TEL--GHLAL-K--RV-R---GAFK--RPV-----------P	217
Dasypus novemcinctus CXNL	211	IFILFMLSVA-SV-SLFLNI---MEM--SHLGL-K--RI-Q---SAFK--KPV-----------E	248
Loxodonta africana CXNL	210	VFILFMLSVA-SV-SLFLNI---MEI--SYLGL-K--RI-R---AAFK--KPV-----------E	247
Homo sapiens CXNM	210	IFLLFMQSIA-TI-SLFLNI---LEI--FHLGF-K-----K--IKRGLWGKY------------K	247
Pan troglodytes CXNM	210	IFLLFMQSIA-TI-SLFLNI---LEI--FHLGF-K-----K--IKRGLWGKY------------K	247
Pongo abelii CXNM	210	IFLLFMQSIA-TI-SLFLNI---LEI--FHLGF-K-----K--MKRGLWGKY------------K	247
Nomascus leucogenys CXNM	210	IFLLFMQSIA-TI-SLFLNI---LEI--FHLGF-K-----K--IKRGLWGKY------------K	247
Macaca mulatta CXNM	210	IFLLFMQSIA-TI-SLFLNI---LEI--FHLGF-K-----K--IKRGIWGKY------------K	247
Callithrix jacchus CXNM	209	IFLLFMQSIA-TI-SLFLNI---LEI--FHLGF-K-----K--VKRGLWGKY------------K	246
Tarsius syrichta CXNM	210	IFLLFMQSIA-TI-SLFLSI---LEI--FHLGF-K-----K--IKRGLWGH-------------K	246
Microcebus murinus CXNM	210	IFLLFMQSIA-TI-SLFLNI---LEI--FHLGF-K-----K--IKRGLWGQY------------K	247
Dipodomys ordii CXNM	211	IFLLFMQFIA-TV-SLFLNI---LEI--FHLGF-K-----K--IKKGLWGQY------------K	248
Oryctolagus cuniculus CXNM	208	IFLLFMQSIA-TV-SLFLSI---LEI--FYLGF-K-----K--IKRGLWGQS------------K	245
Equus caballus CXNM	210	IFLLFMQSIA-TV-SLFLNV---LEI--FHLGF-K-----K--IKRGLWGQY------------K	247
Canis lupus familiaris CXNM	210	IFLLFMQSIA-TV-SLFLNV---LEI--FHLGF-K-----K--IKRGLWGQY------------K	247
Pteropus vampyrus CXNM	209	IFLLFMQSIA-TV-SLFLNV---LEI--FHLGF-K-----K--VKRGLWGRY------------K	246
Loxodonta africana CXNM	210	IFLLFMQSIA-TV-SLFLNV---LEI--AHLGF-K-----K--IKGGLWGQY------------K	247

```
Homo sapiens CXNN                210 IFMLFMHSIA-AI-SLLLNI---LEI--FHLGI-R-----K--IMRTLY-K-------------K 245
Pan troglodytes CXNN             210 IFMLFMHSIA-AI-SLLLNI---LEI--FHLGI-R-----K--IMRTLY-K-------------K 245
Pongo abelii CXNN                210 IFMLFMHSIA-AI-SLLLNI---LEI--FHLGI-R-----K--IMRTLY-K-------------K 245
Nomascus leucogenys CXNN         210 IFMLFMHSIA-AI-SLLLNI---LEI--FHLGI-R-----K--IMRTLY-K-------------K 245
Macaca mulatta CXNN              210 IFMLFMHSIA-AI-SLLLNI---LEI--FHLGI-R-----K--IMRTLY-K-------------K 245
Callithrix jacchus CXNN          210 IFMLFMHSIA-AI-SLLLNI---LEI--FHLGI-R-----K--VMRTLY-K-------------K 245
Microcebus murinus CXNN          210 IFMLFMHSIA-AI-SLLLNI---LEI--FHLGI-R-----K--IMRTLH-K-------------K 245
Mus musculus Cxnn                210 IFMLFMHSIA-AI-SLLLNI---LEI--FHLGI-R-----K--IMRALD-G-------------K 245
Rattus norvegicus Cxnn           210 IFMLFMHSIA-AI-SLLLNI---LEI--FHLGI-R-----K--IMRALD-G-------------K 245
Oryctolagus cuniculus CXNN       240 IFMLFMQSIA-AI-SLLLNV---LEI--FHLGV-R-----K--IMKALY-E-------------K 275
Bos taurus CXNN                  210 IFMLFMHSIA-AI-SLFLNI---LEI--FHLGI-R-----K--IMRALY-D-------------K 245
Equus caballus CXNN              210 IFMLFMHSIA-AI-SLFLNV---LEI--FHLGI-R-----K--IMRALY-E-------------R 245
Canis lupus familiaris CXNN      210 IFMLFMHSIA-AI-SLFLNV---LEI--FHLGI-R-----K--ITRALY-E-------------K 245
Sorex araneus CXNN               210 IFMLFMQSIA-AV-SLFLNG---LEI--FHLGI-R-----K--IIRAVH-E-------------K 245
Loxodonta africana CXNN          210 IFMIFMHSIA-AI-SLFLNV---LEI--FHLGI-R-----K--IGRALY-E-------------K 245
Homo sapiens CXNO                266 VFLLVMYVVSC-LC-LLLNLC---EM--AHLGL-GS-A--Q-----------------------D 296
Papio hamadryas CXNO             266 VFLLVMYVVSC-LC-LLLNLC---EM--AHLGL-GS-A--Q-----------------------D 296
Mus musculus Cxno                276 VFLLVMYVVSC-LC-LLLNLC---EM--AHLGL-GS-A--Q-----------------------D 306
Cavia porcellus CXNO             260 VFLLVMYVVSC-LC-LLLNLC---EM--AHLGL-GS-A--Q-----------------------D 290
Homo sapiens CXNP1               190 IFLKTMFGVS-GFC-LLFTF---LEL--VLLGL-G-----R-----------------------W 218
Pan troglodytes CXNP1            190 IFLKTMFGVS-GFC-LLFTF---LEL--VLLGL-G-----R-----------------------W 218
Pongo abelii CXNP1               190 IFLKTMFGLS-GFC-LLFTF---LEL--VLLGL-G-----R-----------------------W 218
Callithrix jacchus CXNP1         191 IFLKTMFGVS-GFC-LLFTL---LEL--VLLGL-E-----R-----------------------W 219
Otolemur garnettii CXNP1         191 VFLKTMFGVS-GFC-LLFTL---LEL--VLLGL-T-----R-----------------------W 219
Tupaia belangeri CXNP1           208 IFLKTMFGVS-GFC-LLLAV---LEL--VLLSL-G-----R-----------------------W 236
Mus musculus Cxnp1               191 IFLNIMFGIS-GAC-FLFIF---LEL--ALLGL-G-----R-----------------------F 219
Rattus norvegicus Cxnp1          190 ILLNTMFGIS-GAC-LLFIF---LEL--VLLGL-G-----R-----------------------V 218
Cavia porcellus CXNP1            191 IFLKTMFGVN-GLC-LLFTL---LEL--MLLGL-G-----R-----------------------L 219
Oryctolagus cuniculus CXNP1      191 VFLKAMFGVN-GLC-LFFTL---LEL--VLLGL-G-----R-----------------------W 219
Oryctolagus cuniculus CXNP2      191 IFLKTMFGVS-GVC-LFFTL---LEL--VLLGL-G-----R-----------------------W 219
Ochotona princeps CXNP1          191 IFLKAMFGIS-GLC-LFLTL---LEL--VLLGL-G-----R-----------------------W 219
Bos taurus CXNP1                 192 IFLKTMFGVT-GLC-LLFTL---LEL--VLLGL-G-----R-----------------------W 220
Equus caballus CXNP1             191 IFLKTMFGVT-GLC-LLFTL---LEL--VLLGL-G-----R-----------------------W 219
Canis lupus familiaris CXNP1     189 IFLKTMFGVS-GLC-LLFTL---VEL--VLLGL-G-----K-----------------------W 217
Felis catus CXNP1                212 IFLKTMFGVS-GLC-LLFTL---LEL--VLLGL-G-----R-----------------------W 240
Myotis lucifugus CXNP1           191 IFLKTMFGLS-GLC-LFFTL---LEL--VLLGL-G-----R-----------------------L 219
Dasypus novemcinctus CXNP1       190 LFLQAMFGVS-GLC-LCLTL---LEL--V-LGL-G-----R-----------------------W 217
Dasypus novemcinctus CXNP2       190 LFLQAMFGVS-GLC-LCLTL---LEL--V-LGL-R-----R-----------------------W 217
Dasypus novemcinctus CXNP3       190 IFLKAMFGVS-GLC-LCLTL---LEL--VLLGL-G-----R-----------------------W 218
Dasypus novemcinctus CXNP4       190 LFLQAMFGVS-GLC-LCLTF---LEL--V-LGL-G-----R-----------------------W 217
Choloepus hoffmanni CXNP1        189 IFLKTMFGVS-WLC-FSSMF---LEL--VLLGL-R-----R-----------------------R 217
Loxodonta africana CXNP1           - -----------------------------------------------------------------   -
Homo sapiens CXNQ                230 IFLLIMYGVT-GLC-LLLNI---WEM--LHLGF-GT-I--R-----------------------D 260
Pan troglodytes CXNQ             230 IFLLIMYGVT-GLC-LLLNI---WEM--LHLGF-GT-I--R-----------------------D 260
Pongo abelii CXNQ                230 IFLLIMYGVT-GLC-LLLNI---WEM--LHLGF-GT-I--R-----------------------D 260
Macaca mulatta CXNQ              230 IFLLIMYGVT-GLC-LLLNI---WEM--LHLGF-GT-I--R-----------------------D 260
Tupaia belangeri CXNQ            230 IFLLIMYGVT-GLC-LLLNI---WEM--LHLGF-GT-I--R-----------------------D 260
Mus musculus Cxnq                230 IFLLIMYGVT-GLC-LLLNI---WEM--LHLGF-GT-I--R-----------------------D 260
Rattus norvegicus Cxnq           230 IFLLIMYGVT-GLC-LLLNI---WEM--LHLGF-GT-I--R-----------------------D 260
Cavia porcellus CXNQ             230 IFLLIMYGVT-GLC-LLLNI---WEM--LHLGF-GT-I--R-----------------------D 260
S.tridecemlineatus CXNQ          244 IFLLIMYGVT-GLC-LLLNI---WEM--LHLGF-GT-I--R-----------------------D 274
Oryctolagus cuniculus CXNQ       230 IFLLIMYGVT-GLC-LLLNI---WEM--LHLGF-GT-I--R-----------------------D 260
Bos taurus CXNQ                  230 IFLLIMYGVT-GLC-LLLNI---WEM--LHLGF-GT-I--R-----------------------D 260
Vicugna pacos CXNQ               230 IFLLIMYGVT-GLC-LLLNI---WEM--LHLGF-GT-I--R-----------------------D 260
Equus caballus CXNQ              230 IFLLIMYGVT-GLC-LLLNI---WEM--LHLGF-GT-I--R-----------------------D 260
Canis lupus familiaris CXNQ      230 IFLLIMYGVT-GLC-LLLNI---WEM--LHLGF-GT-I--R-----------------------D 260
Myotis lucifugus CXNQ            230 IFLLIMYGVT-GLC-LLLNI---WEM--LHLGF-GT-I--R-----------------------D 260
Pteropus vampyrus CXNQ           230 IFLLIMYGVT-GLC-LLLNV---WEM--LHLGF-GT-I--R-----------------------D 260
Loxodonta africana CXNQ          230 IFLLIMYGVT-GLC-LLLNI---WEM--LHLGF-GT-I--R-----------------------D 260
Homo sapiens CXNR                189 VFVLFYFAV--GL--LSALL-SVAEL--GHL-L-WK-G--R----P-RAG--------------E 222
Pan troglodytes CXNR             189 VFVLFYFAV--GL--LSALL-SVAEL--GHL-L-WK-G--R----P-RAG--------------E 222
Pongo abelii CXNR                189 VFVLFYFAV--GL--LSALL-SVAEL--GHL-L-WK-G--R----P-RAG--------------E 222
Papio hamadryas CXNR             189 VFVLFYFAV--GL--LSALL-SVAEL--GHL-L-WK-G--R----P-RAG--------------E 222
Mus musculus Cxnr                189 VFVVFYFAV--GL--LSALL-SVAEL--GHL-L-WK-G--R----Q-RA---------------- 220
Rattus norvegicus Cxnr           189 VFVVFYFAV--GL--LSALL-SVAEL--GHL-L-WK-G--R----Q-RA---------------- 220
Oryctolagus cuniculus CXNR       197 VFLLFYFAV--GL--LSALL-SVAEL--GHL-L-SK-G--R----P-RAG--------------E 230
Bos taurus CXNR                  179 VFLVFYFAV--GL--LSALL-SVAEL--GHL-L-WK-G--G----S-RAGSYLQAA--------E 218
Homo sapiens CXNS                201 VFLVFMFAVS-GIC-VVLNL---AEL--NHLG--WR----K----I-KL---------------- 231
Pan troglodytes CXNS             201 VFLVFMFAVS-GIC-VVLNL---AEL--NHLG--WR----K----I-KL---------------- 231
Pongo abelii CXNS                201 VFLVFMFAVS-GIC-VVLNL---AEL--NHLG--WR----K----I-KL---------------- 231
Nomascus leucogenys CXNS         201 VFLVFMFAVS-GIC-VVLNL---AEL--NHLG--WR----K----I-KL---------------- 231
Macaca mulatta CXNS              201 VFLVFMFAVS-GIC-VVLNL---AEL--NHLG--WR----K----I-KL---------------- 231
Papio hamadryas CXNS             201 VFLVFMFAVS-GIC-VVLNL---AEL--NHLG--WR----K----I-KL---------------- 231
Otolemur garnettii CXNS          201 VFLVFMFAVS-GIC-VVLNL---AEL--NHLG--WR----K----I-KL---------------- 231
Tupaia belangeri CXNS            201 VFLVFMFAVS-GIC-VVLNL---AEL--NHLG--WR----K----I-KL---------------- 231
Mus musculus Cxns                241 VFLVFMFAVS-GIC-VVLNL---AEL--NHLG--WR----K----I-KL---------------- 271
Rattus norvegicus Cxns           201 VFLVFMFAVS-GIC-VVLNL---AEL--NHLG--WR----K----I-KL---------------- 231
Cavia porcellus CXNS             201 VFLVFMFAVS-GIC-VVLNL---AEL--NHLG--WR----K----I-KL---------------- 231
S.tridecemlineatus CXNS          201 VFLVFMFAVS-GIC-VVLNL---AEL--NHLG--WR----K----I-KL---------------- 231
```

```
Oryctolagus cuniculus CXNS    201 VFLVFMFAVS-GIC-VVLNL---AEL--NHLG--WR----K----I-KL---------------- 231
Ochotona princeps CXNS        201 VFLVFMFAVS-GIC-VVLNL---AEL--NHLG--WR----K----I-KL---------------- 231
Bos taurus CXNS               201 VFLVFMFAVS-GIC-VVLNL---AEL--NHLG--WR----K----I-KL---------------- 231
Equus caballus CXNS           201 VFLVFMFAVS-GIC-VVLNL---AEL--NHLG--WR----K----I-KL---------------- 231
Canis lupus familiaris CXNS   201 VFLVFMFAVS-GIC-VVLNL---AEL--NHLG--WR----K----I-KL---------------- 231
Myotis lucifugus CXNS         201 VFLVFMFAVS-GIC-VVLNL---AEL--NHLG--WR----K----I-KL---------------- 231
Dasypus novemcinctus CXNS     201 VFLVFMFAVS-GIC-VVLNL---AEL--NHLG--WR----K----I-KL---------------- 231
Loxodonta africana CXNS       329 VFLVFMFAVS-GIC-VVLNL---AEL--NHLG--WR----K----I-KL---------------- 359
Homo sapiens CXNT             171 IFLIAINTFT-TI-TILL--F-VAEI--FEIIF-RR----LY--FPFRQ---------------- 205
Pan troglodytes CXNT          171 IFLIAINTFT-TI-TILL--F-VAEI--FEIIF-RR----LY--FPFRQ---------------- 205
Nomascus leucogenys CXNT      174 IFLIAMNTFT-AI-TMVL--C-VAEI--FEIIF-RR----LY--FPFRQ---------------- 208
Mus musculus Cxnt             175 IFLIAMYTFT-VI-TMVL--C-VAEV--FEIIF-RR----SC--FLFKR---------------- 209
Rattus norvegicus Cxnt        175 IFLIAMYTFT-VI-TMVL--C-VAEI--FEIIF-RR----SC--FLFKR---------------- 209
Cavia porcellus CXNT          171 IFLIAMYTFT-VI-TMVL--C-IGEV--FEIIF-RR----LC--F-VRQ---------------- 204
Oryctolagus cuniculus CXNT    175 IFLIAMYTFT-VI-TIIL--C-IAEV--FEIIF-RR----LC--FLIRQ---------------- 209
Bos taurus CXNT               175 IFLIAMYTFT-AI-TIVL--C-VAEI--FEIIF-RR----LC--FLISQ---------------- 209
Vicugna pacos CXNT            175 IFLIAMYTFT-VI-TIVL--C-VAEI--FEIVF-RR----LC--LLMMQ---------------- 209
Equus caballus CXNT           175 IFLIAMYTFT-VI-TMVL--C-VAEI--FEIIF-RR-----C--FLIRQ---------------- 208
Canis lupus familiaris CXNT   175 IFLIAMYTFT-VI-TVVL--C-VAEV--FEIIF-RR----LC--FLIRQ---------------- 209
Myotis lucifugus CXNT         175 IFLIAMYTFT-VI-TMVL--C-VAEV--FEIIF-RR----LC--FLIKQ---------------- 209
Dasypus novemcinctus CXNT     175 IFLISMYTFT-VI-TMVL--C-AAEV--FEIIF-RR----LC--HLNHQ---------------- 209
Loxodonta africana CXNT       171 IFLVAMYTFT-VI-TMVL--C-VAEI--FEIIF-RR----LC--SLI------------------ 203
Homo sapiens CXNU             182 LLMLFLWAVS-AL-SFLL---GLADLVCS-L---RR----R---MRRRPG--------------- 215
Pan troglodytes CXNU          182 LLMLFLWAVS-AL-SFLL---GLADLVCS-L---RL----R---MRRRQR--------------- 215
Pongo abelii CXNU             167 LLMLFLWAVS-AL-SFLL---GLTDLVCS-L---RR----R---MRRRPG--------------- 200
Nomascus leucogenys CXNU      179 LLMLFLWAVS-AL-SFLL---GLGDLVCS-L---RR----R---MRRRPG--------------- 212
Macaca mulatta CXNU           182 LLMLFLWAVS-AL-SFLL---GLADLVCS-L---RR----R---MRRRPG--------------- 215
Mus musculus Cxnu             152 LLILFFWAVS-AL-SFLL---SLADLL-W-I---LP----R---RKTLRTTQWVNGEARPVCE-- 197
Cavia porcellus CXNU          145 IMVLFTWTMS-VL-SFLL---SVADLVCS-V---QG----R---ISRRQGPGQRVSSSCVGKEYG 193
Bos taurus CXNU               165 ILMLFVWAMC-AL-SFLL---TVADLVCS-V---CW----K---TQGRPGE-------------- 199
Equus caballus CXNU           173 IMMLFLWAVS-AL-SLLL---SVADLACS-A---RR----R---MRRGSGRGCG----------- 210
Myotis lucifugus CXNU         189 MLMLFLWALS-AL-SVLL---SVADLLCS-L---GR----R---TLGEPG--------------- 222
Sorex araneus CXNU            161 ILMLFVWAVS-AL-SLLL---SVADLVCS-L---RR----R---LSPSRG--------------- 194
Dasypus novemcinctus CXNU     191 IMMLFIWGAS-AL-SLLL---TVADLLCS-V---RR----R---LARRRGSTQKAENSLLRKGDD 239
Loxodonta africana CXNU       200 IMMLFIWGVS-TL-SFLL---GLADLMCS-V---RR----R---VRRRRAPKRVARTSSSRTESQ 248
```

```
Homo sapiens CXNA              221 KAQAMCT-----G---H----H--PHGTTSSC-----------K--QDDL-LS-GDL--IF-LGS 253
                                      590       600       610       620       630       640       650
Homo sapiens CXNA              221 KAQAMCT-----G---H----H--PHGTTSSC-----------K--QDDL-LS-GDL--IF-LGS 253
Pan troglodytes CXNA           221 KARAMCT-----A---H----H--PHGTTSSC-----------K--QDDL-LS-GDL--IF-LGS 253
Gorilla gorilla CXNA           221 KAQAMCT-----G---H----H--PHGTTSSC-----------K--QDDL-LS-GDL--IF-LGS 253
Pongo abelii CXNA              221 KARAMCT-----G---H----H--PHGTTSSC-----------K--QDDL-LS-GDL--IF-LGS 253
Nomascus leucogenys CXNA       221 KARAMCT-----G---H----H--PHGTTSSC-----------K--QDDL-LS-GDL--IF-LGS 253
Macaca mulatta CXNA            221 KAQAMYT-----G---H----H--PHGTTSSC-----------K--QDDL-LS-GDL--IF-LGS 253
Callithrix jacchus CXNA        221 KARATCT-----G---Y----H--PHGATSPC-----------K--QDDL-LL-GDL--IF-LAS 253
Mus musculus Cxna              219 RPPTAHA-----K---N----D--PNWANSPS-----------K--EKDF-LS-CDL--IF-LGS 251
Rattus norvegicus Cxna         219 RPPTAHA-----K---N----D--PNWANPSS-----------K--EKDF-LS-SDL--IF-LGS 251
Cavia porcellus CXNA           271 KVQATPV-----G---H----Y--PDCVTASC-----------K--QKDL-LS-GDL--IF-LGP 303
Ochotona princeps CXNA         221 RAQNTYL-----R---S----H--PDSV---C-----------K--QDDL-FS-GDL--IF-LGC 250
Bos taurus CXNA                221 KARSESL-----D---H----R--LDWVTSSS-----------K--QGDL-LS-GDL--IF-LDS 253
Equus caballus CXNA            221 KTQGAGV-----G---H----R--QDCATSFR-----------K--QDDL-LS-DDL--IF-LGS 253
Canis lupus familiaris CXNA    221 TARAEGA-----C---R----C--PDCTASSR-----------A--GRDL-LS-GDL--LL-LGS 253
Felis catus CXNA               221 RARATGG-----G---H----R--PDCPTSSY-----------K--EDDL-LS-GDL--IF-LGS 253
Myotis lucifugus CXNA          253 KARAASA---------------------SSC-----------K--QEDL-LS-GDL--IF-LGS 277
Dasypus novemcinctus CXNA      221 RGQATSL-----E---L----R--PDGGPSPC-----------K--QDDL-LS-GDL--IF-LGS 253
Loxodonta africana CXNA        221 RARTPYK-----E---H----H--PDCATSSC-----------K--QDDI-LL-GDL--VF-LES 253
Homo sapiens CXNB              221 HRRPRCR-----E---C----L--PD--T--C-----------P--PY-V-LSQGGH--PE-DG- 248
Gorilla gorilla CXNB           221 HRRPRRR-----E---R----L--PD--T--C-----------P--PY-V-LSQGGH--PE-DG- 248
Nomascus leucogenys CXNB       221 HRRSRRR-----E---R----L--PD--T--C-----------P--PY-V-LSQGGH--PE-DG- 248
Macaca mulatta CXNB            221 HRRSRRR-----E---R----L--PD--T--C-----------P--PY-V-LSQGGH--PE-DG- 248
Callithrix jacchus CXNB        221 HRRSRCR-----E---R----L--PD--T--C-----------P--PY-V-LPQGGH--PE-DG- 248
Otolemur garnettii CXNB        221 RRRSRRR-----Q--GC----L--PD--T--C-----------P--PY-V-LSQGGH--PE-DG- 249
Mus musculus Cxnb              221 RRKASRR-----H---Q----L--PD--T--C-----------P--PY-V-ISKGGH--PQ-DE- 248
Rattus norvegicus Cxnb         221 RRKTSRR-----H---Q----L--PD--T--C-----------P--PY-V-ISK-GH--PQ-DE- 247
Dipodomys ordii CXNB           221 RRKSRHR-----H---H----L--PD--T--C-----------P--PY-V-ISQGGH--PH-EG- 248
Cavia porcellus CXNB           221 PRKSRHR-----C---H----L--PD--T--C-----------P--PY-V-VSQEGH--PQ-DG- 248
Oryctolagus cuniculus CXNB     129 RRRSRHR-----S---R----L--PD--T--C-----------P--PY-V-LSQGEP--PG-KE- 156
Ochotona princeps CXNB         221 HRHSRSR-----G-----------D--T--C-----------P--PY-I-LSYREH--TQ-NG- 245
Bos taurus CXNB                221 PRGSRRP-----A---R----L--LE--A--C-----------P--PY-A-LSPGEH--PQ-DG- 248
Equus caballus CXNB            221 HQKSRRH-----G---H----L--PD--T--C-----------P--PY-A-LSQGEP--HQ-DG- 248
Canis lupus familiaris CXNB    271 RRRPHHR-----D---R----L--PD--T--C-----------P--PY-A-LSQGEP--PQ-DG- 298
Myotis lucifugus CXNB          222 RHRSRRR-----S---C----L--HD--S--C-----------P--PY-A-LSQGDH--PQ-DG- 249
Erinaceus europaeus CXNB       221 RRRSRRQ-----N---H----L--PD--T--C-----------P--PY-I-LSQGEH--TQ-DG- 248
Loxodonta africana CXNB        221 RRRSGHR-----D---H----P--PD--T--C-----------L--PY-A-LSQGGH--PQ-DG- 248
Homo sapiens CXNC              222 KPRGGCS-----P---S----S--SASRASTC-----------RC-HHKL-VEAGEV-DPD-PG- 256
Gorilla gorilla CXNC           222 KPRGGCS-----P---S----S--SASRASTC-----------RC-HHKL-VEAGEV-DPD-PG- 256
Pongo abelii CXNC              222 KPRGGRS-----P---S----S--STSRASTC-----------RC-HRKL-VEAGEV-DPD-PG- 256
Nomascus leucogenys CXNC       222 KPRGGRS-----P---S----S--SASRASTC-----------RC-HHKL-VEAGEL-DPD-PG- 256
Callithrix jacchus CXNC        222 KPQGGHT-----P---S----S--FASRASTC-----------RC-HHKL-VEAGEL-DPH-PG- 256
Microcebus murinus CXNC        252 RPPRGYS-----P---S----P--SKSRASTC-----------RC-HHKL-VEAGEL-GLD-PG- 286
Otolemur garnettii CXNC        222 RPSGVRG-----S---S----A--SNSRASTC-----------RC-HHKL-VEAGEL-DPD-LG- 256
Mus musculus Cxnc              222 KSTKSIS-----S---P----K--SSSRASTC-----------RC-HHKL-LESGDP-EAD-PA- 256
Rattus norvegicus Cxnc         222 KSTKSIS-----S---P----K--SSSRASTC-----------RC-HHKL-LESGDL-EAV-PA- 256
Cavia porcellus CXNC           222 KVPKGHS--------------T--SS---STC-----------RC-HHKL-VEAGEV-GPD-LG- 251
Oryctolagus cuniculus CXNC     332 RPSKGRS-----P---P----A--STSRASIC-----------HC-HHKL-LEAGDL-EPE-AN- 366
Bos taurus CXNC                222 RQPGGRG-----H---A----P--SASRASTC-----------RC-HHKL-LEAGDL-SPD-SH- 256
Equus caballus CXNC            222 RPPGGHS-----P---A----S--STSQASTC-----------HR-HHKLLVEAGEL-ELD-PG- 257
Canis lupus familiaris CXNC    222 RSPKSRG-----P---P----S--STSRASTC-----------RC-HHKL-VDAGEL-GCD-GG- 256
Felis catus CXNC               222 KPPKGHS-----L---P----S--STSRASTC-----------RC-HHKL-VEAGEL-GSD-SG- 256
Myotis lucifugus CXNC          222 RPPGSRN-----R---K----S--SASRASTC-----------RC-HHKL-VE-GEF-VLD-AH- 255
Dasypus novemcinctus CXNC      224 RSPQARG-----P---S----S--SASRASTC-----------RC-HHKL-LQAGGP-SPN-PG- 258
Loxodonta africana CXNC        222 KGPRGSS-----R---P----S--STSRASTC-----------RC-HHKL-IEAAEL------G- 252
Homo sapiens CXND              215 LKK-PQV-----LS--V------------------------------------------------- 223
Pan troglodytes CXND           215 LKK-PQV-----LS--V------------------------------------------------- 223
Macaca mulatta CXND            215 SKKTPIP-----QC--VS-------ATASDML-----------N--VVGE-GPYSRI-IIH-TWP 249
Callithrix jacchus CXND        215 SKKFKSS-----VC--EC---H---GLRYVQC-----------G--GRGA-PPYFRI-IIH-AWP 250
Dipodomys ordii CXND           215 SKRLKCS-----EY--QS---H---HLRSVEC-----------G--VMGA-PP-QLY-NHH-LDL 249
Oryctolagus cuniculus CXND     215 SKNLRCS-----GC--EC---H---NFRCVGS-----------N--KMGI-PA-LLQ-NHH-SGL 249
Tursiops truncatus CXND        215 SKKLKSS-----VC--EC---H---NLRYVKY-----------G--EMGA-PP-LLQ-KHH-LDS 249
Bos taurus CXND                133 SKRLKSS-----TC--EC---H---NLKYVTC-----------A--EIGA-PP-LLQ-KHS-SDL 167
Equus caballus CXND            234 PKSLKSS-----VC--EC---H---HLRYIEC-----------G--ETGV-PP-LLQ-NHH-SDS 268
Canis lupus familiaris CXND    215 SKRLQSS-----AC--EC---H---DLRYVEC-----------G--VIAA-PP-LLQ-NCR-SDL 249
Myotis lucifugus CXND          215 SKRPKFS-----VC--EC---P---NLRYVEC-----------G--EVGA-LP-LLQ-NHH-SAS 249
Erinaceus europaeus CXND       215 SRRFTSS-----VC--EC---H---NLKYFEC-----------V--ETGT-FPPQLQ-NPL-SEV 250
Dasypus novemcinctus CXND      215 FKELKPS-----VC--EC---H---NLPYVEC-----------G--EVRA-PP-LLQ-NHN-SDL 249
Choloepus hoffmanni CXND       215 SKKLKSS-----VY--EC---H---NLRYIEC-----------G--ETGA-PP-LLQ-KHN-SDL 249
Loxodonta africana CXND        215 SKKLKTS-----VC--EC---H---NLSYVER-----------G--EIGG-PP-LLQ-KHN-SDS 249
Homo sapiens CXNE                - ----------------------------------------------------------------   -
Pan troglodytes CXNE             - ----------------------------------------------------------------   -
Pongo abelii CXNE                - ----------------------------------------------------------------   -
Macaca mulatta CXNE              - ----------------------------------------------------------------   -
Papio hamadryas CXNE             - ----------------------------------------------------------------   -
Callithrix jacchus CXNE          - ----------------------------------------------------------------   -
Otolemur garnettii CXNE          - ----------------------------------------------------------------   -
Mus musculus Cxne                - ----------------------------------------------------------------   -
```

```
Rattus norvegicus Cxne          - -----------------------------------------------------------------   -
Dipodomys ordii CXNE            - -----------------------------------------------------------------   -
Cavia porcellus CXNE            - -----------------------------------------------------------------   -
Oryctolagus cuniculus CXNE      - -----------------------------------------------------------------   -
Equus caballus CXNE             - -----------------------------------------------------------------   -
Canis lupus familiaris CXNE     - -----------------------------------------------------------------   -
Felis catus CXNE                - -----------------------------------------------------------------   -
Myotis lucifugus CXNE           - -----------------------------------------------------------------   -
Sorex araneus CXNE              - -----------------------------------------------------------------   -
Dasypus novemcinctus CXNE       - -----------------------------------------------------------------   -
Loxodonta africana CXNE         - -----------------------------------------------------------------   -
Homo sapiens CXNF             225 AQTQKNH-----P---N---------HALK----------------ESKQ-NEMNEL-IS--D-S 251
Pan troglodytes CXNF          225 AQTQKNH-----P---N---------HALK----------------ESKQ-NEMNEL-IS--D-S 251
Gorilla gorilla CXNF          225 AQTQKNH-----P---N---------HALK----------------ESKQ-NEMNEL-IS--D-S 251
Nomascus leucogenys CXNF      225 AQTQRNH-----P---N---------HALK----------------ESKQ-NEMNEL-IS--D-S 251
Callithrix jacchus CXNF       225 AQSQSNH-----P---N---------HTLK----------------ENKQ-NEMNEL-IS--D-S 251
Mus musculus Cxnf             225 TQAQRNH-----P---N---------HALK----------------ESKQ-NEMNEL-IS--D-S 251
Rattus norvegicus Cxnf        225 TQAQRNH-----P---N---------HALK----------------ESKQ-NEMNEL-IS--D-S 251
S.tridecemlineatus CXNF       225 TQTQRNH-----P---N---------HAIK----------------ESKQ-NEMNEL-IS--D-S 251
Oryctolagus cuniculus CXNF    225 AQAQRNH-----P---N---------YALK----------------ESKQ-NEMNEL-IS--D-S 251
Ochotona princeps CXNF        225 AQAHKNH-----P---N---------HTLR----------------ESKE-NEMNEL-IS--D-S 251
Bos taurus CXNF               225 AQTQKAP-----P---N---------HALK----------------ESKQ-NEMNEL-IS--E-G 251
Equus caballus CXNF           225 GQTQRNH-----P---N---------HALK----------------ESKQ-NEMNEL-IS--D-S 251
Canis lupus familiaris CXNF   225 TQTQRNH-----P---N---------HALK----------------ESKQ-NEMNEL-IS--D-S 251
Myotis lucifugus CXNF         225 AQTQRNH-----P---S---------HALK----------------ESKQ-NEMNEL-IS--D-S 251
Dasypus novemcinctus CXNF     225 AQVQRNH-----P---N---------HAIK----------------ESKQ-NEINEL-IS--D-S 251
Choloepus hoffmanni CXNF      225 AQTEKNH-----P---N---------HAIK----------------ESKQ-NEMNEL-IS--D-S 251
Loxodonta africana CXNF       225 AQMQRNH-----T---D---------HALK----------------ESKQ-NEMNEL-IS--D-S 251
Homo sapiens CXNG             225 SNPPSRK-----G---S----G--FGHRLS--------------P--EYKQ-NEINKL-LSEQDGS 258
Pan troglodytes CXNG          225 SNPPSRK-----G---S----G--FGHRLS--------------P--EYKQ-NEINKL-LSEQDGS 258
Gorilla gorilla CXNG          225 SNPPSRK-----G---S----G--FGHRLS--------------P--EYKQ-NEINKL-LSEQDGS 258
Pongo abelii CXNG             225 SNPPSRK-----G---S----G--FGHRLS--------------P--EYKQ-NEINKL-LSEQDGS 258
Nomascus leucogenys CXNG      225 SNPPSRK-----G---S----G--FGHRLS--------------P--EYKQ-NEINKL-LSEQDGS 258
Macaca mulatta CXNG           225 SNPPSRK-----G---S----G--FGHRLS--------------P--EYKQ-NEINKL-LSEQDGS 258
Papio hamadryas CXNG          225 SNPPSRK-----G---S----G--FGHRLS--------------P--EYKQ-NEINKL-LSEQDGS 258
Callithrix jacchus CXNG       225 SNPPSRK-----G---S----G--FGHRLS--------------P--EYKQ-NEINKL-LSEQDGS 258
Microcebus murinus CXNG       225 SNPPSRK-----G---S----G--FGHRLS--------------P--EYKQ-NEINKL-LSEQDGS 258
Mus musculus Cxng             225 SNPPSRK-----G---S----G--FGHRLS--------------P--EYKQ-NEINKL-LSEQDGS 258
Rattus norvegicus Cxng        225 SNPPSRK-----G---S----G--FGHRLS--------------P--EYKQ-NEINKL-LSEQDGS 258
Oryctolagus cuniculus CXNG    225 SNPPSRK-----G---S----G--FGHRLS--------------P--EYKQ-NEINKL-LSEQDGS 258
Bos taurus CXNG               225 SNPPSRK-----G---S---GG--FGHRLS--------------P--EYKQ-NEINKL-LSEQDGS 259
Equus caballus CXNG           225 SNPPSRK-----G---S----G--FGHRLS--------------P--EYKQ-NEINKL-LSEQDGS 258
Canis lupus familiaris CXNG   225 SNPPSRK-----G---S----G--FGHRLS--------------P--EYKQ-NEINKL-LSEQDGS 258
Felis catus CXNG              225 SNPPSRK-----G---S----G--FGHRLS--------------P--EYKQ-NEINKL-LSEQDGS 258
Myotis lucifugus CXNG         225 SNPPSRK-----G---S----G--FGHRLS--------------P--EYKQ-NEINKL-LSEQDGS 258
Pteropus vampyrus CXNG        225 SNPTSRK-----G---S----G--FGHRLS--------------P--EYKQ-NEINKL-LSEQDGS 258
Dasypus novemcinctus CXNG     225 SNPPSRK-----G---S----G--FGHRLS--------------P--EYKQ-NEINKL-LSEQDGS 258
Loxodonta africana CXNG       225 SNPPSRK-----G---S----G--FGHRLS--------------P--EYKQ-NEINKL-LSEQDGS 258
Procavia capensis CXNG        225 SNPPSRK-----G---S----G--FGHRLS--------------P--EYKQ-NEINKL-LSEQDGS 258
Homo sapiens CXNH1            248 APPTQGT---SSD--PY-T-DQ--VFFY-L-------------P-VGQGP-SSPP--C-P-TYNG 283
Pan troglodytes CXNH1         248 APPTQGT---SSD--PY-T-DQ--VFFY-L-------------P-VGQGP-SSPP--C-P-TYNG 283
Gorilla gorilla CXNH1         248 APPTQGT---SSD--PY-T-DQ--VFFY-L-------------P-VGQGP-SSPP--C-P-TYNG 283
Pongo abelii CXNH1            248 APPTQGT---SSD--PY-T-DQ--VFFY-L-------------P-VGQGP-SSPP--C-P-TYNG 283
Nomascus leucogenys CXNH1     248 APPTQGT---SSD--PY-T-DQ--VFFY-L-------------P-VGQGP-SSPP--C-P-TYNG 283
Macaca mulatta CXNH1          248 APPTQGT---SSD--PY-T-DQ--VFFY-L-------------P-MGQGP-SSPP--C-P-TYNG 283
Otolemur garnettii CXNH1      245 TPPVQGT---SSE--RF-T-NQ--VFIY-LT------------H-KGQEP-SSPS--C-P-TDNA 281
Mus musculus Cxnh1            248 ARPAQGS---ASD--PY-P-EQ--VFFY-L-------------P-MGEGP-SSPP--C-P-TYNG 283
Rattus norvegicus Cxnh1       248 TRPAQGS---ASD--PY-P-EQ--VFFY-L-------------P-MGEGP-SSPP--C-P-TYNG 283
Cavia porcellus CXNH1         248 VSPARAT---FSD--SY-P-DQ--VFFY-L-------------P-VGEGP-SSPP--C-P-TYTG 283
Oryctolagus cuniculus CXNH1   248 APPAQGT---ASD--PY-A-DQ--VFFY-L-------------P-MGEGP-SSPP--C-P-TYNG 283
Ochotona princeps CXNH1       262 APPAQDA---SSD--PY-A-DQ--VFFY-L-------------P-MGEGP-SSPP--C-P-TYNG 297
Equus caballus CXNH1          248 APPAQGT---SSE--PY-A-DQ--VFFY-L-------------P-MGEGP-SSPP--C-P-TYNG 283
Canis lupus familiaris CXNH1  248 APPPRGA---SSE--PY-A-DQ--VFFY-L-------------P-MGEGP-SSPP--C-P-TYNG 283
Felis catus CXNH1             248 VPPAQRT---SSE--PY-P-DQ--VFFY-L-------------P-VGEGP-SSPP--C-P-TYNG 283
Myotis lucifugus CXNH1        280 APLAQGT---TPE--PY-A-NQ--VF-Y-L-------------P-MGKEP-SSEP--C-P-VYSG 314
Dasypus novemcinctus CXNH1    248 APPAQGT---SSD--PY-A-EQ--VFFY-L-------------P-MGEGP-SSPP--F-P-TYNG 283
Loxodonta africana CXNH1      248 APPAQGA---SSD--PY-S-DQ--VFFY-L-------------P-MGKGP-SSPP--C-P-TYNG 283
Homo sapiens CXNI             243 Q-HMA-KC--QL----S-G-PS--VGIV-QSC--------------TPPP--D-FNQC-L--ENG 274
Pan troglodytes CXNI          243 Q-HMA-KC--QL----S-G-PS--VGIV-QSC--------------TPPP--D-FNQC-L--ENG 274
Pongo abelii CXNI             243 Q-HMA-KC--QL----S-G-PS--VGIV-QSC--------------TPPP--D-FNQC-L--ENG 274
Nomascus leucogenys CXNI      243 Q-HMA-KC--QL----S-G-PS--VGIV-QSC--------------TPPP--D-FNQC-L--ENG 274
Macaca mulatta CXNI           243 Q-HVA-KC--QL----S-G-PS--VSMV-QSC--------------TPPP--D-FNQC-L--ENG 274
Papio hamadryas CXNI          243 Q-HVA-KC--QL----S-G-PS--VSMV-QSC--------------TPPP--D-FNQC-L--ENG 274
Callithrix jacchus CXNI       243 Q-DMA-KC--QL----S-G-PS--ASIV-QSC--------------TPPP--D-FNQC-L--QSG 274
Otolemur garnettii CXNI       242 Q-GMA-EC--QL----P-G-PS--AGMV-QSC--------------TPPP--D-FNRC-L--ENG 273
Mus musculus Cxni             243 Q-GVD-KH--QL----P-G-PP--TSLV-QSL--------------TPPP--D-FNQC-L--KNS 274
Rattus norvegicus Cxni        276 Q-G-D-KH--QL----L-G-PS--TSLV-QGL--------------TPPP--D-FNQC-L--KNS 306
Cavia porcellus CXNI          243 Q-GTA-ER--QL----L-G-PS--TGIV-QNC--------------TPPP--D-FTQC-L--GTG 274
Oryctolagus cuniculus CXNI    243 Q-SSA-EP--RL----P-G-PS--AGTA-QSC--------------TPPP--D-FTQC-L--ENG 274
```

```
Bos taurus CXNI                    244 P-GVG-EC--QL----P-G-PS--AGRV-QSC--------------TPPP--D-FNQC-L--ENG 275
Equus caballus CXNI                243 Q-GMA-EC--QL----P-G-PS--AGIV-QKC--------------TPPL--D-FNQC-L--ENG 274
Canis lupus familiaris CXNI        243 Q-GMA-EC--QL----P-G-PS--AGIV-QNC--------------TPPP--D-FNQC-L--ENG 274
Felis catus CXNI                   243 Q-GTA-EC--QL----P-G-PI--AGIV-QNC--------------TPPP--D-FNQC-L--ENG 274
Pteropus vampyrus CXNI             243 P-GVA-EC--QL----P-G-PA--AGLA-QSC--------------TPPP--A-FSQC-L--ENG 274
Erinaceus europaeus CXNI           245 R-GTA-EC--QP----P---PA--AGLV-QNC--------------TPPP--D-FSQC-L--ENG 275
Dasypus novemcinctus CXNI          243 Q-GMA-ES--QL----P-S-PS--AGMA-QRG--------------TPPP--D-FNQC-L--ENG 274
Loxodonta africana CXNI            243 H-GMT-ES--QL----P-V-PS--TGMA-QSC--------------TPPP--D-FNQC-L--ENG 274
Homo sapiens CXNJ1                 238 L-G-P-D-------------AS--EAPL-GT--------------ADPPP--LPPSS--R-PPAV 264
Pongo abelii CXNJ1                 238 L-G-P-E-------------AS--EAPL-GT--------------ADPPP--LPPSS--R-PPAV 264
Macaca mulatta CXNJ1               237 L-G-P-D-------------AS--EAPP-GT--------------ADPPP--LPRSS--R-P-AA 262
Mus musculus Cxnj1                 244 F-N-P-D-------------AS--EARH-KP--------------LDPLP--TATSS--G-PPSV 270
Rattus norvegicus Cxnj1            244 F-N-P-D-------------AS--EVRH-KP--------------LDPLS--EAANS--G-PPSV 270
Bos taurus CXNJ1                   234 F-R-P-D---TPG---S-R-AG--SVKP-VG--------------GSPLL--LPPNS--A-PPAV 265
Equus caballus CXNJ1               245 Y-S-P-D---TPG---T-G-MG--ATKP-EG--------------VTPLH--VPSNS--A-PATI 276
Myotis lucifugus CXNJ1             234 Y-G-P-D---TVE---S-R-VG--AAIS-GD--------------VIPLP----SH--------- 257
Myotis lucifugus CXNJ2             246 Y-G-P-D---TVE---S-R-VG--AAIS-GD--------------VIPLP----SH--------- 269
Pteropus vampyrus CXNJ1            403 Y-S-V-E---TPQ---S-R-LG--AAKP-GG--------------VSPLP---PSLS--A-PATG 433
Pteropus vampyrus CXNJ2            325 Y-S-L-D---SPE---F-R-MG--PSKP-GS--------------VSPLP---PSH--------- 349
Sorex araneus CXNJ1                224 Y-R---E---VP----P-R-SW--EVGA-EE--------------QVPVL--VLQSQ--V-SGP- 252
Homo sapiens CXNK1                 245 PYHAT-----------T-G-P---LSPA-KDC-------------GSQKY--AYFNGC-S-SPTA 275
Homo sapiens CXNK2                 246 PYHAT-----------S-G-A---LSPA-KDC-------------GSQKY--AYFNGC-S-SPTA 276
Pan troglodytes CXNK1              245 PYHAT-----------T-G-T---LSPA-KDC-------------GSQKY--AYFNGC-S-SPTA 275
Pan troglodytes CXNK2              246 PYHAT-----------S-G-T---LSPA-KDC-------------GSQKY--AYFNGC-S-SPTA 276
Pongo abelii CXNK1                 246 PYHAT-----------S-G-A---LSPA-KDC-------------GSQKY--AYFNGC-S-SPTA 276
Nomascus leucogenys CXNK1          246 PYHAP-----------S-G-P---LSPA-KDC-------------GSQKY--AYFNGC-S-SPTA 276
Callithrix jacchus CXNK1           246 PYHAT-----------T-G-P---LSPS-KDC-------------GSPKY--AYFNGC-S-SPTA 276
Mus musculus Cxnk1                 246 PYHAT-----------T-G-P---LSPS-KDC-------------GSPKY--AYFNGC-S-SPTA 276
Mus musculus Cxnk2                 247 VY----------------------------C--------------DELP--------C-P-SHVS 259
Rattus norvegicus Cxnk1            246 PYHAT-----------T-G-P---LSPS-KDC-------------GSPKY--AYFNGC-S-SPTA 276
Rattus norvegicus Cxnk2            248 VY----------------------------C--------------NPVE--LQKPSC-V-SSSA 265
Cavia porcellus CXNK1              246 PYHPA-----------G-G-P---LSPS-KDC-------------GSPKY--AYFNGC-S-SPTA 276
Oryctolagus cuniculus CXNK1        246 PYHAT-----------T-G-P---LSPS-KDC-------------GSPKY--AYFNGC-S-SPTA 276
Bos taurus CXNK1                   247 PYHTT-----------T-G-P---LSPS-KDC-------------GSPKY--AYFNGC-S-SPTA 277
Vicugna pacos CXNK1                246 PYHAT-----------T-G-P---LSPS-KDC-------------GSPKY--AYFNGC-S-SPTA 276
Equus caballus CXNK1               246 PYHTT-----------I-G-P---LSPS-KDV-------------GSPKY--AYFNGC-S-SPTA 276
Equus caballus CXNK2               224 TYHNT-----------S-A-L---QSPS---------------------------NG--S-SSTA 242
Canis lupus familiaris CXNK1       246 PYHAT-----------T-G-P---LSPS-KDC-------------GSPKY--AYFNGC-S-SPTA 276
Canis lupus familiaris CXNK2       250 TYHDT-----------S-G-L---PSPF---------------------------ND--S-FLVL 268
Felis catus CXNK1                  245 SYYVI-----------S-G-L---LRPS-K-------------------------N---S-SSFV 263
Myotis lucifugus CXNK1             246 PYHAN-----------T-G-P---LSPS-KEC-------------GSPKY--TYFNGC-S-SPTA 276
Dasypus novemcinctus CXNK1         246 S---------------------------------------------------------------- 246
Dasypus novemcinctus CXNK2         246 PYHAT-----------T-G-P---LSPA-KDC-------------GSQKY--AYFNGC-S-SPTA 276
Loxodonta africana CXNK1           246 PYHAT-----------T-G-P---LSPS-KEC-------------GSPKY--AYFNGC-S-SPTA 276
Loxodonta africana CXNK2           246 LCHST-----------S-S-P---RSLP-KDP-------------DSPQY--AYFNAG-F-SSAV 276
Homo sapiens CXNL                  242 QP--LGE---IPE--KS-L-HS--IA-V-SS--------------I-QKA-KGY--Q--LLEEEK 273
Pan troglodytes CXNL               242 QP--LGE---IPE--KS-L-HS--IA-V-SS--------------I-QKA-KGY--Q--LLEEEK 273
Pongo abelii CXNL                  242 QP--LGE---IPE--KS-L-HS--IA-V-SS--------------I-QKA-KGY--Q--LLEEEK 273
Nomascus leucogenys CXNL           242 QP--LGE---IPE--KS-L-HS--IA-V-SS--------------I-QKA-KGY--Q--LLEEEK 273
Macaca mulatta CXNL                248 QP--LGE---IPE--KS-L-HS--IA-V-SS--------------I-QKA-KGY--Q--LLEEEK 279
Callithrix jacchus CXNL            246 QP--LGE---IPE--KS-L-HS--IA-V-SS--------------I-QKA-KGY--Q--LLEEEK 277
Otolemur garnettii CXNL            293 QP--LGE---LPE--KS-L-HS--IA-V-SS--------------I-QKA-KGY--Q--LLEEEK 324
Mus musculus Cxnl                  249 QP--LGE---IAE--KS-L-HS--IA-V-SS--------------I-QKA-KGY--Q--LLEEEK 280
Rattus norvegicus Cxnl             249 QP--LGE---IAE--KS-L-HS--IA-V-SS--------------I-QKA-KGY--Q--LLEEEK 280
Cavia porcellus CXNL               249 QP--LGE---IPE--KS-I-HS--IA-V-SS--------------I-QKA-KGY--Q--LLEEEK 280
Oryctolagus cuniculus CXNL         249 QP--LGE---IPE--KS-L-HS--IA-V-SS--------------I-QKA-KGY--Q--LLEEEK 280
Ochotona princeps CXNL             246 QP--LGE---IPE--KS-L-HS--IA-V-SS--------------I-QKA-KGY--Q--LLEEEK 277
Bos taurus CXNL                    249 QP--LGE---IPE--KS-L-HS--IA-V-SS--------------I-QKA-KGY--Q--LLEEEK 280
Equus caballus CXNL                248 QP--LGE---VPE--KS-L-HS--IA-V-SS--------------I-QKA-KGY--Q--LLEEEK 279
Canis lupus familiaris CXNL        249 QP--LGE---IPE--KS-L-HS--IA-V-SS--------------I-QKA-KGY--Q--LLEEEK 280
Felis catus CXNL                   249 QP--LGE---IPE--KS-L-HS--IA-V-SS--------------I-QKA-KGY--Q--LLEEEK 280
Pteropus vampyrus CXNL             249 QP--LGE---IPE--KS-L-HS--IA-V-SS--------------I-QKA-KGY--Q--LLEEEK 280
Sorex araneus CXNL                 218 QS--PGT---ASE--KS-L-HS--VA-I-SS--------------V-QKA-KGY--R--FLEEEK 249
Dasypus novemcinctus CXNL          249 PSRSLGE---TPE--KP-L-HS--IA-V-SS--------------I-QKA-KGY--Q--LLEEEK 282
Loxodonta africana CXNL            248 QP--LGE---ISE--NP-L-HS--IA-V-SS--------------I-QKA-KGY--Q--LLEEEK 279
Homo sapiens CXNM                  248 LKKEHNE-FHAN------K-AK--QNVAKYQ---------------------------STSANS 274
Pan troglodytes CXNM               248 LKKEHNE-FHAN------K-AK--QNVAKYQ---------------------------STSANS 274
Pongo abelii CXNM                  248 LKKEHNE-FHAN------K-AK--QNVAKYH---------------------------STSANS 274
Nomascus leucogenys CXNM           248 LKKEHNE-FHAN------K-AI--QNVAKYQ---------------------------STSANS 274
Macaca mulatta CXNM                248 LKKEHNE-FHAN------E-AK--QNVAKYQ---------------------------STSANS 274
Callithrix jacchus CXNM            247 LKKEHNE-LYAN------K-AK--ENVAKYQ---------------------------SMSANS 273
Tarsius syrichta CXNM              247 LKDEHNE-FYAS------K-SK--QNLVKYQ---------------------------STSANS 273
Microcebus murinus CXNM            248 LKDEHNE-FYAN------K-SK--QNLAKYQ---------------------------NTSANS 274
Dipodomys ordii CXNM               249 SKYKHNQ-YHAN------K-SK--LNLAKYQ---------------------------GTPTNS 275
Oryctolagus cuniculus CXNM         246 WKGEHKE-FHAN------Q-SK--QN----Q---------------------------RAPAKS 268
Equus caballus CXNM                248 LKDEHNE-FYVN------K-SK--QNLAKYQ---------------------------NTSANS 274
Canis lupus familiaris CXNM        248 LKDEHNE-FFAN------K-SK--QNLAKYQ---------------------------STSANS 274
Pteropus vampyrus CXNM             247 LKDEHNE-FY--------K-SK--QNLAKYQ---------------------------STSANS 271
Loxodonta africana CXNM            248 LKDEHNE-FFAN------K-SK--QNPTKYK---------------------------SVSANS 274
```

Homo sapiens CXNN	246	SSSEGIE-DETGP--PF-H-LK--KYSVAQQC-----------M-I-----------C-S-SL-P	277
Pan troglodytes CXNN	246	SSNEGIE-DETGP--PF-H-LK--KYSVAQQC-----------M-I-----------C-S-SL-P	277
Pongo abelii CXNN	246	SSSEGTE-DETGP--PF-H-LK--KYSVAQQC-----------M-I-----------C-S-SL-P	277
Nomascus leucogenys CXNN	246	SSSEGIE-DETGP--PF-H-LK--KYSVAQQC-----------M-I-----------C-S-SL-P	277
Macaca mulatta CXNN	246	SSGEGIE-DETGP--PF-H-LK--KYSVAQQC-----------M-I-----------C-S-SL-P	277
Callithrix jacchus CXNN	246	SSSEGIE-DETGP-PPF-H-LN--KYSVAQQC-----------M-I-----------C-S-SL-P	278
Microcebus murinus CXNN	246	SSNEGIE-NERVS--PF-H-VK--KYSVAQQC-----------M-I-----------C-S-SL-P	277
Mus musculus Cxnn	246	SSSGNTE-NETGP--PF-H-ST--NYSGTQQC-----------M-I-----------C-S-SL-P	277
Rattus norvegicus Cxnn	246	SSSGNTE-NETGP--PF-H-ST--NYSGAQQC-----------M-V-----------C-S-SL-P	277
Oryctolagus cuniculus CXNN	276	SSNEGFE-NERGP--PF-Q-LR--KYSAAQQC-----------M-I-----------C-S-SL-P	307
Bos taurus CXNN	246	SSSEGIE-DERRL--PF-H-LK--KYSVTQEC-----------M-T-----------C-S-PF-P	277
Equus caballus CXNN	246	SSNECIE-EERGP--PV-R-LK--KYSVAQQS-----------M-I-----------C-S-SL-P	277
Canis lupus familiaris CXNN	246	SSNEGTE-EESGT--PF-H-LK--KYSVAQEC-----------M-I-----------C-S-PL-S	277
Sorex araneus CXNN	246	PGGDSTE-SEKGP--QL-A-LE--KYSVVQPC-----------M-I-----------C-S-SL-H	277
Loxodonta africana CXNN	246	PNSEVTE-DERGP--PF-H-LK--KCSVAQQS-----------M-I-----------C-S-SL-P	277
Homo sapiens CXNO	297	AV---RG----RR---G-PPAS---APAPAP---------------RPPP-------C-AFPAAA	324
Papio hamadryas CXNO	297	AV---RG----RR---G-PPASG-PAPAPAP---------------RPPP-------C-AFPAAA	326
Mus musculus Cxno	307	AV---RG----RR---G---ASA-AGPGPTP---------------RPPP-------C-AFPAAA	334
Cavia porcellus CXNO	291	AV---RV----RR---G-PAVSATTSPPAPP---------------RPAP-------C-ALELGA	321
Homo sapiens CXNP1	219	W--	219
Pan troglodytes CXNP1	219	W--	219
Pongo abelii CXNP1	219	W--	219
Callithrix jacchus CXNP1	220	W--	220
Otolemur garnettii CXNP1	220	W--	220
Tupaia belangeri CXNP1	237	W--	237
Mus musculus Cxnp1	220	W--	220
Rattus norvegicus Cxnp1	219	W--	219
Cavia porcellus CXNP1	220	W--	220
Oryctolagus cuniculus CXNP1	220	C--	220
Oryctolagus cuniculus CXNP2	220	W--	220
Ochotona princeps CXNP1	220	W--	220
Bos taurus CXNP1	221	W--	221
Equus caballus CXNP1	220	W--	220
Canis lupus familiaris CXNP1	218	W--	218
Felis catus CXNP1	241	W--	241
Myotis lucifugus CXNP1	220	W--	220
Dasypus novemcinctus CXNP1	218	R--	218
Dasypus novemcinctus CXNP2	218	W--	218
Dasypus novemcinctus CXNP3	219	W--	219
Dasypus novemcinctus CXNP4	218	R--	218
Choloepus hoffmanni CXNP1	218	F--	218
Loxodonta africana CXNP1	-	---	-
Homo sapiens CXNQ	261	SLN--SK----RR---E-LEDPG-AYNYPFT---------------WNTP---------SAPPG-	289
Pan troglodytes CXNQ	261	SLN--SK----RR---E-LEDPG-AYNYPFT---------------WNTP---------SAPPG-	289
Pongo abelii CXNQ	261	SLN--SK----RR---E-LEDPG-AYNYPFT---------------WNTP---------SAPPG-	289
Macaca mulatta CXNQ	261	SLN--SK----RR---E-LEDPG-AYNYPFT---------------WNTP---------SAPPG-	289
Tupaia belangeri CXNQ	261	SLN--SK----RR---E-LEDPG-AYNYPFT---------------WNTP---------SAPPG-	289
Mus musculus Cxnq	261	SLN--SK----RR---E-LDDPG-AYNYPFT---------------WNTP---------SAPPG-	289
Rattus norvegicus Cxnq	261	SLN--SK----RR---E-LDDPG-AYNYPFT---------------WNTP---------SAPPG-	289
Cavia porcellus CXNQ	261	SLH--SK----QR---E-LEEPG-AYSYPFT---------------WNTP---------SAPPG-	289
S.tridecemlineatus CXNQ	275	SLN--SK----RR---E-LEDPG-AYNYPFT---------------WNTP---------SAPPG-	303
Oryctolagus cuniculus CXNQ	261	SLN--SK----RR---E-LEDPG-AYNYPFT---------------WNTP---------SAPPG-	289
Bos taurus CXNQ	261	SLN--SK----RR---E-LEDPG-AYNYPFT---------------WNTP---------SAPPG-	289
Vicugna pacos CXNQ	261	SLN--NK----RR---D-LEDPG-AYNYPFT---------------WNTP---------SAPPG-	289
Equus caballus CXNQ	261	SLN--SK----RR---E-LEDPG-AYNYPFT---------------WNTP---------SAPPG-	289
Canis lupus familiaris CXNQ	261	SLN--SK----RR---E-LEDPG-AYNYPFT---------------WNTP---------SAPPG-	289
Myotis lucifugus CXNQ	261	SLN--SK----RR---E-LEDPG-VYNYPFT---------------WNTP---------SAPPG-	289
Pteropus vampyrus CXNQ	261	SLN--SK----RR---E-LEDPG-VYNYPFT---------------WNTP---------SAPPG-	289
Loxodonta africana CXNQ	261	SLN--SK----RR---E-LEDPG-AYNYPFT---------------WNTP---------SAPPG-	289
Homo sapiens CXNR	223	RDN--R-C---NR---A-HEEAQ-KLLPPPP---------------PPPP---------P-PALP	251
Pan troglodytes CXNR	223	RDN--R-C---NR---A-HEEAQ-KLLPPP----------------PPPP---------P-PALP	250
Pongo abelii CXNR	223	CDN--R-C---NR---A-HEEAQ-KLLPPPP---------------PPPP---------P-PALP	251
Papio hamadryas CXNR	223	RDN--R-C---NR---A-HEEAQ-KLLPPPP---------------PPPP---------PLPTLP	252
Mus musculus Cxnr	221	------------------------KLLPPPP---------------P-------------SPSLP	233
Rattus norvegicus Cxnr	221	------------------------KLLPPPP---------------P-------------SPSLP	233
Oryctolagus cuniculus CXNR	231	RDN--R-C---NR---A-HEEAQ-KLLRPPA---------------P--------------PAPP	255
Bos taurus CXNR	219	RDN--R-C---NR---A-HEKAQ-QLLQP-------------------------------LPALP	241
Homo sapiens CXNS	232	AV---RG-AQAKR--KS-IYEIRNKDLPRVS---------------VP-----------------	257
Pan troglodytes CXNS	232	AV---RG-AQAKR--KS-IYEIRNKDLPRVS---------------VP-----------------	257
Pongo abelii CXNS	232	AV---RG-AQAKR--KS-IYEIRNKDLPRVS---------------VP-----------------	257
Nomascus leucogenys CXNS	232	AV---RG-AQAKR--KS-IYEIRNKDLPRVS---------------VP-----------------	257
Macaca mulatta CXNS	232	AV---RG-AQAKR--KS-IYEIRNKDLPRVS---------------VP-----------------	257
Papio hamadryas CXNS	232	AV---RG-AQAKR--KS-IYEIRNKDLPRVS---------------VP-----------------	257
Otolemur garnettii CXNS	232	AV---RG-AQAKR--KS-VYEIRNKDLPRVS---------------VP-----------------	257
Tupaia belangeri CXNS	232	AV---RG-AQAKR--KS-VYEIRNKDLPRVS---------------VP-----------------	257
Mus musculus Cxns	272	AV---RG-AQAKR--KS-VYEIRNKDLPRVS---------------VP-----------------	297
Rattus norvegicus Cxns	232	AV---RG-AQAKR--KS-VYEIRNKDLPRVS---------------VP-----------------	257
Cavia porcellus CXNS	232	AV---RG-AQAKR--KS-VYEIRNKDLPRVS---------------VP-----------------	257
S.tridecemlineatus CXNS	232	AV---RG-AQAKR--KS-VYEIRNKDLPRVS---------------VP-----------------	257

```
Oryctolagus cuniculus CXNS    232 AV---RG-AQAKR--KS-VYEIRNKDLPRVS---------------VP----------------- 257
Ochotona princeps CXNS        232 AV---RG-AQAKR--KS-VYEIRNKDLPRVS---------------VP----------------- 257
Bos taurus CXNS               232 AV---RG-AQAKR--KS-VYEIRNKDLPRVS---------------VP----------------- 257
Equus caballus CXNS           232 AV---RG-AQAKR--KS-VYEIRNKDLPRVS---------------VP----------------- 257
Canis lupus familiaris CXNS   232 AV---RG-AQAKR--KS-VYEIRNKDLPRVS---------------VP----------------- 257
Myotis lucifugus CXNS         232 AV---RG-AQAKR--KS-VYEIRNKDLPRVS---------------VP----------------- 257
Dasypus novemcinctus CXNS     232 AV---RG-AQAKR--KS-VYEIRNKDLPRVS---------------VP----------------- 257
Loxodonta africana CXNS       360 AV---RG-AQAKR--KS-VYEIRNKDLPRVS---------------VP----------------- 385
Homo sapiens CXNT                - -----------------------------------------------------------------   -
Pan troglodytes CXNT             - -----------------------------------------------------------------   -
Nomascus leucogenys CXNT         - -----------------------------------------------------------------   -
Mus musculus Cxnt                - -----------------------------------------------------------------   -
Rattus norvegicus Cxnt           - -----------------------------------------------------------------   -
Cavia porcellus CXNT             - -----------------------------------------------------------------   -
Oryctolagus cuniculus CXNT       - -----------------------------------------------------------------   -
Bos taurus CXNT                  - -----------------------------------------------------------------   -
Vicugna pacos CXNT               - -----------------------------------------------------------------   -
Equus caballus CXNT              - -----------------------------------------------------------------   -
Canis lupus familiaris CXNT      - -----------------------------------------------------------------   -
Myotis lucifugus CXNT            - -----------------------------------------------------------------   -
Dasypus novemcinctus CXNT        - -----------------------------------------------------------------   -
Loxodonta africana CXNT          - -----------------------------------------------------------------   -
Homo sapiens CXNU             216 -PP--TS-PSIRK--QS-G-ASGHAEG-RRT---DEEGGREEEG-APAPP---------GARAGG 258
Pan troglodytes CXNU          216 -PP--TN-PSIRK--QS-G-ASGHAEG-RRT---DEEDGREEEG-APVPP---------GARAGG 258
Pongo abelii CXNU             201 -PS--TS-PTIRK--QS-G-APGHAEG-RRT---DEESSREEEG-TPAPP---------GARAGG 243
Nomascus leucogenys CXNU      213 -RP--TS-PSIRK--HS-G-APGHAEG-RWT---DEEGGREEEG-APAPP---------GARAGG 255
Macaca mulatta CXNU           216 -PP--TS-PSIRK--QS-G-APGHPEG-RPT---DKEGGREQEG-APAPP---------VARAGG 258
Mus musculus Cxnu             198 VPA---P-PPCLL--QN----PQGYLS-QGQ--VDQEDRQEEQV-VPEFP---------CMWTAG 239
Cavia porcellus CXNU          194 VRA--SP-PGSAK--GN-Q--PSKV---------DEDGRWEEEV-LPSLP---------DEWAGG 231
Bos taurus CXNU               200 -AR--RR-PCPGA--RD-A-AP---EG-RGA-R-EGLGVHEGRG-ERART---------GLGTRG 240
Equus caballus CXNU           211 -AA--RA-PRGQG--LA-G-GRS------PG-HVARRGEWEGGA-VPALP---------ARWAGG 250
Myotis lucifugus CXNU         223 -PA--EC-PRERR----------------PD-PVVMDRLGEERV-LLAHQ---------GLWTRG 256
Sorex araneus CXNU            195 -AH--ST-PRGLP-------------A-ATK-ALGQDGGWEAAG-PPAHP---------SSWRGT 230
Dasypus novemcinctus CXNU     240 VSR--CP-PGQAE--KR-V-ASQSLGR-KPS-QISEGGGWEAEG-VSALP---------GMWPRE 285
Loxodonta africana CXNU       249 GLK--SP-PGHSA--ER-V-ASRSLER-RRS-HISEDDGWEEQE-VPIHP---------RVWARE 294
```

```
Homo sapiens CXNA              254 DSHP-PLLPDRPRD-HV--KK-TIL--------------------------------------- 273
                                           660       670       680       690       700       710
Homo sapiens CXNA              254 DSHP-PLLPDRPRD-HV--KK-TIL--------------------------------------- 273
Pan troglodytes CXNA           254 DSHP-PLLPDRPRD-HV--KK-TIL--------------------------------------- 273
Gorilla gorilla CXNA           254 DSHP-PLLPDRPRD-HV--KK-TIL--------------------------------------- 273
Pongo abelii CXNA              254 DSHP-PLLPDRPRD-HV--KK-TIL--------------------------------------- 273
Nomascus leucogenys CXNA       254 DSHP-PLLPDRPQD-HV--KT-TIL--------------------------------------- 273
Macaca mulatta CXNA            254 DSHP-PLLPDRPQD-HV--KK-TIL--------------------------------------- 273
Callithrix jacchus CXNA        254 DSHP-SLLPDCPRD-HA--KK-TIL--------------------------------------- 273
Mus musculus Cxna              252 DAHP-PLLPDRPRA-HV--KK-TIL--------------------------------------- 271
Rattus norvegicus Cxna         252 DTHP-PLLPDRPRA-HV--KK-TIL--------------------------------------- 271
Cavia porcellus CXNA           304 DTHS-PLVPDCPRD-YV--KK-TVV--------------------------------------- 323
Ochotona princeps CXNA         251 DTPP-PLLPDCPRD-NV--KK-TIL--------------------------------------- 270
Bos taurus CXNA                254 DVPP-PLLPDHPQD-HV--KK-TML--------------------------------------- 273
Equus caballus CXNA            254 EAHP-PLLPNRPRG-HV--KK-TIL--------------------------------------- 273
Canis lupus familiaris CXNA    254 DPQP-PLSPDHPRD-HV--KK-TIL--------------------------------------- 273
Felis catus CXNA               254 DSHP-PLLPDRPRD-HV--KK-TML--------------------------------------- 273
Myotis lucifugus CXNA          278 DTHP-PLLPDLARD-YV--KK-TIL--------------------------------------- 297
Dasypus novemcinctus CXNA      254 DAHP-PLLPDQPRD-HV--KK-TVL--------------------------------------- 273
Loxodonta africana CXNA        254 DTHP-PLLSDHPRD-HV--KK-TIL--------------------------------------- 273
Homo sapiens CXNB              249 NSVL-MKAGSAPVD-AG--GY-P----------------------------------------- 266
Gorilla gorilla CXNB           249 NSVL-MKAGSAPVD-AG--GY-P----------------------------------------- 266
Nomascus leucogenys CXNB       249 NSVL-MKAGSAPVD-AG--GY-P----------------------------------------- 266
Macaca mulatta CXNB            249 NSVL-IKAGSAPVD-AG--RY-P----------------------------------------- 266
Callithrix jacchus CXNB        249 NSVL-MKAGSALVD-AG--GY-P----------------------------------------- 266
Otolemur garnettii CXNB        250 NSVL-MKAGSAPMD-AG--GY-P----------------------------------------- 267
Mus musculus Cxnb              249 SVIL-TKAGMATVD-AG--VY-P----------------------------------------- 266
Rattus norvegicus Cxnb         248 STVL-TKAGMATVD-AG--VY-P----------------------------------------- 265
Dipodomys ordii CXNB           249 SSAL-VKASSATME-AG--GY-P----------------------------------------- 266
Cavia porcellus CXNB           249 NSVL-KKAESALMD-ES--GY-P----------------------------------------- 266
Oryctolagus cuniculus CXNB     157 DSVL-MKA---PVD-TG--VY-P----------------------------------------- 171
Ochotona princeps CXNB         246 DSVP-MKAVPAAVD-TG--MF-P----------------------------------------- 263
Bos taurus CXNB                249 NSVL-MKAEMTTVD-AA--GF-P----------------------------------------- 266
Equus caballus CXNB            249 TSVL-MKAGSATVD-AA--VY-P----------------------------------------- 266
Canis lupus familiaris CXNB    299 NSVL-MKAGSASMD-AG--GF-P----------------------------------------- 316
Myotis lucifugus CXNB          250 NSVL-MKASSAEVD-AG--GY-P----------------------------------------- 267
Erinaceus europaeus CXNB       249 NSVL-MKAGSVAMN-TG--GY-A----------------------------------------- 266
Loxodonta africana CXNB        249 TSVL-VKAGLAPMD-TD--GY-T----------------------------------------- 266
Homo sapiens CXNC              257 NNKL--QA-SAPNL-T------PI---------------------------------------- 270
Gorilla gorilla CXNC           257 NNKL--QA-SAPNL-T------PI---------------------------------------- 270
Pongo abelii CXNC              257 NNKL--QA-SAPNL-T------PI---------------------------------------- 270
Nomascus leucogenys CXNC       257 SNKL--QA-SAPNL-T------PI---------------------------------------- 270
Callithrix jacchus CXNC        257 NNKL--QA-SAPNM-T------PI---------------------------------------- 270
Microcebus murinus CXNC        287 NDKL--QA-SAPNL-T------PT---------------------------------------- 300
Otolemur garnettii CXNC        257 DDKL--QA-SAPKL-T------PI---------------------------------------- 270
Mus musculus Cxnc              257 SEKL--QA-SAPSL-T------PI---------------------------------------- 270
Rattus norvegicus Cxnc         257 DDKL--QA-SAPSL-T------PI---------------------------------------- 270
Cavia porcellus CXNC           252 DAKL--EA-SAPKL-T------PI---------------------------------------- 265
Oryctolagus cuniculus CXNC     367 DAKL--RA-SAPKL-S------AI---------------------------------------- 380
Bos taurus CXNC                257 DNKL--CA-SAPTM-T------PI---------------------------------------- 270
Equus caballus CXNC            258 SDKP--CA-SAPSM-T------PI---------------------------------------- 271
Canis lupus familiaris CXNC    257 DDEG--HA-SAPHM-T------PI---------------------------------------- 270
Felis catus CXNC               257 DDKR--HA-SAPSM-T------PI---------------------------------------- 270
Myotis lucifugus CXNC          256 GDKP--HA-SAPAL-T------PI---------------------------------------- 269
Dasypus novemcinctus CXNC      259 DDAS--QA-SAPNL-T------PV---------------------------------------- 272
Loxodonta africana CXNC        253 DDRL--QA-SAPNL-T------SI---------------------------------------- 266
Homo sapiens CXND                - ----------------------------------------------------------------   -
Pan troglodytes CXND             - ----------------------------------------------------------------   -
Macaca mulatta CXND              - ----------------------------------------------------------------   -
Callithrix jacchus CXND          - ----------------------------------------------------------------   -
Dipodomys ordii CXND           250 AIGI-PQQPEA-KL-LF--DT-QEN--------------------------------------- 268
Oryctolagus cuniculus CXND     250 TTGT-PQQPEA-KL-LC--DT-QVG--------------------------------------- 268
Tursiops truncatus CXND        250 AMST-AQGGET-KV-LC--GT-QEA--------------------------------------- 268
Bos taurus CXND                168 ATSI-AQGGET-KL-LC--DT-QEA--------------------------------------- 186
Equus caballus CXND            269 AIST-PQRGET-KL-LC--DT-HKS--------------------------------------- 287
Canis lupus familiaris CXND    250 AIGA-SQRGET-KP-LF----------------------------------------------- 263
Myotis lucifugus CXND          250 AIST-TRESET-KL-LC--NT-L----------------------------------------- 266
Erinaceus europaeus CXND       251 VISA-TQQGET-KL-QS--DT-FKD--------------------------------------- 269
Dasypus novemcinctus CXND      250 TLST-PQQPGA-KL-LC--DT-QEG--------------------------------------- 268
Choloepus hoffmanni CXND       250 IKST-HQQPEA-KL-LY--DT-REG--------------------------------------- 268
Loxodonta africana CXND        250 AIST-AQQPEA-KL-LC--DT------------------------------------------- 265
Homo sapiens CXNE              225 -----------PV--------------------------------------------------- 226
Pan troglodytes CXNE           225 -----------PV--------------------------------------------------- 226
Pongo abelii CXNE              225 -----------PV--------------------------------------------------- 226
Macaca mulatta CXNE            225 -----------PV--------------------------------------------------- 226
Papio hamadryas CXNE           225 -----------PV--------------------------------------------------- 226
Callithrix jacchus CXNE        225 -----------PV--------------------------------------------------- 226
Otolemur garnettii CXNE        225 -----------PV--------------------------------------------------- 226
Mus musculus Cxne              238 -----------PV--------------------------------------------------- 239
```

```
Rattus norvegicus Cxne        244 -----------PV---------------------------------------------------- 245
Dipodomys ordii CXNE          244 -----------PV---------------------------------------------------- 245
Cavia porcellus CXNE          272 -----------PV---------------------------------------------------- 273
Oryctolagus cuniculus CXNE    244 -----------PA---------------------------------------------------- 245
Equus caballus CXNE           225 -----------PV---------------------------------------------------- 226
Canis lupus familiaris CXNE   225 -----------PV---------------------------------------------------- 226
Felis catus CXNE              225 -----------PV---------------------------------------------------- 226
Myotis lucifugus CXNE         225 -----------PV---------------------------------------------------- 226
Sorex araneus CXNE            225 -----------PV---------------------------------------------------- 226
Dasypus novemcinctus CXNE     225 -----------PV---------------------------------------------------- 226
Loxodonta africana CXNE       225 -----------PV---------------------------------------------------- 226
Homo sapiens CXNF             252 G-QN-AITGF-PS---------------------------------------------------- 261
Pan troglodytes CXNF          252 G-QN-AITGF-PS---------------------------------------------------- 261
Gorilla gorilla CXNF          252 G-QN-AITGF-PS---------------------------------------------------- 261
Nomascus leucogenys CXNF      252 G-QN-AITGF-PS---------------------------------------------------- 261
Callithrix jacchus CXNF       252 G-QN-AITGF-PS---------------------------------------------------- 261
Mus musculus Cxnf             252 G-QN-AITSF-PS---------------------------------------------------- 261
Rattus norvegicus Cxnf        252 G-QN-AITSF-PS---------------------------------------------------- 261
S.tridecemlineatus CXNF       252 G-QN-AITGF-PS---------------------------------------------------- 261
Oryctolagus cuniculus CXNF    252 G-QN-AITGF-PS---------------------------------------------------- 261
Ochotona princeps CXNF        252 G-QN-AITGF-PS---------------------------------------------------- 261
Bos taurus CXNF               252 G-QN-AITGF-PS---------------------------------------------------- 261
Equus caballus CXNF           252 G-QN-ATTGF-PS---------------------------------------------------- 261
Canis lupus familiaris CXNF   252 G-QN-AITGF-PS---------------------------------------------------- 261
Myotis lucifugus CXNF         252 G-QN-ATTGF-PS---------------------------------------------------- 261
Dasypus novemcinctus CXNF     252 G-QN-AVTGF-PS---------------------------------------------------- 261
Choloepus hoffmanni CXNF      252 G-QN-AITGF-PS---------------------------------------------------- 261
Loxodonta africana CXNF       252 G-QN-ASTGF-PS---------------------------------------------------- 261
Homo sapiens CXNG             259 L-KD-ILRRS-PGTGAGLAEK-SDRCSAC------------------------------------ 283
Pan troglodytes CXNG          259 L-KD-ILRRS-PGTGAGLAEK-SDRCSAC------------------------------------ 283
Gorilla gorilla CXNG          259 L-KD-ILRRS-PGTGAGLAEK-SDRCSAC------------------------------------ 283
Pongo abelii CXNG             259 L-KD-ILRRS-PGTGAGLAEK-SDRCSAC------------------------------------ 283
Nomascus leucogenys CXNG      259 L-KD-ILRRS-PGTGAGLAEK-SDRCSAC------------------------------------ 283
Macaca mulatta CXNG           259 L-KD-ILRRS-PGTGAGLAEK-SDRCSAC------------------------------------ 283
Papio hamadryas CXNG          259 L-KD-ILRRS-PGTGAGLAEK-SDRCSAC------------------------------------ 283
Callithrix jacchus CXNG       259 L-KD-ILRRS-PGTGAGLAEK-SDRCSAC------------------------------------ 283
Microcebus murinus CXNG       259 L-KD-ILRRS-PGSGAGLAEK-SDRCSAC------------------------------------ 283
Mus musculus Cxng             259 L-KD-ILRRS-PGTGAGLAEK-SDRCSAC------------------------------------ 283
Rattus norvegicus Cxng        259 L-KD-ILRRS-PGTGAGLAEK-SDRCSAC------------------------------------ 283
Oryctolagus cuniculus CXNG    259 L-KD-ILRRS-PGTGAGLAEK-SDRCSAC------------------------------------ 283
Bos taurus CXNG               260 L-KD-ILRRS-PGTGAGLAEK-SDRCSAC------------------------------------ 284
Equus caballus CXNG           259 L-KD-ILRRS-PGTGAGLAEK-SDRCSAC------------------------------------ 283
Canis lupus familiaris CXNG   259 L-KD-ILRRS-PGTGAGLAEK-SDRCSAC------------------------------------ 283
Felis catus CXNG              259 L-KD-ILRRS-PGTGAGLAEK-SDRCSAC------------------------------------ 283
Myotis lucifugus CXNG         259 L-KD-ILRRS-PGTGAGLAEK-SDRCSAC------------------------------------ 283
Pteropus vampyrus CXNG        259 L-KD-ILRRR-PSTGAGMADK-SDRCSAC------------------------------------ 283
Dasypus novemcinctus CXNG     259 L-KD-ILRRS-PGTGAGLAEK-SDRCSAC------------------------------------ 283
Loxodonta africana CXNG       259 L-KD-ILRRS-PGTGAGLAEK-SDRCSAC------------------------------------ 283
Procavia capensis CXNG        259 L-KD-ILRRS-PGTGAGLTEK-SDRCSAC------------------------------------ 283
Homo sapiens CXNH1            284 --LS------------------------------------------------------------- 285
Pan troglodytes CXNH1         284 --LS------------------------------------------------------------- 285
Gorilla gorilla CXNH1         284 --LS------------------------------------------------------------- 285
Pongo abelii CXNH1            284 --LS------------------------------------------------------------- 285
Nomascus leucogenys CXNH1     284 --LS------------------------------------------------------------- 285
Macaca mulatta CXNH1          284 --LS------------------------------------------------------------- 285
Otolemur garnettii CXNH1      282 --FS------------------------------------------------------------- 283
Mus musculus Cxnh1            284 --LS------------------------------------------------------------- 285
Rattus norvegicus Cxnh1       284 --LS------------------------------------------------------------- 285
Cavia porcellus CXNH1         284 --LS------------------------------------------------------------- 285
Oryctolagus cuniculus CXNH1   284 --LS------------------------------------------------------------- 285
Ochotona princeps CXNH1       298 --LS------------------------------------------------------------- 299
Equus caballus CXNH1          284 --LS------------------------------------------------------------- 285
Canis lupus familiaris CXNH1  284 --LS------------------------------------------------------------- 285
Felis catus CXNH1             284 --LS------------------------------------------------------------- 285
Myotis lucifugus CXNH1        315 --LS------------------------------------------------------------- 316
Dasypus novemcinctus CXNH1    284 --LS------------------------------------------------------------- 285
Loxodonta africana CXNH1      284 --LS------------------------------------------------------------- 285
Homo sapiens CXNI             275 P-GG-KF--FNP-F--------------------------------------------------- 283
Pan troglodytes CXNI          275 P-GG-KF--FNP-F--------------------------------------------------- 283
Pongo abelii CXNI             275 P-GG-KF--FNP-F--------------------------------------------------- 283
Nomascus leucogenys CXNI      275 P-GG-KF--FNP-F--------------------------------------------------- 283
Macaca mulatta CXNI           275 P-GG-KF--FNP-F--------------------------------------------------- 283
Papio hamadryas CXNI          275 P-GG-KF--FNP-F--------------------------------------------------- 283
Callithrix jacchus CXNI       275 P-GG-KF--FHS-F--------------------------------------------------- 283
Otolemur garnettii CXNI       274 P-GG-KF--FSP-F--------------------------------------------------- 282
Mus musculus Cxni             275 S-GE-KF--FSD-F--------------------------------------------------- 283
Rattus norvegicus Cxni        307 P-DE-KF--FSD-F--------------------------------------------------- 315
Cavia porcellus CXNI          275 P-GG-KF--FSP-F--------------------------------------------------- 283
Oryctolagus cuniculus CXNI    275 P-GG-RF--FST-F--------------------------------------------------- 283
```

Bos taurus CXNI	276	P-GG-KF--FSP-F--	284
Equus caballus CXNI	275	P-GG-KF--FNP-F--	283
Canis lupus familiaris CXNI	275	P-GG-KF--FNP-F--	283
Felis catus CXNI	275	P-GG-KF--FNP-F--	283
Pteropus vampyrus CXNI	275	P-GG-KF--FNP-F--	283
Erinaceus europaeus CXNI	276	P-GN-KF--FQP-F--	284
Dasypus novemcinctus CXNI	275	P-GE-KF--FNP-F--	283
Loxodonta africana CXNI	275	P-EG-KF--FNP-F--	283
Homo sapiens CXNJ1	265	A-IG-----FPP-YYAHTA--APLG-QA-RAVGYPGAPPPAADFKM--LALTEA--RGKGQSAKL	314
Pongo abelii CXNJ1	265	A-IG-----FPP-YYAHTA--APLG-QA-RAVGYLGAPPPAADFKM--LALTEA--RGKGQSAKL	314
Macaca mulatta CXNJ1	263	A-LG-----FPP-YYAHTA---PAG-QA-RALGYPGAPPPAADFKL--LALTEA--RGRCPSAQL	311
Mus musculus Cxnj1	271	S-IG-----FPP-YYTHPA--CPTV-QA-KAIGFPGAPLSPADFTV--VTLNDA--QGRNHPVKH	320
Rattus norvegicus Cxnj1	271	S-IG-----LPP-YYTHPA--CPTV-QA-KATGFPGAPLLPADFTV--VTLNDA--QGRGHPVKH	320
Bos taurus CXNJ1	266	T-IG-----FPP-YYAPSA--SSLG-QA-SAPGYPEPPPPAA--LP--GT-PGT--PGGG-----	307
Equus caballus CXNJ1	277	T-IG-----FPP-YYTHSA--SSLG-QA-MATGYPGAPPPPTDFKM--VALSEA--HRNGHPANF	326
Myotis lucifugus CXNJ1	-	---	-
Myotis lucifugus CXNJ2	-	---	-
Pteropus vampyrus CXNJ1	434	T-GA-----CPP-YYTQSA--SSLG-QA-MSRAYPGAPLPATDVTK--EALSEA--QGKGHPDKF	483
Pteropus vampyrus CXNJ2	-	---	-
Sorex araneus CXNJ1	-	---	-
***Homo sapiens* CXNK1**	276	P-LS-PM--SLP-GY-KLV----NG-D--R-----------------------------------	292
***Homo sapiens* CXNK2**	277	P-LS-PM--SPP-GY-KLV----TG-D--R-----------------------------------	293
***Pan troglodytes* CXNK1**	276	P-LS-PM--SLP-GY-KLV----TG-D--R-----------------------------------	292
***Pan troglodytes* CXNK2**	277	P-LS-PM--SPP-GY-KLV----TG-D--R-----------------------------------	293
***Pongo abelii* CXNK1**	277	P-LS-PM--SPP-GY-KLV----TG-D--R-----------------------------------	293
***Nomascus leucogenys* CXNK1**	277	P-LS-PM--SPP-GY-KLV----TG-D--R-----------------------------------	293
***Callithrix jacchus* CXNK1**	277	P-LS-PM--SPP-GY-KLV----TG-D--R-----------------------------------	293
***Mus musculus* Cxnk1**	277	P-LS-PM--SPP-GY-KLV----TG-D--R-----------------------------------	293
***Mus musculus* Cxnk2**	260	S-ST-VLT-TID-SS-EQA----VP-V--E-----------------------------------	277
***Rattus norvegicus* Cxnk1**	277	P-LS-PM--SPP-GY-KLV----TG-D--R-----------------------------------	293
***Rattus norvegicus* Cxnk2**	266	V-LT-TI--	270
***Cavia porcellus* CXNK1**	277	P-LS-PM--SPP-GY-KLV----TG-D--R-----------------------------------	293
***Oryctolagus cuniculus* CXNK1**	277	P-LS-PM--SPP-GY-KLV----TG-D--R-----------------------------------	293
***Bos taurus* CXNK1**	278	P-LS-PM--SPP-GY-KLV----TG-D--R-----------------------------------	294
***Vicugna pacos* CXNK1**	277	P-LS-PM--SPP-GY-KLV----TG-D--R-----------------------------------	293
***Equus caballus* CXNK1**	277	P-LS-PM--SPP-GY-KLV----TG-D--R-----------------------------------	293
***Equus caballus* CXNK2**	243	P-LS-TT--SPP-RD-KLV----LG-D--R-----------------------------------	259
***Canis lupus familiaris* CXNK1**	277	P-LS-PM--SPP-GY-KLV----TG-D--R-----------------------------------	293
***Canis lupus familiaris* CXNK2**	269	P-FS-AM--SFH-KD-KMV----PG-N--Q-----------------------------------	285
***Felis catus* CXNK1**	264	A-LS-DM--SVP-RN-KLV----PG-N--A-----------------------------------	280
***Myotis lucifugus* CXNK1**	277	P-LS-PM--SPP-GY-KLV----TG-D--R-----------------------------------	293
***Dasypus novemcinctus* CXNK1**	-	---	-
***Dasypus novemcinctus* CXNK2**	277	P-LS-PM--SPP-GY-KLV----TG-D--R-----------------------------------	293
***Loxodonta africana* CXNK1**	277	P-LS-PM--SPP-GY-KLV----TG-D--R-----------------------------------	293
***Loxodonta africana* CXNK2**	277	S-LL-PL--SPP-RD-K-V----TG-D--R-----------------------------------	292
Homo sapiens CXNL	274	I-VS-HY--FPL-TEVGMVETSPL--PA-K-------PFNQFEEKISTGPLGDL-SRGYQETLPS	322
Pan troglodytes CXNL	274	I-VS-HY--FPL-TEVGMVETSPL--PA-K-------PFNQFEEKISTGPLGDL-SRGYQETLPS	322
Pongo abelii CXNL	274	I-VS-HY--FPL-TEVGMVETSPL--PA-K-------PFNQFEEKISTGPLGDL-SRGYQETLPS	322
Nomascus leucogenys CXNL	274	I-VS-HY--FPL-TEVGMVETTPL--SA-K-------PFNQFEEKISTGPLGDL-SRGYQETLPS	322
Macaca mulatta CXNL	280	I-VS-HY--FPL-TEVGMVET------A-K-------PFSQFEEKITTGPPEDL-SRGYQETLPS	324
Callithrix jacchus CXNL	278	I-VS-HY--FPL-TEVGMVETSPV--SA-K-------PFNQFEEKISTGPLGDL-SRGYQETLPS	326
Otolemur garnettii CXNL	325	I-VS-HY--FPL-SEVGMVETSPL--SA-K-------PFSQFEEKVSTGPLGDL-SRGYQETLPS	373
Mus musculus Cxnl	281	I-VS-HY--FPL-TEVGMVETSPL--SA-K-------PFSQFEEKIGTGPLADM-SRSYQETLPS	329
Rattus norvegicus Cxnl	281	I-VS-HY--FPL-TEVGMVETSPL--SA-K-------PFSQFEEKIGTGPLADM-SRGYQETLPS	329
Cavia porcellus CXNL	281	I-VS-HY--FPL-TEVGMVETSPL--SA-K-------PFSQLEEKISTGPLADV-SRGYQETLPS	329
Oryctolagus cuniculus CXNL	281	I-VS-HY--FPL-TEVGMVETSPL--SA-K-------PFSQFEEKISTGPLADM-PRSYQETLPS	329
Ochotona princeps CXNL	278	I-VS-HY--FPL-TEVGMVETSPL--SA-K-------PFGQFEEKIGSGSLAEM-PRGYQETLPS	326
Bos taurus CXNL	281	I-VS-HY--FPL-TEVGMVEASPL--SA-K-------PFSQFEEKVGPGPLGDL-SRAYQETLPS	329
Equus caballus CXNL	280	I-VS-HY--FPL-AEVGMVETSPL--SA-K-------PFSQFEEKMGTGPLGDL-SRAYQETLPS	328
Canis lupus familiaris CXNL	281	I-VS-HY--FPL-TEVGMVETSPL--SA-K-------PFSQFEEKMGTGPLGDL-SRAYQETLPS	329
Felis catus CXNL	281	I-VS-HY--FPL-TEVGMVETSPL--SA-K-------PFAQFEEKMGPGPLGDL-SRAYQETLPS	329
Pteropus vampyrus CXNL	281	I-VS-HY--FPL-TEVGMVETSPL--SA-K-------PFGQFEEKMGTGPLGDL-SRAYQETLPS	329
Sorex araneus CXNL	250	I-VS-HY--FPL-SEVGTAETSPL--SP-A-------PCTQLEAKVGPDALGDL-SQACQETLP-	297
Dasypus novemcinctus CXNL	283	I-VS-HY--FPL-TEVGMVETTPL--SA-K-------PFHQFEEKIGTGPLGDL-SRAYQETLPS	331
Loxodonta africana CXNL	280	I-VS-HY--FPL-TEVGMVETSPL--SA-K-------PFSQFEEKIGTGPLGDL-SQVYQETLPS	328
Homo sapiens CXNM	275	LKRL-P---SAP-DYNLLVEKQ-TH----TAV-YP--------SLNSSS-VFQPNPDNHSVNDEK	319
Pan troglodytes CXNM	275	LKRL-P---SAP-DYNLLVEKQ-TH----TAV-YP--------SLNSSS-VFQPNPDNHSVNDEK	319
Pongo abelii CXNM	275	LKRL-P---SAP-DYNLLVEKQ-TH----TAV-YP--------SLNSFS-VFQPNPDNHSVNDEK	319
Nomascus leucogenys CXNM	275	LKRL-P---SAP-DYNLLVEKQ-TH----TAV-YP--------SLNSSS-VFQPNPDNHSVNDEK	319
Macaca mulatta CXNM	275	LKRL-P---SAP-DYNLLVEKQ-TH----TAV-YP--------SLNSSS-VFQPNPDNHSVNDEK	319
Callithrix jacchus CXNM	274	LKRL-P---SAP-DYNLLVEKQ-TH----TAV-YP--------HLNSSS-VFQPNPDNHNVNYEK	318
Tarsius syrichta CXNM	274	VKRL-P---SAP-DYNLLVEKQ-TH----TAV-YP--------NLNSSS-AFQANPDTHSVNDKK	318
Microcebus murinus CXNM	275	LKRL-P---CAP-DYNLFVEKQ-TH----TAV-YP--------SFNSSS-AFQANPDNHSVNDEK	319
Dipodomys ordii CXNM	276	LKRF-S---SVS-DDNLLVEKP-TH----TAV-HQ--------SLNSFS-VFQASPNSH------	314
Oryctolagus cuniculus CXNM	269	PKRL-P---PAP-GH-LSAEKQ-TH----RAV-HP--------SLNPSP-PFQADPGSHSVNDQQ	312
Equus caballus CXNM	275	LKQP-S---SAS-DYNLLVEKQ-TH----TAV-HP--------SLNS-S-AFQADPDNHSGNDEK	318
Canis lupus familiaris CXNM	275	LKGF-S---SAP-DYYLLMEKQ-KH----PAA-CP--------SLSS-P-AFQADPDNHSGNDEK	318
Pteropus vampyrus CXNM	272	LKQL-S---SAP-DYNLLVEKQ-TH----TAV-YP--------SLNL-S-AFQADPDNHKGNDEK	315
Loxodonta africana CXNM	275	LKGL-P---SAP-DYNLLVEKK-TH----KTV-YP--------SFNSSS-ALQVDPDSYSGNDEK	319

Homo sapiens CXNN	278	ERIS-PL--QA---NN---QQQ-VI----RVN-VP-------KSKTMWQ-IPQ--PRQLEV-DPS	316
Pan troglodytes CXNN	278	ERIS-PL--QA---NN---QQQ-VI----RVN-VP-------KSKTMWQ-IPQ--PRQLEV-DPS	316
Pongo abelii CXNN	278	ERIS-PL--QA---NN---QQQ-VI----RVN-VP-------KSKTMWQ-IPQ--PRQLEV-DPS	316
Nomascus leucogenys CXNN	278	ERIS-PL--QA---NN---QQQ-VI----RVN-VP-------KSKTMWQ-TPQ--PRQLEV-DPS	316
Macaca mulatta CXNN	278	ETIS-PL--QA---NN---QQQ-VI----RVN-VP-------KSKTMWQ-IPQ--PRQLEV-DPS	316
Callithrix jacchus CXNN	279	ERIS-PL--QA---NN---QQQ-VI----RVN-IP-------KSKTMWQ-IPQ--PRQLEV-DPS	317
Microcebus murinus CXNN	278	ERIS-LL--QA---NN---QQQ-VI----QVN-VP-------KSKSMWQ-IPQ--PRQLEV-DPS	316
Mus musculus Cxnn	278	ERIS-LL--QA---NN---KQQ-VI----RVN-IP-------RSKSMWQ-IPH--PRQLEV-DVS	316
Rattus norvegicus Cxnn	278	ERVS-LL--QA---NN---KQQ-VI----RVN-IP-------RSKSVWQ-IPH--PRQLEV-DVS	316
Oryctolagus cuniculus CXNN	308	DRIS-LL--QA---NN---QQQ-GI----RIN-VP-------KSKTMWQ-IPQ--PRQPEA-DSS	346
Bos taurus CXNN	278	EKIS-LL--QA---NN---QQQ-VI----RVN-VP-------NSKTTWH-IPQ--ARQLDV-DPC	316
Equus caballus CXNN	278	EKIS-LL--QA---NN---QQQ-VI----QVN-VP-------KSKTMWQ-IQQ--PRQLEV-DP-	315
Canis lupus familiaris CXNN	278	KRIS-LL--QA---NN---QQQ-VI----RAS-VP-------KSKTTWQ-SSE--PMPVEV-DPC	316
Sorex araneus CXNN	278	ERSS-LL--QA---NN---QQH-PV----QAS-AP-------KFNTMRQ-IPQ--PRQLEV-DAC	316
Loxodonta africana CXNN	278	DRPS-LL--QA---NN---RQK-II----RVN-MP-------KSETIWQ-IPQ--AKRLEV-VPC	316
Homo sapiens CXNO	325	AGLAC-----PP-DY-SLVVRAAER--A-RA----------------------------------	345
Papio hamadryas CXNO	327	AGLAC-----PP-DY-SLVVRATER--A-RA----------------------------------	347
Mus musculus Cxno	335	AGLAC-----PP-DY-SLVVRAAER--A-RA----------------------------------	355
Cavia porcellus CXNO	322	PGPAC-----PP-DY-SLVVRAV--	337
Homo sapiens CXNP1	-	---	-
Pan troglodytes CXNP1	-	---	-
Pongo abelii CXNP1	-	---	-
Callithrix jacchus CXNP1	-	---	-
Otolemur garnettii CXNP1	-	---	-
Tupaia belangeri CXNP1	-	---	-
Mus musculus Cxnp1	-	---	-
Rattus norvegicus Cxnp1	-	---	-
Cavia porcellus CXNP1	-	---	-
Oryctolagus cuniculus CXNP1	-	---	-
Oryctolagus cuniculus CXNP2	-	---	-
Ochotona princeps CXNP1	-	---	-
Bos taurus CXNP1	-	---	-
Equus caballus CXNP1	-	---	-
Canis lupus familiaris CXNP1	-	---	-
Felis catus CXNP1	-	---	-
Myotis lucifugus CXNP1	-	---	-
Dasypus novemcinctus CXNP1	-	---	-
Dasypus novemcinctus CXNP2	-	---	-
Dasypus novemcinctus CXNP3	-	---	-
Dasypus novemcinctus CXNP4	-	---	-
Choloepus hoffmanni CXNP1	-	---	-
Loxodonta africana CXNP1	-	---	-
Homo sapiens CXNQ	290	YNIA-----VKP-D--QI--QYTEL--S-NA----------------------------------	307
Pan troglodytes CXNQ	290	YNIA-----VKP-D--QI--QYTEL--S-NA----------------------------------	307
Pongo abelii CXNQ	290	YNIA-----VKP-D--QI--QYTEL--S-NA----------------------------------	307
Macaca mulatta CXNQ	290	YNIA-----VKP-D--QI--QYTEL--S-NA----------------------------------	307
Tupaia belangeri CXNQ	290	YNIA-----VKP-D--QI--QYTEL--S-NA----------------------------------	307
Mus musculus Cxnq	290	YNIA-----VKP-D--QI--QYTEL--S-NA----------------------------------	307
Rattus norvegicus Cxnq	290	YNIA-----VKP-D--QI--QYTEL--S-NA----------------------------------	307
Cavia porcellus CXNQ	290	YNIA-----VKP-D--HV--QYTEL--S-NA----------------------------------	307
S.tridecemlineatus CXNQ	304	YNIA-----VKP-D--QI--QYTEL--S-NA----------------------------------	321
Oryctolagus cuniculus CXNQ	290	YNIA-----VKP-D--QI--QYTEL--S-NA----------------------------------	307
Bos taurus CXNQ	290	YNIA-----VKP-D--QI--QYTEL--S-NA----------------------------------	307
Vicugna pacos CXNQ	290	YNVA-----VKP-D--QI--QYTEL--S-NA----------------------------------	307
Equus caballus CXNQ	290	YNIA-----IKP-D--QI--QYTEL--S-NA----------------------------------	307
Canis lupus familiaris CXNQ	290	YNIA-----VKP-D--QI--QYTEL--S-NA----------------------------------	307
Myotis lucifugus CXNQ	290	YNIA-----VKP-D--HI--QYTEL--S-NA----------------------------------	307
Pteropus vampyrus CXNQ	290	YNIA-----VKP-D--QI--QYTEL--S-NA----------------------------------	307
Loxodonta africana CXNQ	290	YNIV-----VKP-D--QM--QYTEL--S-NA----------------------------------	307
Homo sapiens CXNR	252	SRRP------GP-EP-CAPPAYAHP--A-PA----------------------------------	271
Pan troglodytes CXNR	251	SRRP------GP-EP-CAPPAYAHP--A-PA----------------------------------	270
Pongo abelii CXNR	252	SRRP------GP-EP-CDPPAYAHR--A-PA----------------------------------	271
Papio hamadryas CXNR	253	SRHA------GP-ES-CAPPAYAHR--A-PA----------------------------------	272
Mus musculus Cxnr	234	SQRG------DP-DP-FGPPAYAHR--S-PA----------------------------------	253
Rattus norvegicus Cxnr	234	SQRG------DP-DP-FGPPAYAHR--S-PA----------------------------------	253
Oryctolagus cuniculus CXNR	256	APGA------GP-DP-YAPPAYAHQ--A-PA----------------------------------	275
Bos taurus CXNR	242	TRRP------GT-DP-YAPPAYAHG--A-PA----------------------------------	261
Homo sapiens CXNS	-	---	-
Pan troglodytes CXNS	-	---	-
Pongo abelii CXNS	-	---	-
Nomascus leucogenys CXNS	-	---	-
Macaca mulatta CXNS	-	---	-
Papio hamadryas CXNS	-	---	-
Otolemur garnettii CXNS	-	---	-
Tupaia belangeri CXNS	-	---	-
Mus musculus Cxns	-	---	-
Rattus norvegicus Cxns	-	---	-
Cavia porcellus CXNS	-	---	-
S.tridecemlineatus CXNS	-	---	-

```
Oryctolagus cuniculus CXNS         - -----------------------------------------------------------------   -
Ochotona princeps CXNS             - -----------------------------------------------------------------   -
Bos taurus CXNS                    - -----------------------------------------------------------------   -
Equus caballus CXNS                - -----------------------------------------------------------------   -
Canis lupus familiaris CXNS        - -----------------------------------------------------------------   -
Myotis lucifugus CXNS              - -----------------------------------------------------------------   -
Dasypus novemcinctus CXNS          - -----------------------------------------------------------------   -
Loxodonta africana CXNS            - -----------------------------------------------------------------   -
Homo sapiens CXNT                  - -----------------------------------------------------------------   -
Pan troglodytes CXNT               - -----------------------------------------------------------------   -
Nomascus leucogenys CXNT           - -----------------------------------------------------------------   -
Mus musculus Cxnt                  - -----------------------------------------------------------------   -
Rattus norvegicus Cxnt             - -----------------------------------------------------------------   -
Cavia porcellus CXNT               - -----------------------------------------------------------------   -
Oryctolagus cuniculus CXNT         - -----------------------------------------------------------------   -
Bos taurus CXNT                    - -----------------------------------------------------------------   -
Vicugna pacos CXNT                 - -----------------------------------------------------------------   -
Equus caballus CXNT                - -----------------------------------------------------------------   -
Canis lupus familiaris CXNT        - -----------------------------------------------------------------   -
Myotis lucifugus CXNT              - -----------------------------------------------------------------   -
Dasypus novemcinctus CXNT          - -----------------------------------------------------------------   -
Loxodonta africana CXNT            - -----------------------------------------------------------------   -
Homo sapiens CXNU                259 EG-A----------G-S--PRRTSR--V-SG---------------------------------- 272
Pan troglodytes CXNU             259 EG-A----------G-S--PRHTSR--V-SG---------------------------------- 272
Pongo abelii CXNU                244 EG-A----------G-S--LRGTSG--V-SG---------------------------------- 257
Nomascus leucogenys CXNU         256 EG-A----------G-S--PRGTSR--V-SG---------------------------------- 269
Macaca mulatta CXNU              259 EG-A----------G-S--PRVTSR--V-SG---------------------------------- 272
Mus musculus Cxnu                240 QS-D----------N-S--NVGQAC--V-SG---------------------------------- 253
Cavia porcellus CXNU             232 QS-I----------N-N--LSGQPP--A-LG---------------------------------- 245
Bos taurus CXNU                  241 PG-A----------H-S--PAAESA--A-PG---------------------------------- 254
Equus caballus CXNU              251 EG-A----------P-S--PTGRS---V-SG---------------------------------- 263
Myotis lucifugus CXNU            257 HG-A----------H-S--PSGQWV--V-SG---------------------------------- 270
Sorex araneus CXNU               231 QG-A----------D-S--PSGQSA--V-SG---------------------------------- 244
Dasypus novemcinctus CXNU        286 EG-A-----RAH-LN-C--PSEKSG--M-SG---------------------------------- 303
Loxodonta africana CXNU          295 EG-T-----RTN-VN-S--PSEKSA--V-SG---------------------------------- 312
```

```
Homo sapiens CXNA                 - ----------------------------------------------------------------- -
                                        720       730       740       750       760       770       780
Homo sapiens CXNA                 - ----------------------------------------------------------------- -
Pan troglodytes CXNA              - ----------------------------------------------------------------- -
Gorilla gorilla CXNA              - ----------------------------------------------------------------- -
Pongo abelii CXNA                 - ----------------------------------------------------------------- -
Nomascus leucogenys CXNA          - ----------------------------------------------------------------- -
Macaca mulatta CXNA               - ----------------------------------------------------------------- -
Callithrix jacchus CXNA           - ----------------------------------------------------------------- -
Mus musculus Cxna                 - ----------------------------------------------------------------- -
Rattus norvegicus Cxna            - ----------------------------------------------------------------- -
Cavia porcellus CXNA              - ----------------------------------------------------------------- -
Ochotona princeps CXNA            - ----------------------------------------------------------------- -
Bos taurus CXNA                   - ----------------------------------------------------------------- -
Equus caballus CXNA               - ----------------------------------------------------------------- -
Canis lupus familiaris CXNA       - ----------------------------------------------------------------- -
Felis catus CXNA                  - ----------------------------------------------------------------- -
Myotis lucifugus CXNA             - ----------------------------------------------------------------- -
Dasypus novemcinctus CXNA         - ----------------------------------------------------------------- -
Loxodonta africana CXNA           - ----------------------------------------------------------------- -
Homo sapiens CXNB                 - ----------------------------------------------------------------- -
Gorilla gorilla CXNB              - ----------------------------------------------------------------- -
Nomascus leucogenys CXNB          - ----------------------------------------------------------------- -
Macaca mulatta CXNB               - ----------------------------------------------------------------- -
Callithrix jacchus CXNB           - ----------------------------------------------------------------- -
Otolemur garnettii CXNB           - ----------------------------------------------------------------- -
Mus musculus Cxnb                 - ----------------------------------------------------------------- -
Rattus norvegicus Cxnb            - ----------------------------------------------------------------- -
Dipodomys ordii CXNB              - ----------------------------------------------------------------- -
Cavia porcellus CXNB              - ----------------------------------------------------------------- -
Oryctolagus cuniculus CXNB        - ----------------------------------------------------------------- -
Ochotona princeps CXNB            - ----------------------------------------------------------------- -
Bos taurus CXNB                   - ----------------------------------------------------------------- -
Equus caballus CXNB               - ----------------------------------------------------------------- -
Canis lupus familiaris CXNB       - ----------------------------------------------------------------- -
Myotis lucifugus CXNB             - ----------------------------------------------------------------- -
Erinaceus europaeus CXNB          - ----------------------------------------------------------------- -
Loxodonta africana CXNB           - ----------------------------------------------------------------- -
Homo sapiens CXNC                 - ----------------------------------------------------------------- -
Gorilla gorilla CXNC              - ----------------------------------------------------------------- -
Pongo abelii CXNC                 - ----------------------------------------------------------------- -
Nomascus leucogenys CXNC          - ----------------------------------------------------------------- -
Callithrix jacchus CXNC           - ----------------------------------------------------------------- -
Microcebus murinus CXNC           - ----------------------------------------------------------------- -
Otolemur garnettii CXNC           - ----------------------------------------------------------------- -
Mus musculus Cxnc                 - ----------------------------------------------------------------- -
Rattus norvegicus Cxnc            - ----------------------------------------------------------------- -
Cavia porcellus CXNC              - ----------------------------------------------------------------- -
Oryctolagus cuniculus CXNC        - ----------------------------------------------------------------- -
Bos taurus CXNC                   - ----------------------------------------------------------------- -
Equus caballus CXNC               - ----------------------------------------------------------------- -
Canis lupus familiaris CXNC       - ----------------------------------------------------------------- -
Felis catus CXNC                  - ----------------------------------------------------------------- -
Myotis lucifugus CXNC             - ----------------------------------------------------------------- -
Dasypus novemcinctus CXNC         - ----------------------------------------------------------------- -
Loxodonta africana CXNC           - ----------------------------------------------------------------- -
Homo sapiens CXND                 - ----------------------------------------------------------------- -
Pan troglodytes CXND              - ----------------------------------------------------------------- -
Macaca mulatta CXND               - ----------------------------------------------------------------- -
Callithrix jacchus CXND           - ----------------------------------------------------------------- -
Dipodomys ordii CXND              - ----------------------------------------------------------------- -
Oryctolagus cuniculus CXND        - ----------------------------------------------------------------- -
Tursiops truncatus CXND           - ----------------------------------------------------------------- -
Bos taurus CXND                   - ----------------------------------------------------------------- -
Equus caballus CXND               - ----------------------------------------------------------------- -
Canis lupus familiaris CXND       - ----------------------------------------------------------------- -
Myotis lucifugus CXND             - ----------------------------------------------------------------- -
Erinaceus europaeus CXND          - ----------------------------------------------------------------- -
Dasypus novemcinctus CXND         - ----------------------------------------------------------------- -
Choloepus hoffmanni CXND          - ----------------------------------------------------------------- -
Loxodonta africana CXND           - ----------------------------------------------------------------- -
Homo sapiens CXNE                 - ----------------------------------------------------------------- -
Pan troglodytes CXNE              - ----------------------------------------------------------------- -
Pongo abelii CXNE                 - ----------------------------------------------------------------- -
Macaca mulatta CXNE               - ----------------------------------------------------------------- -
Papio hamadryas CXNE              - ----------------------------------------------------------------- -
Callithrix jacchus CXNE           - ----------------------------------------------------------------- -
Otolemur garnettii CXNE           - ----------------------------------------------------------------- -
Mus musculus Cxne                 - ----------------------------------------------------------------- -
```

```
Rattus norvegicus Cxne            -  -----------------------------------------------------------------  -
Dipodomys ordii CXNE              -  -----------------------------------------------------------------  -
Cavia porcellus CXNE              -  -----------------------------------------------------------------  -
Oryctolagus cuniculus CXNE        -  -----------------------------------------------------------------  -
Equus caballus CXNE               -  -----------------------------------------------------------------  -
Canis lupus familiaris CXNE       -  -----------------------------------------------------------------  -
Felis catus CXNE                  -  -----------------------------------------------------------------  -
Myotis lucifugus CXNE             -  -----------------------------------------------------------------  -
Sorex araneus CXNE                -  -----------------------------------------------------------------  -
Dasypus novemcinctus CXNE         -  -----------------------------------------------------------------  -
Loxodonta africana CXNE           -  -----------------------------------------------------------------  -
Homo sapiens CXNF                 -  -----------------------------------------------------------------  -
Pan troglodytes CXNF              -  -----------------------------------------------------------------  -
Gorilla gorilla CXNF              -  -----------------------------------------------------------------  -
Nomascus leucogenys CXNF          -  -----------------------------------------------------------------  -
Callithrix jacchus CXNF           -  -----------------------------------------------------------------  -
Mus musculus Cxnf                 -  -----------------------------------------------------------------  -
Rattus norvegicus Cxnf            -  -----------------------------------------------------------------  -
S.tridecemlineatus CXNF           -  -----------------------------------------------------------------  -
Oryctolagus cuniculus CXNF        -  -----------------------------------------------------------------  -
Ochotona princeps CXNF            -  -----------------------------------------------------------------  -
Bos taurus CXNF                   -  -----------------------------------------------------------------  -
Equus caballus CXNF               -  -----------------------------------------------------------------  -
Canis lupus familiaris CXNF       -  -----------------------------------------------------------------  -
Myotis lucifugus CXNF             -  -----------------------------------------------------------------  -
Dasypus novemcinctus CXNF         -  -----------------------------------------------------------------  -
Choloepus hoffmanni CXNF          -  -----------------------------------------------------------------  -
Loxodonta africana CXNF           -  -----------------------------------------------------------------  -
Homo sapiens CXNG                 -  -----------------------------------------------------------------  -
Pan troglodytes CXNG              -  -----------------------------------------------------------------  -
Gorilla gorilla CXNG              -  -----------------------------------------------------------------  -
Pongo abelii CXNG                 -  -----------------------------------------------------------------  -
Nomascus leucogenys CXNG          -  -----------------------------------------------------------------  -
Macaca mulatta CXNG               -  -----------------------------------------------------------------  -
Papio hamadryas CXNG              -  -----------------------------------------------------------------  -
Callithrix jacchus CXNG           -  -----------------------------------------------------------------  -
Microcebus murinus CXNG           -  -----------------------------------------------------------------  -
Mus musculus Cxng                 -  -----------------------------------------------------------------  -
Rattus norvegicus Cxng            -  -----------------------------------------------------------------  -
Oryctolagus cuniculus CXNG        -  -----------------------------------------------------------------  -
Bos taurus CXNG                   -  -----------------------------------------------------------------  -
Equus caballus CXNG               -  -----------------------------------------------------------------  -
Canis lupus familiaris CXNG       -  -----------------------------------------------------------------  -
Felis catus CXNG                  -  -----------------------------------------------------------------  -
Myotis lucifugus CXNG             -  -----------------------------------------------------------------  -
Pteropus vampyrus CXNG            -  -----------------------------------------------------------------  -
Dasypus novemcinctus CXNG         -  -----------------------------------------------------------------  -
Loxodonta africana CXNG           -  -----------------------------------------------------------------  -
Procavia capensis CXNG            -  -----------------------------------------------------------------  -
Homo sapiens CXNH1              286  -----------------------------------------------SS-EQNWANLTTEER---  299
Pan troglodytes CXNH1           286  -----------------------------------------------SS-EQNWANLTTEER---  299
Gorilla gorilla CXNH1           286  -----------------------------------------------SS-EQNWANLTTEER---  299
Pongo abelii CXNH1              286  -----------------------------------------------SS-EQNWANLTTEER---  299
Nomascus leucogenys CXNH1       286  -----------------------------------------------SS-EQNWANLTTEER---  299
Macaca mulatta CXNH1            286  -----------------------------------------------SS-EQNWANLTTEER---  299
Otolemur garnettii CXNH1        284  -----------------------------------------------SS-EENRANLTTEER---  297
Mus musculus Cxnh1              286  -----------------------------------------------ST-EQNWANLTTEER---  299
Rattus norvegicus Cxnh1         286  -----------------------------------------------ST-EQNWANLTTEER---  299
Cavia porcellus CXNH1           286  -----------------------------------------------SS-EQNWANLTTEER---  299
Oryctolagus cuniculus CXNH1     286  -----------------------------------------------SS-EQNWANLTTEER---  299
Ochotona princeps CXNH1         300  -----------------------------------------------SS-EQNWANLTTEER---  313
Equus caballus CXNH1            286  -----------------------------------------------SD-EQIWANLTTEEG---  299
Canis lupus familiaris CXNH1    286  -----------------------------------------------SS-EQNWANLTTEER---  299
Felis catus CXNH1               286  -----------------------------------------------SS-EQNWANLTTEER---  299
Myotis lucifugus CXNH1          317  -----------------------------------------------SS-EQNWANLTTEER---  330
Dasypus novemcinctus CXNH1      286  -----------------------------------------------SS-EQNWANLTAEER---  299
Loxodonta africana CXNH1        286  -----------------------------------------------SS-EQNWANLTTEER---  299
Homo sapiens CXNI               284  -------------------------------------------SNNMAS-QQ--------NTD--  294
Pan troglodytes CXNI            284  -------------------------------------------SNNMAS-QQ--------NTD--  294
Pongo abelii CXNI               284  -------------------------------------------SNNMAS-QQ--------NTD--  294
Nomascus leucogenys CXNI        284  -------------------------------------------NNNMAS-QQ--------NTD--  294
Macaca mulatta CXNI             284  -------------------------------------------SNNMAS-QQ--------NTD--  294
Papio hamadryas CXNI            284  -------------------------------------------SNNMAS-QQ--------NTD--  294
Callithrix jacchus CXNI         284  -------------------------------------------SNNMAS-QQ--------NTD--  294
Otolemur garnettii CXNI         283  -------------------------------------------SNNMAS-QQ--------NTD--  293
Mus musculus Cxni               284  -------------------------------------------SNNMGS-RK--------NPD--  294
Rattus norvegicus Cxni          316  -------------------------------------------SNNMGS-RK--------NPD--  326
Cavia porcellus CXNI            284  -------------------------------------------SNNTVS-QQ--------NTD--  294
Oryctolagus cuniculus CXNI      284  -------------------------------------------STNTAS-QQ--------NTD--  294
```

```
Bos taurus CXNI                285 -------------------------------------------SNKMAS-QQ--------NTD-- 295
Equus caballus CXNI            284 -------------------------------------------SNKMAS-QQ--------NID-- 294
Canis lupus familiaris CXNI    284 -------------------------------------------SNKMAS-QQ--------NTD-- 294
Felis catus CXNI               284 -------------------------------------------SNKMAS-QQ--------NTD-- 294
Pteropus vampyrus CXNI         284 -------------------------------------------SNQVAS-QQ--------NTD-- 294
Erinaceus europaeus CXNI       285 -------------------------------------------SHKLAS-QQ--------NTD-- 295
Dasypus novemcinctus CXNI      284 -------------------------------------------SNKMAS-QQ--------NTH-- 294
Loxodonta africana CXNI        284 -------------------------------------------SNKMAS-QQ--------NTD-- 294
Homo sapiens CXNJ1             315 -Y-NGHHHLLM-TEQNWANQAAERQPPALKAYP-AAST-PAAPSPVGSS-SP-PL---AHEAE-- 367
Pongo abelii CXNJ1             315 -Y-NGHHHLLM-TEQNWANQAAEQQPPALKAYP-AAST-PAAPSPVGSS-SP-PL---AHEAE-- 367
Macaca mulatta CXNJ1           312 -Y-HGHHHLPL-TEQNWAGRAAEQQRHELRAYP-AAPT-PAAPSPTGSS-SQ-PP---THEAE-- 364
Mus musculus Cxnj1             321 -C-NG-HHL-T-TEQNWTRQVAEQQTPASK------PS-SAASSPDGRK---------------- 357
Rattus norvegicus Cxnj1        321 -C-NG-HHL-T-TEQNWASLGAEPQTPASK------PS-SAASSPHGRK---------------- 357
Bos taurus CXNJ1               308 ----GNQGLRA-RAQNWANREAEPQTSSRKASP-PAPTPPAAESPGG------------------ 348
Equus caballus CXNJ1           327 -C-NGNHHLLM-TEQNWANQEAEQQRSVRKASL-PAST-SASLTPPGSP-QQ-LP--QLGGAG-- 380
Myotis lucifugus CXNJ1           - -----------------------------------------------------------------   -
Myotis lucifugus CXNJ2           - -----------------------------------------------------------------   -
Pteropus vampyrus CXNJ1        484 -Y-DSSQHLLT-AEQNWATQEAEQQLSVRKATP-PPVSPSSSLTPPGSP-QP-PP--QE------ 534
Pteropus vampyrus CXNJ2          - -----------------------------------------------------------------   -
Sorex araneus CXNJ1              - -----------------------------------------------------------------   -
Homo sapiens CXNK1             293 --------------------------------------------NN-SSCRN-YDK-QASEQNWA 310
Homo sapiens CXNK2             294 --------------------------------------------NN-SSCRN-YNK-QASEQNWA 311
Pan troglodytes CXNK1          293 --------------------------------------------NN-SSCRN-YDK-QASEQNWA 310
Pan troglodytes CXNK2          294 --------------------------------------------NN-SSCRN-YNK-QASEQNWA 311
Pongo abelii CXNK1             294 --------------------------------------------NN-SSCRN-YNK-QASEQNWA 311
Nomascus leucogenys CXNK1      294 --------------------------------------------NN-SSCRN-YNK-QASEQNWA 311
Callithrix jacchus CXNK1       294 --------------------------------------------NN-SSCRN-YNK-QASEQNWA 311
Mus musculus Cxnk1             294 --------------------------------------------NN-SSCRN-YNK-QASEQNWA 311
Mus musculus Cxnk2             278 --------------------------------------------LS-SVCI-------------- 283
Rattus norvegicus Cxnk1        294 --------------------------------------------NN-SSCRN-YNK-QASEQNWA 311
Rattus norvegicus Cxnk2        271 -------------------------------------------------CSS-DQV-VPVGLS-S 283
Cavia porcellus CXNK1          294 --------------------------------------------NN-SSCRN-YNK-QASEQNWA 311
Oryctolagus cuniculus CXNK1    294 --------------------------------------------NN-SSCRN-YNK-QASEQNWA 311
Bos taurus CXNK1               295 --------------------------------------------NN-SSCRN-YNK-QASEQNWA 312
Vicugna pacos CXNK1            294 --------------------------------------------NN-SSCRN-YNK-QASEQNWA 311
Equus caballus CXNK1           294 --------------------------------------------NN-SSCRN-YNK-QASEQNWA 311
Equus caballus CXNK2           260 --------------------------------------------NS-SSCRN-YNK-QGSEQNRA 277
Canis lupus familiaris CXNK1   294 --------------------------------------------NN-SSCRN-YNK-QASEQNWA 311
Canis lupus familiaris CXNK2   286 --------------------------------------------NS-SSYRS-YNK-QGNEQNCA 303
Felis catus CXNK1              281 --------------------------------------------NS-SSCLS-YSK-QGNEQNRA 298
Myotis lucifugus CXNK1         294 --------------------------------------------NN-SSCRN-YNK-QASEQNWA 311
Dasypus novemcinctus CXNK1       - -----------------------------------------------------------------   -
Dasypus novemcinctus CXNK2     294 --------------------------------------------NN-SSCRN-YNK-QASEQNWA 311
Loxodonta africana CXNK1       294 --------------------------------------------NN-SSCRN-YNK-QASEQNWA 311
Loxodonta africana CXNK2       293 --------------------------------------------SS-SSCYS-YDK-QGSDQNCD 310
Homo sapiens CXNL              323 -YAQVGAQEVE-GEG---PPAEEGAEPEV-----------------GEK-KEEAERLTTEEQE-- 362
Pan troglodytes CXNL           323 -YAQVGAQEVE-GEG---PPAEEGAEPEV-----------------GEK-KEEAERLTTEEQE-- 362
Pongo abelii CXNL              323 -YAQVGAQEVE-GGG---PPAEEGAEPEV-----------------GEK-KQEAERLTTEEQE-- 362
Nomascus leucogenys CXNL       323 -YAQVGAQEVE-GEG---PPAEEGAEPEV-----------------GEK-QQEAERLTTEEQE-- 362
Macaca mulatta CXNL            325 -YAQVGAQEVE-GEG---PPAEEGAEPEV-----------------GEK-KPEEERLTMEEQE-- 364
Callithrix jacchus CXNL        327 -YAQVGVQEVE-GEA---LPAEEGAEPEV-----------------GEK--QEAERVTTEEQE-- 365
Otolemur garnettii CXNL        374 -YAQVGAQEAE-GEG---QPAEEGAEPEA-----------------GDK-RLEAEKATAEGLE-- 413
Mus musculus Cxnl              330 -YAQVGVQEVE-REE---PPIEEAVEPEV-----------------GEK-KQEAEKVAPEGQE-- 369
Rattus norvegicus Cxnl         330 -YAQVGAQEVE-REE---QPVEEAVEPEV-----------------GEK-KQEAEKVAPEGQE-- 369
Cavia porcellus CXNL           330 -YAQVGAQEVE-GEE---QPVEEAAEPEM-----------------EEK-KPEAEKVTLEVQE-- 369
Oryctolagus cuniculus CXNL     330 -YAQVGAPEVE-GEE---PPVEEAAEPEV-----------------GEK-RPEAEKVTAEGPE-- 369
Ochotona princeps CXNL         327 -YAQVGVPEVE-AKE---PAVEEAAEPEV-----------------GEK-KPEAEKVTLEAAE-- 366
Bos taurus CXNL               330 -YAQVGAQEGV-EEE---QPVEAAAEPEV-----------------GEK-SQEAERVSTEGQE-- 369
Equus caballus CXNL           329 -YAQVGAQEVE-GEGE-EQPVEEAAEPEA-----------------GEK-SQEAEKVSTEGQE-- 370
Canis lupus familiaris CXNL   330 -YAQVGAQEGE-GEE---QPVEEGAEPEV-----------------GDK-RQEAETVSTEGQE-- 369
Felis catus CXNL              330 -YAQVGAQEGE-SGE---QPVEEGAEPEV-----------------GDK-RQEAERVSTEGQE-- 369
Pteropus vampyrus CXNL        330 -YAQVGAQEVE-AEE---PPAEEGAEAEV-----------------AEK-RQEADRVSTEGQE-- 369
Sorex araneus CXNL            298 ------LQE-D-RDK---Q-AEPRGAGRA-----------------EEE-PGEGPEV-------- 324
Dasypus novemcinctus CXNL     332 -YAQVGAQEVE-GEE---QHVEAGAEPEL-----------------GEK-RQEAERVSTEGQE-- 371
Loxodonta africana CXNL       329 -YAQVGAPEVE-GEE---QPVEEGAEPEV-----------------EEK-RQEAERVTTDGQE-- 368
Homo sapiens CXNM             320 CIL--DEQETVLSNE---------IS-TLS-TSC--SHFQH---ISSNN-NKD-TH-KIFGKE-L 362
Pan troglodytes CXNM          320 CIL--DEQETVLSNE---------IS-TLS-TSC--SHFQH---ISSNN-NKD-TH-KIFGKE-L 362
Pongo abelii CXNM             320 CIL--DEQETVLSNE---------IS-TLS-TSC--SHFQH---ISSNN-NKD-TH-KIFGKE-V 362
Nomascus leucogenys CXNM      320 CIL--DEQETVLSNE---------IS-TLS-TSC--SHFQH---ISSNS-NKD-TH-KIFGKE-V 362
Macaca mulatta CXNM           320 CIL--NEQETVLSNE---------IS-TLS-TSC--THFQH---IGSNN-NKD-TH-KIFGKE-V 362
Callithrix jacchus CXNM       319 CIL--DEQETILSNE---------IC-TLS-TSC--SHFQH---ISSNN-NKD-TH-KIFGKE-V 361
Tarsius syrichta CXNM         319 RST--DEQETTVS-E---------IC-TVS-TTC--RHFQD---IGSNN--KE-TH-KICGK--V 358
Microcebus murinus CXNM       320 CIL--DDQETVLSNE---------IC-TFS-TSC--SHLQH---IGSNS-NKD-TH-KIFGKE-V 362
Dipodomys ordii CXNM          315 -IL--DEEEN---NG---------MC-TLS-TSC--SVLHP---IDLGN-NKG-TH-KVSGKE-V 353
Oryctolagus cuniculus CXNM    313 CAL--DEPGAVFSDK---------MR-SLH-TAY--SHLQH---IRSNH-SKD-PH-KTFGKA-A 355
Equus caballus CXNM           319 CIL--DEQETAFSKE---------MC-TFS-TTC--SHLQH---IGSTN-KED-IH-KIFGKE-V 361
Canis lupus familiaris CXNM   319 CIL--DEQETVLSDE---------MR-TLS-ATC--SHLQH---ISSCN-NED-TH-KIFRRE-V 361
Pteropus vampyrus CXNM        316 CIL--DELETVLSNE---------KC-TLS-TTC--SHIQS---IGSST-NED-AH-KIFRKE-V 358
Loxodonta africana CXNM       320 CIL--DEQETVLSSK---------MH-TIS-TTG--SHLQH---INSNN--DG-TH-KISGKD-V 361
```

```
Homo sapiens CXNN                317 -NGKKDWSEKDQHSG---------QL-HVH-SPC--PWAGSA-GNQHLG-QQS-DH-SSFGLQ-- 361
Pan troglodytes CXNN             317 -NGKKDWSEKDQHSG---------QL-HVH-SPC--PWAGSA-GNQHLG-QQS-DH-SSFGLQ-- 361
Pongo abelii CXNN                317 -NGKKDWSEKDQHSG---------QL-HVH-SPC--PWAGSA-GNQHLG-QQS-DH-SSFGLQ-- 361
Nomascus leucogenys CXNN         317 -NGKKDWSEKDQHSG---------QL-HVH-SPC--PWAGSA-GNQHLG-QQS-DH-SSFGLQ-- 361
Macaca mulatta CXNN              317 -NGKKDWSEKDQHIG---------QL-HVH-SPC--PWADSA-GNQHLG-QQS-DQ-SSFGLQ-- 361
Callithrix jacchus CXNN          318 -NGKKDWSEKDQHSG---------QL-HVH-SPC--PWAGSG-GNQHLG-QQS-DY-SSFGLQ-- 362
Microcebus murinus CXNN          317 -CSKKDWAEKVQYSG---------QL-HVH-SPC--PWTGNS-RIQHPG-QQP-DP-SAFGLQ-- 361
Mus musculus Cxnn                317 -CGKRDWAEKIESCA---------QL-HVH-SPC--PHDRSA-RIQHPG-QQP-CH-SVFGPK-- 361
Rattus norvegicus Cxnn           317 -CGKRGWAERVENSE---------QL-HVH-SPC--PHERSA-RIQHPG-QQP-CH-SIVCPK-- 361
Oryctolagus cuniculus CXNN       347 -FSKKEWAEKDQHSG---------QL-HVH-SPC--PQDAST-RIQHSG-QQP-DH-SSFAVQ-- 391
Bos taurus CXNN                  317 -YSKKDWAEKNQHNG---------QL-HVH-SPC--PWDDSA-RIQHPG-QQP-DH-SLFGLQ-- 361
Equus caballus CXNN              316 -CSKKDWPEKDQHSG---------QL-HVH-SPC--PWDDDA-RIQHLG-QQP-DH-SSFALQ-- 360
Canis lupus familiaris CXNN      317 -YVKKEWAGKDQYRG---------QL-HVH-SPC--PWDGVT-QIQHPG-QQP-DL-SS-GLE-- 360
Sorex araneus CXNN               317 -CSQEDSAEKDQHGG---------QL-HVH-EPG--LWTHSA-TLQHPG-QHS-GK--PFGPQ-- 360
Loxodonta africana CXNN          317 -YDKKDWAQKDQHSG---------QL-LVH-SPC--TWNSSV-RMQDHG-QQP-DHPSSFGFQ-- 362
Homo sapiens CXNO                346 --------------------------------------------H--DQ-NLANLALQA-LRD-- 360
Papio hamadryas CXNO             348 --------------------------------------------H--DQ-NLANLALQA-LRD-- 362
Mus musculus Cxno                356 --------------------------------------------H--DQ-NLANLALQA-LRD-- 370
Cavia porcellus CXNO             338 -----------------------------------------------DP-SLAQLGLRT-LRD-- 351
Homo sapiens CXNP1                 - -----------------------------------------------------------------   -
Pan troglodytes CXNP1              - -----------------------------------------------------------------   -
Pongo abelii CXNP1                 - -----------------------------------------------------------------   -
Callithrix jacchus CXNP1           - -----------------------------------------------------------------   -
Otolemur garnettii CXNP1           - -----------------------------------------------------------------   -
Tupaia belangeri CXNP1             - -----------------------------------------------------------------   -
Mus musculus Cxnp1                 - -----------------------------------------------------------------   -
Rattus norvegicus Cxnp1            - -----------------------------------------------------------------   -
Cavia porcellus CXNP1              - -----------------------------------------------------------------   -
Oryctolagus cuniculus CXNP1        - -----------------------------------------------------------------   -
Oryctolagus cuniculus CXNP2        - -----------------------------------------------------------------   -
Ochotona princeps CXNP1            - -----------------------------------------------------------------   -
Bos taurus CXNP1                   - -----------------------------------------------------------------   -
Equus caballus CXNP1               - -----------------------------------------------------------------   -
Canis lupus familiaris CXNP1       - -----------------------------------------------------------------   -
Felis catus CXNP1                  - -----------------------------------------------------------------   -
Myotis lucifugus CXNP1             - -----------------------------------------------------------------   -
Dasypus novemcinctus CXNP1         - -----------------------------------------------------------------   -
Dasypus novemcinctus CXNP2         - -----------------------------------------------------------------   -
Dasypus novemcinctus CXNP3         - -----------------------------------------------------------------   -
Dasypus novemcinctus CXNP4         - -----------------------------------------------------------------   -
Choloepus hoffmanni CXNP1          - -----------------------------------------------------------------   -
Loxodonta africana CXNP1           - -----------------------------------------------------------------   -
Homo sapiens CXNQ                308 --------------------------------------------K--IA-YKQNKANTA--QE-- 321
Pan troglodytes CXNQ             308 --------------------------------------------K--IA-YKQNKANTA--QE-- 321
Pongo abelii CXNQ                308 --------------------------------------------K--IA-YKQNKANTA--QE-- 321
Macaca mulatta CXNQ              308 --------------------------------------------K--IA-YKQNKANTA--QE-- 321
Tupaia belangeri CXNQ            308 --------------------------------------------K--IA-YKQNKANSA--QE-- 321
Mus musculus Cxnq                308 --------------------------------------------K--IA-YKQNKANIA--QE-- 321
Rattus norvegicus Cxnq           308 --------------------------------------------K--IA-YKQNKANIA--QE-- 321
Cavia porcellus CXNQ             308 --------------------------------------------K--IA-YRQNKANTA--QE-- 321
S.tridecemlineatus CXNQ          322 --------------------------------------------K--IA-YKQNKANIA--QE-- 335
Oryctolagus cuniculus CXNQ       308 --------------------------------------------K--IA-YKQNKANIA--QE-- 321
Bos taurus CXNQ                  308 --------------------------------------------K--IA-YKQNKANIA--QE-- 321
Vicugna pacos CXNQ               308 --------------------------------------------K--IA-YKQNKANIA--QE-- 321
Equus caballus CXNQ              308 --------------------------------------------K--IA-YKQNKANIA--QE-- 321
Canis lupus familiaris CXNQ      308 --------------------------------------------K--IA-YKQNKANIA--QE-- 321
Myotis lucifugus CXNQ            308 --------------------------------------------K--IA-YKQNKANIA--QE-- 321
Pteropus vampyrus CXNQ           308 --------------------------------------------K--IA-YKQNKANIA--QE-- 321
Loxodonta africana CXNQ          308 --------------------------------------------K--IA-YKQNKANIA--QE-- 321
Homo sapiens CXNR                  - -----------------------------------------------------------------   -
Pan troglodytes CXNR               - -----------------------------------------------------------------   -
Pongo abelii CXNR                  - -----------------------------------------------------------------   -
Papio hamadryas CXNR               - -----------------------------------------------------------------   -
Mus musculus Cxnr                  - -----------------------------------------------------------------   -
Rattus norvegicus Cxnr             - -----------------------------------------------------------------   -
Oryctolagus cuniculus CXNR         - -----------------------------------------------------------------   -
Bos taurus CXNR                    - -----------------------------------------------------------------   -
Homo sapiens CXNS                  - -----------------------------------------------------------------   -
Pan troglodytes CXNS               - -----------------------------------------------------------------   -
Pongo abelii CXNS                  - -----------------------------------------------------------------   -
Nomascus leucogenys CXNS           - -----------------------------------------------------------------   -
Macaca mulatta CXNS                - -----------------------------------------------------------------   -
Papio hamadryas CXNS               - -----------------------------------------------------------------   -
Otolemur garnettii CXNS            - -----------------------------------------------------------------   -
Tupaia belangeri CXNS              - -----------------------------------------------------------------   -
Mus musculus Cxns                  - -----------------------------------------------------------------   -
Rattus norvegicus Cxns             - -----------------------------------------------------------------   -
Cavia porcellus CXNS               - -----------------------------------------------------------------   -
S.tridecemlineatus CXNS            - -----------------------------------------------------------------   -
```

Oryctolagus cuniculus CXNS	-	---	-
Ochotona princeps CXNS	-	---	-
Bos taurus CXNS	-	---	-
Equus caballus CXNS	-	---	-
Canis lupus familiaris CXNS	-	---	-
Myotis lucifugus CXNS	-	---	-
Dasypus novemcinctus CXNS	-	---	-
Loxodonta africana CXNS	-	---	-
Homo sapiens CXNT	-	---	-
Pan troglodytes CXNT	-	---	-
Nomascus leucogenys CXNT	-	---	-
Mus musculus Cxnt	-	---	-
Rattus norvegicus Cxnt	-	---	-
Cavia porcellus CXNT	-	---	-
Oryctolagus cuniculus CXNT	-	---	-
Bos taurus CXNT	-	---	-
Vicugna pacos CXNT	-	---	-
Equus caballus CXNT	-	---	-
Canis lupus familiaris CXNT	-	---	-
Myotis lucifugus CXNT	-	---	-
Dasypus novemcinctus CXNT	-	---	-
Loxodonta africana CXNT	-	---	-
Homo sapiens CXNU	273	--HTKIP-DEDESEVTSSASE--	290
Pan troglodytes CXNU	273	--HTKIP-DEDESEVTSSASE--	290
Pongo abelii CXNU	258	--HTKIP-DEDESEVTSSASE--	275
Nomascus leucogenys CXNU	270	--HTKIP-DEDESEVTSSAGE--	287
Macaca mulatta CXNU	273	--HTKIP-DEDASEVTSSASE--	290
Mus musculus Cxnu	254	--LLEHS-DQDASEATSSAGD--	271
Cavia porcellus CXNU	246	--RVALL-EENGSEGLTSTSD--	263
Bos taurus CXNU	255	--RLELP-GEDESDALSSASD--	272
Equus caballus CXNU	264	--LMELP-DEDESEAMSLASD--	281
Myotis lucifugus CXNU	271	--RMELP-EEDESEGLSFTSD--	288
Sorex araneus CXNU	245	--QMELP-EELESEAMSLGSD--	262
Dasypus novemcinctus CXNU	304	--RTELP-DEVGTEVLSSAS---	320
Loxodonta africana CXNU	313	--RMELP-DEDESEVMSSASE--	330

```
Homo sapiens CXNA                 - ----------------------------------------------------------------- -
                                           790       800       810       820       830       840
Homo sapiens CXNA                 - ----------------------------------------------------------------- -
Pan troglodytes CXNA              - ----------------------------------------------------------------- -
Gorilla gorilla CXNA              - ----------------------------------------------------------------- -
Pongo abelii CXNA                 - ----------------------------------------------------------------- -
Nomascus leucogenys CXNA          - ----------------------------------------------------------------- -
Macaca mulatta CXNA               - ----------------------------------------------------------------- -
Callithrix jacchus CXNA           - ----------------------------------------------------------------- -
Mus musculus Cxna                 - ----------------------------------------------------------------- -
Rattus norvegicus Cxna            - ----------------------------------------------------------------- -
Cavia porcellus CXNA              - ----------------------------------------------------------------- -
Ochotona princeps CXNA            - ----------------------------------------------------------------- -
Bos taurus CXNA                   - ----------------------------------------------------------------- -
Equus caballus CXNA               - ----------------------------------------------------------------- -
Canis lupus familiaris CXNA       - ----------------------------------------------------------------- -
Felis catus CXNA                  - ----------------------------------------------------------------- -
Myotis lucifugus CXNA             - ----------------------------------------------------------------- -
Dasypus novemcinctus CXNA         - ----------------------------------------------------------------- -
Loxodonta africana CXNA           - ----------------------------------------------------------------- -
Homo sapiens CXNB                 - ----------------------------------------------------------------- -
Gorilla gorilla CXNB              - ----------------------------------------------------------------- -
Nomascus leucogenys CXNB          - ----------------------------------------------------------------- -
Macaca mulatta CXNB               - ----------------------------------------------------------------- -
Callithrix jacchus CXNB           - ----------------------------------------------------------------- -
Otolemur garnettii CXNB           - ----------------------------------------------------------------- -
Mus musculus Cxnb                 - ----------------------------------------------------------------- -
Rattus norvegicus Cxnb            - ----------------------------------------------------------------- -
Dipodomys ordii CXNB              - ----------------------------------------------------------------- -
Cavia porcellus CXNB              - ----------------------------------------------------------------- -
Oryctolagus cuniculus CXNB        - ----------------------------------------------------------------- -
Ochotona princeps CXNB            - ----------------------------------------------------------------- -
Bos taurus CXNB                   - ----------------------------------------------------------------- -
Equus caballus CXNB               - ----------------------------------------------------------------- -
Canis lupus familiaris CXNB       - ----------------------------------------------------------------- -
Myotis lucifugus CXNB             - ----------------------------------------------------------------- -
Erinaceus europaeus CXNB          - ----------------------------------------------------------------- -
Loxodonta africana CXNB           - ----------------------------------------------------------------- -
Homo sapiens CXNC                 - ----------------------------------------------------------------- -
Gorilla gorilla CXNC              - ----------------------------------------------------------------- -
Pongo abelii CXNC                 - ----------------------------------------------------------------- -
Nomascus leucogenys CXNC          - ----------------------------------------------------------------- -
Callithrix jacchus CXNC           - ----------------------------------------------------------------- -
Microcebus murinus CXNC           - ----------------------------------------------------------------- -
Otolemur garnettii CXNC           - ----------------------------------------------------------------- -
Mus musculus Cxnc                 - ----------------------------------------------------------------- -
Rattus norvegicus Cxnc            - ----------------------------------------------------------------- -
Cavia porcellus CXNC              - ----------------------------------------------------------------- -
Oryctolagus cuniculus CXNC        - ----------------------------------------------------------------- -
Bos taurus CXNC                   - ----------------------------------------------------------------- -
Equus caballus CXNC               - ----------------------------------------------------------------- -
Canis lupus familiaris CXNC       - ----------------------------------------------------------------- -
Felis catus CXNC                  - ----------------------------------------------------------------- -
Myotis lucifugus CXNC             - ----------------------------------------------------------------- -
Dasypus novemcinctus CXNC         - ----------------------------------------------------------------- -
Loxodonta africana CXNC           - ----------------------------------------------------------------- -
Homo sapiens CXND                 - ----------------------------------------------------------------- -
Pan troglodytes CXND              - ----------------------------------------------------------------- -
Macaca mulatta CXND               - ----------------------------------------------------------------- -
Callithrix jacchus CXND           - ----------------------------------------------------------------- -
Dipodomys ordii CXND              - ----------------------------------------------------------------- -
Oryctolagus cuniculus CXND        - ----------------------------------------------------------------- -
Tursiops truncatus CXND           - ----------------------------------------------------------------- -
Bos taurus CXND                   - ----------------------------------------------------------------- -
Equus caballus CXND               - ----------------------------------------------------------------- -
Canis lupus familiaris CXND       - ----------------------------------------------------------------- -
Myotis lucifugus CXND             - ----------------------------------------------------------------- -
Erinaceus europaeus CXND          - ----------------------------------------------------------------- -
Dasypus novemcinctus CXND         - ----------------------------------------------------------------- -
Choloepus hoffmanni CXND          - ----------------------------------------------------------------- -
Loxodonta africana CXND           - ----------------------------------------------------------------- -
Homo sapiens CXNE                 - ----------------------------------------------------------------- -
Pan troglodytes CXNE              - ----------------------------------------------------------------- -
Pongo abelii CXNE                 - ----------------------------------------------------------------- -
Macaca mulatta CXNE               - ----------------------------------------------------------------- -
Papio hamadryas CXNE              - ----------------------------------------------------------------- -
Callithrix jacchus CXNE           - ----------------------------------------------------------------- -
Otolemur garnettii CXNE           - ----------------------------------------------------------------- -
Mus musculus Cxne                 - ----------------------------------------------------------------- -
```

```
Rattus norvegicus Cxne            -  -----------------------------------------------------------------  -
Dipodomys ordii CXNE              -  -----------------------------------------------------------------  -
Cavia porcellus CXNE              -  -----------------------------------------------------------------  -
Oryctolagus cuniculus CXNE        -  -----------------------------------------------------------------  -
Equus caballus CXNE               -  -----------------------------------------------------------------  -
Canis lupus familiaris CXNE       -  -----------------------------------------------------------------  -
Felis catus CXNE                  -  -----------------------------------------------------------------  -
Myotis lucifugus CXNE             -  -----------------------------------------------------------------  -
Sorex araneus CXNE                -  -----------------------------------------------------------------  -
Dasypus novemcinctus CXNE         -  -----------------------------------------------------------------  -
Loxodonta africana CXNE           -  -----------------------------------------------------------------  -
Homo sapiens CXNF                 -  -----------------------------------------------------------------  -
Pan troglodytes CXNF              -  -----------------------------------------------------------------  -
Gorilla gorilla CXNF              -  -----------------------------------------------------------------  -
Nomascus leucogenys CXNF          -  -----------------------------------------------------------------  -
Callithrix jacchus CXNF           -  -----------------------------------------------------------------  -
Mus musculus Cxnf                 -  -----------------------------------------------------------------  -
Rattus norvegicus Cxnf            -  -----------------------------------------------------------------  -
S.tridecemlineatus CXNF           -  -----------------------------------------------------------------  -
Oryctolagus cuniculus CXNF        -  -----------------------------------------------------------------  -
Ochotona princeps CXNF            -  -----------------------------------------------------------------  -
Bos taurus CXNF                   -  -----------------------------------------------------------------  -
Equus caballus CXNF               -  -----------------------------------------------------------------  -
Canis lupus familiaris CXNF       -  -----------------------------------------------------------------  -
Myotis lucifugus CXNF             -  -----------------------------------------------------------------  -
Dasypus novemcinctus CXNF         -  -----------------------------------------------------------------  -
Choloepus hoffmanni CXNF          -  -----------------------------------------------------------------  -
Loxodonta africana CXNF           -  -----------------------------------------------------------------  -
Homo sapiens CXNG                 -  -----------------------------------------------------------------  -
Pan troglodytes CXNG              -  -----------------------------------------------------------------  -
Gorilla gorilla CXNG              -  -----------------------------------------------------------------  -
Pongo abelii CXNG                 -  -----------------------------------------------------------------  -
Nomascus leucogenys CXNG          -  -----------------------------------------------------------------  -
Macaca mulatta CXNG               -  -----------------------------------------------------------------  -
Papio hamadryas CXNG              -  -----------------------------------------------------------------  -
Callithrix jacchus CXNG           -  -----------------------------------------------------------------  -
Microcebus murinus CXNG           -  -----------------------------------------------------------------  -
Mus musculus Cxng                 -  -----------------------------------------------------------------  -
Rattus norvegicus Cxng            -  -----------------------------------------------------------------  -
Oryctolagus cuniculus CXNG        -  -----------------------------------------------------------------  -
Bos taurus CXNG                   -  -----------------------------------------------------------------  -
Equus caballus CXNG               -  -----------------------------------------------------------------  -
Canis lupus familiaris CXNG       -  -----------------------------------------------------------------  -
Felis catus CXNG                  -  -----------------------------------------------------------------  -
Myotis lucifugus CXNG             -  -----------------------------------------------------------------  -
Pteropus vampyrus CXNG            -  -----------------------------------------------------------------  -
Dasypus novemcinctus CXNG         -  -----------------------------------------------------------------  -
Loxodonta africana CXNG           -  -----------------------------------------------------------------  -
Procavia capensis CXNG            -  -----------------------------------------------------------------  -
Homo sapiens CXNH1              300  ---------------------------------------------LASSR--------PP-L-FL  309
Pan troglodytes CXNH1           300  ---------------------------------------------LASSR--------PP-L-FL  309
Gorilla gorilla CXNH1           300  ---------------------------------------------LASSR--------PP-L-FL  309
Pongo abelii CXNH1              300  ---------------------------------------------LASSR--------PP-L-FL  309
Nomascus leucogenys CXNH1       300  ---------------------------------------------LASSR--------PP-L-FL  309
Macaca mulatta CXNH1            300  ---------------------------------------------LASSR--------PP-L-FL  309
Otolemur garnettii CXNH1        298  ---------------------------------------------LDSSR--------TP-L-SQ  307
Mus musculus Cxnh1              300  ---------------------------------------------LTSSR--------PP-P-FV  309
Rattus norvegicus Cxnh1         300  ---------------------------------------------LTSTR--------PP-P-FV  309
Cavia porcellus CXNH1           300  ---------------------------------------------LASSR--------GP-L-FL  309
Oryctolagus cuniculus CXNH1     300  ---------------------------------------------LASSR--------PP-L-FL  309
Ochotona princeps CXNH1         314  ---------------------------------------------LTSSR--------AP-L-FL  323
Equus caballus CXNH1            300  ---------------------------------------------LASSG--------PP-L-FL  309
Canis lupus familiaris CXNH1    300  ---------------------------------------------LASSR--------AP-L-FL  309
Felis catus CXNH1               300  ---------------------------------------------LASSR--------AP-L-FL  309
Myotis lucifugus CXNH1          331  ---------------------------------------------LASSR--------PP-P-FL  340
Dasypus novemcinctus CXNH1      300  ---------------------------------------------LASSR--------SP-L-YL  309
Loxodonta africana CXNH1        300  ---------------------------------------------LVSSS--------PH-L-FL  309
Homo sapiens CXNI               295  NLV-TEQVR-GQE-QTPGE-GF-IQVRY-G-QKP--EVP-NGV--SPGHR---------L----P  334
Pan troglodytes CXNI            295  NLA-TEQVR-GQE-QTPGE-GF-IQVRY-G-QKP--EVP-NGA--SPGHR---------L----P  334
Pongo abelii CXNI               295  NLA-TEQVR-GQE-QTPGE-GF-IQVRY-G-QKP--EVP-NGV--SPGHR---------L----P  334
Nomascus leucogenys CXNI        295  NLA-TEQVR-GQE-QTPGE-GF-IPVCY-G-QKP--EVP-NGV--SPGHR---------L----P  334
Macaca mulatta CXNI             295  NLA-TEQVR-GQE-QTPGE-GF-IQVRY-G-QKP--EVP-NGV--SPGHR---------L----P  334
Papio hamadryas CXNI            295  NLA-TEQVR-GQE-QTPGE-GF-IQVRY-G-QKP--EVP-NGV--SPGHR---------L----P  334
Callithrix jacchus CXNI         295  NLA-TEQVR-GQE-QTPGQ-GF-IQVPY-D-QKP--EVP-NGV--SPGHR---------L----P  334
Otolemur garnettii CXNI         294  NLA-TEQVR-GQE-QVPGE-GF-IHIHY-G-QKP--EVP-NGA--SPGHC---------L----P  333
Mus musculus Cxni               295  ALA-TGEVP-NQE-QIPGE-GF-IHMHY-S-QKP--EYA-SGA--SAGHR---------L----P  334
Rattus norvegicus Cxni          327  PLA-TEEVP-NQE-QIPEE-GF-IHTQY-G-QKP--EQP-SGA--SAGHR---------F----P  366
Cavia porcellus CXNI            295  NLA-TEQVR-GQE-RIPGE-DF-IHIHY-G-EKP--DVP-SGV--SSGHR---------L----P  334
Oryctolagus cuniculus CXNI      295  NLA-TERVR-GRE-QVPGE-GF-IHIRY-D-QKP--DVP-DGV--SPGHP---------N----P  334
```

```
Bos taurus CXNI                   296 NLA-TEQVR-SQE-QIPRE-GF-IHIRY-A-QKP--EVP-NEG--SPGPS---------L----P 335
Equus caballus CXNI               295 NLA-TEQVR-DQE-QIPGE-GF-IHIRY-A-QKP--EVP-NGV--SPGHR---------L----P 334
Canis lupus familiaris CXNI       295 NLA-TEQVQ-GQE-PIPGE-GF-IHIRY-A-QKP--EVP-NGA--SPGHR---------L----P 334
Felis catus CXNI                  295 NLA-TEQVQ-GQE-QIPGE-GF-IHIRY-A-QKP--EVP-NGA--SPGHR---------I----P 334
Pteropus vampyrus CXNI            295 NLA-TEQVR-GQE-QVPGE-GF-IHVHY-S-QKP--EVP-SEV--SSGHR---------L----P 334
Erinaceus europaeus CXNI          296 NLA-AEQGR-GQE-QGPRE-GF-IHIHY-A-QKP--EIP-NEV--CPGHR--------GL----P 336
Dasypus novemcinctus CXNI         295 NLA-TEQVR-GQE-ETPGE-DF-IHIRY-A-QKP--EVP-NGA--SPGHR---------L----P 334
Loxodonta africana CXNI           295 NLA-TEQVR-GQE-EIPGE-GF-INIRY-A-QRP--EVP-NGV--SPGHR---------L----P 334
Homo sapiens CXNJ1                368 AGA-APLLL-DGS-GSSLE-GS-ALAGT-P-EEE--EQAVTTA--AQMHQ---------P----P 408
Pongo abelii CXNJ1                368 AGA-APLLL-DGS-GSSLE-GS-ALAGT-P-EEE--EQAVTTA--AQMHQ---------P----P 408
Macaca mulatta CXNJ1              365 AGA-AHLLL-DGS-GSSLE-GS-ALAGT-P-EEE--EQAVTTA--AQMHQ---------P----P 405
Mus musculus Cxnj1                358 ------GLI-DSS-GSSLQ-ES-ALVVT-P-EEG--EQALATT--VEMHS---------P----P 393
Rattus norvegicus Cxnj1           358 ------GLT-DSS-GSSLE-ES-ALVVT-P-E-G--EQALATT--VEMHS---------P----P 392
Bos taurus CXNJ1                  349 -----------GP-QQSLP-EG-AAGSS-G-DSD--GEGAVTA--VELHA---------P----P 380
Equus caballus CXNJ1              381 SKA-PALLA-NGN-GSPLG-ES-KLEVT-P-DEE--EWAVTTA--VEMHA---------P----P 421
Myotis lucifugus CXNJ1              - ----------------------------------------------------------------  -
Myotis lucifugus CXNJ2              - ----------------------------------------------------------------  -
Pteropus vampyrus CXNJ1           535 ----PVPVE-NGN-GHSLG-ES-SLEVT-P-DEG--VPAVTSP--VDMHA---------P----P 572
Pteropus vampyrus CXNJ2             - ----------------------------------------------------------------  -
Sorex araneus CXNJ1               253 ------------------------------------PRAVGA--LQMHG---------P----P 265
Homo sapiens CXNK1                311 NYS-AEQNRMGQA-GSTIS-NSHAQPFD-F-PDD--NQNSKKL--AAGHE---------LQ---P 354
Homo sapiens CXNK2                312 NYS-AEQNRMGQA-GSTIS-NSHAQPFD-F-PDD--NQNSKKL--AAGHE---------LQ---P 355
Pan troglodytes CXNK1             311 NYS-AEQNRMGQA-GSTIS-NSHAQPFD-F-PDD--NQNSKKL--AAGHE---------LQ---P 354
Pan troglodytes CXNK2             312 NYS-AEQNRMGQA-GSTIS-NSHAQPFD-F-PDD--NQNSKKL--AAGHE---------LQ---P 355
Pongo abelii CXNK1                312 NYS-AEQNRMGQA-GSTIS-NSHAQPFD-F-PDD--NQNSKKL--AAGHE---------LQ---P 355
Nomascus leucogenys CXNK1         312 NYS-AEQNRMGQA-GSTIS-NSHAQPFD-F-PDD--NQNSKKL--AAGHE---------LQ---P 355
Callithrix jacchus CXNK1          312 NYS-AEQNRMGQA-GSTIS-NSHAQPFD-F-PDD--NQNSKKL--AAGHE---------LQ---P 355
Mus musculus Cxnk1                312 NYS-AEQNRMGQA-GSTIS-NSHAQPFD-F-PDD--SQNAKKV--AAGHE---------LQ---P 355
Mus musculus Cxnk2                  - ----------------------------------------------------------------  -
Rattus norvegicus Cxnk1           312 NYS-AEQNRMGQA-GSTIS-NSHAQPFD-F-PDD--NQNAKKV--AAGHE---------LQ---P 355
Rattus norvegicus Cxnk2           284 FYM-------------------------------------------------------------- 286
Cavia porcellus CXNK1             312 NYS-AEQNRMGQA-GSTIS-NSHAQPFD-F-PDD--NQNSKKI--SAGHE---------LQ---P 355
Oryctolagus cuniculus CXNK1       312 NYS-AEQNRMGQA-GSTIS-NSHAQPFD-F-PDD--NQNSKKL--AAGHE---------LQ---P 355
Bos taurus CXNK1                  313 NYS-AEQNRMGQA-GSTIS-NSHAQPFD-F-PDD--HQNSKKL--DAGHE---------LQ---P 356
Vicugna pacos CXNK1               312 NYS-AEQNRMGQA-GSTIS-NSHAQPFD-F-PDD--NQNAKKL--DAGHE---------LQ---P 355
Equus caballus CXNK1              312 NYS-AEQNRMGQA-GSTIS-NSHAQPFD-F-PDD--NQNPKKL--DTRHE---------LQ---P 355
Equus caballus CXNK2              278 NYS-A------------------------------------------------------------ 281
Canis lupus familiaris CXNK1      312 NYS-AEQNRMGQA-GSTIS-NSHAQPFD-F-PDD--NQNSKKL--AAGHE---------LQ---P 355
Canis lupus familiaris CXNK2      304 KQS-A------------------------------------------------------------ 307
Felis catus CXNK1                 299 K---------------------------------------------------------------- 299
Myotis lucifugus CXNK1            312 NYS-AEQNRMGQA-GSTIS-NSHAQPFD-F-PDD--NQNSKKL--AAGHE---------LQ---P 355
Dasypus novemcinctus CXNK1          - ----------------------------------------------------------------  -
Dasypus novemcinctus CXNK2        312 NYS-AEQNRMGQA-GSTIS-NSHAQPFD-F-PDD--NQNSKKL-VAAGHE---------LQ---P 356
Loxodonta africana CXNK1          312 NYS-AEQNRMGQA-GSTIS-NSHAQPFD-F-PDD--NENSKKL--ATGHE---------LQ---P 355
Loxodonta africana CXNK2          311 DYS-A------------------------------------------------------------ 314
Homo sapiens CXNL                 363 KVAVPEGEK-V-E--TPGV-DK-EGEKE-E-PQS--EKVSKQG--LPAEK--------TPSLC-P 406
Pan troglodytes CXNL              363 KVAVPEGEK-V-E--TPRV-DK-EGEKE-E-LQS--EKVSKQG--LPAEK--------TPSLC-P 406
Pongo abelii CXNL                 363 KVAMPEGEK-V-E--TPGV-EK-ESEKE-E-LQS--EKVSKQG--LPAEK--------TPSLC-P 406
Nomascus leucogenys CXNL          363 KVAVPEGEK-V-E--TPGV-GK-EGEKE-E-LQS--EKVSKQG--LPAER--------TPSLC-P 406
Macaca mulatta CXNL               365 KVAVPEGEE-V-E--TPGV-GK-EGEKE-E-LQS--EKVSKQG--LPAEK--------TPSLC-Q 408
Callithrix jacchus CXNL           366 KVAVPDREK-V-E--TPGV-GK-EGEKE-E-LQS--E-VAKQG--LPAEK--------TPSLC-P 408
Otolemur garnettii CXNL           414 TAAVSDGEK-A-E--TPGT-GK-EGEKE-E-LQA--EKVSKQG--LLAEK--------TPSLC-P 457
Mus musculus Cxnl                 370 TVAVPDRER-V-E--TPGV-GK-EDEKE-E-LQA--EKVTKQG--LSAEK--------APSLC-P 413
Rattus norvegicus Cxnl            370 TVAVPDGEK-V-E--TPGV-GK-DDEKE-E-LQA--EKVTKQG--LSAEK--------APTLC-P 413
Cavia porcellus CXNL              370 ALAEPDGER-P-G--TPGV-GK-EGEKE-E-LQA--ETVTKQG--LSTEK--------TPSLC-P 413
Oryctolagus cuniculus CXNL        370 MVAVLEAEK-I-E--PPAV-GK-EGEKE-E-LQA--E-VAKQG--LAAEK--------TPPLC-P 412
Ochotona princeps CXNL            367 TVQVPEGEK-V-E--TPGV-GT-EGEKE-E-LPA--EKVSKPG--LLAEK--------TPSLC-P 410
Bos taurus CXNL                   370 TLAVLEVEK-V-E--PPEV-EK-EVEKE-E-PPP--EKVSKQE--LTPEK--------APSLC-A 413
Equus caballus CXNL               371 TTAVLEGEK-V-E--TPKV-GK-EAEKE-E-LQA--EKVSKQE--LPAEK--------APSLC-P 414
Canis lupus familiaris CXNL       370 TMAVLEGEK-A-E--TPEV-GK-EGEKE-E-LQA--E-VSKQG--LPAEK--------SPSLC-P 412
Felis catus CXNL                  370 TLAVLEGEK-V-E--TPEV-GK-EGEKD-E-LQA--EKVAKQG--LPVEK--------SPSLC-P 413
Pteropus vampyrus CXNL            370 TNDELEGDR-V-E--TPGA-GK-EGEKE-E-LQA--EKASKQG--LPAEK--------APSLC-P 413
Sorex araneus CXNL                325 ------GE--V-E--SPEA-GT-ESEDE-E-LQT--KKPP------------------PPLS-S 352
Dasypus novemcinctus CXNL         372 MVVVPEGEK-I-E--PPEG-EK-QGEKE-E-LQA--EMVSKQR--VPAEK--------APALC-A 415
Loxodonta africana CXNL           369 --AVLEGEK-G-E--TPKV-GK-ESEKE-E-LQA--EQISKQG--LPAEK--------APSLC-A 410
Homo sapiens CXNM                 363 N-GNQLMEK--RE-TEGKD--SKRNYYSRG-HRSIPGV-AIDG--ENNMRQSPQTVFSLPANC-D 416
Pan troglodytes CXNM              363 N-GNQLMEK--RE-TEGKD--SKRNYYSRG-HRSIPGV-AIDG--ENNMRQSPQTAFSLPANC-D 416
Pongo abelii CXNM                 363 N-GNQLMVK--RE-TEGKD--SKRNYYSRG-HRSIPGV-AIDG--ENNM-FSPQTVFSLPANC-D 415
Nomascus leucogenys CXNM          363 N-GNQLMEK--RE-TEGKD--SKRNYYSRG-HRSIPGV-AIDG--ENNMRQSPQTVFSLPANC-D 416
Macaca mulatta CXNM               363 N-GNQLREK--RE-TEGKD--RKRNYYSRG-HRSIPGV-AIDG--ENNMRQSPQTAFSLPANC-D 416
Callithrix jacchus CXNM           362 N-GNQLREK--RE-TEGKD--SKRNHHSRG-HSSIPGV-AMDG--ENYRRQSPQTAFSLPANC-D 415
Tarsius syrichta CXNM             359 --GDQLREK--RE-TDGKD--SQRN--SRD-HCSIPGV-AINL--DNHMGQSPQTACPLPANS-T 409
Microcebus murinus CXNM           363 N-GTQLREK--RE-TDGKD--SKKN--SRC-HCSIPGV-ATDL--DNHMGQLPQTAFSRSANC-T 414
Dipodomys ordii CXNM              354 G-GKNLR-K-------------------------------------------------------- 360
Oryctolagus cuniculus CXNM        356 N-GNQLREK--RA-TDGQD--SQRNHCSRG-HCSVSSV-AGDL--DNYTERLPQAGVSLPANH-T 409
Equus caballus CXNM               362 N-GNQLREK--RE-IDGKD--SKRNDYCRG-HCPIPGV-AVDP--DNHAGQSSQTAFSQPPNC-T 415
Canis lupus familiaris CXNM       362 --GTPLKEK--RE-MACKD--GKRNHCSRG-HCSIPGV-AIEL--DNHMGQSSQTAFPPPAQG-A 414
Pteropus vampyrus CXNM            359 N-GNHLREK--K--IDGKD--SKRNQNARG-NCSIPGV-ARDL--DNHMGLTSQRAFSLPANC-M 411
Loxodonta africana CXNM           362 D-DNQVKER--RE-NFGTD--SERCHYSK-------GV-SVDL--ENHVSQSPQKVFSLPANC-N 409
```

Homo sapiens CXNN	362	NTMSQSWLG--TT-TAPRNCPS----FAVG---T-WEQ-SQDP--EPS-GE-PLT--DLHSHC-R	407
Pan troglodytes CXNN	362	NTMSQSWLG--TT-TAPRNCPS----FAIG---T-WEQ-SQDP--EPS-GE-PLT--DLHSHC-R	407
Pongo abelii CXNN	362	NTMSQSWLG--TT-TAPRNCPS----YAIG---T-WEQ-SQDP--EPS-GE-PLT--DLHSHC-R	407
Nomascus leucogenys CXNN	362	NTMSQSWLG--TT-TAPRNCPS----YAIG---T-WEQ-SEDP--EPS-GE-PLT--DLHSHC-R	407
Macaca mulatta CXNN	362	NTMSQSWLG--TT-TAPRNCPS----YAIG---T-WEQ-SQDP--EPS-GE-PLT--DLHSHC-R	407
Callithrix jacchus CXNN	363	NTISQSWLG--TT-MAPRNCPS----HAIG---T-WEQ-SQDL--EPS-GE-PLT--DLHGHC-R	408
Microcebus murinus CXNN	362	NAMSQSWLG---M-TAPKNCPS----YAIG---T-WEQ-PQDL--EPS-GE--LT--DLHSHY-R	405
Mus musculus Cxnn	362	NAMSQSWFG--TM-TASQHRPS----SALE---T-WER-SQGP--EAS-GR-SLT--DRQSHF-Q	407
Rattus norvegicus Cxnn	362	NVMSQSWLG--TM-TASQHRPS----SALE---T-WER-SQGP--EVS-GK-PLT--DRQSHF-Q	407
Oryctolagus cuniculus CXNN	392	NTTIQSWPG--TT-TTSRHCPA----YAVG---T-WKQ-SQDL--EPL-GE-PLT--DLHS----	434
Bos taurus CXNN	362	NIRPHSWLG--TK-MAPRHCPS----HTTG---P-WEQ-SQDL--QPS-GE-PLA--DLHSHC-R	407
Equus caballus CXNN	361	KTTSQSCLG--ST-TAPRHCPS----YAIG---T-WEQ-SQDL--KLS-GE-PLT--DFHSHC-R	406
Canis lupus familiaris CXNN	361	NAKSQSWVG--TE-MASKHYQS----YAVG---T-WEQ-SHGR--RSS-RE-PLT--DLHSFC-R	406
Sorex araneus CXNN	361	NAKCHSWLL--TA-APPRQGAS----YAPG---P-WEQ-AQVR--HLA-GE-SLT--DAQSLC-R	406
Loxodonta africana CXNN	363	NTVSQSWLE--RT-TAPEHCPS----YVPG---T-WEL-PQDL--KPP-GE-PLT--DLHSHC-K	408
Homo sapiens CXNO	361	G-A-A--AG-DRDRDSSP-CVGLPAA-SRG-PPRAGAPASRTGS-AT----SAGTVGEQGR---P	409
Papio hamadryas CXNO	363	G-A-A--AG-DRDRDSSP-CVGLPAA-SRG-PLRAGAPTSGTGS-AT----SAGTVGEQGR---P	411
Mus musculus Cxno	371	G-A-AVAAV-SADRDSPP-CAGLNAT-SRG-APRVGGLASGTGS-AT----SGGTVGEQSR---P	421
Cavia porcellus CXNO	352	--------P-DHDHDSPP-CAGLSAA-SRG-PPRAGAPASGSGSNASG---SGGAMGDRSR----	397
Homo sapiens CXNP1	-	---	-
Pan troglodytes CXNP1	-	---	-
Pongo abelii CXNP1	-	---	-
Callithrix jacchus CXNP1	-	---	-
Otolemur garnettii CXNP1	-	---	-
Tupaia belangeri CXNP1	-	---	-
Mus musculus Cxnp1	-	---	-
Rattus norvegicus Cxnp1	-	---	-
Cavia porcellus CXNP1	-	---	-
Oryctolagus cuniculus CXNP1	-	---	-
Oryctolagus cuniculus CXNP2	-	---	-
Ochotona princeps CXNP1	-	---	-
Bos taurus CXNP1	-	---	-
Equus caballus CXNP1	-	---	-
Canis lupus familiaris CXNP1	-	---	-
Felis catus CXNP1	-	---	-
Myotis lucifugus CXNP1	-	---	-
Dasypus novemcinctus CXNP1	-	---	-
Dasypus novemcinctus CXNP2	-	---	-
Dasypus novemcinctus CXNP3	-	---	-
Dasypus novemcinctus CXNP4	-	---	-
Choloepus hoffmanni CXNP1	-	---	-
Loxodonta africana CXNP1	-	---	-
Homo sapiens CXNQ	322	Q-Q----YG-SHE-ENLP--ADLEAL-QRE-IRMAQERLDLAVQ-AY----SHQNNPHGPR----	366
Pan troglodytes CXNQ	322	Q-Q----YG-SHE-ENLP--ADLEAL-QRE-IRMAQERLDLAVQ-AY----SHQNNPHGPR----	366
Pongo abelii CXNQ	322	Q-Q----YG-SHE-ENLP--ADLEAL-QRE-IRMAQERLDLAVQ-AY----SHQNNPHGPR----	366
Macaca mulatta CXNQ	322	Q-Q----YG-SHE-ENLP--ADLEAL-QRE-IRMAQERLDLAIQ-AY----SHQNNPHGPR----	366
Tupaia belangeri CXNQ	322	Q-Q----YG-SHE-ESLP--ADLETL-QRE-IRMAQERLDLAIQ-AY----NHQNNPHGPR----	366
Mus musculus Cxnq	322	Q-Q----YG-SHE-EHLP--ADLETL-QRE-IRMAQERLDLAIQ-AY----HHQNNPHGPR----	366
Rattus norvegicus Cxnq	322	Q-Q----YG-SHE-EHLP--ADLETL-QRE-IRMAQERLDLAIQ-AY----HHQNNPHGPR----	366
Cavia porcellus CXNQ	322	Q-Q----YG-SRE-EPLP--AELETL-QRE-IRLAQERLDLAIQ-AF----HHQTHLPAAR----	366
S.tridecemlineatus CXNQ	336	Q-Q----YG-SHE-EHLP--ADLETL-QRE-IRMAQERLDLAIQ-AY----NHQNNPHGPR----	380
Oryctolagus cuniculus CXNQ	322	Q-Q----YG-SHE-EHLP--ADLETL-QRE-IRMAQERLDLAIQ-AY----SHQNNPHGPR----	366
Bos taurus CXNQ	322	Q-Q----YG-SHE-DNLP--PDLETL-QRE-IRMAQERLDLAIQ-AY----NHQNNPHGSR----	366
Vicugna pacos CXNQ	322	Q-Q----YG-SHE-ANLP--ADLETL-QRE-IRMAQERLDLAIQ-AY----NHQNNPHGPR----	366
Equus caballus CXNQ	322	Q-Q----YR-SHE-ENLP--ADLETL-QRE-IRMAQERLDLAIQ-AY----SHQNNPHGPR----	366
Canis lupus familiaris CXNQ	322	Q-Q----YG-SHE-ENLP--ADLETL-QRE-IKMAQERLDLAIQ-AY----SHQNNPHGPR----	366
Myotis lucifugus CXNQ	322	Q-Q----YG-SHE-EHLP--ADLETL-QRE-IRMAQERLDLAIQ-AY----NHQNNPPAPR----	366
Pteropus vampyrus CXNQ	322	Q-Q----YG-SHE-EHLP--ADLESL-QRE-IRMAQERLDLAIQ-AY----NHQNNPHGPR----	366
Loxodonta africana CXNQ	322	Q-Q----YG-SHE-ENLP--ADLETL-QRE-IRMAQERLDLAIQ-AY----AHQNNPHGPR----	366
Homo sapiens CXNR	272	---------------SLR---ECGSG--RGKASPATGRRDLAI----------------------	294
Pan troglodytes CXNR	271	---------------SLR---ECGSG--RGKASPATGRRDLAI----------------------	293
Pongo abelii CXNR	272	---------------SLR---EGGSG--RGKASPATGRRDLAI----------------------	294
Papio hamadryas CXNR	273	---------------SHR---EGGSG--RGKASPATGRRDLAI----------------------	295
Mus musculus Cxnr	254	-------------GDSEG---EGGSG--HSKASLATVRQDLAI----------------------	278
Rattus norvegicus Cxnr	254	-------------GDSEG---EGGSG--HSKASLATVRQDLAI----------------------	278
Oryctolagus cuniculus CXNR	276	---------------SAG---EGGSA--RSKASLATVRQDLAI----------------------	298
Bos taurus CXNR	262	-------------GDS-----EGGSG--RSKASLATIRQDLAI----------------------	284
Homo sapiens CXNS	-	---	-
Pan troglodytes CXNS	-	---	-
Pongo abelii CXNS	-	---	-
Nomascus leucogenys CXNS	-	---	-
Macaca mulatta CXNS	-	---	-
Papio hamadryas CXNS	-	---	-
Otolemur garnettii CXNS	-	---	-
Tupaia belangeri CXNS	-	---	-
Mus musculus Cxns	-	---	-
Rattus norvegicus Cxns	-	---	-
Cavia porcellus CXNS	-	---	-
S.tridecemlineatus CXNS	-	---	-

```
Oryctolagus cuniculus CXNS       - -----------------------------------------------------------------   -
Ochotona princeps CXNS           - -----------------------------------------------------------------   -
Bos taurus CXNS                  - -----------------------------------------------------------------   -
Equus caballus CXNS              - -----------------------------------------------------------------   -
Canis lupus familiaris CXNS      - -----------------------------------------------------------------   -
Myotis lucifugus CXNS            - -----------------------------------------------------------------   -
Dasypus novemcinctus CXNS        - -----------------------------------------------------------------   -
Loxodonta africana CXNS          - -----------------------------------------------------------------   -
Homo sapiens CXNT                - -----------------------------------------------------------------   -
Pan troglodytes CXNT             - -----------------------------------------------------------------   -
Nomascus leucogenys CXNT         - -----------------------------------------------------------------   -
Mus musculus Cxnt                - -----------------------------------------------------------------   -
Rattus norvegicus Cxnt           - -----------------------------------------------------------------   -
Cavia porcellus CXNT             - -----------------------------------------------------------------   -
Oryctolagus cuniculus CXNT       - -----------------------------------------------------------------   -
Bos taurus CXNT                  - -----------------------------------------------------------------   -
Vicugna pacos CXNT               - -----------------------------------------------------------------   -
Equus caballus CXNT              - -----------------------------------------------------------------   -
Canis lupus familiaris CXNT      - -----------------------------------------------------------------   -
Myotis lucifugus CXNT            - -----------------------------------------------------------------   -
Dasypus novemcinctus CXNT        - -----------------------------------------------------------------   -
Loxodonta africana CXNT          - -----------------------------------------------------------------   -
Homo sapiens CXNU              291 KLG--RQPR----GRPHR---EAAQD-PRG---SGSE--EQP-S-AAP---SRLA-AP-PS---- 329
Pan troglodytes CXNU           291 KLG--RQPR----GRPHR---EAAQD-PRG---SGSE--EQP-S-AAL---SRLA-AH-PS---- 329
Pongo abelii CXNU              276 KLG--RQPR----GRPHR---EAAQD-PRG---LGSE--EQP-S-AAP---SRLA-AH-PS---- 314
Nomascus leucogenys CXNU       288 KLG--RQPR----GRPHR---EAAQD-PRG---SGSE--EQP-S-AAP---SRLA-AH-PS---- 326
Macaca mulatta CXNU            291 KLG--RQPR----GRPYR---EAAQD-PRG---SGSE--EQP-S-AAP---SHLA-AH-PS---- 329
Mus musculus Cxnu              272 RLT--VAHT-AHELRFHR---ETSLD-LGGK-NTQAD--ELS-L-ATQ---SHLA-RH-SS---- 315
Cavia porcellus CXNU           264 KAP--VAHR-EPGGRCPR---EATQD-PKSRGLAHSE--VCS-L-ASQ---SHLA-GH-YS---- 308
Bos taurus CXNU                273 QPR--LA-R--PGAAA-----------------LGWE--ERP-C-APR---AQLS-GP-G----- 301
Equus caballus CXNU            282 KPA--RAGP-EPGSRPHG---EASQD-LRSEGLAGSE--EPP-P-APR---SRLA-RR-CP---- 326
Myotis lucifugus CXNU          289 KVS--RACT-EPGGRP-R--AEVPPD-SRN---------EHP-L-KPH---RLQA-QH-CP---- 326
Sorex araneus CXNU             263 KLA--ERCR-DGEGGPGG--AEAVHH-VR-----------PA-S-APR---SRLA-QN-S----- 298
Dasypus novemcinctus CXNU      321 ----------------------------RSEGHPRSQ--GLP-S-APR---SHVA-GH-DS---- 344
Loxodonta africana CXNU        331 KLA--RAHP-ELRGRLHR---EATQD-PRREGHADWG--ELP-S-APR---SRLA-GH-YS---- 375
```

```
Homo sapiens CXNA                  - ----------------------------------------------------------------- -
                                      850       860       870       880       890       900       910
Homo sapiens CXNA                  - ----------------------------------------------------------------- -
Pan troglodytes CXNA               - ----------------------------------------------------------------- -
Gorilla gorilla CXNA               - ----------------------------------------------------------------- -
Pongo abelii CXNA                  - ----------------------------------------------------------------- -
Nomascus leucogenys CXNA           - ----------------------------------------------------------------- -
Macaca mulatta CXNA                - ----------------------------------------------------------------- -
Callithrix jacchus CXNA            - ----------------------------------------------------------------- -
Mus musculus Cxna                  - ----------------------------------------------------------------- -
Rattus norvegicus Cxna             - ----------------------------------------------------------------- -
Cavia porcellus CXNA               - ----------------------------------------------------------------- -
Ochotona princeps CXNA             - ----------------------------------------------------------------- -
Bos taurus CXNA                    - ----------------------------------------------------------------- -
Equus caballus CXNA                - ----------------------------------------------------------------- -
Canis lupus familiaris CXNA        - ----------------------------------------------------------------- -
Felis catus CXNA                   - ----------------------------------------------------------------- -
Myotis lucifugus CXNA              - ----------------------------------------------------------------- -
Dasypus novemcinctus CXNA          - ----------------------------------------------------------------- -
Loxodonta africana CXNA            - ----------------------------------------------------------------- -
Homo sapiens CXNB                  - ----------------------------------------------------------------- -
Gorilla gorilla CXNB               - ----------------------------------------------------------------- -
Nomascus leucogenys CXNB           - ----------------------------------------------------------------- -
Macaca mulatta CXNB                - ----------------------------------------------------------------- -
Callithrix jacchus CXNB            - ----------------------------------------------------------------- -
Otolemur garnettii CXNB            - ----------------------------------------------------------------- -
Mus musculus Cxnb                  - ----------------------------------------------------------------- -
Rattus norvegicus Cxnb             - ----------------------------------------------------------------- -
Dipodomys ordii CXNB               - ----------------------------------------------------------------- -
Cavia porcellus CXNB               - ----------------------------------------------------------------- -
Oryctolagus cuniculus CXNB         - ----------------------------------------------------------------- -
Ochotona princeps CXNB             - ----------------------------------------------------------------- -
Bos taurus CXNB                    - ----------------------------------------------------------------- -
Equus caballus CXNB                - ----------------------------------------------------------------- -
Canis lupus familiaris CXNB        - ----------------------------------------------------------------- -
Myotis lucifugus CXNB              - ----------------------------------------------------------------- -
Erinaceus europaeus CXNB           - ----------------------------------------------------------------- -
Loxodonta africana CXNB            - ----------------------------------------------------------------- -
Homo sapiens CXNC                  - ----------------------------------------------------------------- -
Gorilla gorilla CXNC               - ----------------------------------------------------------------- -
Pongo abelii CXNC                  - ----------------------------------------------------------------- -
Nomascus leucogenys CXNC           - ----------------------------------------------------------------- -
Callithrix jacchus CXNC            - ----------------------------------------------------------------- -
Microcebus murinus CXNC            - ----------------------------------------------------------------- -
Otolemur garnettii CXNC            - ----------------------------------------------------------------- -
Mus musculus Cxnc                  - ----------------------------------------------------------------- -
Rattus norvegicus Cxnc             - ----------------------------------------------------------------- -
Cavia porcellus CXNC               - ----------------------------------------------------------------- -
Oryctolagus cuniculus CXNC         - ----------------------------------------------------------------- -
Bos taurus CXNC                    - ----------------------------------------------------------------- -
Equus caballus CXNC                - ----------------------------------------------------------------- -
Canis lupus familiaris CXNC        - ----------------------------------------------------------------- -
Felis catus CXNC                   - ----------------------------------------------------------------- -
Myotis lucifugus CXNC              - ----------------------------------------------------------------- -
Dasypus novemcinctus CXNC          - ----------------------------------------------------------------- -
Loxodonta africana CXNC            - ----------------------------------------------------------------- -
Homo sapiens CXND                  - ----------------------------------------------------------------- -
Pan troglodytes CXND               - ----------------------------------------------------------------- -
Macaca mulatta CXND                - ----------------------------------------------------------------- -
Callithrix jacchus CXND            - ----------------------------------------------------------------- -
Dipodomys ordii CXND               - ----------------------------------------------------------------- -
Oryctolagus cuniculus CXND         - ----------------------------------------------------------------- -
Tursiops truncatus CXND            - ----------------------------------------------------------------- -
Bos taurus CXND                    - ----------------------------------------------------------------- -
Equus caballus CXND                - ----------------------------------------------------------------- -
Canis lupus familiaris CXND        - ----------------------------------------------------------------- -
Myotis lucifugus CXND              - ----------------------------------------------------------------- -
Erinaceus europaeus CXND           - ----------------------------------------------------------------- -
Dasypus novemcinctus CXND          - ----------------------------------------------------------------- -
Choloepus hoffmanni CXND           - ----------------------------------------------------------------- -
Loxodonta africana CXND            - ----------------------------------------------------------------- -
Homo sapiens CXNE                  - ----------------------------------------------------------------- -
Pan troglodytes CXNE               - ----------------------------------------------------------------- -
Pongo abelii CXNE                  - ----------------------------------------------------------------- -
Macaca mulatta CXNE                - ----------------------------------------------------------------- -
Papio hamadryas CXNE               - ----------------------------------------------------------------- -
Callithrix jacchus CXNE            - ----------------------------------------------------------------- -
Otolemur garnettii CXNE            - ----------------------------------------------------------------- -
Mus musculus Cxne                  - ----------------------------------------------------------------- -
```

```
Rattus norvegicus Cxne          -  -----------------------------------------------------------------  -
Dipodomys ordii CXNE            -  -----------------------------------------------------------------  -
Cavia porcellus CXNE            -  -----------------------------------------------------------------  -
Oryctolagus cuniculus CXNE      -  -----------------------------------------------------------------  -
Equus caballus CXNE             -  -----------------------------------------------------------------  -
Canis lupus familiaris CXNE     -  -----------------------------------------------------------------  -
Felis catus CXNE                -  -----------------------------------------------------------------  -
Myotis lucifugus CXNE           -  -----------------------------------------------------------------  -
Sorex araneus CXNE              -  -----------------------------------------------------------------  -
Dasypus novemcinctus CXNE       -  -----------------------------------------------------------------  -
Loxodonta africana CXNE         -  -----------------------------------------------------------------  -
Homo sapiens CXNF               -  -----------------------------------------------------------------  -
Pan troglodytes CXNF            -  -----------------------------------------------------------------  -
Gorilla gorilla CXNF            -  -----------------------------------------------------------------  -
Nomascus leucogenys CXNF        -  -----------------------------------------------------------------  -
Callithrix jacchus CXNF         -  -----------------------------------------------------------------  -
Mus musculus Cxnf               -  -----------------------------------------------------------------  -
Rattus norvegicus Cxnf          -  -----------------------------------------------------------------  -
S.tridecemlineatus CXNF         -  -----------------------------------------------------------------  -
Oryctolagus cuniculus CXNF      -  -----------------------------------------------------------------  -
Ochotona princeps CXNF          -  -----------------------------------------------------------------  -
Bos taurus CXNF                 -  -----------------------------------------------------------------  -
Equus caballus CXNF             -  -----------------------------------------------------------------  -
Canis lupus familiaris CXNF     -  -----------------------------------------------------------------  -
Myotis lucifugus CXNF           -  -----------------------------------------------------------------  -
Dasypus novemcinctus CXNF       -  -----------------------------------------------------------------  -
Choloepus hoffmanni CXNF        -  -----------------------------------------------------------------  -
Loxodonta africana CXNF         -  -----------------------------------------------------------------  -
Homo sapiens CXNG               -  -----------------------------------------------------------------  -
Pan troglodytes CXNG            -  -----------------------------------------------------------------  -
Gorilla gorilla CXNG            -  -----------------------------------------------------------------  -
Pongo abelii CXNG               -  -----------------------------------------------------------------  -
Nomascus leucogenys CXNG        -  -----------------------------------------------------------------  -
Macaca mulatta CXNG             -  -----------------------------------------------------------------  -
Papio hamadryas CXNG            -  -----------------------------------------------------------------  -
Callithrix jacchus CXNG         -  -----------------------------------------------------------------  -
Microcebus murinus CXNG         -  -----------------------------------------------------------------  -
Mus musculus Cxng               -  -----------------------------------------------------------------  -
Rattus norvegicus Cxng          -  -----------------------------------------------------------------  -
Oryctolagus cuniculus CXNG      -  -----------------------------------------------------------------  -
Bos taurus CXNG                 -  -----------------------------------------------------------------  -
Equus caballus CXNG             -  -----------------------------------------------------------------  -
Canis lupus familiaris CXNG     -  -----------------------------------------------------------------  -
Felis catus CXNG                -  -----------------------------------------------------------------  -
Myotis lucifugus CXNG           -  -----------------------------------------------------------------  -
Pteropus vampyrus CXNG          -  -----------------------------------------------------------------  -
Dasypus novemcinctus CXNG       -  -----------------------------------------------------------------  -
Loxodonta africana CXNG         -  -----------------------------------------------------------------  -
Procavia capensis CXNG          -  -----------------------------------------------------------------  -
Homo sapiens CXNH1            310  D--PPPQNGQ-----KSPSRPSSSAS-K-K-------Q---YV----------------------  333
Pan troglodytes CXNH1         310  D--PPPQNGQ-----KSPSRPSSSAS-K-K-------Q---YV----------------------  333
Gorilla gorilla CXNH1         310  D--PPPQNGQ-----KSPSRPSSSAS-K-K-------Q---YV----------------------  333
Pongo abelii CXNH1            310  D--PPPQNGQ-----KSPSRPSSSAS-K-K-------Q---YV----------------------  333
Nomascus leucogenys CXNH1     310  D--PPPQNGP-----KSPSRPSSSAS-K-K-------Q---YV----------------------  333
Macaca mulatta CXNH1          310  D--PPPQNDR-----KSPSRPSSSAS-K-K-------Q---YV----------------------  333
Otolemur garnettii CXNH1      308  D--PTP-----------------------------------------------------------  311
Mus musculus Cxnh1            310  N--TAPQGGR-----KSPSRPNSSAS-K-K-------Q---YV----------------------  333
Rattus norvegicus Cxnh1       310  N--AAPQGGQ-----KSSSRPNSSAS-K-K-------Q---YV----------------------  333
Cavia porcellus CXNH1         310  D--PPPQSGH-----KYSSRPSSSAS-K-K-------Q---YV----------------------  333
Oryctolagus cuniculus CXNH1   310  D--PPPQSGR-----KSPSRPSSSAS-K-K-------Q---YV----------------------  333
Ochotona princeps CXNH1       324  D--PPPQSGR-----KSPSRPSSSAS-K-K-------Q---YV----------------------  347
Equus caballus CXNH1          310  D--PSPQSGQ-----KSPRRPDSSAS-K-K-------Q---YV----------------------  333
Canis lupus familiaris CXNH1  310  D--PPPESGQ-----KSPSRPSSSAS-K-K-------Q---YV----------------------  333
Felis catus CXNH1             310  D--PPPESGQ-----KSPSRPSSSAS-K-K-------Q---YV----------------------  333
Myotis lucifugus CXNH1        341  D--PYPQSSQ-----KY---PSSHAS-K-K-------Q---YL----------------------  361
Dasypus novemcinctus CXNH1    310  D--PPPQSVQ-----KSPSRPGSSSS-K-K-------Q---YV----------------------  333
Loxodonta africana CXNH1      310  D--PPPQSGR-----KLPSRPSSSAS-K-K-------Q---YV----------------------  333
Homo sapiens CXNI             335  H---GYHSD------KR--RLSKASS-KAR-------SDDLSV----------------------  358
Pan troglodytes CXNI          335  H---GYHSD------KR--RLSKASS-KAR-------SDDLSV----------------------  358
Pongo abelii CXNI             335  H---GYHSD------KR--RLSKASS-KAR-------SDDLSV----------------------  358
Nomascus leucogenys CXNI      335  H---GYHSD------KR--RLSKASS-KAR-------SDDLSV----------------------  358
Macaca mulatta CXNI           335  H---GYHSD------KR--RLSKASS-KAR-------SDDLSV----------------------  358
Papio hamadryas CXNI          335  H---GYHSD------KR--RLSKASS-KAR-------SDDLSV----------------------  358
Callithrix jacchus CXNI       335  H---GYHTD------KR--RLSKASS-KAR-------SDDLSV----------------------  358
Otolemur garnettii CXNI       334  H---GYHSD------KR--RLSKASS-KAR-------SDDLSV----------------------  357
Mus musculus Cxni             335  Q---GYHSD------KR--RLSKASS-KAR-------SDDLSV----------------------  358
Rattus norvegicus Cxni        367  Q---GYHSD------KR--RLSKASS-KAR-------SDDLSV----------------------  390
Cavia porcellus CXNI          335  H---GYYSE------KR--RLSKTSS-RAR-------SDDLSV----------------------  358
Oryctolagus cuniculus CXNI    335  H---GYQSD------KR--RLSKASS-KAR-------SDDLSV----------------------  358
```

```
Bos taurus CXNI                 336 H---GYQSD------KR--RLSKASS-KAR-------SDDLSV---------------------- 359
Equus caballus CXNI             335 H---CYQND------KP--RLSKASS-KAR-------SDDLSV---------------------- 358
Canis lupus familiaris CXNI     335 H---GYQSD------KR--RLSKASS-KAR-------SDDLSV---------------------- 358
Felis catus CXNI                335 H---GYQSD------KR--RLSKASS-KAR-------SDDLSV---------------------- 358
Pteropus vampyrus CXNI          335 H---SYQSD------KR--RLSKASS-KAR-------SDDLSV---------------------- 358
Erinaceus europaeus CXNI        337 Q---SYQSD------KR--RFSKASS-KAR-------SDDLSV---------------------- 360
Dasypus novemcinctus CXNI       335 Q---GYHSD------KR--RLSKASS-KAR-------SDDLSV---------------------- 358
Loxodonta africana CXNI         335 H---GYHSD------KR--RLSKASS-KAR-------SDDLSV---------------------- 358
Homo sapiens CXNJ1              409 ----LPLGDP-----GRASKASRASSGRAR-------PEDLAI---------------------- 435
Pongo abelii CXNJ1              409 ----SLLGDP-----GRASKASRASSGRAR-------PEDLAI---------------------- 435
Macaca mulatta CXNJ1            406 ----LLLGDP-----GRASKASRASGGRAR-------PEDLAI---------------------- 432
Mus musculus Cxnj1              394 ----LVLLDP-----GRSSK---SSNGRAR-------PGDLAI---------------------- 417
Rattus norvegicus Cxnj1         393 ----LVLLDP-----ERSSK---SSSGRAR-------PGDLAI---------------------- 416
Bos taurus CXNJ1                381 ----EPPADP-----GRSSKASKSSGGRAR-------AGDLAI---------------------- 407
Equus caballus CXNJ1            422 ----LPPTDP-----GRSSKASKSSGSRAR-------PDDLAI---------------------- 448
Myotis lucifugus CXNJ1            - -----------------------------------------------------------------   -
Myotis lucifugus CXNJ2            - -----------------------------------------------------------------   -
Pteropus vampyrus CXNJ1         573 ----LLPAEP-----RRSSTASKASGGRAR-------PSDLAI---------------------- 599
Pteropus vampyrus CXNJ2           - -----------------------------------------------------------------   -
Sorex araneus CXNJ1             266 -----LP-DP-----DR---VSKGSSGRAR-------NSDLAI---------------------- 287
Homo sapiens CXNK1              355 ----LAIVDQ-----QPSSRASSRASSRPQ-------PDDLEI---------------------- 381
Homo sapiens CXNK2              356 ----LAIVDQ-----RPSSRASSRASSRPR-------PDDLEI---------------------- 382
Pan troglodytes CXNK1           355 ----LAIVDQ-----QPSSRASSRASSRPQ-------PDDLEI---------------------- 381
Pan troglodytes CXNK2           356 ----LAIVDQ-----RPSSRASSRASSRPR-------PDDLEI---------------------- 382
Pongo abelii CXNK1              356 ----LAIVDQ-----RPSSRASSRASSRPR-------PDDLEI---------------------- 382
Nomascus leucogenys CXNK1       356 ----LAIVDQ-----RPSSRASSRASSRPR-------PDDLEI---------------------- 382
Callithrix jacchus CXNK1        356 ----LAIVDQ-----RPSSRASSRASSRPR-------PDDLEI---------------------- 382
Mus musculus Cxnk1              356 ----LAIVDQ-----RPSSRASSRASSRPR-------PDDLEI---------------------- 382
Mus musculus Cxnk2                - -----------------------------------------------------------------   -
Rattus norvegicus Cxnk1         356 ----LAIVDQ-----RPSSRASSRASSRPR-------PDDLEI---------------------- 382
Rattus norvegicus Cxnk2           - -----------------------------------------------------------------   -
Cavia porcellus CXNK1           356 ----LAIVDQ-----RPSSRASSRASSRPR-------PDDLEI---------------------- 382
Oryctolagus cuniculus CXNK1     356 ----LAIVDQ-----RPSSRASSRASSRPR-------PDDLEI---------------------- 382
Bos taurus CXNK1                357 ----LAIVDQ-----RPSSRASSRASSRPR-------PDDLEI---------------------- 383
Vicugna pacos CXNK1             356 ----LAIVDQ-----RPSSRASSRASSRPR-------PDDLEI---------------------- 382
Equus caballus CXNK1            356 ----LAIVDQ-----GPSSRASSRASSRPR-------PDDLEI---------------------- 382
Equus caballus CXNK2              - -----------------------------------------------------------------   -
Canis lupus familiaris CXNK1    356 ----LAIVDQ-----RPSSRASSRASSRPR-------PDDLEI---------------------- 382
Canis lupus familiaris CXNK2      - -----------------------------------------------------------------   -
Felis catus CXNK1                 - -----------------------------------------------------------------   -
Myotis lucifugus CXNK1          356 ----LAIVDQ-----RPASRASSRASSRPR-------PDDLEI---------------------- 382
Dasypus novemcinctus CXNK1        - -----------------------------------------------------------------   -
Dasypus novemcinctus CXNK2      357 ----LTLVDQ-----RPSSRASSRASSRPR-------PDDLEI---------------------- 383
Loxodonta africana CXNK1        356 ----LAIVDQ-----RPSSRASSRASSRPR-------PDDLEI---------------------- 382
Loxodonta africana CXNK2          - -----------------------------------------------------------------   -
Homo sapiens CXNL               407 E---LTTDDA-----RPLSRLSKASS-RAR-------SDDLTV---------------------- 433
Pan troglodytes CXNL            407 E---LTTDDA-----RPLSRLSKASS-RAR-------SDDLTV---------------------- 433
Pongo abelii CXNL               407 E---LTTDDA-----RPLSRLSKASS-RAR-------SDDLTV---------------------- 433
Nomascus leucogenys CXNL        407 E---QTTDDA-----RPLSRLSKASS-RAR-------SDDLTV---------------------- 433
Macaca mulatta CXNL             409 E---LTTDEA-----RPLSRLSKASS-RAR-------SDDLTV---------------------- 435
Callithrix jacchus CXNL         409 E---LTTDDA-----RPLSRLSKASS-RAR-------SDDLTV---------------------- 435
Otolemur garnettii CXNL         458 E---LTADDS-----RPLSRLSKASS-RAR-------SDDLTI---------------------- 484
Mus musculus Cxnl               414 E---LTTDDN-----RPLSRLSKASS-RAR-------SDDLTI---------------------- 440
Rattus norvegicus Cxnl          414 E---LTTDDN-----RPLSRLSKASS-RAR-------SDDLTI---------------------- 440
Cavia porcellus CXNL            414 E---LTADDT-----RPLSRLSKASS-RAR-------SDDLTI---------------------- 440
Oryctolagus cuniculus CXNL      413 E---LTSEDT-----RPLSRLSKASS-RAR-------SDDLTI---------------------- 439
Ochotona princeps CXNL          411 E---LTTDDI-----RPLSRLSKASS-RAR-------SDDLTI---------------------- 437
Bos taurus CXNL                 414 E---LPGDDT-----RPLSRLSKTSS-RAR-------SDDLTV---------------------- 440
Equus caballus CXNL             415 E---LSGDDT-----RPLSRLSKASS-RAR-------SDDLTI---------------------- 441
Canis lupus familiaris CXNL     413 D---LTRDDT-----RPLSRLSKASS-RAR-------SDDLTV---------------------- 439
Felis catus CXNL                414 D---PSRDDT-----RPLSRLSKTSS-RAR-------SDDLTV---------------------- 440
Pteropus vampyrus CXNL          414 E---LTGDDT-----RPLSRLSKASS-RAR-------SDDLTV---------------------- 440
Sorex araneus CXNL              353 E---LRQEVP-----RDLSRPS-PTS-RAR-------PDDLTV---------------------- 378
Dasypus novemcinctus CXNL       416 E---STSDDT-----RPLSRVSKTSS-RAR-------SDDLTV---------------------- 442
Loxodonta africana CXNL         411 E---VTTDET-----RPLSRLSKASS-RAR-------SDDLTV---------------------- 437
Homo sapiens CXNM               417 WK-PRWLRAT----WGSSTEHENR-GSPPKG------NLKGQFRKGTVRTLP-P-SQGDSQSLDI 467
Pan troglodytes CXNM            417 WK-PRWLRAT----WGSSTEHENR-GSPPNG------NLKGQFRKGTVRTLP-P-SQGDSQSLDI 467
Pongo abelii CXNM               416 WK-PRWLRAT----WGSSTEHENR-GSPPKG------NLKGQFRKGTVRTLP-P-SQGQSQSLDI 466
Nomascus leucogenys CXNM        417 WK-PRWLRAT----WGSSTEPENR-GSPPKG------NLKGQFRKGTVRTLP-P-SQGESQSLDI 467
Macaca mulatta CXNM             417 WK-PRWLRAT----WGSSTEHENQ-GSPPKG------NLKGQFRKGIVRTLP-P-SQGKSQSLDI 467
Callithrix jacchus CXNM         416 WK-PRWLRAT----WAFSKEDENC-KSPPKG------NLKGQFREGTVRSLS-P-SQRESQSIDI 466
Tarsius syrichta CXNM           410 WK-PRWLRTT----WSHSTEDKNW-GSPPKG------NLKGQFREDTIRTLS-P-SQGDSQSLDI 460
Microcebus murinus CXNM         415 WK-PKWIRAT----WDPSTEEENR-GSPSKG------NLEGQFREGTIRTLP-S-SQGDSHSLDI 465
Dipodomys ordii CXNM            361 -------------------------------------------------------QGGNSQ--PV 368
Oryctolagus cuniculus CXNM      410 WK-PRWLSAT----RGPSTEGGNW-RSPPNGHLIPNGNLEGQFLEGTTSTFP-P-SQGNSQLLPV 466
Equus caballus CXNM             416 WK-PRWLRAT----WGPATEDEKQ-ASPPKGT-----NIKGQLREGTIRTLP-P-SQGDFQPLDI 467
Canis lupus familiaris CXNM     415 WE-QSWLSTT----WGPSPEEENQ-GSPPKG------NLKGQCREGTIRTLP-P-SQGDCQAPDI 465
Pteropus vampyrus CXNM          412 ET-PRWLCAT----LSPSIEDENW-ESPPKG------NLKGQFRDGTIRTLS-P-LQGDFQPLNI 462
Loxodonta africana CXNM         410 WK-PKRLSAT----WRPFTEDQNQ-RLPPKG------SLQGPFGEGTIRTLP-P-LQGDSRPLDI 460
```

Homo sapiens CXNN	408	DS-EGSMRESGV--WIDRSRPGSRKAS--FL------S-RL-LSE-K-RHLH-SDS-GSSGSRNS	455
Pan troglodytes CXNN	408	DS-EGSMRESGV--WIDRSRPGSRKAS--FL------S-RL-LSE-K-RHLH-SDS-GSSGSRNS	455
Pongo abelii CXNN	408	DS-EGSMRESGV--WIDRSRPGSRKAS--FL------S-RL-LSE-K-QHLH-SDS-GSSGSRNS	455
Nomascus leucogenys CXNN	408	DS-EGSMRESGV--WIDRSRPGSRKAS--FL------S-RL-LSE-K-RHLH-SDS-GSSGSRNS	455
Macaca mulatta CXNN	408	DS-EGSMRESGV--WIERSRPGSRKAS--FL------S-RL-LSE-K-RHLH-SDS-GSSGSQNS	455
Callithrix jacchus CXNN	409	DS-EGSMRESGV--WIDRSHPGSHKGS--FL------S-RL-LSE-K-RHLH-SDS-GSSSSQNS	456
Microcebus murinus CXNN	406	DS-DGSMRESGV--WIDRSRSGSRKAS--FL------S-RL-LSE-K-GQLH-SDS-GSSGTQNS	453
Mus musculus Cxnn	408	GS-DGSARESGV--WTDRLGPGSRKAS--FL------S-RL-MSE-K-GQRH-SDS-GSSRSLNS	455
Rattus norvegicus Cxnn	408	GS-DGSARESGV--WTDRLGPGSRKAS--FL------S-RL-ISE-K-GQQH-SDS-GSSRSLNS	455
Oryctolagus cuniculus CXNN	435	---DSSVRESGG--WVDKSRPGSRKAS--FL------S-RL-LSE-K-AQLQ-SDL-GSSSSQNS	480
Bos taurus CXNN	408	HS-DGSMTDSRVQEARDRSYPGSRKAS--FL------S-RL-FSE-K-GQLY-SDS-GSSTSRNS	457
Equus caballus CXNN	407	DS-DGSVRESGV--WTDRSRSGSRKAS--FL------S-RL-LSE-K-GQLP-NDS-GSSSSRNS	454
Canis lupus familiaris CXNN	407	DS-NGSVRKSGV--WTDRSYPGSRKAS--FL------S-RL-LSE-K-GQLH-SDS-GSSNSRNS	454
Sorex araneus CXNN	407	DS-EGSVRESGV--WMDRSCPASRKAS--FL------S-RV-MSE-K-GQLH-SDS-GSSGSQKN	454
Loxodonta africana CXNN	409	DS-DGSVRESGV--WIDRSRPGSRKAS--FL------S-RL-LSE-K-GQPH-SDS-GSSSSRNS	456
Homo sapiens CXNO	410	GTHERPGAKP-----RAGSEKGSASS-R-------DGKTTVWI----------------------	439
Papio hamadryas CXNO	412	GTHERPGAKP-----RAGSEKGSASS-R-------DGKTTVWI----------------------	441
Mus musculus Cxno	422	GAQEQLATKP-----RAGSEKGSTGS-R-------DGKATVWI----------------------	451
Cavia porcellus CXNO	398	--------KP-----KAGSEQGSGGS-R-------DGKTTVWI----------------------	419
Homo sapiens CXNP1	220	---------------RTWKHKSS-SS-K-Y----FL--TSEST---R-RHKKATDSLPVVE-TKE	255
Pan troglodytes CXNP1	220	---------------RTWKHKSS-SS-K-Y----FP--TSEST---R-RHKKATDSLPVVE-TKE	255
Pongo abelii CXNP1	220	---------------RTWKHKSS-SS-K-H----FP--TSERT---R-RHKKATDSLPVVE-TKE	255
Callithrix jacchus CXNP1	221	---------------RTRNTNLP-NA-S-R----LQ--RAPED---T-RDQPITSQWWKPK-S--	254
Otolemur garnettii CXNP1	221	---------------ETQKHNTS-SS-N-Y----CP--TSEST---R-RCKEPTGNFPVVE-TKE	256
Tupaia belangeri CXNP1	238	---------------RTQKHKSP-SS-K-H----FL--TLESI---R-RHKKPTDNFPEVE-TKE	273
Mus musculus Cxnp1	221	---------------RIYKHKLS-FL-K-K----LP--TSESS---V-RSKDTTDELSVVE-AKE	256
Rattus norvegicus Cxnp1	220	---------------KTYRHKLP-LF-K-N----LS--TSERS---V-RHKDTTDDLSVVE-TKE	255
Cavia porcellus CXNP1	221	---------------KIQRHRFL-SS-N-C----FS--TSEST---T-RQKEPTNTFPVVE-TKE	256
Oryctolagus cuniculus CXNP1	221	---------------ERLRKFLG-VS-S-S-----P--SHTLP---L-DSVRKLQETP-------	249
Oryctolagus cuniculus CXNP2	221	---------------RSRKHQSS-SS-N-Y----FP--TSEST---R-SHKEPTNTFPVME-TKQ	256
Ochotona princeps CXNP1	221	---------------RIRKQRSS-SS-N-Y----FP--PSESA---K-SHKEPTNTIPGME-TKQ	256
Bos taurus CXNP1	222	---------------RIWRHKSP-SS-N-Y----SP--TSQSA---K-RCKAPTDNFPVVE-IRE	257
Equus caballus CXNP1	221	---------------RTRKHKSP-SS-N-Y----FP--TSEST---R-RHKEPTDKFLVVE-TKE	256
Canis lupus familiaris CXNP1	219	---------------RTWKHKSP--S-N-Y----SS--TSEST---K-KHKDPTDNFPAVE-AKE	253
Felis catus CXNP1	242	---------------RARKHRPP-PS-N-Y----SP--TSEST---R-RHKEPTENFPVVE-TKE	277
Myotis lucifugus CXNP1	221	---------------QTWKQKSS-SS-N-Y----FT--TSETT---R-KHKEPNDNFPVVE-SKE	256
Dasypus novemcinctus CXNP1	219	---------------RPLG-KFL-GV-S-L----SS--SHN----------------PVPGYTK-	241
Dasypus novemcinctus CXNP2	219	---------------RPLG-KFL-GV-S-L----SS--SHY----------------PVPGYTK-	241
Dasypus novemcinctus CXNP3	220	---------------RTLKHK---CS-N-Y----FP--TSKSP---R-RHKEPT-NCPVVG-TKE	252
Dasypus novemcinctus CXNP4	219	---------------RPLG-KFL-GV-S-L----SS--SHN----------------PVPGYTK-	241
Choloepus hoffmanni CXNP1	219	---------------RGLG-NLL-GV-S-F----PS--SHT----------------PAPAAPK-	241
Loxodonta africana CXNP1	-	---	-
Homo sapiens CXNQ	367	EKKAKVGSK-------AGSNKSTASS-K-S----GDGKTSVWI----------------------	396
Pan troglodytes CXNQ	367	EKKAKVGSK-------AGSNKSTASS-K-S----GDGKTSVWI----------------------	396
Pongo abelii CXNQ	367	EKKAKVGSK-------AGSNKSTASS-K-S----GDGKTSVWI----------------------	396
Macaca mulatta CXNQ	367	EKKAKVGSK-------AGSNKSTASS-K-S----GDGKTSVWI----------------------	396
Tupaia belangeri CXNQ	367	EKKAKVGSK-------AGSNKSSASS-K-S----GDGKTSVWI----------------------	396
Mus musculus Cxnq	367	EKKAKVGSK-------SGSNKSSISS-K-S----GDGKTSVWI----------------------	396
Rattus norvegicus Cxnq	367	EKKAKVGSK-------SGSNKSSISS-K-S----GDGKTSVWI----------------------	396
Cavia porcellus CXNQ	367	EKKAR-----------TGSNKSSASS-K-S----GDGKTSVWI----------------------	392
S.tridecemlineatus CXNQ	381	EKKAKVGSK-------AGSNKSSVSS-K-S----GDGKTSVWI----------------------	410
Oryctolagus cuniculus CXNQ	367	EKRAKVGSK-------AGSNKSSASS-K-S----GDGKTSVWI----------------------	396
Bos taurus CXNQ	367	EKKAKVGSK-------AGSNKSSASS-K-S----GDGKTSVWI----------------------	396
Vicugna pacos CXNQ	367	EKKAKVGSK-------AGSNKSSASS-K-S----GDGKTSVWI----------------------	396
Equus caballus CXNQ	367	GKKAKVGSK-------AGSNKSSASS-K-S----GDGKTSVWI----------------------	396
Canis lupus familiaris CXNQ	367	EKKAKVGSK-------AGSNKSSASS-K-S----GDGKTSVWI----------------------	396
Myotis lucifugus CXNQ	367	EQKAKVGSK-------AGSNKSSTSS-K-S----GNGKTSVWI----------------------	396
Pteropus vampyrus CXNQ	367	GKKAKVGSK-------AGSNKSSASS-K-S----GNGKTSVWI----------------------	396
Loxodonta africana CXNQ	367	EKRAKV-----------GSNKSSASS-K-S----GDVKTSVWI----------------------	392
Homo sapiens CXNR	-	---	-
Pan troglodytes CXNR	-	---	-
Pongo abelii CXNR	-	---	-
Papio hamadryas CXNR	-	---	-
Mus musculus Cxnr	-	---	-
Rattus norvegicus Cxnr	-	---	-
Oryctolagus cuniculus CXNR	-	---	-
Bos taurus CXNR	-	---	-
Homo sapiens CXNS	258	---------------NFGRTQSS-DS-A-Y----V------------------------------	270
Pan troglodytes CXNS	258	---------------NFGRTQSS-DS-A-Y----V------------------------------	270
Pongo abelii CXNS	258	---------------NFGRTQSS-DS-A-Y----V------------------------------	270
Nomascus leucogenys CXNS	258	---------------NFGRTQSS-DS-A-Y----V------------------------------	270
Macaca mulatta CXNS	258	---------------NFGRTQSS-DS-A-Y----V------------------------------	270
Papio hamadryas CXNS	258	---------------NFGRTQSS-DS-A-Y----V------------------------------	270
Otolemur garnettii CXNS	258	---------------NFGRTQSS-DS-A-Y----V------------------------------	270
Tupaia belangeri CXNS	258	---------------NFGRTQSS-DS-A-Y----V------------------------------	270
Mus musculus Cxns	298	---------------NFGRTQSS-DS-A-Y----V------------------------------	310
Rattus norvegicus Cxns	258	---------------NFGRTQSS-DS-A-Y----V------------------------------	270
Cavia porcellus CXNS	258	---------------NFGRTQSS-DS-A-Y----V------------------------------	270
S.tridecemlineatus CXNS	258	---------------NFGRTQSS-DS-A-Y----V------------------------------	270

```
Oryctolagus cuniculus CXNS   258 ---------------NFGRTQSS-DS-A-Y----V------------------------------ 270
Ochotona princeps CXNS       258 ---------------NFGRTQSS-DS-A-Y----V------------------------------ 270
Bos taurus CXNS              258 ---------------NFGRTQSS-DS-A-Y----V------------------------------ 270
Equus caballus CXNS          258 ---------------NFGRTQSS-DS-A-Y----V------------------------------ 270
Canis lupus familiaris CXNS  258 ---------------NFGRTQSS-DS-A-Y----V------------------------------ 270
Myotis lucifugus CXNS        258 ---------------NFGRTQSS-DS-A-Y----V------------------------------ 270
Dasypus novemcinctus CXNS    258 ---------------NFGRTQSS-DS-A-Y----V------------------------------ 270
Loxodonta africana CXNS      386 ---------------NFGRTQSS-DS-A-Y----V------------------------------ 398
Homo sapiens CXNT              - -----------------------------------------------------------------   -
Pan troglodytes CXNT           - -----------------------------------------------------------------   -
Nomascus leucogenys CXNT       - -----------------------------------------------------------------   -
Mus musculus Cxnt              - -----------------------------------------------------------------   -
Rattus norvegicus Cxnt         - -----------------------------------------------------------------   -
Cavia porcellus CXNT           - -----------------------------------------------------------------   -
Oryctolagus cuniculus CXNT     - -----------------------------------------------------------------   -
Bos taurus CXNT                - -----------------------------------------------------------------   -
Vicugna pacos CXNT             - -----------------------------------------------------------------   -
Equus caballus CXNT            - -----------------------------------------------------------------   -
Canis lupus familiaris CXNT    - -----------------------------------------------------------------   -
Myotis lucifugus CXNT          - -----------------------------------------------------------------   -
Dasypus novemcinctus CXNT      - -----------------------------------------------------------------   -
Loxodonta africana CXNT        - -----------------------------------------------------------------   -
Homo sapiens CXNU            330 CSSLQPPDPP----------ASSSG--APH----LRARKSEWV---------------------- 356
Pan troglodytes CXNU         330 CSSLQPPDPP----------ASSGG--APH----LRARKSEWV---------------------- 356
Pongo abelii CXNU            315 CSSLQPPDLP----------ASSGG--APH----LRARKSEWV---------------------- 341
Nomascus leucogenys CXNU     327 CSRLQPPDPP----------ASSGG--APH----RRARKSEWV---------------------- 353
Macaca mulatta CXNU          330 CSRLQPPDPP----------ASSVG--APH----LRARKSEWV---------------------- 356
Mus musculus Cxnu            316 ASKPQAPCRL----------TTSGS--APH----LRTKKSEWV---------------------- 342
Cavia porcellus CXNU         309 SGLLQSPQQL----------VPSGS--VPH----LRTKKSEWV---------------------- 335
Bos taurus CXNU              302 --------------------------------------KSEWV---------------------- 306
Equus caballus CXNU          327 SSPLQPPARP----------GPAGS--VPV----LRTRRSEWV---------------------- 353
Myotis lucifugus CXNU        327 SSLWQPSTRQ----------AGAGS--APL----LGTRRSEWV---------------------- 353
Sorex araneus CXNU           299 -----PSARP----------GSSRS--APH----LRTRKSEWV---------------------- 320
Dasypus novemcinctus CXNU    345 PRQLQPSPLL----------ANSGS--ATH----LRTKKSEWV---------------------- 371
Loxodonta africana CXNU      376 STELQPPDRL----------SKSGS--ATH----LRTKRSEWV---------------------- 402
```

```
Homo sapiens CXNA                  - ----------------------------------------------------------------- -
                                           920       930       940       950       960       970
Homo sapiens CXNA                  - ----------------------------------------------------------------- -
Pan troglodytes CXNA               - ----------------------------------------------------------------- -
Gorilla gorilla CXNA               - ----------------------------------------------------------------- -
Pongo abelii CXNA                  - ----------------------------------------------------------------- -
Nomascus leucogenys CXNA           - ----------------------------------------------------------------- -
Macaca mulatta CXNA                - ----------------------------------------------------------------- -
Callithrix jacchus CXNA            - ----------------------------------------------------------------- -
Mus musculus Cxna                  - ----------------------------------------------------------------- -
Rattus norvegicus Cxna             - ----------------------------------------------------------------- -
Cavia porcellus CXNA               - ----------------------------------------------------------------- -
Ochotona princeps CXNA             - ----------------------------------------------------------------- -
Bos taurus CXNA                    - ----------------------------------------------------------------- -
Equus caballus CXNA                - ----------------------------------------------------------------- -
Canis lupus familiaris CXNA        - ----------------------------------------------------------------- -
Felis catus CXNA                   - ----------------------------------------------------------------- -
Myotis lucifugus CXNA              - ----------------------------------------------------------------- -
Dasypus novemcinctus CXNA          - ----------------------------------------------------------------- -
Loxodonta africana CXNA            - ----------------------------------------------------------------- -
Homo sapiens CXNB                  - ----------------------------------------------------------------- -
Gorilla gorilla CXNB               - ----------------------------------------------------------------- -
Nomascus leucogenys CXNB           - ----------------------------------------------------------------- -
Macaca mulatta CXNB                - ----------------------------------------------------------------- -
Callithrix jacchus CXNB            - ----------------------------------------------------------------- -
Otolemur garnettii CXNB            - ----------------------------------------------------------------- -
Mus musculus Cxnb                  - ----------------------------------------------------------------- -
Rattus norvegicus Cxnb             - ----------------------------------------------------------------- -
Dipodomys ordii CXNB               - ----------------------------------------------------------------- -
Cavia porcellus CXNB               - ----------------------------------------------------------------- -
Oryctolagus cuniculus CXNB         - ----------------------------------------------------------------- -
Ochotona princeps CXNB             - ----------------------------------------------------------------- -
Bos taurus CXNB                    - ----------------------------------------------------------------- -
Equus caballus CXNB                - ----------------------------------------------------------------- -
Canis lupus familiaris CXNB        - ----------------------------------------------------------------- -
Myotis lucifugus CXNB              - ----------------------------------------------------------------- -
Erinaceus europaeus CXNB           - ----------------------------------------------------------------- -
Loxodonta africana CXNB            - ----------------------------------------------------------------- -
Homo sapiens CXNC                  - ----------------------------------------------------------------- -
Gorilla gorilla CXNC               - ----------------------------------------------------------------- -
Pongo abelii CXNC                  - ----------------------------------------------------------------- -
Nomascus leucogenys CXNC           - ----------------------------------------------------------------- -
Callithrix jacchus CXNC            - ----------------------------------------------------------------- -
Microcebus murinus CXNC            - ----------------------------------------------------------------- -
Otolemur garnettii CXNC            - ----------------------------------------------------------------- -
Mus musculus Cxnc                  - ----------------------------------------------------------------- -
Rattus norvegicus Cxnc             - ----------------------------------------------------------------- -
Cavia porcellus CXNC               - ----------------------------------------------------------------- -
Oryctolagus cuniculus CXNC         - ----------------------------------------------------------------- -
Bos taurus CXNC                    - ----------------------------------------------------------------- -
Equus caballus CXNC                - ----------------------------------------------------------------- -
Canis lupus familiaris CXNC        - ----------------------------------------------------------------- -
Felis catus CXNC                   - ----------------------------------------------------------------- -
Myotis lucifugus CXNC              - ----------------------------------------------------------------- -
Dasypus novemcinctus CXNC          - ----------------------------------------------------------------- -
Loxodonta africana CXNC            - ----------------------------------------------------------------- -
Homo sapiens CXND                  - ----------------------------------------------------------------- -
Pan troglodytes CXND               - ----------------------------------------------------------------- -
Macaca mulatta CXND                - ----------------------------------------------------------------- -
Callithrix jacchus CXND            - ----------------------------------------------------------------- -
Dipodomys ordii CXND               - ----------------------------------------------------------------- -
Oryctolagus cuniculus CXND         - ----------------------------------------------------------------- -
Tursiops truncatus CXND            - ----------------------------------------------------------------- -
Bos taurus CXND                    - ----------------------------------------------------------------- -
Equus caballus CXND                - ----------------------------------------------------------------- -
Canis lupus familiaris CXND        - ----------------------------------------------------------------- -
Myotis lucifugus CXND              - ----------------------------------------------------------------- -
Erinaceus europaeus CXND           - ----------------------------------------------------------------- -
Dasypus novemcinctus CXND          - ----------------------------------------------------------------- -
Choloepus hoffmanni CXND           - ----------------------------------------------------------------- -
Loxodonta africana CXND            - ----------------------------------------------------------------- -
Homo sapiens CXNE                  - ----------------------------------------------------------------- -
Pan troglodytes CXNE               - ----------------------------------------------------------------- -
Pongo abelii CXNE                  - ----------------------------------------------------------------- -
Macaca mulatta CXNE                - ----------------------------------------------------------------- -
Papio hamadryas CXNE               - ----------------------------------------------------------------- -
Callithrix jacchus CXNE            - ----------------------------------------------------------------- -
Otolemur garnettii CXNE            - ----------------------------------------------------------------- -
Mus musculus Cxne                  - ----------------------------------------------------------------- -
```

```
Rattus norvegicus Cxne          - -----------------------------------------------------------------   -
Dipodomys ordii CXNE            - -----------------------------------------------------------------   -
Cavia porcellus CXNE            - -----------------------------------------------------------------   -
Oryctolagus cuniculus CXNE      - -----------------------------------------------------------------   -
Equus caballus CXNE             - -----------------------------------------------------------------   -
Canis lupus familiaris CXNE     - -----------------------------------------------------------------   -
Felis catus CXNE                - -----------------------------------------------------------------   -
Myotis lucifugus CXNE           - -----------------------------------------------------------------   -
Sorex araneus CXNE              - -----------------------------------------------------------------   -
Dasypus novemcinctus CXNE       - -----------------------------------------------------------------   -
Loxodonta africana CXNE         - -----------------------------------------------------------------   -
Homo sapiens CXNF               - -----------------------------------------------------------------   -
Pan troglodytes CXNF            - -----------------------------------------------------------------   -
Gorilla gorilla CXNF            - -----------------------------------------------------------------   -
Nomascus leucogenys CXNF        - -----------------------------------------------------------------   -
Callithrix jacchus CXNF         - -----------------------------------------------------------------   -
Mus musculus Cxnf               - -----------------------------------------------------------------   -
Rattus norvegicus Cxnf          - -----------------------------------------------------------------   -
S.tridecemlineatus CXNF         - -----------------------------------------------------------------   -
Oryctolagus cuniculus CXNF      - -----------------------------------------------------------------   -
Ochotona princeps CXNF          - -----------------------------------------------------------------   -
Bos taurus CXNF                 - -----------------------------------------------------------------   -
Equus caballus CXNF             - -----------------------------------------------------------------   -
Canis lupus familiaris CXNF     - -----------------------------------------------------------------   -
Myotis lucifugus CXNF           - -----------------------------------------------------------------   -
Dasypus novemcinctus CXNF       - -----------------------------------------------------------------   -
Choloepus hoffmanni CXNF        - -----------------------------------------------------------------   -
Loxodonta africana CXNF         - -----------------------------------------------------------------   -
Homo sapiens CXNG               - -----------------------------------------------------------------   -
Pan troglodytes CXNG            - -----------------------------------------------------------------   -
Gorilla gorilla CXNG            - -----------------------------------------------------------------   -
Pongo abelii CXNG               - -----------------------------------------------------------------   -
Nomascus leucogenys CXNG        - -----------------------------------------------------------------   -
Macaca mulatta CXNG             - -----------------------------------------------------------------   -
Papio hamadryas CXNG            - -----------------------------------------------------------------   -
Callithrix jacchus CXNG         - -----------------------------------------------------------------   -
Microcebus murinus CXNG         - -----------------------------------------------------------------   -
Mus musculus Cxng               - -----------------------------------------------------------------   -
Rattus norvegicus Cxng          - -----------------------------------------------------------------   -
Oryctolagus cuniculus CXNG      - -----------------------------------------------------------------   -
Bos taurus CXNG                 - -----------------------------------------------------------------   -
Equus caballus CXNG             - -----------------------------------------------------------------   -
Canis lupus familiaris CXNG     - -----------------------------------------------------------------   -
Felis catus CXNG                - -----------------------------------------------------------------   -
Myotis lucifugus CXNG           - -----------------------------------------------------------------   -
Pteropus vampyrus CXNG          - -----------------------------------------------------------------   -
Dasypus novemcinctus CXNG       - -----------------------------------------------------------------   -
Loxodonta africana CXNG         - -----------------------------------------------------------------   -
Procavia capensis CXNG          - -----------------------------------------------------------------   -
Homo sapiens CXNH1              - -----------------------------------------------------------------   -
Pan troglodytes CXNH1           - -----------------------------------------------------------------   -
Gorilla gorilla CXNH1           - -----------------------------------------------------------------   -
Pongo abelii CXNH1              - -----------------------------------------------------------------   -
Nomascus leucogenys CXNH1       - -----------------------------------------------------------------   -
Macaca mulatta CXNH1            - -----------------------------------------------------------------   -
Otolemur garnettii CXNH1        - -----------------------------------------------------------------   -
Mus musculus Cxnh1              - -----------------------------------------------------------------   -
Rattus norvegicus Cxnh1         - -----------------------------------------------------------------   -
Cavia porcellus CXNH1           - -----------------------------------------------------------------   -
Oryctolagus cuniculus CXNH1     - -----------------------------------------------------------------   -
Ochotona princeps CXNH1         - -----------------------------------------------------------------   -
Equus caballus CXNH1            - -----------------------------------------------------------------   -
Canis lupus familiaris CXNH1    - -----------------------------------------------------------------   -
Felis catus CXNH1               - -----------------------------------------------------------------   -
Myotis lucifugus CXNH1          - -----------------------------------------------------------------   -
Dasypus novemcinctus CXNH1      - -----------------------------------------------------------------   -
Loxodonta africana CXNH1        - -----------------------------------------------------------------   -
Homo sapiens CXNI               - -----------------------------------------------------------------   -
Pan troglodytes CXNI            - -----------------------------------------------------------------   -
Pongo abelii CXNI               - -----------------------------------------------------------------   -
Nomascus leucogenys CXNI        - -----------------------------------------------------------------   -
Macaca mulatta CXNI             - -----------------------------------------------------------------   -
Papio hamadryas CXNI            - -----------------------------------------------------------------   -
Callithrix jacchus CXNI         - -----------------------------------------------------------------   -
Otolemur garnettii CXNI         - -----------------------------------------------------------------   -
Mus musculus Cxni               - -----------------------------------------------------------------   -
Rattus norvegicus Cxni          - -----------------------------------------------------------------   -
Cavia porcellus CXNI            - -----------------------------------------------------------------   -
Oryctolagus cuniculus CXNI      - -----------------------------------------------------------------   -
```

Bos taurus CXNI	-	---	-
Equus caballus CXNI	-	---	-
Canis lupus familiaris CXNI	-	---	-
Felis catus CXNI	-	---	-
Pteropus vampyrus CXNI	-	---	-
Erinaceus europaeus CXNI	-	---	-
Dasypus novemcinctus CXNI	-	---	-
Loxodonta africana CXNI	-	---	-
Homo sapiens CXNJ1	-	---	-
Pongo abelii CXNJ1	-	---	-
Macaca mulatta CXNJ1	-	---	-
Mus musculus Cxnj1	-	---	-
Rattus norvegicus Cxnj1	-	---	-
Bos taurus CXNJ1	-	---	-
Equus caballus CXNJ1	-	---	-
Myotis lucifugus CXNJ1	-	---	-
Myotis lucifugus CXNJ2	-	---	-
Pteropus vampyrus CXNJ1	-	---	-
Pteropus vampyrus CXNJ2	-	---	-
Sorex araneus CXNJ1	-	---	-
Homo sapiens CXNK1	-	---	-
Homo sapiens CXNK2	-	---	-
Pan troglodytes CXNK1	-	---	-
Pan troglodytes CXNK2	-	---	-
Pongo abelii CXNK1	-	---	-
Nomascus leucogenys CXNK1	-	---	-
Callithrix jacchus CXNK1	-	---	-
Mus musculus Cxnk1	-	---	-
Mus musculus Cxnk2	-	---	-
Rattus norvegicus Cxnk1	-	---	-
Rattus norvegicus Cxnk2	-	---	-
Cavia porcellus CXNK1	-	---	-
Oryctolagus cuniculus CXNK1	-	---	-
Bos taurus CXNK1	-	---	-
Vicugna pacos CXNK1	-	---	-
Equus caballus CXNK1	-	---	-
Equus caballus CXNK2	-	---	-
Canis lupus familiaris CXNK1	-	---	-
Canis lupus familiaris CXNK2	-	---	-
Felis catus CXNK1	-	---	-
Myotis lucifugus CXNK1	-	---	-
Dasypus novemcinctus CXNK1	-	---	-
Dasypus novemcinctus CXNK2	-	---	-
Loxodonta africana CXNK1	-	---	-
Loxodonta africana CXNK2	-	---	-
Homo sapiens CXNL	-	---	-
Pan troglodytes CXNL	-	---	-
Pongo abelii CXNL	-	---	-
Nomascus leucogenys CXNL	-	---	-
Macaca mulatta CXNL	-	---	-
Callithrix jacchus CXNL	-	---	-
Otolemur garnettii CXNL	-	---	-
Mus musculus Cxnl	-	---	-
Rattus norvegicus Cxnl	-	---	-
Cavia porcellus CXNL	-	---	-
Oryctolagus cuniculus CXNL	-	---	-
Ochotona princeps CXNL	-	---	-
Bos taurus CXNL	-	---	-
Equus caballus CXNL	-	---	-
Canis lupus familiaris CXNL	-	---	-
Felis catus CXNL	-	---	-
Pteropus vampyrus CXNL	-	---	-
Sorex araneus CXNL	-	---	-
Dasypus novemcinctus CXNL	-	---	-
Loxodonta africana CXNL	-	---	-
Homo sapiens CXNM	468	P-NTADSLGGLSFEPGLVRTCNNP-VCP-PNHVVSLTNNLIGRR-V-P-TD-L-Q-I--------	515
Pan troglodytes CXNM	468	P-NTADSLGGLSFEPGLVRTCNNP-VCP-PNHVVSLTNNLIGRR-V-P-TD-L-Q-I--------	515
Pongo abelii CXNM	467	P-NTADSLGGLSFEPGLVRTCNNP-VCP-PNHVVSLTNNLIGRR-V-P-TD-L-Q-I--------	514
Nomascus leucogenys CXNM	468	P-NTADSLGGLSFEPGLVRTCNNP-VCP-PNHAVSLTNNLIGRR-V-P-TD-L-Q-I--------	515
Macaca mulatta CXNM	468	P-NTADSLGGLSFELGLVRTCNNP-VCP-PNHVVSLTNNLIGRR-V-P-TD-L-Q-I--------	515
Callithrix jacchus CXNM	467	P-NIADSLGELSFEPGLVRTCNNP-VCP-TNHVVSLRNNLISRR-V-P-TD-L-Q-I--------	514
Tarsius syrichta CXNM	461	P-NTPDSLGELSFELESVRTCNKPAVSP-PNHMASLMNNFIGRR-V-P-TD-L-Q-I--------	509
Microcebus murinus CXNM	466	L-NTPDSSGELSFEPELVRTCNNPTVCS-SNHIVSLMNNLTGRR-V-P-TD-L-R-I--------	514
Dipodomys ordii CXNM	369	--DTPDSLGQLSFEPKSVRTYNNP---P-S-KSVSLRNNFIGRR-V-S-TD-L-Q-I--------	412
Oryctolagus cuniculus CXNM	467	P-QGPCSLREPSCEPGLVRPCNNP-VCP-PNPAVSLTNSLAGRR-L-P-TD-L-Q-I--------	514
Equus caballus CXNM	468	P-DTPDSLGELSFESKSVRTCNNPTACP-PNRLVLLANNLSGRR-A-P-TD-L-Q-I--------	516
Canis lupus familiaris CXNM	466	S-DTPDSLGQLTFDSDLVRGCNNPTACP-PNHLVLLTNHLTGRR-A-P-TD-L-Q-I--------	514
Pteropus vampyrus CXNM	463	P-DTPNSLGELSFGSEFVRTCNNPTTCP-PNHLVLLTNNLISRR-A-P-TD-L-Q-I--------	511
Loxodonta africana CXNM	461	P-GTPDSWGELSFETKFVRTCKNPSAYP-SNHLVSLTNNVIGRR-A-P-TD-L-Q-I--------	509

```
Homo sapiens CXNN              456 SC--LD-F--PHWE-------NSPS--PLPS-VTGHRTS-MVRQAALPIME-LSQELFH-SGCFL 502
Pan troglodytes CXNN           456 FC--LD-F--PHWE-------NSPS--PLPS-VTGHRTS-MVRQTALPIME-LSQELFH-SGCFL 502
Pongo abelii CXNN              456 SC--LD-L--PHWE-------NSPS--PLPS-VTGHRTS-MVRQTALPIMQ-LSQELFH-SECFL 502
Nomascus leucogenys CXNN       456 SC--LD-F--PHWE-------NSPS--PLPS-VTGHRTS-MVRQTALPIME-LSQELFH-SGCFL 502
Macaca mulatta CXNN            456 SC--LD-F--PHWE-------NSPS--PLPS-VPGHRTS-MVRQTALPIME-LSQKLFH-PGCFL 502
Callithrix jacchus CXNN        457 SC--LD-F--PHRE-------NSSS--PLPS-VTGHRTS-MVRQTAIPIME-LSQELFQ-SGYFF 503
Microcebus murinus CXNN        454 SC--LD-C--PHRE-------NSPS--PLPS-ATGHRTS-MVRQTDLLIIE-LSQELWPL---FP 498
Mus musculus Cxnn              456 SC--LD-F--SHGE-------NSPS--PLPS-ATGHRAS-MVSKSSHVDSPPHSS--FIIHETYV 502
Rattus norvegicus Cxnn         456 SC--LD-F--PHGE-------NSPS--PLPS-ATGHRAS-MVSKSSHVDSPSVSS-PFIICGTYV 503
Oryctolagus cuniculus CXNN     481 SR--LD-F--QHQE-------NSPS--PAPS-ITGQRTS-MVSKDTSPADYGTITRGFPLRLPLS 529
Bos taurus CXNN                458 SC--QG-F--PHRE-------NSPS--LLPA-APGRRTS-MVSKVRQSD---------------- 490
Equus caballus CXNN            455 SS--LD-F--PHRE-------NSPS--LLPS-AAGHRTS-MVSKDKQPD---------------- 487
Canis lupus familiaris CXNN    455 SC--LD-L--LHRE-------NSPS--LLPS-ATGYRTS-MVSKDRQLD---------------- 487
Sorex araneus CXNN             455 SC--VD-S--PYLD-------NSPS--LLPS-ATGRRRS-MVSKGRLNQPDWS------------ 491
Loxodonta africana CXNN        457 SC--LD-I--PHRE-------NSPS--PLPS-ATGHRTS-MVSKNRQPY---------------- 489
Homo sapiens CXNO                - -----------------------------------------------------------------   -
Papio hamadryas CXNO             - -----------------------------------------------------------------   -
Mus musculus Cxno                - -----------------------------------------------------------------   -
Cavia porcellus CXNO             - -----------------------------------------------------------------   -
Homo sapiens CXNP1             256 Q--FQEA-GEKDT---L-SSCH------------------------------------------- 270
Pan troglodytes CXNP1          256 Q--FQEA-GEKDT---L-SSCH------------------------------------------- 270
Pongo abelii CXNP1             256 Q--FQEA-GEKDT---L-SSCN------------------------------------------- 270
Callithrix jacchus CXNP1       255 S--FKKQ-VRR-T---L-SSCH------------------------------------------- 268
Otolemur garnettii CXNP1       257 Q--FREA-GERDA---L-SY--------------------------------------------- 269
Tupaia belangeri CXNP1         274 Q--MQEA-GEKDINDSV-SA-TDVSVPVISF---------------------------------- 299
Mus musculus Cxnp1             257 P--F------------------------------------------------------------- 258
Rattus norvegicus Cxnp1        256 P--F------------------------------------------------------------- 257
Cavia porcellus CXNP1            - -----------------------------------------------------------------   -
Oryctolagus cuniculus CXNP1      - -----------------------------------------------------------------   -
Oryctolagus cuniculus CXNP2    257 Q--FREA-GEEDTNDPV-SSCP------------------------------------------- 274
Ochotona princeps CXNP1        257 K--LREA-GEEDGSGLL-LSCP------------------------------------------- 274
Bos taurus CXNP1               258 R--PGEA-GERGSEVPL-SARP------------------------------------------- 275
Equus caballus CXNP1           257 Q--FREA-GEKGTHIPL-SSCP------------------------------------------- 274
Canis lupus familiaris CXNP1   254 Q--FQEA-GEKALASLL-LLASDVSVLMISHSRSLYL-WEAQLPHVA-KQKTRF----------- 301
Felis catus CXNP1              278 P--FREA-GEKSTDVPL-SSCS------------------------------------------- 295
Myotis lucifugus CXNP1         257 Q--FRVA-GEKSTNVPH-SSF-----LMIPSWQDPY--PPGSTTPT-CYKREKSEGIKQIQTKQN 309
Dasypus novemcinctus CXNP1     242 ----------SLSSAP--SPCA------------------------------------------- 251
Dasypus novemcinctus CXNP2     242 ----------SLSSAP--SPCA------------------------------------------- 251
Dasypus novemcinctus CXNP3     253 Q--RPEA-GEMDTGDPL-SSF---SILMISS-QVRFLSLLGSTTSTWCNL-ESQASVRCVLTTAL 308
Dasypus novemcinctus CXNP4     242 ----------SLSSAP--SPCA------------------------------------------- 251
Choloepus hoffmanni CXNP1      242 ----------ASISHS--SPSA------------------------------------------- 251
Loxodonta africana CXNP1         - -----------------------------------------------------------------   -
Homo sapiens CXNQ                - -----------------------------------------------------------------   -
Pan troglodytes CXNQ             - -----------------------------------------------------------------   -
Pongo abelii CXNQ                - -----------------------------------------------------------------   -
Macaca mulatta CXNQ              - -----------------------------------------------------------------   -
Tupaia belangeri CXNQ            - -----------------------------------------------------------------   -
Mus musculus Cxnq                - -----------------------------------------------------------------   -
Rattus norvegicus Cxnq           - -----------------------------------------------------------------   -
Cavia porcellus CXNQ             - -----------------------------------------------------------------   -
S.tridecemlineatus CXNQ          - -----------------------------------------------------------------   -
Oryctolagus cuniculus CXNQ       - -----------------------------------------------------------------   -
Bos taurus CXNQ                  - -----------------------------------------------------------------   -
Vicugna pacos CXNQ               - -----------------------------------------------------------------   -
Equus caballus CXNQ              - -----------------------------------------------------------------   -
Canis lupus familiaris CXNQ      - -----------------------------------------------------------------   -
Myotis lucifugus CXNQ            - -----------------------------------------------------------------   -
Pteropus vampyrus CXNQ           - -----------------------------------------------------------------   -
Loxodonta africana CXNQ          - -----------------------------------------------------------------   -
Homo sapiens CXNR                - -----------------------------------------------------------------   -
Pan troglodytes CXNR             - -----------------------------------------------------------------   -
Pongo abelii CXNR                - -----------------------------------------------------------------   -
Papio hamadryas CXNR             - -----------------------------------------------------------------   -
Mus musculus Cxnr                - -----------------------------------------------------------------   -
Rattus norvegicus Cxnr           - -----------------------------------------------------------------   -
Oryctolagus cuniculus CXNR       - -----------------------------------------------------------------   -
Bos taurus CXNR                  - -----------------------------------------------------------------   -
Homo sapiens CXNS                - -----------------------------------------------------------------   -
Pan troglodytes CXNS             - -----------------------------------------------------------------   -
Pongo abelii CXNS                - -----------------------------------------------------------------   -
Nomascus leucogenys CXNS         - -----------------------------------------------------------------   -
Macaca mulatta CXNS              - -----------------------------------------------------------------   -
Papio hamadryas CXNS             - -----------------------------------------------------------------   -
Otolemur garnettii CXNS          - -----------------------------------------------------------------   -
Tupaia belangeri CXNS            - -----------------------------------------------------------------   -
Mus musculus Cxns                - -----------------------------------------------------------------   -
Rattus norvegicus Cxns           - -----------------------------------------------------------------   -
Cavia porcellus CXNS             - -----------------------------------------------------------------   -
S.tridecemlineatus CXNS          - -----------------------------------------------------------------   -
```

```
Oryctolagus cuniculus CXNS      - -----------------------------------------------------------------   -
Ochotona princeps CXNS          - -----------------------------------------------------------------   -
Bos taurus CXNS                 - -----------------------------------------------------------------   -
Equus caballus CXNS             - -----------------------------------------------------------------   -
Canis lupus familiaris CXNS     - -----------------------------------------------------------------   -
Myotis lucifugus CXNS           - -----------------------------------------------------------------   -
Dasypus novemcinctus CXNS       - -----------------------------------------------------------------   -
Loxodonta africana CXNS         - -----------------------------------------------------------------   -
Homo sapiens CXNT               - -----------------------------------------------------------------   -
Pan troglodytes CXNT            - -----------------------------------------------------------------   -
Nomascus leucogenys CXNT        - -----------------------------------------------------------------   -
Mus musculus Cxnt               - -----------------------------------------------------------------   -
Rattus norvegicus Cxnt          - -----------------------------------------------------------------   -
Cavia porcellus CXNT            - -----------------------------------------------------------------   -
Oryctolagus cuniculus CXNT      - -----------------------------------------------------------------   -
Bos taurus CXNT                 - -----------------------------------------------------------------   -
Vicugna pacos CXNT              - -----------------------------------------------------------------   -
Equus caballus CXNT             - -----------------------------------------------------------------   -
Canis lupus familiaris CXNT     - -----------------------------------------------------------------   -
Myotis lucifugus CXNT           - -----------------------------------------------------------------   -
Dasypus novemcinctus CXNT       - -----------------------------------------------------------------   -
Loxodonta africana CXNT         - -----------------------------------------------------------------   -
Homo sapiens CXNU               - -----------------------------------------------------------------   -
Pan troglodytes CXNU            - -----------------------------------------------------------------   -
Pongo abelii CXNU               - -----------------------------------------------------------------   -
Nomascus leucogenys CXNU        - -----------------------------------------------------------------   -
Macaca mulatta CXNU             - -----------------------------------------------------------------   -
Mus musculus Cxnu               - -----------------------------------------------------------------   -
Cavia porcellus CXNU            - -----------------------------------------------------------------   -
Bos taurus CXNU                 - -----------------------------------------------------------------   -
Equus caballus CXNU             - -----------------------------------------------------------------   -
Myotis lucifugus CXNU           - -----------------------------------------------------------------   -
Sorex araneus CXNU              - -----------------------------------------------------------------   -
Dasypus novemcinctus CXNU       - -----------------------------------------------------------------   -
Loxodonta africana CXNU         - -----------------------------------------------------------------   -
```

```
Homo sapiens CXNA               - --------------------------------------------------------------& -
                                    980       990       1000      1010      1020      1030
Homo sapiens CXNA               - --------------------------------------------------------------& -
Pan troglodytes CXNA            - --------------------------------------------------------------& -
Gorilla gorilla CXNA            - --------------------------------------------------------------& -
Pongo abelii CXNA               - --------------------------------------------------------------& -
Nomascus leucogenys CXNA        - --------------------------------------------------------------& -
Macaca mulatta CXNA             - --------------------------------------------------------------& -
Callithrix jacchus CXNA         - --------------------------------------------------------------& -
Mus musculus Cxna               - --------------------------------------------------------------& -
Rattus norvegicus Cxna          - --------------------------------------------------------------& -
Cavia porcellus CXNA            - --------------------------------------------------------------& -
Ochotona princeps CXNA          - --------------------------------------------------------------& -
Bos taurus CXNA                 - --------------------------------------------------------------& -
Equus caballus CXNA             - --------------------------------------------------------------& -
Canis lupus familiaris CXNA     - --------------------------------------------------------------& -
Felis catus CXNA                - --------------------------------------------------------------& -
Myotis lucifugus CXNA           - --------------------------------------------------------------& -
Dasypus novemcinctus CXNA       - --------------------------------------------------------------& -
Loxodonta africana CXNA         - --------------------------------------------------------------& -
Homo sapiens CXNB               - --------------------------------------------------------------& -
Gorilla gorilla CXNB            - --------------------------------------------------------------& -
Nomascus leucogenys CXNB        - --------------------------------------------------------------& -
Macaca mulatta CXNB             - --------------------------------------------------------------& -
Callithrix jacchus CXNB         - --------------------------------------------------------------& -
Otolemur garnettii CXNB         - --------------------------------------------------------------& -
Mus musculus Cxnb               - --------------------------------------------------------------& -
Rattus norvegicus Cxnb          - --------------------------------------------------------------& -
Dipodomys ordii CXNB            - --------------------------------------------------------------& -
Cavia porcellus CXNB            - --------------------------------------------------------------& -
Oryctolagus cuniculus CXNB      - --------------------------------------------------------------& -
Ochotona princeps CXNB          - --------------------------------------------------------------& -
Bos taurus CXNB                 - --------------------------------------------------------------& -
Equus caballus CXNB             - --------------------------------------------------------------& -
Canis lupus familiaris CXNB     - --------------------------------------------------------------& -
Myotis lucifugus CXNB           - --------------------------------------------------------------& -
Erinaceus europaeus CXNB        - --------------------------------------------------------------& -
Loxodonta africana CXNB         - --------------------------------------------------------------& -
Homo sapiens CXNC               - --------------------------------------------------------------& -
Gorilla gorilla CXNC            - --------------------------------------------------------------& -
Pongo abelii CXNC               - --------------------------------------------------------------& -
Nomascus leucogenys CXNC        - --------------------------------------------------------------& -
Callithrix jacchus CXNC         - --------------------------------------------------------------& -
Microcebus murinus CXNC         - --------------------------------------------------------------& -
Otolemur garnettii CXNC         - --------------------------------------------------------------& -
Mus musculus Cxnc               - --------------------------------------------------------------& -
Rattus norvegicus Cxnc          - --------------------------------------------------------------& -
Cavia porcellus CXNC            - --------------------------------------------------------------& -
Oryctolagus cuniculus CXNC      - --------------------------------------------------------------& -
Bos taurus CXNC                 - --------------------------------------------------------------& -
Equus caballus CXNC             - --------------------------------------------------------------& -
Canis lupus familiaris CXNC     - --------------------------------------------------------------& -
Felis catus CXNC                - --------------------------------------------------------------& -
Myotis lucifugus CXNC           - --------------------------------------------------------------& -
Dasypus novemcinctus CXNC       - --------------------------------------------------------------& -
Loxodonta africana CXNC         - --------------------------------------------------------------& -
Homo sapiens CXND               - --------------------------------------------------------------& -
Pan troglodytes CXND            - --------------------------------------------------------------& -
Macaca mulatta CXND             - --------------------------------------------------------------& -
Callithrix jacchus CXND         - --------------------------------------------------------------& -
Dipodomys ordii CXND            - --------------------------------------------------------------& -
Oryctolagus cuniculus CXND      - --------------------------------------------------------------& -
Tursiops truncatus CXND         - --------------------------------------------------------------& -
Bos taurus CXND                 - --------------------------------------------------------------& -
Equus caballus CXND             - --------------------------------------------------------------& -
Canis lupus familiaris CXND     - --------------------------------------------------------------& -
Myotis lucifugus CXND           - --------------------------------------------------------------& -
Erinaceus europaeus CXND        - --------------------------------------------------------------& -
Dasypus novemcinctus CXND       - --------------------------------------------------------------& -
Choloepus hoffmanni CXND        - --------------------------------------------------------------& -
Loxodonta africana CXND         - --------------------------------------------------------------& -
Homo sapiens CXNE               - --------------------------------------------------------------& -
Pan troglodytes CXNE            - --------------------------------------------------------------& -
Pongo abelii CXNE               - --------------------------------------------------------------& -
Macaca mulatta CXNE             - --------------------------------------------------------------& -
Papio hamadryas CXNE            - --------------------------------------------------------------& -
Callithrix jacchus CXNE         - --------------------------------------------------------------& -
Otolemur garnettii CXNE         - --------------------------------------------------------------& -
Mus musculus Cxne               - --------------------------------------------------------------& -
```

Rattus norvegicus Cxne - --& -
Dipodomys ordii CXNE - --& -
Cavia porcellus CXNE - --& -
Oryctolagus cuniculus CXNE - --& -
Equus caballus CXNE - --& -
Canis lupus familiaris CXNE - --& -
Felis catus CXNE - --& -
Myotis lucifugus CXNE - --& -
Sorex araneus CXNE - --& -
Dasypus novemcinctus CXNE - --& -
Loxodonta africana CXNE - --& -
Homo sapiens CXNF - --& -
Pan troglodytes CXNF - --& -
Gorilla gorilla CXNF - --& -
Nomascus leucogenys CXNF - --& -
Callithrix jacchus CXNF - --& -
Mus musculus Cxnf - --& -
Rattus norvegicus Cxnf - --& -
S.tridecemlineatus CXNF - --& -
Oryctolagus cuniculus CXNF - --& -
Ochotona princeps CXNF - --& -
Bos taurus CXNF - --& -
Equus caballus CXNF - --& -
Canis lupus familiaris CXNF - --& -
Myotis lucifugus CXNF - --& -
Dasypus novemcinctus CXNF - --& -
Choloepus hoffmanni CXNF - --& -
Loxodonta africana CXNF - --& -
Homo sapiens CXNG - --& -
Pan troglodytes CXNG - --& -
Gorilla gorilla CXNG - --& -
Pongo abelii CXNG - --& -
Nomascus leucogenys CXNG - --& -
Macaca mulatta CXNG - --& -
Papio hamadryas CXNG - --& -
Callithrix jacchus CXNG - --& -
Microcebus murinus CXNG - --& -
Mus musculus Cxng - --& -
Rattus norvegicus Cxng - --& -
Oryctolagus cuniculus CXNG - --& -
Bos taurus CXNG - --& -
Equus caballus CXNG - --& -
Canis lupus familiaris CXNG - --& -
Felis catus CXNG - --& -
Myotis lucifugus CXNG - --& -
Pteropus vampyrus CXNG - --& -
Dasypus novemcinctus CXNG - --& -
Loxodonta africana CXNG - --& -
Procavia capensis CXNG - --& -
Homo sapiens CXNH1 - --& -
Pan troglodytes CXNH1 - --& -
Gorilla gorilla CXNH1 - --& -
Pongo abelii CXNH1 - --& -
Nomascus leucogenys CXNH1 - --& -
Macaca mulatta CXNH1 - --& -
Otolemur garnettii CXNH1 - --& -
Mus musculus Cxnh1 - --& -
Rattus norvegicus Cxnh1 - --& -
Cavia porcellus CXNH1 - --& -
Oryctolagus cuniculus CXNH1 - --& -
Ochotona princeps CXNH1 - --& -
Equus caballus CXNH1 - --& -
Canis lupus familiaris CXNH1 - --& -
Felis catus CXNH1 - --& -
Myotis lucifugus CXNH1 - --& -
Dasypus novemcinctus CXNH1 - --& -
Loxodonta africana CXNH1 - --& -
Homo sapiens CXNI - --& -
Pan troglodytes CXNI - --& -
Pongo abelii CXNI - --& -
Nomascus leucogenys CXNI - --& -
Macaca mulatta CXNI - --& -
Papio hamadryas CXNI - --& -
Callithrix jacchus CXNI - --& -
Otolemur garnettii CXNI - --& -
Mus musculus Cxni - --& -
Rattus norvegicus Cxni - --& -
Cavia porcellus CXNI - --& -
Oryctolagus cuniculus CXNI - --& -

Bos taurus CXNI	-	--&	-
Equus caballus CXNI	-	--&	-
Canis lupus familiaris CXNI	-	--&	-
Felis catus CXNI	-	--&	-
Pteropus vampyrus CXNI	-	--&	-
Erinaceus europaeus CXNI	-	--&	-
Dasypus novemcinctus CXNI	-	--&	-
Loxodonta africana CXNI	-	--&	-
Homo sapiens CXNJ1	-	--&	-
Pongo abelii CXNJ1	-	--&	-
Macaca mulatta CXNJ1	-	--&	-
Mus musculus Cxnj1	-	--&	-
Rattus norvegicus Cxnj1	-	--&	-
Bos taurus CXNJ1	-	--&	-
Equus caballus CXNJ1	-	--&	-
Myotis lucifugus CXNJ1	-	--&	-
Myotis lucifugus CXNJ2	-	--&	-
Pteropus vampyrus CXNJ1	-	--&	-
Pteropus vampyrus CXNJ2	-	--&	-
Sorex araneus CXNJ1	-	--&	-
Homo sapiens CXNK1	-	--&	-
Homo sapiens CXNK2	-	--&	-
Pan troglodytes CXNK1	-	--&	-
Pan troglodytes CXNK2	-	--&	-
Pongo abelii CXNK1	-	--&	-
Nomascus leucogenys CXNK1	-	--&	-
Callithrix jacchus CXNK1	-	--&	-
Mus musculus Cxnk1	-	--&	-
Mus musculus Cxnk2	-	--&	-
Rattus norvegicus Cxnk1	-	--&	-
Rattus norvegicus Cxnk2	-	--&	-
Cavia porcellus CXNK1	-	--&	-
Oryctolagus cuniculus CXNK1	-	--&	-
Bos taurus CXNK1	-	--&	-
Vicugna pacos CXNK1	-	--&	-
Equus caballus CXNK1	-	--&	-
Equus caballus CXNK2	-	--&	-
Canis lupus familiaris CXNK1	-	--&	-
Canis lupus familiaris CXNK2	-	--&	-
Felis catus CXNK1	-	--&	-
Myotis lucifugus CXNK1	-	--&	-
Dasypus novemcinctus CXNK1	-	--&	-
Dasypus novemcinctus CXNK2	-	--&	-
Loxodonta africana CXNK1	-	--&	-
Loxodonta africana CXNK2	-	--&	-
Homo sapiens CXNL	-	--&	-
Pan troglodytes CXNL	-	--&	-
Pongo abelii CXNL	-	--&	-
Nomascus leucogenys CXNL	-	--&	-
Macaca mulatta CXNL	-	--&	-
Callithrix jacchus CXNL	-	--&	-
Otolemur garnettii CXNL	-	--&	-
Mus musculus Cxnl	-	--&	-
Rattus norvegicus Cxnl	-	--&	-
Cavia porcellus CXNL	-	--&	-
Oryctolagus cuniculus CXNL	-	--&	-
Ochotona princeps CXNL	-	--&	-
Bos taurus CXNL	-	--&	-
Equus caballus CXNL	-	--&	-
Canis lupus familiaris CXNL	-	--&	-
Felis catus CXNL	-	--&	-
Pteropus vampyrus CXNL	-	--&	-
Sorex araneus CXNL	-	--&	-
Dasypus novemcinctus CXNL	-	--&	-
Loxodonta africana CXNL	-	--&	-
Homo sapiens CXNM	-	--&	-
Pan troglodytes CXNM	-	--&	-
Pongo abelii CXNM	-	--&	-
Nomascus leucogenys CXNM	-	--&	-
Macaca mulatta CXNM	-	--&	-
Callithrix jacchus CXNM	-	--&	-
Tarsius syrichta CXNM	-	--&	-
Microcebus murinus CXNM	-	--&	-
Dipodomys ordii CXNM	-	--&	-
Oryctolagus cuniculus CXNM	-	--&	-
Equus caballus CXNM	-	--&	-
Canis lupus familiaris CXNM	-	--&	-
Pteropus vampyrus CXNM	-	--&	-
Loxodonta africana CXNM	-	--&	-

```
Homo sapiens CXNN              503 FPFFLPGVCMYVCVDREAD-----GGGDYLWRDKIIHSIHSVKF-NS---------------& 543
Pan troglodytes CXNN           503 FPFFLPGVCMYVCVDREAD-----GGGDYLWRDKIIHSIHSVKF-NS---------------& 543
Pongo abelii CXNN              503 FPFFLRGVCMYVFVDREAD-----GEGDYLWRDKIIHSILLVKF-NS---------------& 543
Nomascus leucogenys CXNN       503 FPFFLPGVCMYVCVDREAD-----GEGDYLWREKIIHSIHSVKF-NS---------------& 543
Macaca mulatta CXNN            503 FPFFLPGVCMYVSVDREAD-----GEGDYLWRDKIIHSIHSVKF-NS---------------& 543
Callithrix jacchus CXNN        504 LSSLLGCVCMFVVTERQMEREIIYGEIKLFIQYIQLNSIHKINLENTYFIPSTLISTGHRNG& 565
Microcebus murinus CXNN        499 LSSFLACIC----VEREMD-------GR-LI-YGAIHSIHSVKF-NS---------------& 531
Mus musculus Cxnn              503 YVY-----------------------------------------------------------& 505
Rattus norvegicus Cxnn         504 YVY-----------------------------------------------------------& 506
Oryctolagus cuniculus CXNN     530 LSSFLLCVCVCVCVQRSGQRGD----------------------------------------& 551
Bos taurus CXNN                  - --------------------------------------------------------------&   -
Equus caballus CXNN              - --------------------------------------------------------------&   -
Canis lupus familiaris CXNN      - --------------------------------------------------------------&   -
Sorex araneus CXNN               - --------------------------------------------------------------&   -
Loxodonta africana CXNN          - --------------------------------------------------------------&   -
Homo sapiens CXNO                - --------------------------------------------------------------&   -
Papio hamadryas CXNO             - --------------------------------------------------------------&   -
Mus musculus Cxno                - --------------------------------------------------------------&   -
Cavia porcellus CXNO             - --------------------------------------------------------------&   -
Homo sapiens CXNP1               - --------------------------------------------------------------&   -
Pan troglodytes CXNP1            - --------------------------------------------------------------&   -
Pongo abelii CXNP1               - --------------------------------------------------------------&   -
Callithrix jacchus CXNP1         - --------------------------------------------------------------&   -
Otolemur garnettii CXNP1         - --------------------------------------------------------------&   -
Tupaia belangeri CXNP1           - --------------------------------------------------------------&   -
Mus musculus Cxnp1               - --------------------------------------------------------------&   -
Rattus norvegicus Cxnp1          - --------------------------------------------------------------&   -
Cavia porcellus CXNP1            - --------------------------------------------------------------&   -
Oryctolagus cuniculus CXNP1      - --------------------------------------------------------------&   -
Oryctolagus cuniculus CXNP2      - --------------------------------------------------------------&   -
Ochotona princeps CXNP1          - --------------------------------------------------------------&   -
Bos taurus CXNP1                 - --------------------------------------------------------------&   -
Equus caballus CXNP1             - --------------------------------------------------------------&   -
Canis lupus familiaris CXNP1     - --------------------------------------------------------------&   -
Felis catus CXNP1                - --------------------------------------------------------------&   -
Myotis lucifugus CXNP1         310 NTS-----------------------------------------------------------& 312
Dasypus novemcinctus CXNP1       - --------------------------------------------------------------&   -
Dasypus novemcinctus CXNP2       - --------------------------------------------------------------&   -
Dasypus novemcinctus CXNP3     309 NLSPGPVPTTVTFPSNEPGSE-----------------------------------------& 329
Dasypus novemcinctus CXNP4       - --------------------------------------------------------------&   -
Choloepus hoffmanni CXNP1        - --------------------------------------------------------------&   -
Loxodonta africana CXNP1         - --------------------------------------------------------------&   -
Homo sapiens CXNQ                - --------------------------------------------------------------&   -
Pan troglodytes CXNQ             - --------------------------------------------------------------&   -
Pongo abelii CXNQ                - --------------------------------------------------------------&   -
Macaca mulatta CXNQ              - --------------------------------------------------------------&   -
Tupaia belangeri CXNQ            - --------------------------------------------------------------&   -
Mus musculus Cxnq                - --------------------------------------------------------------&   -
Rattus norvegicus Cxnq           - --------------------------------------------------------------&   -
Cavia porcellus CXNQ             - --------------------------------------------------------------&   -
S.tridecemlineatus CXNQ          - --------------------------------------------------------------&   -
Oryctolagus cuniculus CXNQ       - --------------------------------------------------------------&   -
Bos taurus CXNQ                  - --------------------------------------------------------------&   -
Vicugna pacos CXNQ               - --------------------------------------------------------------&   -
Equus caballus CXNQ              - --------------------------------------------------------------&   -
Canis lupus familiaris CXNQ      - --------------------------------------------------------------&   -
Myotis lucifugus CXNQ            - --------------------------------------------------------------&   -
Pteropus vampyrus CXNQ           - --------------------------------------------------------------&   -
Loxodonta africana CXNQ          - --------------------------------------------------------------&   -
Homo sapiens CXNR                - --------------------------------------------------------------&   -
Pan troglodytes CXNR             - --------------------------------------------------------------&   -
Pongo abelii CXNR                - --------------------------------------------------------------&   -
Papio hamadryas CXNR             - --------------------------------------------------------------&   -
Mus musculus Cxnr                - --------------------------------------------------------------&   -
Rattus norvegicus Cxnr           - --------------------------------------------------------------&   -
Oryctolagus cuniculus CXNR       - --------------------------------------------------------------&   -
Bos taurus CXNR                  - --------------------------------------------------------------&   -
Homo sapiens CXNS                - --------------------------------------------------------------&   -
Pan troglodytes CXNS             - --------------------------------------------------------------&   -
Pongo abelii CXNS                - --------------------------------------------------------------&   -
Nomascus leucogenys CXNS         - --------------------------------------------------------------&   -
Macaca mulatta CXNS              - --------------------------------------------------------------&   -
Papio hamadryas CXNS             - --------------------------------------------------------------&   -
Otolemur garnettii CXNS          - --------------------------------------------------------------&   -
Tupaia belangeri CXNS            - --------------------------------------------------------------&   -
Mus musculus Cxns                - --------------------------------------------------------------&   -
Rattus norvegicus Cxns           - --------------------------------------------------------------&   -
Cavia porcellus CXNS             - --------------------------------------------------------------&   -
S.tridecemlineatus CXNS          - --------------------------------------------------------------&   -
```

Oryctolagus cuniculus CXNS - --& -
Ochotona princeps CXNS - --& -
Bos taurus CXNS - --& -
Equus caballus CXNS - --& -
Canis lupus familiaris CXNS - --& -
Myotis lucifugus CXNS - --& -
Dasypus novemcinctus CXNS - --& -
Loxodonta africana CXNS - --& -
Homo sapiens CXNT - --& -
Pan troglodytes CXNT - --& -
Nomascus leucogenys CXNT - --& -
Mus musculus Cxnt - --& -
Rattus norvegicus Cxnt - --& -
Cavia porcellus CXNT - --& -
Oryctolagus cuniculus CXNT - --& -
Bos taurus CXNT - --& -
Vicugna pacos CXNT - --& -
Equus caballus CXNT - --& -
Canis lupus familiaris CXNT - --& -
Myotis lucifugus CXNT - --& -
Dasypus novemcinctus CXNT - --& -
Loxodonta africana CXNT - --& -
Homo sapiens CXNU - --& -
Pan troglodytes CXNU - --& -
Pongo abelii CXNU - --& -
Nomascus leucogenys CXNU - --& -
Macaca mulatta CXNU - --& -
Mus musculus Cxnu - --& -
Cavia porcellus CXNU - --& -
Bos taurus CXNU - --& -
Equus caballus CXNU - --& -
Myotis lucifugus CXNU - --& -
Sorex araneus CXNU - --& -
Dasypus novemcinctus CXNU - --& -
Loxodonta africana CXNU - --& -
